# Detecting departures from the conditional independence assumption in diagnostic latent class models: a simulation study

**DOI:** 10.1186/s12874-024-02432-x

**Published:** 2024-12-05

**Authors:** Yasin Okkaoglu, Nicky J. Welton, Hayley E. Jones

**Affiliations:** Population Health Sciences, Bristol Medical School, University of Bristol, Bristol, UK

**Keywords:** Latent class model, Diagnostic accuracy, Conditional independence, Model selection, Goodness of fit, Residual correlation plots

## Abstract

**Background:**

Latent class models can be used to estimate diagnostic accuracy without a gold standard test. Early studies often assumed independence between tests given the true disease state, however this can lead to biased estimates when there are inter-test dependencies. Residual correlation plots and chi-squared statistics have been commonly utilized to assess the validity of the conditional independence assumption and, when it does not hold, identify which test pairs are conditionally dependent. We aimed to assess the performance of these tools with a simulation study covering a wide range of scenarios.

**Methods:**

We generated data sets from a model with four tests and a dependence between tests 1 and 2 within the diseased group. We varied sample size, prevalence, covariance, sensitivity and specificity, with 504 combinations of these in total, and 1000 data sets for each combination. We fitted the conditional independence model in a Bayesian framework, and reported absolute bias, coverage, and how often the residual correlation plots, ${G}^{2}$ and ${\chi }^{2}$ statistics indicated lack-of-fit globally or for each test pair.

**Results:**

Across all settings, residual correlation plots, pairwise ${G}^{2}$ and ${\chi }^{2}$ detected the correct correlated pair of tests only 12.1%, 10.3%, and 10.3% of the time, respectively, but incorrectly suggested dependence between tests 3 and 4 64.9%, 49.7%, and 49.5% of the time. We observed some variation in this across parameter settings, with these tools appearing to perform more as intended when tests 3 and 4 were both much more accurate than tests 1 and 2. Residual correlation plots, ${G}^{2}$ and ${\chi }^{2}$ statistics identified a lack of *overall* fit in 74.3%, 64.5% and 67.5% of models, respectively. The conditional independence model tended to overestimate the sensitivities of the correlated tests (median bias across all scenarios 0.094, 2.5th and 97.5th percentiles -0.003, 0.397) and underestimate prevalence and the specificities of the uncorrelated tests.

**Conclusions:**

Residual correlation plots and chi-squared statistics cannot be relied upon to identify which tests are conditionally dependent, and also have relatively low power to detect lack of overall fit. This is important since failure to account for conditional dependence can lead to highly biased parameter estimates.

**Supplementary Information:**

The online version contains supplementary material available at 10.1186/s12874-024-02432-x.

## Background

Latent Class Models (LCMs) have been proposed to estimate diagnostic test accuracy and the prevalence of a target condition when there is no gold standard test available [1]. LCMs treat the true condition status of subjects as a latent (unobserved) variable and estimate the accuracy of tests by establishing a probabilistic relationship between this latent variable and each combination of diagnostic test results on the same subjects. Early work on LCMs assumed that results of different diagnostic tests on the same subjects were independent of each other, conditional on the subject’s true condition status (i.e. Conditional Independence (CInd) assumption) [1–3]. However, the CInd assumption may often not hold in practice as different tests often work in a similar manner, e.g. measure the same biomarker, or the sensitivity of all tests could depend on the severity of the target condition [4].

Ignoring conditional dependence can yield biased estimates of accuracy (typically quantified as sensitivity and specificity) and prevalence [5–7]. Various types of LCMs have been proposed that allow for some conditional dependencies between tests [6, 8–10]. In cases where the CInd assumption does not hold, the analyst needs to know which tests are conditionally dependent (CDep) in order to account for this in the model. Ideally, the CDep structure would be pre-specified based on knowledge of how the diagnostic tests under evaluation work. Subsequently, dependence terms can be incorporated into the model for these correlations [9, 11–13]. However, this approach may not always be feasible. It is also generally not feasible to include all potential pairwise dependencies simultaneously, as doing so will typically lead to non-identifiability problems, heightened computational complexity, and increased uncertainty in the model [14]. An alternative approach is to first fit a CInd model and then use measures of its fit to assess whether CDep is present and, if so, which tests are conditionally dependent [10, 14].

Several tools have been suggested and utilized to assess the lack of overall fit of the CInd model, including Pearson’s ${\chi }^{2}$ and likelihood-ratio $\left({G}^{2}\right)$ goodness of fit statistics [14–24]. In some studies, the overall fit of the model has also been examined using pairwise versions of these statistics. Specifically, the fitted model is considered to show a good overall fit when there is no evidence of lack of fit for any pairs [15, 17, 25]. Qu et al. [10] proposed use of residual correlation plots, which display the differences between the observed and fitted Pearson correlations for each pair of tests. When a model provides an adequate fit, these residual correlations are expected to be scattered randomly around zero [10]. Although Qu et al. [10] originally intended to use residual correlation plots to identify unexplained pairwise dependencies in the fitted model to aid in model selection, these plots have also been employed as a measure of overall goodness of fit in several studies [14, 24, 26–28]. Pairwise ${\chi }^{2}$ and ${G}^{2}$ statistics, as well as residual correlation plots, have also been adopted to identify underlying conditional dependencies between diagnostic tests that are not explained by a fitted LCM [10, 14, 15, 19, 21, 23, 29–32]. Based on the results of these, dependency terms can be added to the model for pairs exhibiting evidence of misfit, aiming to account for correlations unexplained by the CInd model [10, 14, 21, 23, 29–32].

Despite the widespread use of these goodness-of-fit measures for latent class models in diagnostic accuracy research, there is limited knowledge of their performance. Van Smeden et al. [33] demonstrated that the overall (asymptotic and parametric bootstrapped) ${\chi }^{2}$ and ${G}^{2}$ tests can have low power to detect violation of the CInd assumption for data with similar characteristics to three data sets from the literature [34–36] when the sample size is low. Of even more concern is an unintuitive finding by Subtil et al. [37]. They simulated from models with a single conditional dependence term between two diagnostic tests and demonstrated that residual correlation plots had a tendency to suggest dependence between the wrong (uncorrelated) pair of diagnostic tests. In addition, the residual correlation plots, χ^2^, and G^2^ statistics were able to detect departures from the CInd assumption at most approximately 50% of the time across the simulated data sets.

In this simulation study, we aimed to investigate whether the concerning findings reported by Subtil et al. [37] extend across a much broader range of parameter settings and sample sizes. We also quantify bias in estimates of sensitivity and specificity from the CInd model for each parameter combination, and consider the relationship between this and the performance of tools to detect violation of the CInd assumption.

## Methods

### Conditional independence latent class model

Let ${T}_{i}={t}_{i}$, $i=1,\dots ,{n}_{tests}$, denote the diagnostic test result of the $i$ th test, where ${t}_{i}=0$ indicates a negative result and ${t}_{i}=1$ corresponds to a positive test result. Then the CInd model can be given as [1]:

1 $$P\left({T}_{1}={t}_{1},{T}_{2}={t}_{2},\dots ,{T}_{{n}_{tests}}={t}_{{n}_{tests}}\right)=\pi \prod_{i=1}^{{n}_{tests}}{Se}_{i}^{{t}_{i}}{\left(1-{Se}_{i}\right)}^{1-{t}_{i}}+\left(1-\pi \right)\prod_{i=1}^{{n}_{tests}}{Sp}_{i}^{1-{t}_{i}}{\left(1-{Sp}_{i}\right)}^{{t}_{i}}$$

where $\pi$ denotes the prevalence of the target condition in the study population ($P\left(D=1\right)$); ${Se}_{i}$ denotes the sensitivity of the $i$ th test ($P\left({T}_{i}=1|D=1\right)$); and ${Sp}_{i}$ denotes the specificity of the $i$ th test ($P\left({T}_{i}=0|D=0\right)$); where $D$ is the latent target condition status of a subject, taking the value 1 when the condition is present and 0 when the condition is absent.

### Methods to detect lack of fit

The residual correlation plot [10] shows the residual correlation, often plotted with 95% confidence intervals (CIs), for each pair of tests. Large residual correlations, or those with 95% CI not including zero, are used to identify areas in which the current model does not fit well. Let ${P}_{obs}\left(A\right)$ and ${P}_{M}\left(A\right)$ denote the observed proportions and fitted probabilities of event A, respectively. The residual correlation between tests $i$ and $j$ is defined as

2 $${\rho }_{ij}={r}_{ij}-{r}_{ij}^{M}$$

where ${r}_{ij}$ is the observed marginal correlation between tests $i$ and $j$, given by

3 $${r}_{ij}=\frac{{P}_{obs}\left({T}_{i}=1,{T}_{j}=1\right)-{P}_{obs}\left({T}_{i}=1\right){P}_{obs}\left({T}_{j}=1\right)}{\sqrt{{P}_{obs}\left({T}_{i}=1\right)\left(1-{P}_{obs}\left({T}_{i}=1\right)\right){P}_{obs}\left({T}_{j}=1\right)\left(1-{P}_{obs}\left({T}_{j}=1\right)\right)}}$$

and ${r}_{ij}^{M}$ is the fitted marginal correlation from the LCM, given by

4 $${r}_{ij}^{M}=\frac{{P}_{M}\left({T}_{i}=1,{T}_{j}=1\right)-{P}_{M}\left({T}_{i}=1\right){P}_{M}\left({T}_{j}=1\right)}{\sqrt{{P}_{M}\left({T}_{i}=1\right)\left(1-{P}_{M}\left({T}_{i}=1\right)\right){P}_{M}\left({T}_{j}=1\right)\left(1-{P}_{M}\left({T}_{j}=1\right)\right)}}.$$

Within a frequentist framework, ${r}_{ij}^{M}$ can be obtained by plugging the fitted probabilities into Eq. (4). In this study, we fit all models in a Bayesian framework and compute all fitted and residual correlations at each step of the Markov Chain Monte Carlo (MCMC) simulations. We then calculate the posterior median of each residual correlation and 95% credible intervals.

Pearson ${\chi }^{2}$ and likelihood ratio ${G}^{2}$ goodness of fit statistics are also commonly used methods for evaluating the lack of fit of a diagnostic LCM [14–24]. These are given by

5 $$\begin{array}{c}{\chi }^{2}=\sum\limits_{k=1}^{{{2}{n}_{tests}}}\frac{{\left({O}_{k}-{E}_{k}\right)}^{2}}{{E}_{k}}\\ {G}^{2}=2\sum\limits_{k=1}^{{{2}{n}_{tests}}}{O}_{k}\text{log}\left(\frac{{O}_{k}}{{E}_{k}}\right)\end{array}$$

where ${O}_{k}$ and ${E}_{k}$, $k=1,\dots ,{2}^{{n}_{tests}}$, are the observed and fitted frequencies of each combination of test results. We will refer to these as overall ${\chi }^{2}$ and ${G}^{2}$ statistics. ${\chi }^{2}$ and ${G}^{2}$ statistics estimated from 2 × 2 pairwise agreement tables have also been used for examining the adequacy of an LCM, pinpointing areas of lack of fit, and aiding in decisions regarding the inclusion of conditional dependence terms. We will refer to these as pairwise ${\chi }^{2}$ and ${G}^{2}$ statistics.

In a frequentist framework, these statistics are referred to the corresponding critical values of a ${\chi }^{2}$ distribution with degrees of freedom ($df$) equal to ${2}^{{n}_{tests}}-p-1$ for the ‘overall’ statistics, where $p$ is the number of unconstrainedly estimated parameters [38], and $df=1$ for the pairwise tests. In a Bayesian framework, each ${E}_{k}$ and hence ${\chi }^{2}$ and ${G}^{2}$ has a posterior distribution, and there are multiple options for how these might be summarised and used. To ensure our results are comparable with previous studies using a frequentist approach [33, 37], we evaluated Eq. (5) at the posterior medians of the underlying parameter estimates (prevalence, sensitivities and specificities) rather than at each iteration of the MCMC simulations, and referred these statistics to the critical values described above.

### Simulation study

This simulation study is presented using the ADEMP (Aims, Data-generating mechanisms, Estimands, Methods, Performance Measures) framework [39].

#### Aim

The aim of this simulation study was to evaluate the performance of global and pairwise goodness-of-fit tools commonly used to assess the fit of diagnostic latent class models. Specific aims were:

- To assess the performance of residual correlation plots, overall ${\chi }^{2}$ and ${G}^{2}$ goodness of fit statistics in detecting departures from the CInd assumption.
- To explore whether residual correlation plots, pairwise ${\chi }^{2}$ and ${G}^{2}$ statistics can correctly identify the correlated pair violating the CInd assumption, and the false positive rate of these tools.
- To quantify the bias in and 95% coverage of the estimates of sensitivity, specificity and prevalence when CInd is incorrectly assumed.

#### Data generating mechanism

We simulated results of four binary diagnostic tests, under an assumption that only the first two tests were correlated within the diseased group ($D=1$). We simulated data from the same two-class ‘fixed effect’ CDep model used in the previous simulation study of Subtil et al. [37], which has also been used by others (e.g. [9]). The model is specified as:

6 $$P\left({T}_{1}={t}_{1},{T}_{2}={t}_{2},{T}_{3}={t}_{3},{T}_{4}={t}_{4}\right)=\pi \left\{\prod_{i=1}^{2}{Se}_{i}^{{t}_{i}}{\left(1-{Se}_{i}\right)}^{1-{t}_{i}}+{\left(-1\right)}^{{t}_{1}-{t}_{2}}{covse}_{12}\right\} \times \prod_{i=3}^{4}{Se}_{i}^{{t}_{i}}{\left(1-{Se}_{i}\right)}^{1-{t}_{i}}+\left(1-\pi \right)\prod_{i=1}^{4}{Sp}_{i}^{{1-t}_{i}}{\left(1-{Sp}_{i}\right)}^{{t}_{i}}$$

where ${covse}_{12}=Cov\left({T}_{1},{T}_{2}|D=1\right)$ is the covariance, assumed to be positive, between tests 1 and 2 within the diseased class. Data were generated from a multinomial likelihood using these probabilities (6).

This CDep model (6) implies a Pearson correlation between ${T}_{1}$ and ${T}_{2}$ within the diseased group of magnitude

7 $$Corr\left({T}_{1},{T}_{2}|D=1\right)=\frac{{covse}_{12}}{\sqrt{{Se}_{1}\left(1-{Se}_{1}\right){Se}_{2}\left(1-{Se}_{2}\right)}}$$

while assuming conditional independence for all other pairs within the diseased group and for all pairs within the disease-free group.

We simulated data with three distinct sample sizes, i.e. number of study participants (${n}_{obs}$): 500, 2000, and 5000. We assumed two different values for prevalence: $0.2$ and $0.5$. We considered two values for sensitivities and specificities: $0.6$ (‘low’, L) and $0.9$ (‘high’, H). The sensitivity and specificity combinations used for generating data are presented in Tables 1 and 2, respectively. To keep the number of parameter settings manageable, we only examined scenarios where both ${Se}_{1}$ and ${Se}_{2}$ are simultaneously high or low. All potential specificity combinations were included.

**Table 1:** Combinations of sensitivity ($Se$) assumed in the data generating mechanism

${Se}_{1}$	${Se}_{2}$	${Se}_{3}$	${Se}_{4}$
H	H	L	L
H	H	H	L
H	H	H	H
L	L	L	L
L	L	H	L
L	L	H	H

*H* High (0.9), *L* Low (0.6)

**Table 2:** Combinations of specificity ($Sp$) assumed in the data generating mechanism

${Sp}_{1}$	${Sp}_{2}$	${Sp}_{3}$	${Sp}_{4}$
H	H	L	L
H	H	H	L
H	H	H	H
L	L	L	L
L	L	H	L
L	L	H	H
L	H	L	H

*H* High (0.9) *L* Low (0.6)

The positive covariance term ${covse}_{12}$ is bounded to ensure the probability of each combination of test results (6) is less than or equal to 1, and this leads to an upper limit for the covariance term given by

8 $${covse}_{12}\le min\left({Se}_{1},{Se}_{2}\right)-{Se}_{1}{Se}_{2}.$$

For each ${Se}_{1}$, ${Se}_{2}$ combination, we set ${covse}_{12}$ as a proportion ($\omega$) of the maximum possible covariance term:

9 $${covse}_{12}=\omega *\left\{min\left({Se}_{1},{Se}_{2}\right)-{Se}_{1}{Se}_{2}\right\},$$

where $\omega =\text{0.5,0.9}$. As ${Se}_{1}={Se}_{2}$ in all scenarios considered, $\omega$ can also be interpreted as the Pearson correlation between ${T}_{1}$ and ${T}_{2}$ within the diseased state (7).

With three different sample sizes, two prevalences, six combinations of sensitivities, seven combinations of specificities, and two covariances, there were 504 parameter combinations in total.

In determining the number of simulations for each scenario, we considered the length of $95\%$ Monte Carlo confidence intervals (MC CI) around estimates of the proportion, *p*, of instances where a tool indicates evidence of lack of fit. We estimated the required number of simulations (${n}_{req}$) to produce a $95\%$ MC CI of maximum length *L* using the formula [40]

10 $${n}_{req}={\left(\frac{{z}_{0.025}\sqrt{p\left(1-p\right)}}{L/2}\right)}^{2}$$

where ${z}_{0.025}$ is the 2.5th percentile of the standard normal distribution and $p\left(1-p\right)$ is the variance of the binomial proportion. We set *L* = 0.05. We considered a scenario in which *p* is 0.8, for which the required number of simulations would be 983. Therefore, we set the number of simulations (${n}_{sim}$) as 1000. The required number of simulations for different values of $p$ is provided in Additional file 1.

#### Estimands

We summarised posterior distributions of the following parameters or quantities, using posterior medians and 95% credible intervals (CrIs):

- Pairwise residual correlations,
- Sensitivities and specificities of all tests,
- Prevalence of the target condition.

We calculated the overall and pairwise ${\chi }^{2}$ and ${G}^{2}$ goodness of fit statistics at the posterior median values of prevalence, sensitivities, and specificities.

We also obtained the posterior median fitted pairwise agreement probabilities (${P}_{M}\left({T}_{i}={T}_{j}=1\right)+{P}_{M}\left({T}_{i}={T}_{j}=0\right)$) to explore how the CInd model (1) fits pairwise agreements in different scenarios.

#### Methods

The CInd model (1) was fitted to all 504,000 data sets (1000 data sets for each of the 504 parameter combinations) simulated from the CDep model (6) in JAGS (Just Another Gibbs Sampler) through the R software [41], using the “runjags” [42] package. All models were fitted using vague priors ($Beta\left(\text{1,1}\right))$ for prevalence, and all sensitivities and specificities. Additionally, the constraint ${Se}_{i}\ge 1-{Sp}_{i}$, $i=\text{1,2},\text{3,4}$, was enforced to avoid a potential label-switching (mirror) issue [7]. This constraint ensures that false positive and false negative fractions are not incorrectly estimated as specificities and sensitivities. R and JAGS code to simulate data with a particular combination of parameter values and fit the CInd model is provided in Additional file 2.

For all data sets, the numbers of chains, burn-in samples and iterations after burn-in were set to 3, 5000 and 10,000, respectively. The convergence of the models was checked using $\widehat{R}$ statistics and the effective sample size (${n}_{eff}$). Any data sets providing either an $\widehat{R}$ greater than $1.1$ [43] and/or an ${n}_{eff}$ lower than 400 (equivalent to a Monte Carlo (MC) error greater than $5\%$ of the posterior standard deviation) [44] for any model parameter were excluded from the analysis.

#### Performance measures

We employed the following tool-specific strategies to assess the performance of the tools in detecting lack of fit:

##### Residual correlation plot

For each test pair, we assessed whether the 95% CrI around the residual correlation contained zero. If it did not, we considered there to be evidence of a lack of pairwise fit of the CInd model for that pair. Additionally, if the residual correlations indicated lack of pairwise fit for *any* test pair, we considered the tool to have suggested a lack of global fit. That is, we evaluated (across the simulated data sets for each scenario with converged results):

- Percentage of instances where the 95% CrIs around *any* of the residual correlations did not include zero.
- Percentage of instances where the 95% CrI around *each* residual correlation did not include zero.

##### Pairwise ${\chi }^{2}$ and ${G}^{2}$ tests

For each test pair, we calculated the percentage of data sets for which the test statistic was greater than the 95% critical value of a ${\chi }_{1}^{2}$ distribution.

##### Overall ${\chi }^{2}$ and ${G}^{2}$ tests

Similar to the pairwise version of these statistics, we computed the percentage of instances where these test statistics were greater than the 95% critical value of a ${\chi }_{6}^{2}$ distribution, where $df$ = ${2}^{4}-9-1$ = $6.$

For each scenario, we also quantified the mean absolute bias of estimates (posterior medians) of sensitivity, specificity and prevalence, across the simulated data sets with converged results, and the coverage of 95% CrIs for these parameters. For all percentages, average biases and coverages, 95% MC CIs were also calculated. For proportions, Clopper-Peason confidence intervals [45] were used, while for average biases and coverages we adopted the $estimates\pm {(Z}_{0.025}\times MCSE)$ formula, where $MCSE$ denotes the Monte Carlo standard errors given by Morris et al. [39].

Given the large number of parameter combinations, in addition to producing results for each parameter combination separately we also evaluated the performance measures above across all converged data sets (of the 504,000), across all converged data sets with each value of prevalence ($\pi$) or correlation ($\omega$), and across all with sample size of 500 or 5000. In these summaries, we described biases using medians with 2.5th and 97th percentiles.

## Results

Out of 504,000 simulated data sets, the CInd model did not meet our convergence criteria for 30,632 (6.1%) data sets, and these data sets were excluded from the analysis.

We present summaries of results across scenarios, and results for 14 of the 504 parameter combinations as illustrative examples. These were selected post hoc to demonstrate the role of the combinations of true sensitivity and specificity in bias and tool performance. The 14 combinations have the same prevalence ($\pi =0.5$), sample size (${n}_{obs}=2000$) and covariance ($\omega =0.5$) but varying sensitivity (two sensitivity combinations: HHHH, LLHH) and specificity (all specificity combinations given in Table 2). The percentage of converged models (out of 1000 fitted models) varied across these 14 sets of parameter combinations. For all seven combinations with sensitivity HHHH, at least 98.5% of the models converged. However, only 61.7% and 79.8% of the 1000 fitted CInd models converged when the sensitivity–specificity combinations were LLHH-LLHL and LLHH-LHLH, respectively.

Complete results, including biases of the parameter estimates, 95% CrI coverages for the parameters, tool performance percentages, and numbers of the converged data sets for each of the 504 parameter combinations, are available in Additional File 3.

### Bias and coverage

Percentiles of absolute biases (2.5th, 50th, 97.5th) for the model parameters across all data sets in different scenarios are available in Additional file 4. As we might anticipate, the median bias across all data sets was greatest in the estimates of ${Se}_{1}$ and ${Se}_{2}$ (i.e., sensitivities of the correlated tests). These sensitivities were overestimated by a median of 0.094 (2.5th and 97.5th percentiles: -0.003, 0.397). The CInd model tended to overestimate the sensitivity of ${T}_{1}$ and ${T}_{2}$ but also underestimate the prevalence and the specificities of ${T}_{3}$ and ${T}_{4}$. The magnitude of these biases was greatest when prevalence and correlation were higher. For example, across all converged models for data sets with $\omega =0.9$, ${Se}_{1}$ and ${Se}_{2}$ were over-estimated by a median of 0.098. In contrast, when $\omega =0.5$, the biases were lower, with median bias of 0.067 and 0.070, respectively.

**Fig. 1:**
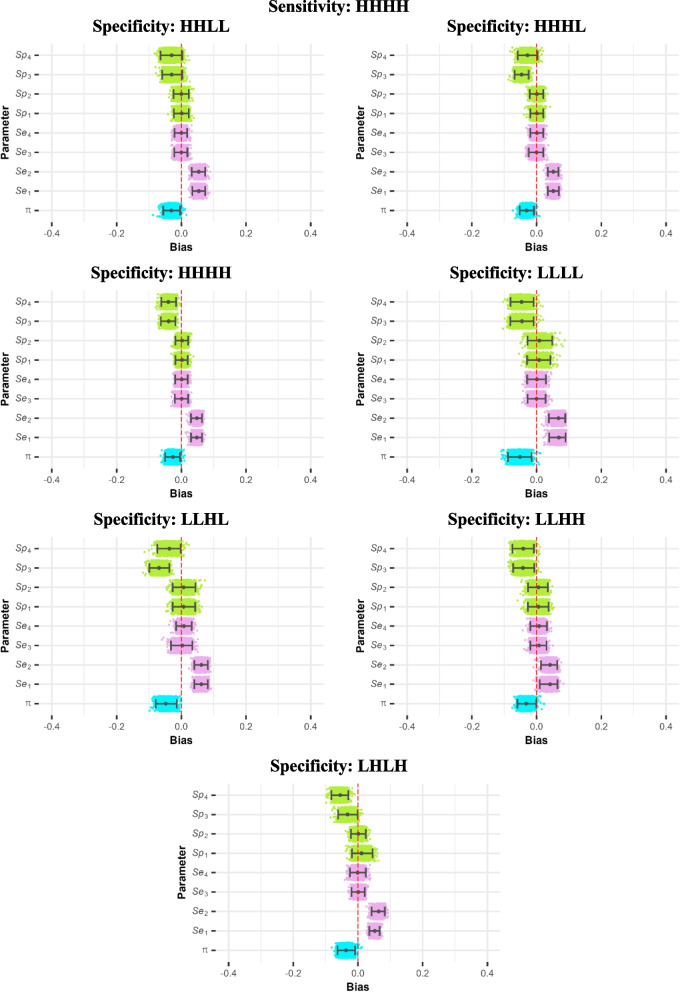
Bias jitter plots for the combinations with sensitivity = HHHH and varying specificities ($\uppi =0.5$, $\upomega =0.5$, ${\text{n}}_{\text{obs}}=2000$). The grey points represent median bias, and the error bars show 2.5th and 97.5th percentiles. The blue, pink, and green points represent bias in prevalence, sensitivity, and specificity estimates, respectively

**Fig. 2:**
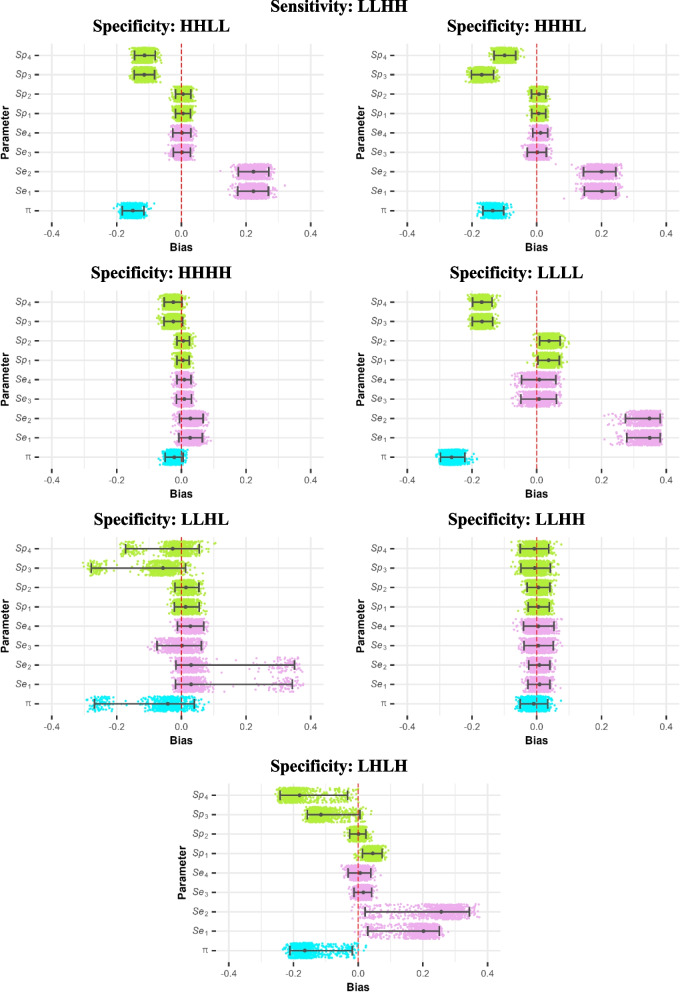
Bias jitter plots for the combinations with sensitivity = LLHH and varying specificities ($\uppi =0.5$, $\upomega =0.5$, ${\text{n}}_{\text{obs}}=2000$). The grey points represent median bias, and the error bars show 2.5th and 97.5th percentiles. The blue, pink, and green points represent bias in prevalence, sensitivity, and specificity estimates, respectively

Figures 1 and 2 demonstrate that the extent of bias varied by sensitivity–specificity combination. Jitter plots were used to represent biases in each parameter estimate, allowing for a comparison of the magnitudes of biases for the same parameter across different diagnostic tests within the same sensitivity–specificity combination, as well as between different sensitivity–specificity combinations. The y-axis spread within the bar for each parameter is arbitrary, to provide some separation between points [39]. When sensitivity = HHHH, the extent of bias was similar across different combinations of specificity: the CInd model overestimated sensitivities of the correlated pair (${T}_{1}$ and ${T}_{2}$) and underestimated the prevalence and specificities of ${T}_{3}$ and ${T}_{4}$ (Fig. 1). However, when sensitivity = LLHH, biases in estimates of prevalence, sensitivities of ${T}_{1}$ and ${T}_{2}$ and specificities of ${T}_{3}$ and ${T}_{4}$ differed considerably according to the tests’ specificities (Fig. 2). Sensitivity–specificity combinations LLHH-HHLL, LLHH-HHHL, LLHH-LLLL produced the same general patterns of bias as seen overall and in the combinations where sensitivity was HHLL, although with different magnitudes (e.g. average overestimation of ${Se}_{1}$ and ${Se}_{2}$ by 0.223 and 0.222 in the combination LLHH-HHLL, compared with 0.343 and 0.342 in the combination LLHH-LLLL). In contrast, estimates were unbiased on average for the combination LLHH-LLHH, or close to unbiased for the combination LLHH-HHHH. In the combinations LLHH-LLHL and LLHH-LHLH, the magnitudes of biases were large for some data sets but very close to zero for others, indicating that the CInd model provided different parameter estimates yielding similar fits (identifiability issues). This is consistent with the much lower convergence rates observed for these two combinations, suggesting that different chains may have converged to different results.

Coverages of 95% CrIs of parameter estimates, summarised across different prevalence, correlation and sample size settings, are also available in Additional file 4. Across all converged models, the 95% CrIs for ${Se}_{1}$ and ${Se}_{2}$ contained the true parameter values only around 18% of the time. Coverages were the lowest for the sensitivities of the correlated test pair (${T}_{1}$ and ${T}_{2}$), as we would expect. Coverages for all parameters were higher in the scenarios with lower prevalence, correlation, and sample sizes. When ${n}_{obs}=5000$, 95% CrIs for ${Se}_{1}$ and ${Se}_{2}$ contained the true values for only 5.0% and 5.3% of the converged data sets, respectively.

### Overall fit measures

Table 3 displays the percentage of converged models for which each tool indicated lack of overall fit. Results are shown overall and summarised across scenarios with each prevalence, correlation or sample size. Across scenarios, ${\chi }^{2}$ and ${G}^{2}$ goodness of fit statistics consistently showed lower power than residual correlation plots in identifying lack of fit resulting from the violation of the CInd assumption. Table 3 also suggests slightly higher power of the overall ${\chi }^{2}$ test compared with overall ${G}^{2}$. As we would expect, all tools were less likely to detect lack of overall fit when sample size or prevalence was low and when the magnitude of the conditional correlation was lower.

**Table 3:** Performance of tools in detecting overall lack of fit in different scenarios

Scenarios	Residual Correlation Plot	Overall ${{\varvec{G}}}^{2}$	Overall ${{\varvec{\chi}}}^{2}$
**All**	74.3 (74.1,74.4)	64.5 (64.4,64.6)	67.5 (67.4,67.6)
${\varvec{\pi}}=0.5$	82.2 (82.1,82.4)	74.0 (73.8,74.2)	77.1 (76.9,77.3)
${\varvec{\pi}}=0.2$	66.1 (65.9,66.3)	54.7 (54.5,54.9)	57.6 (57.4,57.8)
${\varvec{\omega}}=0.9$	81.0 (80.8,81.1)	71.6 (71.4,71.8)	76.3 (76.1,76.5)
${\varvec{\omega}}=0.5$	67.2 (67.0,67.4)	57.0 (56.8,57.2)	57.1 (56.9,57.3)
${{\varvec{n}}}_{{\varvec{o}}{\varvec{b}}{\varvec{s}}}=5000$	86.9 (86.8,87.1)	80.5 (80.3,80.7)	80.7 (80.5,80.9)
${{\varvec{n}}}_{{\varvec{o}}{\varvec{b}}{\varvec{s}}}=500$	58.5 (58.2,58.7)	42.1 (41.8,42.3)	50.7 (50.4,50.9)

Percentage of converged models for which the residual correlation plot, overall ${G}^{2}$ and overall ${\chi }^{2}$ statistics detected a lack of overall fit in different scenarios. Percentages are shown with 95% MC CIs

Table 4 shows results of the same format but for the 14 example parameter combinations. When the sensitivities were HHHH, the performance of all three tools varied immensely depending on the specificities. For example, residual correlation plots, ${G}^{2}$ and ${\chi }^{2}$ tests identified a lack of fit of the CInd model for 64.9%, 25.1% and 24.2% of converged datasets, respectively, for the sensitivity–specificity combination HHHH-HHLL, but 99.9%, 100%, 100% of the time for the combination HHHH-HHHH. In contrast, all tools detected a lack of overall fit for all data sets when sensitivity was LLHH, regardless of the specificity values.

**Table 4:** Performance of tools in detecting overall lack of fit for different sensitivity–specificity combinations

Sensitivity	Specificity	Tool		
		**Residual Correlation Plot**	**Overall** ${{\varvec{G}}}^{2}$	**Overall** ${{\varvec{\chi}}}^{2}$
HHHH	HHLL	64.9 (61.9,67.9)	25.1 (22.4,27.9)	24.2 (21.6,27.0)
	HHHL	88.4 (86.3,90.3)	87.0 (84.8,89.0)	86.5 (84.2,88.6)
	HHHH	99.9 (99.4,100.0)	100.0 (99.6,100.0)	100.0 (99.6,100.0)
	LLLL	86.5 (84.2,88.6)	70.3 (67.3,73.1)	70.9 (67.9,73.7)
	LLHL	98.7 (97.8,99.3)	99.8 (99.3,100.0)	99.8 (99.3,100.0)
	LLHH	98.8 (97.9,99.4)	100.0 (99.6,100.0)	100.0 (99.6,100.0)
	LHLH	96.3 (94.9,97.4)	97.9 (96.8,98.7)	97.8 (96.7,98.6)
LLHH	HHLL	100.0 (99.6,100.0)	100.0 (99.6,100.0)	100.0 (99.6,100.0)
	HHHL	100.0 (99.6,100.0)	100.0 (99.6,100.0)	100.0 (99.6,100.0)
	HHHH	100.0 (99.6,100.0)	100.0 (99.6,100.0)	100.0 (99.6,100.0)
	LLLL	100.0 (99.6,100.0)	100.0 (99.6,100.0)	100.0 (99.6,100.0)
	LLHL	100.0 (99.4,100.0)	100.0 (99.4,100.0)	100.0 (99.4,100.0)
	LLHH	100.0 (99.6,100.0)	100.0 (99.6,100.0)	100.0 (99.6,100.0)
	LHLH	100.0 (99.5,100.0)	100.0 (99.5,100.0)	100.0 (99.5,100.0)

Percentage of converged models for which the residual correlation plot, overall ${G}^{2}$ and overall ${\chi }^{2}$ statistics detected a lack of overall fit in different combinations of sensitivities and specificities from where $\pi =0.5$, $\omega =0.5$, ${n}_{obs}=2000$. Percentages are shown with 95% MC CIs

### Pairwise fit measures

All pairwise tools exhibited markedly poor performance in detecting the correct conditionally correlated pair of tests. Counterintuitively, on average across all scenarios, all pairwise tools indicated lack of fit much more frequently for the pair ${T}_{3}\times {T}_{4}$ than for ${T}_{1}\times {T}_{2}$. On average across data sets, residual correlation plots only suggested ${T}_{1}\times {T}_{2}$ misfit 12.1% of the time, but T3 x T4 misfit 64.9% of the time. The percentage of data sets where pairwise ${G}^{2}$ and ${\chi }^{2}$ tests indicated a misfit for ${T}_{1}\times {T}_{2}$ were both 10.3%, but those for ${T}_{3}\times {T}_{4}$ were 49.7% and 49.5%. This tendency to highlight lack of fit for ${T}_{3}\times {T}_{4}$ was exacerbated with larger sample size and higher prevalence (Table 5). All three tools were more likely to detect ${T}_{1}\times {T}_{2}$ misfit with larger sample size and prevalence, but also more likely to suggest misfit across all other pairs. Counterintuitively, a higher true conditional correlation between ${T}_{1}{\times T}_{2}$ also resulted in reduced likelihood of detecting ${T}_{1}{\times T}_{2}$ misfit and an increased likelihood of incorrectly detecting ${T}_{3}\times {T}_{4}$ misfit, for all three methods (Table 5).

**Table 5:** Performance of tools in detecting pairwise lack of fit in different scenarios

	**Tool: Residual Correlation Plot**					
**Scenarios**	${{\varvec{T}}}_{1}\times {{\varvec{T}}}_{2}$	${{\varvec{T}}}_{1}\times {{\varvec{T}}}_{3}$	${{\varvec{T}}}_{1}\times {{\varvec{T}}}_{4}$	${{\varvec{T}}}_{2}\times {{\varvec{T}}}_{3}$	${{\varvec{T}}}_{2}\times {{\varvec{T}}}_{4}$	${{\varvec{T}}}_{3}\times {{\varvec{T}}}_{4}$
**All**	12.1 (12.1,12.2)	20.8 (20.7,20.9)	21.2 (21.1,21.3)	13.8 (13.7,13.9)	13.8 (13.7,13.9)	64.9 (64.7,65.0)
${\varvec{\pi}}=0.5$	12.5 (12.3,12.6)	24.8 (24.6,24.9)	24.5 (24.3,24.6)	17.4 (17.3,17.6)	15.9 (15.8,16.1)	75.7 (75.6,75.9)
${\varvec{\pi}}=0.2$	11.8 (11.7,12.0)	16.7 (16.6,16.9)	17.9 (17.7,18.0)	10.1 (9.9,10.2)	11.6 (11.5,11.8)	53.7 (53.5,53.9)
${\varvec{\omega}}=0.9$	9.2 (9.1,9.3)	30.0 (29.9,30.2)	28.0 (27.8,28.1)	21.6 (21.4,21.8)	18.1 (18.0,18.3)	74.3 (74.1,74.5)
${\varvec{\omega}}=0.5$	15.3 (15.1,15.4)	11.0 (10.9,11.2)	14.1 (13.9,14.2)	5.6 (5.5,5.7)	9.2 (9.1,9.4)	54.8 (54.6,55.1)
${{\varvec{n}}}_{{\varvec{o}}{\varvec{b}}{\varvec{s}}}=5000$	17.3 (17.1,17.5)	30.8 (30.5,31.0)	31.3 (31.0,31.5)	22.1 (21.9,22.3)	21.3 (21.1,21.5)	80.0 (79.8,80.2)
${{\varvec{n}}}_{{\varvec{o}}{\varvec{b}}{\varvec{s}}}=500$	7.2 (7.0,7.3)	10.3 (10.1,10.5)	11.1 (10.9,11.3)	5.3 (5.2,5.5)	6.9 (6.8,7.0)	46.6 (46.3,46.8)
	**Pairwise** ${{\varvec{G}}}^{2}$					
**Scenarios**	${{\varvec{T}}}_{1}\times {{\varvec{T}}}_{2}$	${{\varvec{T}}}_{1}\times {{\varvec{T}}}_{3}$	${{\varvec{T}}}_{1}\times {{\varvec{T}}}_{4}$	${{\varvec{T}}}_{2}\times {{\varvec{T}}}_{3}$	${{\varvec{T}}}_{2}\times {{\varvec{T}}}_{4}$	${{\varvec{T}}}_{3}\times {{\varvec{T}}}_{4}$
**All**	10.3 (10.2,10.4)	14.0 (13.9,14.1)	13.6 (13.5,13.7)	10.2 (10.1,10.3)	7.8 (7.7,7.9)	49.7 (49.5,49.8)
${\varvec{\pi}}=0.5$	11.4 (11.3,11.5)	18.8 (18.6,19.0)	17.9 (17.7,18.0)	13.8 (13.7,14.0)	10.7 (10.5,10.8)	61.4 (61.2,61.6)
${\varvec{\pi}}=0.2$	9.2 (9.0,9.3)	9.1 (9.0,9.2)	9.3 (9.2,9.4)	6.4 (6.3,6.5)	4.9 (4.8,5.0)	37.6 (37.4,37.8)
${\varvec{\omega}}=0.9$	7.3 (7.2,7.4)	22.3 (22.2,22.5)	19.8 (19.6,19.9)	17.1 (16.9,17.2)	11.6 (11.5,11.7)	57.4 (57.2,57.6)
${\varvec{\omega}}=0.5$	13.5 (13.3,13.6)	5.2 (5.1,5.3)	7.1 (7.0,7.2)	2.9 (2.9,3.0)	3.8 (3.7,3.9)	41.5 (41.3,41.7)
${{\varvec{n}}}_{{\varvec{o}}{\varvec{b}}{\varvec{s}}}=5000$	14.9 (14.7,15.1)	23.5 (23.3,23.7)	23.2 (23.0,23.4)	17.5 (17.3,17.7)	14.3 (14.1,14.5)	67.7 (67.4,97.9)
${{\varvec{n}}}_{{\varvec{o}}{\varvec{b}}{\varvec{s}}}=500$	5.6 (5.5,5.7)	4.2 (4.1,4.3)	4.0 (3.9,4.1)	2.7 (2.6,2.8)	1.7 (1.7,1.8)	28.5 (28.3,28.7)
	**Pairwise** ${{\varvec{\chi}}}^{2}$					
**Scenarios**	${{\varvec{T}}}_{1}\times {{\varvec{T}}}_{2}$	${{\varvec{T}}}_{1}\times {{\varvec{T}}}_{3}$	${{\varvec{T}}}_{1}\times {{\varvec{T}}}_{4}$	${{\varvec{T}}}_{2}\times {{\varvec{T}}}_{3}$	${{\varvec{T}}}_{2}\times {{\varvec{T}}}_{4}$	${{\varvec{T}}}_{3}\times {{\varvec{T}}}_{4}$
**All**	10.3 (10.2,10.3)	14.1 (14.0,14.2)	13.8 (13.7,13.9)	10.3 (10.2,10.4)	7.9 (7.8,8.0)	49.5 (49.4,49.6)
${\varvec{\pi}}=0.5$	11.4 (11.3,11.5)	19.0 (18.8,19.1)	18.0 (17.9,18.2)	14.0 (13.9,14.2)	10.8 (10.7,10.9)	61.2 (61.0,61.4)
${\varvec{\pi}}=0.2$	9.1 (9.0,9.3)	9.1 (9.0,9.3)	9.4 (9.2,9.5)	6.5 (6.4,6.6)	4.9 (4.8,5.0)	37.5 (37.3,37.7)
${\varvec{\omega}}=0.9$	7.3 (7.2,7.4)	22.5 (22.4,22.7)	20.0 (19.8,20.1)	17.2 (17.1,17.4)	11.7 (11.6,11.8)	57.3 (57.1,57.5)
${\varvec{\omega}}=0.5$	13.4 (13.3,13.6)	5.3 (5.2,5.3)	7.2 (7.1,7.3)	3.0 (2.9,3.1)	3.8 (3.8,3.9)	41.3 (41.1,41.5)
${{\varvec{n}}}_{{\varvec{o}}{\varvec{b}}{\varvec{s}}}=5000$	14.9 (14.7,15.0)	23.7 (23.5,23.9)	23.3 (23.1,23.5)	17.6 (17.5,17.8)	14.4 (14.2,14.6)	67.6 (67.4,67.9)
${{\varvec{n}}}_{{\varvec{o}}{\varvec{b}}{\varvec{s}}}=500$	5.6 (5.5,5.7)	4.3 (4.2,4.4)	4.1 (4.0,4.2)	2.8 (2.7,2.9)	1.8 (1.7,1.9)	28.1 (27.9,28.4)

Percentage of converged models for which the residual correlation plot, pairwise ${G}^{2}$ and pairwise ${\chi }^{2}$ statistics detected a lack of pairwise fit for each pair in different scenarios. Percentages are shown with 95% MC CIs

Since all three pairwise tools showed similar performance patterns, for our 14 example parameter combinations we show the performance of residual correlation plots only in Table 6. We observe substantial differences in tool performance across these 14 settings. Most combinations produced results consistent with the general tendency to fail to detect dependence for the correct test pair, ${T}_{1}\times {T}_{2},$ but to suggest lack of model fit for ${T}_{3}\times {T}_{4}$. However, interestingly, for the sensitivity–specificity combinations LLHH-HHHH and LLHH-LLHH, we see 100% probability of correctly indicating ${T}_{1}\times {T}_{2}$ dependence, and a low probability of suggesting dependence between other test pairs. We note that these were the combinations with the lowest average biases (Fig. 2) and are also combinations where tests 3 and 4 are more accurate than tests 1 and 2. The sensitivity–specificity combination LLHH-LLHL also led to 100% detection of ${T}_{1}\times {T}_{2}$ misfit, although with a non-negligible probability of also indicating lack of fit for other pairs (up to 34.0% for ${T}_{2}\times {T}_{4}$). The combination with sensitivity–specificity LLHH-LLLL was also somewhat unusual, in that misfit was indicated more than 75% of the time for ${T}_{1}\times {T}_{3}$, ${T}_{1}\times {T}_{4}, {T}_{2}\times {T}_{3}$ and ${T}_{2}\times {T}_{4}$, in addition to ${T}_{3}\times {T}_{4}.$ This may be associated with bias in additional parameters in this scenario (Fig. 2). The combination where sensitivity–specificity = LLHH-LHLH also showed unusual behaviour, which may relate to the bimodality in bias (Fig. 2).

**Table 6:** Performance of residual correlation plot in detecting pairwise lack of fit for different sensitivity–specificity combinations

		**Test Pairs**					
**Sensitivity**	**Specificity**	${{\varvec{T}}}_{1}\times {{\varvec{T}}}_{2}$	${{\varvec{T}}}_{1}\times {{\varvec{T}}}_{3}$	${{\varvec{T}}}_{1}\times {{\varvec{T}}}_{4}$	${{\varvec{T}}}_{2}\times {{\varvec{T}}}_{3}$	${{\varvec{T}}}_{2}\times {{\varvec{T}}}_{4}$	${{\varvec{T}}}_{3}\times {{\varvec{T}}}_{4}$
HHHH	HHLL	0.0 (0.0,0.4)	0.0 (0.0,0.4)	0.0 (0.0,0.4)	0.0 (0.0,0.4)	0.0 (0.0,0.4)	64.9 (61.9,67.9)
	HHHL	0.0 (0.0,0.4)	0.0 (0.0,0.4)	0.0 (0.0,0.4)	0.0 (0.0,0.4)	0.0 (0.0,0.4)	88.4 (86.3,90.3)
	HHHH	0.1 (0.0,0.6)	0.0 (0.0,0.4)	0.0 (0.0,0.4)	0.0 (0.0,0.4)	0.0 (0.0,0.4)	99.9 (99.4,100.0)
	LLLL	1.5 (0.9,2.5)	2.5 (1.6,3.7)	3.7 (2.6,5.0)	4.2 (3.0,5.6)	3.1 (2.1,4.4)	85.8 (83.4,87.9)
	LLHL	15.8 (13.6,18.2)	0.1 (0.0,0.6)	12.9 (10.9,15.1)	0.2 (0.0,0.7)	12.4 (10.4,14.6)	98.7 (97.8,99.3)
	LLHH	80.1 (77.5,82.5)	3.3 (2.3,4.6)	2.7 (1.8,3.9)	2.8 (1.9,4.0)	2.4 (1.5,3.6)	92.6 (90.8,94.1)
	LHLH	0.0 (0.0,0.4)	16.5 (14.3,18.9)	3.2 (2.2,4.5)	0.0 (0.0,0.4)	0.0 (0.0,0.4)	96.0 (94.6,97.1)
LLHH	HHLL	0.0 (0.0,0.4)	0.0 (0.0,0.4)	0.2 (0.0,0.7)	0.0 (0.0,0.4)	0.1 (0.0,0.6)	100.0 (99.6,100.0)
	HHHL	11.0 (9.1,13.1)	0.0 (0.0,0.4)	12.2 (10.2,14.4)	0.0 (0.0,0.4)	10.2 (8.4,12.3)	100.0 (99.6,100.0)
	HHHH	100.0 (99.6,100.0)	0.9 (0.4,1.7)	0.4 (0.1,1.0)	1.0 (0.5,1.8)	1.0 (0.5,1.8)	5.2 (3.9,6.8)
	LLLL	30.9 (28.0,34.0)	78.6 (75.8,81.2)	79.0 (76.3,81.6)	79.5 (76.7,82.0)	78.6 (75.8,81.2)	100.0 (99.6,100.0)
	LLHL	100.0 (99.4,100.0)	13.1 (10.6,16.1)	32.1 (28.4,35.9)	13.6 (11.0,16.6)	34.0 (30.3,37.9)	13.8 (11.2,16.8)
	LLHH	100.0 (99.6,100.0)	0.0 (0.0,0.4)	0.0 (0.0,0.4)	0.0 (0.0,0.4)	0.0 (0.0,0.4)	0.0 (0.0,0.4)
	LHLH	45.1 (41.6,48.6)	95.1 (93.4,96.5)	97.2 (95.9,98.3)	3.3 (2.1,4.7)	0.0 (0.0,0.5)	94.7 (93.0,96.2)

Percentage of converged models for which the residual correlation plots detected a lack of pairwise fit for each pair in different combinations of sensitivities and specificities from where $\pi =0.5$, $\omega =0.5$, ${n}_{obs}=2000$. Percentages are shown with 95% MC CIs

For a better understanding of varying performances of the pairwise tools depending on different combinations of sensitivity and specificity, we selected two parameter combinations from the set of 14 for further exploration:

- Sensitivity–specificity = HHHH-HHHH, a combination that is typical in that tools indicated a lack of fit only for ${T}_{3}\times {T}_{4}$. As was seen in Fig. 1, prevalence, and specificities of ${T}_{3}$ and ${T}_{4}$ were slightly underestimated, while sensitivities of the correlated tests (${Se}_{1}$ and ${Se}_{2}$) were slightly overestimated on average in this scenario (Fig. 2).
- Sensitivity–specificity = LLHH-LLHH, where tools indicated a lack of fit for the correct pair and not for any other pairs. In this scenario, all parameter estimates were unbiased (Fig. 2).

Table 7 shows, for each test pair, the observed pairwise agreement probabilities and the fitted agreement probabilities according to the CInd model for these two combinations. We also show the expected pairwise agreement probabilities under a CInd model with all parameter values set to match our data generating model, except for $\omega$ = 0 (labelled ‘True CInd’).

**Table 7:** Observed, fitted and true CInd (CInd-assumed data-generating observed) pairwise agreement probabilities for different sensitivity–specificity combinations

**Combination**	**Pairwise Agreement Probability**	${{\varvec{T}}}_{1}\times {{\varvec{T}}}_{2}$	${{\varvec{T}}}_{1}\times {{\varvec{T}}}_{3}$	${{\varvec{T}}}_{1}\times {{\varvec{T}}}_{4}$	${{\varvec{T}}}_{2}\times {{\varvec{T}}}_{3}$	${{\varvec{T}}}_{2}\times {{\varvec{T}}}_{4}$	${{\varvec{T}}}_{3}\times {{\varvec{T}}}_{4}$
Sensitivity: HHHH Specificity: HHHH	**Observed**	0.87	0.82	0.82	0.82	0.82	0.82
	**Fitted**	0.86	0.82	0.82	0.82	0.82	0.79
	**True CInd**	0.82	0.82	0.82	0.82	0.82	0.82
Sensitivity: LLHH Specificity: LLHH	**Observed**	0.64	0.58	0.58	0.58	0.58	0.82
	**Fitted**	0.52	0.58	0.58	0.58	0.58	0.82
	**True CInd**	0.52	0.58	0.58	0.58	0.58	0.82

Two sensitivity–specificity combinations providing different agreement structures. Values of other parameters set at: $\pi =0.5$, $\omega =0.5$, ${n}_{obs}=2000$

As the data-generating model incorporates a conditional dependence only for the pair ${T}_{1}\times {T}_{2}$, the true CInd probabilities and observed probabilities are identical for the other diagnostic test pairs (Table 7). In the combination HHHH-HHHH, introducing a conditional dependence for the pair ${T}_{1}\times {T}_{2}$ increases the observed pairwise agreement for this pair from 0.82 to 0.87, making it the pair with the highest observed pairwise agreement. The fitted CInd model succeeds in explaining this high pairwise agreement (fitted value = 0.86), but at the cost of failing to fully accommodate the observed ${T}_{3}\times {T}_{4}$ agreement. Essentially, in order to accommodate the observed ${T}_{1}\times {T}_{2}$ agreement the ‘wrong’ CInd model is fitted, producing biased parameter estimates. Pairwise tools subsequently indicate ${T}_{3}\times {T}_{4}$ misfit. In contrast, the parameter values in the combination where sensitivity–specificity = LLHH-LLHH lead to the largest pairwise agreement being between ${T}_{3}$ and ${T}_{4},$ despite the conditional dependence being between ${T}_{1}$ and ${T}_{2}.$ In this relatively extreme scenario in which ${T}_{3}$ and ${T}_{4}$ have substantially higher sensitivity and specificity than either ${T}_{1}$ or ${T}_{2},$ the fitted CInd model succeeds in accommodating the ${T}_{3}\times {T}_{4}$ agreement, at the cost of failing to accommodate the observed ${T}_{1}\times {T}_{2}$ agreement. The ‘correct’ CInd model is fitted, i.e. a CInd model with unbiased parameter estimates, and the pairwise tools then behave as originally intended – suggesting a lack of fit for ${T}_{1}\times {T}_{2}$.

## Discussion

We explored the performance of pairwise and overall goodness of fit tools widely used in the diagnostic test LCM literature with a simulation study. Evaluating the fit of these models is important, since fitting the wrong model can produce highly biased estimates of test accuracy. Our results, consistent with previous literature [5, 6], demonstrate that failure to account for conditional dependence tends to result in biased model parameters, and the magnitude of bias increases with higher conditional correlations. Consistent with the study of Keddie et al. [7], we found that failure to account for the conditional dependence between ${T}_{1}$ and ${T}_{2}$ led not only to bias in estimates of ${Se}_{1}$ and ${Se}_{2}$ and $\pi$, but also to bias in estimates of ${Sp}_{3}$ and ${Sp}_{4}$ (i.e., specificities of the uncorrelated tests).

The global statistics (overall ${G}^{2}$ and ${\chi }^{2}$) and residual correlation plots, on average across all converged models, showed relatively low power in identifying a lack of overall fit. Unsurprisingly, the power of these tools was at its lowest when prevalence, conditional correlation or sample size were low.

The results of this paper show differences from and similarities with two previous studies [33, 37]. The power of the residual correlation plot, overall ${G}^{2}$ and ${\chi }^{2}$ statistics to detect departures from the CInd assumption appeared somewhat better in the current study. However, this may be explained by the different selection of the model parameters, correlation structure and—in particular—the use of larger sample sizes (largest sample sizes used in studies: 5000 in current study, 2000 in Van Smeden et al. [33], and 1000 in Subtil et al. [37]). In line with the findings from Subtil et al. [37], all pairwise tools indicated lack of fit for a conditionally independent pair (${T}_{3}\times {T}_{4}$) instead of the correlated pair (${T}_{1}\times {T}_{2}$) from the fitted CInd model most of the time. We extended the Subtil et al. [37] work by including a much broader range of parameter settings, and observing some relationship between pairwise tool performance and bias.

We found that this interesting and unintuitive result from Subtil et al. [37] held across the vast majority of parameter combinations considered. Our results appear to suggest that the CInd model tends to work to fit the highest marginal pairwise agreement between tests. Typically (but not always – as these marginal agreements are also impacted upon by test sensitivities and specificities, and prevalence) this can be expected to be the conditionally dependent test pair. Accommodating this high observed ${T}_{1}\times {T}_{2}$ pairwise agreement within a CInd model leads to biased parameter estimates and a lack of fit elsewhere in the model. The somewhat unintuitive consequence of this is that the areas in which the fitted CInd model has the worst fit do not appear to be informative in highlighting where conditional dependence parameters need to be added. We emphasise that our results are consistent with these pairwise model fit tools performing well at detecting where the fitted CInd model fits poorly – but not at indicating where additional dependence terms need to be added.

There was, however, some variation in tool performance across parameter settings. In a limited number of particular scenarios, we observed that the residual correlation plots (and other pairwise tools) behaved as originally intended by Qu et al. [10]: showing a lack of fit for the conditionally dependent pair only. An example was the sensitivity–specificity combination LLHH-LLHH, where tests 3 and 4 have substantially higher sensitivity and specificity than tests 1 and 2, heavily impacting upon the marginal correlation structure. We note that in this particular situation the fitted CInd model also provided unbiased estimates. As the ‘correct’ CInd model (i.e., the CInd model with the correct prevalence, sensitivities and specificities but without the covariance term) has been fitted, the lack of model fit is where we might anticipate it to be (${T}_{1}\times {T}_{2})$, such that the pairwise fit tools behave as intended. However, we note that determining the correct correlation structure may not be imperative in such a scenario in which CInd model parameter estimates were already unbiased.

Based on these findings, we conclude that residual correlation plots and pairwise ${G}^{2}$ and ${\chi }^{2}$ statistics cannot be relied upon to indicate which pairs of tests are conditionally dependent. Alternative model selection strategies are therefore needed for the situation in which there is no a priori biological knowledge available. One approach might be to adopt a forward selection strategy, based on values of an information criterion, to select among different models in which correlation terms are added sequentially.

This study was one of the most extensive simulation studies in diagnostic latent class models, encompassing 504 distinct parameter settings, with 1000 simulated data sets generated per parameter setting. However, we acknowledge that we only considered a simple correlation structure with a single conditional dependence term within the diseased group. We additionally only considered situations in which the two positively correlated tests had simultaneously high or low sensitivities, which may not always be the case in practice. Furthermore, certain parameter combinations, such as those with uniformly low sensitivities and specificities, may not accurately reflect real-world scenarios. The tools evaluated in this study (i.e., residual correlation plots, ${\chi }^{2}$ and ${G}^{2}$ tests) were originally developed within the frequentist framework, whereas we evaluated Bayesian analogues of these. Qu et al. [10] proposed using 95% bootstrap CIs around residual correlation estimates to assess evidence of unexplained pairwise correlations in the fitted model, therefore we used 95% CrIs in our evaluation. Future work might explore tool performance in the presence of more complex correlation structures, and/or conditional dependence within both the diseased and disease-free group.

## Conclusions

Residual correlation plots, and pairwise ${G}^{2}$ and ${\chi }^{2}$ statistics cannot be relied upon to determine which tests are conditionally dependent. Also, residual correlation plots and overall versions of those statistics have relatively low power in detecting lack of overall fit of a CInd model. However, assuming conditional independence incorrectly tends to produce highly biased parameter estimates. We therefore advise against selecting the conditional dependence structure solely based on these tools. Further work is needed to develop statistical selection strategies that can be used alongside clinical information on how the tests work to identify dependencies between tests, so that reliable test accuracy estimates can be obtained from latent class models.

## Supplementary Information

Additional file 1: Required number of simulations for different binomial proportions, to achieve a Monte Carlo confidence interval with length *L* = *0.05*.

Additional file 2: R and JAGS scripts to simulate data and fit conditional independence models.

Additional file 3: Full results for all converged conditional independence models.

Additional file 4: Median absolute biases and 95% CrI coverages summarised across all converged data sets (of the 504,000), across all converged data sets with each value of prevalence ($\pi$) or correlation ($\omega$), and across all converged data sets with each sample size.

## Data Availability

The R script simulating data from an example scenario (set values of prevalence, sample size and covariance) for the 42 combinations of sensitivity and specificity combinations, and the JAGS script to fit the conditional independence model to those data sets, are available in Additional file 2.

## Abbreviations

LCM: Latent Class Model
CInd: Conditional Independence
CDep: Conditional Dependence
CI: Confidence Interval
MCMC: Markov Chain Monte Carlo
df: Degrees of freedom
ADEMP: Aims, Data-generating mechanisms, Estimands, Methods, Performance measures
MC: Monte Carlo
CrI: Credible Intervals
JAGS: Just Another Gibbs Sampler
