# Supplementary material for: Detecting departures from the conditional independence assumption in diagnostic latent class models: a simulation study
Source: BMC Med Res Methodol. 2024 Dec 5;24:299. doi: 10.1186/s12874-024-02432-x (PMC11619692; doi:10.1186/s12874-024-02432-x)

Required number of simulations for different binomial proportions (p), to achieve a Monte Carlo confidence interval with length *L=0.05*

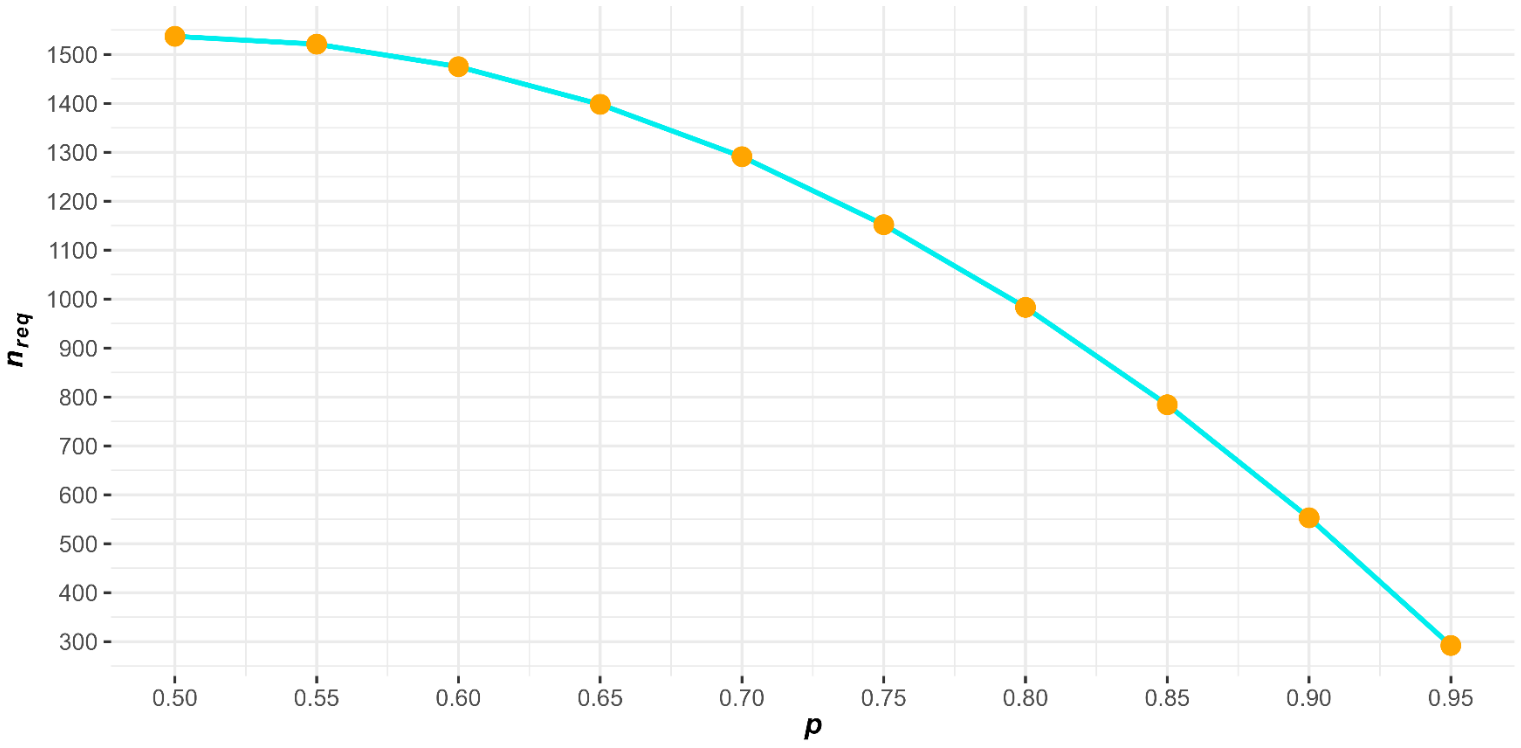

Supplement: Supplementary file 1 — Additional file 1: Required number of simulations for different binomial proportions, to achieve a Monte Carlo confidence interval with length L = 0.05. [file 12874_2024_2432_MOESM1_ESM.pdf]
