# Supplementary material for: Detecting departures from the conditional independence assumption in diagnostic latent class models: a simulation study
Source: BMC Med Res Methodol. 2024 Dec 5;24:299. doi: 10.1186/s12874-024-02432-x (PMC11619692; doi:10.1186/s12874-024-02432-x)
Supplement: Supplementary file 2 — Additional file 2: R and JAGS scripts to simulate data and fit conditional independence models. [file 12874_2024_2432_MOESM2_ESM.pdf]

R and JAGS Scripts to Simulate Data and Fit Conditional Independence Models

i. R Script

The following R code (with comments) simulates 1,000 data sets with a parameter setting: $n_{obs} = 5000$, $\pi = 0.5$, and $\omega = 0.9$ for all 42 sensitivity-specificity combinations.

```
## This code simulates 1000 data sets for 4 diagnostic tests' results from
a conditional dependence model with a pre-specified sample size, prevalence
and covariance (between Test 1 and Test 2 within the diseased group)
#for all 42 sensitivity-specificity combinations and fits a conditional
independence model to each dataset

##Call required packages to run this code parallelly on the High
Performance Computing System of the University of Bristol
require(Rmpi)
require(doMPI)
require(foreach)
require(R2jags)
require(parallel)

##Create a folder to save the outputs (NB, no need to specify a directory
as it is specified on the Linux system)
folder <- "Scenario1_ssize=5000, prev=0.5, cor_coef=0.5"
if(!file.exists(folder)) dir.create(folder)

##Set a seed for the reproducibility of the results
set.seed(351)

##MCMC settings to fit the conditional independence models: number of burn-
in iterations, number of iterations, thinning, number of chains
n.burnin <- 5000
n.iter <- 10000
n.thin <- 1
n.chains <- 3

##Set the number of simulated data sets, sample size, prevalence,
coefficient of the maximum available covariance
nsim <- 1000
ssize <- 5000
prev=0.5
cor_coef=0.9

##Define all sensitivity and specificity combinations with both magnitudes
and letters and combine them
sec1<-c(0.9,0.9,0.6,0.6)
sec2<-c(0.9,0.9,0.9,0.6)
sec3<-c(0.9,0.9,0.9,0.9)
sec4<-c(0.6,0.6,0.6,0.6)
sec5<-c(0.6,0.6,0.9,0.6)
sec6<-c(0.6,0.6,0.9,0.9)
secn1<-"H,H,L,L"
secn2<-"H,H,H,L"
```

```
secn3<-"H,H,H,H"
secn4<-"L,L,L,L"
secn5<-"L,L,H,L"
secn6<-"L,L,H,H"

spc1<-c(0.9,0.9,0.6,0.6)
spc2<-c(0.9,0.9,0.9,0.6)
spc3<-c(0.9,0.9,0.9,0.9)
spc4<-c(0.6,0.6,0.6,0.6)
spc5<-c(0.6,0.6,0.9,0.6)
spc6<-c(0.6,0.6,0.9,0.9)
spc7<-c(0.6,0.9,0.6,0.9)
spcn1<-"H,H,L,L"
spcn2<-"H,H,H,L"
spcn3<-"H,H,H,H"
spcn4<-"L,L,L,L"
spcn5<-"L,L,H,L"
spcn6<-"L,L,H,H"
spcn7<-"L,H,L,H"

sec<-cbind(sec1,sec2,sec3,sec4,sec5,sec6)
secn<-cbind(secn1,secn2,secn3,secn4,secn5,secn6)

spc<-cbind(spc1,spc2,spc3,spc4,spc5,spc6,spc7)
spcn<-cbind(spcn1,spcn2,spcn3,spcn4,spcn5,spcn6,spcn7)

##Define a function to simulate data from the conditional dependence model
and to fit the conditional independence model
#using the prevalence, sensitivities, specificities, and coefficient of the
maximum covariance as inputs
scenario1<-function(prev,se,sp,cor_coef){
##Define the covariance between Test1 and Test2 as the coefficient
multiplied by the maximum possible covariance
  covse12<-cor_coef*min(se[1]*(1-se[2]),se[2]*(1-se[1]))

  p<-matrix(NA,nrow=16,ncol = 2)
##Define test results patterns
  Y<-expand.grid(Y1=c(0,1),Y2=c(0,1),Y3=c(0,1),Y4=c(0,1))

##Calculate true probabilities given the disease status for the patterns
defined above
  for(i in 1:16){
    p[i,1]<-prev*((se[1]^(Y[i,1])*(1-se[1])^(1-Y[i,1])*se[2]^(Y[i,2])*(1-
se[2])^(1-Y[i,2])+(-1)^(Y[i,1]-Y[i,2])*covse12)*se[3]^(Y[i,3])*(1-
se[3])^(1-Y[i,3])*se[4]^(Y[i,4])*(1-se[4])^(1-Y[i,4]))
    p[i,2]<-(1-prev)*(sp[1]^(1-Y[i,1])*(1-sp[1])^(Y[i,1])*sp[2]^(1-
Y[i,2])*(1-sp[2])^(Y[i,2])*sp[3]^(1-Y[i,3])*(1-sp[3])^(Y[i,3])*sp[4]^(1-
Y[i,4])*(1-sp[4])^(Y[i,4]))
  }

##Calculate true marginal probabilities for each test result pattern
  prob<-p[,1]+p[,2]


  simulated<-matrix(NA,nrow=16,ncol=nsim)

##Simulate nsim datasets from a multinomial distribution with the true
marginal probabilities
    for(j in 1:nsim){
      simulated[,j]<-rmultinom(1,ssize,prob)
    }
```

```
  Yarray<-array(NA,dim=c(16,5,nsim))

##Put all simulated frequencies and observed patterns in an array
  for(i in 1:16){
    for(j in 1:4){
      for(k in 1:nsim){
        Yarray[i,j,k]<-Y[i,j]
        Yarray[i,5,k]<-simulated[i,k]
      }
    }
  }

  Corlowertrianglevectors<-matrix(NA,nrow = 6,ncol = nsim)

##Calculate lower triangle of the observed Pearson correlation matrix for all simulated datasets
  for(k in 1:nsim){
    Corlowertrianglevectors[1,k]<-
(ssize*sum((Yarray[,1,k]+1)*(Yarray[,2,k]+1)*Yarray[,5,k])-
sum((Yarray[,1,k]+1)*Yarray[,5,k])*sum((Yarray[,2,k]+1)*Yarray[,5,k]))/(
      sqrt(ssize*sum((Yarray[,1,k]+1)^2*Yarray[,5,k])-
sum((Yarray[,1,k]+1)*Yarray[,5,k])^2)*sqrt(ssize*sum((Yarray[,2,k]+1)^2*Yarray[,5,k])-sum((Yarray[,2,k]+1)*Yarray[,5,k])^2))
    Corlowertrianglevectors[2,k]<-
(ssize*sum((Yarray[,1,k]+1)*(Yarray[,3,k]+1)*Yarray[,5,k])-
sum((Yarray[,1,k]+1)*Yarray[,5,k])*sum((Yarray[,3,k]+1)*Yarray[,5,k]))/(
      sqrt(ssize*sum((Yarray[,1,k]+1)^2*Yarray[,5,k])-
sum((Yarray[,1,k]+1)*Yarray[,5,k])^2)*sqrt(ssize*sum((Yarray[,3,k]+1)^2*Yarray[,5,k])-sum((Yarray[,3,k]+1)*Yarray[,5,k])^2))
    Corlowertrianglevectors[3,k]<-
(ssize*sum((Yarray[,1,k]+1)*(Yarray[,4,k]+1)*Yarray[,5,k])-
sum((Yarray[,1,k]+1)*Yarray[,5,k])*sum((Yarray[,4,k]+1)*Yarray[,5,k]))/(
      sqrt(ssize*sum((Yarray[,1,k]+1)^2*Yarray[,5,k])-
sum((Yarray[,1,k]+1)*Yarray[,5,k])^2)*sqrt(ssize*sum((Yarray[,4,k]+1)^2*Yarray[,5,k])-sum((Yarray[,4,k]+1)*Yarray[,5,k])^2))
    Corlowertrianglevectors[4,k]<-
(ssize*sum((Yarray[,2,k]+1)*(Yarray[,3,k]+1)*Yarray[,5,k])-
sum((Yarray[,2,k]+1)*Yarray[,5,k])*sum((Yarray[,3,k]+1)*Yarray[,5,k]))/(
      sqrt(ssize*sum((Yarray[,2,k]+1)^2*Yarray[,5,k])-
sum((Yarray[,2,k]+1)*Yarray[,5,k])^2)*sqrt(ssize*sum((Yarray[,3,k]+1)^2*Yarray[,5,k])-sum((Yarray[,3,k]+1)*Yarray[,5,k])^2))
    Corlowertrianglevectors[5,k]<-
(ssize*sum((Yarray[,2,k]+1)*(Yarray[,4,k]+1)*Yarray[,5,k])-
sum((Yarray[,2,k]+1)*Yarray[,5,k])*sum((Yarray[,4,k]+1)*Yarray[,5,k]))/(
      sqrt(ssize*sum((Yarray[,2,k]+1)^2*Yarray[,5,k])-
sum((Yarray[,2,k]+1)*Yarray[,5,k])^2)*sqrt(ssize*sum((Yarray[,4,k]+1)^2*Yarray[,5,k])-sum((Yarray[,4,k]+1)*Yarray[,5,k])^2))
    Corlowertrianglevectors[6,k]<-
(ssize*sum((Yarray[,3,k]+1)*(Yarray[,4,k]+1)*Yarray[,5,k])-
sum((Yarray[,3,k]+1)*Yarray[,5,k])*sum((Yarray[,4,k]+1)*Yarray[,5,k]))/(
      sqrt(ssize*sum((Yarray[,3,k]+1)^2*Yarray[,5,k])-
sum((Yarray[,3,k]+1)*Yarray[,5,k])^2)*sqrt(ssize*sum((Yarray[,4,k]+1)^2*Yarray[,5,k])-sum((Yarray[,4,k]+1)*Yarray[,5,k])^2))
  }


  obs.prob.ag<-matrix(NA,nrow=6,ncol = nsim)

##Calculate observed pairwise agreement probabilities for all test pairs
```

```
  for(k in 1:nsim){
    obs.prob.ag[1,k]<-
(sum(Yarray[Yarray[,1,k]==1&Yarray[,2,k]==1,5,k])+sum(Yarray[Yarray[,1,k]==
0&Yarray[,2,k]==0,5,k]))/ssize
    obs.prob.ag[2,k]<-
(sum(Yarray[Yarray[,1,k]==1&Yarray[,3,k]==1,5,k])+sum(Yarray[Yarray[,1,k]==
0&Yarray[,3,k]==0,5,k]))/ssize
    obs.prob.ag[3,k]<-
(sum(Yarray[Yarray[,1,k]==1&Yarray[,4,k]==1,5,k])+sum(Yarray[Yarray[,1,k]==
0&Yarray[,4,k]==0,5,k]))/ssize
    obs.prob.ag[4,k]<-
(sum(Yarray[Yarray[,2,k]==1&Yarray[,3,k]==1,5,k])+sum(Yarray[Yarray[,2,k]==
0&Yarray[,3,k]==0,5,k]))/ssize
    obs.prob.ag[5,k]<-
(sum(Yarray[Yarray[,2,k]==1&Yarray[,4,k]==1,5,k])+sum(Yarray[Yarray[,2,k]==
0&Yarray[,4,k]==0,5,k]))/ssize
    obs.prob.ag[6,k]<-
(sum(Yarray[Yarray[,3,k]==1&Yarray[,4,k]==1,5,k])+sum(Yarray[Yarray[,3,k]==
0&Yarray[,4,k]==0,5,k]))/ssize
  }

##Specify the parameters to be monitored
  mymonitoredparamslist <- c("prev","se","sp","res.cor","fitted.prob.ag")

  prevmedian<-rep(NA,nsim)
  bias_prev<-rep(NA,nsim)
  prevlower<-rep(NA,nsim)
  prevupper<-rep(NA,nsim)
  semedian<-matrix(NA,nrow=4,ncol=nsim)
  bias_se<-matrix(NA,nrow = 4,ncol=nsim)
  selower<-matrix(NA,nrow=4,ncol=nsim)
  seupper<-matrix(NA,nrow=4,ncol=nsim)
  spmedian<-matrix(NA,nrow=4,ncol=nsim)
  bias_sp<-matrix(NA,nrow = 4, ncol=nsim)
  splower<-matrix(NA,nrow=4,ncol=nsim)
  spupper<-matrix(NA,nrow=4,ncol=nsim)
  rescor<-matrix(NA,nrow=6,ncol=nsim)
  rescorlower<-matrix(NA,nrow=6,ncol = nsim)
  rescorupper<-matrix(NA,nrow = 6,ncol=nsim)
  fitted.prob.ag<-matrix(NA,nrow = 6,ncol=nsim)

##Fit a conditional independence model to each simulated data set
  for(j in 1:nsim){
    mydatalist =
list(Tally_RRRRR=simulated[,j],Y=Y,obs.cor=Corlowertrianglevectors[,j],
ssize=ssize)
    fit <- jags(data=mydatalist,
                parameters.to.save= mymonitoredparamslist,
                model.file= "simulated_hpc.txt",
                n.chains=n.chains,
                n.iter= n.iter,
                n.burnin =  n.burnin,
                n.thin = n.thin,
                progress.bar = "text")

if(any(fit$BUGSoutput$summary[,8]>1.1)|any(fit$BUGSoutput$summary[,9]<400))
{  #Convergence Rule: if Rhat >1.1 or neff<400 for any parameter, save all
outputs as missing values
      prevmedian[j]<-prevlower[j]<-prevupper[j]<-bias_prev[j]<-NA
      semedian[,j]<-selower[,j]<-seupper[,j]<-spmedian[,j]<-splower[,j]<-
spupper[,j]<-bias_se[,j]<-bias_sp[,j]<-NA
```

```
      rescor[,j]<-rescorlower[,j]<-rescorupper[,j]<-obs.prob.ag[,j]<-
fitted.prob.ag[,j]<-NA
    }
    else{ #if the model converged, then
      prevmedian[j]<-fit$BUGSoutput$median$prev #Save posterior median
prevalence
      bias_prev[j]<-prevmedian[j]-prev #Save bias for prevalence as
posterior median prevalence minus true value
      prevlower[j]<-fit$BUGSoutput$summary[7,3] #Save lower limits of 95%
CrIs for prevalence (for coverage)
      prevupper[j]<-fit$BUGSoutput$summary[7,7] #Save upper limits of 95%
CrIs for prevalence (for coverage)
      semedian[,j]<-fit$BUGSoutput$median$se #Save posterior median
sensitivities
      bias_se[,j]<-semedian[,j]-se #Save bias for sensitivities as
posterior median sensitivity minus true values
      selower[,j]<-fit$BUGSoutput$summary[15:18,3] #Save lower limits of
95% CrIs for sensitivities (for coverage)
      seupper[,j]<-fit$BUGSoutput$summary[15:18,7] #Save upper limits of
95% CrIs for sensitivities (for coverage)
      spmedian[,j]<-fit$BUGSoutput$median$sp #Save posterior median
specificities
      bias_sp[,j]<-spmedian[,j]-sp #Save bias for specificities as
posterior median sensitivity minus true values
      splower[,j]<-fit$BUGSoutput$summary[19:22,3] #Save lower limits of
95% CrIs for specificities (for coverage)
      spupper[,j]<-fit$BUGSoutput$summary[19:22,7] #Save upper limits of
95% CrIs for specificities (for coverage)
      rescor[,j]<-fit$BUGSoutput$median$res.cor #Save posterior median
residual correlations
      rescorlower[,j]<-fit$BUGSoutput$summary[9:14,3] #Save lower limits of
95% CrIs for residual correlations to check if this is lower than zero
      rescorupper[,j]<-fit$BUGSoutput$summary[9:14,7] #Save upper limits of
95% CrIs for residual correlations to check if this is greater than zero
      fitted.prob.ag[,j]<-fit$BUGSoutput$median$fitted.prob.ag #Save fitted
pairwise agreement probabilities
    }
  } ##Save the vectors and matrices defined above in the output
  assign("prevmedian",prevmedian,envir = .GlobalEnv)
  assign("bias_prev",bias_prev, envir = .GlobalEnv)
  assign("prevlower",prevlower,envir = .GlobalEnv)
  assign("prevupper",prevupper, envir = .GlobalEnv)
  assign("semedian",semedian,envir = .GlobalEnv)
  assign("bias_se",bias_se,envir = .GlobalEnv)
  assign("selower",selower,envir = .GlobalEnv)
  assign("seupper",seupper, envir = .GlobalEnv)
  assign("spmedian",spmedian,envir = .GlobalEnv)
  assign("bias_sp",bias_sp,envir = .GlobalEnv)
  assign("splower",splower,envir = .GlobalEnv)
  assign("spupper",spupper,envir = .GlobalEnv)
  assign("rescor",rescor,envir = .GlobalEnv)
  assign("rescorlower",rescorlower,envir = .GlobalEnv)
  assign("rescorupper",rescorupper,envir = .GlobalEnv)
  assign("se",se,envir = .GlobalEnv)
  assign("sp",sp,envir = .GlobalEnv)
  assign("fitted.prob.ag",fitted.prob.ag,envir = .GlobalEnv)
}

##Create an array including numerical values of all possible sensitivity-
specificity combinations
sec_spc_array<-array(NA,dim=c(4,2,42))
```

```
sec_spc_array[,1,1:7]<-sec[,1]
sec_spc_array[,1,8:14]<-sec[,2]
sec_spc_array[,1,15:21]<-sec[,3]
sec_spc_array[,1,22:28]<-sec[,4]
sec_spc_array[,1,29:35]<-sec[,5]
sec_spc_array[,1,36:42]<-sec[,6]
sec_spc_array[,2,1:7]<-spc[,1:7]
sec_spc_array[,2,8:14]<-spc[,1:7]
sec_spc_array[,2,15:21]<-spc[,1:7]
sec_spc_array[,2,22:28]<-spc[,1:7]
sec_spc_array[,2,29:35]<-spc[,1:7]
sec_spc_array[,2,36:42]<-spc[,1:7]



##Create a vector showing all possible sensitivity specificity combinations
with letters
secn_spcn_vector<-rep(NA,42)

secn_spcn_vector[1:7]<-paste(secn[1],"    ",spcn[1:7])
secn_spcn_vector[8:14]<-paste(secn[2],"    ",spcn[1:7])
secn_spcn_vector[15:21]<-paste(secn[3],"    ",spcn[1:7])
secn_spcn_vector[22:28]<-paste(secn[4],"    ",spcn[1:7])
secn_spcn_vector[29:35]<-paste(secn[5],"    ",spcn[1:7])
secn_spcn_vector[36:42]<-paste(secn[6],"    ",spcn[1:7])


##Run each step of the for loop on different nodes on the high performance
computer.
cl <- startMPIcluster()
registerDoMPI(cl)
foreach (i= 1:42, .packages = "R2jags") %dopar% {
  scenario1(prev,sec_spc_array[,1,i],sec_spc_array[,2,i],cor_coef) #Run the
function defined above for all possible sensitivity-specificty combinations
where ssize=5000, prev=0.5, cor_coef=0.9
  save.image(file=file.path(folder, paste(secn_spcn_vector[i],".RData")))
#Save the output data with the letters of sensitivity specificity
combinations, for example, HHHH,HHLL.RData
}

closeCluster(cl)
mpi.quit()
```

ii. JAGS Script

The following JAGS code specifies a conditional independence latent class model and calculates residual correlations and pairwise agreement statistics.

```
model{
	##Define the likelihood (Tally_RRRRR: observed frequencies)
	Tally_RRRRR[1:16] ~ dmulti(prob_RRRRR[1:16], ssize)

	##Define priors
	prev ~ dbeta(1,1)

for(i in 1:4){
	se[i] ~ dbeta(1,1)T(1-sp[i], )
	sp[i] ~ dbeta(1,1)
	}

	##Calculate probabilities (prob_RRRRR:unconditional probabilities, 
p1:probabilities within the diseased state, p0:probabilities within the 
disease-free state)
for(i in 1:16){
		prob_RRRRR[i] <- p0[i]+p1[i]
		p1[i] <- prev*(se[1]^(Y[i,1])*(1-se[1])^(1-
Y[i,1])*se[2]^(Y[i,2])*(1-se[2])^(1-Y[i,2])*se[3]^(Y[i,3])*(1-se[3])^(1-
Y[i,3])*se[4]^(Y[i,4])*(1-se[4])^(1-Y[i,4]))
		p0[i] <- (1-prev)*(sp[1]^(1-Y[i,1])*(1-
sp[1])^(Y[i,1])*sp[2]^(1-Y[i,2])*(1-sp[2])^(Y[i,2]) *sp[3]^(1-Y[i,3])*(1-
sp[3])^(Y[i,3])*sp[4]^(1-Y[i,4])*(1-sp[4])^(Y[i,4]))
	}
	##Calculate expected frequencies
for(i in 1:16) {
		Exp.freq[i]<-prob_RRRRR[i]*ssize
	}

	##Calculate fitted pairwise correlations
fitted.cor[1]<-(ssize*sum((Y[,1]+1)*(Y[,2]+1)*Exp.freq)-
sum((Y[,1]+1)*Exp.freq)*sum((Y[,2]+1)*Exp.freq))/(sqrt(ssize*sum((Y[,1]+1)^
2*Exp.freq)-
sum((Y[,1]+1)*Exp.freq)^2)*sqrt(ssize*sum((Y[,2]+1)^2*Exp.freq)-
sum((Y[,2]+1)*Exp.freq)^2))
fitted.cor[2]<-(ssize*sum((Y[,1]+1)*(Y[,3]+1)*Exp.freq)-
sum((Y[,1]+1)*Exp.freq)*sum((Y[,3]+1)*Exp.freq))/(sqrt(ssize*sum((Y[,1]+1)^
2*Exp.freq)-
sum((Y[,1]+1)*Exp.freq)^2)*sqrt(ssize*sum((Y[,3]+1)^2*Exp.freq)-
sum((Y[,3]+1)*Exp.freq)^2))
fitted.cor[3]<-(ssize*sum((Y[,1]+1)*(Y[,4]+1)*Exp.freq)-
sum((Y[,1]+1)*Exp.freq)*sum((Y[,4]+1)*Exp.freq))/(sqrt(ssize*sum((Y[,1]+1)^
2*Exp.freq)-
sum((Y[,1]+1)*Exp.freq)^2)*sqrt(ssize*sum((Y[,4]+1)^2*Exp.freq)-
sum((Y[,4]+1)*Exp.freq)^2))
fitted.cor[4]<-(ssize*sum((Y[,2]+1)*(Y[,3]+1)*Exp.freq)-
sum((Y[,2]+1)*Exp.freq)*sum((Y[,3]+1)*Exp.freq))/(sqrt(ssize*sum((Y[,2]+1)^
2*Exp.freq)-
sum((Y[,2]+1)*Exp.freq)^2)*sqrt(ssize*sum((Y[,3]+1)^2*Exp.freq)-
sum((Y[,3]+1)*Exp.freq)^2))
fitted.cor[5]<-(ssize*sum((Y[,2]+1)*(Y[,4]+1)*Exp.freq)-
sum((Y[,2]+1)*Exp.freq)*sum((Y[,4]+1)*Exp.freq))/(sqrt(ssize*sum((Y[,2]+1)^
2*Exp.freq)-
```

```
sum((Y[,2]+1)*Exp.freq)^2)*sqrt(ssize*sum((Y[,4]+1)^2*Exp.freq)-
sum((Y[,4]+1)*Exp.freq)^2))
fitted.cor[6]<-(ssize*sum((Y[,3]+1)*(Y[,4]+1)*Exp.freq)-
sum((Y[,3]+1)*Exp.freq)*sum((Y[,4]+1)*Exp.freq))/(sqrt(ssize*sum((Y[,3]+1)^
2*Exp.freq)-
sum((Y[,3]+1)*Exp.freq)^2)*sqrt(ssize*sum((Y[,4]+1)^2*Exp.freq)-
sum((Y[,4]+1)*Exp.freq)^2))

      ##Create fitted pairwise tables to calculate pairwise agreement
probabilities

pair.fitted[1,1]<-sum(Y[,1]*Y[,2]*Exp.freq)
pair.fitted[2,1]<-sum(Y[,1]*(1-Y[,2])*Exp.freq)
pair.fitted[3,1]<-sum((1-Y[,1])*Y[,2]*Exp.freq)
pair.fitted[4,1]<-sum((1-Y[,1])*(1-Y[,2])*Exp.freq)

pair.fitted[1,2]<-sum(Y[,1]*Y[,3]*Exp.freq)
pair.fitted[2,2]<-sum(Y[,1]*(1-Y[,3])*Exp.freq)
pair.fitted[3,2]<-sum((1-Y[,1])*Y[,3]*Exp.freq)
pair.fitted[4,2]<-sum((1-Y[,1])*(1-Y[,3])*Exp.freq)

pair.fitted[1,3]<-sum(Y[,1]*Y[,4]*Exp.freq)
pair.fitted[2,3]<-sum(Y[,1]*(1-Y[,4])*Exp.freq)
pair.fitted[3,3]<-sum((1-Y[,1])*Y[,4]*Exp.freq)
pair.fitted[4,3]<-sum((1-Y[,1])*(1-Y[,4])*Exp.freq)

pair.fitted[1,4]<-sum(Y[,2]*Y[,3]*Exp.freq)
pair.fitted[2,4]<-sum(Y[,2]*(1-Y[,3])*Exp.freq)
pair.fitted[3,4]<-sum((1-Y[,2])*Y[,3]*Exp.freq)
pair.fitted[4,4]<-sum((1-Y[,2])*(1-Y[,3])*Exp.freq)

pair.fitted[1,5]<-sum(Y[,2]*Y[,4]*Exp.freq)
pair.fitted[2,5]<-sum(Y[,2]*(1-Y[,4])*Exp.freq)
pair.fitted[3,5]<-sum((1-Y[,2])*Y[,4]*Exp.freq)
pair.fitted[4,5]<-sum((1-Y[,2])*(1-Y[,4])*Exp.freq)

pair.fitted[1,6]<-sum(Y[,3]*Y[,4]*Exp.freq)
pair.fitted[2,6]<-sum(Y[,3]*(1-Y[,4])*Exp.freq)
pair.fitted[3,6]<-sum((1-Y[,3])*Y[,4]*Exp.freq)
pair.fitted[4,6]<-sum((1-Y[,3])*(1-Y[,4])*Exp.freq)

      ##Calculate pairwise residual correlations and agreement
probabilities
for(i in 1:6){
      res.cor[i]<-obs.cor[i]-fitted.cor[i]
      fitted.prob.ag[i]<-
(pair.fitted[1,i]+pair.fitted[4,i])/sum(Tally_RRRRR)
}
}
```
