# Supplementary material for: Detecting departures from the conditional independence assumption in diagnostic latent class models: a simulation study
Source: BMC Med Res Methodol. 2024 Dec 5;24:299. doi: 10.1186/s12874-024-02432-x (PMC11619692; doi:10.1186/s12874-024-02432-x)
Supplement: Supplementary file 3 — Additional file 3: Full results for all converged conditional independence models. [file 12874_2024_2432_MOESM3_ESM.pdf]

Full Results for All Converged Conditional Independence Models

In this document, we present full results for all 473,368 data sets with converged conditional independence models (out of 504,000). We classified the results into 12 combinations of prevalence (π), conditional Pearson correlation (ω) and sample sizes (n_{obs}). For each of these 12 combinations (2 prevalences, 2 covariances, 3 sample sizes), there are 42 combinations of sensitivities ($Se_i, i = 1,2,3,4$) and specificities ($Sp_i, i = 1,2,3,4$). For each of the 12 parameter settings, results are presented in 5 tables showing (for all 42 sensitivity/specificity combinations):

- **Table 1:** Number of converged data sets
- **Table 2: Performance of tools in detecting overall lack of fit.** Percentages of the time 95% credible intervals (CrIs) for residual correlations did not included '0' for **any** pairs, and percentages of the time that the overall χ^2 or G^2 statistic indicated a lack of overall fit
- **Table 3: Performance of tools in detecting pairwise lack of fit.** Percentages of the time 95% CrIs for residual correlations did not included '0' for **each** pair, and percentages of the time the pairwise χ^2 or G^2 statistic for **each** pair indicated a lack of pairwise fit
- **Table 4: Bias.** Mean absolute biases (x10^{-2}) of posterior medians of each parameter obtained from the conditional independence model
- **Table 5: Coverage**. Coverages of the 95% CrIs for each model parameter

To quantify uncertainties around simulations, 95% Monte Carlo (MC) confidence intervals (CIs) for percentages (Pearson-Clopper CI), biases and coverages were also provided in tables.

Denominators for all percentages are the numbers of data sets with converged results (maximum of 1000).

Throughout the document, the letters '**H**' and '**L**' refer to 0.9 (High) and 0.6 (Low) sensitivity and specificity settings in data generation, respectively.

i. Setting 1: $\pi = 0.5, \omega = 0.9, n_{obs} = 5000$

Table i.1 Number of converged data sets for different sensitivity-specificity combinations

				**Number of converged data sets**
**Specificity**				**Sensitivity: H H L L**
**H**	**H**	**L**	**L**	959
**H**	**H**	**H**	**L**	1000
**H**	**H**	**H**	**H**	1000
**L**	**L**	**L**	**L**	913
**L**	**L**	**H**	**L**	1000
**L**	**L**	**H**	**H**	1000
**L**	**H**	**L**	**H**	1000
**Specificity**				**Sensitivity: H H H L**
**H**	**H**	**L**	**L**	1000
**H**	**H**	**H**	**L**	1000
**H**	**H**	**H**	**H**	1000
**L**	**L**	**L**	**L**	1000
**L**	**L**	**H**	**L**	999
**L**	**L**	**H**	**H**	999
**L**	**H**	**L**	**H**	1000
**Specificity**				**Sensitivity: H H H H**
**H**	**H**	**L**	**L**	1000
**H**	**H**	**H**	**L**	1000
**H**	**H**	**H**	**H**	1000
**L**	**L**	**L**	**L**	999
**L**	**L**	**H**	**L**	997
**L**	**L**	**H**	**H**	1000
**L**	**H**	**L**	**H**	1000
**Specificity**				**Sensitivity: L L L L**
**H**	**H**	**L**	**L**	908
**H**	**H**	**H**	**L**	999
**H**	**H**	**H**	**H**	1000
**L**	**L**	**L**	**L**	933
**L**	**L**	**H**	**L**	998
**L**	**L**	**H**	**H**	998
**L**	**H**	**L**	**H**	999
**Specificity**				**Sensitivity: L L H L**
**H**	**H**	**L**	**L**	998

**H**	**H**	**H**	**L**	999
**H**	**H**	**H**	**H**	1000
**L**	**L**	**L**	**L**	999
**L**	**L**	**H**	**L**	1000
**L**	**L**	**H**	**H**	998
**L**	**H**	**L**	**H**	999
**Specificity**				**Sensitivity: L L H H**
**H**	**H**	**L**	**L**	999
**H**	**H**	**H**	**L**	1000
**H**	**H**	**H**	**H**	998
**L**	**L**	**L**	**L**	999
**L**	**L**	**H**	**L**	999
**L**	**L**	**H**	**H**	770
**L**	**H**	**L**	**H**	999

Table i.2 Percentages of the time 95% credible intervals (CrIs) for residual correlations did not included '0' for any pairs, and percentages of the time that the overall χ^2 or G^2 statistic indicated a lack of overall fit

**Sensitivity: H H L L**						
**Specificity**				**Res. Cor.**	**Overall G^2**	**Overall χ^2**
**H**	**H**	**L**	**L**	65.0 (61.8,68.0)	17.4 (15.1,20.0)	17.5 (15.2,20.1)
**H**	**H**	**H**	**L**	68.4 (65.4,71.3)	62.1 (59.0,65.1)	60.9 (57.8,63.9)
**H**	**H**	**H**	**H**	99.7 (99.1,99.9)	100.0 (99.6,100.0)	100.0 (99.6,100.0)
**L**	**L**	**L**	**L**	75.2 (72.3,78.0)	70.3 (67.2,73.3)	70.9 (67.8,73.8)
**L**	**L**	**H**	**L**	98.5 (97.5,99.2)	100.0 (99.6,100.0)	100.0 (99.6,100.0)
**L**	**L**	**H**	**H**	100.0 (99.6,100.0)	100.0 (99.6,100.0)	100.0 (99.6,100.0)
**L**	**H**	**L**	**H**	98.9 (98.0,99.4)	98.8 (97.9,99.4)	98.9 (98.0,99.4)
**Sensitivity: H H H L**						
**Specificity**				**Res. Cor.**	**Overall G^2**	**Overall χ^2**
**H**	**H**	**L**	**L**	65.4 (62.4,68.3)	36.0 (33.0,39.1)	35.8 (32.8,38.9)
**H**	**H**	**H**	**L**	85.2 (85.9,90.0)	91.0 (89.1,92.7)	90.6 (88.6,92.3)
**H**	**H**	**H**	**H**	100.0 (99.6,100.0)	100.0 (99.6,100.0)	100.0 (99.6,100.0)
**L**	**L**	**L**	**L**	97.6 (96.4,98.5)	99.5 (98.8,99.8)	99.5 (98.8,99.8)
**L**	**L**	**H**	**L**	100.0 (99.4,100.0)	100.0 (99.6,100.0)	100.0 (99.6,100.0)
**L**	**L**	**H**	**H**	100.0 (99.6,100.0)	100.0 (99.6,100.0)	100.0 (99.6,100.0)
**L**	**H**	**L**	**H**	100.0 (99.6,100.0)	100.0 (99.6,100.0)	100.0 (99.6,100.0)
**Sensitivity: H H H H**						
**Specificity**				**Res. Cor.**	**Overall G^2**	**Overall χ^2**
**H**	**H**	**L**	**L**	100.0 (99.6,100.0)	96.8 (95.5,97.8)	96.9 (95.6,97.9)
**H**	**H**	**H**	**L**	100.0 (99.6,100.0)	100.0 (99.6,100.0)	100.0 (99.6,100.0)

**H**	**H**	**H**	**H**	100.0 (99.6,100.0)	100.0 (99.6,100.0)	100.0 (99.6,100.0)
**L**	**L**	**L**	**L**	100.0 (99.6,100.0)	100.0 (99.6,100.0)	100.0 (99.6,100.0)
**L**	**L**	**H**	**L**	100.0 (99.6,100.0)	100.0 (99.6,100.0)	100.0 (99.6,100.0)
**L**	**L**	**H**	**H**	100.0 (99.6,100.0)	100.0 (99.6,100.0)	100.0 (99.6,100.0)
**L**	**H**	**L**	**H**	100.0 (99.6,100.0)	100.0 (99.6,100.0)	100.0 (99.6,100.0)
**Sensitivity: L L L L**						
**Specificity**				**Res. Cor.**	**Overall G^2**	**Overall χ^2**
**H**	**H**	**L**	**L**	92.3 (90.4,93.9)	61.1 (57.9,64.3)	61.5 (58.2,64.6)
**H**	**H**	**H**	**L**	100.0 (99.6,100.0)	100.0 (99.6,100.0)	100.0 (99.6,100.0)
**H**	**H**	**H**	**H**	100.0 (99.6,100.0)	100.0 (99.6,100.0)	100.0 (99.6,100.0)
**L**	**L**	**L**	**L**	100.0 (99.6,100.0)	100.0 (99.6,100.0)	100.0 (99.6,100.0)
**L**	**L**	**H**	**L**	100.0 (99.6,100.0)	100.0 (99.6,100.0)	100.0 (99.6,100.0)
**L**	**L**	**H**	**H**	100.0 (99.6,100.0)	100.0 (99.6,100.0)	100.0 (99.6,100.0)
**L**	**H**	**L**	**H**	100.0 (99.6,100.0)	100.0 (99.6,100.0)	100.0 (99.6,100.0)
**Sensitivity: L L H L**						
**Specificity**				**Res. Cor.**	**Overall G^2**	**Overall χ^2**
**H**	**H**	**L**	**L**	100.0 (99.6,100.0)	99.8 (99.3,100.0)	99.9 (99.4,100.0)
**H**	**H**	**H**	**L**	100.0 (99.6,100.0)	100.0 (99.6,100.0)	100.0 (99.6,100.0)
**H**	**H**	**H**	**H**	100.0 (99.6,100.0)	100.0 (99.6,100.0)	100.0 (99.6,100.0)
**L**	**L**	**L**	**L**	100.0 (99.6,100.0)	100.0 (99.6,100.0)	100.0 (99.6,100.0)
**L**	**L**	**H**	**L**	100.0 (99.6,100.0)	100.0 (99.6,100.0)	100.0 (99.6,100.0)
**L**	**L**	**H**	**H**	100.0 (99.6,100.0)	100.0 (99.6,100.0)	100.0 (99.6,100.0)
**L**	**H**	**L**	**H**	100.0 (99.4,100.0)	100.0 (99.6,100.0)	100.0 (99.6,100.0)
**Sensitivity: L L H H**						
**Specificity**				**Res. Cor.**	**Overall G^2**	**Overall χ^2**
**H**	**H**	**L**	**L**	100.0 (99.6,100.0)	100.0 (99.6,100.0)	100.0 (99.6,100.0)
**H**	**H**	**H**	**L**	100.0 (99.6,100.0)	100.0 (99.6,100.0)	100.0 (99.6,100.0)
**H**	**H**	**H**	**H**	100.0 (99.6,100.0)	100.0 (99.6,100.0)	100.0 (99.6,100.0)
**L**	**L**	**L**	**L**	100.0 (99.6,100.0)	100.0 (99.6,100.0)	100.0 (99.6,100.0)
**L**	**L**	**H**	**L**	100.0 (99.6,100.0)	100.0 (99.6,100.0)	100.0 (99.6,100.0)
**L**	**L**	**H**	**H**	100.0 (99.5,100.0)	100.0 (99.5,100.0)	100.0 (99.5,100.0)
**L**	**H**	**L**	**H**	100.0 (99.6,100.0)	100.0 (99.6,100.0)	100.0 (99.6,100.0)

Table i.3 Percentages of the time 95% CrIs for residual correlations did not included '0' for each pair, and percentages of the time the pairwise χ^2 or G^2 statistic for each pair indicated a lack of pairwise fit

**Sensitivity: H H L L**										
**Specificity**				**Tool**	**T_1&T_2**	**T_1&T_3**	**T_1&T_4**	**T_2&T_3**	**T_2&T_4**	**T_3&T_4**
**H**	**H**	**L**	**L**	**Res. Cor.**	0.0 (0.0,0.4)	0.0 (0.0,0.4)	0.0 (0.0,0.4)	0.0 (0.0,0.4)	0.0 (0.0,0.4)	65.0 (61.8,68.0)
				**Pairwise G^2**	0.0 (0.0,0.4)	0.0 (0.0,0.4)	0.0 (0.0,0.4)	0.0 (0.0,0.4)	0.0 (0.0,0.4)	6.7 (5.2,8.4)

				Pairwise χ²	0.0 (0.0,0.4)	0.0 (0.0,0.4)	0.0 (0.0,0.4)	0.0 (0.0,0.4)	0.0 (0.0,0.4)	6.9 (5.4,8.7)
**H**	**H**	**H**	**L**	**Res. Cor.**	0.0 (0.0,0.4)	0.0 (0.0,0.4)	0.0 (0.0,0.4)	0.0 (0.0,0.4)	0.0 (0.0,0.4)	68.4 (65.4,71.3)
				**Pairwise G²**	0.0 (0.0,0.4)	0.0 (0.0,0.4)	0.0 (0.0,0.4)	0.0 (0.0,0.4)	0.0 (0.0,0.4)	24.8 (22.2,27.6)
				**Pairwise χ²**	0.0 (0.0,0.4)	0.0 (0.0,0.4)	0.0 (0.0,0.4)	0.0 (0.0,0.4)	0.0 (0.0,0.4)	24.8 (22.2,27.6)
**H**	**H**	**H**	**H**	**Res. Cor.**	0.0 (0.0,0.4)	0.0 (0.0,0.4)	0.0 (0.0,0.4)	0.0 (0.0,0.4)	0.0 (0.0,0.4)	99.7 (99.1,99.9)
				**Pairwise G²**	0.0 (0.0,0.4)	0.0 (0.0,0.4)	0.0 (0.0,0.4)	0.0 (0.0,0.4)	0.0 (0.0,0.4)	97.8 (96.7,98.6)
				**Pairwise χ²**	0.0 (0.0,0.4)	0.0 (0.0,0.4)	0.0 (0.0,0.4)	0.0 (0.0,0.4)	0.0 (0.0,0.4)	97.8 (96.7,98.6)
**L**	**L**	**L**	**L**	**Res. Cor.**	0.0 (0.0,0.4)	6.0 (4.6,7.8)	5.3 (3.9,6.9)	5.3 (3.9,6.9)	7.0 (5.4,8.9)	68.8 (65.7,71.8)
				**Pairwise G²**	0.0 (0.0,0.4)	1.9 (1.1,3.0)	1.5 (0.8,2.6)	0.9 (0.4,1.7)	1.1 (0.5,2.0)	11.1 (9.1,13.3)
				**Pairwise χ²**	0.0 (0.0,0.4)	1.9 (1.1,3.0)	1.5 (0.8,2.6)	1.0 (0.5,1.9)	1.1 (0.5,2.0)	11.1 (9.1,13.3)
**L**	**L**	**H**	**L**	**Res. Cor.**	0.0 (0.0,0.4)	63.9 (60.8,66.9)	12.3 (10.3,14.5)	60.1 (57.0,63.2)	9.6 (7.8,11.6)	86.7 (84.4,88.7)
				**Pairwise G²**	0.0 (0.0,0.4)	45.3 (42.2,48.4)	2.5 (1.6,3.7)	42.3 (39.2,45.4)	2.4 (1.5,3.6)	53.1 (50.0,56.2)
				**Pairwise χ²**	0.0 (0.0,0.4)	55.4 (52.3,58.5)	54.6 (51.5,57.7)	55.1 (52.0,58.2)	58.9 (55.8,62.0)	100.0 (99.6,100.0)
**L**	**L**	**H**	**H**	**Res. Cor.**	0.0 (0.0,0.4)	72.9 (70.0,75.6)	73.4 (70.5,76.1)	73.5 (70.6,76.2)	74.8 (72.0,77.5)	100.0 (99.6,100.0)
				**Pairwise G²**	9.3 (7.6,11.3)	89.2 (87.1,91.1)	89.9 (87.9,91.7)	88.1 (85.9,90.0)	90.2 (88.2,92.0)	100.0 (99.6,100.0)
				**Pairwise χ²**	8.7 (7.0,10.6)	89.7 (87.6,91.5)	90.5 (88.5,92.2)	88.5 (86.4,90.4)	90.5 (88.5,92.2)	100.0 (99.6,100.0)
**L**	**H**	**L**	**H**	**Res. Cor.**	0.0 (0.0,0.4)	24.9 (22.2,27.7)	93.9 (92.2,95.3)	0.0 (0.0,0.4)	0.0 (0.0,0.4)	72.1 (69.2,74.9)
				**Pairwise G²**	0.0 (0.0,0.4)	6.5 (5.1,8.2)	85.6 (83.3,87.7)	0.0 (0.0,0.4)	0.0 (0.0,0.4)	31.1 (28.2,34.1)
				**Pairwise χ²**	0.0 (0.0,0.4)	6.5 (5.1,8.2)	86.5 (84.2,88.6)	0.0 (0.0,0.4)	0.0 (0.0,0.4)	31.1 (28.2,34.1)
**Sensitivity: H H H L**										
**Specificity**				**Tool**	**T_1&T_2**	**T_1&T_3**	**T_1&T_4**	**T_2&T_3**	**T_2&T_4**	**T_3&T_4**
**H**	**H**	**L**	**L**	**Res. Cor.**	0.0 (0.0,0.4)	0.2 (0.0,0.7)	0.0 (0.0,0.4)	0.0 (0.0,0.4)	0.0 (0.0,0.4)	65.4 (62.4,68.3)
				**Pairwise G²**	0.0 (0.0,0.4)	0.1 (0.0,0.6)	0.0 (0.0,0.4)	0.0 (0.0,0.4)	0.0 (0.0,0.4)	22.7 (20.1,25.4)
				**Pairwise χ²**	0.0 (0.0,0.4)	0.1 (0.0,0.6)	0.0 (0.0,0.4)	0.0 (0.0,0.4)	0.0 (0.0,0.4)	22.7 (20.1,25.4)
**H**	**H**	**H**	**L**	**Res. Cor.**	0.0 (0.0,0.4)	0.0 (0.0,0.4)	0.0 (0.0,0.4)	0.0 (0.0,0.4)	0.0 (0.0,0.4)	85.2 (82.8,87.3)
				**Pairwise G²**	0.0 (0.0,0.4)	0.0 (0.0,0.4)	0.0 (0.0,0.4)	0.0 (0.0,0.4)	0.0 (0.0,0.4)	61.2 (58.1,64.2)
				**Pairwise χ²**	0.0 (0.0,0.4)	0.0 (0.0,0.4)	0.0 (0.0,0.4)	0.0 (0.0,0.4)	0.0 (0.0,0.4)	61.2 (58.1,64.2)
**H**	**H**	**H**	**H**	**Res. Cor.**	0.0 (0.0,0.4)	0.0 (0.0,0.4)	0.0 (0.0,0.4)	0.0 (0.0,0.4)	0.0 (0.0,0.4)	100.0 (99.6,100.0)
				**Pairwise G²**	0.0 (0.0,0.4)	0.0 (0.0,0.4)	0.0 (0.0,0.4)	0.0 (0.0,0.4)	0.0 (0.0,0.4)	100.0 (99.6,100.0)
				**Pairwise χ²**	0.0 (0.0,0.4)	0.0 (0.0,0.4)	0.0 (0.0,0.4)	0.0 (0.0,0.4)	0.0 (0.0,0.4)	100.0 (99.6,100.0)
**L**	**L**	**L**	**L**	**Res. Cor.**	0.0 (0.0,0.4)	58.5 (55.4,61.6)	12.6 (10.6,14.8)	61.7 (58.6,64.7)	14.3 (12.2,16.6)	84.7 (82.3,86.9)
				**Pairwise G²**	0.0 (0.0,0.4)	35.5 (32.5,38.6)	3.5 (2.4,4.8)	36.3 (33.3,39.4)	5.1 (3.8,6.7)	48.8 (45.7,51.9)
				**Pairwise χ²**	0.0 (0.0,0.4)	35.9 (32.9,39.0)	3.6 (2.5,4.9)	37.0 (34.0,40.1)	5.1 (3.8,6.7)	48.8 (45.7,51.9)
**L**	**L**	**H**	**L**	**Res. Cor.**	0.0 (0.0,0.4)	99.2 (98.4,99.7)	22.1 (19.6,24.8)	98.7 (97.8,99.3)	24.0 (21.4,26.8)	97.8 (96.7,98.6)
				**Pairwise G²**	0.0 (0.0,0.4)	96.5 (65.2,97.5)	7.4 (5.9,9.2)	95.6 (94.1,96.8)	7.7 (6.1,9.5)	91.1 (89.2,92.8)
				**Pairwise χ²**	0.0 (0.0,0.4)	96.9 (95.6,97.9)	7.4 (5.9,9.2)	95.7 (94.2,96.9)	7.8 (6.2,9.6)	91.1 (89.2,92.8)
**L**	**L**	**H**	**H**	**Res. Cor.**	83.1 (80.6,85.4)	99.7 (99.1,99.9)	90.3 (88.3,92.1)	99.7 (99.1,99.9)	90.6 (88.6,92.3)	100.0 (99.6,100.0)
				**Pairwise G²**	67.2 (64.2,70.1)	99.2 (98.4,99.7)	77.6 (74.9,80.1)	99.2 (98.4,99.7)	76.6 (73.8,79.2)	100.0 (99.6,100.0)
				**Pairwise χ²**	67.1 (64.1,70.1)	99.4 (98.7,99.8)	78.6 (75.9,81.1)	99.3 (98.6,99.7)	77.9 (75.2,80.4)	100.0 (99.6,100.0)
**L**	**H**	**L**	**H**	**Res. Cor.**	0.0 (0.0,0.4)	88.4 (86.3,90.3)	96.0 (94.6,97.1)	0.0 (0.0,0.4)	0.0 (0.0,0.4)	100.0 (99.6,100.0)
				**Pairwise G²**	0.0 (0.0,0.4)	64.6 (61.5,67.6)	86.0 (83.7,88.1)	0.0 (0.0,0.4)	0.0 (0.0,0.4)	99.8 (99.3,100.0)

				**Pairwise χ^2**	0.0 (0.0,0.4)	65.0 (62.0,68.0)	86.9 (84.6,88.9)	0.0 (0.0,0.4)	0.0 (0.0,0.4)	99.8 (99.3,100.0)
**Sensitivity: H H H H**										
**Specificity**				**Tool**	**T_1&T_2**	**T_1&T_3**	**T_1&T_4**	**T_2&T_3**	**T_2&T_4**	**T_3&T_4**
				**Res. Cor.**	0.0 (0.0,0.4)	0.1 (0.0,0.6)	0.2 (0.0,0.7)	0.1 (0.0,0.6)	0.2 (0.0,0.7)	100.0 (99.6,100.0)
**H**	**H**	**L**	**L**	**Pairwise G^2**	0.0 (0.0,0.4)	0.0 (0.0,0.4)	0.1 (0.0,0.6)	0.0 (0.0,0.4)	0.1 (0.0,0.6)	97.6 (96.4,98.5)
				**Pairwise χ^2**	0.0 (0.0,0.4)	0.0 (0.0,0.4)	0.1 (0.0,0.6)	0.0 (0.0,0.4)	0.1 (0.0,0.6)	97.6 (96.4,98.5)
				**Res. Cor.**	0.0 (0.0,0.4)	0.0 (0.0,0.4)	0.0 (0.0,0.4)	0.0 (0.0,0.4)	0.2 (0.0,0.7)	100.0 (99.6,100.0)
**H**	**H**	**H**	**L**	**Pairwise G^2**	0.0 (0.0,0.4)	0.0 (0.0,0.4)	0.0 (0.0,0.4)	0.0 (0.0,0.4)	0.2 (0.0,0.7)	100.0 (99.6,100.0)
				**Pairwise χ^2**	0.0 (0.0,0.4)	0.0 (0.0,0.4)	0.0 (0.0,0.4)	0.0 (0.0,0.4)	0.2 (0.0,0.7)	100.0 (99.6,100.0)
				**Res. Cor.**	0.0 (0.0,0.4)	0.0 (0.0,0.4)	0.0 (0.0,0.4)	0.1 (0.0,0.6)	0.1 (0.0,0.6)	100.0 (99.6,100.0)
**H**	**H**	**H**	**H**	**Pairwise G^2**	0.0 (0.0,0.4)	0.0 (0.0,0.4)	0.0 (0.0,0.4)	0.0 (0.0,0.4)	0.0 (0.0,0.4)	100.0 (99.6,100.0)
				**Pairwise χ^2**	0.0 (0.0,0.4)	0.0 (0.0,0.4)	0.0 (0.0,0.4)	0.0 (0.0,0.4)	0.0 (0.0,0.4)	100.0 (99.6,100.0)
				**Res. Cor.**	21.7 (19.2,24.4)	82.0 (79.5,84.3)	84.0 (81.6,86.2)	80.7 (78.1,83.1)	82.4 (79.9,84.7)	100.0 (99.6,100.0)
**L**	**L**	**L**	**L**	**Pairwise G^2**	9.5 (7.8,11.5)	57.2 (54.0,60.3)	60.1 (56.9,63.1)	56.9 (53.7,60.0)	57.0 (53.8,60.1)	99.8 (99.3,100.0)
				**Pairwise χ^2**	9.3 (7.6,11.3)	57.3 (54.1,60.3)	60.3 (57.1,63.3)	56.9 (53.7,60.0)	57.5 (54.3,60.5)	99.8 (99.3,100.0)
				**Res. Cor.**	84.2 (81.7,86.4)	99.8 (99.3,100.0)	92.4 (90.6,93.9)	99.9 (99.4,100.0)	90.7 (88.7,92.4)	100.0 (99.6,100.0)
**L**	**L**	**H**	**L**	**Pairwise G^2**	65.5 (62.5,68.4)	98.7 (97.8,99.3)	72.2 (69.3,75.0)	98.7 (97.8,99.3)	72.7 (69.8,75.5)	100.0 (99.6,100.0)
				**Pairwise χ^2**	65.4 (62.4,68.4)	98.8 (97.9,99.4)	72.3 (69.4,75.1)	98.8 (97.9,99.4)	73.4 (70.6,76.1)	100.0 (99.6,100.0)
				**Res. Cor.**	99.1 (98.3,99.6)	100.0 (99.6,100.0)	100.0 (99.6,100.0)	100.0 (99.6,100.0)	100.0 (99.6,100.0)	100.0 (99.6,100.0)
**L**	**L**	**H**	**H**	**Pairwise G^2**	95.4 (93.9,96.6)	99.6 (99.0,99.9)	99.7 (99.1,99.9)	99.5 (98.8,99.8)	99.9 (99.4,100.0)	100.0 (99.6,100.0)
				**Pairwise χ^2**	95.4 (93.9,96.6)	99.6 (99.0,99.9)	99.7 (99.1,99.9)	99.5 (98.8,99.8)	99.9 (99.4,100.0)	100.0 (99.6,100.0)
				**Res. Cor.**	0.1 (0.0,0.6)	89.5 (87.4,91.3)	100.0 (99.6,100.0)	0.0 (0.0,0.4)	0.0 (0.0,0.4)	100.0 (99.6,100.0)
**L**	**H**	**L**	**H**	**Pairwise G^2**	0.1 (0.0,0.6)	65.7 (62.7,68.6)	100.0 (99.6,100.0)	0.0 (0.0,0.4)	0.0 (0.0,0.4)	100.0 (99.6,100.0)
				**Pairwise χ^2**	0.1 (0.0,0.6)	66.2 (63.2,69.1)	100.0 (99.6,100.0)	0.0 (0.0,0.4)	0.0 (0.0,0.4)	100.0 (99.6,100.0)
**Sensitivity: L L L L**										
**Specificity**				**Tool**	**T_1&T_2**	**T_1&T_3**	**T_1&T_4**	**T_2&T_3**	**T_2&T_4**	**T_3&T_4**
				**Res. Cor.**	0.0 (0.0,0.4)	0.3 (0.1,1.0)	0.1 (0.0,0.6)	0.0 (0.0,0.4)	0.0 (0.0,0.4)	92.2 (90.2,93.8)
**H**	**H**	**L**	**L**	**Pairwise G^2**	0.0 (0.0,0.4)	0.1 (0.0,0.6)	0.1 (0.0,0.6)	0.0 (0.0,0.4)	0.0 (0.0,0.4)	41.2 (38.0,44.5)
				**Pairwise χ^2**	0.0 (0.0,0.4)	0.1 (0.0,0.6)	0.1 (0.0,0.6)	0.0 (0.0,0.4)	0.0 (0.0,0.4)	41.3 (38.1,44.6)
				**Res. Cor.**	0.0 (0.0,0.4)	0.5 (0.2,1.2)	0.0 (0.0,0.4)	0.4 (0.1,1.0)	0.1 (0.0,0.6)	100.0 (99.6,100.0)
**H**	**H**	**H**	**L**	**Pairwise G^2**	0.0 (0.0,0.4)	0.3 (0.1,0.9)	0.0 (0.0,0.4)	0.0 (0.0,0.4)	0.0 (0.0,0.4)	99.7 (99.1,99.9)
				**Pairwise χ^2**	0.0 (0.0,0.4)	0.3 (0.1,0.9)	0.0 (0.0,0.4)	0.0 (0.0,0.4)	0.0 (0.0,0.4)	99.7 (99.1,99.9)
				**Res. Cor.**	0.0 (0.0,0.4)	1.3 (0.7,2.2)	1.5 (0.8,2.5)	1.5 (0.8,2.5)	0.6 (0.2,1.3)	100.0 (99.6,100.0)
**H**	**H**	**H**	**H**	**Pairwise G^2**	0.0 (0.0,0.4)	0.5 (0.2,1.2)	0.8 (0.3,1.6)	0.3 (0.1,0.9)	0.1 (0.0,0.6)	100.0 (99.6,100.0)
				**Pairwise χ^2**	0.0 (0.0,0.4)	0.5 (0.2,1.2)	0.8 (0.3,1.6)	0.3 (0.1,0.9)	0.1 (0.0,0.6)	100.0 (99.6,100.0)
				**Res. Cor.**	0.0 (0.0,0.4)	65.3 (62.1,68.3)	68.5 (65.4,71.5)	68.0 (64.9,70.9)	66.2 (63.1,69.3)	97.2 (95.9,98.2)
**L**	**L**	**L**	**L**	**Pairwise G^2**	0.0 (0.0,0.4)	40.7 (37.6,44.0)	43.3 (40.1,46.6)	42.2 (39.0,45.5)	40.7 (37.6,44.0)	57.6 (54.3,60.8)
				**Pairwise χ^2**	0.0 (0.0,0.4)	41.1 (37.9,44.3)	43.7 (40.5,47.0)	42.4 (39.2,45.7)	41.1 (37.9,44.3)	57.7 (54.4,60.9)
				**Res. Cor.**	0.0 (0.0,0.4)	100.0 (99.6,100.0)	77.4 (74.6,79.9)	100.0 (99.6,100.0)	75.9 (73.1,78.5)	100.0 (99.6,100.0)
**L**	**L**	**H**	**L**	**Pairwise G^2**	0.0 (0.0,0.4)	100.0 (99.6,100.0)	51.7 (48.6,54.8)	100.0 (99.6,100.0)	52.5 (49.4,55.6)	100.0 (99.6,100.0)
				**Pairwise χ^2**	0.0 (0.0,0.4)	100.0 (99.6,100.0)	52.1 (49.0,55.2)	100.0 (99.6,100.0)	52.5 (49.4,55.6)	100.0 (99.6,100.0)

L	L	H	H	Res. Cor.	0.0 (0.0,0.4)	100.0 (99.6,100.0)	100.0 (99.6,100.0)	100.0 (99.6,100.0)	100.0 (99.6,100.0)	100.0 (99.6,100.0)
				Pairwise G^2	0.0 (0.0,0.4)	100.0 (99.6,100.0)	100.0 (99.6,100.0)	100.0 (99.6,100.0)	100.0 (99.6,100.0)	100.0 (99.6,100.0)
				Pairwise χ^2	0.0 (0.0,0.4)	100.0 (99.6,100.0)	100.0 (99.6,100.0)	100.0 (99.6,100.0)	100.0 (99.6,100.0)	100.0 (99.6,100.0)
L	H	L	H	Res. Cor.	0.0 (0.0,0.4)	95.4 (93.9,96.6)	100.0 (99.6,100.0)	0.0 (0.0,0.4)	0.0 (0.0,0.4)	100.0 (99.6,100.0)
				Pairwise G^2	0.0 (0.0,0.4)	81.0 (78.4,83.4)	100.0 (99.6,100.0)	0.0 (0.0,0.4)	0.0 (0.0,0.4)	99.9 (99.4,100.0)
				Pairwise χ^2	0.0 (0.0,0.4)	81.1 (78.5,83.5)	100.0 (99.6,100.0)	0.0 (0.0,0.4)	0.0 (0.0,0.4)	99.9 (99.4,100.0)
**Sensitivity: L L H L**										
**Specificity**				**Tool**	**T_1&T_2**	**T_1&T_3**	**T_1&T_4**	**T_2&T_3**	**T_2&T_4**	**T_3&T_4**
H	H	L	L	Res. Cor.	0.0 (0.0,0.4)	11.8 (8.9,14.0)	0.6 (0.2,1.3)	10.2 (8.4,12.3)	0.2 (0.0,0.7)	100.0 (99.6,100.0)
				Pairwise G^2	0.0 (0.0,0.4)	5.4 (4.1,7.0)	0.1 (0.0,0.6)	3.9 (2.8,5.3)	0.1 (0.0,0.6)	99.5 (98.8,99.8)
				Pairwise χ^2	0.0 (0.0,0.4)	6.0 (4.6,7.7)	0.1 (0.0,0.6)	4.0 (2.9,5.4)	0.1 (0.0,0.6)	99.5 (98.8,99.8)
H	H	H	L	Res. Cor.	0.0 (0.0,0.4)	29.1 (26.3,32.1)	0.2 (0.0,0.7)	27.1 (24.4,30.0)	0.1 (0.0,0.6)	100.0 (99.6,100.0)
				Pairwise G^2	0.0 (0.0,0.4)	17.8 (15.5,20.3)	0.0 (0.0,0.4)	15.8 (13.6,18.2)	0.0 (0.0,0.4)	100.0 (99.6,100.0)
				Pairwise χ^2	0.0 (0.0,0.4)	18.4 (16.1,21.0)	0.0 (0.0,0.4)	16.7 (14.5,19.2)	0.0 (0.0,0.4)	100.0 (99.6,100.0)
H	H	H	H	Res. Cor.	0.0 (0.0,0.4)	43.8 (40.7,46.9)	1.9 (1.1,3.0)	44.5 (41.4,47.6)	3.2 (2.2,4.5)	100.0 (99.6,100.0)
				Pairwise G^2	0.0 (0.0,0.4)	28.2 (25.4,31.1)	0.4 (0.1,1.0)	29.5 (26.7,32.4)	0.7 (0.3,1.4)	100.0 (99.6,100.0)
				Pairwise χ^2	0.0 (0.0,0.4)	30.4 (27.6,33.4)	0.6 (0.2,1.3)	30.9 (28.0,33.9)	0.8 (0.3,1.6)	100.0 (99.6,100.0)
L	L	L	L	Res. Cor.	0.0 (0.0,0.4)	100.0 (99.6,100.0)	80.4 (77.8,82.8)	100.0 (99.6,100.0)	80.3 (77.7,82.7)	100.0 (99.6,100.0)
				Pairwise G^2	0.0 (0.0,0.4)	100.0 (99.6,100.0)	56.5 (53.3,59.6)	100.0 (99.6,100.0)	55.6 (52.4,58.7)	100.0 (99.6,100.0)
				Pairwise χ^2	0.0 (0.0,0.4)	100.0 (99.6,100.0)	56.9 (53.7,60.0)	100.0 (99.6,100.0)	55.6 (52.4,58.7)	100.0 (99.6,100.0)
L	L	H	L	Res. Cor.	0.0 (0.0,0.4)	100.0 (99.6,100.0)	92.2 (90.4,93.8)	100.0 (99.6,100.0)	92.2 (90.4,93.8)	100.0 (99.6,100.0)
				Pairwise G^2	0.0 (0.0,0.4)	100.0 (99.6,100.0)	72.1 (69.2,74.9)	100.0 (99.6,100.0)	71.5 (68.6,74.3)	100.0 (99.6,100.0)
				Pairwise χ^2	0.0 (0.0,0.4)	100.0 (99.6,100.0)	72.2 (69.3,75.0)	100.0 (99.6,100.0)	72.0 (69.1,74.8)	100.0 (99.6,100.0)
L	L	H	H	Res. Cor.	92.7 (90.9,94.2)	100.0 (99.6,100.0)	100.0 (99.6,100.0)	100.0 (99.6,100.0)	100.0 (99.6,100.0)	100.0 (99.6,100.0)
				Pairwise G^2	95.8 (94.4,97.0)	100.0 (99.6,100.0)	100.0 (99.6,100.0)	100.0 (99.6,100.0)	100.0 (99.6,100.0)	100.0 (99.6,100.0)
				Pairwise χ^2	95.8 (94.4,97.0)	100.0 (99.6,100.0)	100.0 (99.6,100.0)	100.0 (99.6,100.0)	100.0 (99.6,100.0)	100.0 (99.6,100.0)
L	H	L	H	Res. Cor.	0.0 (0.0,0.4)	100.0 (99.4,100.0)	100.0 (99.4,100.0)	0.4 (0.1,1.0)	0.0 (0.0,0.4)	100.0 (99.4,100.0)
				Pairwise G^2	0.0 (0.0,0.4)	100.0 (99.6,100.0)	100.0 (99.6,100.0)	0.1 (0.0,0.6)	0.0 (0.0,0.4)	100.0 (99.6,100.0)
				Pairwise χ^2	0.0 (0.0,0.4)	100.0 (99.6,100.0)	100.0 (99.6,100.0)	0.1 (0.0,0.6)	0.0 (0.0,0.4)	100.0 (99.6,100.0)
**Sensitivity: L L H H**										
**Specificity**				**Tool**	**T_1&T_2**	**T_1&T_3**	**T_1&T_4**	**T_2&T_3**	**T_2&T_4**	**T_3&T_4**
H	H	L	L	Res. Cor.	0.0 (0.0,0.4)	12.8 (10.8,15.0)	14.4 (12.3,16.7)	16.1 (13.9,18.5)	13.9 (11.8,16.2)	100.0 (99.6,100.0)
				Pairwise G^2	0.0 (0.0,0.4)	4.9 (3.7,4.0)	5.3 (4.0,6.9)	7.4 (5.9,9.2)	5.4 (4.1,7.0)	100.0 (99.6,100.0)
				Pairwise χ^2	0.0 (0.0,0.4)	5.2 (3.9,6.8)	5.4 (4.1,7.0)	7.6 (6.0,9.4)	5.9 (4.5,7.6)	100.0 (99.6,100.0)
H	H	H	L	Res. Cor.	0.0 (0.0,0.4)	38.2 (35.2,41.3)	19.7 (17.3,22.3)	37.7 (34.7,40.8)	17.4 (15.1,19.9)	100.0 (99.6,100.0)
				Pairwise G^2	0.0 (0.0,0.4)	23.0 (20.4,25.7)	7.8 (6.2,9.6)	23.9 (21.3,26.7)	7.7 (6.1,9.5)	100.0 (99.6,100.0)
				Pairwise χ^2	0.0 (0.0,0.4)	24.6 (22.0,27.4)	9.0 (7.3,10.9)	24.9 (22.2,27.7)	8.1 (6.5,10.0)	100.0 (99.6,100.0)
H	H	H	H	Res. Cor.	0.0 (0.0,0.4)	87.6 (85.4,89.6)	89.4 (87.3,91.2)	88.7 (86.5,90.6)	89.2 (87.1,91.0)	100.0 (99.6,100.0)
				Pairwise G^2	0.0 (0.0,0.4)	79.4 (76.7,81.8)	81.9 (79.3,84.2)	79.4 (76.7,81.8)	80.4 (77.8,82.8)	100.0 (99.6,100.0)
				Pairwise χ^2	0.0 (0.0,0.4)	80.5 (77.9,82.9)	82.9 (80.4,85.2)	81.3 (78.7,83.6)	82.1 (79.5,84.4)	100.0 (99.6,100.0)
L	L	L	L	Res. Cor.	10.8 (9.0,12.9)	100.0 (99.6,100.0)	100.0 (99.6,100.0)	100.0 (99.6,100.0)	100.0 (99.6,100.0)	100.0 (99.6,100.0)

				**Pairwise G^2**	12.5 (10.5,14.7)	100.0 (99.6,100.0)	100.0 (99.6,100.0)	100.0 (99.6,100.0)	100.0 (99.6,100.0)	100.0 (99.6,100.0)
				**Pairwise χ^2**	12.5 (10.5,14.7)	100.0 (99.6,100.0)	100.0 (99.6,100.0)	100.0 (99.6,100.0)	100.0 (99.6,100.0)	100.0 (99.6,100.0)
**L**	**L**	**H**	**L**	**Res. Cor.**	100.0 (99.6,100.0)	100.0 (99.6,100.0)	100.0 (99.6,100.0)	100.0 (99.6,100.0)	100.0 (99.6,100.0)	100.0 (99.6,100.0)
				**Pairwise G^2**	100.0 (99.6,100.0)	100.0 (99.6,100.0)	100.0 (99.6,100.0)	100.0 (99.6,100.0)	100.0 (99.6,100.0)	100.0 (99.6,100.0)
				**Pairwise χ^2**	100.0 (99.6,100.0)	100.0 (99.6,100.0)	100.0 (99.6,100.0)	100.0 (99.6,100.0)	100.0 (99.6,100.0)	100.0 (99.6,100.0)
**L**	**L**	**H**	**H**	**Res. Cor.**	100.0 (99.5,100.0)	12.7 (10.5,15.3)	13.8 (11.4,16.4)	11.8 (9.6,14.3)	13.0 (10.7,15.6)	0.1 (0.0,0.7)
				**Pairwise G^2**	100.0 (99.5,100.0)	4.7 (3.3,6.4)	6.4 (4.7,8.3)	6.1 (4.5,8.0)	5.8 (4.3,7.7)	0.1 (0.0,0.7)
				**Pairwise χ^2**	100.0 (99.5,100.0)	4.7 (3.3,6.4)	6.8 (5.1,8.8)	6.1 (4.5,8.0)	6.2 (4.6,8.2)	0.1 (0.0,0.7)
**L**	**H**	**L**	**H**	**Res. Cor.**	0.1 (0.0,0.6)	100.0 (99.6,100.0)	100.0 (99.6,100.0)	7.5 (6.0,9.3)	0.9 (0.4,1.7)	100.0 (99.6,100.0)
				**Pairwise G^2**	0.5 (0.2,1.2)	100.0 (99.6,100.0)	100.0 (99.6,100.0)	3.0 (2.0,4.3)	0.2 (0.0,0.7)	100.0 (99.6,100.0)
				**Pairwise χ^2**	0.4 (0.1,1.0)	100.0 (99.6,100.0)	100.0 (99.6,100.0)	3.4 (2.4,4.7)	0.2 (0.0,0.7)	100.0 (99.6,100.0)

Table i.4 Mean absolute biases (x10^{-2}) of posterior medians of each parameter obtained from the conditional independence model

**Sensitivity: H H L L**												
**Specificity**				π	Se_1	Se_2	Se_3	Se_4	Sp_1	Sp_2	Sp_3	Sp_4
**H**	**H**	**L**	**L**	-4.4 (-4.4,-4.3)	8.9 (8.8,8.9)	8.9 (8.9,8.9)	0.0 (-0.1,0.0)	-0.1 (-0.2,0.0)	1.0 (0.9,1.0)	1.0 (1.0,1.1)	-1.7 (-1.7, -1.6)	-1.7 (-1.7, -1.6)
**H**	**H**	**H**	**L**	-5.0 (-5.1,-5.0)	9.5 (9.5,9.5)	9.5 (9.5,9.5)	-0.1 (-0.2,-0.1)	0.0 (-0.1,0.0)	0.5 (0.5,0.5)	0.5 (0.4,0.5)	-4.6 (-4.7,-4.6)	-1.8 (-1.9,-1.8)
**H**	**H**	**H**	**H**	-5.0 (-5.0,-4.9)	9.5 (9.5,9.5)	9.5 (9.5,9.5)	-0.1 (-0.1,0.0)	0.0 (-0.1,0.0)	0.5 (0.4,0.5)	0.5 (0.4,0.5)	-4.6 (-4.6,-4.5)	-4.6 (-4.6,-4.6)
**L**	**L**	**L**	**L**	-2.6 (-2.7,-2.5)	8.9 (8.9,8.9)	8.9 (8.9,8.9)	-1.0 (-1.1,-0.9)	-1.1 (-1.1,-1.0)	5.4 (5.4,5.5)	5.4 (5.3,5.4)	-1.9 (-1.9,-1.8)	-2.0 (-2.1,-1.9)
**L**	**L**	**H**	**L**	-4.1 (-4.2,-4.1)	9.8 (9.8,9.8)	9.8 (9.8,9.8)	-2.0 (-2.1,-2.0)	-0.7 (-0.8,-0.6)	4.5 (4.4,4.5)	4.4 (4.3,4.5)	-5.6 (-5.6,-5.5)	-2.1 (-2.2,-2.1)
**L**	**L**	**H**	**H**	-4.6 (-4.7,-4.6)	9.8 (9.8,9.8)	9.8 (9.8,9.8)	-1.4 (-1.5,-1.3)	-1.5 (-1.5,-1.4)	3.9 (3.9,4.0)	3.9 (3.9,4.0)	-5.5 (-5.5,-5.4)	-5.5 (-5.5,-5.4)
**L**	**H**	**L**	**H**	-4.9 (-5.0,-4.9)	9.4 (9.4,9.5)	9.8 (9.8,9.8)	-0.2 (-0.2,-0.1)	-0.4 (-0.5,-0.3)	3.2 (3.1,3.3)	0.8 (0.7,0.8)	-2.0 (-2.0,-1.9)	-4.8 (-4.9,-4.8)
**Sensitivity: H H H L**												
**Specificity**				π	Se_1	Se_2	Se_3	Se_4	Sp_1	Sp_2	Sp_3	Sp_4
**H**	**H**	**L**	**L**	-4.8 (-4.9,-4.8)	9.4 (9.4,9.4)	9.4 (9.4,9.4))	-0.1 (-0.2,-0.1)	0.0 (-0.1,0.0))	0.6 (0.6,0.6)	0.6 (0.5,0.6)	-4.5 (-4.6,-4.4)	-1.8 (-1.9,-1.8)
**H**	**H**	**H**	**L**	-5.2 (-5.2,-5.1)	9.7 (9.7,9.7)	9.7 (9.7,9.7)	-0.1 (-0.1,-0.1)	0.0 (-0.1,0.0)	0.3 (0.3,0.4)	0.4 (0.3,0.4)	-7.5 (-7.6,-7.5)	-1.9 (-1.9,-1.8)
**H**	**H**	**H**	**H**	-5.0 (-5.0,-4.9)	9.4 (9.4,9.5)	9.4 (9.4,9.4)	-0.1 (-0.1,-0.1)	0.1 (0.0,0.1)	0.5 (0.5,0.5)	0.5 (0.5,0.5)	-7.3 (-7.3,-7.3)	-4.5 (-4.5,-4.4)
**L**	**L**	**L**	**L**	-3.9 (-3.9,-3.8)	9.6 (9.6,9.6)	9.6 (9.6,9.6)	-2.0 (-2.1,-2.0)	-0.6 (-0.7,-0.6)	4.6 (4.5,4.7)	4.5 (4.5,4.6)	-5.3 (-5.4,-5.2)	-2.0 (-2.1,-2.0)
**L**	**L**	**H**	**L**	-4.5 (-4.6,-4.4)	9.9 (9.9,9.9)	9.9 (9.9,9.9)	-2.9 (-2.9,-2.8)	-0.3 (-0.3,-0.2)	4.1 (4.0,4.1)	4.1 (4.0,4.2)	-9.1 (-9.1,-9.0)	-1.9 (-2.0,-1.9)
**L**	**L**	**H**	**H**	-5.7 (-5.8,-5.7)	9.8 (9.8,9.9)	9.9 (9.8,9.9)	-0.7 (-0.7,-0.6)	-0.1 (-0.2,0.0)	2.7 (2.6,2.7)	2.6 (2.6,2.7)	-8.7 (-8.8,-8.7)	-5.2 (-5.3,-5.2)
**L**	**H**	**L**	**H**	-5.0 (-5.0,-5.0)	9.4 (9.4,9.4)	9.8 (9.8,9.8)	-0.3 ()-0.3,-0.2	-0.3 (-0.4,-0.2)	3.1 (3.1,3.2)	0.7 (0.7,0.7)	-4.8 (-4.8,-4.7)	-4.8 (-4.9,-4.8)
**Sensitivity: H H H H**												
**Specificity**				π	Se_1	Se_2	Se_3	Se_4	Sp_1	Sp_2	Sp_3	Sp_4
**H**	**H**	**L**	**L**	-4.9 (-5.0,-4.9)	9.5 (9.5,9.5)	9.4 (9.4,9.5)	-0.1 (-0.1,0.0)	-0.1 (-0.1,0.0)	0.5 (0.5,0.6)	0.5 (0.5,0.6)	-4.6 (-4.7,-4.6)	-4.6 (-4.7,-4.6)
**H**	**H**	**H**	**L**	-5.1 (-5.1,-5.0)	9.5 (9.5,9.5)	9.5 (9.5,9.5)	0.0 (-0.1,0.0)	0.0 (0.0,0.0)	0.4 (0.4,0.5)	0.4 (0.4,0.5)	-7.4 (-7.4,-7.4)	-4.5 (-4.6,-4.5)
**H**	**H**	**H**	**H**	-4.9 (-4.9,-4.8)	9.3 (9.3,9.3)	9.3 (9.3,9.3)	0.0 (-0.1,0.0)	0.0 (-0.1,0.0)	0.6 (0.5,0.6)	0.6 (0.5,0.6)	-7.1 (-7.2,-7.1)	-7.1 (-7.2,-7.1)
**L**	**L**	**L**	**L**	-4.9 (-5.0,-4.9)	9.8 (9.7,9.8)	9.8 (9.7,9.8)	-0.7 (-0.7,-0.6)	-0.7 (-0.8,-0.7)	3.5 (3.4,3.6)	3.5 (3.4,3.6)	-5.1 (-5.2,-5.0)	-5.1 (-5.2,-5.0)
**L**	**L**	**H**	**L**	-5.8 (-5.9.-5.7)	9.9 (9.9,9.9)	9.9 (9.9,9.9)	-0.5 (-0.6,-0.5)	0.4 (0.4,0.4)	2.6 (2.5,2.7)	2.6 (2.5,2.6)	-8.8 (-8.8,-8.7)	-4.9 (-5.0,-4.9)

**L**	**L**	**H**	**H**	-6.5 (-6.6,-6.5)	9.8 (9.8,9.8)	9.8 (9.8,9.8)	0.9 (0.9,1.0)	0.9 (0.9,1.0)	1.7 (1.7,1.8)	1.8 (1.7,1.8)	-8.5 (-8.5,-8.4)	-8.5 (-8.5,-8.4)
**L**	**H**	**L**	**H**	-5.2 (-5.2,-5.1)	9.4 (9.4,9.4)	9.9 (9.9,9.9)	0.0 (-0.1,0.0)	-0.2 (-0.3,-0.2)	2.9 (2.9,3.0)	0.4 (0.4,0.5)	-4.8 (-4.8,-4.7)	-7.7 (-7.8,-7.7)
**Sensitivity: L L L L**												
**Specificity**				π	Se_1	Se_2	Se_3	Se_4	Sp_1	Sp_2	Sp_3	Sp_4
**H**	**H**	**L**	**L**	-20.1 (-20.2,-20.1)	38.4 (38.4,38.5)	38.4 (38.4,38.5)	-0.1 (-0.2,0.0)	-0.2 (-0.3,-0.1)	1.9 (1.8,1.9)	1.9 (1.8,1.9)	-5.8 (-5.9,-5.8)	-5.8 (-5.9,-5.8)
**H**	**H**	**H**	**L**	-20.8 (-20.9,-20.8)	39.5 (39.4,39.5)	39.5 (39.4,39.5)	-0.3 (-0.4,-0.3)	-0.1 (-0.2,0.0)	1.5 (1.4,1.5)	1.5 (1.4,1.5)	-14.9 (-14.9,-14.8)	-5.9 (-6.0,-5.9)
**H**	**H**	**H**	**H**	-20.8 (-20.9,-20.8)	39.4 (39.4,39.4)	39.4 (39.3,39.4)	-0.2 (-0.3,-0.1)	-0.3 (-0.4,-0.2)	1.5 (1.4,1.5)	1.5 (1.4,1.5)	-14.8 (-14.9,-14.8)	-14.8 (-14.8,-14.8)
**L**	**L**	**L**	**L**	-16.7 (-16.8,-16.7)	38.9 (38.9,39.0)	38.9 (38.9,38.9)	-3.2 (-3.3,-3.2)	-3.2 (-3.3,-3.1)	14.2 (14.2,14.3)	14.2 (14.1,14.2)	-6.6 (-6.7,-6.6)	-6.7 (-6.7,-6.6)
**L**	**L**	**H**	**L**	-17.8 (-17.8,-17.7)	39.8 (39.7,39.8)	39.8 (39.8,39.8)	-7.5 (-7.6,-7.4)	-2.8 (-2.8,-2.7)	13.6 (13.5,13.6)	13.6 (13.5,13.6)	-16.7 (-16.8,-16.7)	-6.6 (-6.7,-6.7)
**L**	**L**	**H**	**H**	-18.2 (-18.3,-18.2)	39.8 (39.8,39.8)	39.8 (39.8,39.8)	-6.8 (-6.9,-6.8)	-6.8 (-6.9,-6.7)	13.3 (13.2,13.3)	13.3 (13.2,13.3)	-16.5 (-16.6,-16.5)	-16.6 (-16.6,-16.5)
**L**	**H**	**L**	**H**	-20.5 (-20.5,-20.4)	39.0 (39.0,39.1)	39.8 (39.8,39.8)	-0.5 (-0.5,-0.4)	-1.5 (-1.6,-1.4)	10.5 (10.5,10.6)	2.1 (2.0,2.1)	-6.1 (-6.1,-6.0)	-15.2 (-15.2,-15.1)
**Sensitivity: L L H L**												
**Specificity**				π	Se_1	Se_2	Se_3	Se_4	Sp_1	Sp_2	Sp_3	Sp_4
**H**	**H**	**L**	**L**	-20.8 (-20.8,-20.7)	39.4 (39.3,39.4)	39.4 (39.4,39.4)	-0.3 (-0.4,-0.2)	-0.1 (-0.1,0.0)	1.5 (1.5,1.5)	1.5 (1.4,1.5)	-14.8 (-14.9,-14.8)	-5.9 (-6.0,-5.9)
**H**	**H**	**H**	**L**	-21.1 (-21.1,-21.0)	39.7 (39.7,39.7)	39.7 (39.7,39.7)	-0.4 (-0.5,-0.4)	-0.1 (-0.2,0.0)	1.3 (1.3,1.4)	1.3 (1.3,1.3)	-23.9 (-23.9,-23.8)	-6.0 (-6.0,-5.9)
**H**	**H**	**H**	**H**	-20.8 (-20.9,-20.8)	39.3 (39.2,39.3)	39.3 (39.2,39.3	-0.3 (-0.4,-0.3)	0.0 (-0.1,0.1)	1.5 (1.4,1.5)	1.5 (1.5,1.5)	-23.6 (-23.7,-23.6)	-14.7 (-14.7,-14.6)
**L**	**L**	**L**	**L**	-17.7 (-17.7,-17.6)	39.7 (39.7,39.7)	39.7 (39.7,39.7)	-7.4 (-7.5,-7.3)	-2.7 (-2.7,-2.6)	13.6 (13.5,13.7)	13.6 (13.5,13.6)	-16.6 (-16.7,-16.6)	-6.5 (-6.6,-6.4)
**L**	**L**	**H**	**L**	-18.1 (-18.2,-18.1)	39.9 (39.9,39.9)	39.9 (39.9,39.9)	-11.7 (-11.8,-11.6)	-2.2 (-2.3,-2.2)	13.4 (13.4,13.5)	13.3 (13.3,13.4)	-26.7 (-26.8,-26.7)	-6.3 (-6.4,-6.3)
**L**	**L**	**H**	**H**	-19.4 (-19.4),-19.3)	39.9 (39.9,39.9)	39.9 (39.9,39.9)	-8.5 (-8.6,-8.4)	-4.3 (-4.4,-4.2)	12.0 (11.9,12.0)	11.9 (11.9,12.0)	-26.2 (-26.2,-26.1)	-15.9 (-16.0,-15.9)
**L**	**H**	**L**	**H**	-20.6 (-20.6,-20.5)	38.9 (38.8,38.9)	39.8 (39.8,39.8)	-1.2 (-1.2,-1.1)	-1.2 (-1.3,-1.1)	10.4 (10.3,10.4)	2.1 (2.0,2.1)	-15.1 (-15.1,-15.0)	-15.1 (-15.1,-15.1)
**Sensitivity: L L H H**												
**Specificity**				π	Se_1	Se_2	Se_3	Se_4	Sp_1	Sp_2	Sp_3	Sp_4
**H**	**H**	**L**	**L**	-20.8 (-20.9,-20.8)	39.4 (39.4,39.4)	39.4 (39.3,39.4)	-0.2 (-0.3,-0.2)	-0.2 (-0.2,-0.1)	1.4 (1.4,1.5)	1.5 (1.4,1.5)	-14.8 (-14.9,-14.8)	14.8 (-14.9,-14.7)
**H**	**H**	**H**	**L**	-20.9 (-20.9,-20.8)	39.4 (39.4,39.4)	39.4 (39.4,39.4)	-0.3 (-0.3,-0.2)	-0.1 (-0.2,-0.1)	1.4 (1.4,1.4)	1.4 (1.4,1.4)	-23.7 (-23.8,-23.7)	-14.8 (-14.8,-14.7)
**H**	**H**	**H**	**H**	-20.4 (-20.5,-20.4)	38.6 (38.5,38.6)	38.6 (38.5,38.6)	-0.1 (-0.2,-0.1)	-0.1 (-0.1,0.0)	1.7 (1.7,1.7)	1.7 (1.7,1.7)	-23.2 (-23.3,-23.2)	-23.3 (-23.3,-23.2)

**L**	**L**	**L**	**L**	-18.7 (-18.8,-18.7)	39.8 (39.8,39.8)	39.8 (39.8,39.8)	-5.4 (-5.5,-5.3)	-5.3 (-5.3,-5.2)	12.7 (12.6,12.7)	12.7 (12.6,12.7)	-16.1 (-16.2,-16.1)	-16.1 (-16.2,-16.0)
**L**	**L**	**H**	**L**	-20.4 (-20.5,-20.4)	39.9 (39.9,39.9)	39.9 (39.9,39.9)	-6.2 (-6.3,-6.1)	-1.6 (-1.7,-1.5)	11.0 (10.9,11.0)	10.9 (10.9,11.0)	-25.8 (-25.9,-25.8)	-15.2 (-15.2,-15.1)
**L**	**L**	**H**	**H**	-1.2 (-1.3,-1.1)	1.7 (1.5,1.8)	1.7 (1.5,1.8)	0.8 (0.7,0.9)	0.8 (0.7,0.9)	1.0 (1.0,1.1)	1.1 (1.0,1.2)	-1.2 (-1.3,-1.1)	-1.2 (-1.3,-1.1)
**L**	**H**	**L**	**H**	-20.9 (-20.9,-20.8)	38.9 (38.9,38.9)	39.9 (39.9,39.9)	-0.3 (-0.4,-0.3)	-1.3 (-1.4,-1.3)	10.1 (10.0,10.1)	1.7 (1.6,1.7)	-14.9 (-14.9,-14.8)	-24.1 (-24.2,-24.1)

Table i.5 Coverages of the 95% CrIs for each model parameter

**Sensitivity: H H L L**												
**Specificity**				π	Se_1	Se_2	Se_3	Se_4	Sp_1	Sp_2	Sp_3	Sp_4
**H**	**H**	**L**	**L**	0.5 (0.1,1.1)	0.0 (0.0,0.0)	0.0 (0.0,0.0)	94.0 (92.4,95.5)	95.1 (93.7,96.5)	83.0 (80.6,85.4)	81.1 (78.6,83.6)	59.6 (56.5,62.8)	59.4 (56.3,62.5)
**H**	**H**	**H**	**L**	0.0 (0.0,0.0)	0.0 (0.0,0.0)	0.0 (0.0,0.0)	95.0 (93.6,96.4)	95.0 (93.6,96.4)	89.3 (87.4,91.2)	89.6 (87.7,91.5)	0.0 (0.0,0.0)	51.0 (47.9,54.1)
**H**	**H**	**H**	**H**	0.0 (0.0,0.0)	0.0 (0.0,0.0)	0.0 (0.0,0.0)	94.9 (93.5,96.3)	94.6 (93.2,96.0)	89.3 (87.4,91.2)	88.1 (86.1,90.1)	0.0 (0.0,0.0)	0.0 (0.0,0.0)
**L**	**L**	**L**	**L**	69.9 (66.9,72.9)	0.0 (0.0,0.0)	0.0 (0.0,0.0)	85.3 (83.0,87.6)	85.1 (82.8,87.4)	0.5 (0.1,1.0)	1.2 (0.5,1.9)	57.7 (54.5,60.9)	54.0 (50.8,57.2)
**L**	**L**	**H**	**L**	0.7 (0.2,1.2)	0.0 (0.0,0.0)	0.0 (0.0,0.0)	60.4 (57.4,63.4)	90.5 (88.7,82.3)	3.7 (2.5,4.9)	5.0 (3.6,6.4)	0.0 (0.0,0.0)	44.6 (41.5,47.7)
**L**	**L**	**H**	**H**	0.2 (0.0,0.5)	0.0 (0.0,0.0)	0.0 (0.0,0.0)	76.8 (74.2,79.4)	76.3 (73.7,78.9)	6.4 (4.9,4.9)	7.9 (6.2,9.6)	0.0 (0.0,0.0)	0.0 (0.0,0.0)
**L**	**H**	**L**	**H**	0.0 (0.0,0.0)	0.0 (0.0,0.0)	0.0 (0.0,0.0)	96.2 (95.0,97.4)	93.5 (92.0,95.0)	9.9 (8.0,11.8)	86.2 (84.1,88.3)	46.7 (43.6,49.8)	0.0 (0.0,0.0)
**Sensitivity: H H H L**												
**Specificity**				π	Se_1	Se_2	Se_3	Se_4	Sp_1	Sp_2	Sp_3	Sp_4
**H**	**H**	**L**	**L**	0.0 (0.0,0.0)	0.0 (0.0,0.0)	0.0 (0.0,0.0)	93.0 (91.4,94.6)	93.7 (92.2,95.2)	86.6 (84.5,88.7)	89.4 (87.5,91.3)	0.7 (0.2,1.2)	48.1 (45.0,51.2)
**H**	**H**	**H**	**L**	0.0 (0.0,0.0)	0.0 (0.0,0.0)	0.0 (0.0,0.0)	94.9 (93.5,96.3)	93.6 (92.1,95.1)	91.7 (90.0,93.4)	90.6 (88.8,92.4)	0.0 (0.0,0.0)	49.7 (46.6,52.8)
**H**	**H**	**H**	**H**	0.0 (0.0,0.0)	0.0 (0.0,0.0)	0.0 (0.0,0.0)	94.4 (93.0,95.8)	95.7 (94.4,97.0)	87.2 (85.1,89.3)	88.7 (86.7,90.7)	0.0 (0.0,0.0)	0.0 (0.0,0.0)
**L**	**L**	**L**	**L**	1.5 (0.7,2.3)	0.0 (0.0,0.0)	0.0 (0.0,0.0)	44.5 (41.4,47.6)	91.9 (90.2,93.6)	2.1 (1.2,3.0)	2.9 (1.9,3.9)	-0.1 (0.1,0.3)	48.2 (45.1,51.3)
**L**	**L**	**H**	**L**	0.0 (0.0,0.0)	0.0 (0.0,0.0)	0.0 (0.0,0.0)	31.8 (28.9,34.7)	94.7 (93.3,96.1)	5.4 (4.0,6.8)	5.6 (4.2,7.0)	0.0 (0.0,0.0)	49.1 (46.0,52.2)
**L**	**L**	**H**	**H**	0.0 (0.0,0.0)	0.0 (0.0,0.0)	0.0 (0.0,0.0)	88.1 (86.1,90.1)	94.0 (92.5,95.5)	28.8 (26.0,31.6)	30.6 (27.8,33.5)	0.0 (0.0,0.0)	0.0 (0.0,0.0)
**L**	**H**	**L**	**H**	0.0 (0.0,0.0)	0.0 (0.0,0.0)	0.0 (0.0,0.0)	93.7 (92.2,95.2)	94.4 (93.0,95.8)	10.7 (8.8,12.6)	86.9 (84.8,89.0)	0.1 (0.0,0.3)	0.0 (0.0,0.0)
**Sensitivity: H H H H**												
**Specificity**				π	Se_1	Se_2	Se_3	Se_4	Sp_1	Sp_2	Sp_3	Sp_4
**H**	**H**	**L**	**L**	0.0 (0.0,0.0)	0.0 (0.0,0.0)	0.0 (0.0,0.0)	95.6 (94.3,96.9)	94.6 (93.2,96.0)	89.2 (87.3,91.1)	87.2 (85.1,89.3)	0.2 (0.0,0.5)	0.1 (0.0,0.3)
**H**	**H**	**H**	**L**	0.0 (0.0,0.0)	0.0 (0.0,0.0)	0.0 (0.0,0.0)	95.6 (94.3,96.9)	95.1 (93.8,96.4)	90.1 (88.2,92.0)	90.6 (88.8,92.4)	0.0 (0.0,0.0)	0.6 (0.1,1.1)
**H**	**H**	**H**	**H**	0.0 (0.0,0.0)	0.0 (0.0,0.0)	0.0 (0.0,0.0)	94.8 (93.4,96.2)	96.0 (94.8,97.2)	85.8 (83.6,88.0)	84.5 (82.3,86.7)	0.0 (0.0,0.0)	0.0 (0.0,0.0)
**L**	**L**	**L**	**L**	0.0 (0.0,0.0)	0.0 (0.0,0.0)	0.0 (0.0,0.0)	87.9 (85.9,89.9)	86.8 (84.7,88.9)	12.0 (10.0,14.0)	12.9 (10.8,15.0)	0.0 (0.0,0.0)	0.1 (0.0,0.3)
**L**	**L**	**H**	**L**	0.0 (0.0,0.0)	0.0 (0.0,0.0)	0.0 (0.0,0.0)	90.2 (88.3,92.0)	92.3 (90.6,93.9)	32.9 (30.0,35.8)	33.3 (30.4,36.2)	0.0 (0.0,0.0)	0.1 (0.0,0.3)
**L**	**L**	**H**	**H**	0.0 (0.0,0.0)	0.0 (0.0,0.0)	0.0 (0.0,0.0)	77.8 (75.2,80.4)	79.4 (76.9,81.9)	59.0 (56.0,62.0)	57.4 (54.3,60.5)	0.0 (0.0,0.0)	0.0 (0.0,0.0)
**L**	**H**	**L**	**H**	0.0 (0.0,0.0)	0.0 (0.0,0.0)	0.0 (0.0,0.0)	95.5 (94.2,96.8)	94.0 (92.5,95.5)	14.6 (12.4,16.8)	90.7 (88.9,92.5)	0.1 (0.0,0.3)	0.0 (0.0,0.0)
**Sensitivity: L L L L**												
**Specificity**				π	Se_1	Se_2	Se_3	Se_4	Sp_1	Sp_2	Sp_3	Sp_4
**H**	**H**	**L**	**L**	0.0 (0.0,0.0)	0.0 (0.0,0.0)	0.0 (0.0,0.0)	95.0 (93.6,96.5)	96.4 (95.1,97.6)	13.7 (11.4,15.9)	13.5 (11.3,15.8)	0.0 (0.0,0.0)	0.0 (0.0,0.0)

**H**	**H**	**H**	**L**	0.0 (0.0,0.0)	0.0 (0.0,0.0)	0.0 (0.0,0.0)	94.1 (92.6,95.6)	93.7 (92.2,95.2)	24.7 (22.0,27.4)	21.4 (18.9,24.0)	0.0 (0.0,0.0)	0.0 (0.0,0.0)
**H**	**H**	**H**	**H**	0.0 (0.0,0.0)	0.0 (0.0,0.0)	0.0 (0.0,0.0)	96.0 (94.8,97.2)	96.1 (94.9,97.3)	23.5 (20.9,26.1)	22.5 (19.9,25.1)	0.0 (0.0,0.0)	0.0 (0.0,0.0)
**L**	**L**	**L**	**L**	0.0 (0.0,0.0)	0.0 (0.0,0.0)	0.0 (0.0,0.0)	32.5 (29.5,35.5)	32.2 (29.2,35.2)	0.0 (0.0,0.0)	0.0 (0.0,0.0)	0.0 (0.0,0.0)	0.0 (0.0,0.0)
**L**	**L**	**H**	**L**	0.0 (0.0,0.0)	0.0 (0.0,0.0)	0.0 (0.0,0.0)	0.0 (0.0,0.0)	46.0 (42.9,49.1)	0.0 (0.0,0.0)	0.0 (0.0,0.0)	0.0 (0.0,0.0)	0.0 (0.0,0.0)
**L**	**L**	**H**	**H**	0.0 (0.0,0.0)	0.0 (0.0,0.0)	0.0 (0.0,0.0)	0.3 (0.0,0.6)	0.3 (0.0,0.6)	0.0 (0.0,0.0)	0.0 (0.0,0.0)	0.0 (0.0,0.0)	0.0 (0.0,0.0)
**L**	**H**	**L**	**H**	0.0 (0.0,0.0)	0.0 (0.0,0.0)	0.0 (0.0,0.0)	94.2 (92.7,95.6)	80.6 (78.1,83.0)	0.0 (0.0,0.0)	10.1 (8.2,12.0)	0.0 (0.0,0.0)	0.0 (0.0,0.0)
**Sensitivity: L L H L**												
**Specificity**				π	Se_1	Se_2	Se_3	Se_4	Sp_1	Sp_2	Sp_3	Sp_4
**H**	**H**	**L**	**L**	0.0 (0.0,0.0)	0.0 (0.0,0.0)	0.0 (0.0,0.0)	93.2 (91.6,94.7)	94.7 (93.3,96.1)	21.4 (18.9,24.0)	23.0 (20.4,25.7)	0.0 (0.0,0.0)	0.0 (0.0,0.0)
**H**	**H**	**H**	**L**	0.0 (0.0,0.0)	0.0 (0.0,0.0)	0.0 (0.0,0.0)	92.3 (90.6,93.9)	96.3 (95.1,97.5)	27.7 (25.0,30.5)	28.3 (25.5,31.1)	0.0 (0.0,0.0)	0.0 (0.0,0.0)
**H**	**H**	**H**	**H**	0.0 (0.0,0.0)	0.0 (0.0,0.0)	0.0 (0.0,0.0)	94.0 (92.5,95.5)	94.3 (92.9,95.7)	20.9 (18.4,23.4)	19.9 (17.4,22.4)	0.0 (0.0,0.0)	0.0 (0.0,0.0)
**L**	**L**	**L**	**L**	0.0 (0.0,0.0)	0.0 (0.0,0.0)	0.0 (0.0,0.0)	0.0 (0.0,0.0)	49.6 (46.5,52.8)	0.0 (0.0,0.0)	0.0 (0.0,0.0)	0.0 (0.0,0.0)	0.0 (0.0,0.0)
**L**	**L**	**H**	**L**	0.0 (0.0,0.0)	0.0 (0.0,0.0)	0.0 (0.0,0.0)	0.0 (0.0,0.0)	60.7 (57.7,63.7)	0.0 (0.0,0.0)	0.0 (0.0,0.0)	0.0 (0.0,0.0)	0.0 (0.0,0.0)
**L**	**L**	**H**	**H**	0.0 (0.0,0.0)	0.0 (0.0,0.0)	0.0 (0.0,0.0)	0.1 (0.0,0.3)	20.3 (17.8,22.8)	0.0 (0.0,0.0)	0.0 (0.0,0.0)	0.0 (0.0,0.0)	0.0 (0.0,0.0)
**L**	**H**	**L**	**H**	0.0 (0.0,0.0)	0.0 (0.0,0.0)	0.0 (0.0,0.0)	74.3 (71.6,77.0)	85.8 (83.6,88.0)	0.0 (0.0,0.0)	10.8 (8.9,12.7)	0.0 (0.0,0.0)	0.0 (0.0,0.0)
**Sensitivity: L L H H**												
**Specificity**				π	Se_1	Se_2	Se_3	Se_4	Sp_1	Sp_2	Sp_3	Sp_4
**H**	**H**	**L**	**L**	0.0 (0.0,0.0)	0.0 (0.0,0.0)	0.0 (0.0,0.0)	94.9 (93.5,96.3)	95.2 (93.9,96.5)	23.0 (20.4,25.6)	20.8 (18.3,23.3)	0.0 (0.0,0.0)	0.0 (0.0,0.0)
**H**	**H**	**H**	**L**	0.0 (0.0,0.0)	0.0 (0.0,0.0)	0.0 (0.0,0.0)	92.6 (91.0,94.2)	94.8 (93.4,96.2)	25.2 (22.5,27.9)	24.7 (22.0,27.4)	0.0 (0.0,0.0)	0.0 (0.0,0.0)
**H**	**H**	**H**	**H**	0.0 (0.0,0.0)	0.0 (0.0,0.0)	0.0 (0.0,0.0)	94.8 (93.4,96.2)	96.3 (95.1,97.5)	11.6 (9.6,13.6)	14.0 (11.9,16.2)	0.0 (0.0,0.0)	0.0 (0.0,0.0)
**L**	**L**	**L**	**L**	0.0 (0.0,0.0)	0.0 (0.0,0.0)	0.0 (0.0,0.0)	0.3 (0.0,0.6)	0.6 (0.1,1.1)	0.0 (0.0,0.0)	0.0 (0.0,0.0)	0.0 (0.0,0.0)	0.0 (0.0,0.0)
**L**	**L**	**H**	**L**	0.0 (0.0,0.0)	0.0 (0.0,0.0)	0.0 (0.0,0.0)	3.0 (1.9,4.1)	69.6 (66.7,72.4)	0.0 (0.0,0.0)	0.0 (0.0,0.0)	0.0 (0.0,0.0)	0.0 (0.0,0.0)
**L**	**L**	**H**	**H**	84.3 (81.7,86.9)	66.4 (63.0,69.7)	68.2 (64.9,71.5)	88.8 (86.6,91.1)	91.2 (89.2,93.2)	82.7 (80.1,85.4)	81.8 (79.1,84.5)	85.3 (82.8,87.8)	83.0 (80.3,85.6)
**L**	**H**	**L**	**H**	0.0 (0.0,0.0)	0.0 (0.0,0.0)	0.0 (0.0,0.0)	93.1 (91.5,94.7)	72.7 (69.9,75.4)	0.0 (0.0,0.0)	24.2 (21.6,26.9)	0.0 (0.0,0.0)	0.0 (0.0,0.0)

ii. Setting 2: $\pi = 0.5, \omega = 0.5, n_{obs} = 5000$

Table ii.1 Number of converged data sets for different sensitivity-specificity combinations

				**Number of converged data sets**
**Specificity**				**Sensitivity: H H L L**
**H**	**H**	**L**	**L**	820
**H**	**H**	**H**	**L**	1000
**H**	**H**	**H**	**H**	1000
**L**	**L**	**L**	**L**	657
**L**	**L**	**H**	**L**	978
**L**	**L**	**H**	**H**	997
**L**	**H**	**L**	**H**	998

Specificity				Sensitivity: H H H L
H	H	L	L	999
H	H	H	L	1000
H	H	H	H	1000
L	L	L	L	949
L	L	H	L	990
L	L	H	H	1000
L	H	L	H	1000
**Specificity**				**Sensitivity: H H H H**
H	H	L	L	1000
H	H	H	L	1000
H	H	H	H	1000
L	L	L	L	988
L	L	H	L	1000
L	L	H	H	1000
L	H	L	H	1000
**Specificity**				**Sensitivity: L L L L**
H	H	L	L	487
H	H	H	L	962
H	H	H	H	993
L	L	L	L	380
L	L	H	L	938
L	L	H	H	910
L	H	L	H	916
**Specificity**				**Sensitivity: L L H L**
H	H	L	L	981
H	H	H	L	1000
H	H	H	H	978
L	L	L	L	926
L	L	H	L	986
L	L	H	H	846
L	H	L	H	952
**Specificity**				**Sensitivity: L L H H**
H	H	L	L	998
H	H	H	L	1000
H	H	H	H	1000
L	L	L	L	914
L	L	H	L	690
L	L	H	H	914
L	H	L	H	856

Table ii.2 Percentages of the time 95% credible intervals (CrIs) for residual correlations did not included '0' for any pairs, and percentages of the time that the overall χ^2 or G^2 statistic indicated a lack of overall fit

**Sensitivity: H H L L**						
**Specificity**				**Res. Cor.**	**Overall G^2**	**Overall χ^2**
**H**	**H**	**L**	**L**	55.7 (52.3,59.2)	6.7 (5.1,8.6)	6.6 (5.0,8.5)
**H**	**H**	**H**	**L**	40.8 (37.7,43.9)	17.9 (15.6,20.4)	17.9 (15.6,20.4)
**H**	**H**	**H**	**H**	86.9 (84.6,88.9)	98.9 (98.0,99.4)	99.3 (98.6,99.7)
**L**	**L**	**L**	**L**	58.3 (20.7,27.3)	12.8 (10.3,15.6)	12.9 (10.5,15.7)
**L**	**L**	**H**	**L**	72.6 (69.7,75.4)	53.3 (50.1,56.4)	54.1 (50.9,57.2)
**L**	**L**	**H**	**H**	99.4 (98.7,99.8)	100.0 (99.6,100.0)	100.0 (99.6,100.0)
**L**	**H**	**L**	**H**	60.6 (57.5,63.7)	36.3 (33.3,39.3)	36.9 (33.9,40.0)
**Sensitivity: H H H L**						
**Specificity**				**Res. Cor.**	**Overall G^2**	**Overall χ^2**
**H**	**H**	**L**	**L**	43.2 (40.1,46.4)	12.4 (10.4,14.6)	12.0 (10.1,14.2)
**H**	**H**	**H**	**L**	34.1 (31.2,37.1)	43.2 (40.1,46.3)	43.5 (40.4,46.6)
**H**	**H**	**H**	**H**	99.6 (99.0,99.9)	100.0 (99.6,100.0)	100.0 (99.6,100.0)
**L**	**L**	**L**	**L**	71.0 (68.0,73.9)	34.9 (31.8,38.0)	35.2 (32.2,38.3)
**L**	**L**	**H**	**L**	87.4 (85.1,89.4)	91.0 (89.1,92.7)	91.4 (89.5,93.1)
**L**	**L**	**H**	**H**	100.0 (99.6,100.0)	100.0 (99.6,100.0)	100.0 (99.6,100.0)
**L**	**H**	**L**	**H**	98.3 (97.3,99.0)	98.9 (98.0,99.4)	99.0 (98.2,99.5)
**Sensitivity: H H H H**						
**Specificity**				**Res. Cor.**	**Overall G^2**	**Overall χ^2**
**H**	**H**	**L**	**L**	89.2 (87.1,91.1)	55.8 (52.7,58.9)	55.3 (52.2,58.4)
**H**	**H**	**H**	**L**	99.9 (99.4,100.0)	100.0 (99.6,100.0)	100.0 (99.6,100.0)
**H**	**H**	**H**	**H**	100.0 (99.6,100.0)	100.0 (99.6,100.0)	100.0 (99.6,100.0)
**L**	**L**	**L**	**L**	99.9 (99.4,100.0)	98.1 (97.0,98.8)	98.2 (97.1,98.9)
**L**	**L**	**H**	**L**	100.0 (99.6,100.0)	100.0 (99.6,100.0)	100.0 (99.6,100.0)
**L**	**L**	**H**	**H**	100.0 (99.6,100.0)	100.0 (99.6,100.0)	100.0 (99.6,100.0)
**L**	**H**	**L**	**H**	100.0 (99.6,100.0)	100.0 (99.6,100.0)	100.0 (99.6,100.0)
**Sensitivity: L L L L**						
**Specificity**				**Res. Cor.**	**Overall G^2**	**Overall χ^2**
**H**	**H**	**L**	**L**	84.0 (80.4,87.1)	19.1 (15.7,22.9)	19.1 (15.7,22.9)
**H**	**H**	**H**	**L**	99.9 (99.4,100.0)	95.1 (93.6,96.4)	95.8 (93.8,96.6)
**H**	**H**	**H**	**H**	100.0 (99.6,100.0)	100.0 (99.6,100.0)	100.0 (99.6,100.0)
**L**	**L**	**L**	**L**	99.2 (97.7,99.8)	82.4 (78.2,86.1)	82.9 (78.7,86.5)
**L**	**L**	**H**	**L**	100.0 (99.6,100.0)	100.0 (99.6,100.0)	100.0 (99.6,100.0)
**L**	**L**	**H**	**H**	100.0 (99.6,100.0)	100.0 (99.6,100.0)	100.0 (99.6,100.0)
**L**	**H**	**L**	**H**	100.0 (99.6,100.0)	100.0 (99.6,100.0)	100.0 (99.6,100.0)

Sensitivity: L L H L						
Specificity				Res. Cor.	Overall G^2	Overall χ^2
H	H	L	L	99.5 (98.8,99.8)	88.0 (85.8,89.9)	88.1 (85.9,90.0)
H	H	H	L	100.0 (99.6,100.0)	100.0 (99.6,100.0)	100.0 (99.6,100.0)
H	H	H	H	100.0 (99.6,100.0)	100.0 (99.6,100.0)	100.0 (99.6,100.0)
L	L	L	L	100.0 (99.6,100.0)	100.0 (99.6,100.0)	100.0 (99.6,100.0)
L	L	H	L	100.0 (99.6,100.0)	100.0 (99.6,100.0)	100.0 (99.6,100.0)
L	L	H	H	100.0 (99.6,100.0)	100.0 (99.6,100.0)	100.0 (99.6,100.0)
L	H	L	H	100.0 (99.6,100.0)	100.0 (99.6,100.0)	100.0 (99.6,100.0)
Sensitivity: L L H H						
Specificity				Res. Cor.	Overall G^2	Overall χ^2
H	H	L	L	100.0 (99.6,100.0)	100.0 (99.6,100.0)	100.0 (99.6,100.0)
H	H	H	L	100.0 (99.6,100.0)	100.0 (99.6,100.0)	100.0 (99.6,100.0)
H	H	H	H	100.0 (99.6,100.0)	100.0 (99.6,100.0)	100.0 (99.6,100.0)
L	L	L	L	100.0 (99.6,100.0)	100.0 (99.6,100.0)	100.0 (99.6,100.0)
L	L	H	L	100.0 (99.5,100.0)	100.0 (99.5,100.0)	100.0 (99.5,100.0)
L	L	H	H	100.0 (99.6,100.0)	100.0 (99.6,100.0)	100.0 (99.6,100.0)
L	H	L	H	100.0 (99.6,100.0)	100.0 (99.6,100.0)	100.0 (99.6,100.0)

Table ii.3 Percentages of the time 95% CrIs for residual correlations did not included '0' for each pair, and percentages of the time the pairwise χ^2 or G^2 statistic for each pair indicated a lack of pairwise fit

Sensitivity: H H L L										
Specificity				Tool	T_1&T_2	T_1&T_3	T_1&T_4	T_2&T_3	T_2&T_4	T_3&T_4
H	H	L	L	Res. Cor.	0.0 (0.0,0.4)	0.0 (0.0,0.4)	0.0 (0.0,0.4)	0.0 (0.0,0.4)	0.0 (0.0,0.4)	55.7 (52.3,59.2)
				Pairwise G^2	0.0 (0.0,0.4)	0.0 (0.0,0.4)	0.0 (0.0,0.4)	0.0 (0.0,0.4)	0.0 (0.0,0.4)	4.3 (3.0,5.9)
				Pairwise χ^2	0.0 (0.0,0.4)	0.0 (0.0,0.4)	0.0 (0.0,0.4)	0.0 (0.0,0.4)	0.0 (0.0,0.4)	4.4 (3.1,6.0)
H	H	H	L	Res. Cor.	0.0 (0.0,0.4)	0.0 (0.0,0.4)	0.0 (0.0,0.4)	0.0 (0.0,0.4)	0.0 (0.0,0.4)	40.8 (37.7,43.9)
				Pairwise G^2	0.0 (0.0,0.4)	0.0 (0.0,0.4)	0.0 (0.0,0.4)	0.0 (0.0,0.4)	0.0 (0.0,0.4)	9.6 (7.8,11.6)
				Pairwise χ^2	0.0 (0.0,0.4)	0.0 (0.0,0.4)	0.0 (0.0,0.4)	0.0 (0.0,0.4)	0.0 (0.0,0.4)	9.5 (7.8,11.5)
H	H	H	H	Res. Cor.	0.0 (0.0,0.4)	0.0 (0.0,0.4)	0.0 (0.0,0.4)	0.0 (0.0,0.4)	0.0 (0.0,0.4)	86.9 (84.6,88.9)
				Pairwise G^2	0.0 (0.0,0.4)	0.0 (0.0,0.4)	0.0 (0.0,0.4)	0.0 (0.0,0.4)	0.0 (0.0,0.4)	59.3 (56.2,62.4)
				Pairwise χ^2	0.0 (0.0,0.4)	0.0 (0.0,0.4)	0.0 (0.0,0.4)	0.0 (0.0,0.4)	0.0 (0.0,0.4)	59.2 (56.1,62.3)
L	L	L	L	Res. Cor.	0.0 (0.0,0.6)	0.3 (0.0,1.1)	0.3 (0.0,1.1)	0.5 (0.1,1.3)	0.8 (0.2,1.8)	57.5 (53.7,61.3)
				Pairwise G^2	0.0 (0.0,0.6)	0.0 (0.0,0.6)	0.0 (0.0,0.6)	0.2 (0.0,0.8)	0.0 (0.0,0.6)	8.8 (6.8,11.3)
				Pairwise χ^2	0.0 (0.0,0.6)	0.0 (0.0,0.6)	0.0 (0.0,0.6)	0.2 (0.0,0.8)	0.0 (0.0,0.6)	8.8 (6.8,11.3)
L	L	H	L	Res. Cor.	0.0 (0.0,0.4)	0.0 (0.0,0.4)	3.6 (2.5,4.9)	0.0 (0.0,0.4)	2.7 (1.7,3.9)	71.7 (68.7,74.5)
				Pairwise G^2	0.0 (0.0,0.4)	0.0 (0.0,0.4)	0.4 (0.1,1.0)	0.0 (0.0,0.4)	0.3 (0.1,0.9)	34.4 (31.4,37.4)
				Pairwise χ^2	0.0 (0.0,0.4)	0.0 (0.0,0.4)	0.4 (0.1,1.0)	0.0 (0.0,0.4)	0.3 (0.1,0.9)	34.4 (31.4,37.4)

				**Res. Cor.**	0.5 (0.2,1.2)	5.8 (4.4,7.5)	4.5 (3.3,6.0)	6.3 (4.9,8.0)	5.1 (3.8,6.7)	99.3 (98.6,99.7)
**L**	**L**	**H**	**H**	**Pairwise G^2**	0.2 (0.0,0.7)	1.6 (0.9,2.6)	1.5 (0.8,2.5)	2.3 (1.5,3.4)	1.5 (0.8,2.5)	96.3 (94.9,97.4)
				**Pairwise χ^2**	0.2 (0.0,0.7)	1.9 (1.2,3.0)	1.5 (0.8,2.5)	2.6 (1.7,3.8)	1.7 (1.0,2.7)	96.3 (94.9,97.4)
				**Res. Cor.**	0.0 (0.0,0.4)	9.4 (7.7,11.4)	0.1 (0.0,0.6)	0.0 (0.0,0.4)	0.0 (0.0,0.4)	56.9 (53.8,60.0)
**L**	**H**	**L**	**H**	**Pairwise G^2**	0.0 (0.0,0.4)	1.4 (0.8,2.3)	0.0 (0.0,0.4)	0.0 (0.0,0.4)	0.0 (0.0,0.4)	20.3 (17.9,23.0)
				**Pairwise χ^2**	0.0 (0.0,0.4)	1.4 (0.8,2.3)	0.0 (0.0,0.4)	0.0 (0.0,0.4)	0.0 (0.0,0.4)	20.3 (17.9,23.0)
**Sensitivity: H H H L**										
**Specificity**				**Tool**	**T_1&T_2**	**T_1&T_3**	**T_1&T_4**	**T_2&T_3**	**T_2&T_4**	**T_3&T_4**
				**Res. Cor.**	0.0 (0.0,0.4)	0.0 (0.0,0.4)	0.0 (0.0,0.4)	0.0 (0.0,0.4)	0.0 (0.0,0.4)	43.2 (40.1,46.4)
**H**	**H**	**L**	**L**	**Pairwise G^2**	0.0 (0.0,0.4)	0.0 (0.0,0.4)	0.0 (0.0,0.4)	0.0 (0.0,0.4)	0.0 (0.0,0.4)	10.1 (8.3,12.1)
				**Pairwise χ^2**	0.0 (0.0,0.4)	0.0 (0.0,0.4)	0.0 (0.0,0.4)	0.0 (0.0,0.4)	0.0 (0.0,0.4)	10.1 (8.3,12.1)
				**Res. Cor.**	0.0 (0.0,0.4)	0.0 (0.0,0.4)	0.0 (0.0,0.4)	0.0 (0.0,0.4)	0.0 (0.0,0.4)	34.1 (31.2,37.1)
**H**	**H**	**H**	**L**	**Pairwise G^2**	0.0 (0.0,0.4)	0.0 (0.0,0.4)	0.0 (0.0,0.4)	0.0 (0.0,0.4)	0.0 (0.0,0.4)	13.3 (11.3,15.6)
				**Pairwise χ^2**	0.0 (0.0,0.4)	0.0 (0.0,0.4)	0.0 (0.0,0.4)	0.0 (0.0,0.4)	0.0 (0.0,0.4)	13.3 (11.3,15.6)
				**Res. Cor.**	0.0 (0.0,0.4)	0.0 (0.0,0.4)	0.0 (0.0,0.4)	0.0 (0.0,0.4)	0.0 (0.0,0.4)	99.6 (99.0,99.9)
**H**	**H**	**H**	**H**	**Pairwise G^2**	0.0 (0.0,0.4)	0.0 (0.0,0.4)	0.0 (0.0,0.4)	0.0 (0.0,0.4)	0.0 (0.0,0.4)	99.0 (98.2,99.5)
				**Pairwise χ^2**	0.0 (0.0,0.4)	0.0 (0.0,0.4)	0.0 (0.0,0.4)	0.0 (0.0,0.4)	0.0 (0.0,0.4)	99.0 (98.2,99.5)
				**Res. Cor.**	0.0 (0.0,0.4)	0.2 (0.0,0.8)	3.2 (2.1,4.5)	0.4 (0.1,1.1)	4.0 (2.8,5.5)	69.9 (66.8,72.8)
**L**	**L**	**L**	**L**	**Pairwise G^2**	0.0 (0.0,0.4)	0.0 (0.0,0.4)	0.4 (0.1,1.1)	0.0 (0.0,0.4)	0.4 (0.1,1.1)	32.6 (29.6,35.6)
				**Pairwise χ^2**	0.0 (0.0,0.4)	0.0 (0.0,0.4)	0.4 (0.1,1.1)	0.0 (0.0,0.4)	0.4 (0.1,1.1)	32.6 (29.6,35.6)
				**Res. Cor.**	0.0 (0.0,0.4)	0.0 (0.0,0.4)	9.9 (8.1,11.9)	0.0 (0.0,0.4)	10.0 (8.2,12.0)	86.4 (84.1,88.4)
**L**	**L**	**H**	**L**	**Pairwise G^2**	0.0 (0.0,0.4)	0.0 (0.0,0.4)	1.9 (1.2,3.0)	0.0 (0.0,0.4)	1.9 (1.2,3.0)	71.4 (68.5,74.2)
				**Pairwise χ^2**	0.0 (0.0,0.4)	0.0 (0.0,0.4)	2.0 (1.2,3.1)	0.0 (0.0,0.4)	2.0 (1.2,3.1)	71.4 (68.5,74.2)
				**Res. Cor.**	57.3 (54.2,60.4)	14.4 (12.3,16.7)	48.3 (45.2,51.4)	14.0 (11.9,16.3)	48.7 (45.6,51.8)	100.0 (99.6,100.0)
**L**	**L**	**H**	**H**	**Pairwise G^2**	34.8 (31.8,37.8)	7.6 (6.0,9.4)	26.6 (23.9,29.5)	6.8 (5.3,8.5)	25.2 (22.5,28.0)	100.0 (99.6,100.0)
				**Pairwise χ^2**	34.6 (31.7,37.6)	8.0 (6.4,9.9)	28.2 (25.4,31.1)	7.4 (5.9,9.2)	26.4 (23.7,29.2)	100.0 (99.6,100.0)
				**Res. Cor.**	0.0 (0.0,0.4)	19.9 (17.5,22.5)	10.5 (8.7,12.6)	0.0 (0.0,0.4)	0.0 (0.0,0.4)	98.3 (97.3,99.0)
**L**	**H**	**L**	**H**	**Pairwise G^2**	0.0 (0.0,0.4)	4.8 (3.6,6.3)	3.4 (2.4,4.7)	0.0 (0.0,0.4)	0.0 (0.0,0.4)	93.5 (91.8,94.9)
				**Pairwise χ^2**	0.0 (0.0,0.4)	4.8 (3.6,6.3)	3.5 (2.4,4.8)	0.0 (0.0,0.4)	0.0 (0.0,0.4)	93.3 (91.6,94.8)
**Sensitivity: H H H H**										
**Specificity**				**Tool**	**T_1&T_2**	**T_1&T_3**	**T_1&T_4**	**T_2&T_3**	**T_2&T_4**	**T_3&T_4**
				**Res. Cor.**	0.0 (0.0,0.4)	0.0 (0.0,0.4)	0.0 (0.0,0.4)	0.0 (0.0,0.4)	0.0 (0.0,0.4)	89.2 (87.1,91.1)
**H**	**H**	**L**	**L**	**Pairwise G^2**	0.0 (0.0,0.4)	0.0 (0.0,0.4)	0.0 (0.0,0.4)	0.0 (0.0,0.4)	0.0 (0.0,0.4)	65.0 (62.0,68.0)
				**Pairwise χ^2**	0.0 (0.0,0.4)	0.0 (0.0,0.4)	0.0 (0.0,0.4)	0.0 (0.0,0.4)	0.0 (0.0,0.4)	65.0 (62.0,68.0)
				**Res. Cor.**	0.0 (0.0,0.4)	0.0 (0.0,0.4)	0.2 (0.0,0.7)	0.0 (0.0,0.4)	0.1 (0.0,0.6)	99.9 (99.4,100.0)
**H**	**H**	**H**	**L**	**Pairwise G^2**	0.0 (0.0,0.4)	0.0 (0.0,0.4)	0.0 (0.0,0.4)	0.0 (0.0,0.4)	0.0 (0.0,0.4)	99.9 (99.4,100.0)
				**Pairwise χ^2**	0.0 (0.0,0.4)	0.0 (0.0,0.4)	0.0 (0.0,0.4)	0.0 (0.0,0.4)	0.0 (0.0,0.4)	99.9 (99.4,100.0)
				**Res. Cor.**	0.0 (0.0,0.4)	0.0 (0.0,0.4)	0.0 (0.0,0.4)	0.0 (0.0,0.4)	0.0 (0.0,0.4)	100.0 (99.6,100.0)
**H**	**H**	**H**	**H**	**Pairwise G^2**	0.0 (0.0,0.4)	0.0 (0.0,0.4)	0.0 (0.0,0.4)	0.0 (0.0,0.4)	0.0 (0.0,0.4)	100.0 (99.6,100.0)
				**Pairwise χ^2**	0.0 (0.0,0.4)	0.0 (0.0,0.4)	0.0 (0.0,0.4)	0.0 (0.0,0.4)	0.0 (0.0,0.4)	100.0 (99.6,100.0)
**L**	**L**	**L**	**L**	**Res. Cor.**	3.4 (2.4,4.8)	7.4 (5.8,9.2)	9.4 (7.7,11.4)	7.1 (5.6,8.9)	7.3 (5.7,9.1)	99.9 (99.4,100.0)

				**Pairwise G^2**	1.2 (0.6,2.1)	1.4 (0.8,2.4)	1.8 (1.1,2.9)	1.7 (1.0,2.7)	1.8 (1.1,2.9)	98.2 (97.1,98.9)
				**Pairwise χ^2**	1.1 (0.6,2.0)	1.5 (0.9,2.5)	1.8 (1.1,2.9)	1.7 (1.0,2.7)	1.8 (1.1,2.9)	98.2 (97.1,98.9)
**L**	**L**	**H**	**L**	**Res. Cor.**	38.4 (35.4,41.5)	6.8 (5.3,8.5)	34.0 (31.1,37.0)	6.0 (4.6,7.7)	34.6 (31.7,37.6)	100.0 (99.6,100.0)
				**Pairwise G^2**	18.2 (15.9,20.7)	3.1 (2.1,4.4)	11.2 (9.3,13.3)	2.6 (1.7,3.8)	12.5 (10.5,14.7)	100.0 (99.6,100.0)
				**Pairwise χ^2**	18.1 (15.8,20.6)	3.4 (2.4,4.7)	11.2 (9.3,13.3)	2.9 (2.0,4.1)	12.6 (10.6,14.8)	100.0 (99.6,100.0)
**L**	**L**	**H**	**H**	**Res. Cor.**	98.9 (98.0,99.4)	36.7 (33.7,39.8)	35.3 (32.3,38.4)	37.0 (34.0,40.1)	35.7 (32.7,38.8)	100.0 (99.6,100.0)
				**Pairwise G^2**	93.1 (91.3,94.6)	22.2 (19.7,24.9)	23.1 (20.5,25.8)	23.5 (20.9,26.3)	21.9 (19.4,24.6)	100.0 (99.6,100.0)
				**Pairwise χ^2**	93.1 (91.3,94.6)	23.7 (21.1,26.5)	24.4 (21.8,27.2)	24.6 (22.0,27.4)	22.8 (20.2,25.5)	100.0 (99.6,100.0)
**L**	**H**	**L**	**H**	**Res. Cor.**	0.0 (0.0,0.4)	39.3 (36.3,42.4)	30.8 (27.9,33.8)	0.1 (0.0,0.6)	0.0 (0.0,0.4)	100.0 (99.6,100.0)
				**Pairwise G^2**	0.0 (0.0,0.4)	10.4 (8.6,12.5)	17.5 (15.2,20.0)	0.0 (0.0,0.4)	0.0 (0.0,0.4)	99.9 (99.4,100.0)
				**Pairwise χ^2**	0.0 (0.0,0.4)	10.5 (8.7,12.6)	18.3 (15.9,20.8)	0.0 (0.0,0.4)	0.0 (0.0,0.4)	99.9 (99.4,100.0)
**Sensitivity: L L L L**										
**Specificity**				**Tool**	**T_1&T_2**	**T_1&T_3**	**T_1&T_4**	**T_2&T_3**	**T_2&T_4**	**T_3&T_4**
**H**	**H**	**L**	**L**	**Res. Cor.**	0.0 (0.0,0.8)	0.0 (0.0,0.8)	0.0 (0.0,0.8)	0.0 (0.0,0.8)	0.0 (0.0,0.8)	84.0 (80.4,87.1)
				**Pairwise G^2**	0.0 (0.0,0.8)	0.0 (0.0,0.8)	0.0 (0.0,0.8)	0.0 (0.0,0.8)	0.0 (0.0,0.8)	30.6 (26.5,34.9)
				**Pairwise χ^2**	0.0 (0.0,0.8)	0.0 (0.0,0.8)	0.0 (0.0,0.8)	0.0 (0.0,0.8)	0.0 (0.0,0.8)	30.6 (26.5,34.9)
**H**	**H**	**H**	**L**	**Res. Cor.**	0.0 (0.0,0.4)	0.0 (0.0,0.4)	0.2 (0.0,0.7)	0.0 (0.0,0.4)	0.2 (0.0,0.7)	99.9 (99.4,100.0)
				**Pairwise G^2**	0.0 (0.0,0.4)	0.0 (0.0,0.4)	0.2 (0.0,0.7)	0.0 (0.0,0.4)	0.1 (0.0,0.6)	94.7 (93.1,96.0)
				**Pairwise χ^2**	0.0 (0.0,0.4)	0.0 (0.0,0.4)	0.2 (0.0,0.7)	0.0 (0.0,0.4)	0.1 (0.0,0.6)	94.7 (93.1,96.0)
**H**	**H**	**H**	**H**	**Res. Cor.**	0.1 (0.0,0.6)	2.1 (1.3,3.2)	1.8 (1.1,2.8)	1.8 (1.1,2.8)	1.7 (1.0,2.7)	100.0 (99.6,100.0)
				**Pairwise G^2**	0.7 (0.3,1.4)	0.5 (0.2,1.2)	0.3 (0.1,0.9)	0.4 (0.1,1.0)	0.2 (0.0,0.7)	100.0 (99.6,100.0)
				**Pairwise χ^2**	0.7 (0.3,1.4)	0.5 (0.2,1.2)	0.3 (0.1,0.9)	0.5 (0.2,1.2)	0.2 (0.0,0.7)	100.0 (99.6,100.0)
**L**	**L**	**L**	**L**	**Res. Cor.**	0.0 (0.0,1.0)	12.1 (9.0,15.8)	13.7 (10.4,17.6)	14.7 (11.3,18.7)	13.9 (10.6,17.8)	98.2 (96.2,99.3)
				**Pairwise G^2**	0.0 (0.0,1.0)	1.3 (0.4,3.0)	1.8 (0.7,3.8)	2.6 (1.3,4.8)	2.4 (1.1,4.4)	65.0 (60.0,69.8)
				**Pairwise χ^2**	0.0 (0.0,1.0)	1.3 (0.4,3.0)	1.8 (0.7,3.8)	2.6 (1.3,4.8)	2.4 (1.1,4.4)	65.0 (60.0,69.8)
**L**	**L**	**H**	**L**	**Res. Cor.**	0.0 (0.0,0.4)	51.6 (48.3,54.8)	25.4 (22.6,28.3)	51.3 (48.0,54.5)	26.0 (23.2,28.9)	100.0 (99.6,100.0)
				**Pairwise G^2**	0.0 (0.0,0.4)	26.1 (23.3,29.1)	7.0 (5.5,8.9)	23.1 (20.5,26.0)	8.6 (6.9,10.6)	100.0 (99.6,100.0)
				**Pairwise χ^2**	0.0 (0.0,0.4)	26.4 (23.6,29.4)	7.1 (5.6,9.0)	23.2 (20.6,26.1)	8.7 (7.0,10.7)	100.0 (99.6,100.0)
**L**	**L**	**H**	**H**	**Res. Cor.**	29.0 (26.1,32.1)	98.2 (97.2,99.0)	99.2 (98.4,99.7)	98.1 (97.0,98.9)	98.4 (97.3,99.1)	99.8 (99.2,100.0)
				**Pairwise G^2**	36.7 (33.6,39.9)	92.5 (90.6,94.2)	95.3 (93.7,96.6)	93.6 (91.8,95.1)	92.7 (90.9,94.3)	99.8 (99.2,100.0)
				**Pairwise χ^2**	36.7 (33.6,39.9)	92.9 (91.0,94.4)	95.6 (94.1,96.8)	93.6 (91.8,95.1)	92.7 (90.9,94.3)	99.8 (99.2,100.0)
**L**	**H**	**L**	**H**	**Res. Cor.**	0.0 (0.0,0.4)	48.8 (45.5,52.1)	94.5 (92.9,95.9)	0.0 (0.0,0.4)	0.0 (0.0,0.4)	100.0 (99.6,100.0)
				**Pairwise G^2**	0.0 (0.0,0.4)	13.3 (11.2,15.7)	74.3 (71.4,77.1)	0.0 (0.0,0.4)	0.0 (0.0,0.4)	99.7 (99.0,99.9)
				**Pairwise χ^2**	0.0 (0.0,0.4)	13.5 (11.4,15.9)	74.8 (71.8,77.6)	0.0 (0.0,0.4)	0.0 (0.0,0.4)	99.7 (99.0,99.9)
**Sensitivity: L L H L**										
**Specificity**				**Tool**	**T_1&T_2**	**T_1&T_3**	**T_1&T_4**	**T_2&T_3**	**T_2&T_4**	**T_3&T_4**
**H**	**H**	**L**	**L**	**Res. Cor.**	0.0 (0.0,0.4)	0.0 (0.0,0.4)	0.3 (0.1,0.9)	0.0 (0.0,0.4)	0.8 (0.4,1.6)	99.5 (98.8,99.8)
				**Pairwise G^2**	0.0 (0.0,0.4)	0.0 (0.0,0.4)	0.0 (0.0,0.4)	0.0 (0.0,0.4)	0.2 (0.0,0.7)	94.9 (93.3,96.2)
				**Pairwise χ^2**	0.0 (0.0,0.4)	0.0 (0.0,0.4)	0.0 (0.0,0.4)	0.0 (0.0,0.4)	0.2 (0.0,0.7)	94.9 (93.3,96.2)
**H**	**H**	**H**	**L**	**Res. Cor.**	0.0 (0.0,0.4)	0.0 (0.0,0.4)	5.6 (4.3,7.2)	0.0 (0.0,0.4)	5.5 (4.2,7.1)	100.0 (99.6,100.0)
				**Pairwise G^2**	0.0 (0.0,0.4)	0.0 (0.0,0.4)	1.6 (0.9,2.6)	0.0 (0.0,0.4)	1.6 (0.9,2.6)	100.0 (99.6,100.0)

				**Pairwise χ^2**	0.0 (0.0,0.4)	0.0 (0.0,0.4)	1.6 (0.9,2.6)	0.0 (0.0,0.4)	1.6 (0.9,2.6)	100.0 (99.6,100.0)
**H**	**H**	**H**	**H**	**Res. Cor.**	100.0 (99.6,100.0)	1.6 (0.9,2.6)	96.8 (95.5,97.8)	1.4 (0.8,2.4)	97.6 (96.5,98.5)	100.0 (99.6,100.0)
				**Pairwise G^2**	100.0 (99.6,100.0)	0.6 (0.2,1.3)	89.6 (87.5,91.4)	0.6 (0.2,1.3)	91.1 (89.1,92.8)	100.0 (99.6,100.0)
				**Pairwise χ^2**	100.0 (99.6,100.0)	0.6 (0.2,1.3)	89.8 (87.7,91.6)	0.6 (0.2,1.3)	91.3 (89.4,93.0)	100.0 (99.6,100.0)
**L**	**L**	**L**	**L**	**Res. Cor.**	0.0 (0.0,0.4)	63.0 (59.8,66.1)	32.1 (29.1,35.2)	66.8 (63.7,69.9)	33.9 (30.9,37.1)	100.0 (99.6,100.0)
				**Pairwise G^2**	0.0 (0.0,0.4)	30.8 (27.8,33.9)	9.6 (7.8,11.7)	34.3 (31.3,37.5)	7.9 (6.2,9.8)	100.0 (99.6,100.0)
				**Pairwise χ^2**	0.0 (0.0,0.4)	31.0 (28.0,34.1)	9.8 (8.0,11.9)	34.7 (31.6,37.8)	7.9 (6.2,9.8)	100.0 (99.6,100.0)
**L**	**L**	**H**	**L**	**Res. Cor.**	0.0 (0.0,0.4)	93.2 (91.5,94.7)	57.3 (54.1,60.4)	93.2 (91.5,94.7)	52.6 (49.5,55.8)	100.0 (99.6,100.0)
				**Pairwise G^2**	0.0 (0.0,0.4)	78.7 (76.0,81.2)	26.2 (23.4,29.0)	74.2 (71.4,76.9)	23.1 (20.5,25.9)	100.0 (99.6,100.0)
				**Pairwise χ^2**	0.0 (0.0,0.4)	78.9 (76.2,81.4)	26.6 (23.8,29.4)	74.4 (71.6,77.1)	23.1 (20.5,25.9)	100.0 (99.6,100.0)
**L**	**L**	**H**	**H**	**Res. Cor.**	100.0 (99.6,100.0)	32.2 (29.0,35.4)	99.4 (98.6,99.8)	30.6 (27.5,33.8)	98.8 (97.8,99.4)	2.0 (1.2,3.2)
				**Pairwise G^2**	100.0 (99.6,100.0)	21.9 (19.1,24.8)	96.9 (95.5,98.0)	20.2 (17.6,23.1)	96.7 (95.3,97.8)	4.3 (3.0,5.8)
				**Pairwise χ^2**	100.0 (99.6,100.0)	22.5 (19.7,25.4)	96.9 (95.5,98.0)	20.4 (17.8,23.3)	96.8 (95.4,97.9)	4.1 (2.9,5.7)
**L**	**H**	**L**	**H**	**Res. Cor.**	0.1 (0.0,0.6)	99.8 (99.2,100.0)	100.0 (99.6,100.0)	0.0 (0.0,0.4)	0.0 (0.0,0.4)	100.0 (99.6,100.0)
				**Pairwise G^2**	0.2 (0.0,0.8)	97.9 (96.8,98.7)	99.9 (99.4,100.0)	0.0 (0.0,0.4)	0.0 (0.0,0.4)	100.0 (99.6,100.0)
				**Pairwise χ^2**	0.2 (0.0,0.8)	97.9 (96.8,98.7)	99.9 (99.4,100.0)	0.0 (0.0,0.4)	0.0 (0.0,0.4)	100.0 (99.6,100.0)
**Sensitivity: L L H H**										
**Specificity**				**Tool**	**T_1&T_2**	**T_1&T_3**	**T_1&T_4**	**T_2&T_3**	**T_2&T_4**	**T_3&T_4**
**H**	**H**	**L**	**L**	**Res. Cor.**	0.0 (0.0,0.4)	0.8 (0.3,1.6)	0.7 (0.3,1.4)	0.6 (0.2,1.3)	0.7 (0.3,1.4)	100.0 (99.6,100.0)
				**Pairwise G^2**	0.0 (0.0,0.4)	0.3 (0.1,0.9)	0.1 (0.0,0.6)	0.1 (0.0,0.6)	0.0 (0.0,0.4)	100.0 (99.6,100.0)
				**Pairwise χ^2**	0.0 (0.0,0.4)	0.3 (0.1,0.9)	0.2 (0.0,0.7)	0.1 (0.0,0.6)	0.0 (0.0,0.4)	100.0 (99.6,100.0)
**H**	**H**	**H**	**L**	**Res. Cor.**	78.5 (75.8,81.0)	0.6 (0.2,1.3)	57.2 (54.1,60.3)	0.4 (0.1,1.0)	56.4 (53.3,59.5)	100.0 (99.6,100.0)
				**Pairwise G^2**	0.0 (0.0,0.4)	0.0 (0.0,0.4)	1.6 (0.9,2.6)	0.0 (0.0,0.4)	1.6 (0.9,2.6)	100.0 (99.6,100.0)
				**Pairwise χ^2**	0.0 (0.0,0.4)	0.0 (0.0,0.4)	1.6 (0.9,2.6)	0.0 (0.0,0.4)	1.6 (0.9,2.6)	100.0 (99.6,100.0)
**H**	**H**	**H**	**H**	**Res. Cor.**	100.0 (99.6,100.0)	30.2 (27.4,33.2)	29.3 (26.5,32.2)	31 (28.1,34.0)	28.2 (25.4,31.1)	69.7 (66.7,72.5)
				**Pairwise G^2**	100.0 (99.6,100.0)	18.5 (16.1,21.0)	17.6 (15.3,20.1)	17.5 (15.2,20.0)	18.3 (15.9,20.8)	81.7 (79.2,84.1)
				**Pairwise χ^2**	100.0 (99.6,100.0)	20.2 (17.8,22.8)	19.2 (16.8,21.8)	19.2 (16.8,21.8)	18.9 (16.5,21.5)	80.4 (77.8,82.8)
**L**	**L**	**L**	**L**	**Res. Cor.**	88.9 (86.7,90.9)	99.5 (98.7,99.8)	99.0 (98.1,99.5)	99.6 (98.9,99.9)	99.7 (99.0,99.9)	100.0 (99.6,100.0)
				**Pairwise G^2**	84.9 (82.4,87.2)	95.6 (94.1,96.9)	95.2 (93.6,96.5)	96.1 (94.6,97.2)	96.3 (94.8,97.4)	100.0 (99.6,100.0)
				**Pairwise χ^2**	84.8 (82.3,87.1)	95.7 (94.2,96.9)	95.4 (93.8,96.7)	96.1 (94.6,97.2)	96.3 (94.8,97.4)	100.0 (99.6,100.0)
**L**	**L**	**H**	**L**	**Res. Cor.**	100.0 (99.5,100.0)	9.1 (7.1,11.5)	67.8 (64.2,71.3)	9.0 (7.0,11.4)	64.8 (61.1,68.3)	19.1 (16.3,22.3)
				**Pairwise G^2**	100.0 (99.5,100.0)	6.2 (4.5,8.3)	46.1 (42.3,49.9)	7.5 (5.7,9.8)	40.6 (36.9,44.3)	38.7 (35.0,42.4)
				**Pairwise χ^2**	100.0 (99.5,100.0)	6.2 (4.5,8.3)	46.8 (43.0,50.6)	7.5 (5.7,9.8)	41.2 (37.5,44.9)	38.0 (34.3,41.7)
**L**	**L**	**H**	**H**	**Res. Cor.**	100.0 (99.6,100.0)	0.0 (0.0,0.4)	0.0 (0.0,0.4)	0.1 (0.0,0.6)	0.0 (0.0,0.4)	0.0 (0.0,0.4)
				**Pairwise G^2**	100.0 (99.6,100.0)	0.0 (0.0,0.4)	0.0 (0.0,0.4)	0.0 (0.0,0.4)	0.0 (0.0,0.4)	0.0 (0.0,0.4)
				**Pairwise χ^2**	100.0 (99.6,100.0)	0.0 (0.0,0.4)	0.0 (0.0,0.4)	0.0 (0.0,0.4)	0.0 (0.0,0.4)	0.0 (0.0,0.4)
**L**	**H**	**L**	**H**	**Res. Cor.**	94.6 (92.9,96.0)	100.0 (99.6,100.0)	100.0 (99.6,100.0)	47.3 (43.9,50.7)	0.0 (0.0,0.4)	100.0 (99.6,100.0)
				**Pairwise G^2**	96.7 (95.3,97.8)	99.9 (99.4,100.0)	100.0 (99.6,100.0)	31.3 (28.2,34.5)	0.0 (0.0,0.4)	100.0 (99.6,100.0)
				**Pairwise χ^2**	96.6 (95.2,97.7)	100.0 (99.6,100.0)	100.0 (99.6,100.0)	33.4 (30.3,36.7)	0.0 (0.0,0.4)	100.0 (99.6,100.0)

Table ii.4 Mean absolute biases (x10^{-2}) of posterior medians of each parameter obtained from the conditional independence model

Specificity				π	Se_1	Se_2	Se_3	Se_4	Sp_1	Sp_2	Sp_3	Sp_4
**Sensitivity: H H L L**												
**H**	**H**	**L**	**L**	-3.2 (-3.3,-3.1)	5.5 (5.4,5.7)	5.6 (5.5,5.7)	0.0 (-0.1,0.1)	0.0 (-0.1,0.1)	0.0 (-0.1,0.1)	0.0 (-0.1,0.1)	-1.2 (-1.3,-1.1)	-1.3 (-1.3,-1.2)
**H**	**H**	**H**	**L**	-3.2 (-3.3.-3.2)	5.5 (5.5,5.6)	5.5 (5.4,5.5)	-0.1 (-0.1,0.0)	0.1 (0.0,0.2)	0.1 (0.0,0.1)	0.0 (0.0,0.1)	-3.0 (-3.1,-3.0)	-1.2 (-1.2,-1.1)
**H**	**H**	**H**	**H**	-3.1 (-3.1,-3.0)	5.4 (5.4,5.4)	5.4 (5.3,5.4)	0.0 (0.0,0.1)	0.1 (0.0,0.1)	0.1 (0.0,0.1)	0.1 (0.0,0.1)	-2.9 (-2.9,-2.9)	-2.9 (-2.9,-2.9)
**L**	**L**	**L**	**L**	-4.5 (-4.6,-4.3)	7.0 (6.9,7.1)	6.9 (6.8,7.0)	-0.4 (-0.5,-0.3)	-0.4 (-0.5,-0.3)	1.6 (1.5,1.7)	1.6 (1.5,1.7)	-1.9 (-2.0,-1.8)	-2.0 (-2.0,-1.9)
**L**	**L**	**H**	**L**	-6.1 (-6.2,-6.0)	7.9 (7.8,7.9)	7.8 (7.8,7.9)	-0.3 (-0.4,-0.2)	0.1 (0.1,0.2)	0.8 (0.7,0.8)	0.7 (0.6,0.8)	-5.7 (-5.8,-5.7)	-2.2 (-2.2,-2.1)
**L**	**L**	**H**	**H**	-4.7 (-4.8,-4.6)	6.5 (6.5,6.6)	6.5 (6.5,6.6)	0.0 (-0.1,0.1)	0.0 (0.0,0.1)	1.1 (1.0,1.1)	1.1 (1.0,1.2)	-4.3 (-4.4,-4.3)	-4.3 (-4.4,-4.3)
**L**	**H**	**L**	**H**	-4.4 (-4.5,-4.3)	5.5 (5.4,5.5)	7.9 (7.8,8.0)	0.1 (0.0,0.2)	-0.1 (-0.2,0.0)	0.6 (0.5,0.7)	0.1 (0.0,0.1)	-1.6 (-1.6,-1.5)	-4.2 (-4.2,-4.1)
**Sensitivity: H H H L**												
**Specificity**				π	Se_1	Se_2	Se_3	Se_4	Sp_1	Sp_2	Sp_3	Sp_4
**H**	**H**	**L**	**L**	-3.2 (-3.3,-3.1)	5.6 (5.5,5.6)	5.6 (5.5,5.6)	0.0 (-0.1,0.0)	0.0 (0.0,0.1)	0.0 (-0.1,0.1)	0.0 (-0.1,0.0)	-3.1 (-3.1,-3.0)	-1.2 (-1.3,-1.2)
**H**	**H**	**H**	**L**	-3.2 (-3.3.-3.2)	5.5 (5.5,5.6)	5.6 (5.5,5.6)	0.0 (0.0,0.0)	0.1 (0.1,0.2)	0.0 (-0.1,0.0)	0.0 (0.0,0.0)	-4.9 (-5.0,-4.9)	-1.1 (-1.2,-1.1)
**H**	**H**	**H**	**H**	-3.0 (-3.1,-3.0)	5.3 (5.2,5.3)	5.3 (5.3,5.3)	0.0 (0.0,0.1)	0.3 (0.2,0.4)	0.1 (0.1,0.1)	0.1 (0.1,0.1)	-4.5 (-4.6,-4.5)	-2.6 (-2.6,-2.6)
**L**	**L**	**L**	**L**	-5.8 (-5.9,-5.7)	7.7 (7.6.7.7)	7.6 (7.6.7.7)	-0.3 (-0.4,-0.3)	0.0 (-0.1,0.1)	0.9 (0.8,0.9)	0.9 (0.8,0.9)	-5.5 (-5.6,-5.4)	-2.0 (-2.0,-1.9)
**L**	**L**	**H**	**L**	-6.5 (-6.5,-6.4)	8.0 (8.0,8.1)	8.0 (8.0,8.0)	-0.2 (-0.3,-0.1)	0.4 (0.4,0.5)	0.6 (0.5,0.6)	0.4 (0.4,0.5)	-9.4 (-9.4,-9.3)	-2.0 (-2.0,-1.9)
**L**	**L**	**H**	**H**	-4.5 (-4.6,-4.5)	5.9 (5.9,6.0)	5.9 (5.9,6.0)	0.4 (0.4,0.5)	1.1 (1.0,1.2)	0.8 (0.7,0.8)	0.8 (0.7,0.8)	-6.4 (-6.4,-6.3)	-3.3 (-3.4,-3.3)
**L**	**H**	**L**	**H**	-3.7 (-3.8,-3.7)	5.3 (5.3,5.4)	6.7 (6.7,6.8)	0.0 (0.0,0.1)	-0.1 (-0.1,0.0)	1.1 (1.1,1.2)	0.2 (0.1,0.2)	-3.5 (-3.5,-3.4)	-3.5 (-3.6,-3.5)
**Sensitivity: H H H H**												
**Specificity**				π	Se_1	Se_2	Se_3	Se_4	Sp_1	Sp_2	Sp_3	Sp_4
**H**	**H**	**L**	**L**	-3.0 (-3.1,-3.0)	5.4 (5.3,5.4)	5.3 (5.3,5.4)	0.0 (-0.1,0.0)	0.0 (-0.1,0.0)	0.2 (0.1,0.2)	0.1 (0.1,0.2)	-2.8 (-2.9,-2.7)	-2.9 (-3.0,-2.8)
**H**	**H**	**H**	**L**	-3.0 (-3.1,-3.0)	5.3 (5.3,5.3)	5.3 (5.3,5.3)	0.0 (0.0,0.0)	0.1 (0.1,0.2)	0.2 (0.1,0.2)	0.1 (0.1,0.2)	-4.6 (-4.6,-4.5)	-2.7 (-2.8,-2.6)
**H**	**H**	**H**	**H**	-2.7 (-2.7,-2.6)	4.8 (4.8,4.8)	4.8 (4.8,4.8)	0.1 (0.1,0.2)	0.1 (0.1,0.1)	0.2 (0.2,0.2)	0.2 (0.2,0.3)	-4.0 (-4.0,-3.9)	-4.0 (-4.0,-4.0)
**L**	**L**	**L**	**L**	-5.3 (-5.3,-5.2)	6.9 (6.8,6.9)	6.9 (6.9,7.0)	0.2 (0.1,0.2)	0.2 (0.1,0.3)	0.8 (0.7,0.9)	0.8 (0.8,0.9)	-4.6 (-4.7,-4.5)	-4.6 (-4.7,-4.5)
**L**	**L**	**H**	**L**	-4.9 (-4.9,-4.8)	6.3 (6.2,6.3)	6.3 (6.2,6.3)	0.4 (0.3,0.4)	0.8 (0.8,0.9)	0.7 (0.6,0.8)	0.8 (0.7,0.8)	-6.8 (-6.9,-6.8)	-3.8 (-3.8,-3.7)
**L**	**L**	**H**	**H**	-3.2 (-3.3,-3.2)	4.2 (4.2,4.3)	4.3 (4.2,4.3)	0.8 (0.8,0.9)	0.8 (0.8,0.9)	0.7 (0.6,0.7)	0.7 (0.6,0.7)	-4.2 (-4.2,-4.1)	-4.2 (-4.3,-4.2)
**L**	**H**	**L**	**H**	-3.7 (-3.8,-3.7)	5.3 (5.2,5.3)	6.4 (6.4,6.5)	0.2 (0.2,0.3)	0.0 (0.0,0.1)	1.1 (1.0,1.1)	0.1 (0.1,0.1)	-3.3 (-3.3,-3.2)	-5.5 (-5.6,-5.4)
**Sensitivity: L L L L**												
**Specificity**				π	Se_1	Se_2	Se_3	Se_4	Sp_1	Sp_2	Sp_3	Sp_4
**H**	**H**	**L**	**L**	-16.2 (-16.4,-15.9)	24.2 (23.8,24.5)	23.9 (23.6,24.2)	0.0 (-0.2,0.1)	0.1 (0.0,0.2)	0.1 (-0.1,0.3)	0.0 (-0.1,0.2)	-4.9 (-5.0,-4.8)	-4.9 (-5.0,-4.8)
**H**	**H**	**H**	**L**	-16.0 (-16.1,-15.9)	23.7 (23.6,23.8)	23.7 (23.5,23.8)	0.0 (-0.1,0.1)	0.5 (0.4,0.6)	0.1 (0.0,0.2)	0.1 (0.0,0.1)	-12.1 (-12.2,-12.1)	-4.6 (-4.7,-4.6)
**H**	**H**	**H**	**H**	-14.8 (-14.9,-14.7)	21.8 (21.7,21.9)	21.8 (21.7,21.9)	0.9 (0.8,0.9)	0.9 (0.8,0.9)	0.4 (0.4,0.5)	0.4 (0.4,0.5)	-11.0 (-11.0,-10.9)	-11.0 (-11.1,-10.9)
**L**	**L**	**L**	**L**	-24.4 (-24.5,-24.2)	35.5 (35.2,35.7)	35.2 (35.0,35.5)	-1.7 (-1.9,-1.5)	-1.7 (-1.9,-1.5)	5.4 (5.2,5.5)	5.4 (5.3,5.5)	-7.2 (-7.3,-7.1)	-7.2 (-7.2,-7.1)
**L**	**L**	**H**	**L**	-26.7 (-26.8,-26.7)	38.0 (37.9,38.1)	38.0 (37.9,38.0	-4.0 (-4.1,-3.9)	-0.9 (-1.0,-0.8)	4.5 (4.4,4.6)	4.4 (4.4,4.5)	-18.6 (-18.7,-18.6)	-7.3 (-7.3,-7.2)

**L**	**L**	**H**	**H**	-25.9 (-26.0,-25.8)	34.9 (34.7,35.0)	34.9 (34.7,35.0)	-1.1 (-1.2,-0.9)	-1.0 (-1.2,-0.9)	4.2 (4.2,4.3)	4.2 (4.1,4.2)	-17.4 (-17.5,-17.3)	-17.4 (-17.5,-17.3)
**L**	**H**	**L**	**H**	-21.2 (-21.2,-21.1)	23.2 (23.1,23.3)	38.3 (38.3,38.4)	0.0 (-0.1,0.1)	-0.5 (-0.6,-0.3)	3.4 (3.4,3.5)	0.5 (0.4,0.6)	-6.0 (-6.0,-5.9)	-15.1 (-15.2,-15.1)
**Sensitivity: L L H L**												
**Specificity**				π	Se_1	Se_2	Se_3	Se_4	Sp_1	Sp_2	Sp_3	Sp_4
**H**	**H**	**L**	**L**	-16.1 (-16.1,-16.0)	23.8 (23.7,23.9)	23.8 (23.7,23.9)	0.0 (-0.1,0.1)	0.5 (0.4,0.5)	0.1 (0.0,0.1)	0.1 (0.0,0.1)	-12.1 (-12.2,-12.0)	-4.7 (-4.7,-4.6)
**H**	**H**	**H**	**L**	-15.9 (-15.9,-15.8)	23.5 (23.4,23.5)	23.5 (23.4,23.6)	0.0 (-0.1,0.0)	1.0 (1.0,1.1)	0.0 (0.0,0.1)	0.1 (0.0,0.1)	-19.3 (-19.3,-19.2)	-4.3 (-4.4,-4.3)
**H**	**H**	**H**	**H**	-12.3 (-12.4,-12.2)	17.6 (17.4,17.8)	17.6 (17.5,17.7)	0.4 (0.3,0.4)	2.7 (2.6,2.8)	0.7 (0.6,0.7)	0.7 (0.6,0.7)	-15.6 (-15.7,-15.5)	-8.3 (-8.4,-8.2)
**L**	**L**	**L**	**L**	-26.7 (-26.7,-26.6)	37.9 (37.8,37.9)	37.8 (37.8,37.9)	-4.0 (-4.1,-3.9)	-0.7 (-0.8,-0.5)	4.5 (4.4,4.5)	4.5 (4.4,4.5)	-18.6 (-18.6,-18.5)	-7.2 (-7.2,-7.1)
**L**	**L**	**H**	**L**	-27.7 (-27.8,-27.6)	38.7 (38.7,38.8)	38.8 (38.7,38.8)	-5.2 (-5.4,-5.1)	0.9 (0.7,1.0)	3.9 (3.8,4.0)	3.9 (3.8,4.0)	-30.0 (-30.1,-30.0)	-6.9 (-7.0,-6.8)
**L**	**L**	**H**	**H**	-6.8 (-7.0,-6.7)	4.7 (4.5,4.8)	4.7 (4.6,4.8)	1.2 (1.1,1.4)	5.9 (5.8,6.0)	1.1 (1.1,1.2)	1.2 (1.1,1.2)	-8.6 (-8.7,-8.5)	-1.5 (-1.6,-1.5)
**L**	**H**	**L**	**H**	-19.3 (-19.4,-19.2)	22.5 (22.4,22.6)	31.9 (31.7,32.1)	0.5 (0.4,0.5)	0.6 (0.5,0.7)	4.4 (4.4,4.5)	0.2 (0.2,0.3)	-13.7 (-13.8,-13.6)	-13.6 (-13.7,-13.5)
**Sensitivity: L L H H**												
**Specificity**				π	Se_1	Se_2	Se_3	Se_4	Sp_1	Sp_2	Sp_3	Sp_4
**H**	**H**	**L**	**L**	-14.8 (-14.9,-14.8)	22.1 (22.1,22.2)	22.2 (22.1,22.3)	0.3 (0.2,0.3)	0.3 (0.2,0.3)	0.6 (0.5,0.6)	0.6 (0.5,0.6)	-11.3 (-11.3,-11.2)	-11.4 (-11.4,-11.3)
**H**	**H**	**H**	**L**	-13.7 (-13.7,-13.6)	20.0 (19.9,20.1)	20.0 (19.9,20.0)	0.3 (0.2,0.3)	1.2 (1.2,1.3)	0.7 (0.6,0.7)	0.6 (0.6,0.7)	-17.0 (-17.1,-17.0)	-10.1 (-10.1,-10.0)
**H**	**H**	**H**	**H**	-2.1 (-2.2,-2.1)	2.9 (2.8,3.0)	2.9 (2.8,3.0)	0.9 (0.8,0.9)	0.9 (0.9,1.0)	0.6 (0.6,0.7)	0.6 (0.6,0.7)	-2.5 (-2.5,-2.4)	-2.5 (-2.5,-2.4)
**L**	**L**	**L**	**L**	-27.1 (-27.2,-27.0)	35.6 (35.5,35.8)	35.6 (35.5,35.8)	0.9 (0.8,1.0)	0.9 (0.8,1.0)	3.5 (3.4,3.5)	3.5 (3.4,3.6)	-17.3 (-17.4,-17.2)	-17.3 (-17.4,-17.2)
**L**	**L**	**H**	**L**	-4.5 (-4.8,-4.1)	4.0 (3.6,4.3)	3.9 (3.6,4.3)	-0.1 (-0.3,0.1)	3.0 (2.9,3.1)	1.2 (1.1,1.3)	1.3 (1.2,1.4)	-6.5 (-6.8,-6.2)	-1.5 (-1.8,-1.3)
**L**	**L**	**H**	**H**	-0.7 (-0.8,-0.6)	0.8 (0.8,0.9)	0.9 (0.8,1.0)	0.5 (0.4,0.6)	0.5 (0.4,0.6)	0.5 (0.4,0.5)	0.5 (0.5,0.6)	-0.7 (-0.8,-0.6)	-0.7 (-0.8,-0.6)
**L**	**H**	**L**	**H**	-16.3 (-16.5,-16.2)	19.5 (19.3,19.7)	24.9 (24.6,25.2)	1.8 (1.8,1.9)	0.7 (0.6,0.8)	4.8 (4.8,4.9)	0.1 (0.1,0.2)	-11.3 (-11.5,-11.2)	-19.3 (-19.5,-19.1)

Table ii.5 Coverages of the 95% CrIs for each model parameter

**Sensitivity: H H L L**												
**Specificity**				π	Se_1	Se_2	Se_3	Se_4	Sp_1	Sp_2	Sp_3	Sp_4
**H**	**H**	**L**	**L**	36.8 (33.5,40.1)	4.6 (3.2,6.1)	4.0 (2.7,5.4)	95.1 (93.6,96.6)	95.6 (94.2,97.0)	96.1 (94.8,97.4)	94.9 (93.4,96.4)	77.7 (74.8,80.5)	75.6 (72.7,78.5)
**H**	**H**	**H**	**L**	3.4 (2.3,4.5)	0.0 (0.0,0.0)	0.0 (0.0,0.0)	94.8 (93.4,96.2)	96.0 (94.8,97.2)	94.6 (93.2,96.0)	94.2 (92.8,95.6)	0.7 (0.2,1.2)	76.9 (74.3,79.5)
**H**	**H**	**H**	**H**	3.5 (2.4,4.6)	0.0 (0.0,0.0)	0.0 (0.0,0.0)	95.4 (94.1,96.7)	93.7 (92.2,95.2)	95.5 (94.2,96.8)	94.9 (93.5,96.3)	0.6 (0.1,1.1)	1.1 (0.5,1.7)

**L**	**L**	**L**	**L**	68.8 (65.3,72.3)	7.6 (5.6,9.6)	9.3 (7.1,11.5)	94.7 (93.0,96.4)	94.4 (92.6,96.1)	93.0 (91.0,94.9)	91.9 (89.9,94.0)	62.4 (58.7,66.1)	62.4 (58.7,66.1)
**L**	**L**	**H**	**L**	2.8 (1.7,3.8)	0.0 (0.0,0.0)	0.0 (0.0,0.0)	92.1 (90.4,93.8)	96.0 (94.8,97.2)	91.7 (90.0,93.4)	92.4 (90.8,94.1)	0.0 (0.0,0.0)	44.9 (41.8,48.0)
**L**	**L**	**H**	**H**	2.9 (1.9,4.0)	0.0 (0.0,0.0)	0.0 (0.0,0.0)	95.5 (94.2,96.8)	94.7 (93.3,96.1)	88.2 (86.2,90.2)	85.7 (83.5,87.8)	0.2 (0.0,0.5)	0.2 (0.0,0.5)
**L**	**H**	**L**	**H**	2.1 (1.2,3.0)	0.0 (0.0,0.0)	0.0 (0.0,0.0)	95.7 (94.4,97.0)	94.7 (93.3,96.1)	94.3 (92.8,95.7)	95.9 (94.7,97.1)	65.3 (62.4,68.3)	0.2 (0.0,0.5)
**Sensitivity: H H H L**												
**Specificity**				π	Se_1	Se_2	Se_3	Se_4	Sp_1	Sp_2	Sp_3	Sp_4
**H**	**H**	**L**	**L**	6.5 (5.0,8.0)	0.0 (0.0,0.0)	0.0 (0.0,0.0)	96.5 (95.4,97.6)	95.7 (94.4,97.0)	95.1 (93.8,96.4)	96.2 (95.0,97.4)	18.0 15.6,20.4)	78.4 (75.8,80.9)
**H**	**H**	**H**	**L**	1.0 (0.4,1.6)	0.0 (0.0,0.0)	0.0 (0.0,0.0)	94.3 (92.9,95.7)	95.2 (93.9,96.5)	95.1 (93.8,96.4)	95.6 (94.3,96.9)	0.0 (0.0,0.0)	79.6 (77.1,82.1)
**H**	**H**	**H**	**H**	2.8 (1.8,3.8)	0.0 (0.0,0.0)	0.0 (0.0,0.0)	94.7 (93.3,96.1)	94.9 (93.5,96.3)	94.4 (93.0,95.8)	94.8 (93.4,96.2)	0.0 (0.0,0.0)	2.6 (1.6,3.6)
**L**	**L**	**L**	**L**	9.1 (7.2,10.9)	0.0 (0.0,0.0)	0.0 (0.0,0.0)	93.8 (92.2,95.3)	95.0 (93.7,96.4)	92.7 (91.1,94.4)	92.9 (91.3,94.6)	1.9 (1.0,2.8)	54.9 (51.7,58.1)
**L**	**L**	**H**	**L**	0.0 (0.0,0.0)	0.0 (0.0,0.0)	0.0 (0.0,0.0)	93.8 (92.3,95.3)	93.3 (91.8,94.9)	93.4 (91.9,95.0)	93.8 (92.3,95.3)	0.0 (0.0,0.0)	51.5 (48.4,54.6)
**L**	**L**	**H**	**H**	0.7 (0.2,1.2)	0.0 (0.0,0.0)	0.0 (0.0,0.0)	93.2 (91.6,94.8)	83.3 (81.0,85.6)	90.2 (88.4,92.0)	90.1 (88.2,92.0)	0.0 (0.0,0.0)	0.6 (0.1,1.1)
**L**	**H**	**L**	**H**	3.5 (2.4,4.6)	0.0 (0.0,0.0)	0.0 (0.0,0.0)	95.2 (93.9,96.5)	95.1 (93.8,96.4)	81.6 (79.2,84.0)	94.2 (92.8,95.6)	9.5 (7.7,11.3)	0.4 (0.0,0.8)
**Sensitivity: H H H H**												
**Specificity**				π	Se_1	Se_2	Se_3	Se_4	Sp_1	Sp_2	Sp_3	Sp_4
**H**	**H**	**L**	**L**	4.3 (3.0,5.6)	0.0 (0.0,0.0)	0.0 (0.0,0.0)	94.9 (93.5,96.3)	95.4 (94.1,96.7)	95.0 (93.6,96.4)	93.2 (91.6,94.8)	21.1 (18.6,23.6)	18.8 (16.4,21.2)
**H**	**H**	**H**	**L**	2.0 (1.1,2.9)	0.0 (0.0,0.0)	0.0 (0.0,0.0)	95.5 (94.2,96.8)	95.5 (94.2,96.8)	94.0 (92.5,95.5)	95.4 (94.1,96.7)	0.0 (0.0,0.0)	23.2 (20.6,25.8)
**H**	**H**	**H**	**H**	5.1 (3.7,6.5)	0.0 (0.0,0.0)	0.0 (0.0,0.0)	94.3 (92.9,95.7)	94.1 (92.6,95.6)	94.3 (92.9,95.7)	93.3 (91.8,94.8)	0.0 (0.0,0.0)	0.0 (0.0,0.0)
**L**	**L**	**L**	**L**	3.1 (2.1,4.2)	0.0 (0.0,0.0)	0.0 (0.0,0.0)	94.0 (92.6,95.5)	93.4 (91.9,95.0)	91.8 (90.1,93.5)	90.9 (89.1,92.7)	3.6 (2.5,4.8)	3.7 (2.6,4.9)
**L**	**L**	**H**	**L**	0.3 (0.0,0.6)	0.0 (0.0,0.0)	0.0 (0.0,0.0)	91.6 (89.9,93.3)	80.2 (77.7,82.7)	90.6 (88.8,92.4)	88.5 (86.5,90.5)	0.0 (0.0,0.0)	7.4 (5.8,9.0)
**L**	**L**	**H**	**H**	7.3 (5.7,8.9)	0.6 (0.1,1.1)	0.1 (0.0,0.3)	81.8 (79.4,84.2)	83.7 (81.4,86.0)	90.8 (89.0,92.6)	90.7 (88.9,92.5)	1.4 (0.7,2.1)	1.3 (0.6,2.0)
**L**	**H**	**L**	**H**	0.7 (0.2,1.2)	0.0 (0.0,0.0)	0.0 (0.0,0.0)	94.5 (93.1,95.9)	94.3 (92.9,95.7)	81.7 (79.4,84.2)	94.9 (93.5,96.3)	9.8 (8.0,11.6)	0.0 (0.0,0.0)
**Sensitivity: L L L L**												
**Specificity**				π	Se_1	Se_2	Se_3	Se_4	Sp_1	Sp_2	Sp_3	Sp_4
**H**	**H**	**L**	**L**	0.0 (0.0,0.0)	0.0 (0.0,0.0)	0.0 (0.0,0.0)	94.9 (92.9,96.8)	93.4 (91.2,95.6)	93.8 (91.7,96.0)	94.7 (92.7,96.7)	0.0 (0.0,0.0)	0.4 (0.0,1.0)
**H**	**H**	**H**	**L**	0.0 (0.0,0.0)	0.0 (0.0,0.0)	0.0 (0.0,0.0)	94.3 (92.8,95.7)	93.6 (92.0,95.1)	95.4 (94.1,96.7)	95.9 (94.7,97.2)	0.0 (0.0,0.0)	0.0 (0.0,0.0)
**H**	**H**	**H**	**H**	0.0 (0.0,0.0)	0.0 (0.0,0.0)	0.0 (0.0,0.0)	91.2 (89.5,93.0)	90.6 (88.8,92.4)	91.6 (89.9,93.4)	91.9 (90.3,93.6)	0.0 (0.0,0.0)	0.0 (0.0,0.0)
**L**	**L**	**L**	**L**	0.0 (0.0,0.0)	0.0 (0.0,0.0)	0.0 (0.0,0.0)	87.4 (84.0,90.7)	88.2 (84.9,91.4)	0.8 (-0.1,1.7)	1.1 (0.0,2.1)	0.0 (0.0,0.0)	0.0 (0.0,0.0)
**L**	**L**	**H**	**L**	0.0 (0.0,0.0)	0.0 (0.0,0.0)	0.0 (0.0,0.0)	50.4 (47.2,53.6)	93.2 (91.6,94.8)	2.0 (1.1,2.9)	0.6 (0.1,1.1)	0.0 (0.0,0.0)	0.0 (0.0,0.0)
**L**	**L**	**H**	**H**	0.1 (0.0,0.3)	0.0 (0.0,0.0)	0.0 (0.0,0.0)	86.9 (84.7,89.1)	89.9 (87.9,91.8)	3.8 (2.6,5.1)	4.2 (2.9,5.5)	0.2 (0.0,0.5)	0.2 (0.0,0.5)
**L**	**H**	**L**	**H**	0.0 (0.0,0.0)	0.0 (0.0,0.0)	0.0 (0.0,0.0)	94.7 (93.2,96.1)	93.7 (92.1,95.2)	2.8 (1.8,3.9)	93.0 (91.4,94.7)	0.0 (0.0,0.0)	0.0 (0.0,0.0)
**Sensitivity: L L H L**												
**Specificity**				π	Se_1	Se_2	Se_3	Se_4	Sp_1	Sp_2	Sp_3	Sp_4
**H**	**H**	**L**	**L**	0.0 (0.0,0.0)	0.0 (0.0,0.0)	0.0 (0.0,0.0)	94.7 (93.3,96.1)	93.5 (91.9,95.0)	95.4 (94.1,96.7)	95.5 (94.2,96.8)	0.0 (0.0,0.0)	0.4 (0.0,0.8)
**H**	**H**	**H**	**L**	0.0 (0.0,0.0)	0.0 (0.0,0.0)	0.0 (0.0,0.0)	95.0 (93.6,96.4)	86.4 (84.3,88.5)	95.9 (94.7,97.1)	95.4 (94.1,96.7)	0.0 (0.0,0.0)	0.3 (0.0,0.6)
**H**	**H**	**H**	**H**	0.0 (0.0,0.0)	0.0 (0.0,0.0)	0.0 (0.0,0.0)	92.8 (91.2,94.5)	41.7 (38.6,44.8)	85.2 (82.9,87.4)	81.6 (79.2,84.0)	0.0 (0.0,0.0)	0.1 (0.0,0.3)
**L**	**L**	**L**	**L**	0.0 (0.0,0.0)	0.0 (0.0,0.0)	0.0 (0.0,0.0)	40.5 (37.3,43.7)	93.0 (91.3,94.6)	1.1 (0.4,1.7)	1.1 (0.4,1.7)	0.0 (0.0,0.0)	0.0 (0.0,0.0)
**L**	**L**	**H**	**L**	0.0 (0.0,0.0)	0.0 (0.0,0.0)	0.0 (0.0,0.0)	39.5 (36.4,42.5)	93.0 (91.4,94.6)	3.2 (2.1,4.4)	3.5 (2.4,4.7)	0.0 (0.0,0.0)	0.0 (0.0,0.0)
**L**	**L**	**H**	**H**	11.2 (9.1,13.4)	13.8 (11.5,16.2)	14.3 (11.9,16.7)	90.1 (88.1,92.1)	8.3 (6.4,10.1)	81.8 (79.2,84.4)	82.0 (79.4,84.6)	2.6 (1.5,3.7)	75.3 (72.4,78.2)
**L**	**H**	**L**	**H**	0.0 (0.0,0.0)	0.0 (0.0,0.0)	0.0 (0.0,0.0)	93.7 (92.2,95.2)	93.1 (91.5,94.7)	0.4 (0.0,0.8)	93.8 (92.3,95.3)	0.0 (0.0,0.0)	0.0 (0.0,0.0)
**Sensitivity: L L H H**												

Specificity				π	Se_1	Se_2	Se_3	Se_4	Sp_1	Sp_2	Sp_3	Sp_4
**H**	**H**	**L**	**L**	0.0 (0.0,0.0)	0.0 (0.0,0.0)	0.0 (0.0,0.0)	94.0 (92.5,95.5)	94.9 (93.5,96.3)	88.1 (86.1,90.1)	88.3 (86.3,90.3)	0.0 (0.0,0.0)	0.0 (0.0,0.0)
**H**	**H**	**H**	**L**	0.0 (0.0,0.0)	0.0 (0.0,0.0)	0.0 (0.0,0.0)	94.2 (92.7,95.6)	67.2 (64.3,70.1)	83.6 (81.3,85.9)	85.5 (83.3,87.7)	0.0 (0.0,0.0)	0.0 (0.0,0.0)
**H**	**H**	**H**	**H**	32.4 (29.5,35.3)	26.3 (23.6,29.0)	27.5 (24.7,30.3)	78.1 (75.5,80.7)	76.6 (74.0,79.2)	85.1 (82.9,87.3)	84.9 (82.7,87.1)	17.6 (15.2,20.0)	18.6 (16.2,21.0)
**L**	**L**	**L**	**L**	0.0 (0.0,0.0)	0.0 (0.0,0.0)	0.0 (0.0,0.0)	89.8 (87.9,91.8)	91.0 (89.2,92.9)	10.3 (8.3,12.3)	10.9 (8.9,13.0)	0.0 (0.0,0.0)	0.0 (0.0,0.0)
**L**	**L**	**H**	**L**	61.7 (58.1,65.4)	36.1 (32.5,39.7)	39.1 (35.5,42.8)	93.8 (92.0,95.6)	38.6 (34.9,42.2)	81.4 (78.5,84.3)	81.0 (78.1,83.9)	20.7 (17.7,23.7)	85.5 (82.9,88.1)
**L**	**L**	**H**	**H**	91.4 (89.5,93.2)	87.0 (84.8,89.2)	84.8 (82.5,87.1)	93.9 (92.3,95.4)	93.9 (92.3,95.4)	91.6 (89.8,93.4)	91.5 (89.7,93.3)	91.9 (90.1,93.7)	91.7 (89.9,93.5)
**L**	**H**	**L**	**H**	0.7 (0.1,1.3)	0.0 (0.0,0.0)	0.8 (0.2,1.4)	47.5 (44.2,50.9)	91.4 (89.5,93.2)	0.9 (0.3,1.6)	94.4 (92.9,95.9)	2.5 (1.4,3.5)	0.0 (0.0,0.0)

iii. Setting 3: $\pi = 0.5, \omega = 0.9, n_{obs} = 2000$

Table iii.1 Number of converged data sets for different sensitivity-specificity combinations

				**Number of converged data sets**
**Specificity**				**Sensitivity: H H L L**
**H**	**H**	**L**	**L**	947
**H**	**H**	**H**	**L**	1000
**H**	**H**	**H**	**H**	1000
**L**	**L**	**L**	**L**	883
**L**	**L**	**H**	**L**	999
**L**	**L**	**H**	**H**	1000
**L**	**H**	**L**	**H**	1000
**Specificity**				**Sensitivity: H H H L**
**H**	**H**	**L**	**L**	1000
**H**	**H**	**H**	**L**	1000
**H**	**H**	**H**	**H**	1000
**L**	**L**	**L**	**L**	999
**L**	**L**	**H**	**L**	998
**L**	**L**	**H**	**H**	1000
**L**	**H**	**L**	**H**	999
**Specificity**				**Sensitivity: H H H H**
**H**	**H**	**L**	**L**	1000
**H**	**H**	**H**	**L**	1000
**H**	**H**	**H**	**H**	1000
**L**	**L**	**L**	**L**	1000
**L**	**L**	**H**	**L**	1000
**L**	**L**	**H**	**H**	1000
**L**	**H**	**L**	**H**	999

Specificity				Sensitivity: L L L L
H	H	L	L	887
H	H	H	L	998
H	H	H	H	1000
L	L	L	L	711
L	L	H	L	998
L	L	H	H	999
L	H	L	H	999
**Specificity**				**Sensitivity: L L H L**
H	H	L	L	998
H	H	H	L	999
H	H	H	H	1000
L	L	L	L	997
L	L	H	L	999
L	L	H	H	993
L	H	L	H	1000
**Specificity**				**Sensitivity: L L H H**
H	H	L	L	999
H	H	H	L	1000
H	H	H	H	996
L	L	L	L	998
L	L	H	L	994
L	L	H	H	735
L	H	L	H	1000

Table iii.2 Percentages of the time 95% credible intervals (CrIs) for residual correlations did not included '0' for any pairs, and percentages of the time that the overall χ^2 or G^2 statistic indicated a lack of overall fit

Sensitivity: H H L L						
**Specificity**				**Res. Cor.**	**Overall G^2**	**Overall χ^2**
H	H	L	L	60.2 (57.0,63.3)	14.8 (12.6,17.2)	13.6 (11.5,16.0)
H	H	H	L	47.8 (44.7,50.9)	34.3 (31.4,37.3)	30.4 (27.6,33.4)
H	H	H	H	88.3 (86.1,90.2)	94.7 (93.1,96.0)	98.3 (97.3,99.0)
L	L	L	L	63.8 (60.5,66.9)	44.1 (40.7,47.4)	43.7 (40.4,47.1)
L	L	H	L	69.1 (66.1,71.9)	95.7 (94.2,96.9)	95.9 (94.5,97.0)
L	L	H	H	98.6 (97.7,99.2)	99.6 (99.0,99.9)	100.0 (99.6,100.0)
L	H	L	H	74.0 (71.2,76.7)	80.4 (77.8,82.8)	79.5 (76.9,82.0)
**Sensitivity: H H H L**						
**Specificity**				**Res. Cor.**	**Overall G^2**	**Overall χ^2**
H	H	L	L	42.4 (39.3,45.5)	22.1 (19.6,24.8)	21.8 (19.3,24.5)

**H**	**H**	**H**	**L**	43.2 (40.1,16.3)	53.6 (50.5,56.7)	51.6 (48.5,54.7)
**H**	**H**	**H**	**H**	100.0 (99.6,100.0)	98.8 (97.9,99.4)	100.0 (99.6,100.0)
**L**	**L**	**L**	**L**	73.9 (71.0,76.6)	82.6 (80.1,84.9)	82.9 (80.4,85.2)
**L**	**L**	**H**	**L**	93.3 (91.6,94.8)	100.0 (99.6,100.0)	100.0 (99.6,100.0)
**L**	**L**	**H**	**H**	100.0 (99.6,100.0)	99.9 (99.4,100.0)	100.0 (99.6,100.0)
**L**	**H**	**L**	**H**	98.5 (97.5,99.2)	97.4 (96.2,98.3)	99.5 (98.8,99.8)
**Sensitivity: H H H H**						
**Specificity**				**Res. Cor.**	**Overall G^2**	**Overall χ^2**
**H**	**H**	**L**	**L**	92.8 (91.0,94.3)	65.5 (62.5,68.4)	65.9 (62.9,68.8)
**H**	**H**	**H**	**L**	100.0 (99.6,100.0)	99.6 (99.0,99.9)	99.9 (99.4,100.0)
**H**	**H**	**H**	**H**	100.0 (99.6,100.0)	99.9 (99.4,100.0)	100.0 (99.6,100.0)
**L**	**L**	**L**	**L**	98.6 (97.7,99.2)	99.8 (99.3,100.0)	99.9 (99.4,100.0)
**L**	**L**	**H**	**L**	100.0 (99.6,100.0)	100.0 (99.6,100.0)	100.0 (99.6,100.0)
**L**	**L**	**H**	**H**	100.0 (99.6,100.0)	100.0 (99.6,100.0)	100.0 (99.6,100.0)
**L**	**H**	**L**	**H**	100.0 (99.6,100.0)	99.3 (98.6,99.7)	100.0 (99.6,100.0)
**Sensitivity: L L L L**						
**Specificity**				**Res. Cor.**	**Overall G^2**	**Overall χ^2**
**H**	**H**	**L**	**L**	83.2 (80.6,85.6)	34.2 (31.0,37.4)	34.5 (31.4,37.7)
**H**	**H**	**H**	**L**	97.9 (96.8,98.7)	94.1 (92.4,95.5)	93.4 (91.7,94.8)
**H**	**H**	**H**	**H**	100.0 (99.6,100.0)	100.0 (99.6,100.0)	100.0 (99.6,100.0)
**L**	**L**	**L**	**L**	95.6 (93.9,97.0)	98.9 (97.8,99.5)	98.9 (97.8,99.5)
**L**	**L**	**H**	**L**	100.0 (99.6,100.0)	100.0 (99.6,100.0)	100.0 (99.6,100.0)
**L**	**L**	**H**	**H**	100.0 (99.6,100.0)	100.0 (99.6,100.0)	100.0 (99.6,100.0)
**L**	**H**	**L**	**H**	100.0 (99.6,100.0)	100.0 (99.6,100.0)	100.0 (99.6,100.0)
**Sensitivity: L L H L**						
**Specificity**				**Res. Cor.**	**Overall G^2**	**Overall χ^2**
**H**	**H**	**L**	**L**	98.9 (98.0,99.4)	88.5 (86.3,90.4)	88.8 (86.7,90.7)
**H**	**H**	**H**	**L**	100.0 (99.6,100.0)	99.9 (99.4,100.0)	99.9 (99.4,100.0)
**H**	**H**	**H**	**H**	100.0 (99.6,100.0)	100.0 (99.6,100.0)	100.0 (99.6,100.0)
**L**	**L**	**L**	**L**	100.0 (99.6,100.0)	100.0 (99.6,100.0)	100.0 (99.6,100.0)
**L**	**L**	**H**	**L**	100.0 (99.6,100.0)	100.0 (99.6,100.0)	100.0 (99.6,100.0)
**L**	**L**	**H**	**H**	100.0 (99.6,100.0)	100.0 (99.6,100.0)	100.0 (99.6,100.0)
**L**	**H**	**L**	**H**	100.0 (99.6,100.0)	99.2 (98.4,99.7)	100.0 (99.6,100.0)
**Sensitivity: L L H H**						
**Specificity**				**Res. Cor.**	**Overall G^2**	**Overall χ^2**
**H**	**H**	**L**	**L**	100.0 (99.6,100.0)	100.0 (99.6,100.0)	100.0 (99.6,100.0)
**H**	**H**	**H**	**L**	100.0 (99.6,100.0)	100.0 (99.6,100.0)	100.0 (99.6,100.0)
**H**	**H**	**H**	**H**	100.0 (99.6,100.0)	100.0 (99.6,100.0)	100.0 (99.6,100.0)
**L**	**L**	**L**	**L**	100.0 (99.6,100.0)	100.0 (99.6,100.0)	100.0 (99.6,100.0)
**L**	**L**	**H**	**L**	100.0 (99.6,100.0)	100.0 (99.6,100.0)	100.0 (99.6,100.0)
**L**	**L**	**H**	**H**	100.0 (99.5,100.0)	100.0 (99.5,100.0)	100.0 (99.5,100.0)

**L**	**H**	**L**	**H**	100.0 (99.6,100.0)	99.6 (99.0,99.9)	100.0 (99.6,100.0)

Table iii.3 Percentages of the time 95% CrIs for residual correlations did not included '0' for each pair, and percentages of the time the pairwise χ^2 or G^2 statistic for each pair indicated a lack of pairwise fit

**Sensitivity: H H L L**										
**Specificity**				**Tool**	**T_1&T_2**	**T_1&T_3**	**T_1&T_4**	**T_2&T_3**	**T_2&T_4**	**T_3&T_4**
**H**	**H**	**L**	**L**	**Res. Cor.**	0.0 (0.0,0.4)	0.0 (0.0,0.4)	0.0 (0.0,0.4)	0.0 (0.0,0.4)	0.0 (0.0,0.4)	60.2 (57.0,63.3)
				**Pairwise G^2**	0.0 (0.0,0.4)	0.0 (0.0,0.4)	0.0 (0.0,0.4)	0.0 (0.0,0.4)	0.0 (0.0,0.4)	5.2 (3.9,6.8)
				**Pairwise χ^2**	0.0 (0.0,0.4)	0.0 (0.0,0.4)	0.0 (0.0,0.4)	0.0 (0.0,0.4)	0.0 (0.0,0.4)	5.2 (3.9,6.8)
**H**	**H**	**H**	**L**	**Res. Cor.**	0.0 (0.0,0.4)	0.0 (0.0,0.4)	0.0 (0.0,0.4)	0.0 (0.0,0.4)	0.0 (0.0,0.4)	47.8 (44.7,50.9)
				**Pairwise G^2**	0.0 (0.0,0.4)	0.0 (0.0,0.4)	0.0 (0.0,0.4)	0.0 (0.0,0.4)	0.0 (0.0,0.4)	10.6 (8.8,12.7)
				**Pairwise χ^2**	0.0 (0.0,0.4)	0.0 (0.0,0.4)	0.0 (0.0,0.4)	0.0 (0.0,0.4)	0.0 (0.0,0.4)	10.6 (8.8,12.7)
**H**	**H**	**H**	**H**	**Res. Cor.**	0.0 (0.0,0.4)	0.0 (0.0,0.4)	0.0 (0.0,0.4)	0.0 (0.0,0.4)	0.0 (0.0,0.4)	88.3 (86.1,90.2)
				**Pairwise G^2**	0.0 (0.0,0.4)	0.0 (0.0,0.4)	0.0 (0.0,0.4)	0.0 (0.0,0.4)	0.0 (0.0,0.4)	63.1 (60.0,66.1)
				**Pairwise χ^2**	0.0 (0.0,0.4)	0.0 (0.0,0.4)	0.0 (0.0,0.4)	0.0 (0.0,0.4)	0.0 (0.0,0.4)	62.9 (59.8,65.9)
**L**	**L**	**L**	**L**	**Res. Cor.**	0.0 (0.0,0.4)	2.3 (1.4,3.5)	2.3 (1.4,3.5)	2.6 (1.7,3.9)	2.2 (1.3,3.3)	60.8 (57.5,64.1)
				**Pairwise G^2**	0.0 (0.0,0.4)	0.7 (0.2,1.5)	0.6 (0.2,1.3)	0.3 (0.1,1.0)	0.8 (0.3,1.6)	7.1 (5.5,9.0)
				**Pairwise χ^2**	0.0 (0.0,0.4)	0.7 (0.2,1.5)	0.7 (0.2,1.5)	0.3 (0.1,1.0)	0.8 (0.3,1.6)	7.1 (5.5,9.0)
**L**	**L**	**H**	**L**	**Res. Cor.**	0.0 (0.0,0.4)	10.4 (8.6,12.5)	4.7 (3.5,6.2)	11.2 (9.3,13.3)	5.7 (4.3,7.3)	60.3 (57.1,63.3)
				**Pairwise G^2**	0.0 (0.0,0.4)	4.0 (2.9,5.4)	0.5 (0.2,1.2)	3.7 (2.6,5.1)	1.4 (0.8,2.3)	21.6 (19.1,24.3)
				**Pairwise χ^2**	0.0 (0.0,0.4)	4.7 (3.5,6.2)	0.6 (0.2,1.3)	4.5 (3.3,6.0)	1.4 (0.8,2.3)	21.4 (18.9,24.1)
**L**	**L**	**H**	**H**	**Res. Cor.**	0.0 (0.0,0.4)	17.9 (15.6,20.4)	19.4 (17.0,22.0)	20.7 (18.2,23.3)	19.3 (16.9,21.9)	98.3 (97.3,99.0)
				**Pairwise G^2**	0.0 (0.0,0.4)	8.2 (6.6,10.1)	8.4 (6.8,10.3)	7.4 (5.9,9.2)	8.5 (6.8,10.4)	92.4 (90.6,94.0)
				**Pairwise χ^2**	0.0 (0.0,0.4)	8.4 (6.8,10.3)	9.8 (8.0,11.8)	8.8 (7.1,10.7)	9.7 (7.9,11.7)	92.4 (90.6,94.0)
**L**	**H**	**L**	**H**	**Res. Cor.**	0.0 (0.0,0.4)	12.8 (10.8,15.0)	50.4 (47.3,53.5)	0.0 (0.0,0.4)	0.0 (0.0,0.4)	46.3 (43.2,49.4)
				**Pairwise G^2**	0.0 (0.0,0.4)	1.6 (0.9,2.6)	27.4 (24.7,30.3)	0.0 (0.0,0.4)	0.0 (0.0,0.4)	13.1 (11.1,15.4)
				**Pairwise χ^2**	0.0 (0.0,0.4)	1.9 (1.1,3.0)	29.0 (26.2,31.9)	0.0 (0.0,0.4)	0.0 (0.0,0.4)	13.1 (11.1,15.4)
**Sensitivity: H H H L**										
**Specificity**				**Tool**	**T_1&T_2**	**T_1&T_3**	**T_1&T_4**	**T_2&T_3**	**T_2&T_4**	**T_3&T_4**
**H**	**H**	**L**	**L**	**Res. Cor.**	0.0 (0.0,0.4)	0.0 (0.0,0.4)	0.0 (0.0,0.4)	0.0 (0.0,0.4)	0.0 (0.0,0.4)	42.4 (39.3,45.5)
				**Pairwise G^2**	0.0 (0.0,0.4)	0.0 (0.0,0.4)	0.0 (0.0,0.4)	0.0 (0.0,0.4)	0.0 (0.0,0.4)	9.5 (7.8,11.5)
				**Pairwise χ^2**	0.0 (0.0,0.4)	0.0 (0.0,0.4)	0.0 (0.0,0.4)	0.0 (0.0,0.4)	0.0 (0.0,0.4)	9.5 (7.8,11.5)
**H**	**H**	**H**	**L**	**Res. Cor.**	0.0 (0.0,0.4)	0.0 (0.0,0.4)	0.0 (0.0,0.4)	0.0 (0.0,0.4)	0.0 (0.0,0.4)	43.2 (40.1,46.3)
				**Pairwise G^2**	0.0 (0.0,0.4)	0.0 (0.0,0.4)	0.0 (0.0,0.4)	0.0 (0.0,0.4)	0.0 (0.0,0.4)	17.5 (15.2,20.0)
				**Pairwise χ^2**	0.0 (0.0,0.4)	0.0 (0.0,0.4)	0.0 (0.0,0.4)	0.0 (0.0,0.4)	0.0 (0.0,0.4)	17.4 (15.1,19.9)
**H**	**H**	**H**	**H**	**Res. Cor.**	0.0 (0.0,0.4)	0.0 (0.0,0.4)	0.0 (0.0,0.4)	0.0 (0.0,0.4)	0.0 (0.0,0.4)	100.0 (99.6,100.0)
				**Pairwise G^2**	0.0 (0.0,0.4)	0.0 (0.0,0.4)	0.0 (0.0,0.4)	0.0 (0.0,0.4)	0.0 (0.0,0.4)	99.8 (99.3,100.0)
				**Pairwise χ^2**	0.0 (0.0,0.4)	0.0 (0.0,0.4)	0.0 (0.0,0.4)	0.0 (0.0,0.4)	0.0 (0.0,0.4)	99.8 (99.3,100.0)

**L**	**L**	**L**	**L**	**Res. Cor.**	0.0 (0.0,0.4)	20.0 (17.6,.22.6)	6.2 (4.8,7.9)	22.0 (19.5,24.7)	6.5 (5.1,8.2)	54.7 (51.5,57.8)
				**Pairwise G^2**	0.0 (0.0,0.4)	8.2 (6.6,10.1)	0.9 (0.4,1.7)	8.8 (7.1,10.7)	1.4 (0.8,2.3)	19.0 (16.6,21.6)
				**Pairwise χ^2**	0.0 (0.0,0.4)	8.4 (6.8,10.3)	0.9 (0.4,1.7)	8.8 (7.1,10.7)	1.4 (0.8,2.3)	18.9 (16.5,21.5)
**L**	**L**	**H**	**L**	**Res. Cor.**	0.0 (0.0,0.4)	53.2 (50.1,56.3)	11.3 (9.4,13.5)	55.7 (52.6,58.8)	10.3 (8.5,12.4)	66.2 (63.2,69.2)
				**Pairwise G^2**	0.0 (0.0,0.4)	35.3 (32.3,38.3)	1.9 (1.2,3.0)	36.8 (33.8,39.9)	1.7 (1.0,2.7)	40.7 (37.6,43.8)
				**Pairwise χ^2**	0.0 (0.0,0.4)	37.6 (34.6,40.7)	2.1 (1.3,3.2)	38.6 (35.5,41.7)	1.7 (1.0,2.7)	40.7 (37.6,43.8)
**L**	**L**	**H**	**H**	**Res. Cor.**	34.5 (31.6,37.5)	68.5 (65.5,71.4)	39.0 (36.0,42.1)	72.0 (69.1,74.8)	35.0 (32.0,38.0)	100.0 (99.6,100.0)
				**Pairwise G^2**	18.9 (16.5,21.5)	50.2 (47.1,53.3)	19.0 (16.6,21.6)	52.0 (48.9,55.1)	16.7 (14.4,19.2)	100.0 (99.6,100.0)
				**Pairwise χ^2**	18.5 (16.1,21.0)	52.0 (48.9,55.1)	20.3 (17.8,22.9)	54.7 (51.6,57.8)	18.1 (15.8,20.6)	100.0 (99.6,100.0)
**L**	**H**	**L**	**H**	**Res. Cor.**	0.0 (0.0,0.4)	54.8 (51.6,57.9)	54.4 (51.2,57.5)	0.0 (0.0,0.4)	0.0 (0.0,0.4)	96.8 (95.5,97.8)
				**Pairwise G^2**	0.0 (0.0,0.4)	22.5 (20.0,25.2)	29.7 (26.9,32.7)	0.0 (0.0,0.4)	0.0 (0.0,0.4)	83.8 (81.3,86.0)
				**Pairwise χ^2**	0.0 (0.0,0.4)	22.6 (20.1,25.3)	31.3 (28.5,34.3)	0.0 (0.0,0.4)	0.0 (0.0,0.4)	83.6 (81.1,85.8)
**Sensitivity: H H H H**										
**Specificity**				**Tool**	**T_1&T_2**	**T_1&T_3**	**T_1&T_4**	**T_2&T_3**	**T_2&T_4**	**T_3&T_4**
**H**	**H**	**L**	**L**	**Res. Cor.**	0.0 (0.0,0.4)	0.0 (0.0,0.4)	0.1 (0.0,0.6)	0.0 (0.0,0.4)	0.0 (0.0,0.4)	92.8 (91.0,94.3)
				**Pairwise G^2**	0.0 (0.0,0.4)	0.0 (0.0,0.4)	0.0 (0.0,0.4)	0.0 (0.0,0.4)	0.0 (0.0,0.4)	63.0 (59.9,66.0)
				**Pairwise χ^2**	0.0 (0.0,0.4)	0.0 (0.0,0.4)	0.0 (0.0,0.4)	0.0 (0.0,0.4)	0.0 (0.0,0.4)	63.0 (59.9,66.0)
**H**	**H**	**H**	**L**	**Res. Cor.**	0.0 (0.0,0.4)	0.0 (0.0,0.4)	0.2 (0.0,0.7)	0.0 (0.0,0.4)	0.1 (0.0,0.6)	100.0 (99.6,100.0)
				**Pairwise G^2**	0.0 (0.0,0.4)	0.0 (0.0,0.4)	0.0 (0.0,0.4)	0.0 (0.0,0.4)	0.0 (0.0,0.4)	99.6 (99.0,99.9)
				**Pairwise χ^2**	0.0 (0.0,0.4)	0.0 (0.0,0.4)	0.0 (0.0,0.4)	0.0 (0.0,0.4)	0.0 (0.0,0.4)	99.5 (98.8,99.8)
**H**	**H**	**H**	**H**	**Res. Cor.**	0.0 (0.0,0.4)	0.0 (0.0,0.4)	0.0 (0.0,0.4)	0.0 (0.0,0.4)	0.0 (0.0,0.4)	100.0 (99.6,100.0)
				**Pairwise G^2**	0.0 (0.0,0.4)	0.0 (0.0,0.4)	0.0 (0.0,0.4)	0.0 (0.0,0.4)	0.0 (0.0,0.4)	100.0 (99.6,100.0)
				**Pairwise χ^2**	0.0 (0.0,0.4)	0.0 (0.0,0.4)	0.0 (0.0,0.4)	0.0 (0.0,0.4)	0.0 (0.0,0.4)	100.0 (99.6,100.0)
**L**	**L**	**L**	**L**	**Res. Cor.**	4.0 (2.9,5.4)	39.2 (36.2,42.3)	41.9 (38.8,45.0)	39.0 (36.0,42.1)	37.8 (34.8,40.9)	96.8 (95.5,97.8)
				**Pairwise G^2**	2.1 (1.3,3.2)	18.5 (16.1,21.0)	17.4 (15.1,19.9)	17.1 (14.8,19.6)	14.8 (12.7,17.2)	81.6 (79.1,84.0)
				**Pairwise χ^2**	2.0 (1.2,3.1)	19.2 (16.8,21.8)	17.7 (15.4,20.2)	17.7 (15.4,20.2)	15.8 (13.6,18.2)	81.5 (79.0,83.9)
**L**	**L**	**H**	**L**	**Res. Cor.**	41.7 (38.6,44.8)	69.6 (66.6,72.4)	54.1 (51.0,57.2)	68.1 (65.1,71.0)	52.3 (49.2,55.4)	100.0 (99.6,100.0)
				**Pairwise G^2**	22.0 (19.5,24.7)	51.1 (48.0,54.2)	25.6 (22.9,28.4)	50.4 (47.3,53.5)	22.1 (19.6,24.8)	100.0 (99.6,100.0)
				**Pairwise χ^2**	21.4 (18.9,24.1)	53.4 (50.3,56.5)	26.1 (23.4,28.9)	52.4 (49.3,55.5)	22.5 (19.9,25.2)	100.0 (99.6,100.0)
**L**	**L**	**H**	**H**	**Res. Cor.**	85.5 (83.2,87.6)	80.5 (77.9,82.9)	78.8 (76.1,81.3)	80.0 (77.4,82.4)	78.7 (76.0,81.2)	100.0 (99.6,100.0)
				**Pairwise G^2**	59.9 (56.8,63.0)	63.0 (59.9,66.0)	61.2 (58.1,64.2)	62.8 (59.7,65.8)	61.0 (57.9,64.0)	100.0 (99.6,100.0)
				**Pairwise χ^2**	59.0 (55.9,62.1)	64.5 (61.4,67.5)	63.3 (60.2,66.3)	64.8 (61.7,67.8)	62.1 (59.0,65.1)	100.0 (99.6,100.0)
**L**	**H**	**L**	**H**	**Res. Cor.**	0.0 (0.0,0.4)	59.4 (56.2,62.4)	93.5 (91.8,94.9)	0.1 (0.0,0.6)	0.0 (0.0,0.4)	100.0 (99.6,100.0)
				**Pairwise G^2**	0.0 (0.0,0.4)	24.5 (21.9,27.3)	81.5 (78.9,83.8)	0.0 (0.0,0.4)	0.0 (0.0,0.4)	99.8 (99.3,100.0)
				**Pairwise χ^2**	0.0 (0.0,0.4)	24.9 (22.3,27.7)	82.8 (80.3,85.1)	0.0 (0.0,0.4)	0.0 (0.0,0.4)	99.8 (99.3,100.0)
**Sensitivity: L L L L**										
**Specificity**				**Tool**	**T_1&T_2**	**T_1&T_3**	**T_1&T_4**	**T_2&T_3**	**T_2&T_4**	**T_3&T_4**
**H**	**H**	**L**	**L**	**Res. Cor.**	0.0 (0.0,0.4)	0.0 (0.0,0.4)	0.0 (0.0,0.4)	0.0 (0.0,0.4)	0.1 (0.0,0.6)	83.2 (80.6,85.6)
				**Pairwise G^2**	0.0 (0.0,0.4)	0.0 (0.0,0.4)	0.0 (0.0,0.4)	0.0 (0.0,0.4)	0.0 (0.0,0.4)	16.3 (14.0,18.9)
				**Pairwise χ^2**	0.0 (0.0,0.4)	0.0 (0.0,0.4)	0.0 (0.0,0.4)	0.0 (0.0,0.4)	0.0 (0.0,0.4)	16.3 (14.0,18.9)
**H**	**H**	**H**	**L**	**Res. Cor.**	0.0 (0.0,0.4)	0.0 (0.0,0.4)	0.1 (0.0,0.6)	0.0 (0.0,0.4)	0.0 (0.0,0.4)	97.9 (96.8,98.7)

				**Pairwise G^2**	0.0 (0.0,0.4)	0.0 (0.0,0.4)	0.0 (0.0,0.4)	0.0 (0.0,0.4)	0.0 (0.0,0.4)	79.9 (77.2,82.3)
				**Pairwise χ^2**	0.0 (0.0,0.4)	0.0 (0.0,0.4)	0.0 (0.0,0.4)	0.0 (0.0,0.4)	0.0 (0.0,0.4)	79.9 (77.2,82.3)
**H**	**H**	**H**	**H**	**Res. Cor.**	0.0 (0.0,0.4)	0.0 (0.0,0.4)	0.1 (0.0,0.6)	0.0 (0.0,0.4)	0.1 (0.0,0.6)	100.0 (99.6,100.0)
				**Pairwise G^2**	0.0 (0.0,0.4)	0.0 (0.0,0.4)	0.1 (0.0,0.6)	0.0 (0.0,0.4)	0.0 (0.0,0.4)	100.0 (99.6,100.0)
				**Pairwise χ^2**	0.0 (0.0,0.4)	0.0 (0.0,0.4)	0.1 (0.0,0.6)	0.0 (0.0,0.4)	0.0 (0.0,0.4)	100.0 (99.6,100.0)
**L**	**L**	**L**	**L**	**Res. Cor.**	0.0 (0.0,0.5)	23.9 (20.8,27.2)	22.4 (19.3,25.6)	19.5 (16.7,22.7)	25.5 (22.3,28.8)	89.3 (86.8,91.5)
				**Pairwise G^2**	0.0 (0.0,0.5)	6.3 (4.7,8.4)	7.2 (5.4,9.3)	6.3 (4.7,8.4)	9.1 (7.1,11.5)	26.3 (23.1,29.7)
				**Pairwise χ^2**	0.0 (0.0,0.5)	6.5 (4.8,8.5)	7.2 (5.4,9.3)	6.8 (5.0,8.9)	9.3 (7.3,11.7)	26.6 (23.4,30.0)
**L**	**L**	**H**	**L**	**Res. Cor.**	0.0 (0.0,0.4)	99.8 (99.3,100.0)	36.4 (33.4,39.4)	99.9 (99.4,100.0)	35.8 (32.8,38.8)	100.0 (99.6,100.0)
				**Pairwise G^2**	0.0 (0.0,0.4)	97.7 (96.6,98.5)	13.0 (11.0,15.3)	98.3 (97.3,99.0)	12.7 (10.7,15.0)	97.2 (96.0,98.1)
				**Pairwise χ^2**	0.0 (0.0,0.4)	97.7 (96.6,98.5)	13.1 (11.1,15.4)	98.4 (97.4,99.1)	12.8 (10.8,15.1)	97.2 (96.0,98.1)
**L**	**L**	**H**	**H**	**Res. Cor.**	0.0 (0.0,0.4)	99.9 (99.4,100.0)	99.9 (99.4,100.0)	99.9 (99.4,100.0)	99.9 (99.4,100.0)	100.0 (99.6,100.0)
				**Pairwise G^2**	0.0 (0.0,0.4)	99.2 (98.4,99.7)	99.6 (99.0,99.9)	99.2 (98.4,99.7)	99.2 (98.4,99.7)	100.0 (99.6,100.0)
				**Pairwise χ^2**	0.0 (0.0,0.4)	99.3 (98.6,99.7)	99.6 (99.0,99.9)	99.2 (98.4,99.7)	99.4 (98.7,99.8)	100.0 (99.6,100.0)
**L**	**H**	**L**	**H**	**Res. Cor.**	0.0 (0.0,0.4)	69.5 (66.5,72.3)	100.0 (99.6,100.0)	0.0 (0.0,0.4)	0.0 (0.0,0.4)	99.0 (98.2,99.5)
				**Pairwise G^2**	0.0 (0.0,0.4)	35.3 (32.4,38.4)	100.0 (99.6,100.0)	0.0 (0.0,0.4)	0.0 (0.0,0.4)	86.3 (84.0,88.4)
				**Pairwise χ^2**	0.0 (0.0,0.4)	35.3 (32.4,38.4)	100.0 (99.6,100.0)	0.0 (0.0,0.4)	0.0 (0.0,0.4)	86.2 (83.9,88.3)
**Sensitivity: L L H L**										
**Specificity**				**Tool**	**T_1&T_2**	**T_1&T_3**	**T_1&T_4**	**T_2&T_3**	**T_2&T_4**	**T_3&T_4**
**H**	**H**	**L**	**L**	**Res. Cor.**	0.0 (0.0,0.4)	2.9 (2.0,4.1)	0.0 (0.0,0.4)	2.9 (2.0,4.1)	0.0 (0.0,0.4)	98.8 (97.9,99.4)
				**Pairwise G^2**	0.0 (0.0,0.4)	0.9 (0.4,1.7)	0.0 (0.0,0.4)	0.8 (0.3,1.6)	0.0 (0.0,0.4)	83.1 (80.6,85.3)
				**Pairwise χ^2**	0.0 (0.0,0.4)	1.0 (0.5,1.8)	0.0 (0.0,0.4)	1.0 (0.5,1.8)	0.0 (0.0,0.4)	83.1 (80.6,85.3)
**H**	**H**	**H**	**L**	**Res. Cor.**	0.0 (0.0,0.4)	3.0 (2.0,4.3)	0.0 (0.0,0.4)	2.9 (2.0,4.1)	0.0 (0.0,0.4)	100.0 (99.6,100.0)
				**Pairwise G^2**	0.0 (0.0,0.4)	1.4 (0.8,2.3)	0.0 (0.0,0.4)	1.3 (0.7,2.2)	0.0 (0.0,0.4)	99.6 (99.0,99.9)
				**Pairwise χ^2**	0.0 (0.0,0.4)	1.7 (1.0,2.7)	0.0 (0.0,0.4)	1.5 (0.8,2.5)	0.0 (0.0,0.4)	99.6 (99.0,99.9)
**H**	**H**	**H**	**H**	**Res. Cor.**	0.0 (0.0,0.4)	4.7 (3.5,6.2)	0.1 (0.0,0.6)	4.6 (3.4,6.1)	0.3 (0.1,0.9)	100.0 (99.6,100.0)
				**Pairwise G^2**	0.0 (0.0,0.4)	1.6 (0.9,2.6)	0.1 (0.0,0.6)	1.6 (0.9,2.6)	0.0 (0.0,0.4)	100.0 (99.6,100.0)
				**Pairwise χ^2**	0.0 (0.0,0.4)	1.7 (1.0,2.7)	0.1 (0.0,0.6)	1.8 (1.1,2.8)	0.0 (0.0,0.4)	100.0 (99.6,100.0)
**L**	**L**	**L**	**L**	**Res. Cor.**	0.0 (0.0,0.4)	99.6 (99.0,99.9)	39.3 (36.3,42.4)	99.3 (98.6,99.7)	35.6 (32.6,38.7)	99.9 (99.4,100.0)
				**Pairwise G^2**	0.0 (0.0,0.4)	95.6 (94.1,96.8)	16.1 (13.9,18.6)	96.7 (95.4,97.7)	14.8 (12.7,17.2)	95.2 (93.7,96.4)
				**Pairwise χ^2**	0.0 (0.0,0.4)	95.8 (94.3,96.9)	16.1 (13.9,18.6)	96.7 (95.4,97.7)	14.9 (12.8,17.3)	95.2 (93.7,96.4)
**L**	**L**	**H**	**L**	**Res. Cor.**	0.0 (0.0,0.4)	100.0 (99.6,100.0)	49.5 (46.4,52.7)	100.0 (99.6,100.0)	49.4 (46.3,52.6)	100.0 (99.6,100.0)
				**Pairwise G^2**	0.0 (0.0,0.4)	100.0 (99.6,100.0)	23.8 (21.2,26.6)	100.0 (99.6,100.0)	22.0 (19.5,24.7)	100.0 (99.6,100.0)
				**Pairwise χ^2**	0.0 (0.0,0.4)	100.0 (99.6,100.0)	24.1 (21.5,26.9)	100.0 (99.6,100.0)	22.2 (19.7,24.9)	100.0 (99.6,100.0)
**L**	**L**	**H**	**H**	**Res. Cor.**	19.3 (16.9,21.9)	100.0 (99.6,100.0)	100.0 (99.6,100.0)	100.0 (99.6,100.0)	100.0 (99.6,100.0)	100.0 (99.6,100.0)
				**Pairwise G^2**	29.1 (26.3,32.0)	100.0 (99.6,100.0)	100.0 (99.6,100.0)	100.0 (99.6,100.0)	100.0 (99.6,100.0)	100.0 (99.6,100.0)
				**Pairwise χ^2**	28.5 (25.7,31.4)	100.0 (99.6,100.0)	100.0 (99.6,100.0)	100.0 (99.6,100.0)	100.0 (99.6,100.0)	100.0 (99.6,100.0)
**L**	**H**	**L**	**H**	**Res. Cor.**	0.0 (0.0,0.4)	100.0 (99.6,100.0)	100.0 (99.6,100.0)	0.0 (0.0,0.4)	0.0 (0.0,0.4)	100.0 (99.6,100.0)
				**Pairwise G^2**	0.0 (0.0,0.4)	99.9 (99.4,100.0)	100.0 (99.6,100.0)	0.0 (0.0,0.4)	0.0 (0.0,0.4)	100.0 (99.6,100.0)
				**Pairwise χ^2**	0.0 (0.0,0.4)	99.9 (99.4,100.0)	100.0 (99.6,100.0)	0.0 (0.0,0.4)	0.0 (0.0,0.4)	100.0 (99.6,100.0)
**Sensitivity: L L H H**										

Specificity				Tool	T_1&T_2	T_1&T_3	T_1&T_4	T_2&T_3	T_2&T_4	T_3&T_4
H	H	L	L	**Res. Cor.**	0.0 (0.0,0.4)	3.0 (2.0,4.3)	3.2 (2.2,4.5)	2.8 (1.9,4.0)	3.8 (2.7,5.2)	100.0 (99.6,100.0)
				**Pairwise G^2**	0.0 (0.0,0.4)	0.8 (0.3,1.6)	1.3 (0.7,2.2)	0.8 (0.3,1.6)	0.9 (0.4,1.7)	100.0 (99.6,100.0)
				**Pairwise χ^2**	0.0 (0.0,0.4)	0.9 (0.4,1.7)	1.6 (0.9,2.6)	0.9 (0.4,1.7)	1.3 (0.7,2.2)	100.0 (99.6,100.0)
H	H	H	L	**Res. Cor.**	0.0 (0.0,0.4)	5.4 (4.1,7.0)	6.3 (4.9,8.0)	4.1 (3.0,5.5)	3.9 (2.8,5.3)	100.0 (99.6,100.0)
				**Pairwise G^2**	0.0 (0.0,0.4)	1.4 (0.8,2.3)	0.0 (0.0,0.4)	1.3 (0.7,2.2)	0.0 (0.0,0.4)	100.0 (99.6,100.0)
				**Pairwise χ^2**	0.0 (0.0,0.4)	1.7 (1.0,2.7)	0.0 (0.0,0.4)	1.5 (0.8,2.5)	0.0 (0.0,0.4)	100.0 (99.6,100.0)
H	H	H	H	**Res. Cor.**	0.0 (0.0,0.4)	12.4 (10.5,14.7)	12.8 (10.7,15.0)	10.9 (9.1,13.0)	14.1 (12.0,16.4)	100.0 (99.6,100.0)
				**Pairwise G^2**	0.0 (0.0,0.4)	5.9 (4.5,7.6)	5.7 (4.4,7.4)	5.4 (4.1,7.0)	5.6 (4.3,7.2)	100.0 (99.6,100.0)
				**Pairwise χ^2**	0.0 (0.0,0.4)	6.7 (5.3,8.5)	6.4 (5.0,8.1)	6.1 (4.7,7.8)	7.1 (5.6,8.9)	100.0 (99.6,100.0)
L	L	L	L	**Res. Cor.**	0.3 (0.1,0.9)	99.9 (99.4,100.0)	100.0 (99.6,100.0)	100.0 (99.6,100.0)	99.9 (99.4,100.0)	100.0 (99.6,100.0)
				**Pairwise G^2**	0.9 (0.4,1.7)	99.3 (98.6,99.7)	99.3 (98.6,99.7)	99.6 (99.0,99.9)	99.1 (98.3,99.6)	100.0 (99.6,100.0)
				**Pairwise χ^2**	0.7 (0.3,1.4)	99.3 (98.6,99.7)	99.3 (98.6,99.7)	99.6 (99.0,99.9)	99.1 (98.3,99.6)	100.0 (99.6,100.0)
L	L	H	L	**Res. Cor.**	88.6 (84.3,88.7)	100.0 (99.6,100.0)	100.0 (99.6,100.0)	100.0 (99.6,100.0)	100.0 (99.6,100.0)	100.0 (99.6,100.0)
				**Pairwise G^2**	88.8 (86.7,90.7)	100.0 (99.6,100.0)	100.0 (99.6,100.0)	100.0 (99.6,100.0)	100.0 (99.6,100.0)	100.0 (99.6,100.0)
				**Pairwise χ^2**	88.6 (86.5,90.5)	100.0 (99.6,100.0)	100.0 (99.6,100.0)	100.0 (99.6,100.0)	100.0 (99.6,100.0)	100.0 (99.6,100.0)
L	L	H	H	**Res. Cor.**	100.0 (99.5,100.0)	0.5 (0.1,1.4)	0.3 (0.0,0.1)	0.1 (0.0,0.8)	0.4 (0.1,1.2)	0.0 (0.0,0.5)
				**Pairwise G^2**	100.0 (99.5,100.0)	0.0 (0.0,0.5)	0.0 (0.0,0.5)	0.0 (0.0,0.5)	0.0 (0.0,0.5)	0.0 (0.0,0.5)
				**Pairwise χ^2**	100.0 (99.5,100.0)	0.0 (0.0,0.5)	0.0 (0.0,0.5)	0.0 (0.0,0.5)	0.0 (0.0,0.5)	0.0 (0.0,0.5)
L	H	L	H	**Res. Cor.**	0.1 (0.0,0.6)	100.0 (99.6,100.0)	100.0 (99.6,100.0)	1.0 (0.5,1.8)	0.0 (0.0,0.4)	100.0 (99.6,100.0)
				**Pairwise G^2**	0.1 (0.0,0.6)	99.9 (99.4,100.0)	100.0 (99.6,100.0)	0.4 (0.1,1.0)	0.0 (0.0,0.4)	100.0 (99.6,100.0)
				**Pairwise χ^2**	0.1 (0.0,0.6)	99.9 (99.4,100.0)	100.0 (99.6,100.0)	0.4 (0.1,1.0)	0.0 (0.0,0.4)	100.0 (99.6,100.0)

Table iii.4 Mean absolute biases (x10^{-2}) of posterior medians of each parameter obtained from the conditional independence model)

**Sensitivity: H H L L**												
**Specificity**				π	Se_1	Se_2	Se_3	Se_4	Sp_1	Sp_2	Sp_3	Sp_4
H	H	L	L	-3.7 (-3.7,-3.6)	8.2 (8.2,8.3)	8.2 (8.2,8.3)	-0.2 (-0.3,-0.1)	-0.2 (-0.3,0.0)	1.5 (1.4,1.6)	1.5 (1.4,1.6)	-1.6 (-1.7,-1.5)	-1.6 (-1.6,-1.5)
H	H	H	L	-4.7 (-4.8,-4.6)	9.2 (9.2,9.2)	9.2 (9.2,9.3)	-0.3 (-0.4,-0.2)	-0.1 (-0.2,0.0)	0.7 (0.6,0.7)	0.7 (0.6,0.7)	-4.5 (-4.6,-4.4)	-1.8 (-1.9,-1.7)
H	H	H	H	-4.8 (-4.9,-4.8)	9.3 (9.3,9.3)	9.3 (9.3,9.3)	-0.1 (-0.2,0.0)	-0.1 (-0.2,0.0)	0.5 (0.5,0.6)	0.6 (0.5,0.6)	-4.6 (-4.6,-4.5)	-4.5 (-4.6,-4.4)
L	L	L	L	-1.0 (-1.2,-0.9)	7.8 (7.8,7.9)	7.9 (7.8,7.9)	-1.3 (-1.4,-1.2)	-1.3 (-1.4,-1.2)	6.3 (6.1,6.4)	6.4 (6.2,6.5)	-1.6 (-1.7,-1.5)	-1.6 (-1.7,-1.4)
L	L	H	L	-3.7 (-3.8,-3.6)	9.5 (9.4,9.5)	9.4 (9.4,9.5)	-2.1 (-2.3,-2.0)	-0.6 (-0.7,-0.5)	4.5 (4.3,4.6)	4.5 (4.4,4.6)	-5.4 (-5.5,-5.3)	-2.1 (-2.2,-2.0)
L	L	H	H	-4.4 (-4.5,-4.3)	9.6 (9.6,9.6)	9.6 (9.6,9.6)	-1.5 (-1.6,-1.4)	-1.5 (-1.6,-1.4)	3.9 (3.8,4.1)	4.0 (3.8,4.1)	-5.3 (-5.4,-5.2)	-5.4 (-5.4,-5.3)
L	H	L	H	-4.7 (-4.7,-4.6)	9.2 (9.1,9.2)	9.5 (9.5,9.5)	-0.2 (-0.3,-0.1)	-0.5 (-0.6,-0.3)	3.4 (3.3,3.5)	1.0 (0.9,1.1)	-2.0 (-2.1,-1.9)	-4.7 (-4.7,-4.6)
**Sensitivity: H H H L**												
**Specificity**				π	Se_1	Se_2	Se_3	Se_4	Sp_1	Sp_2	Sp_3	Sp_4
H	H	L	L	-4.5 (-4.6,-4.4)	9.1 (9.0,9.1)	9.1 (9.0,9.1)	-0.1 (-0.2,-0.1)	-0.1 (-0.2,0.1)	0.8 (0.7,0.8)	0.8 (0.7,0.8)	-4.4 (-4.5,-4.3)	-1.7 (-1.8,-1.6)
H	H	H	L	-5.0 (-5.1,-4.9)	9.5 (9.4,9.5)	9.5 (9.5,9.5)	-0.2 (-0.2,-0.1)	0.0 (-0.1,0.1)	0.4 (0.4,0.5)	0.5 (0.4,0.5)	-7.5 (-7.5,-7.4)	-1.9 (-2.0,-1.8)
H	H	H	H	-4.9 (-4.9,-4.8)	9.3 (9.3,9.3)	9.3 (9.3,9.4)	-0.2 (-0.2,-0.1)	0.0 (-0.1,0.1)	0.5 (0.4,0.6)	0.5 (0.5,0.6)	-7.3 (-7.4,-7.2)	-4.5 (-4.6,-4.4)

**L**	**L**	**L**	**L**	-3.3 (-3.4,-3.2)	9.2 (9.1,9.2)	9.1 (9.1,9.2)	-2.2 (-2.3,-2.1)	-0.8 (-0.9,-0.6)	4.9 (4.7,5.0)	4.8 (4.7,5.0)	-4.9 (-5.0,-4.9)	-1.9 (-2.0,-1.8)
**L**	**L**	**H**	**L**	-4.3 (-4.4,-4.2)	9.7 (9.7,9.7)	9.7 (9.7,9.7)	-2.9 (-3.0,-2.8)	-0.4 (-0.5,-0.2)	4.1 (4.0,4.3)	4.1 (4.0,4.2)	-8.9 (-9.0,-8.8)	-1.9 (-2.0,-1.8)
**L**	**L**	**H**	**H**	-5.6 (-5.7,-5.5)	9.7 (9.7,9.7)	9.7 (9.7,9.7)	-0.6 (-0.7,-0.5)	0.0 (-0.2,0.1)	2.7 (2.6,2.8)	2.6 (2.5,2.7)	-8.6 (-8.7,-8.5)	-5.1 (-5.2,-5.0)
**L**	**H**	**L**	**H**	-4.8 (-4.9,-4.7)	9.2 (9.2,9.2)	9.5 (9.5,9.5)	-0.2 (-0.3,-0.2)	-0.4 (-0.5,-0.3)	3.2 (3.1,3.3)	0.8 (0.8,0.9)	-4.7 (-4.8,-4.6)	-4.7 (-4.8,-4.7)
**Sensitivity: H H H H**												
**Specificity**				π	Se_1	Se_2	Se_3	Se_4	Sp_1	Sp_2	Sp_3	Sp_4
**H**	**H**	**L**	**L**	-4.7 (-4.8,-4.6)	9.2 (9.2,9.2)	9.2 (9.2,9.2)	-0.1 (-0.2,-0.1)	-0.1 (-0.2,0.0)	0.6 (0.6,0.7)	0.6 (0.6,0.7)	-4.4 (-4.5,-4.3)	-4.4 (-4.5,-4.3)
**H**	**H**	**H**	**L**	-4.9 (-5.0,-4.8)	9.4 (9.3,9.4)	9.4 (9.4,9.4)	-0.1 (-0.2,0.0)	-0.1 (-0.1,0.0)	0.5 (0.4,0.6)	0.5 (0.4,0.5)	-7.3 (-7.4,-7.3)	-4.6 (-4.7,-4.5)
**H**	**H**	**H**	**H**	-4.9 (-4.9,-4.8)	9.2 (9.2,9.2)	9.2 (9.2,9.2)	-0.1 (-0.1,0.0)	-0.1 (-0.2,0.0)	0.5 (0.5,0.6)	0.6 (0.5,0.6)	-7.1 (-7.2,-7.0)	-7.1 (-7.2,-7.0)
**L**	**L**	**L**	**L**	-4.5 (-4.6,-4.4)	9.4 (9.4,9.4)	9.5 (9.4,9.5)	-0.9 (-1.0,-0.8)	-0.8 (-0.9,-0.7)	3.6 (3.5,3.7)	3.6 (3.5,3.7)	-4.9 (-5.0,-4.8)	-4.8 (-4.9,-4.7)
**L**	**L**	**H**	**L**	-5.6 (-5.7,-5.5)	9.7 (9.7,9.7)	9.7 (9.7,9.7)	-0.7 (-0.8,-0.6)	0.3 (0.2,0.3)	2.8 (2.7,2.9)	2.7 (2.6,2.8)	-8.6 (-8.7,-8.5)	-4.9 (-5.0,-4.8)
**L**	**L**	**H**	**H**	-6.4 (-6.5,-6.3)	9.6 (9.6,9.6)	9.6 (9.6,9.6)	0.9 (0.8,1.0)	0.9 (0.8,1.0)	1.8 (1.7,1.9)	1.7 (1.6,1.8)	-8.4 (-8.5,-8.3)	-8.3 (-8.4,-8.3)
**L**	**H**	**L**	**H**	-5.1 (-5.1,-5.0)	9.3 (9.3,9.3)	9.7 (9.7,9.7)	-0.1 (-0.1,0.0)	-0.3 (-0.4,-0.2)	3.0 (2.9,3.1)	0.5 (0.4,0.5)	-4.7 (-4.8,-4.6)	-7.7 (-7.8,-7.6)
**Sensitivity: L L L L**												
**Specificity**				π	Se_1	Se_2	Se_3	Se_4	Sp_1	Sp_2	Sp_3	Sp_4
**H**	**H**	**L**	**L**	-19.2 (-19.3,-19.1)	37.2 (37.1,37.3)	37.2 (37.1,37.3)	-0.3 (-0.5,-0.2)	-0.2 (-0.4,-0.1)	2.5 (2.4,2.5)	2.4 (2.4,2.5)	-5.8 (-5.9,-5.7)	-5.8 (-5.9,-5.7)
**H**	**H**	**H**	**L**	-20.5 (-20.5,-20.4)	38.8 (38.8,38.9)	38.9 (38.8,38.9)	-0.5 (-0.7,-0.4)	-0.1 (-0.2,0.1)	1.7 (1.6,1.7)	1.7 (1.6,1.7)	-14.7 (-14.8,-14.7)	-6.0 (-6.0,-5.9)
**H**	**H**	**H**	**H**	-20.5 (-20.6,-20.5)	38.9 (38.9,39.0)	38.9 (38.9,39.0)	-0.3 (-0.5,-0.2)	-0.2 (-0.4,-0.1)	1.6 (1.6,1.7)	1.6 (1.6,1.7)	-14.7 (-14.8,-14.6)	-14.8 (-14.8,-14.7)
**L**	**L**	**L**	**L**	-15.5 (-15.6,-15.4)	37.5 (37.5,37.6)	37.6 (37.5,37.6)	-3.2 (-3.4,-3.1)	-3.3 (-3.5,-3.2)	14.7 (14.6,14.9)	14.7 (14.6,14.9)	-6.4 (-6.5,-6.3)	-6.5 (-6.6,-6.4)
**L**	**L**	**H**	**L**	-17.5 (-17.6,-17.4)	39.4 (39.4,39.4)	39.4 (39.4,39.4)	-7.8 (-7.9,-7.6)	-2.8 (-2.9,-2.7)	13.7 (13.6,13.8)	13.7 (13.6,13.8)	-16.8 (-16.9,-16.7)	-6.6 (-6.7,-6.5)
**L**	**L**	**H**	**H**	-18.0 (-18.1,-17.9)	39.6 (39.6,39.6)	39.6 (39.6,39.6)	-6.9 (-7.0,-6.8)	-6.9 (-7.0,-6.7)	13.3 (13.2,13.4)	13.3 (13.2,13.4)	-16.6 (-16.7,-16.5)	-16.5 (-16.6,-16.4)
**L**	**H**	**L**	**H**	-20.1 (-20.2,-20.0)	38.5 (38.4,38.5)	39.5 (39.5,39.5)	-0.7 (-0.8,-0.6)	-1.8 (-1.9,-1.7)	10.5 (10.5,10.6)	2.4 (2.3,2.4,)	-6.1 (-6.2,-6.0)	-15.1 (-15.2,-15.1)
**Sensitivity: L L H L**												
**Specificity**				π	Se_1	Se_2	Se_3	Se_4	Sp_1	Sp_2	Sp_3	Sp_4
**H**	**H**	**L**	**L**	-20.4 (-20.4,-20.3)	38.7 (38.7,38.8)	38.7 (38.7,38.8)	-0.5 (-0.5,-0.4)	-0.2 (-0.3,-0.1)	1.7 (1.7,1.8)	1.7 (1.7,1.8)	-14.7 (-14.7,-14.6)	-5.9 (-6.0,-5.8)
**H**	**H**	**H**	**L**	-20.8 (-20.9,-20.8)	39.4 (39.3,39.4)	39.4 (39.3,39.4)	-0.5 (-0.6,-0.4)	-0.1 (-0.3,0.0)	1.4 (1.3,1.4)	1.4 (1.4,1.4)	-23.8 (-23.9,-23.7)	-6.0 (-6.1,-5.9)
**H**	**H**	**H**	**H**	-20.6 (-20.6,-20.5)	38.9 (38.9,39.0)	38.9 (38.9,39.0)	-0.4 (-0.5-0.3)	-0.1 (-0.2,0.0)	1.6 (1.5,1.6)	1.6 (1.5,1.6)	-23.5 (-23.6,-23.4)	-14.6 (-14.7,-14.6)
**L**	**L**	**L**	**L**	-17.3 (-17.4,-17.3)	39.3 (39.2,39.3)	39.3 (39.3,39.3)	-7.7 (-7.8,-7.6)	-2.8 (-2.9,-2.7)	13.8 (13.7,13.9)	13.8 (13.7,13.9)	-16.5 (-16.6,-16.4)	-6.5 (-6.6,-6.4)
**L**	**L**	**H**	**L**	-18.0 (-18.0,-17.9)	39.7 (39.7,39.7)	39.7 (39.7,39.7)	-11.8 (-11.9,-11.7)	-2.2 (-2.3,-2.0)	13.4 (13.3,13.5)	13.4 (13.3,13.5)	-26.7 (-26.8,-26.7)	-6.4 (-6.5,-6.3)

**L**	**L**	**H**	**H**	-19.3 (-19.4,-19.2)	39.7 (39.7,39.7)	39.7 (39.7,39.7)	-8.6 (-8.7,-8.4)	-4.3 (-4.5,-4.2)	11.9 (11.8,12.0)	12.0 (11.9,12.1)	-26.1 (-26.2,-26.0)	-15.9 (-16.0,-15.8)
**L**	**H**	**L**	**H**	-20.4 (-20.4,-20.3)	38.5 (38.4,38.5)	39.6 (39.6,39.6)	-1.3 (-1.4,-1.2)	-1.4 (-1.5,-1.2)	10.4 (10.4,10.5)	2.2 (2.2,2.3)	-15.0 (-15.0,-14.9)	-15.0 (-15.1,-14.9)
**Sensitivity: L L H H**												
**Specificity**				$\boldsymbol{\pi}$	$\boldsymbol{Se_1}$	$\boldsymbol{Se_2}$	$\boldsymbol{Se_3}$	$\boldsymbol{Se_4}$	$\boldsymbol{Sp_1}$	$\boldsymbol{Sp_2}$	$\boldsymbol{Sp_3}$	$\boldsymbol{Sp_4}$
**H**	**H**	**L**	**L**	-20.5 (-20.6,-20.4)	38.9 (38.8,38.9)	38.9 (38.8,38.9)	-0.4 (-0.5,-0.3)	-0.3 (-0.4,-0.3)	1.6 (1.6,1.7)	1.6 (1.6,1.7)	-14.7 (-14.8,-14.6)	-14.7 (-14.8,-14.7)
**H**	**H**	**H**	**L**	-20.6 (-20.7,-20.5)	39.0 (39.0,39.0)	39.0 (39.0,39.0)	-0.4 (-0.5,-0.3)	-0.1 (-0.2,0.0)	1.5 (1.4,1.5)	1.5 (1.5,1.6)	-23.6 (-23.6,-23.5)	-14.7 (-14.8,-14.6)
**H**	**H**	**H**	**H**	-20.3 (-20.4,-20.3)	38.4 (38.4,38.5)	38.4 (38.3,38.4)	-0.1 (-0.2,0.0)	-0.2 (-0.2,-0.1)	1.7 (1.6,1.7)	1.7 (1.6,1.7)	-23.2 (-23.3,-23.1)	-23.2 (-23.3,-23.1)
**L**	**L**	**L**	**L**	-18.6 (-18.7,-18.5)	39.6 (39.6,39.6)	39.6 (39.6,39.6)	-5.4 (-5.5,-5.3)	-5.4 (-5.6,-5.3)	12.7 (12.6,12.8)	12.6 (12.5,12.7)	-16.1 (-16.1,-16.0)	-16.0 (-16.1,-15.9)
**L**	**L**	**H**	**L**	-20.3 (-20.4,-20.2)	39.7 (39.7,39.7)	39.7 (39.7,39.7)	-6.4 (-6.6,-6.2)	-1.8 (-2.0,-1.7)	11.0 (10.9,11.1)	11.0 (10.9,11.1)	-25.8 (-25.9,-25.7)	-15.3 (-15.4,-15.2)
**L**	**L**	**H**	**H**	-1.2 (-1.3,-1.0)	1.6 (1.4,1.7)	1.6 (1.4,1.7)	0.8 (0.6,0.9)	0.8 (0.7,1.0)	1.0 (0.9,1.1)	1.0 (0.8,1.1)	-1.1 (-1.3,-1.0)	-1.2 (-1.3,-1.0)
**L**	**H**	**L**	**H**	-20.8 (-20.9,-20.7)	38.6 (38.5,38.6)	39.7 (39.7,39.7)	-0.5 (-0.6,-0.4)	-1.4 (-1.5,-1.3)	10.1 (10.0,10.2)	1.7 (1.7,1.8)	-14.9 (-14.9,-14.8)	-24.1 (-24.1,-24.0)

Table iii.5 Coverages of the 95% CrIs for each model parameter

**Sensitivity: H H L L**												
**Specificity**				$\boldsymbol{\pi}$	$\boldsymbol{Se_1}$	$\boldsymbol{Se_2}$	$\boldsymbol{Se_3}$	$\boldsymbol{Se_4}$	$\boldsymbol{Sp_1}$	$\boldsymbol{Sp_2}$	$\boldsymbol{Sp_3}$	$\boldsymbol{Sp_4}$
**H**	**H**	**L**	**L**	33.6 (30.6,36.6)	0.7 (0.2,1.3)	0.4 (0.0,0.8)	95.0 (93.7,96.4)	94.2 (92.7,95.7)	85.7 (83.5,88.0)	83.8 (81.5,86.2)	82.6 (80.2,85.0)	84.8 (82.5,87.1)
**H**	**H**	**H**	**L**	2.5 (1.5,3.5)	0.0 (0.0,0.0)	0.0 (0.0,0.0)	95.1 (93.8,96.4)	94.1 (92.6,95.6)	91.8 (90.1,93.5)	91.1 (89.3,92.9)	0.5 (0.1,0.9)	77.5 (74.9,80.1)
**H**	**H**	**H**	**H**	0.9 (0.3,1.5)	0.0 (0.0,0.0)	0.0 (0.0,0.0)	95.4 (94.1,96.7)	95.9 (94.7,97.1)	92.1 (90.4,93.8)	93.4 (91.9,94.9)	0.5 (0.1,0.9)	0.7 (0.2,1.2)
**L**	**L**	**L**	**L**	98.8 (98.0,99.5)	8.2 (6.3,10.0)	7.4 (5.6,9.1)	87.9 (85.7,90.0)	90.1 (88.2,92.1)	18.6 (16.0,21.1)	16.9 (14.4,19.3)	85.7 (83.4,88.0)	87.3 (85.1,89.5)
**L**	**L**	**H**	**L**	32.9 (30.0,35.8)	0.0 (0.0,0.0)	0.0 (0.0,0.0)	79.5 (77.0,82.0)	93.2 (91.6,94.8)	34.1 (31.2,37.1)	32.4 (29.5,35.3)	0.0 (0.0,0.0)	77.0 (74.4,79.6)
**L**	**L**	**H**	**H**	13.5 (11.4,15.6)	0.0 (0.0,0.0)	0.0 (0.0,0.0)	87.8 (85.8,89.8)	86.6 (84.5,88.7)	41.9 (38.8,45.0)	42.5 (39.4,45.6)	0.2 (0.0,0.5)	0.0 (0.0,0.0)
**L**	**H**	**L**	**H**	4.4 (3.1,5.7)	0.0 (0.0,0.0)	0.0 (0.0,0.0)	94.5 (93.1,95.9)	93.6 (92.1,95.1)	43.0 (39.9,46.1)	89.0 (87.1,90.9)	75.0 (72.3,77.7)	0.4 (0.0,0.8)
**Sensitivity: H H H L**												
**Specificity**				$\boldsymbol{\pi}$	$\boldsymbol{Se_1}$	$\boldsymbol{Se_2}$	$\boldsymbol{Se_3}$	$\boldsymbol{Se_4}$	$\boldsymbol{Sp_1}$	$\boldsymbol{Sp_2}$	$\boldsymbol{Sp_3}$	$\boldsymbol{Sp_4}$
**H**	**H**	**L**	**L**	3.7 (2.5,4.9)	0.0 (0.0,0.0)	0.0 (0.0,0.0)	95.3 (94.0,96.6)	95.1 (93.8,96.4)	90.5 (88.7,92.3)	89.9 (88.0,91.8)	20.5 (18.0,23.0)	80.7 (78.3,83.1)
**H**	**H**	**H**	**L**	0.7 (0.2,1.2)	0.0 (0.0,0.0)	0.0 (0.0,0.0)	94.7 (93.3,96.1)	94.1 (92.6,95.6)	92.7 (91.1,94.3)	92.4 (90.8,94.0)	0.0 (0.0,0.0)	75.0 (72.3,77.7)
**H**	**H**	**H**	**H**	1.4 (0.7,2.1)	0.0 (0.0,0.0)	0.0 (0.0,0.0)	93.9 (92.4,95.4)	95.8 (94.6,97.0)	92.5 (90.9,94.1)	92.8 (91.2,94.4)	0.0 (0.0,0.0)	0.3 (0.0,0.6)
**L**	**L**	**L**	**L**	54.3 (51.2,57.3)	0.0 (0.0,0.0)	0.0 (0.0,0.0)	70.0 (67.1,72.8)	92.5 (90.9,94.1)	30.1 (27.3,33.0)	27.4 (24.7,30.2)	16.2 (13.9,18.5)	78.0 (75.4,80.5)
**L**	**L**	**H**	**L**	15.0 (12.8,17.2)	0.0 (0.0,0.0)	0.0 (0.0,0.0)	65.6 (62.7,98.6)	93.7 (92.2,95.2)	37.5 (34.5,40.5)	38.3 (35.3,41.3)	0.0 (0.0,0.0)	75.6 (72.9,78.2)
**L**	**L**	**H**	**H**	1.4 (0.7,2.1)	0.0 (0.0,0.0)	0.0 (0.0,0.0)	92.9 (91.3,94.5)	94.0 (92.5,95.5)	65.2 (62.2,68.2)	63.9 (60.9,66.9)	0.0 (0.0,0.0)	0.3 (0.0,0.6)
**L**	**H**	**L**	**H**	2.5 (1.5,3.5)	0.0 (0.0,0.0)	0.0 (0.0,0.0)	94.3 (92.9,95.7)	95.1 (93.8,96.4)	47.0 (44.0,50.1)	89.5 (87.6,91.4)	12.8 (10.7,14.9)	0.5 (0.1,0.9)

Sensitivity: H H H H												
**Specificity**				π	Se_1	Se_2	Se_3	Se_4	Sp_1	Sp_2	Sp_3	Sp_4
**H**	**H**	**L**	**L**	1.7 (0.9,2.5)	0.0 (0.0,0.0)	0.0 (0.0,0.0)	94.6 (93.2,96.0)	95.0 (93.6,96.4)	92.2 (90.5,93.9)	91.3 (89.6,93.0)	18.7 (16.3,21.1)	15.6 (13.4,17.8)
**H**	**H**	**H**	**L**	0.6 (0.1,1.1)	0.0 (0.0,0.0)	0.0 (0.0,0.0)	94.8 (93.4,96.2)	95.1 (93.8,96.4)	93.0 (91.4,94.6)	93.3 (91.8,94.8)	0.0 (0.0,0.0)	15.9 (13.6,18.2)
**H**	**H**	**H**	**H**	1.0 (0.4,1.6)	0.0 (0.0,0.0)	0.0 (0.0,0.0)	95.5 (94.2,96.8)	94.6 (93.2,96.0)	92.5 (90.9,94.1)	91.0 (89.2,92.8)	0.0 (0.0,0.0)	0.0 (0.0,0.0)
**L**	**L**	**L**	**L**	14.5 (12.3,16.7)	0.0 (0.0,0.0)	0.0 (0.0,0.0)	89.5 (87.6,91.4)	90.3 (88.5,92.1)	47.7 (44.6,50.8)	49.2 (46.1,52.3)	15.3 (13.1,17.5)	15.1 (12.9,17.3)
**L**	**L**	**H**	**L**	0.6 (0.1,1.1)	0.0 (0.0,0.0)	0.0 (0.0,0.0)	91.9 (90.2,93.6)	95.0 (93.6,96.4)	61.7 (58.7,64.7)	64.6 (61.6,67.6)	0.0 (0.0,0.0)	13.2 (11.1,15.3)
**L**	**L**	**H**	**H**	0.0 (0.0,0.0)	0.0 (0.0,0.0)	0.0 (0.0,0.0)	88.6 (86.6,90.6)	89.4 (87.5,91.3)	80.8 (78.4,83.2)	79.9 (77.4,82.4)	0.0 (0.0,0.0)	0.0 (0.0,0.0)
**L**	**H**	**L**	**H**	0.7 (0.2,1.2)	0.0 (0.0,0.0)	0.0 (0.0,0.0)	95.4 (94.1,96.7)	94.3 (92.9,95.7)	48.0 (44.9,51.1)	92.9 (91.3,94.5)	12.0 (10.0,14.0)	0.0 (0.0,0.0)
**Sensitivity: L L L L**												
**Specificity**				π	Se_1	Se_2	Se_3	Se_4	Sp_1	Sp_2	Sp_3	Sp_4
**H**	**H**	**L**	**L**	0.0 (0.0,0.0)	0.0 (0.0,0.0)	0.0 (0.0,0.0)	94.0 (92.5,95.6)	94.1 (92.6,95.7)	37.0 (33.8,40.2)	39.9 (36.7,43.1)	0.5 (0.0,0.9)	1.5 (0.7,2.3)
**H**	**H**	**H**	**L**	0.0 (0.0,0.0)	0.0 (0.0,0.0)	0.0 (0.0,0.0)	94.9 (93.5,96.3)	94.4 (93.0,95.8)	54.5 (51.4,57.6)	53.0 (49.9,56.1)	0.0 (0.0,0.0)	1.1 (0.5,1.7)
**H**	**H**	**H**	**H**	0.0 (0.0,0.0)	0.0 (0.0,0.0)	0.0 (0.0,0.0)	95.0 (93.6,96.4)	95.2 (93.9,96.5)	53.2 (50.1,56.3)	53.3 (50.2,56.4)	0.0 (0.0,0.0)	0.0 (0.0,0.0)
**L**	**L**	**L**	**L**	0.4 (0.0,0.9)	0.0 (0.0,0.0)	0.0 (0.0,0.0)	66.7 (63.2,70.1)	64.4 (60.9,67.9)	0.0 (0.0,0.0)	0.0 (0.0,0.0)	0.7 (0.1,1.3)	0.6 (0.1,1.1)
**L**	**L**	**H**	**L**	0.0 (0.0,0.0)	0.0 (0.0,0.0)	0.0 (0.0,0.0)	6.3 (4.8,7.8)	71.7 (69.0,74.5)	0.0 (0.0,0.0)	0.0 (0.0,0.0)	0.0 (0.0,0.0)	0.4 (0.0,0.8)
**L**	**L**	**H**	**H**	0.0 (0.0,0.0)	0.0 (0.0,0.0)	0.0 (0.0,0.0)	13.4 (11.3,15.5)	13.4 (11.3,15.5)	0.0 (0.0,0.0)	0.0 (0.0,0.0)	0.0 (0.0,0.0)	0.0 (0.0,0.0)
**L**	**H**	**L**	**H**	0.0 (0.0,0.0)	0.0 (0.0,0.0)	0.0 (0.0,0.0)	93.8 (92.3,95.3)	85.4 (83.2,87.6)	0.0 (0.0,0.0)	40.7 (37.7,43.8)	0.2 (0.0,0.5)	0.0 (0.0,0.0)
**Sensitivity: L L H L**												
**Specificity**				π	Se_1	Se_2	Se_3	Se_4	Sp_1	Sp_2	Sp_3	Sp_4
**H**	**H**	**L**	**L**	0.0 (0.0,0.0)	0.0 (0.0,0.0)	0.0 (0.0,0.0)	93.3 (91.7,94.8)	94.9 (93.5,96.3)	52.0 (48.9,55.1)	53.0 (49.9,56.1)	0.0 (0.0,0.0)	0.4 (0.0,0.8)
**H**	**H**	**H**	**L**	0.0 (0.0,0.0)	0.0 (0.0,0.0)	0.0 (0.0,0.0)	93.8 (92.3,95.3)	95.2 (93.9,96.5)	63.6 (60.6,66.5)	62.4 (59.4,65.4)	0.0 (0.0,0.0)	0.4 (0.0,0.8)
**H**	**H**	**H**	**H**	0.0 (0.0,0.0)	0.0 (0.0,0.0)	0.0 (0.0,0.0)	93.6 (92.1,95.1)	94.0 (92.5,95.5)	53.5 (50.4,56.6)	53.8 (50.7,56.9)	0.0 (0.0,0.0)	0.0 (0.0,0.0)
**L**	**L**	**L**	**L**	0.0 (0.0,0.0)	0.0 (0.0,0.0)	0.0 (0.0,0.0)	1.4 (0.7,2.1)	75.0 (72.3,77.7)	0.0 (0.0,0.0)	0.0 (0.0,0.0)	0.0 (0.0,0.0)	0.4 (0.0,0.8)
**L**	**L**	**H**	**L**	0.0 (0.0,0.0)	0.0 (0.0,0.0)	0.0 (0.0,0.0)	0.0 (0.0,0.0)	82.2 (79.8,84.6)	0.0 (0.0,0.0)	0.0 (0.0,0.0)	0.0 (0.0,0.0)	0.4 (0.0,0.8)
**L**	**L**	**H**	**H**	0.0 (0.0,0.0)	0.0 (0.0,0.0)	0.0 (0.0,0.0)	9.2 (7.4,11.0)	54.3 (51.2,57.4)	0.0 (0.0,0.0)	0.0 (0.0,0.0)	0.0 (0.0,0.0)	0.0 (0.0,0.0)
**L**	**H**	**L**	**H**	0.0 (0.0,0.0)	0.0 (0.0,0.0)	0.0 (0.0,0.0)	86.7 (84.6,88.8)	91.2 (89.4,93.0)	0.0 (0.0,0.0)	42.1 (39.0,45.2)	0.0 (0.0,0.0)	0.0 (0.0,0.0)
**Sensitivity: L L H H**												
**Specificity**				π	Se_1	Se_2	Se_3	Se_4	Sp_1	Sp_2	Sp_3	Sp_4
**H**	**H**	**L**	**L**	0.0 (0.0,0.0)	0.0 (0.0,0.0)	0.0 (0.0,0.0)	92.8 (91.2,94.4)	93.2 (91.6,94.8)	54.6 (51.5,57.6)	56.2 (53.1,59.2)	0.0 (0.0,0.0)	0.0 (0.0,0.0)
**H**	**H**	**H**	**L**	0.0 (0.0,0.0)	0.0 (0.0,0.0)	0.0 (0.0,0.0)	93.7 (92.2,95.2)	94.7 (93.3,96.1)	58.3 (55.2,61.4)	58.1 (55.0,61.2)	0.0 (0.0,0.0)	0.0 (0.0,0.0)
**H**	**H**	**H**	**H**	0.0 (0.0,0.0)	0.0 (0.0,0.0)	0.0 (0.0,0.0)	94.4 (92.9,95.8)	94.0 (92.5,95.5)	48.4 (45.3,51.5)	47.3 (44.2,50.4)	0.0 (0.0,0.0)	0.0 (0.0,0.0)
**L**	**L**	**L**	**L**	0.0 (0.0,0.0)	0.0 (0.0,0.0)	0.0 (0.0,0.0)	18.8 (16.4,21.3)	17.6 (15.3,20.0)	0.0 (0.0,0.0)	0.0 (0.0,0.0)	0.0 (0.0,0.0)	0.0 (0.0,0.0)
**L**	**L**	**H**	**L**	0.0 (0.0,0.0)	0.0 (0.0,0.0)	0.0 (0.0,0.0)	24.4 (21.8,27.1)	79.2 (76.7,81.7)	0.0 (0.0,0.0)	0.0 (0.0,0.0)	0.0 (0.0,0.0)	0.0 (0.0,0.0)
**L**	**L**	**H**	**H**	91.2 (89.1,93.2)	83.9 (81.3,86.6)	85.0 (82.5,87.6)	94.4 (92.8,96.1)	92.5 (90.6,94.4)	90.6 (88.5,92.7)	92.5 (90.6,94.4)	91.7 (89.7,93.7)	90.1 (87.9,92.2)
**L**	**H**	**L**	**H**	0.0 (0.0,0.0)	0.0 (0.0,0.0)	0.0 (0.0,0.0)	94.2 (92.8,95.6)	83.9 (81.6,86.2)	0.0 (0.0,0.0)	60.7 (57.7,63.7)	0.0 (0.0,0.0)	0.0 (0.0,0.0)

iv. Setting 4: $\pi = 0.5, \omega = 0.9, n_{obs} = 500$

Table iv.1 Number of converged data sets for different sensitivity-specificity combinations

				Number of converged data sets
Specificity				Sensitivity: H H L L
H	H	L	L	978
H	H	H	L	1000
H	H	H	H	999
L	L	L	L	823
L	L	H	L	999
L	L	H	H	1000
L	H	L	H	999
Specificity				Sensitivity: H H H L
H	H	L	L	1000
H	H	H	L	1000
H	H	H	H	999
L	L	L	L	998
L	L	H	L	1000
L	L	H	H	1000
L	H	L	H	999
Specificity				Sensitivity: H H H H
H	H	L	L	1000
H	H	H	L	1000
H	H	H	H	1000
L	L	L	L	1000
L	L	H	L	1000
L	L	H	H	1000
L	H	L	H	1000
Specificity				Sensitivity: L L L L
H	H	L	L	952
H	H	H	L	994
H	H	H	H	1000
L	L	L	L	670
L	L	H	L	963
L	L	H	H	994
L	H	L	H	996
Specificity				Sensitivity: L L H L
H	H	L	L	999
H	H	H	L	1000

H	H	H	H	1000
L	L	L	L	977
L	L	H	L	998
L	L	H	H	974
L	H	L	H	999
Specificity				Sensitivity: L L H H
H	H	L	L	1000
H	H	H	L	1000
H	H	H	H	983
L	L	L	L	999
L	L	H	L	982
L	L	H	H	650
L	H	L	H	1000

Table iv.2 Percentages of the time 95% credible intervals (CrIs) for residual correlations did not included '0' for any pairs, and percentages of the time that the overall χ^2 or G^2 statistic indicated a lack of overall fit

Sensitivity: H H L L						
Specificity				Res. Cor.	Overall G^2	Overall χ^2
H	H	L	L	60.2 (57.1,63.3)	12.5 (10.5,14.7)	11.6 (9.6,13.7)
H	H	H	L	30.4 (27.6,33.4)	4.3 (3.1,5.7)	12.0 (10.1,14.2)
H	H	H	H	51.1 (47.9,54.2)	12.9 (10.9,15.2)	58.1 (54.9,61.1)
L	L	L	L	56.0 (52.5,59.4)	23.9 (21.1,27.0)	22.7 (19.9,25.7)
L	L	H	L	32.9 (30.0,35.9)	45.5 (42.4,48.7)	51.1 (47.9,54.2)
L	L	H	H	58.2 (55.1,61.3)	48.4 (45.3,51.5)	90.9 (88.9,92.6)
L	H	L	H	37.4 (34.4,40.5)	16.3 (14.1,18.8)	30.9 (28.1,33.9)
Sensitivity: H H H L						
Specificity				Res. Cor.	Overall G^2	Overall χ^2
H	H	L	L	30.1 (27.3,33.0)	14.6 (12.5,16.9)	12.7 (10.7,14.9)
H	H	H	L	12.4 (10.4,14.6)	10.1 (8.3,12.1)	17.9 (15.6,20.4)
H	H	H	H	67.3 (64.3,70.2)	35.1 (32.2,38.2)	88.0 (85.8,89.9)
L	L	L	L	35.5 (32.5,38.5)	38.2 (35.2,41.3)	38.4 (35.3,41.5)
L	L	H	L	26.9 (37.7,43.9)	73.7 (70.9,76.4)	79.4 (76.8,81.9)
L	L	H	H	79.8 (77.2,82.2)	69.0 (66.0,71.9)	99.6 (99.0,99.9)
L	H	L	H	61.3 (58.2,64.3)	30.7 (27.9,33.7)	65.3 (62.2,68.2)
Sensitivity: H H H H						
Specificity				Res. Cor.	Overall G^2	Overall χ^2
H	H	L	L	50.4 (47.3,53.5)	26.2 (23.5,29.0)	26.9 (24.2,29.8)
H	H	H	L	73.9 (71.1,76.6)	29.1 (26.3,32.0)	64.1 (61.0,67.1)
H	H	H	H	99.0 (98.2,99.5)	45.1 (42.0,48.2)	99.9 (99.4,100.0)

**L**	**L**	**L**	**L**	60.3 (57.2,63.3)	70.1 (67.2,72.9)	70.9 (68.0,73.7)
**L**	**L**	**H**	**L**	85.0 (82.6,87.2)	91.0 (89.1,92.7)	97.4 (96.2,98.3)
**L**	**L**	**H**	**H**	99.4 (98.7,99.8)	82.2 (79.7,84.5)	100.0 (99.6,100.0)
**L**	**H**	**L**	**H**	84.7 (82.3,86.9)	51.9 (48.8,55.0)	91.1 (89.2,92.8)
**Sensitivity: L L L L**						
**Specificity**				**Res. Cor.**	**Overall G^2**	**Overall χ^2**
**H**	**H**	**L**	**L**	76.8 (74.0,79.4)	21.2 (18.7,24.0)	19.2 (16.8,21.9)
**H**	**H**	**H**	**L**	76.6 (73.8,79.2)	35.1 (32.1,38.2)	43.4 (40.3,46.5)
**H**	**H**	**H**	**H**	99.5 (98.8,99.8)	71.6 (68.7,74.4)	98.9 (98.0,99.4)
**L**	**L**	**L**	**L**	87.8 (85.0,90.1)	60.0 (56.2,63.7)	59.1 (55.3,62.9)
**L**	**L**	**H**	**L**	95.1 (93.6,96.4)	96.7 (95.3,97.7)	99.4 (98.6,99.8)
**L**	**L**	**H**	**H**	100.0 (99.6,100.0)	88.3 (86.2,90.3)	100.0 (99.6,100.0)
**L**	**H**	**L**	**H**	97.5 (96.3,98.4)	81.2 (78.7,83.6)	95.6 (94.1,96.8)
**Sensitivity: L L H L**						
**Specificity**				**Res. Cor.**	**Overall G^2**	**Overall χ^2**
**H**	**H**	**L**	**L**	75.9 (73.1,78.5)	37.4 (34.4,40.5)	36.1 (33.2,39.2)
**H**	**H**	**H**	**L**	90.8 (88.8,92.5)	69.0 (66.0,71.9)	73.8 (71.0,76.5)
**H**	**H**	**H**	**H**	100.0 (99.6,100.0)	72.7 (69.8,75.4)	100.0 (99.6,100.0)
**L**	**L**	**L**	**L**	98.3 (89.7,93.3)	98.1 (97.0,98.8)	97.9 (96.7,98.7)
**L**	**L**	**H**	**L**	100.0 (99.6,100.0)	98.7 (97.8,99.3)	100.0 (99.6,100.0)
**L**	**L**	**H**	**H**	100.0 (99.6,100.0)	95.8 (94.3,97.0)	100.0 (99.6,100.0)
**L**	**H**	**L**	**H**	100.0 (99.6,100.0)	66.5 (63.4,69.4)	99.9 (99.4,100.0)
**Sensitivity: L L H H**						
**Specificity**				**Res. Cor.**	**Overall G^2**	**Overall χ^2**
**H**	**H**	**L**	**L**	99.9 (99.4,100.0)	86.0 (83.7,88.1)	94.8 (93.2,96.1)
**H**	**H**	**H**	**L**	100.0 (99.6,100.0)	65.2 (62.2,68.2)	100.0 (99.6,100.0)
**H**	**H**	**H**	**H**	100.0 (99.6,100.0)	71.3 (68.4,74.1)	100.0 (99.6,100.0)
**L**	**L**	**L**	**L**	100.0 (99.6,100.0)	100.0 (99.6,100.0)	100.0 (99.6,100.0)
**L**	**L**	**H**	**L**	100.0 (99.6,100.0)	97.1 (95.9,98.1)	100.0 (99.6,100.0)
**L**	**L**	**H**	**H**	100.0 (99.4,100.0)	98.2 (96.8,99.0)	100.0 (99.4,100.0)
**L**	**H**	**L**	**H**	100.0 (99.6,100.0)	75.8 (73.0,78.4)	100.0 (99.6,100.0)

Table iv.3 Percentages of the time 95% CrIs for residual correlations did not included '0' for each pair, and percentages of the time the pairwise χ^2 or G^2 statistic for each pair indicated a lack of pairwise fit

**Sensitivity: H H L L**										
**Specificity**				**Tool**	**T_1&T_2**	**T_1&T_3**	**T_1&T_4**	**T_2&T_3**	**T_2&T_4**	**T_3&T_4**
**H**	**H**	**L**	**L**	**Res. Cor.**	0.0 (0.0,0.4)	0.0 (0.0,0.4)	0.0 (0.0,0.4)	0.0 (0.0,0.4)	0.0 (0.0,0.4)	60.2 (57.1,63.3)
				**Pairwise G^2**	0.0 (0.0,0.4)	0.0 (0.0,0.4)	0.0 (0.0,0.4)	0.0 (0.0,0.4)	0.0 (0.0,0.4)	3.9 (2.8,5.3)

				**Pairwise χ^2**	0.0 (0.0,0.4)	0.0 (0.0,0.4)	0.0 (0.0,0.4)	0.0 (0.0,0.4)	0.0 (0.0,0.4)	3.9 (2.8,5.3)
**H**	**H**	**H**	**L**	**Res. Cor.**	0.0 (0.0,0.4)	0.0 (0.0,0.4)	0.0 (0.0,0.4)	0.0 (0.0,0.4)	0.0 (0.0,0.4)	30.4 (27.6,33.4)
				**Pairwise G^2**	0.0 (0.0,0.4)	0.0 (0.0,0.4)	0.0 (0.0,0.4)	0.0 (0.0,0.4)	0.0 (0.0,0.4)	4.3 (3.1,5.7)
				**Pairwise χ^2**	0.0 (0.0,0.4)	0.0 (0.0,0.4)	0.0 (0.0,0.4)	0.0 (0.0,0.4)	0.0 (0.0,0.4)	4.2 (3.0,5.6)
**H**	**H**	**H**	**H**	**Res. Cor.**	0.0 (0.0,0.4)	0.0 (0.0,0.4)	0.0 (0.0,0.4)	0.0 (0.0,0.4)	0.0 (0.0,0.4)	51.1 (47.9,54.2)
				**Pairwise G^2**	0.0 (0.0,0.4)	0.0 (0.0,0.4)	0.0 (0.0,0.4)	0.0 (0.0,0.4)	0.0 (0.0,0.4)	18.9 (16.5,21.5)
				**Pairwise χ^2**	0.0 (0.0,0.4)	0.0 (0.0,0.4)	0.0 (0.0,0.4)	0.0 (0.0,0.4)	0.0 (0.0,0.4)	18.4 (16.1,21.0)
**L**	**L**	**L**	**L**	**Res. Cor.**	0.0 (0.0,0.4)	1.2 (0.6,2.2)	1.0 (0.4,1.9)	1.1 (0.5,2.1)	1.7 (0.9,2.8)	54.7 (51.2,58.1)
				**Pairwise G^2**	0.0 (0.0,0.4)	0.0 (0.0,0.4)	0.2 (0.0,0.9)	0.1 (0.0,0.7)	0.0 (0.0,0.4)	4.6 (3.3,6.3)
				**Pairwise χ^2**	0.0 (0.0,0.4)	0.0 (0.0,0.4)	0.2 (0.0,0.9)	0.1 (0.0,0.7)	0.0 (0.0,0.4)	4.7 (3.4,6.4)
**L**	**L**	**H**	**L**	**Res. Cor.**	0.0 (0.0,0.4)	0.0 (0.0,0.4)	2.7 (1.8,3.9)	0.2 (0.0,0.7)	4.1 (3.0,5.5)	29.2 (26.4,32.2)
				**Pairwise G^2**	0.0 (0.0,0.4)	0.0 (0.0,0.4)	0.4 (0.1,1.0)	0.1 (0.0,0.6)	0.2 (0.0,0.7)	6.3 (4.9,8.0)
				**Pairwise χ^2**	0.0 (0.0,0.4)	0.0 (0.0,0.4)	0.4 (0.1,1.0)	0.1 (0.0,0.6)	0.2 (0.0,0.7)	6.2 (4.8,7.9)
**L**	**L**	**H**	**H**	**Res. Cor.**	0.0 (0.0,0.4)	1.0 (0.5,1.8)	0.7 (0.3,1.4)	0.8 (0.3,1.6)	0.3 (0.1,0.9)	57.8 (54.7,60.9)
				**Pairwise G^2**	0.0 (0.0,0.4)	0.1 (0.0,0.6)	0.0 (0.0,0.4)	0.1 (0.0,0.6)	0.0 (0.0,0.4)	32.3 (29.4,35.3)
				**Pairwise χ^2**	0.0 (0.0,0.4)	0.3 (0.1,0.9)	0.1 (0.0,0.6)	0.2 (0.0,0.7)	0.0 (0.0,0.4)	32.1 (29.2,35.1)
**L**	**H**	**L**	**H**	**Res. Cor.**	0.0 (0.0,0.4)	8.9 (7.2,10.8)	4.4 (3.2,5.9)	0.1 (0.0,0.6)	0.0 (0.0,0.4)	28.9 (26.1,31.8)
				**Pairwise G^2**	0.0 (0.0,0.4)	1.4 (0.8,2.3)	0.3 (0.1,0.9)	0.0 (0.0,0.4)	0.0 (0.0,0.4)	4.1 (3.0,5.5)
				**Pairwise χ^2**	0.0 (0.0,0.4)	1.4 (0.8,2.3)	0.5 (0.2,1.2)	0.0 (0.0,0.4)	0.0 (0.0,0.4)	4.0 (2.9,5.4)
**Sensitivity: H H H L**										
**Specificity**				**Tool**	**T_1&T_2**	**T_1&T_3**	**T_1&T_4**	**T_2&T_3**	**T_2&T_4**	**T_3&T_4**
**H**	**H**	**L**	**L**	**Res. Cor.**	0.0 (0.0,0.4)	0.0 (0.0,0.4)	0.0 (0.0,0.4)	0.0 (0.0,0.4)	0.1 (0.0,0.6)	30.0 (27.2,32.9)
				**Pairwise G^2**	0.0 (0.0,0.4)	0.0 (0.0,0.4)	0.0 (0.0,0.4)	0.0 (0.0,0.4)	0.0 (0.0,0.4)	2.6 (1.7,3.8)
				**Pairwise χ^2**	0.0 (0.0,0.4)	0.0 (0.0,0.4)	0.0 (0.0,0.4)	0.0 (0.0,0.4)	0.0 (0.0,0.4)	2.6 (1.7,3.8)
**H**	**H**	**H**	**L**	**Res. Cor.**	0.0 (0.0,0.4)	0.0 (0.0,0.4)	0.0 (0.0,0.4)	0.0 (0.0,0.4)	0.0 (0.0,0.4)	12.4 (10.4,14.6)
				**Pairwise G^2**	0.0 (0.0,0.4)	0.0 (0.0,0.4)	0.0 (0.0,0.4)	0.0 (0.0,0.4)	0.0 (0.0,0.4)	2.3 (1.5,3.4)
				**Pairwise χ^2**	0.0 (0.0,0.4)	0.0 (0.0,0.4)	0.0 (0.0,0.4)	0.0 (0.0,0.4)	0.0 (0.0,0.4)	2.1 (1.3,3.2)
**H**	**H**	**H**	**H**	**Res. Cor.**	0.0 (0.0,0.4)	0.0 (0.0,0.4)	0.0 (0.0,0.4)	0.0 (0.0,0.4)	0.0 (0.0,0.4)	67.3 (64.3,70.2)
				**Pairwise G^2**	0.0 (0.0,0.4)	0.0 (0.0,0.4)	0.0 (0.0,0.4)	0.0 (0.0,0.4)	0.0 (0.0,0.4)	47.8 (44.7,51.0)
				**Pairwise χ^2**	0.0 (0.0,0.4)	0.0 (0.0,0.4)	0.0 (0.0,0.4)	0.0 (0.0,0.4)	0.0 (0.0,0.4)	45.3 (42.2,48.5)
**L**	**L**	**L**	**L**	**Res. Cor.**	0.0 (0.0,0.4)	2.4 (1.5,3.6)	3.5 (2.5,4.8)	3.5 (2.5,4.8)	3.0 (2.0,4.3)	28.3 (25.5,31.2)
				**Pairwise G^2**	0.0 (0.0,0.4)	0.4 (0.1,1.0)	0.4 (0.1,1.0)	0.9 (0.4,1.7)	0.4 (0.1,1.0)	5.3 (4.0,6.9)
				**Pairwise χ^2**	0.0 (0.0,0.4)	0.5 (0.2,1.2)	0.4 (0.1,1.0)	0.9 (0.4,1.7)	0.4 (0.1,1.0)	5.2 (3.9,6.8)
**L**	**L**	**H**	**L**	**Res. Cor.**	0.0 (0.0,0.4)	1.3 (0.7,2.2)	5.4 (4.1,7.0)	1.8 (1.1,2.8)	4.8 (3.6,6.3)	18.0 (15.7,20.5)
				**Pairwise G^2**	0.0 (0.0,0.4)	0.3 (0.1,0.9)	0.8 (0.3,1.6)	0.6 (0.2,1.3)	0.5 (0.2,1.2)	5.5 (4.2,7.1)
				**Pairwise χ^2**	0.0 (0.0,0.4)	0.3 (0.1,0.9)	0.8 (0.3,1.6)	0.8 (0.3,1.6)	0.5 (0.2,1.2)	5.4 (4.1,7.0)
**L**	**L**	**H**	**H**	**Res. Cor.**	8.7 (7.0,10.6)	3.7 (2.6,5.1)	2.6 (1.7,3.8)	4.8 (3.6,6.3)	3.1 (2.1,4.4)	78.6 (75.9,81.1)
				**Pairwise G^2**	4.4 (3.2,5.9)	0.6 (0.2,1.3)	0.4 (0.1,1.0)	0.9 (0.4,1.7)	0.3 (0.1,0.9)	69.2 (66.2,72.1)
				**Pairwise χ^2**	4.0 (2.9,5.4)	0.9 (0.4,1.7)	0.6 (0.2,1.3)	1.4 (0.8,2.3)	0.5 (0.2,1.2)	66.9 (63.9,69.8)
**L**	**H**	**L**	**H**	**Res. Cor.**	0.0 (0.0,0.4)	19.1 (16.7,21.7)	7.1 (5.6,8.9)	0.0 (0.0,0.4)	0.0 (0.0,0.4)	52.8 (49.6,55.9)
				**Pairwise G^2**	0.0 (0.0,0.4)	3.8 (2.7,5.2)	0.7 (0.3,1.4)	0.0 (0.0,0.4)	0.0 (0.0,0.4)	24.5 (21.9,27.3)

				**Pairwise χ^2**	0.0 (0.0,0.4)	3.9 (2.8,5.3)	1.3 (0.7,2.2)	0.0 (0.0,0.4)	0.0 (0.0,0.4)	22.0 (19.5,24.7)
**Sensitivity: H H H H**										
**Specificity**				**Tool**	**T_1&T_2**	**T_1&T_3**	**T_1&T_4**	**T_2&T_3**	**T_2&T_4**	**T_3&T_4**
				**Res. Cor.**	0.0 (0.0,0.4)	0.0 (0.0,0.4)	0.0 (0.0,0.4)	0.0 (0.0,0.4)	0.1 (0.0,0.6)	50.4 (47.3,53.5)
**H**	**H**	**L**	**L**	**Pairwise G^2**	0.0 (0.0,0.4)	0.0 (0.0,0.4)	0.0 (0.0,0.4)	0.0 (0.0,0.4)	0.0 (0.0,0.4)	14.5 (12.4,16.8)
				**Pairwise χ^2**	0.0 (0.0,0.4)	0.0 (0.0,0.4)	0.0 (0.0,0.4)	0.0 (0.0,0.4)	0.0 (0.0,0.4)	14.3 (12.2,16.6)
				**Res. Cor.**	0.0 (0.0,0.4)	0.0 (0.0,0.4)	0.0 (0.0,0.4)	0.0 (0.0,0.4)	0.0 (0.0,0.4)	73.9 (71.1,76.6)
**H**	**H**	**H**	**L**	**Pairwise G^2**	0.0 (0.0,0.4)	0.0 (0.0,0.4)	0.0 (0.0,0.4)	0.0 (0.0,0.4)	0.0 (0.0,0.4)	47.5 (44.4,50.6)
				**Pairwise χ^2**	0.0 (0.0,0.4)	0.0 (0.0,0.4)	0.0 (0.0,0.4)	0.0 (0.0,0.4)	0.0 (0.0,0.4)	46.3 (43.2,49.4)
				**Res. Cor.**	0.0 (0.0,0.4)	0.0 (0.0,0.4)	0.0 (0.0,0.4)	0.0 (0.0,0.4)	0.0 (0.0,0.4)	99.0 (98.2,99.5)
**H**	**H**	**H**	**H**	**Pairwise G^2**	0.0 (0.0,0.4)	0.0 (0.0,0.4)	0.0 (0.0,0.4)	0.0 (0.0,0.4)	0.0 (0.0,0.4)	95.7 (94.3,96.9)
				**Pairwise χ^2**	0.0 (0.0,0.4)	0.0 (0.0,0.4)	0.0 (0.0,0.4)	0.0 (0.0,0.4)	0.0 (0.0,0.4)	95.0 (93.5,96.3)
				**Res. Cor.**	1.5 (0.8,2.5)	7.6 (6.0,9.4)	7.2 (5.7,9.0)	10.0 (8.2,12.0)	7.8 (6.2,9.6)	50.5 (47.4,53.6)
**L**	**L**	**L**	**L**	**Pairwise G^2**	0.7 (0.3,1.4)	1.8 (1.1,2.8)	1.7 (1.0,2.7)	2.8 (1.9,4.0)	1.6 (0.9,2.6)	20.2 (17.8,22.8)
				**Pairwise χ^2**	0.7 (0.3,1.4)	1.8 (1.1,2.8)	1.7 (1.0,2.7)	2.9 (2.0,4.1)	1.7 (1.0,2.7)	20.0 (17.6,22.6)
				**Res. Cor.**	10.5 (8.7,12.6)	4.6 (3.4,6.1)	12.9 (10.9,15.1)	3.4 (2.4,4.7)	12.7 (10.7,14.9)	80.7 (78.1,83.1)
**L**	**L**	**H**	**L**	**Pairwise G^2**	3.6 (2.5,4.9)	1.3 (0.7,2.2)	2.8 (1.9,4.0)	1.0 (0.5,1.8)	3.1 (2.1,4.4)	61.3 (58.2,64.3)
				**Pairwise χ^2**	3.6 (2.5,4.9)	1.7 (1.0,2.7)	2.8 (1.9,4.0)	1.2 (0.6,2.1)	3.3 (2.3,4.6)	58.3 (55.2,61.4)
				**Res. Cor.**	40.2 (37.1,43.3)	7.3 (5.8,9.1)	5.9 (4.5,7.5)	6.0 (4.6,7.7)	6.8 (5.3,8.5)	99.4 (98.7,99.8)
**L**	**L**	**H**	**H**	**Pairwise G^2**	17.1 (14.8,19.6)	1.7 (1.0,2.7)	1.5 (0.8,2.5)	1.8 (1.1,2.8)	1.4 (0.8,2.3)	97.8 (96.7,98.6)
				**Pairwise χ^2**	16.2 (14.0,18.6)	2.3 (1.5,3.4)	2.0 (1.2,3.1)	2.3 (1.5,3.4)	2.0 (1.2,3.1)	97.2 (96.0,98.1)
				**Res. Cor.**	0.3 (0.1,0.9)	20.9 (18.4,23.6)	20.5 (18.0,23.1)	0.0 (0.0,0.4)	0.0 (0.0,0.4)	79.7 (77.1,82.2)
**L**	**H**	**L**	**H**	**Pairwise G^2**	0.2 (0.0,0.7)	4.9 (3.6,6.4)	7.1 (5.6,8.9)	0.0 (0.0,0.4)	0.0 (0.0,0.4)	54.5 (51.4,57.6)
				**Pairwise χ^2**	0.1 (0.0,0.6)	5.4 (4.1,7.0)	8.4 (6.8,10.3)	0.0 (0.0,0.4)	0.0 (0.0,0.4)	52.0 (48.9,55.1)
**Sensitivity: L L L L**										
**Specificity**				**Tool**	**T_1&T_2**	**T_1&T_3**	**T_1&T_4**	**T_2&T_3**	**T_2&T_4**	**T_3&T_4**
				**Res. Cor.**	0.0 (0.0,0.4)	1.4 (0.7,2.3)	1.3 (0.7,2.2)	0.6 (0.2,1.3)	1.3 (0.7,2.2)	76.3 (73.4,78.9)
**H**	**H**	**L**	**L**	**Pairwise G^2**	0.0 (0.0,0.4)	0.0 (0.0,0.4)	0.0 (0.0,0.4)	0.0 (0.0,0.4)	0.0 (0.0,0.4)	8.8 (7.1,10.8)
				**Pairwise χ^2**	0.0 (0.0,0.4)	0.0 (0.0,0.4)	0.0 (0.0,0.4)	0.0 (0.0,0.4)	0.0 (0.0,0.4)	8.8 (7.1,10.8)
				**Res. Cor.**	0.0 (0.0,0.4)	0.0 (0.0,0.4)	1.4 (0.8,2.4)	0.0 (0.0,0.4)	1.1 (0.6,2.0)	76.2 (73.4,78.8)
**H**	**H**	**H**	**L**	**Pairwise G^2**	0.0 (0.0,0.4)	0.0 (0.0,0.4)	0.0 (0.0,0.4)	0.0 (0.0,0.4)	0.0 (0.0,0.4)	25.1 (22.4,27.9)
				**Pairwise χ^2**	0.0 (0.0,0.4)	0.0 (0.0,0.4)	0.0 (0.0,0.4)	0.0 (0.0,0.4)	0.0 (0.0,0.4)	24.8 (22.2,27.7)
				**Res. Cor.**	0.0 (0.0,0.4)	0.0 (0.0,0.4)	0.0 (0.0,0.4)	0.0 (0.0,0.4)	0.0 (0.0,0.4)	99.5 (98.8,99.8)
**H**	**H**	**H**	**H**	**Pairwise G^2**	0.0 (0.0,0.4)	0.0 (0.0,0.4)	0.0 (0.0,0.4)	0.0 (0.0,0.4)	0.0 (0.0,0.4)	94.9 (93.3,96.2)
				**Pairwise χ^2**	0.0 (0.0,0.4)	0.0 (0.0,0.4)	0.0 (0.0,0.4)	0.0 (0.0,0.4)	0.0 (0.0,0.4)	94.8 (93.2,96.1)
				**Res. Cor.**	0.0 (0.0,0.5)	21.8 (18.7,25.1)	19.7 (16.8,22.9)	19.9 (16.9,23.1)	19.7 (16.8,22.9)	80.1 (76.9,83.1)
**L**	**L**	**L**	**L**	**Pairwise G^2**	0.0 (0.0,0.5)	1.3 (0.6,2.5)	1.3 (0.6,2.5)	0.1 (0.0,0.8)	2.1 (1.1,3.5)	9.1 (7.0,11.5)
				**Pairwise χ^2**	0.0 (0.0,0.5)	1.3 (0.6,2.5)	1.3 (0.6,2.5)	0.1 (0.0,0.8)	2.1 (1.1,3.5)	9.1 (7.0,11.5)
				**Res. Cor.**	0.0 (0.0,0.4)	25.8 (23.0,28.6)	26.4 (23.6,29.3)	25.1 (22.4,28.0)	22.9 (20.3,25.7)	88.6 (86.4,90.5)
**L**	**L**	**H**	**L**	**Pairwise G^2**	0.0 (0.0,0.4)	9.4 (7.7,11.5)	2.4 (1.5,3.6)	8.1 (6.5,10.0)	2.4 (1.5,3.6)	42.5 (39.3,45.7)
				**Pairwise χ^2**	0.0 (0.0,0.4)	9.7 (7.9,11.7)	2.5 (1.6,3.7)	8.7 (7.0,10.7)	2.4 (1.5,3.6)	42.3 (39.1,45.5)

**L**	**L**	**H**	**H**	**Res. Cor.**	0.0 (0.0,0.4)	47.1 (43.9,50.2)	46.8 (43.6,49.9)	43.1 (40.0,46.2)	45.3 (42.1,48.4)	100.0 (99.6,100.0)
				**Pairwise G^2**	0.1 (0.0,0.6)	21.6 (19.1,24.3)	19.4 (17.0,22.0)	19.3 (16.9,21.9)	20.4 (18.0,23.1)	99.5 (98.8,99.8)
				**Pairwise χ^2**	0.1 (0.0,0.6)	22.0 (19.5,24.7)	20.2 (17.8,22.9)	20.3 (17.9,23.0)	21.3 (18.8,24.0)	99.5 (98.8,99.8)
**L**	**H**	**L**	**H**	**Res. Cor.**	0.0 (0.0,0.4)	32.3 (29.4,35.3)	86.0 (83.7,88.1)	1.4 (0.8,2.3)	0.0 (0.0,0.4)	80.3 (77.7,82.7)
				**Pairwise G^2**	0.0 (0.0,0.4)	5.3 (4.0,6.9)	60.4 (57.3,63.5)	0.0 (0.0,0.4)	0.0 (0.0,0.4)	32.0 (29.1,35.0)
				**Pairwise χ^2**	0.0 (0.0,0.4)	5.5 (4.2,7.1)	61.7 (58.6,64.8)	0.0 (0.0,0.4)	0.0 (0.0,0.4)	32.0 (29.1,35.0)
**Sensitivity: L L H L**										
**Specificity**				**Tool**	**T_1&T_2**	**T_1&T_3**	**T_1&T_4**	**T_2&T_3**	**T_2&T_4**	**T_3&T_4**
**H**	**H**	**L**	**L**	**Res. Cor.**	0.0 (0.0,0.4)	0.2 (0.0,0.7)	1.4 (0.8,2.3)	0.3 (0.1,0.9)	1.6 (0.9,2.6)	75.1 (72.3,77.7)
				**Pairwise G^2**	0.0 (0.0,0.4)	0.0 (0.0,0.4)	0.0 (0.0,0.4)	0.1 (0.0,0.6)	0.0 (0.0,0.4)	24.1 (21.5,26.9)
				**Pairwise χ^2**	0.0 (0.0,0.4)	0.0 (0.0,0.4)	0.0 (0.0,0.4)	0.1 (0.0,0.6)	0.0 (0.0,0.4)	24.0 (21.4,26.8)
**H**	**H**	**H**	**L**	**Res. Cor.**	0.0 (0.0,0.4)	0.0 (0.0,0.4)	0.8 (0.3,1.6)	0.1 (0.0,0.6)	0.5 (0.2,1.2)	90.8 (88.8,92.5)
				**Pairwise G^2**	0.0 (0.0,0.4)	0.0 (0.0,0.4)	0.0 (0.0,0.4)	0.0 (0.0,0.4)	0.0 (0.0,0.4)	59.2 (56.1,62.3)
				**Pairwise χ^2**	0.0 (0.0,0.4)	0.0 (0.0,0.4)	0.0 (0.0,0.4)	0.0 (0.0,0.4)	0.0 (0.0,0.4)	59.0 (55.9,62.1)
**H**	**H**	**H**	**H**	**Res. Cor.**	0.0 (0.0,0.4)	0.2 (0.0,0.7)	0.0 (0.0,0.4)	0.1 (0.0,0.6)	0.0 (0.0,0.4)	100.0 (99.6,100.0)
				**Pairwise G^2**	0.0 (0.0,0.4)	0.0 (0.0,0.4)	0.0 (0.0,0.4)	0.0 (0.0,0.4)	0.0 (0.0,0.4)	100.0 (99.6,100.0)
				**Pairwise χ^2**	0.0 (0.0,0.4)	0.0 (0.0,0.4)	0.0 (0.0,0.4)	0.0 (0.0,0.4)	0.0 (0.0,0.4)	100.0 (99.6,100.0)
**L**	**L**	**L**	**L**	**Res. Cor.**	0.0 (0.0,0.4)	38.5 (35.4,41.6)	22.3 (19.7,25.1)	43.3 (40.2,46.5)	23.7 (21.1,26.5)	89.5 (87.4,91.3)
				**Pairwise G^2**	0.0 (0.0,0.4)	19.7 (17.2,22.3)	2.0 (1.3,3.1)	21.4 (18.9,24.1)	2.7 (1.7,3.9)	42.0 (38.8,45.1)
				**Pairwise χ^2**	0.0 (0.0,0.4)	19.9 (17.4,22.5)	2.0 (1.3,3.1)	22.1 (19.5,24.8)	2.7 (1.7,3.9)	42.0 (38.8,45.1)
**L**	**L**	**H**	**L**	**Res. Cor.**	0.0 (0.0,0.4)	87.5 (85.3,89.5)	28.2 (25.4,31.1)	87.2 (84.9,89.2)	25.9 (23.2,28.7)	97.3 (96.1,98.2)
				**Pairwise G^2**	0.0 (0.0,0.4)	69.5 (66.6,72.4)	4.6 (3.4,6.1)	71.3 (68.4,74.1)	3.0 (2.0,4.3)	77.6 (74.8,80.1)
				**Pairwise χ^2**	0.0 (0.0,0.4)	70.5 (67.6,73.4)	4.6 (3.4,6.1)	71.6 (68.7,74.4)	3.0 (2.0,4.3)	77.6 (74.8,80.1)
**L**	**L**	**H**	**H**	**Res. Cor.**	3.6 (2.5,5.0)	96.3 (94.9,97.4)	72.4 (69.5,75.2)	95.0 (93.4,96.3)	71.0 (68.1,73.9)	99.7 (99.1,99.9)
				**Pairwise G^2**	6.1 (4.6,7.7)	88.0 (85.8,90.0)	43.3 (40.2,46.5)	84.5 (82.1,86.7)	42.4 (39.3,45.6)	99.7 (99.1,99.9)
				**Pairwise χ^2**	6.1 (4.6,7.7)	88.5 (86.3,90.4)	44.6 (41.4,47.7)	85.0 (82.6,87.2)	42.9 (39.8,46.1)	99.7 (99.1,99.9)
**L**	**H**	**L**	**H**	**Res. Cor.**	0.0 (0.0,0.4)	88.1 (85.9,90.0)	86.8 (84.5,88.8)	0.1 (0.0,0.6)	0.0 (0.0,0.4)	99.8 (99.3,100.0)
				**Pairwise G^2**	0.0 (0.0,0.4)	60.4 (57.3,63.4)	60.7 (57.6,63.7)	0.0 (0.0,0.4)	0.0 (0.0,0.4)	98.9 (98.0,99.4)
				**Pairwise χ^2**	0.0 (0.0,0.4)	61.3 (58.2,64.3)	61.8 (58.7,64.8)	0.1 (0.0,0.6)	0.0 (0.0,0.4)	98.8 (97.9,99.4)
**Sensitivity: L L H H**										
**Specificity**				**Tool**	**T_1&T_2**	**T_1&T_3**	**T_1&T_4**	**T_2&T_3**	**T_2&T_4**	**T_3&T_4**
**H**	**H**	**L**	**L**	**Res. Cor.**	0.0 (0.0,0.4)	0.6 (0.2,1.3)	0.6 (0.2,1.3)	0.5 (0.2,1.2)	0.8 (0.3,1.6)	99.9 (99.4,100.0)
				**Pairwise G^2**	0.0 (0.0,0.4)	0.2 (0.0,0.7)	0.2 (0.0,0.7)	0.2 (0.0,0.7)	0.4 (0.1,1.0)	96.6 (95.3,97.6)
				**Pairwise χ^2**	0.0 (0.0,0.4)	0.2 (0.0,0.7)	0.3 (0.1,0.9)	0.2 (0.0,0.7)	0.5 (0.2,1.2)	96.6 (95.3,97.6)
**H**	**H**	**H**	**L**	**Res. Cor.**	0.0 (0.0,0.4)	0.2 (0.0,0.7)	1.2 (0.6,2.1)	0.3 (0.1,0.9)	1.1 (0.6,2.0)	100.0 (99.6,100.0)
				**Pairwise G^2**	0.0 (0.0,0.4)	0.1 (0.0,0.6)	0.3 (0.1,0.9)	0.1 (0.0,0.6)	0.1 (0.0,0.6)	100.0 (99.6,100.0)
				**Pairwise χ^2**	0.0 (0.0,0.4)	0.1 (0.0,0.6)	0.5 (0.2,1.2)	0.1 (0.0,0.6)	0.1 (0.0,0.6)	100.0 (99.6,100.0)
**H**	**H**	**H**	**H**	**Res. Cor.**	0.2 (0.0,0.7)	0.1 (0.0,0.6)	0.4 (0.1,1.0)	0.0 (0.0,0.4)	0.0 (0.0,0.4)	99.8 (99.3,100.0)
				**Pairwise G^2**	0.2 (0.0,0.7)	0.0 (0.0,0.4)	0.0 (0.0,0.4)	0.0 (0.0,0.4)	0.0 (0.0,0.4)	99.8 (99.3,100.0)
				**Pairwise χ^2**	0.2 (0.0,0.7)	0.0 (0.0,0.4)	0.0 (0.0,0.4)	0.0 (0.0,0.4)	0.0 (0.0,0.4)	99.8 (99.3,100.0)
**L**	**L**	**L**	**L**	**Res. Cor.**	0.3 (0.1,0.9)	64.4 (61.3,67.3)	63.6 (60.5,66.6)	62.3 (59.2,65.3)	63.6 (60.5,66.6)	100.0 (99.6,100.0)

				**Pairwise G^2**	0.2 (0.0,0.7)	39.2 (36.2,42.3)	38.0 (35.0,41.1)	35.6 (32.7,38.7)	35.3 (32.4,38.4)	99.8 (99.3,100.0)
				**Pairwise χ^2**	0.2 (0.0,0.7)	40.0 (37.0,43.2)	38.8 (35.8,41.9)	37.2 (34.2,40.3)	36.1 (33.2,39.2)	99.8 (99.3,100.0)
**L**	**L**	**H**	**L**	**Res. Cor.**	15.7 (13.5,18.1)	97.1 (95.9,98.1)	83.9 (81.5,86.2)	96.7 (95.4,97.8)	83.4 (80.9,85.7)	99.7 (99.1,99.9)
				**Pairwise G^2**	17.5 (15.2,20.0)	89.0 (86.9,90.9)	58.7 (55.5,61.8)	89.7 (87.6,91.5)	59.5 (56.3,62.6)	99.7 (99.1,99.9)
				**Pairwise χ^2**	16.6 (14.3,19.1)	89.3 (87.2,91.2)	60.0 (56.8,63.1)	90.3 (88.3,92.1)	60.7 (57.6,63.8)	99.7 (99.1,99.9)
**L**	**L**	**H**	**H**	**Res. Cor.**	98.3 (97.0,99.2)	13.1 (10.6,15.9)	13.5 (11.0,16.4)	13.4 (10.9,16.2)	13.4 (10.9,16.2)	13.4 (10.9,16.2)
				**Pairwise G^2**	96.3 (94.6,97.6)	12.0 (9.6,14.7)	12.0 (9.6,14.7)	12.6 (10.2,15.4)	12.6 (10.2,15.4)	13.4 (10.9,16.2)
				**Pairwise χ^2**	96.2 (94.4,97.5)	12.0 (9.6,14.7)	12.0 (9.6,14.7)	12.6 (10.2,15.4)	12.6 (10.2,15.4)	13.4 (10.9,16.2)
**L**	**H**	**L**	**H**	**Res. Cor.**	0.1 (0.0,0.6)	88.2 (86.0,90.1)	99.8 (99.3,100.0)	0.4 (0.1,1.0)	0.0 (0.0,0.4)	100.0 (99.6,100.0)
				**Pairwise G^2**	0.1 (0.0,0.6)	64.1 (61.0,67.1)	98.9 (98.0,99.4)	0.0 (0.0,0.4)	0.0 (0.0,0.4)	100.0 (99.6,100.0)
				**Pairwise χ^2**	0.1 (0.0,0.6)	65.2 (62.2,68.2)	98.9 (98.0,99.4)	0.1 (0.0,0.6)	0.0 (0.0,0.4)	100.0 (99.6,100.0)

Table iv.4 Mean absolute biases (x10^{-2}) of posterior medians of each parameter obtained from the conditional independence model

**Sensitivity: H H L L**												
**Specificity**				π	Se_1	Se_2	Se_3	Se_4	Sp_1	Sp_2	Sp_3	Sp_4
**H**	**H**	**L**	**L**	-2.1 (-2.2,-1.9)	6.7 (6.6,6.8)	6.7 (6.6,6.8)	-0.6 (-0.8,-0.4)	-0.5 (-0.7,-0.3)	2.6 (2.5,2.8)	2.7 (2.6,2.9)	-1.6 (-1.8,-1.4)	-1.6 (-1.7,-1.4)
**H**	**H**	**H**	**L**	-4.1 (-4.2,-3.9)	8.4 (8.4,8.5)	8.4 (8.3,8.4)	-0.5 (-0.7,-0.3)	-0.1 (-0.3,0.2)	1.1 (1.0,1.3)	0.9 (0.8,1.1)	-4.4 (-4.5,-4.3)	-1.7 (-1.9,-1.5)
**H**	**H**	**H**	**H**	-4.4 (-4.5,-4.3)	8.7 (8.7,8.7)	8.7 (8.7,8.7)	-0.4 (-0.6,-0.2)	-0.3 (-0.5,-0.1)	0.7 (0.6,0.8)	0.6 (0.5,0.7)	-4.6 (-4.7,-4.5)	-4.5 (-4.6,-4.4)
**L**	**L**	**L**	**L**	3.5 (3.2,3.7)	4.9 (4.8,5.1)	4.9 (4.7,5.1)	-1.9 (-2.2,-1.7)	-2.0 (-2.2,-1.8)	9.1 (8.8,9.4)	9.1 (8.7,9.4)	-0.9 (-1.2,-0.7)	-1.2 (-1.4,-1.0)
**L**	**L**	**H**	**L**	-2.5 (-2.7,-2.3)	8.3 (8.2,8.3)	8.2 (8.2,8.3)	-2.6 (-2.9,-2.4)	-0.8 (-1.0,-0.6)	5.2 (5.0,5.4)	5.1 (4.9,5.4)	-4.9 (-5.1,-4.8)	-2.0 (-2.2,-1.8)
**L**	**L**	**H**	**H**	-3.7 (-3.8,-3.5)	8.8 (8.7,8.8)	8.8 (8.8,8.8)	-1.5 (-1.7,-1.3)	-1.8 (-2.0,-1.5)	4.0 (3.7,4.2)	4.1 (3.9,4.4)	-5.2 (-5.3,-5.0)	-5.1 (-5.2,-5.0)
**L**	**H**	**L**	**H**	-3.6 (-3.8,-3.5)	8.4 (8.3,8.4)	8.2 (8.2,8.3)	-0.4 (-0.6,-0.2)	-1.0 (-1.2,-0.8)	3.7 (3.6,3.9)	1.4 (1.3,1.6)	-1.9 (-2.1,-1.7)	-4.5 (-4.6,-4.4)
**Sensitivity: H H H L**												
**Specificity**				π	Se_1	Se_2	Se_3	Se_4	Sp_1	Sp_2	Sp_3	Sp_4
**H**	**H**	**L**	**L**	-3.8 (-4.0,-3.7)	8.1 (8.0,8.2)	8.1 (8.0,8.2)	-0.5 (-0.7,-0.4)	-0.1 (-0.4,0.1)	1.3 (1.2,1.4)	1.2 (1.1,1.4)	-3.8 (-4.0,-3.6)	-1.7 (-1.9,-1.5)
**H**	**H**	**H**	**L**	-4.8 (-4.9,-4.6)	8.9 (8.8,8.9)	8.9 (8.9,8.9)	-0.4 (-0.6,-0.3)	0.0 (-0.2,0.2)	0.6 (0.5,0.7)	0.5 (0.4,0.6)	-7.2 (-7.3,-7.0)	-1.9 (-2.1,-1.8)
**H**	**H**	**H**	**H**	-4.6 (-4.7,-4.4)	8.9 (8.9,8.9)	8.8 (8.8,8.9)	-0.3 (-0.5,-0.2)	0.0 (-0.2,0.2)	0.5 (0.3,0.6)	0.5 (0.4,0.7)	-7.2 (-7.3,-7.0)	-4.4 (-4.5,-4.2)
**L**	**L**	**L**	**L**	-1.4 (-1.6,-1.2)	7.6 (7.5,7.7)	7.6 (7.5,7.7)	-2.7 (-2.9,-2.5)	-0.9 (-1.2,-0.7)	5.8 (5.5,6.0)	5.8 (5.6,6.1)	-4.1 (-4.4,-3.9)	-1.8 (-2.0,-1.6)
**L**	**L**	**H**	**L**	-3.6 (-3.8,-3.5)	9.1 (9.0,9.1)	9.0 (9.0,9.1)	-3.3 (-3.5,-3.1)	-0.3 (-0.5,-0.1)	4.3 (4.1,4.5)	4.2 (4.0,4.4)	-8.4 (-8.6,-8.2)	-1.8 (-2.0,-1.6)
**L**	**L**	**H**	**H**	-5.2 (-5.4,-5.0)	9.1 (9.0,9.1)	9.1 (9.0,9.1)	-0.8 (-1.0,-0.6)	-0.3 (-0.5,0.0)	2.8 (2.6,3.0)	2.7 (2.5,2.9)	-8.4 (-8.6,-8.2)	-5.0 (-5.2,-4.9)
**L**	**H**	**L**	**H**	-4.2 (-4.3,-4.0)	8.6 (8.5,8.6)	8.6 (8.5,8.6)	-0.6 (-0.8,-0.5)	-0.6 (-0.8,-0.4)	3.5 (3.3,3.7)	1.0 (0.8,1.1)	-4.4 (-4.6,-4.3)	-4.6 (-4.7,-4.4)
**Sensitivity: H H H H**												
**Specificity**				π	Se_1	Se_2	Se_3	Se_4	Sp_1	Sp_2	Sp_3	Sp_4
**H**	**H**	**L**	**L**	-4.2 (-4.4,-4.1)	8.5 (8.5,8.6)	8.5 (8.4,8.6)	-0.3 (-0.5,-0.2)	-0.3 (-0.5,-0.2)	0.8 (0.7,0.9)	0.8 (0.7,0.9)	-4.3 (-4.5,-4.1)	-4.3 (-4.5,-4.1)
**H**	**H**	**H**	**L**	-4.7 (-4.8,-4.6)	8.9 (8.9,.8.9)	8.9 (8.8,8.9)	-0.4 (-0.6,-0.3)	-0.2 (-0.4,.-0.1)	0.6 (0.4,0.7)	0.5 (0.4,0.6)	-7.2 (-7.4,-7.1)	-4.5 (-4.7,-4.3)
**H**	**H**	**H**	**H**	-4.7 (-4.8,-4.5)	8.8 (8.8,8.9)	8.8 (8.8,8.9)	-0.3 (-0.4,-0.1)	-0.3 (-0.4,-0.1)	0.6 (0.5,0.7)	0.6 (0.5,0.7)	-7.1 (-7.3,-7.0)	-7.2 (-7.3,-7.0)
**L**	**L**	**L**	**L**	-3.4 (-3.6,-3.2)	8.4 (8.4,8.5)	8.3 (8.3,8.4)	-1.2 (-1.4,-1.0)	-1.2 (-1.4,-1.0)	3.9 (3.7,4.2)	4.0 (3.8,4.2)	-4.6 (-4.8,-4.4)	-4.3 (-4.5,-4.1)
**L**	**L**	**H**	**L**	-5.0 (-5.2,-4.8)	9.1 (9.0,9.1)	9.1 (9.0,9.1)	-0.9 (-1.1,-0.8)	0.0 (-0.1,0.2)	2.7 (2.5,2.9)	2.7 (2.5,2.9)	-8.3 (-8.4,-8.1)	-4.8 (-5.0,-4.6)

**L**	**L**	**H**	**H**	-6.0 (-6.2,-5.9)	9.1 (9.0,9.1)	9.0 (9.0,9.1)	0.5 (0.4,0.7)	0.7 (0.6,0.9)	1.6 (1.4,1.8)	1.8 (1.6,2.0)	-8.1 (-8.3,-8.0)	-8.1 (-8.3,-7.9)
**L**	**H**	**L**	**H**	-4.9 (-5.0,-4.7)	8.9 (8.8,8.9)	9.0 (9.0,9.1)	-0.2 (-0.4,-0.1)	-0.5 (-0.7,-0.4)	2.8 (2.6,3.0)	0.4 (0.3,0.5)	-4.4 (-4.6,-4.2)	-7.4 (-7.5,-7.2)
**Sensitivity: L L L L**												
**Specificity**				π	Se_1	Se_2	Se_3	Se_4	Sp_1	Sp_2	Sp_3	Sp_4
**H**	**H**	**L**	**L**	-17.2 (-17.3,-17.0)	34.2 (34.0,34.3)	34.2 (34.0,34.4)	-0.8 (-1.0,-0.5)	-0.8 (-1.0,-0.6)	3.6 (3.5,3.8)	3.6 (3.5,3.7)	-5.7 (-5.8,-5.5)	-5.8 (-5.9,-5.6)
**H**	**H**	**H**	**L**	-19.3 (-19.4,-19.2)	37.1 (37.0,37.2)	37.0 (36.9,37.1)	-1.2 (-1.5,-1.0)	-0.1 (-0.4,0.1)	2.2 (2.1,2.4)	2.2 (2.1,2.3)	-14.7 (-14.9,-14.6)	-5.9 (-6.1,-5.8)
**H**	**H**	**H**	**H**	-19.8 (-19.9,-19.7)	37.7 (37.6,37.8)	37.6 (37.5,37.7)	-0.6 (-0.9,-0.3)	-0.8 (-1.0,-0.5)	2.0 (1.9,2.1)	1.9 (1.8,2.0)	-14.7 (-14.9,-14.5)	-14.6 (-14.8,-14.5)
**L**	**L**	**L**	**L**	-10.0 (-10.3,-9.7)	32.6 (32.4,32.9)	32.5 (32.2,32.7)	-3.9 (-4.2,-3.6)	-3.8 (-4.0,-3.5)	17.6 (17.4,17.9)	17.4 (17.1,17.7)	-6.2 (-6.4,-5.9)	-6.2 (-6.4,-6.0)
**L**	**L**	**H**	**L**	-16.0 (-16.2,-15.8)	37.5 (37.4,37.6)	37.5 (37.4,37.6)	-8.8 (-9.1,-8.5)	-2.8 (-3.0,-2.5)	14.2 (14.0,14.4)	14.4 (14.2,14.6)	-16.7 (-16.8,-16.5)	-6.4 (-6.6,-6.2)
**L**	**L**	**H**	**H**	-17.1 (-17.3,-17.0)	38.4 (38.4,38.4)	38.4 (38.4,38.5)	-7.2 (-7.5,-6.9)	-7.2 (-7.5,-6.9)	13.5 (13.3,13.7)	13.4 (13.2,13.6)	-16.5 (-16.7,-16.4)	-16.7 (-16.8,-16.5)
**L**	**H**	**L**	**H**	-19.1 (-19.2,-19.0)	36.7 (36.6,36.8)	37.9 (37.9,37.9)	-0.7 (-1.0,-0.5)	-2.6 (-2.8,-2.3)	10.8 (10.6,11.0)	3.0 (2.9,3.1)	-5.9 (-6.1,-5.8)	-15.1 (-15.3,-15.0)
**Sensitivity: L L H L**												
**Specificity**				π	Se_1	Se_2	Se_3	Se_4	Sp_1	Sp_2	Sp_3	Sp_4
**H**	**H**	**L**	**L**	-19.2 (-19.3,-19.0)	36.9 (36.8,37.0)	36.8 (36.7,36.9)	-1.1 (-1.3,-1.0)	-0.4 (-0.7,-0.2)	2.3 (2.2,2.4)	2.2 (2.1,2.3)	-14.3 (-14.5,-14.1)	-5.8 (-5.9,-5.6)
**H**	**H**	**H**	**L**	-20.3 (-20.4,-20.2)	38.2 (38.1,38.2)	38.2 (38.1,38.2)	-0.9 (-1.1,-0.8)	0.2 (0.0,0.4)	1.6 (1.5,1.7)	1.6 (1.5,1.7)	-23.6 (-23.8,-23.5)	-6.0 (-6.1,-5.8)
**H**	**H**	**H**	**H**	-20.1 (-20.2,-19.9)	37.9 (37.8,38.0)	37.8 (37.7,37.9)	-0.9 (-1.1,-0.7)	0.1 (-0.2,0.3)	1.7 (1.6,1.8)	1.7 (1.6,1.8)	-23.3 (-23.5,-23.1)	-14.5 (-14.7,-14.4)
**L**	**L**	**L**	**L**	-15.7 (-15.9,-15.6)	37.2 (37.1,37.3)	37.1 (37.1,37.2)	-8.3 (-8.5,-8.1)	-2.6 (-2.8,-2.4)	14.6 (14.4,14.8)	14.4 (14.2,14.5)	-16.5 (-16.6,-16.3)	-6.3 (-6.5,-6.1)
**L**	**L**	**H**	**L**	-17.4 (-17.6,-17.3)	38.8 (38.7,38.8)	38.8 (38.8,38.8)	-12.3 (-12.5,-12.0)	-1.9 (-2.1,-1.6)	13.4 (13.2,13.6)	13.3 (13.1,13.5)	-26.6 (-26.8,-26.5)	-6.2 (-6.4,-6.0)
**L**	**L**	**H**	**H**	-19.0 (-19.2,-18.8)	38.8 (38.6,38.9)	38.8 (38.6,38.9)	-8.2 (-8.5,-7.8)	-3.8 (-4.1,-3.5)	11.6 (11.4,11.8)	11.7 (11.5,11.9)	-26.0 (-26.2,-25.9)	-15.7 (-15.9,-15.6)
**L**	**H**	**L**	**H**	-19.6 (-19.7,-19.4)	37.2 (37.1,37.3)	38.4 (38.4,38.5)	-1.7 (-1.9,-1.5)	-1.7 (-2.0,-1.4)	10.6 (10.4,10.7)	2.6 (2.5,2.7)	-14.8 (-15.0,-14.7)	-15.0 (-15.1,-14.8)
**Sensitivity: L L H H**												
**Specificity**				π	Se_1	Se_2	Se_3	Se_4	Sp_1	Sp_2	Sp_3	Sp_4
**H**	**H**	**L**	**L**	-19.8 (-19.9,-19.7)	37.5 (37.4,37.5)	37.5 (37.4,37.6)	-0.7 (-0.9,-0.6)	-0.8 (-1.0,-0.7)	2.0 (1.9,2.1)	2.0 (1.9,2.1)	-14.6 (-14.7,-14.4)	-14.5 (-14.7,-14.3)
**H**	**H**	**H**	**L**	-20.1 (-20.2,-20.0)	37.9 (37.8,38.0)	37.8 (37.8,37.9)	-0.8 (-1.0,-0.7)	-0.6 (-0.8,-0.5)	1.7 (1.6,1.8)	1.7 (1.6,1.8)	-23.4 (-23.5,-23.2)	-14.6 (-14.8,-14.5)
**H**	**H**	**H**	**H**	-20.0 (-20.1,-19.8)	37.5 (37.4,37.7)	37.6 (37.4,37.7)	-0.5 (-0.6,-0.3)	-0.3 (-0.5,-0.1)	1.7 (1.6,1.8)	1.7 (1.6,1.8)	-23.0 (-23.2,-22.8)	-23.0 (-23.2,-22.8)

**L**	**L**	**L**	**L**	-17.7 (-17.8,-17.5)	38.2 (38.2,38.3)	38.3 (38.2,38.3)	-5.9 (-6.1,-5.6)	-6.0 (-6.2,-5.8)	12.9 (12.7,13.0)	12.9 (12.7,13.1)	-16.0 (-16.1,-15.8)	-15.9 (-16.1,-15.7)
**L**	**L**	**H**	**L**	-19.7 (-19.9,-19.5)	38.8 (38.6,38.9)	38.8 (38.6,38.9)	-6.7 (-7.0,-6.4)	-2.2 (-2.5,-2.0)	10.9 (10.7,11.2)	10.9 (10.7,11.1)	-25.8 (-26.0,-25.6)	-15.1 (-15.3,-14.9)
**L**	**L**	**H**	**H**	-3.0 (-3.6,-2.4)	5.6 (4.6,6.7)	5.6 (4.6,6.7)	0.5 (0.2,0.7)	0.3 (0.1,0.6)	1.5 (1.2,1.8)	1.3 (1.0,1.6)	-3.3 (-4.0,-2.7)	-3.6 (-4.2,-2.9)
**L**	**H**	**L**	**H**	-20.2 (-20.3,-20.0)	37.6 (37.5,37.7)	39.0 (38.9,39.0)	-0.7 (-0.9,-0.5)	-1.9 (-2.1,-1.7)	10.0 (9.9,10.2)	2.0 (1.8,2.1)	-14.9 (-15.1,-14.7)	-23.9 (-24.1,-23.8)

Table iv.5 Coverages of the 95% CrIs for each model parameter

**Sensitivity: H H L L**												
**Specificity**				π	Se_1	Se_2	Se_3	Se_4	Sp_1	Sp_2	Sp_3	Sp_4
**H**	**H**	**L**	**L**	93.0 (91.5,94.6)	38.3 (35.3,41.4)	39.1 (36.0,42.1)	94.3 (92.8,95.7)	94.5 (93.0,95.9)	88.2 (86.2,90.3)	87.3 (85.2,89.4)	91.6 (89.9,93.4)	92.8 (91.2,94.5)
**H**	**H**	**H**	**L**	62.6 (59.6,65.6)	1.0 (0.4,1.6)	0.5 (0.1,0.9)	95.0 (93.6,96.4)	94.8 (93.4,96.2)	92.9 (91.3,94.5)	94.6 (93.2,96.0)	43.7 (40.6,46.8)	90.8 (89.0,92.6)
**H**	**H**	**H**	**H**	53.9 (50.8,56.9)	0.0 (0.0,0.0)	0.0 (0.0,0.0)	95.6 (94.3,96.9)	95.4 (94.1,96.7)	95.6 (94.3,96.9)	94.3 (92.9,95.7)	37.7 (34.7,40.7)	38.3 (35.3,41.4)
**L**	**L**	**L**	**L**	96.2 (94.9,97.5)	90.6 (8.7,92.6)	91.4 (89.5,93.3)	92.1 (90.3,93.9)	91.0 (89.1,93.0)	58.1 (54.7,61.5)	58.6 (55.2,61.9)	96.1 (94.8,97.4)	95.0 (93.5,96.5)
**L**	**L**	**H**	**L**	93.4 (91.9,94.9)	4.2 (3.0,5.4)	5.1 (3.7,6.5)	88.9 (86.9,90.8)	94.6 (93.2,96.0)	73.0 (71.0,76.5)	74.2 (71.5,76.9)	43.2 (40.2,46.3)	92.6 (91.0,94.2)
**L**	**L**	**H**	**H**	79.3 (76.8,81.8)	0.1 (0.0,0.3)	0.1 (0.0,0.3)	93.4 (91.9,94.9)	91.6 (89.9,93.3)	80.5 (78.0,83.0)	80.2 (77.7,82.7)	34.7 (31.7,37.7)	36.1 (33.1,39.1)
**L**	**H**	**L**	**H**	76.1 (73.4,78.7)	0.8 (0.2,1.4)	5.3 (3.9,6.7)	95.3 (94.0,96.6)	93.6 (92.1,95.1)	79.8 (77.3,82.3)	93.8 (92.3,95.3)	92.2 (90.5,93.9)	43.5 (40.5,46.6)
**Sensitivity: H H H L**												
**Specificity**				π	Se_1	Se_2	Se_3	Se_4	Sp_1	Sp_2	Sp_3	Sp_4
**H**	**H**	**L**	**L**	67.0 (64.1,69.9)	1.9 (1.1,2.7)	2.2 (1.3,3.1)	94.1 (92.6,95.6)	94.8 (93.4,96.2)	93.0 (91.4,94.6)	94.2 (92.8,95.6)	76.8 (74.2,79.4)	91.9 (90.2,93.6)
**H**	**H**	**H**	**L**	44.6 (41.5,47.7)	0.0 (0.0,0.0)	0.0 (0.0,0.0)	93.4 (91.9,94.9)	94.0 (92.5,95.5)	94.5 (93.1,95.9)	95.0 (93.6,96.4)	7.1 (5.5,8.7)	90.9 (89.1,92.7)
**H**	**H**	**H**	**H**	47.5 (44.5,50.6)	0.0 (0.0,0.0)	0.0 (0.0,0.0)	93.9 (92.4,95.4)	95.8 (94.6,97.0)	94.9 (93.5,96.3)	94.8 (93.4,96.2)	7.3 (5.7,8.9)	41.8 (38.8,44.9)
**L**	**L**	**L**	**L**	96.3 (95.1,97.5)	15.9 (13.7,18.2)	14.2 (12.1,16.4)	83.9 (81.6,86.1)	93.6 (92.1,95.1)	71.2 (68.4,74.1)	69.3 (66.5,72.2)	79.7 (77.2,82.2)	91.5 (89.8,93.2)
**L**	**L**	**H**	**L**	79.2 (76.7,81.7)	0.1 (0.0,0.3)	0.0 (0.0,0.0)	85.5 (85.3,87.7)	94.5 (93.1,95.9)	80.5 (78.0,83.0)	80.3 (77.8,82.8)	5.7 (4.3,7.1)	93.1 (91.5,94.7)
**L**	**L**	**H**	**H**	52.5 (49.4,55.6)	0.0 (0.0,0.0)	0.0 (0.0,0.0)	94.6 (93.2,96.0)	94.3 (92.9,95.7)	88.0 (86.0,90.0)	89.2 (87.3,91.1)	5.1 (3.7,6.5)	35.4 (32.4,38.4)
**L**	**H**	**L**	**H**	63.9 (60.9,66.8)	0.1 (0.0,0.3)	0.9 (0.3,1.5)	93.8 (92.3,95.3)	94.0 (92.5,95.5)	80.3 (77.8,82.7)	95.0 (93.6,96.3)	71.5 (68.7,74.3)	41.0 (38.0,44.1)
**Sensitivity: H H H H**												
**Specificity**				π	Se_1	Se_2	Se_3	Se_4	Sp_1	Sp_2	Sp_3	Sp_4
**H**	**H**	**L**	**L**	57.8 (54.7,60.9)	0.2 (0.0,0.5)	0.0 (0.0,0.0)	95.0 (93.6,96.4)	94.8 (93.4,96.2)	93.5 (92.0,95.0)	93.7 (92.2,95.2)	72.1 (69.3,74.9)	72.8 (70.0,75.6)
**H**	**H**	**H**	**L**	45.2 (42.1,48.3)	0.0 (0.0,0.0)	0.0 (0.0,0.0)	94.8 (93.4,96.2)	95.7 (94.4,97.0)	94.0 (92.5,95.5)	94.2 (92.8,95.6)	8.0 (6.3,9.7)	67.0 (64.1,69.9)
**H**	**H**	**H**	**H**	43.7 (40.6,46.8)	0.0 (0.0,0.0)	0.0 (0.0,0.0)	94.8 (93.4,96.2)	95.3 (94.0,96.6)	95.3 (94.0,96.6)	94.8 (93.4,96.2)	6.9 (5.3,8.5)	6.7 (5.2,8.2)
**L**	**L**	**L**	**L**	83.3 (81.0,85.6)	1.2 (0.5,1.9)	1.8 (1.0,2.6)	91.9 (90.2,93.6)	93.5 (92.0,95.0)	81.4 (79.0,83.8)	82.4 (80.0,84.8)	74.1 (71.4,76.8)	77.2 (74.6,79.8)
**L**	**L**	**H**	**L**	55.1 (52.0,58.2)	0.0 (0.0,0.0)	0.0 (0.0,0.0)	94.9 (93.5,96.3)	95.1 (93.8,96.4)	87.9 (85.9,89.9)	88.4 (86.4,90.4)	5.5 (4.1,6.9)	68.0 (65.1,70.9)
**L**	**L**	**H**	**H**	31.0 (28.1,33.9)	0.2 (0.0,0.5)	0.1 (0.0,0.3)	95.0 (93.6,96.4)	94.4 (93.0,95.8)	92.6 (91.0,94.2)	90.1 (88.2,92.0)	5.8 (4.4,7.2)	5.6 (4.2,7.0)
**L**	**H**	**L**	**H**	46.6 (43.5,49.7)	0.0 (0.0,0.0)	0.0 (0.0,0.0)	96.2 (95.0,97.4)	95.1 (93.8,96.4)	85.2 (83.0,87.4)	95.0 (93.6,96.4)	70.0 (67.2,72.8)	6.9 (5.3,8.5)
**Sensitivity: L L L L**												
**Specificity**				π	Se_1	Se_2	Se_3	Se_4	Sp_1	Sp_2	Sp_3	Sp_4
**H**	**H**	**L**	**L**	0.0 (0.0,0.0)	0.0 (0.0,0.0)	0.0 (0.0,0.0)	95.5 (94.2,96.8)	95.1 (93.7,96.4)	69.0 (66.1,72.0)	69.5 (66.6,72.5)	44.9 (41.7,48.0)	42.9 (39.7,46.0)

**H**	**H**	**H**	**L**	0.0 (0.0,0.0)	0.0 (0.0,0.0)	0.0 (0.0,0.0)	93.0 (91.4,94.5)	95.3 (94.0,96.6)	80.0 (77.5,82.5)	80.9 (78.4,83.3)	0.0 (0.0,0.0)	39.7 (36.7,42.8)
**H**	**H**	**H**	**H**	0.0 (0.0,0.0)	0.0 (0.0,0.0)	0.0 (0.0,0.0)	93.8 (92.3,95.3)	94.6 (93.2,96.0)	81.8 (79.4,84.2)	81.1 (78.7,83.5)	0.0 (0.0,0.0)	0.0 (0.0,0.0)
**L**	**L**	**L**	**L**	84.5 (81.7,87.2)	0.0 (0.0,0.0)	0.0 (0.0,0.0)	83.3 (80.5,86.1)	83.1 (80.3,86.0)	0.3 (0.0,0.7)	0.3 (0.0,0.7)	44.8 (41.0,48.5)	44.5 (40.7,48.2)
**L**	**L**	**H**	**L**	2.3 (1.3,3.2)	0.0 (0.0,0.0)	0.0 (0.0,0.0)	53.4 (50.2,56.5)	91.1 (89.3,92.9)	0.8 (0.3,1.4)	0.1 (0.0,0.3)	0.0 (0.0,0.0)	35.5 (32.5,38.5)
**L**	**L**	**H**	**H**	0.0 (0.0,0.0)	0.0 (0.0,0.0)	0.0 (0.0,0.0)	65.0 (62.0,68.0)	64.4 (61.4,67.4)	1.4 (0.7,2.1)	1.1 (0.5,1.8)	0.0 (0.0,0.0)	0.0 (0.0,0.0)
**L**	**H**	**L**	**H**	0.0 (0.0,0.0)	0.0 (0.0,0.0)	0.0 (0.0,0.0)	94.9 (93.5,96.2)	90.5 (88.6,92.3)	2.0 (1.1,2.9)	75.3 (72.6,78.0)	40.6 (37.5,43.6)	0.0 (0.0,0.0)
**Sensitivity: L L H L**												
**Specificity**				π	Se_1	Se_2	Se_3	Se_4	Sp_1	Sp_2	Sp_3	Sp_4
**H**	**H**	**L**	**L**	0.0 (0.0,0.0)	0.0 (0.0,0.0)	0.0 (0.0,0.0)	93.5 (92.0,95.0)	93.9 (92.4,95.4)	79.2 (76.7,81.7)	81.4 (79.0,83.8)	0.0 (0.0,0.0)	40.4 (37.4,43.5)
**H**	**H**	**H**	**L**	0.0 (0.0,0.0)	0.0 (0.0,0.0)	0.0 (0.0,0.0)	93.7 (92.2,95.2)	96.2 (95.0,97.4)	86.6 (84.5,88.7)	84.8 (82.6,87.0)	0.0 (0.0,0.0)	39.0 (36.0,42.0)
**H**	**H**	**H**	**H**	0.0 (0.0,0.0)	0.0 (0.0,0.0)	0.0 (0.0,0.0)	95.1 (93.8,96.4)	95.6 (94.3,96.9)	84.1 (81.8,86.4)	82.5 (80.1,84.9)	0.0 (0.0,0.0)	0.0 (0.0,0.0)
**L**	**L**	**L**	**L**	2.1 (1.2,3.1)	0.0 (0.0,0.0)	0.0 (0.0,0.0)	37.8 (34.7,40.8)	90.0 (88.1,91.9)	0.0 (0.0,0.0)	0.8 (0.3,1.4)	0.0 (0.0,0.0)	37.7 (34.6,40.7)
**L**	**L**	**H**	**L**	0.0 (0.0,0.0)	0.0 (0.0,0.0)	0.0 (0.0,0.0)	19.6 (17.2,22.1)	92.0 (90.3,93.7)	0.4 (0.0,0.8)	0.8 (0.2,1.4)	0.0 (0.0,0.0)	36.8 (33.8,39.8)
**L**	**L**	**H**	**H**	0.3 (0.0,0.7)	0.3 (0.0,0.7)	0.3 (0.0,0.7)	55.9 (52.7,59.0)	81.9 (79.5,84.3)	3.9 (2.7,5.1)	4.6 (3.3,5.9)	0.3 (0.0,0.7)	0.3 (0.0,0.7)
**L**	**H**	**L**	**H**	0.0 (0.0,0.0)	0.0 (0.0,0.0)	0.0 (0.0,0.0)	92.1 (90.4,93.8)	92.8 (91.2,94.4)	2.4 (1.4,3.2)	78.4 (75.8,80.9)	0.1 (0.0,0.3)	0.0 (0.0,0.0)
**Sensitivity: L L H H**												
**Specificity**				π	Se_1	Se_2	Se_3	Se_4	Sp_1	Sp_2	Sp_3	Sp_4
**H**	**H**	**L**	**L**	0.0 (0.0,0.0)	0.0 (0.0,0.0)	0.0 (0.0,0.0)	94.0 (92.5,95.5)	94.4 (93.0,95.8)	81.7 (79.3,84.1)	81.8 (79.4,84.2)	0.1 (0.0,0.3)	0.0 (0.0,0.0)
**H**	**H**	**H**	**L**	0.0 (0.0,0.0)	0.0 (0.0,0.0)	0.0 (0.0,0.0)	94.4 (93.0,95.8)	93.5 (92.0,95.0)	85.0 (82.8,87.2)	85.1 (82.9,87.3)	0.0 (0.0,0.0)	0.2 (0.0,0.5)
**H**	**H**	**H**	**H**	0.2 (0.0,0.5)	0.1 (0.0,0.3)	0.2 (0.0,0.5)	94.6 (93.2,96.0)	93.9 (92.4,95.4)	83.7 (81.4,86.0)	81.4 (79.0,83.8)	0.2 (0.0,0.5)	0.2 (0.0,0.5)
**L**	**L**	**L**	**L**	0.0 (0.0,0.0)	0.0 (0.0,0.0)	0.0 (0.0,0.0)	63.2 (60.2,66.2)	60.3 (57.2,63.3)	1.2 (0.5,1.9)	1.4 (0.7,2.1)	0.0 (0.0,0.0)	0.1 (0.0,0.3)
**L**	**L**	**H**	**L**	0.3 (0.0,0.7)	0.3 (0.0,0.7)	0.2 (0.0,0.5)	67.8 (64.9,70.7)	88.3 (86.3,90.3)	5.8 (4.3,7.3)	5.8 (4.3,7.3)	0.3 (0.0,0.7)	0.3 (0.0,0.7)
**L**	**L**	**H**	**H**	83.8 (81.0,86.7)	83.5 (80.7,86.4)	83.1 (80.2,86.0)	97.5 (96.3,98.7)	97.7 (96.5,98.8)	85.7 (83.0,88.4)	84.3 (81.5,87.1)	85.7 (83.0,88.4)	85.5 (82.8,88.2)
**L**	**H**	**L**	**H**	0.0 (0.0,0.0)	0.0 (0.0,0.0)	0.0 (0.0,0.0)	94.0 (92.5,95.5)	94.4 (93.0,95.8)	81.7 (79.3,84.1)	81.8 (79.4,84.2)	0.1 (0.0,0.3)	0.0 (0.0,0.0)

v. Setting 5: $\pi = 0.5, \omega = 0.5, n_{obs} = 2000$

Table v.1 Number of converged data sets for different sensitivity-specificity combinations

				**Number of converged data sets**
**Specificity**				**Sensitivity: H H L L**
**H**	**H**	**L**	**L**	851
**H**	**H**	**H**	**L**	999
**H**	**H**	**H**	**H**	1000
**L**	**L**	**L**	**L**	678
**L**	**L**	**H**	**L**	970
**L**	**L**	**H**	**H**	994
**L**	**H**	**L**	**H**	993

Specificity				Sensitivity: H H H L
H	H	L	L	997
H	H	H	L	1000
H	H	H	H	1000
L	L	L	L	956
L	L	H	L	988
L	L	H	H	1000
L	H	L	H	1000
**Specificity**				**Sensitivity: H H H H**
H	H	L	L	1000
H	H	H	L	1000
H	H	H	H	1000
L	L	L	L	985
L	L	H	L	1000
L	L	H	H	999
L	H	L	H	1000
**Specificity**				**Sensitivity: L L L L**
H	H	L	L	543
H	H	H	L	960
H	H	H	H	999
L	L	L	L	298
L	L	H	L	899
L	L	H	H	882
L	H	L	H	913
**Specificity**				**Sensitivity: L L H L**
H	H	L	L	972
H	H	H	L	1000
H	H	H	H	975
L	L	L	L	891
L	L	H	L	984
L	L	H	H	783
L	H	L	H	953
**Specificity**				**Sensitivity: L L H H**
H	H	L	L	998
H	H	H	L	999
H	H	H	H	1000
L	L	L	L	935
L	L	H	L	617
L	L	H	H	887
L	H	L	H	798

Table v.2 Percentages of the time 95% credible intervals (CrIs) for residual correlations did not included ‘0’ for any pairs, and percentages of the time that the overall χ^2 or G^2 statistic indicated a lack of overall fit

**Sensitivity: H H L L**						
**Specificity**				**Res. Cor.**	**Overall G^2**	**Overall χ^2**
**H**	**H**	**L**	**L**	56.9 (53.5,60.2)	5.8 (4.3,7.5)	5.5 (4.1,7.3)
**H**	**H**	**H**	**L**	32.4 (29.5,35.4)	11.4 (9.5,13.5)	10.9 (9.0,13.0)
**H**	**H**	**H**	**H**	58.5 (55.4,61.6)	74.4 (71.6,77.1)	78.4 (75.7,80.9)
**L**	**L**	**L**	**L**	57.5 (53.7,61.3)	13.3 (10.8,16.1)	14.6 (12.0,17.5)
**L**	**L**	**H**	**L**	42.0 (38.8,45.1)	24.1 (21.5,26.9)	24.1 (21.5,26.9)
**L**	**L**	**H**	**H**	76.6 (73.8,79.2)	95.8 (94.3,96.9)	97.3 (96.1,98.2)
**L**	**H**	**L**	**H**	37.1 (34.0,40.1)	16.8 (14.5,19.3)	16.1 (13.9,18.5)
**Sensitivity: H H H L**						
**Specificity**				**Res. Cor.**	**Overall G^2**	**Overall χ^2**
**H**	**H**	**L**	**L**	32.3 (29.4,35.3)	8.8 (7.1,10.8)	8.0 (6.4,9.9)
**H**	**H**	**H**	**L**	12.2 (10.2,14.4)	18.3 (15.9,20.8)	17.9 (15.6,20.4)
**H**	**H**	**H**	**H**	79.6 (77.0,82.1)	97.8 (96.7,98.6)	98.5 (97.5,99.2)
**L**	**L**	**L**	**L**	40.8 (37.7,44.0)	18.8 (16.4,21.5)	18.8 (16.4,21.5)
**L**	**L**	**H**	**L**	37.3 (34.3,40.4)	45.5 (42.4,49.4)	46.3 (43.1,49.4)
**L**	**L**	**H**	**H**	92.7 (90.9,94.2)	99.9 (99.4,100.0)	99.9 (99.4,100.0)
**L**	**H**	**L**	**H**	76.1 (73.3,78.7)	72.7 (69.8,75.4)	73.1 (70.2,75.8)
**Sensitivity: H H H H**						
**Specificity**				**Res. Cor.**	**Overall G^2**	**Overall χ^2**
**H**	**H**	**L**	**L**	64.9 (61.9,67.9)	25.1 (22.4,27.9)	24.2 (21.6,27.0)
**H**	**H**	**H**	**L**	88.4 (86.3,90.3)	87.0 (84.8,89.0)	86.5 (84.2,88.6)
**H**	**H**	**H**	**H**	99.9 (99.4,100.0)	100.0 (99.6,100.0)	100.0 (99.6,100.0)
**L**	**L**	**L**	**L**	86.5 (84.2,88.6)	70.3 (67.3,73.1)	70.9 (67.9,73.7)
**L**	**L**	**H**	**L**	98.7 (97.8,99.3)	99.8 (99.3,100.0)	99.8 (99.3,100.0)
**L**	**L**	**H**	**H**	98.8 (97.9,99.4)	100.0 (99.6,100.0)	100.0 (99.6,100.0)
**L**	**H**	**L**	**H**	96.3 (94.9,97.4)	97.9 (96.8,98.7)	97.8 (96.7,98.6)
**Sensitivity: L L L L**						
**Specificity**				**Res. Cor.**	**Overall G^2**	**Overall χ^2**
**H**	**H**	**L**	**L**	75.9 (72.0,79.4)	9.6 (7.2,12.4)	8.8 (6.6,11.5)
**H**	**H**	**H**	**L**	91.5 (89.5,93.1)	60.5 (57.3,63.6)	60.8 (57.7,63.9)
**H**	**H**	**H**	**H**	100.0 (99.6,100.0)	100.0 (99.6,100.0)	100.0 (99.6,100.0)
**L**	**L**	**L**	**L**	87.9 (83.7,91.4)	48.3 (42.5,54.2)	49.0 (43.2,54.8)
**L**	**L**	**H**	**L**	99.8 (99.2,100.0)	97.3 (96.1,98.3)	97.3 (96.1,98.3)
**L**	**L**	**H**	**H**	100.0 (99.6,100.0)	100.0 (99.6,100.0)	100.0 (99.6,100.0)
**L**	**H**	**L**	**H**	99.3 (98.6,99.8)	93.5 (91.7,95.0)	93.8 (92.0,95.2)
**Sensitivity: L L H L**						
**Specificity**				**Res. Cor.**	**Overall G^2**	**Overall χ^2**

**H**	**H**	**L**	**L**	92.5 (90.6,94.1)	45.4 (42.2,48.6)	44.4 (41.3,47.6)
**H**	**H**	**H**	**L**	99.4 (98.7,99.8)	93.1 (91.3,94.6)	93.2 (91.5,94.7)
**H**	**H**	**H**	**H**	100.0 (99.6,100.0)	100.0 (99.6,100.0)	100.0 (99.6,100.0)
**L**	**L**	**L**	**L**	100.0 (99.6,100.0)	96.6 (95.2,97.7)	96.7 (95.4,97.8)
**L**	**L**	**H**	**L**	100.0 (99.6,100.0)	100.0 (99.6,100.0)	100.0 (99.6,100.0)
**L**	**L**	**H**	**H**	100.0 (99.5,100.0)	100.0 (99.5,100.0)	100.0 (99.5,100.0)
**L**	**H**	**L**	**H**	100.0 (99.6,100.0)	100.0 (99.6,100.0)	100.0 (99.6,100.0)
**Sensitivity: L L H H**						
**Specificity**				**Res. Cor.**	**Overall G^2**	**Overall χ^2**
**H**	**H**	**L**	**L**	100.0 (99.6,100.0)	100.0 (99.6,100.0)	100.0 (99.6,100.0)
**H**	**H**	**H**	**L**	100.0 (99.6,100.0)	100.0 (99.6,100.0)	100.0 (99.6,100.0)
**H**	**H**	**H**	**H**	100.0 (99.6,100.0)	100.0 (99.6,100.0)	100.0 (99.6,100.0)
**L**	**L**	**L**	**L**	100.0 (99.6,100.0)	100.0 (99.6,100.0)	100.0 (99.6,100.0)
**L**	**L**	**H**	**L**	100.0 (99.4,100.0)	100.0 (99.4,100.0)	100.0 (99.4,100.0)
**L**	**L**	**H**	**H**	100.0 (99.6,100.0)	100.0 (99.6,100.0)	100.0 (99.6,100.0)
**L**	**H**	**L**	**H**	100.0 (99.5,100.0)	100.0 (99.5,100.0)	100.0 (99.5,100.0)

Table v.3 Percentages of the time 95% CrIs for residual correlations did not included '0' for each pair, and percentages of the time the pairwise χ^2 or G^2 statistic for each pair indicated a lack of pairwise fit

**Sensitivity: H H L L**										
**Specificity**				**Tool**	**T_1&T_2**	**T_1&T_3**	**T_1&T_4**	**T_2&T_3**	**T_2&T_4**	**T_3&T_4**
**H**	**H**	**L**	**L**	**Res. Cor.**	0.0 (0.0,0.4)	0.0 (0.0,0.4)	0.0 (0.0,0.4)	0.0 (0.0,0.4)	0.0 (0.0,0.4)	56.9 (53.5,60.2)
				**Pairwise G^2**	0.0 (0.0,0.4)	0.0 (0.0,0.4)	0.0 (0.0,0.4)	0.0 (0.0,0.4)	0.0 (0.0,0.4)	4.8 (3.5,6.5)
				**Pairwise χ^2**	0.0 (0.0,0.4)	0.0 (0.0,0.4)	0.0 (0.0,0.4)	0.0 (0.0,0.4)	0.0 (0.0,0.4)	4.8 (3.5,6.5)
**H**	**H**	**H**	**L**	**Res. Cor.**	0.0 (0.0,0.4)	0.0 (0.0,0.4)	0.0 (0.0,0.4)	0.0 (0.0,0.4)	0.0 (0.0,0.4)	32.4 (29.5,35.4)
				**Pairwise G^2**	0.0 (0.0,0.4)	0.0 (0.0,0.4)	0.0 (0.0,0.4)	0.0 (0.0,0.4)	0.0 (0.0,0.4)	5.7 (4.3.7.3)
				**Pairwise χ^2**	0.0 (0.0,0.4)	0.0 (0.0,0.4)	0.0 (0.0,0.4)	0.0 (0.0,0.4)	0.0 (0.0,0.4)	5.6 (4.3,7.2)
**H**	**H**	**H**	**H**	**Res. Cor.**	0.0 (0.0,0.4)	0.0 (0.0,0.4)	0.0 (0.0,0.4)	0.0 (0.0,0.4)	0.0 (0.0,0.4)	58.5 (55.4,61.6)
				**Pairwise G^2**	0.0 (0.0,0.4)	0.0 (0.0,0.4)	0.0 (0.0,0.4)	0.0 (0.0,0.4)	0.0 (0.0,0.4)	25.4 (22.7,28.2)
				**Pairwise χ^2**	0.0 (0.0,0.4)	0.0 (0.0,0.4)	0.0 (0.0,0.4)	0.0 (0.0,0.4)	0.0 (0.0,0.4)	25.3 (22.6,28.1)
**L**	**L**	**L**	**L**	**Res. Cor.**	0.0 (0.0,0.5)	1.0 (0.4,2.1)	1.0 (0.4,2.1)	1.5 (0.7,2.7)	0.9 (0.3,1.9)	56.0 (52.2,59.8)
				**Pairwise G^2**	0.0 (0.0,0.5)	0.1 (0.0,0.8)	0.1 (0.0,0.8)	0.1 (0.0,0.8)	0.0 (0.0,0.5)	7.1 (5.3,9.3)
				**Pairwise χ^2**	0.0 (0.0,0.5)	0.1 (0.0,0.8)	0.1 (0.0,0.8)	0.3 (0.0,1.1)	0.0 (0.0,0.5)	7.1 (5.3,9.3)
**L**	**L**	**H**	**L**	**Res. Cor.**	0.0 (0.0,0.4)	0.0 (0.0,0.4)	3.1 (2.1,4.4)	0.1 (0.0,0.6)	3.2 (2.2,4.5)	38.8 (35.7,41.9)
				**Pairwise G^2**	0.0 (0.0,0.4)	0.0 (0.0,0.4)	0.5 (0.2,1.2)	0.0 (0.0,0.4)	0.3 (0.1,0.9)	11.5 (9.6,13.7)
				**Pairwise χ^2**	0.0 (0.0,0.4)	0.0 (0.0,0.4)	0.5 (0.2,1.2)	0.0 (0.0,0.4)	0.3 (0.1,0.9)	11.5 (9.6,13.7)
**L**	**L**	**H**	**H**	**Res. Cor.**	0.0 (0.0,0.4)	0.3 (0.1,0.9)	0.6 (0.2,1.3)	0.5 (0.2,1.2)	0.8 (0.3,1.6)	76.6 (73.8,79.2)
				**Pairwise G^2**	0.0 (0.0,0.4)	0.0 (0.0,0.4)	0.1 (0.0,0.6)	0.1 (0.0,0.6)	0.2 (0.0,0.7)	55.7 (52.6,58.9)

				**Pairwise χ^2**	0.0 (0.0,0.4)	0.0 (0.0,0.4)	0.1 (0.0,0.6)	0.2 (0.0,0.7)	0.2 (0.0,0.7)	55.5 (52.4,58.7)
**L**	**H**	**L**	**H**	**Res. Cor.**	0.0 (0.0,0.4)	5.4 (4.1,7.0)	0.1 (0.0,0.6)	0.0 (0.0,0.4)	0.0 (0.0,0.4)	33.3 (30.4,36.4)
				**Pairwise G^2**	0.0 (0.0,0.4)	0.6 (0.2,1.3)	0.0 (0.0,0.4)	0.0 (0.0,0.4)	0.0 (0.0,0.4)	7.7 (6.1,9.5)
				**Pairwise χ^2**	0.0 (0.0,0.4)	0.6 (0.2,1.3)	0.0 (0.0,0.4)	0.0 (0.0,0.4)	0.0 (0.0,0.4)	7.6 (6.0,9.4)
**Sensitivity: H H H L**										
**Specificity**				**Tool**	**T_1&T_2**	**T_1&T_3**	**T_1&T_4**	**T_2&T_3**	**T_2&T_4**	**T_3&T_4**
**H**	**H**	**L**	**L**	**Res. Cor.**	0.0 (0.0,0.4)	0.0 (0.0,0.4)	0.0 (0.0,0.4)	0.0 (0.0,0.4)	0.0 (0.0,0.4)	32.3 (29.4,35.3)
				**Pairwise G^2**	0.0 (0.0,0.4)	0.0 (0.0,0.4)	0.0 (0.0,0.4)	0.0 (0.0,0.4)	0.0 (0.0,0.4)	6.1 (4.7,7.8)
				**Pairwise χ^2**	0.0 (0.0,0.4)	0.0 (0.0,0.4)	0.0 (0.0,0.4)	0.0 (0.0,0.4)	0.0 (0.0,0.4)	6.1 (4.7,7.8)
**H**	**H**	**H**	**L**	**Res. Cor.**	0.0 (0.0,0.4)	0.0 (0.0,0.4)	0.0 (0.0,0.4)	0.0 (0.0,0.4)	0.0 (0.0,0.4)	12.2 (10.2,14.4)
				**Pairwise G^2**	0.0 (0.0,0.4)	0.0 (0.0,0.4)	0.0 (0.0,0.4)	0.0 (0.0,0.4)	0.0 (0.0,0.4)	4.1 (3.0,5.5)
				**Pairwise χ^2**	0.0 (0.0,0.4)	0.0 (0.0,0.4)	0.0 (0.0,0.4)	0.0 (0.0,0.4)	0.0 (0.0,0.4)	4.0 (2.9,5.4)
**H**	**H**	**H**	**H**	**Res. Cor.**	0.0 (0.0,0.4)	0.0 (0.0,0.4)	0.0 (0.0,0.4)	0.0 (0.0,0.4)	0.0 (0.0,0.4)	79.6 (77.0,82.1)
				**Pairwise G^2**	0.0 (0.0,0.4)	0.0 (0.0,0.4)	0.0 (0.0,0.4)	0.0 (0.0,0.4)	0.0 (0.0,0.4)	64.0 (60.9,67.0)
				**Pairwise χ^2**	0.0 (0.0,0.4)	0.0 (0.0,0.4)	0.0 (0.0,0.4)	0.0 (0.0,0.4)	0.0 (0.0,0.4)	63.1 (60.0,66.1)
**L**	**L**	**L**	**L**	**Res. Cor.**	0.0 (0.0,0.4)	0.2 (0.0,0.8)	3.0 (2.0,4.3)	0.1 (0.0,0.6)	3.8 (2.7,5.2)	37.9 (34.8,41.0)
				**Pairwise G^2**	0.0 (0.0,0.4)	0.1 (0.0,0.6)	0.3 (0.1,0.9)	0.0 (0.0,0.4)	0.3 (0.1,0.9)	14.9 (12.7,17.3)
				**Pairwise χ^2**	0.0 (0.0,0.4)	0.1 (0.0,0.6)	0.3 (0.1,0.9)	0.0 (0.0,0.4)	0.3 (0.1,0.9)	14.9 (12.7,17.3)
**L**	**L**	**H**	**L**	**Res. Cor.**	0.0 (0.0,0.4)	0.0 (0.0,0.4)	5.9 (4.5,7.5)	0.0 (0.0,0.4)	6.3 (4.8,8.0)	31.4 (28.5,34.4)
				**Pairwise G^2**	0.0 (0.0,0.4)	0.0 (0.0,0.4)	0.9 (0.4,1.7)	0.0 (0.0,0.4)	1.3 (0.7,2.2)	16.6 (14.3,19.1)
				**Pairwise χ^2**	0.0 (0.0,0.4)	0.0 (0.0,0.4)	0.9 (0.4,1.7)	0.0 (0.0,0.4)	1.3 (0.7,2.2)	16.6 (14.3,19.1)
**L**	**L**	**H**	**H**	**Res. Cor.**	24.0 (21.4,26.8)	0.9 (0.4,1.7)	9.9 (8.1,11.9)	0.6 (0.2,1.3)	10.5 (8.7,12.6)	91.9 (90.0,93.5)
				**Pairwise G^2**	10.8 (8.9,12.9)	0.3 (0.1,0.9)	3.0 (2.0,4.3)	0.1 (0.0,0.6)	2.9 (2.0,4.1)	90.6 (88.6,92.3)
				**Pairwise χ^2**	10.6 (8.8,12.7)	0.3 (0.1,0.9)	3.4 (2.4,4.7)	0.3 (0.1,0.9)	3.4 (2.4,4.7)	89.6 (87.5,91.4)
**L**	**H**	**L**	**H**	**Res. Cor.**	0.0 (0.0,0.4)	11.5 (9.6,13.6)	1.8 (1.1,2.8)	0.0 (0.0,0.4)	0.0 (0.0,0.4)	74.9 (72.1,77.6)
				**Pairwise G^2**	0.0 (0.0,0.4)	1.3 (0.7,2.2)	0.3 (0.1,0.9)	0.0 (0.0,0.4)	0.0 (0.0,0.4)	50.9 (47.8,54.0)
				**Pairwise χ^2**	0.0 (0.0,0.4)	1.3 (0.7,2.2)	0.4 (0.1,1.0)	0.0 (0.0,0.4)	0.0 (0.0,0.4)	49.4 (46.3,52.5)
**Sensitivity: H H H H**										
**Specificity**				**Tool**	**T_1&T_2**	**T_1&T_3**	**T_1&T_4**	**T_2&T_3**	**T_2&T_4**	**T_3&T_4**
**H**	**H**	**L**	**L**	**Res. Cor.**	0.0 (0.0,0.4)	0.0 (0.0,0.4)	0.0 (0.0,0.4)	0.0 (0.0,0.4)	0.0 (0.0,0.4)	64.9 (61.9,67.9)
				**Pairwise G^2**	0.0 (0.0,0.4)	0.0 (0.0,0.4)	0.0 (0.0,0.4)	0.0 (0.0,0.4)	0.0 (0.0,0.4)	31.1 (28.2,34.1)
				**Pairwise χ^2**	0.0 (0.0,0.4)	0.0 (0.0,0.4)	0.0 (0.0,0.4)	0.0 (0.0,0.4)	0.0 (0.0,0.4)	30.7 (27.9,33.7)
**H**	**H**	**H**	**L**	**Res. Cor.**	0.0 (0.0,0.4)	0.0 (0.0,0.4)	0.0 (0.0,0.4)	0.0 (0.0,0.4)	0.0 (0.0,0.4)	88.4 (86.3,90.3)
				**Pairwise G^2**	0.0 (0.0,0.4)	0.0 (0.0,0.4)	0.0 (0.0,0.4)	0.0 (0.0,0.4)	0.0 (0.0,0.4)	73.5 (70.6,76.2)
				**Pairwise χ^2**	0.0 (0.0,0.4)	0.0 (0.0,0.4)	0.0 (0.0,0.4)	0.0 (0.0,0.4)	0.0 (0.0,0.4)	72.8 (69.9,75.5)
**H**	**H**	**H**	**H**	**Res. Cor.**	0.1 (0.0,0.6)	0.0 (0.0,0.4)	0.0 (0.0,0.4)	0.0 (0.0,0.4)	0.0 (0.0,0.4)	99.9 (99.4,100.0)
				**Pairwise G^2**	0.1 (0.0,0.6)	0.0 (0.0,0.4)	0.0 (0.0,0.4)	0.0 (0.0,0.4)	0.0 (0.0,0.4)	99.5 (98.8,99.8)
				**Pairwise χ^2**	0.1 (0.0,0.6)	0.0 (0.0,0.4)	0.0 (0.0,0.4)	0.0 (0.0,0.4)	0.0 (0.0,0.4)	99.5 (98.8,99.8)
**L**	**L**	**L**	**L**	**Res. Cor.**	1.5 (0.9,2.5)	2.5 (1.6,3.7)	3.7 (2.6,5.0)	4.2 (3.0,5.6)	3.1 (2.1,4.4)	85.8 (83.4,87.9)
				**Pairwise G^2**	0.8 (0.4,1.6)	0.2 (0.0,0.7)	0.3 (0.1,0.9)	0.6 (0.2,1.3)	0.4 (0.1,1.0)	65.0 (61.9,68.0)
				**Pairwise χ^2**	0.8 (0.4,1.6)	0.2 (0.0,0.7)	0.3 (0.1,0.9)	0.6 (0.2,1.3)	0.4 (0.1,1.0)	64.9 (61.8,67.9)

**L**	**L**	**H**	**L**	**Res. Cor.**	15.8 (13.6,18.2)	0.1 (0.0,0.6)	12.9 (10.9,15.1)	0.2 (0.0,0.7)	12.4 (10.4,14.6)	98.7 (97.8,99.3)
				**Pairwise G^2**	6.2 (4.8,7.9)	0.0 (0.0,0.4)	3.7 (2.6,5.1)	0.0 (0.0,0.4)	3.3 (2.3,4.6)	96.8 (95.5,97.8)
				**Pairwise χ^2**	6.0 (4.6,7.7)	0.0 (0.0,0.4)	3.7 (2.6,5.1)	0.1 (0.0,0.6)	3.4 (2.4,4.7)	96.8 (95.5,97.8)
**L**	**L**	**H**	**H**	**Res. Cor.**	80.1 (77.5,82.5)	3.3 (2.3,4.6)	2.7 (1.8,3.9)	2.8 (1.9,4.0)	2.4 (1.5,3.6)	92.6 (90.8,94.1)
				**Pairwise G^2**	58.2 (55.0,61.2)	1.3 (0.7,2.2)	1.1 (0.6,2.0)	0.9 (0.4,1.7)	0.8 (0.3,1.6)	95.8 (94.4,97.0)
				**Pairwise χ^2**	57.6 (54.4,60.6)	1.3 (0.7,2.2)	1.3 (0.7,2.2)	1.1 (0.6,2.0)	1.1 (0.6,2.0)	95.3 (93.8.,96.5)
**L**	**H**	**L**	**H**	**Res. Cor.**	0.0 (0.0,0.4)	16.5 (14.3,18.9)	3.2 (2.2,4.5)	0.0 (0.0,0.4)	0.0 (0.0,0.4)	96.0 (94.6,97.1)
				**Pairwise G^2**	0.0 (0.0,0.4)	3.6 (2.5,4.9)	1.3 (0.7,2.2)	0.0 (0.0,0.4)	0.0 (0.0,0.4)	92.1 (90.3,93.7)
				**Pairwise χ^2**	0.0 (0.0,0.4)	3.8 (2.7,5.2)	1.4 (0.8,2.3)	0.0 (0.0,0.4)	0.0 (0.0,0.4)	91.6 (89.7,93.2)
**Sensitivity: L L L L**										
**Specificity**				**Tool**	**T_1&T_2**	**T_1&T_3**	**T_1&T_4**	**T_2&T_3**	**T_2&T_4**	**T_3&T_4**
**H**	**H**	**L**	**L**	**Res. Cor.**	0.0 (0.0,0.7)	0.0 (0.0,0.7)	0.0 (0.0,0.7)	0.2 (0.0,1.0)	0.0 (0.0,0.7)	75.9 (72.0,79.4)
				**Pairwise G^2**	0.0 (0.0,0.7)	0.0 (0.0,0.7)	0.0 (0.0,0.7)	0.0 (0.0,0.7)	0.0 (0.0,0.7)	15.7 (12.7,19.0)
				**Pairwise χ^2**	0.0 (0.0,0.7)	0.0 (0.0,0.7)	0.0 (0.0,0.7)	0.0 (0.0,0.7)	0.0 (0.0,0.7)	15.7 (12.7,19.0)
**H**	**H**	**H**	**L**	**Res. Cor.**	0.0 (0.0,0.4)	0.0 (0.0,0.4)	0.1 (0.0,0.6)	0.0 (0.0,0.4)	0.1 (0.0,0.6)	91.5 (89.5,93.1)
				**Pairwise G^2**	0.0 (0.0,0.4)	0.0 (0.0,0.4)	0.0 (0.0,0.4)	0.0 (0.0,0.4)	0.0 (0.0,0.4)	62.2 (59.0,65.3)
				**Pairwise χ^2**	0.0 (0.0,0.4)	0.0 (0.0,0.4)	0.0 (0.0,0.4)	0.0 (0.0,0.4)	0.0 (0.0,0.4)	62.2 (59.0,65.3)
**H**	**H**	**H**	**H**	**Res. Cor.**	0.1 (0.0,0.6)	0.2 (0.0,0.7)	0.0 (0.0,0.4)	0.4 (0.1,1.0)	0.0 (0.0,0.4)	100.0 (99.6,100.0)
				**Pairwise G^2**	0.3 (0.1,0.9)	0.1 (0.0,0.6)	0.0 (0.0,0.4)	0.0 (0.0,0.4)	0.0 (0.0,0.4)	100.0 (99.6,100.0)
				**Pairwise χ^2**	0.3 (0.1,0.9)	0.1 (0.0,0.6)	0.0 (0.0,0.4)	0.0 (0.0,0.4)	0.0 (0.0,0.4)	100.0 (99.6,100.0)
**L**	**L**	**L**	**L**	**Res. Cor.**	0.0 (0.0,1.2)	7.4 (4.7,11.0)	5.7 (3.4,9.0)	7.4 (4.7,11.0)	9.7 (6.6,13.7)	84.9 (80.3,88.8)
				**Pairwise G^2**	0.0 (0.0,1.2)	0.7 (0.1,2.4)	0.7 (0.1,2.4)	0.7 (0.1,2.4)	1.0 (0.2,2.9)	23.2 (18.5,28.4)
				**Pairwise χ^2**	0.0 (0.0,1.2)	0.7 (0.1,2.4)	0.7 (0.1,2.4)	0.7 (0.1,2.4)	1.0 (0.2,2.9)	23.2 (18.5,28.4)
**L**	**L**	**H**	**L**	**Res. Cor.**	0.0 (0.0,0.4)	18.5 (16.0,21.2)	16.4 (14.0,18.9)	17.7 (15.2,20.3)	16.1 (13.8,18.7)	99.7 (99.0,99.9)
				**Pairwise G^2**	0.0 (0.0,0.4)	3.8 (2.6,5.2)	3.2 (2.2,4.6)	4.3 (3.1,5.9)	2.4 (1.5,3.7)	95.3 (93.7,96.6)
				**Pairwise χ^2**	0.0 (0.0,0.4)	3.8 (2.6,5.2)	3.2 (2.2,4.6)	4.6 (3.3,6.1)	2.4 (1.5,3.7)	95.3 (93.7,96.6)
**L**	**L**	**H**	**H**	**Res. Cor.**	6.7 (5.1,8.5)	66.7 (63.4,69.8)	65.4 (62.2,68.6)	63.9 (60.7,67.1)	64.9 (61.6,68.0)	98.5 (97.5,99.2)
				**Pairwise G^2**	11.6 (9.5,13.9)	33.8 (30.7,37.0)	36.6 (33.4,39.9)	33.6 (30.4,36.8)	36.1 (32.9,39.3)	98.8 (97.8,99.4)
				**Pairwise χ^2**	11.3 (9.3,13.6)	34.5 (31.3,37.7)	37.0 (33.8,40.2)	34.5 (31.3,37.7)	36.3 (33.1,39.6)	98.8 (97.8,99.4)
**L**	**H**	**L**	**H**	**Res. Cor.**	0.0 (0.0,0.4)	39.0 (35.8,42.2)	61.7 (58.4,64.8)	0.0 (0.0,0.4)	0.0 (0.0,0.4)	98.6 (97.6,99.2)
				**Pairwise G^2**	0.0 (0.0,0.4)	6.5 (5.0,8.3)	25.3 (22.5,28.3)	0.0 (0.0,0.4)	0.0 (0.0,0.4)	79.7 (77.0,82.3)
				**Pairwise χ^2**	0.0 (0.0,0.4)	6.5 (5.0,8.3)	25.4 (22.6,28.4)	0.0 (0.0,0.4)	0.0 (0.0,0.4)	79.7 (77.0,82.3)
**Sensitivity: L L H L**										
**Specificity**				**Tool**	**T_1&T_2**	**T_1&T_3**	**T_1&T_4**	**T_2&T_3**	**T_2&T_4**	**T_3&T_4**
**H**	**H**	**L**	**L**	**Res. Cor.**	0.0 (0.0,0.4)	0.0 (0.0,0.4)	0.1 (0.0,0.6)	0.0 (0.0,0.4)	0.1 (0.0,0.6)	92.5 (90.6,94.1)
				**Pairwise G^2**	0.0 (0.0,0.4)	0.0 (0.0,0.4)	0.0 (0.0,0.4)	0.0 (0.0,0.4)	0.0 (0.0,0.4)	61.5 (58.4,64.6)
				**Pairwise χ^2**	0.0 (0.0,0.4)	0.0 (0.0,0.4)	0.0 (0.0,0.4)	0.0 (0.0,0.4)	0.0 (0.0,0.4)	61.5 (58.4,64.6)
**H**	**H**	**H**	**L**	**Res. Cor.**	0.0 (0.0,0.4)	0.0 (0.0,0.4)	2.1 (1.3,3.2)	0.0 (0.0,0.4)	1.8 (1.1,2.8)	99.3 (98.6,99.7)
				**Pairwise G^2**	0.0 (0.0,0.4)	0.0 (0.0,0.4)	0.2 (0.0,0.7)	0.0 (0.0,0.4)	0.5 (0.2,1.2)	93.4 (91.7,94.9)
				**Pairwise χ^2**	0.0 (0.0,0.4)	0.0 (0.0,0.4)	0.3 (0.1,0.9)	0.0 (0.0,0.4)	0.6 (0.2,1.3)	93.4 (91.7,94.9)
**H**	**H**	**H**	**H**	**Res. Cor.**	69.9 (67.0,72.8)	0.0 (0.0,0.4)	48.2 (45.0,51.4)	0.0 (0.0,0.4)	50.7 (47.5,53.9)	99.8 (99.3,100.0)

				**Pairwise G^2**	82.5 (79.9,84.8)	0.0 (0.0,0.4)	29.3 (26.5,32.3)	0.0 (0.0,0.4)	29.4 (26.6,32.4)	100.0 (99.6,100.0)
				**Pairwise χ^2**	81.4 (78.9,83.8)	0.0 (0.0,0.4)	30.3 (27.4,33.2)	0.0 (0.0,0.4)	30.2 (27.3,33.1)	100.0 (99.6,100.0)
**L**	**L**	**L**	**L**	**Res. Cor.**	0.0 (0.0,0.4)	28.7 (25.8,31.8)	18.6 (16.1,21.3)	30.1 (27.1,33.2)	17.5 (15.1,20.2)	99.7 (99.0,99.9)
				**Pairwise G^2**	0.0 (0.0,0.4)	9.4 (7.6,11.5)	3.1 (2.1,4.5)	9.4 (7.6,11.5)	3.5 (2.4,4.9)	94.5 (92.8,95.9)
				**Pairwise χ^2**	0.0 (0.0,0.4)	9.4 (7.6,11.5)	3.1 (2.1,4.5)	10.1 (8.2,12.3)	3.5 (2.4,4.9)	94.5 (92.8,95.9)
**L**	**L**	**H**	**L**	**Res. Cor.**	0.0 (0.0,0.4)	54.7 (51.5,57.8)	31.2 (28.3,34.2)	53.0 (49.9,56.2)	33.2 (30.3,36.3)	100.0 (99.6,100.0)
				**Pairwise G^2**	0.0 (0.0,0.4)	26.4 (23.7,29.3)	8.9 (7.2,10.9)	26.2 (23.5,29.1)	8.7 (7.1,10.7)	100.0 (99.6,100.0)
				**Pairwise χ^2**	0.0 (0.0,0.4)	26.7 (24.0,29.6)	8.9 (7.2,10.9)	26.4 (23.7,29.3)	8.8 (7.1,10.8)	100.0 (99.6,100.0)
**L**	**L**	**H**	**H**	**Res. Cor.**	100.0 (99.5,100.0)	3.7 (2.5,5.3)	57.3 (53.8,60.8)	4.0 (2.7,5.6)	59.3 (55.7,62.7)	1.5 (0.8,2.7)
				**Pairwise G^2**	100.0 (99.5,100.0)	2.9 (1.9,4.4)	32.7 (29.4,36.1)	2.2 (1.3,3.5)	35.2 (31.9,38.7)	3.2 (2.1,4.7)
				**Pairwise χ^2**	100.0 (99.5,100.0)	2.9 (1.9,4.4)	33.0 (29.7,36.4)	2.2 (1.3,3.5)	35.9 (32.5,39.4)	3.2 (2.1,4.7)
**L**	**H**	**L**	**H**	**Res. Cor.**	0.2 (0.0,0.8)	91.3 (89.3,93.0)	93.0 (91.2,94.5)	0.0 (0.0,0.4)	0.0 (0.0,0.4)	100.0 (99.6,100.0)
				**Pairwise G^2**	0.5 (0.2,1.2)	65.3 (62.1,68.3)	70.7 (67.7,73.6)	0.0 (0.0,0.4)	0.0 (0.0,0.4)	100.0 (99.6,100.0)
				**Pairwise χ^2**	0.5 (0.2,1.2)	65.5 (62.4,68.5)	71.1 (68.2,74.0)	0.0 (0.0,0.4)	0.0 (0.0,0.4)	100.0 (99.6,100.0)
**Sensitivity: L L H H**										
**Specificity**				**Tool**	**T_1&T_2**	**T_1&T_3**	**T_1&T_4**	**T_2&T_3**	**T_2&T_4**	**T_3&T_4**
**H**	**H**	**L**	**L**	**Res. Cor.**	0.0 (0.0,0.4)	0.0 (0.0,0.4)	0.2 (0.0,0.7)	0.0 (0.0,0.4)	0.1 (0.0,0.6)	100.0 (99.6,100.0)
				**Pairwise G^2**	0.0 (0.0,0.4)	0.0 (0.0,0.4)	0.0 (0.0,0.4)	0.0 (0.0,0.4)	0.0 (0.0,0.4)	100.0 (99.6,100.0)
				**Pairwise χ^2**	0.0 (0.0,0.4)	0.0 (0.0,0.4)	0.0 (0.0,0.4)	0.0 (0.0,0.4)	0.0 (0.0,0.4)	100.0 (99.6,100.0)
**H**	**H**	**H**	**L**	**Res. Cor.**	11.0 (9.1,13.1)	0.0 (0.0,0.4)	12.2 (10.2,14.4)	0.0 (0.0,0.4)	10.2 (8.4,12.3)	100.0 (99.6,100.0)
				**Pairwise G^2**	23.5 (20.9,26.3)	0.0 (0.0,0.4)	2.9 (2.0,4.1)	0.0 (0.0,0.4)	2.9 (2.0,4.1)	100.0 (99.6,100.0)
				**Pairwise χ^2**	22.6 (20.1,25.3)	0.0 (0.0,0.4)	3.6 (2.5,5.0)	0.0 (0.0,0.4)	3.4 (2.4,4.7)	100.0 (99.6,100.0)
**H**	**H**	**H**	**H**	**Res. Cor.**	100.0 (99.6,100.0)	0.9 (0.4,1.7)	0.4 (0.1,1.0)	1.0 (0.5,1.8)	1.0 (0.5,1.8)	5.2 (3.9,6.8)
				**Pairwise G^2**	100.0 (99.6,100.0)	0.2 (0.0,0.7)	0.3 (0.1,0.9)	0.4 (0.1,1.0)	0.0 (0.0,0.4)	12.3 (10.3,14.5)
				**Pairwise χ^2**	100.0 (99.6,100.0)	0.3 (0.1,0.9)	0.3 (0.1,0.9)	0.4 (0.1,1.0)	0.0 (0.0,0.4)	11.4 (9.5,13.5)
**L**	**L**	**L**	**L**	**Res. Cor.**	30.9 (28.0,34.0)	78.6 (75.8,81.2)	79.0 (76.3,81.6)	79.5 (76.7,82.0)	78.6 (75.8,81.2)	100.0 (99.6,100.0)
				**Pairwise G^2**	28.3 (25.5,31.3)	45.1 (41.9,48.4)	48.6 (45.3,51.8)	50.4 (47.1,53.6)	48.0 (44.8,51.3)	100.0 (99.6,100.0)
				**Pairwise χ^2**	27.8 (25.0,30.8)	45.3 (42.1,48.6)	48.9 (45.6,52.1)	51.0 (47.8,54.3)	48.9 (45.6,52.1)	100.0 (99.6,100.0)
**L**	**L**	**H**	**L**	**Res. Cor.**	100.0 (99.4,100.0)	13.1 (10.6,16.1)	32.1 (28.4,35.9)	13.6 (11.0,16.6)	34.0 (30.3,37.9)	13.8 (11.2,16.8)
				**Pairwise G^2**	99.8 (99.1,100.0)	13.0 (10.4,15.9)	16.2 (13.4,19.4)	13.1 (10.6,16.1)	18.3 (15.3,21.6)	16.9 (14.0,20.0)
				**Pairwise χ^2**	99.8 (99.1,100.0)	13.0 (10.4,15.9)	16.4 (13.5,19.5)	13.1 (10.6,16.1)	18.5 (15.5,21.8)	16.5 (13.7,19.7)
**L**	**L**	**H**	**H**	**Res. Cor.**	100.0 (99.6,100.0)	0.0 (0.0,0.4)	0.0 (0.0,0.4)	0.0 (0.0,0.4)	0.0 (0.0,0.4)	0.0 (0.0,0.4)
				**Pairwise G^2**	100.0 (99.6,100.0)	0.0 (0.0,0.4)	0.0 (0.0,0.4)	0.0 (0.0,0.4)	0.0 (0.0,0.4)	0.0 (0.0,0.4)
				**Pairwise χ^2**	100.0 (99.6,100.0)	0.0 (0.0,0.4)	0.0 (0.0,0.4)	0.0 (0.0,0.4)	0.0 (0.0,0.4)	0.0 (0.0,0.4)
**L**	**H**	**L**	**H**	**Res. Cor.**	45.1 (41.6,48.6)	95.1 (93.4,96.5)	97.2 (95.9,98.3)	3.3 (2.1,4.7)	0.0 (0.0,0.5)	94.7 (93.0,96.2)
				**Pairwise G^2**	57.3 (53.8,60.7)	78.7 (75.7,81.5)	96.5 (95.0,97.7)	0.6 (0.2,1.5)	0.0 (0.0,0.5)	97.9 (96.6,98.8)
				**Pairwise χ^2**	56.5 (53.0,60.0)	78.9 (76.0,81.7)	96.5 (95.0,97.7)	1.1 (0.5,2.1)	0.0 (0.0,0.5)	97.9 (96.6,98.8)

Table v.4 Mean absolute biases (x10^{-2}) of posterior medians of each parameter obtained from the conditional independence model

**Sensitivity: H H L L**												
**Specificity**				π	Se_1	Se_2	Se_3	Se_4	Sp_1	Sp_2	Sp_3	Sp_4
**H**	**H**	**L**	**L**	-2.9 (-3.0,-2.7)	5.4 (5.2,5.5)	5.2 (5.0,5.3)	-0.2 (-0.3,-0.1)	-0.1 (-0.2,0.0)	0.4 (0.2,0.5)	0.2 (0.1,0.4)	-1.2 (-1.3,-1.1)	-1.3 (-1.4,-1.2)
**H**	**H**	**H**	**L**	-3.2 (-3.3,-3.1)	5.5 (5.5,5.6)	5.5 (5.4,5.6)	-0.1 (-0.2,0.0)	0.0 (-0.1,0.1)	0.0 (-0.1,0.1)	0.0 (-0.1,0.1)	-3.2 (-3.3,-3.1)	-1.3 (-1.4,-1.2)
**H**	**H**	**H**	**H**	-3.2 (-3.2,-3.1)	5.4 (5.3,5.5)	5.4 (5.3,5.5)	0.1 (0.0,0.2)	0.2 (0.1,0.3)	0.0 (-0.1,0.1)	0.0 (-0.1,0.1)	-2.9 (-3.0,-2.9)	-2.9 (-3.0,-2.9)
**L**	**L**	**L**	**L**	-2.5 (-2.7,-2.2)	5.6 (5.5,5.8)	5.6 (5.4,5.8)	-0.7 (-0.9,-0.6)	-0.6 (-0.7,-0.5)	2.7 (2.5,2.9)	2.7 (2.5,2.9)	-1.5 (-1.6,-1.3)	-1.5 (-1.6,-1.4)
**L**	**L**	**H**	**L**	-5.4 (-5.6,-5.3)	7.4 (7.3,7.5)	7.4 (7.3,7.5)	-0.6 (-0.7,-0.4)	0.0 (-0.1,0.1)	0.9 (0.7,1.0)	1.0 (0.9,1.2)	-5.4 (-5.5,-5.3)	-2.0 (-2.1,-1.9)
**L**	**L**	**H**	**H**	-4.6 (-4.7,-4.4)	6.4 (6.3,6.5)	6.5 (6.4,6.5)	-0.1 (-0.2,0.1)	-0.2 (-0.3,-0.1)	1.2 (1.1,1.4)	1.2 (1.1,1.3)	-4.3 (-4.4,-4.2)	-4.3 (-4.4,-4.2)
**L**	**H**	**L**	**H**	-4.2 (-4.3,-4.1)	5.4 (5.4,5.5)	7.5 (7.4,7.5)	0.0 (-0.1,0.1)	-0.1 (-0.2,0.0)	0.7 (0.6,0.8)	0.0 (-0.1,0.1)	-1.5 (-1.6,-1.4)	-4.0 (-4.1,-4.0)
**Sensitivity: H H H L**												
**Specificity**				π	Se_1	Se_2	Se_3	Se_4	Sp_1	Sp_2	Sp_3	Sp_4
**H**	**H**	**L**	**L**	-3.2 (-3.3,-3.1)	5.6 (5.5,5.7)	5.5 (5.4,5.6)	-0.1 (-0.2,0.0)	0.0 (-0.1,0.1)	-0.1 (-0.2,0.0)	-0.1 (-0.2,0.0)	-3.2 (-3.3,-3.1)	-1.3 (-1.4,-1.2)
**H**	**H**	**H**	**L**	-3.2 (-3.3,-3.2)	5.5 (5.4,5.5)	5.6 (5.5,5.6)	-0.1 (-0.1,0.0)	0.1 (0.0,0.2)	-0.1 (-0.1,0.0)	0.0 (-0.1,0.1)	-4.9 (-5.0,-4.9)	-1.1 (-1.1,-1.0)
**H**	**H**	**H**	**H**	-3.0 (-3.1,-2.9)	5.2 (5.2,5.3)	5.2 (5.2,5.3)	0.0 (-0.1,0.1)	0.3 (0.2,0.4)	0.0 (0.0,0.1)	0.0 (0.0,0.1)	-4.6 (-4.7,-4.5)	-2.6 (-2.7,-2.6)
**L**	**L**	**L**	**L**	-4.9 (-5.1,-4.8)	7.0 (6.9,7.1)	7.0 (6.9,7.1)	-0.7 (-0.8,-0.6)	-0.1 (-0.2,0.0)	1.2 (1.1,1.4)	1.4 (1.2,1.5)	-4.9 (-5.1,-4.8)	-1.8 (-1.9,-1.7)
**L**	**L**	**H**	**L**	-6.2 (-6.3,-6.1)	7.8 (7.7,7.8)	7.8 (7.7,7.8)	-0.4 (-0.5,-0.3)	0.4 (0.3,0.5)	0.6 (0.5,0.8)	0.6 (0.5,0.7)	-9.1 (-9.2,-9.0)	-1.9 (-2.0,-1.8)
**L**	**L**	**H**	**H**	-4.6 (-4.6,-4.5)	5.8 (5.8,5.9)	5.8 (5.8,5.9)	0.4 (0.3,0.5)	1.0 (0.8,1.1)	0.6 (0.5,0.7)	0.7 (0.6,0.8)	-6.4 (-6.5,-6.3)	-3.3 (-3.4,-3.3)
**L**	**H**	**L**	**H**	-3.8 (-3.9,-3.7)	5.3 (5.3,5.4)	6.6 (6.5,67)	0.0 (-0.1,0.1)	0.1 (-0.1,0.2)	1.1 (1.0,1.2)	0.0 (0.0,0.1)	-3.5 (-3.6,-3.4)	-3.5 (-3.6,-3.4)
**Sensitivity: H H H H**												
**Specificity**				π	Se_1	Se_2	Se_3	Se_4	Sp_1	Sp_2	Sp_3	Sp_4
**H**	**H**	**L**	**L**	-3.1 (-3.2,-3.0)	5.4 (5.4,5.5)	5.4 (5.3,5.4)	0.0 (-0.1,0.0)	0.0 (-0.1,0.0)	0.1 (0.0,0.1)	0.0 (-0.1,0.1)	-2.9 (-3.0,-2.8)	-3.0 (-3.1,-2.9)
**H**	**H**	**H**	**L**	-3.0 (-3.1,-3.0)	5.2 (5.2,5.3)	5.2 (5.2,5.3)	-0.1 (-0.1,0.0)	0.1 (0.1,0.2)	0.1 (0.0,0.1)	0.1 (0.1,0.2)	-4.6 (-4.7,-4.5)	-2.8 (-2.9,-2.7)
**H**	**H**	**H**	**H**	-2.6 (-2.7,-2.6)	4.8 (4.7,4.8)	4.8 (4.7,4.8)	0.1 (0.0,0.1)	0.1 (0.1,0.2)	0.2 (0.1,0.2)	0.2 (0.2,0.3)	-4.0 (-4.0,-3.9)	-4.0 (-4.0,-3.9)
**L**	**L**	**L**	**L**	-5.2 (-5.3,-5.1)	6.7 (6.7,6.8)	6.7 (6.7,6.8)	0.1 (0.0,0.1)	0.1 (0.0,0.2)	0.8 (0.7,0.9)	0.9 (0.8,1.0)	-4.5 (-4.7,-4.4)	-4.6 (-4.7,-4.5)
**L**	**L**	**H**	**L**	-4.8 (-4.9,-4.7)	6.2 (6.1,6.3)	6.2 (6.1,6.2)	0.2 (0.1,0.3)	0.8 (0.7,0.9)	0.8 (0.7,0.9)	0.8 (0.7,0.9)	-6.8 (-6.9,-6.7)	-3.7 (-3.8,-3.6)
**L**	**L**	**H**	**H**	-3.2 (-3.3,-3.1)	4.1 (4.0,4.2)	4.1 (4.0,4.2)	0.7 (0.7,0.8)	0.7 (0.7,0.8)	0.6 (0.5,0.7)	0.6 (0.5,0.7)	-4.1 (-4.2,-4.0)	-4.1 (-4.2,-4.0)
**L**	**H**	**L**	**H**	-3.7 (-3.7,-3.6)	5.2 (5.1,5.2)	6.4 (6.3,6.4)	0.1 (0.1,0.2)	-0.1 (-0.1,0.0)	1.1 (1.0,1.2)	0.1 (0.0,0.2)	-3.2 (-3.3,-3.1)	-5.5 (-5.6,-5.4)
**Sensitivity: L L L L**												
**Specificity**				π	Se_1	Se_2	Se_3	Se_4	Sp_1	Sp_2	Sp_3	Sp_4
**H**	**H**	**L**	**L**	-16.0 (-16.3,-15.7)	24.0 (23.6,24.5)	24.1 (23.7,24.6)	0.1 (-0.1,0.2)	-0.1 (-0.3,0.1)	0.1 (-0.1,0.3)	0.1 (-0.1,0.4)	-4.9 (-5.0,-4.8)	-5.0 (-5.1,-4.9)
**H**	**H**	**H**	**L**	-15.8 (-16.0,-15.7)	23.5 (23.3,23.7)	23.8 (23.6,24.0)	-0.1 (-0.3,0.0)	0.2 (0.1,0.3)	0.1 (0.0,0.2)	0.2 (0.1,0.3)	-12.1 (-12.2,-12.1)	-4.7 (-4.8,-4.6)
**H**	**H**	**H**	**H**	-14.8 (-14.9,-14.7)	21.8 (21.7,22.0)	21.9 (21.7,22.0)	0.7 (0.6,0.9)	0.8 (0.7,0.9)	0.3 (0.3,0.4)	0.4 (0.3,0.5)	-11.0 (-11.1,-11.0)	-11.1 (-11.1,-11.0)
**L**	**L**	**L**	**L**	-21.1 (-21.5,-20.7)	32.1 (31.7,32.6)	31.9 (31.5,32.4)	-2.1 (-2.4,-1.8)	-1.9 (-2.2,-1.5)	6.8 (6.5,7.1)	6.6 (6.3,6.8)	-6.7 (-6.9,-6.5)	-6.6 (-6.8,-6.5)
**L**	**L**	**H**	**L**	-25.3 (-25.4,-25.1)	36.0 (35.9,36.1)	36.0 (35.8,36.1)	-4.6 (-4.8,-4.4)	-1.0 (-1.2,-0.8)	5.0 (4.9,5.1)	5.1 (5.0,5.2)	-18.4 (-18.5,-18.3)	-7.0 (-7.1,-6.9)

**L**	**L**	**H**	**H**	-24.9 (-25.1,-24.7)	33.4 (33.1,33.7)	33.5 (33.2,33.8)	-0.9 (-1.2,-0.7)	-1.0 (-1.3,-0.8)	4.5 (4.3,4.6)	4.5 (4.3,4.6)	-17.0 (-17.2,-16.9)	-17.0 (-17.1,-16.8)
**L**	**H**	**L**	**H**	-20.7 (-20.8,-20.5)	23.2 (23.0,23.3)	36.7 (36.5,36.8)	-0.1 (-0.2,0.1)	-0.4 (-0.6,-0.3)	3.7 (3.6,3.8)	0.5 (0.3,0.6)	-5.9 (-5.9,-5.8)	-14.9 (-14.9,-14.8)
**Sensitivity: L L H L**												
**Specificity**				π	Se_1	Se_2	Se_3	Se_4	Sp_1	Sp_2	Sp_3	Sp_4
**H**	**H**	**L**	**L**	-16.0 (-16.1,-15.9)	23.6 (23.4,23.8)	23.8 (23.6,24.0)	-0.1 (-0.1,0.0)	0.4 (0.3,0.5)	0.0 (-0.1,0.1)	0.1 (0.0,0.2)	-12.1 (-12.2,-12.0)	-4.7 (-4.8,-4.6)
**H**	**H**	**H**	**L**	-15.9 (-16.0,-15.8)	23.5 (23.3,23.6)	23.4 (23.3,23.5)	-0.1 (-0.2,0.0)	0.9 (0.8,1.0)	0.0 (-0.1,0.1)	0.0 (-0.1,0.1)	-19.4 (-19.5,-19.3)	-4.3 (-4.4,-4.2)
**H**	**H**	**H**	**H**	-12.1 (-12.3,-11.9)	17.2 (17.0,17.5)	17.3 (17.1,17.6)	0.4 (0.3,0.4)	2.6 (2.4,2.7)	0.6 (0.5,0.7)	0.6 (0.6,0.7)	-15.4 (-15.6,-15.2)	-8.1 (-8.2,-7.9)
**L**	**L**	**L**	**L**	-25.2 (-25.3,-25.1)	35.9 (35.7,36.0)	35.9 (35.7,36.0)	-4.5 (-4.7,-4.3)	-0.8 (-0.9,-0.6)	5.0 (4.8,5.1)	5.0 (4.9,5.1)	-18.1 (-18.2,-18.0)	-7.0 (-7.0,-6.9)
**L**	**L**	**H**	**L**	-26.9 (-27.0,-26.8)	37.4 (37.3,37.5)	37.4 (37.4,37.5)	-5.7 (-5.9,-5.4)	1.0 (0.8,1.2)	4.1 (4.0,4.2)	4.2 (4.1,4.3)	-29.7 (-29.8,-29.6)	-6.8 (-6.9,-6.7)
**L**	**L**	**H**	**H**	-7.2 (-7.5,-6.9)	5.2 (4.9,5.5)	5.2 (4.9,5.5)	1.3 (1.1,1.5)	5.9 (5.8,6.1)	1.1 (1.0,1.2)	1.2 (1.0,1.3)	-9.0 (-9.3,-8.7)	-1.8 (-2.0,-1.6)
**L**	**H**	**L**	**H**	-19.0 (-19.2,-18.9)	22.3 (22.1,22.5)	31.3 (31.1,31.6)	0.4 (0.3,0.5)	0.6 (0.4,0.7)	4.4 (4.3,4.5)	0.1 (0.0,0.2)	-13.6 (-13.7,-13.5)	-13.5 (-13.6,-13.4)
**Sensitivity: L L H H**												
**Specificity**				π	Se_1	Se_2	Se_3	Se_4	Sp_1	Sp_2	Sp_3	Sp_4
**H**	**H**	**L**	**L**	-15.0 (-15.1,-14.9)	22.3 (22.1,22.4)	22.2 (22.0,22.3)	0.2 (0.1,0.3)	0.2 (0.2,0.3)	0.5 (0.5,0.6)	0.5 (0.4,0.6)	-11.4 (-11.5,-11.3)	-11.4 (-11.5,-11.3)
**H**	**H**	**H**	**L**	-13.5 (-13.7,-13.4)	19.9 (19.7,20.0)	19.9 (19.8,20.1)	0.2 (0.1,0.2)	1.1 (1.0,1.2)	0.6 (0.5,0.7)	0.6 (0.6,0.7)	-16.9 (-17.0,-16.8)	-9.9 (-10.0,-9.8)
**H**	**H**	**H**	**H**	-2.2 (-2.3,-2.1)	2.8 (2.7,3.0)	2.9 (2.8,3.0)	0.8 (0.8,0.9)	0.8 (0.8,0.9)	0.6 (0.5,0.6)	0.6 (0.5,0.6)	-2.4 (-2.5,-2.3)	-2.5 (-2.6,-2.4)
**L**	**L**	**L**	**L**	-26.2 (-26.4,-26.1)	34.3 (34.1,34.5)	34.2 (34.0,34.4)	0.7 (0.5,0.9)	0.7 (0.6,0.9)	3.7 (3.6,3.8)	3.8 (3.7,3.9)	-17.0 (-17.1,-16.9)	-17.0 (-17.1,-16.9)
**L**	**L**	**H**	**L**	-6.2 (-6.8,-5.5)	6.5 (5.7,7.3)	6.4 (5.6,7.2)	0.1 (-0.2,0.4)	2.9 (2.7,3.0)	1.4 (1.3,1.6)	1.5 (1.3,1.6)	-7.9 (-8.5,-7.2)	-2.7 (-3.1,-2.2)
**L**	**L**	**H**	**H**	-0.7 (-0.9,-0.6)	0.8 (0.7,0.9)	0.8 (0.7,0.9)	0.5 (0.4,0.7)	0.5 (0.3,0.6)	0.5 (0.4,0.6)	0.4 (0.3,0.6)	-0.6 (-0.8,-0.5)	-0.7 (-0.8,-0.6)
**L**	**H**	**L**	**H**	15.3 (-15.6,-14.9)	18.4 (18.0,18.8)	23.5 (23.0,24.1)	1.6 (1.5,1.7)	0.5 (0.4,0.6)	4.5 (4.4,4.6)	0.1 (0.0,0.2)	-10.5 (-10.8,-10.2)	-18.1 (-18.5,-17.7)

Table v.5 Coverages of the 95% CrIs for each model parameter

**Sensitivity: H H L L**												
**Specificity**				π	Se_1	Se_2	Se_3	Se_4	Sp_1	Sp_2	Sp_3	Sp_4
**H**	**H**	**L**	**L**	73.8 (70.8,76.8)	35.5 (32.3,38.7)	38.9 (35.6,42.2)	94.8 (93.3,96.3)	96.7 (95.5,97.9)	95.4 (94.0,96.8)	96.7 (95.5,97.9)	86.8 (84.6,89.1)	89.3 (87.2,91.4)
**H**	**H**	**H**	**L**	39.3 (36.3,42.4)	1.8 (1.0,2.6)	1.7 (0.9,2.5)	94.2 (92.7,95.6)	95.2 (93.9,96.5)	96.0 (94.8,97.2)	93.8 (92.3,95.3)	17.8 (15.4,20.2)	86.4 (84.3,88.5)
**H**	**H**	**H**	**H**	28.9 (26.1,31.7)	0.2 (0.0,0.5)	0.3 (0.0,0.6)	95.2 (93.9,96.5)	95.5 (94.2,96.8)	95.5 (94.2,96.8)	94.7 (93.3,96.1)	20.7 (18.2,23.2)	21.3 (18.8,23.8)

**L**	**L**	**L**	**L**	96.6 (95.2,98.0)	56.6 (52.9,60.4)	57.7 (54.0,61.4)	94.2 (92.5,96.0)	94.1 (92.3,95.9)	89.4 (87.1,91.7)	90.9 (88.7,93.0)	87.8 (85.3,90.2)	88.9 (86.6,91.3)
**L**	**L**	**H**	**L**	35.3 (32.3,38.3)	1.0 (0.4,1.7)	0.8 (0.3,1.4)	93.3 (91.7,94.9)	94.4 (93.0,95.9)	93.8 (92.3,95.3)	94.5 (93.1,96.0)	3.7 (2.5,4.9)	78.4 (75.8,80.9)
**L**	**L**	**H**	**H**	36.3 (33.3,39.3)	0.4 (0.0,0.8)	0.3 (0.0,0.6)	95.4 (94.1,96.7)	94.8 (93.4,96.2)	91.0 (89.3,92.8)	92.1 (90.4,93.7)	11.5 (9.5,13.4)	11.0 (9.0,12.9)
**L**	**H**	**L**	**H**	29.3 (26.5,32.1)	1.0 (0.4,1.6)	1.5 (0.8,2.3)	96.6 (95.4,97.7)	94.0 (92.5,95.4)	94.1 (92.6,95.5)	95.8 (94.5,97.0)	82.9 (80.5,85.2)	9.0 (7.2,10.7)
**Sensitivity: H H H L**												
**Specificity**				π	Se_1	Se_2	Se_3	Se_4	Sp_1	Sp_2	Sp_3	Sp_4
**H**	**H**	**L**	**L**	40.2 (37.2,43.3)	1.4 (0.7,2.1)	1.9 (1.1,2.8)	95.8 (94.5,97.0)	96.0 (94.8,97.2)	95.6 (94.3,96.9)	95.8 (94.5,97.0)	50.2 (47.0,53.3)	86.4 (84.2,88.5)
**H**	**H**	**H**	**L**	24.5 (21.8,27.2)	0.1 (0.0,0.3)	0.0 (0.0,0.0)	95.5 (94.2,96.8)	94.9 (93.5,96.3)	94.5 (93.1,95.9)	95.4 (94.1,96.7)	0.8 (0.2,1.4)	89.3 (87.4,91.2)
**H**	**H**	**H**	**H**	30.7 (27.8,33.6)	0.0 (0.0,0.0)	0.0 (0.0,0.0)	94.9 (93.5,96.3)	94.8 (93.4,96.2)	95.5 (94.2,96.8)	95.8 (94.6,97.0)	1.6 (0.8,2.4)	26.5 (23.8,29.2)
**L**	**L**	**L**	**L**	52.7 (49.6,55.9)	4.8 (3.5,6.2)	3.9 (2.6,5.1)	93.3 (91.7,94.9)	94.2 (92.8,95.7)	94.0 (92.5,95.5)	92.1 (90.3,93.8)	29.5 (26.6,32.4)	81.4 (78.9,83.8)
**L**	**L**	**H**	**L**	10.1 (8.2,12.0)	0.0 (0.0,0.0)	0.0 (0.0,0.0)	95.1 (93.8,96.5)	95.6 (94.4,96.9)	93.6 (92.1,95.1)	94.3 (92.9,95.8)	0.0 (0.0,0.0)	77.3 (74.7,79.9)
**L**	**L**	**H**	**H**	18.0 (15.6,20.4)	0.3 (0.0,0.6)	0.4 (0.0,0.8)	94.9 (93.5,96.3)	91.7 (90.0,93.4)	92.1 (90.4,93.8)	92.8 (91.2,94.4)	1.2 (0.5,1.9)	22.6 (20.0,25.2)
**L**	**H**	**L**	**H**	28.2 (25.4,31.0)	0.1 (0.0,0.3)	1.1 (0.5,1.7)	93.8 (92.3,95.3)	95.1 (93.8,96.4)	91.2 (89.4,93.0)	96.7 (95.6,97.8)	44.0 (40.9,47.1)	13.6 (11.5,15.7)
**Sensitivity: H H H H**												
**Specificity**				π	Se_1	Se_2	Se_3	Se_4	Sp_1	Sp_2	Sp_3	Sp_4
**H**	**H**	**L**	**L**	34.3 (31.4,37.2)	0.1 (0.0,0.3)	0.4 (0.0,0.8)	95.1 (93.8,96.4)	95.5 (94.2,96.8)	94.9 (93.5,96.3)	95.1 (93.8,96.4)	54.5 (51.4,57.6)	54.4 (51.3,57.5)
**H**	**H**	**H**	**L**	27.4 (24.6,30.2)	0.0 (0.0,0.0)	0.0 (0.0,0.0)	92.7 (91.1,94.3)	94.4 (93.0,95.8)	95.0 (93.6,96.4)	94.9 (93.5,96.3)	1.2 (0.5,1.9)	55.4 (52.3,58.5)
**H**	**H**	**H**	**H**	39.4 (36.4,42.4)	0.5 (0.1,0.9)	0.3 (0.0,0.6)	95.5 (94.2,96.8)	96.5 (95.4,97.6)	95.0 (93.6,96.4)	94.7 (93.3,96.1)	4.8 (3.5,6.1)	5.6 (4.2,7.0)
**L**	**L**	**L**	**L**	29.7 (26.9,32.6)	0.7 (0.2,1.2)	0.8 (0.3,1.4)	96.0 (94.8,97.3)	95.0 (93.7,96.4)	95.5 (94.2,96.8)	92.2 (90.5,93.9)	33.6 (30.7,36.6)	31.4 (28.5,34.3)
**L**	**L**	**H**	**L**	15.1 (12.9,17.3)	0.0 (0.0,0.0)	0.1 (0.0,0.3)	94.7 (93.3,96.1)	89.2 (87.3,91.1)	93.1 (91.5,94.7)	91.7 (90.0,93.4)	0.1 (0.0,0.3)	43.4 (40.3,46.5)
**L**	**L**	**H**	**H**	39.1 (36.1,42.2)	14.5 (12.3,16.7)	12.3 (10.3,14.3)	91.0 (89.2,92.8)	89.5 (87.6,91.4)	92.5 (90.9,94.1)	94.1 (92.6,95.6)	20.1 (17.6,22.6)	22.5 (19.9,25.1)
**L**	**H**	**L**	**H**	21.4 (18.9,23.9)	0.0 (0.0,0.0)	0.0 (0.0,0.0)	95.8 (94.6,97.0)	94.7 (93.3,96.1)	89.2 (87.3,91.1)	95.6 (94.3,96.9)	48.2 (45.1,51.3)	0.9 (0.3,1.5)
**Sensitivity: L L L L**												
**Specificity**				π	Se_1	Se_2	Se_3	Se_4	Sp_1	Sp_2	Sp_3	Sp_4
**H**	**H**	**L**	**L**	0.6 (0.0,1.2)	0.0 (0.0,0.0)	0.0 (0.0,0.0)	96.1 (94.5,97.8)	95.0 (93.2,96.9)	95.8 (94.1,97.5)	94.1 (92.1,96.1)	12.3 (9.6,15.1)	9.6 (7.1,12.1)
**H**	**H**	**H**	**L**	0.0 (0.0,0.0)	0.0 (0.0,0.0)	0.0 (0.0,0.0)	95.2 (93.9,96.6)	94.5 (93.0,95.9)	95.3 (94.0,96.6)	95.0 (93.6,96.4)	0.0 (0.0,0.0)	11.6 (9.5,13.6)
**H**	**H**	**H**	**H**	0.0 (0.0,0.0)	0.0 (0.0,0.0)	0.0 (0.0,0.0)	93.7 (92.2,95.2)	93.6 (92.1,95.1)	94.6 (93.2,96.0)	94.0 (92.5,95.5)	0.0 (0.0,0.0)	0.0 (0.0,0.0)
**L**	**L**	**L**	**L**	15.8 (11.6,19.9)	0.0 (0.0,0.0)	0.0 (0.0,0.0)	90.3 (86.9,93.6)	91.9 (88.9,95.0)	9.4 (6.1,12.7)	9.4 (6.1,12.7)	2.3 (0.6,4.1)	0.3 (0.0,1.0)
**L**	**L**	**H**	**L**	0.0 (0.0,0.0)	0.0 (0.0,0.0)	0.0 (0.0,0.0)	71.7 (68.8,74.7)	92.8 (91.1,94.5)	18.7 (16.1,21.2)	16.0 (13.6,18.4)	0.0 (0.0,0.0)	0.2 (0.0,0.5)
**L**	**L**	**H**	**H**	0.8 (0.2,1.4)	0.7 (0.1,1.2)	0.7 (0.1,1.2)	91.2 (89.3,93.0)	92.0 (90.2,93.7)	28.8 (25.8,31.8)	30.5 (27.5,33.5)	1.0 (0.4,1.7)	1.2 (0.5,2.0)
**L**	**H**	**L**	**H**	0.0 (0.0,0.0)	0.0 (0.0,0.0)	0.0 (0.0,0.0)	94.2 (92.7,95.7)	94.7 (93.3,96.2)	27.9 (25.0,30.8)	95.4 (94.0,96.8)	2.3 (1.3,3.3)	0.0 (0.0,0.0)
**Sensitivity: L L H L**												
**Specificity**				π	Se_1	Se_2	Se_3	Se_4	Sp_1	Sp_2	Sp_3	Sp_4
**H**	**H**	**L**	**L**	0.0 (0.0,0.0)	0.0 (0.0,0.0)	0.0 (0.0,0.0)	94.4 (93.0,95.9)	95.4 (94.1,96.7)	95.2 (93.8,96.5)	95.4 (94.0,96.7)	0.0 (0.0,0.0)	11.4 (9.4,13.4)
**H**	**H**	**H**	**L**	0.0 (0.0,0.0)	0.0 (0.0,0.0)	0.0 (0.0,0.0)	95.5 (94.2,96.8)	92.6 (91.0,94.2)	94.7 (93.3,96.1)	95.4 (94.1,96.7)	0.0 (0.0,0.0)	16.7 (14.4,19.0)
**H**	**H**	**H**	**H**	0.4 (0.0,0.8)	0.2 (0.0,0.5)	0.1 (0.0,0.3)	94.8 (93.4,96.2)	73.5 (70.8,76.3)	90.9 (89.1,92.7)	92.1 (90.4,93.8)	0.1 (0.0,0.3)	3.5 (2.3,4.6)
**L**	**L**	**L**	**L**	0.0 (0.0,0.0)	0.0 (0.0,0.0)	0.0 (0.0,0.0)	65.9 (62.8,69.0)	94.8 (93.4,96.3)	18.5 (16.0,21.1)	16.2 (13.7,18.6)	0.0 (0.0,0.0)	0.0 (0.0,0.0)
**L**	**L**	**H**	**L**	0.0 (0.0,0.0)	0.0 (0.0,0.0)	0.0 (0.0,0.0)	65.7 (62.7,68.6)	91.9 (90.2,93.6)	31.1 (28.2,34.0)	29.1 (26.2,31.9)	0.0 (0.0,0.0)	0.2 (0.0,0.5)
**L**	**L**	**H**	**H**	42.1 (38.7,45.6)	42.7 (39.2,46.1)	44.2 (40.7,47.7)	94.0 (92.3,95.7)	44.7 (41.2,48.2)	90.7 (88.6,92.7)	89.1 (87.0,91.3)	23.9 (20.9,26.9)	84.5 (82.0,87.1)
**L**	**H**	**L**	**H**	0.0 (0.0,0.0)	0.0 (0.0,0.0)	0.0 (0.0,0.0)	94.4 (93.0,95.9)	95.2 (93.8,96.5)	17.9 (15.5,20.4)	95.3 (93.9,96.6)	0.0 (0.0,0.0)	0.0 (0.0,0.0)
**Sensitivity: L L H H**												

Specificity				π	Se_1	Se_2	Se_3	Se_4	Sp_1	Sp_2	Sp_3	Sp_4
**H**	**H**	**L**	**L**	0.0 (0.0,0.0)	0.0 (0.0,0.0)	0.0 (0.0,0.0)	95.7 (94.4,97.0)	94.8 (93.4,96.2)	92.7 (91.1,94.3)	92.8 (91.2,94.4)	0.0 (0.0,0.0)	0.0 (0.0,0.0)
**H**	**H**	**H**	**L**	0.0 (0.0,0.0)	0.0 (0.0,0.0)	0.0 (0.0,0.0)	94.1 (92.6,95.6)	86.8 (84.7,88.9)	91.6 (89.9,93.3)	91.0 (89.2,92.8)	0.0 (0.0,0.0)	0.0 (0.0,0.0)
**H**	**H**	**H**	**H**	63.5 (60.5,66.5)	62.6 (59.6,65.6)	62.0 (59.0,65.0)	89.1 (87.2,91.0)	89.1 (87.2,91.0)	91.8 (90.1,93.5)	92.1 (90.4,93.8)	54.4 (51.3,57.5)	54.2 (51.1,57.3)
**L**	**L**	**L**	**L**	0.0 (0.0,0.0)	0.0 (0.0,0.0)	0.0 (0.0,0.0)	93.4 (91.8,95.0)	94.0 (92.5,95.5)	43.2 (40.0,46.4)	41.3 (38.1,44.4)	0.0 (0.0,0.0)	0.0 (0.0,0.0)
**L**	**L**	**H**	**L**	74.9 (71.5,78.3)	64.7 (60.9,68.4)	66.9 (63.2,70.6)	94.8 (93.1,96.6)	70.5 (66.9,74.1)	83.6 (80.7,86.6)	84.3 (81.4,87.2)	57.2 (53.3,61.1)	82.8 (79.8,85.8)
**L**	**L**	**H**	**H**	94.4 (92.8,95.9)	92.1 (90.3,93.9)	92.0 (90.2,93.8)	94.8 (93.4,96.3)	93.3 (91.7,95.0)	94.5 (93.0,96.0)	93.6 (92.0,95.2)	93.8 (92.2,95.4)	94.5 (93.0,96.0)
**L**	**H**	**L**	**H**	7.3 (5.5,9.1)	4.5 (3.1,6.0)	7.3 (5.5,9.1)	78.1 (75.2,80.9)	94.2 (92.6,95.9)	15.8 (13.3,18.3)	94.7 (93.2,96.3)	13.3 (10.9,15.6)	4.8 (3.3,6.2)

vi. Setting 6: $\pi = 0.5, \omega = 0.5, n_{obs} = 500$

Table vi.1 Number of converged data sets for different sensitivity-specificity combinations

				**Number of converged data sets**
**Specificity**				**Sensitivity: H H L L**
**H**	**H**	**L**	**L**	953
**H**	**H**	**H**	**L**	1000
**H**	**H**	**H**	**H**	1000
**L**	**L**	**L**	**L**	741
**L**	**L**	**H**	**L**	984
**L**	**L**	**H**	**H**	992
**L**	**H**	**L**	**H**	997
**Specificity**				**Sensitivity: H H H L**
**H**	**H**	**L**	**L**	998
**H**	**H**	**H**	**L**	1000
**H**	**H**	**H**	**H**	1000
**L**	**L**	**L**	**L**	973
**L**	**L**	**H**	**L**	996
**L**	**L**	**H**	**H**	999
**L**	**H**	**L**	**H**	1000
**Specificity**				**Sensitivity: H H H H**
**H**	**H**	**L**	**L**	1000
**H**	**H**	**H**	**L**	1000
**H**	**H**	**H**	**H**	1000
**L**	**L**	**L**	**L**	995
**L**	**L**	**H**	**L**	999
**L**	**L**	**H**	**H**	1000
**L**	**H**	**L**	**H**	1000

Specificity				Sensitivity: L L L L
H	H	L	L	808
H	H	H	L	950
H	H	H	H	985
L	L	L	L	487
L	L	H	L	747
L	L	H	H	731
L	H	L	H	927
Specificity				Sensitivity: L L H L
H	H	L	L	969
H	H	H	L	999
H	H	H	H	977
L	L	L	L	770
L	L	H	L	955
L	L	H	H	734
L	H	L	H	953
Specificity				Sensitivity: L L H H
H	H	L	L	994
H	H	H	L	992
H	H	H	H	989
L	L	L	L	818
L	L	H	L	622
L	L	H	H	947
L	H	L	H	843

Table vi.2 Percentages of the time 95% credible intervals (CrIs) for residual correlations did not included '0' for any pairs, and percentages of the time that the overall χ^2 or G^2 statistic indicated a lack of overall fit

Sensitivity: H H L L						
Specificity				Res. Cor.	Overall G^2	Overall χ^2
H	H	L	L	56.5 (53.2,59.6)	6.3 (4.8,8.0)	4.8 (3.6,6.4)
H	H	H	L	22.9 (20.3,25.6)	6.5 (5.1,8.2)	5.6 (4.3,7.2)
H	H	H	H	29.6 (26.8,32.5)	19.9 (17.5,22.5)	25.4 (22.7,28.2)
L	L	L	L	48.3 (44.7,52.0)	10.4 (8.3,12.8)	9.4 (7.4,11.8)
L	L	H	L	20.1 (17.7,22.8)	14.3 (12.2,16.7)	13.1 (11.1,15.4)
L	L	H	H	19.8 (17.3,22.4)	44.9 (41.7,48.0)	53.7 (50.6,56.9)
L	H	L	H	29.6 (26.8,32.5)	8.4 (6.8,10.3)	7.0 (5.5,8.8)
Sensitivity: H H H L						
Specificity				Res. Cor.	Overall G^2	Overall χ^2
H	H	L	L	23.0 (20.5,25.8)	6.7 (5.2,8.4)	5.6 (4.3,7.2)

**H**	**H**	**H**	**L**	3.9 (2.8,5.3)	8.1 (6.5,10.0)	6.9 (5.4,8.7)
**H**	**H**	**H**	**H**	26.6 (23.9,29.5)	38.0 (35.0,41.1)	51.2 (48.1,54.3)
**L**	**L**	**L**	**L**	23.5 (20.9,26.3)	13.5 (11.4,15.8)	12.3 (10.3,14.6)
**L**	**L**	**H**	**L**	13.9 (11.8,16.2)	19.8 (17.3,22.4)	18.1 (15.7,20.6)
**L**	**L**	**H**	**H**	23.7 (21.1,26.5)	69.9 (66.9,72.7)	77.6 (74.9,80.1)
**L**	**H**	**L**	**H**	29.2 (26.4,32.1)	16.3 (14.1,18.7)	20.7 (18.2,23.3)
**Sensitivity: H H H H**						
**Specificity**				**Res. Cor.**	**Overall G^2**	**Overall χ^2**
**H**	**H**	**L**	**L**	33.1 (30.2,36.1)	8.4 (6.8,10.3)	7.8 (6.2,9.6)
**H**	**H**	**H**	**L**	34.4 (31.5,37.4)	17.9 (15.6,20.4)	25.4 (22.7,28.2)
**H**	**H**	**H**	**H**	51.9 (48.8,55.0)	62.4 (59.3,65.4)	84.4 (82.0,86.6)
**L**	**L**	**L**	**L**	30.5 (27.8,33.6)	23.5 (20.9,26.3)	23.6 (21.0,26.4)
**L**	**L**	**H**	**L**	46.2 (43.1,49.4)	57.1 (53.9,60.2)	61.6 (58.5,64.6)
**L**	**L**	**H**	**H**	52.8 (49.7,55.9)	93.6 (91.6,94.8)	97.3 (96.1,98.2)
**L**	**H**	**L**	**H**	43.2 (40.1,46.3)	36.9 (33.9,40.0)	43.7 (40.6,46.8)
**Sensitivity: L L L L**						
**Specificity**				**Res. Cor.**	**Overall G^2**	**Overall χ^2**
**H**	**H**	**L**	**L**	70.0 (66.8,73.2)	8.8 (6.9,11.0)	7.5 (5.8,9.6)
**H**	**H**	**H**	**L**	60.7 (57.5,63.9)	18.0 (15.6,20.6)	16.8 (14.5,19.4)
**H**	**H**	**H**	**H**	91.9 (90.0,93.5)	87.9 (85.7,89.9)	89.6 (87.6,91.5)
**L**	**L**	**L**	**L**	85.6 (82.2,88.6)	21.6 (18.0,25.5)	21.8 (18.2,25.7)
**L**	**L**	**H**	**L**	83.5 (80.7,86.1)	54.5 (50.8,58.1)	54.4 (50.7,58.0)
**L**	**L**	**H**	**H**	93.6 (91.5,95.2)	99.3 (98.4,99.8)	99.2 (98.2,99.7)
**L**	**H**	**L**	**H**	79.7 (77.0,82.3)	41.6 (38.4,44.9)	40.6 (37.4,43.8)
**Sensitivity: L L H L**						
**Specificity**				**Res. Cor.**	**Overall G^2**	**Overall χ^2**
**H**	**H**	**L**	**L**	64.1 (61.0,67.1)	14.9 (12.7,17.3)	13.0 (10.9,15.3)
**H**	**H**	**H**	**L**	67.0 (64.0,69.9)	23.1 (29.2,35.1)	30.2 (27.4,33.2)
**H**	**H**	**H**	**H**	92.4 (90.6,94.0)	96.3 (94.9,97.4)	99.7 (99.1,99.9)
**L**	**L**	**L**	**L**	89.0 (86.5,91.1)	51.6 (48.0,55.1)	51.8 (48.2,55.4)
**L**	**L**	**H**	**L**	95.2 (93.6,96.5)	84.3 (81.8,86.5)	83.9 (81.4,86.2)
**L**	**L**	**H**	**H**	98.5 (97.3,99.2)	100.0 (99.5,100.0)	100.0 (99.5,100.0)
**L**	**H**	**L**	**H**	98.1 (97.0,98.9)	87.9 (85.7,89.9)	96.1 (94.7,97.3)
**Sensitivity: L L H H**						
**Specificity**				**Res. Cor.**	**Overall G^2**	**Overall χ^2**
**H**	**H**	**L**	**L**	99.0 (98.2,99.5)	59.4 (56.2,62.4)	65.6 (62.5,68.5)
**H**	**H**	**H**	**L**	99.3 (98.6,99.7)	88.5 (86.4,90.4)	99.3 (98.6,99.7)
**H**	**H**	**H**	**H**	99.8 (99.3,100.0)	93.2 (91.5,94.7)	100.0 (99.6,100.0)
**L**	**L**	**L**	**L**	97.9 (96.7,98.8)	98.7 (97.6,99.3)	98.7 (97.6,99.3)
**L**	**L**	**H**	**L**	98.9 (97.7,99.5)	99.5 (98.6,99.9)	100.0 (99.4,100.0)
**L**	**L**	**H**	**H**	100.0 (99.6,100.0)	99.8 (99.2,100.0)	100.0 (99.6,100.0)

L	H	L	H	97.2 (95.8,98.2)	96.3 (94.8,97.5)	100.0 (99.6,100.0)

Table vi.3 Percentages of the time 95% CrIs for residual correlations did not included '0' for each pair, and percentages of the time the pairwise χ^2 or G^2 statistic for each pair indicated a lack of pairwise fit

**Sensitivity: H H L L**										
**Specificity**				**Tool**	**T_1&T_2**	**T_1&T_3**	**T_1&T_4**	**T_2&T_3**	**T_2&T_4**	**T_3&T_4**
**H**	**H**	**L**	**L**	**Res. Cor.**	0.0 (0.0,0.4)	0.0 (0.0,0.4)	0.0 (0.0,0.4)	0.1 (0.0,0.6)	0.0 (0.0,0.4)	56.5 (53.2,59.6)
				**Pairwise G^2**	0.0 (0.0,0.4)	0.0 (0.0,0.4)	0.0 (0.0,0.4)	0.0 (0.0,0.4)	0.0 (0.0,0.4)	4.1 (2.9,5.6)
				**Pairwise χ^2**	0.0 (0.0,0.4)	0.0 (0.0,0.4)	0.0 (0.0,0.4)	0.0 (0.0,0.4)	0.0 (0.0,0.4)	4.1 (2.9,5.6)
**H**	**H**	**H**	**L**	**Res. Cor.**	0.0 (0.0,0.4)	0.0 (0.0,0.4)	0.1 (0.0,0.6)	0.0 (0.0,0.4)	0.2 (0.0,0.7)	22.7 (20.1,25.4)
				**Pairwise G^2**	0.0 (0.0,0.4)	0.0 (0.0,0.4)	0.0 (0.0,0.4)	0.0 (0.0,0.4)	0.1 (0.0,0.6)	3.1 (2.1,4.4)
				**Pairwise χ^2**	0.0 (0.0,0.4)	0.0 (0.0,0.4)	0.0 (0.0,0.4)	0.0 (0.0,0.4)	0.1 (0.0,0.6)	3.1 (2.1,4.4)
**H**	**H**	**H**	**H**	**Res. Cor.**	0.0 (0.0,0.4)	0.0 (0.0,0.4)	0.0 (0.0,0.4)	0.0 (0.0,0.4)	0.0 (0.0,0.4)	29.6 (26.8,32.5)
				**Pairwise G^2**	0.0 (0.0,0.4)	0.0 (0.0,0.4)	0.0 (0.0,0.4)	0.0 (0.0,0.4)	0.0 (0.0,0.4)	8.3 (6.7,10.2)
				**Pairwise χ^2**	0.0 (0.0,0.4)	0.0 (0.0,0.4)	0.0 (0.0,0.4)	0.0 (0.0,0.4)	0.0 (0.0,0.4)	8.1 (6.5,10.0)
**L**	**L**	**L**	**L**	**Res. Cor.**	0.0 (0.0,0.5)	1.8 (0.9,3.0)	0.8 (0.3,1.8)	0.8 (0.3,1.8)	1.1 (0.5,2.1)	46.6 (42.9,50.2)
				**Pairwise G^2**	0.0 (0.0,0.5)	0.0 (0.0,0.5)	0.1 (0.0,0.7)	0.0 (0.0,0.5)	0.0 (0.0,0.5)	3.2 (2.1,4.8)
				**Pairwise χ^2**	0.0 (0.0,0.5)	0.0 (0.0,0.5)	0.1 (0.0,0.7)	0.0 (0.0,0.5)	0.0 (0.0,0.5)	3.2 (2.1,4.8)
**L**	**L**	**H**	**L**	**Res. Cor.**	0.0 (0.0,0.4)	0.0 (0.0,0.4)	3.5 (2.4,4.8)	0.0 (0.0,0.4)	3.6 (2.5,4.9)	16.0 (13.7,18.4)
				**Pairwise G^2**	0.0 (0.0,0.4)	0.0 (0.0,0.4)	0.3 (0.1,0.9)	0.0 (0.0,0.4)	0.2 (0.0,0.7)	2.9 (2.0,4.2)
				**Pairwise χ^2**	0.0 (0.0,0.4)	0.0 (0.0,0.4)	0.3 (0.1,0.9)	0.0 (0.0,0.4)	0.2 (0.0,0.7)	2.9 (2.0,4.2)
**L**	**L**	**H**	**H**	**Res. Cor.**	0.2 (0.0,0.7)	0.2 (0.0,0.7)	0.0 (0.0,0.4)	0.0 (0.0,0.4)	0.0 (0.0,0.4)	19.5 (17.0,22.1)
				**Pairwise G^2**	0.1 (0.0,0.6)	0.0 (0.0,0.4)	0.0 (0.0,0.4)	0.0 (0.0,0.4)	0.0 (0.0,0.4)	10.6 (8.7,12.7)
				**Pairwise χ^2**	0.1 (0.0,0.6)	0.0 (0.0,0.4)	0.0 (0.0,0.4)	0.0 (0.0,0.4)	0.0 (0.0,0.4)	10.3 (8.5,12.3)
**L**	**H**	**L**	**H**	**Res. Cor.**	0.0 (0.0,0.4)	9.6 (7.9,11.6)	0.1 (0.0,0.6)	0.0 (0.0,0.4)	0.0 (0.0,0.4)	22.1 (19.5,24.8)
				**Pairwise G^2**	0.0 (0.0,0.4)	1.0 (0.5,1.8)	0.0 (0.0,0.4)	0.0 (0.0,0.4)	0.0 (0.0,0.4)	3.3 (2.3,4.6)
				**Pairwise χ^2**	0.0 (0.0,0.4)	1.0 (0.5,1.8)	0.0 (0.0,0.4)	0.0 (0.0,0.4)	0.0 (0.0,0.4)	3.3 (2.3,4.6)
**Sensitivity: H H H L**										
**Specificity**				**Tool**	**T_1&T_2**	**T_1&T_3**	**T_1&T_4**	**T_2&T_3**	**T_2&T_4**	**T_3&T_4**
**H**	**H**	**L**	**L**	**Res. Cor.**	0.0 (0.0,0.4)	0.0 (0.0,0.4)	0.0 (0.0,0.4)	0.0 (0.0,0.4)	0.0 (0.0,0.4)	23.0 (20.5,25.8)
				**Pairwise G^2**	0.0 (0.0,0.4)	0.0 (0.0,0.4)	0.0 (0.0,0.4)	0.0 (0.0,0.4)	0.0 (0.0,0.4)	2.1 (1.3,3.2)
				**Pairwise χ^2**	0.0 (0.0,0.4)	0.0 (0.0,0.4)	0.0 (0.0,0.4)	0.0 (0.0,0.4)	0.0 (0.0,0.4)	2.1 (1.3,3.2)
**H**	**H**	**H**	**L**	**Res. Cor.**	0.0 (0.0,0.4)	0.0 (0.0,0.4)	0.2 (0.0,0.7)	0.0 (0.0,0.4)	0.1 (0.0,0.6)	3.8 (2.7,5.2)
				**Pairwise G^2**	0.0 (0.0,0.4)	0.0 (0.0,0.4)	0.0 (0.0,0.4)	0.0 (0.0,0.4)	0.0 (0.0,0.4)	0.8 (0.3,1.6)
				**Pairwise χ^2**	0.0 (0.0,0.4)	0.0 (0.0,0.4)	0.0 (0.0,0.4)	0.0 (0.0,0.4)	0.0 (0.0,0.4)	0.8 (0.3,1.6)
**H**	**H**	**H**	**H**	**Res. Cor.**	0.0 (0.0,0.4)	0.0 (0.0,0.4)	0.0 (0.0,0.4)	0.0 (0.0,0.4)	0.0 (0.0,0.4)	26.6 (23.9,29.5)
				**Pairwise G^2**	0.0 (0.0,0.4)	0.0 (0.0,0.4)	0.0 (0.0,0.4)	0.0 (0.0,0.4)	0.0 (0.0,0.4)	13.8 (11.7,16.1)
				**Pairwise χ^2**	0.0 (0.0,0.4)	0.0 (0.0,0.4)	0.0 (0.0,0.4)	0.0 (0.0,0.4)	0.0 (0.0,0.4)	11.3 (9.4,13.4)

				**Res. Cor.**	0.0 (0.0,0.4)	0.2 (0.0,0.7)	2.5 (1.6,3.6)	0.0 (0.0,0.4)	2.6 (1.7,3.8)	20.1 (17.7,22.8)
**L**	**L**	**L**	**L**	**Pairwise G^2**	0.0 (0.0,0.4)	0.0 (0.0,0.4)	0.0 (0.0,0.4)	0.0 (0.0,0.4)	0.2 (0.0,0.7)	3.6 (2.5,5.0)
				**Pairwise χ^2**	0.0 (0.0,0.4)	0.0 (0.0,0.4)	0.0 (0.0,0.4)	0.0 (0.0,0.4)	0.2 (0.0,0.7)	3.6 (2.5,5.0)
				**Res. Cor.**	0.1 (0.0,0.6)	0.0 (0.0,0.4)	4.9 (3.7,6.5)	0.1 (0.0,0.6)	5.3 (4.0,6.9)	4.7 (3.5,6.2)
**L**	**L**	**H**	**L**	**Pairwise G^2**	0.0 (0.0,0.4)	0.0 (0.0,0.4)	0.2 (0.0,0.7)	0.0 (0.0,0.4)	0.3 (0.1,0.9)	1.4 (0.8,2.3)
				**Pairwise χ^2**	0.0 (0.0,0.4)	0.0 (0.0,0.4)	0.2 (0.0,0.7)	0.0 (0.0,0.4)	0.3 (0.1,0.9)	1.4 (0.8,2.3)
				**Res. Cor.**	6.9 (5.4,8.7)	0.0 (0.0,0.4)	0.2 (0.0,0.7)	0.0 (0.0,0.4)	0.2 (0.0,0.7)	18.6 (16.3,21.2)
**L**	**L**	**H**	**H**	**Pairwise G^2**	2.9 (2.0,4.1)	0.0 (0.0,0.4)	0.0 (0.0,0.4)	0.0 (0.0,0.4)	0.0 (0.0,0.4)	16.0 (13.8,18.4)
				**Pairwise χ^2**	2.7 (1.8,3.9)	0.0 (0.0,0.4)	0.0 (0.0,0.4)	0.0 (0.0,0.4)	0.0 (0.0,0.4)	14.8 (12.7,17.2)
				**Res. Cor.**	0.0 (0.0,0.4)	6.3 (4.9,8.0)	1.2 (0.6,2.1)	0.0 (0.0,0.4)	0.0 (0.0,0.4)	24.6 (22.0,27.4)
**L**	**H**	**L**	**H**	**Pairwise G^2**	0.0 (0.0,0.4)	1.2 (0.6,2.1)	0.2 (0.0,0.7)	0.0 (0.0,0.4)	0.0 (0.0,0.4)	9.4 (7.7,11.4)
				**Pairwise χ^2**	0.0 (0.0,0.4)	1.2 (0.6,2.1)	0.3 (0.1,0.9)	0.0 (0.0,0.4)	0.0 (0.0,0.4)	8.6 (6.9,10.5)
**Sensitivity: H H H H**										
**Specificity**				**Tool**	**T_1&T_2**	**T_1&T_3**	**T_1&T_4**	**T_2&T_3**	**T_2&T_4**	**T_3&T_4**
				**Res. Cor.**	0.0 (0.0,0.4)	0.0 (0.0,0.4)	0.0 (0.0,0.4)	0.0 (0.0,0.4)	0.0 (0.0,0.4)	33.1 (30.2,36.1)
**H**	**H**	**L**	**L**	**Pairwise G^2**	0.0 (0.0,0.4)	0.0 (0.0,0.4)	0.0 (0.0,0.4)	0.0 (0.0,0.4)	0.0 (0.0,0.4)	7.8 (6.2,9.6)
				**Pairwise χ^2**	0.0 (0.0,0.4)	0.0 (0.0,0.4)	0.0 (0.0,0.4)	0.0 (0.0,0.4)	0.0 (0.0,0.4)	7.6 (6.0,9.4)
				**Res. Cor.**	0.0 (0.0,0.4)	0.0 (0.0,0.4)	0.0 (0.0,0.4)	0.0 (0.0,0.4)	0.0 (0.0,0.4)	34.4 (31.5,37.4)
**H**	**H**	**H**	**L**	**Pairwise G^2**	0.0 (0.0,0.4)	0.0 (0.0,0.4)	0.0 (0.0,0.4)	0.0 (0.0,0.4)	0.0 (0.0,0.4)	16.8 (14.5,19.3)
				**Pairwise χ^2**	0.0 (0.0,0.4)	0.0 (0.0,0.4)	0.0 (0.0,0.4)	0.0 (0.0,0.4)	0.0 (0.0,0.4)	15.0 (12.8,17.4)
				**Res. Cor.**	0.0 (0.0,0.4)	0.0 (0.0,0.4)	0.0 (0.0,0.4)	0.0 (0.0,0.4)	0.0 (0.0,0.4)	51.9 (48.8,55.0)
**H**	**H**	**H**	**H**	**Pairwise G^2**	0.0 (0.0,0.4)	0.0 (0.0,0.4)	0.0 (0.0,0.4)	0.0 (0.0,0.4)	0.0 (0.0,0.4)	39.2 (36.2,42.3)
				**Pairwise χ^2**	0.0 (0.0,0.4)	0.0 (0.0,0.4)	0.0 (0.0,0.4)	0.0 (0.0,0.4)	0.0 (0.0,0.4)	36.0 (33.0,39.1)
				**Res. Cor.**	0.7 (0.3,1.4)	1.3 (0.7,2.2)	1.9 (1.2,3.0)	1.6 (0.9,2.6)	1.3 (0.7,2.2)	27.2 (24.5,30.1)
**L**	**L**	**L**	**L**	**Pairwise G^2**	0.2 (0.0,0.7)	0.2 (0.0,0.7)	0.3 (0.1,0.9)	0.2 (0.0,0.7)	0.1 (0.0,0.6)	9.5 (7.8,11.5)
				**Pairwise χ^2**	0.2 (0.0,0.7)	0.2 (0.0,0.7)	0.3 (0.1,0.9)	0.2 (0.0,0.7)	0.2 (0.0,0.7)	9.3 (7.6,11.3)
				**Res. Cor.**	5.7 (4.3,7.3)	0.0 (0.0,0.4)	3.8 (2.7,5.2)	0.0 (0.0,0.4)	4.7 (3.5,6.2)	39.9 (36.9,43.1)
**L**	**L**	**H**	**L**	**Pairwise G^2**	1.4 (0.8,2.3)	0.0 (0.0,0.4)	0.1 (0.0,0.6)	0.0 (0.0,0.4)	0.9 (0.4,1.7)	27.7 (25.0,30.6)
				**Pairwise χ^2**	1.3 (0.7,2.2)	0.0 (0.0,0.4)	0.1 (0.0,0.6)	0.0 (0.0,0.4)	0.9 (0.4,1.7)	25.5 (22.8,28.3)
				**Res. Cor.**	39.0 (36.0,42.1)	0.1 (0.0,0.6)	0.0 (0.0,0.4)	0.2 (0.0,0.7)	0.1 (0.0,0.6)	21.8 (19.3,24.5)
**L**	**L**	**H**	**H**	**Pairwise G^2**	20.1 (17.7,22.7)	0.0 (0.0,0.4)	0.0 (0.0,0.4)	0.0 (0.0,0.4)	0.0 (0.0,0.4)	24.7 (22.1,27.5)
				**Pairwise χ^2**	19.3 (16.9,21.9)	0.0 (0.0,0.4)	0.0 (0.0,0.4)	0.0 (0.0,0.4)	0.0 (0.0,0.4)	23.0 (20.4,25.7)
				**Res. Cor.**	0.2 (0.0,0.7)	9.7 (7.9,11.7)	0.1 (0.0,0.6)	0.0 (0.0,0.4)	0.0 (0.0,0.4)	37.9 (34.9,41.0)
**L**	**H**	**L**	**H**	**Pairwise G^2**	20.1 (17.7,22.7)	0.0 (0.0,0.4)	0.0 (0.0,0.4)	0.0 (0.0,0.4)	0.0 (0.0,0.4)	24.7 (22.1,27.5)
				**Pairwise χ^2**	19.3 (16.9,21.9)	0.0 (0.0,0.4)	0.0 (0.0,0.4)	0.0 (0.0,0.4)	0.0 (0.0,0.4)	23.0 (20.4,25.7)
**Sensitivity: L L L L**										
**Specificity**				**Tool**	**T_1&T_2**	**T_1&T_3**	**T_1&T_4**	**T_2&T_3**	**T_2&T_4**	**T_3&T_4**
				**Res. Cor.**	0.0 (0.0,0.5)	1.6 (0.9,2.7)	0.6 (0.2,1.4)	1.9 (1.0,3.0)	1.1 (0.5,2.1)	69.6 (66.3,72.7)
**H**	**H**	**L**	**L**	**Pairwise G^2**	0.0 (0.0,0.5)	0.0 (0.0,0.5)	0.0 (0.0,0.5)	0.0 (0.0,0.5)	0.0 (0.0,0.5)	7.4 (5.7,9.5)
				**Pairwise χ^2**	0.0 (0.0,0.5)	0.0 (0.0,0.5)	0.0 (0.0,0.5)	0.0 (0.0,0.5)	0.0 (0.0,0.5)	7.4 (5.7,9.5)
**H**	**H**	**H**	**L**	**Res. Cor.**	0.0 (0.0,0.4)	0.0 (0.0,0.4)	1.2 (0.6,2.1)	0.0 (0.0,0.4)	2.0 (1.2,3.1)	59.6 (56.4,62.7)

				**Pairwise G^2**	0.0 (0.0,0.4)	0.0 (0.0,0.4)	0.2 (0.0,0.8)	0.0 (0.0,0.4)	0.0 (0.0,0.4)	19.1 (16.6,21.7)
				**Pairwise χ^2**	0.0 (0.0,0.4)	0.0 (0.0,0.4)	0.2 (0.0,0.8)	0.0 (0.0,0.4)	0.0 (0.0,0.4)	18.9 (16.5,21.6)
**H**	**H**	**H**	**H**	**Res. Cor.**	0.0 (0.0,0.4)	0.0 (0.0,0.4)	0.0 (0.0,0.4)	0.2 (0.0,0.7)	0.2 (0.0,0.7)	91.8 (89.9,93.4)
				**Pairwise G^2**	0.4 (0.1,1.0)	0.0 (0.0,0.4)	0.0 (0.0,0.4)	0.0 (0.0,0.4)	0.1 (0.0,0.6)	78.6 (75.9,81.1)
				**Pairwise χ^2**	0.4 (0.1,1.0)	0.0 (0.0,0.4)	0.0 (0.0,0.4)	0.0 (0.0,0.4)	0.1 (0.0,0.6)	78.6 (75.9,81.1)
**L**	**L**	**L**	**L**	**Res. Cor.**	0.0 (0.0,0.8)	17.0 (13.8,20.7)	15.6 (12.5,19.1)	17.7 (14.4,21.3)	17.7 (14.4,21.3)	75.4 (71.3,79.1)
				**Pairwise G^2**	0.0 (0.0,0.8)	0.8 (0.2,2.1)	0.4 (0.0,1.5)	1.2 (0.5,2.7)	2.1 (1.0,3.7)	12.3 (9.5,15.6)
				**Pairwise χ^2**	0.0 (0.0,0.8)	0.8 (0.2,2.1)	0.4 (0.0,1.5)	1.2 (0.5,2.7)	2.1 (1.0,3.7)	12.3 (9.5,15.6)
**L**	**L**	**H**	**L**	**Res. Cor.**	0.0 (0.0,0.5)	2.7 (1.6,4.1)	23.4 (20.4,26.6)	3.3 (2.2,4.9)	21.7 (18.8,24.8)	78.7 (75.6,81.6)
				**Pairwise G^2**	0.0 (0.0,0.5)	0.5 (0.1,1.4)	1.9 (1.0,3.1)	0.1 (0.0,0.7)	2.7 (1.6,4.1)	37.6 (34.1,41.2)
				**Pairwise χ^2**	0.0 (0.0,0.5)	0.7 (0.2,1.6)	2.0 (1.1,3.3)	0.1 (0.0,0.7)	2.7 (1.6,4.1)	37.3 (33.9,40.9)
**L**	**L**	**H**	**H**	**Res. Cor.**	11.2 (9.0,13.7)	10.7 (8.5,13.1)	11.2 (9.0,13.7)	11.9 (9.6,14.5)	13.0 (10.6,15.7)	80.7 (77.7,83.5)
				**Pairwise G^2**	18.6 (15.8,21.6)	1.6 (0.9,2.8)	1.2 (0.6,2.3)	1.8 (1.0,3.0)	1.5 (0.8,2.7)	88.5 (86.0,90.7)
				**Pairwise χ^2**	18.5 (15.7,21.5)	1.6 (0.9,2.8)	1.5 (0.8,2.7)	1.8 (1.0,3.0)	2.1 (1.2,3.4)	88.5 (86.0,90.7)
**L**	**H**	**L**	**H**	**Res. Cor.**	0.0 (0.0,0.4)	30.1 (27.2,33.2)	27.0 (24.1,29.9)	1.2 (0.6,2.1)	0.0 (0.0,0.4)	66.9 (63.7,69.9)
				**Pairwise G^2**	0.0 (0.0,0.4)	3.1 (2.1,4.5)	5.4 (4.0,7.0)	0.1 (0.0,0.6)	0.0 (0.0,0.4)	24.8 (22.1,27.7)
				**Pairwise χ^2**	0.0 (0.0,0.4)	3.1 (2.1,4.5)	5.5 (4.1,7.2)	0.1 (0.0,0.6)	0.0 (0.0,0.4)	24.5 (21.8,27.4)
**Sensitivity: L L H L**										
**Specificity**				**Tool**	**T_1&T_2**	**T_1&T_3**	**T_1&T_4**	**T_2&T_3**	**T_2&T_4**	**T_3&T_4**
**H**	**H**	**L**	**L**	**Res. Cor.**	0.0 (0.0,0.4)	0.0 (0.0,0.4)	2.0 (1.2,3.0)	0.0 (0.0,0.4)	1.5 (0.9,2.5)	63.4 (60.2,66.4)
				**Pairwise G^2**	0.0 (0.0,0.4)	0.0 (0.0,0.4)	0.1 (0.0,0.6)	0.0 (0.0,0.4)	0.0 (0.0,0.4)	19.8 (17.3,22.5)
				**Pairwise χ^2**	0.0 (0.0,0.4)	0.0 (0.0,0.4)	0.1 (0.0,0.6)	0.0 (0.0,0.4)	0.0 (0.0,0.4)	19.7 (17.2,22.4)
**H**	**H**	**H**	**L**	**Res. Cor.**	0.0 (0.0,0.4)	0.0 (0.0,0.4)	1.5 (0.8,2.5)	0.0 (0.0,0.4)	2.0 (1.2,3.1)	66.5 (63.4,69.4)
				**Pairwise G^2**	0.0 (0.0,0.4)	0.0 (0.0,0.4)	0.1 (0.0,0.6)	0.0 (0.0,0.4)	0.2 (0.0,0.7)	30.4 (27.6,33.4)
				**Pairwise χ^2**	0.0 (0.0,0.4)	0.0 (0.0,0.4)	0.1 (0.0,0.6)	0.0 (0.0,0.4)	0.2 (0.0,0.7)	30.4 (27.6,33.4)
**H**	**H**	**H**	**H**	**Res. Cor.**	21.4 (18.9,24.1)	0.0 (0.0,0.4)	5.3 (4.0,6.9)	0.0 (0.0,0.4)	3.6 (2.5,4.9)	75.3 (72.5,78.0)
				**Pairwise G^2**	35.6 (32.6,38.7)	0.0 (0.0,0.4)	1.4 (0.8,2.4)	0.0 (0.0,0.4)	1.0 (0.5,1.9)	84.4 (82.0,86.7)
				**Pairwise χ^2**	34.9 (31.9,38.0)	0.0 (0.0,0.4)	1.5 (0.9,2.5)	0.0 (0.0,0.4)	1.0 (0.5,1.9)	83.6 (81.2,85.9)
**L**	**L**	**L**	**L**	**Res. Cor.**	0.0 (0.0,0.5)	6.4 (4.7,8.3)	20.8 (18.0,23.8)	7.7 (5.9,9.8)	24.8 (21.8,28.0)	82.3 (79.5,85.0)
				**Pairwise G^2**	0.0 (0.0,0.5)	0.9 (0.4,1.9)	1.3 (0.6,2.4)	1.7 (0.9,2.9)	1.9 (1.1,3.2)	36.6 (33.2,40.1)
				**Pairwise χ^2**	0.0 (0.0,0.5)	0.9 (0.4,1.9)	1.3 (0.6,2.4)	1.7 (0.9,2.9)	1.9 (1.1,3.2)	36.6 (33.2,40.1)
**L**	**L**	**H**	**L**	**Res. Cor.**	0.0 (0.0,0.4)	9.5 (7.7,11.6)	26.1 (23.3,29.0)	8.4 (6.7,10.3)	23.9 (21.2,26.7)	92.9 (91.1,94.4)
				**Pairwise G^2**	0.0 (0.0,0.4)	2.0 (1.2,3.1)	4.1 (2.9,5.5)	1.2 (0.6,2.1)	2.5 (1.6,3.7)	70.5 (67.5,73.3)
				**Pairwise χ^2**	0.0 (0.0,0.4)	2.1 (1.3,3.2)	4.1 (2.9,5.5)	1.5 (0.8,2.4)	2.5 (1.6,3.7)	70.5 (67.5,73.3)
**L**	**L**	**H**	**H**	**Res. Cor.**	91.6 (89.3,93.5)	2.7 (1.7,4.2)	11.2 (9.0,13.7)	2.9 (1.8,4.3)	12.3 (10.0,14.9)	7.2 (5.5,9.3)
				**Pairwise G^2**	93.3 (91.3,95.0)	1.4 (0.7,2.5)	2.9 (1.8,4.3)	1.9 (1.0,3.2)	2.3 (1.4,3.7)	14.4 (12.0,17.2)
				**Pairwise χ^2**	93.2 (91.1,94.9)	1.4 (0.7,2.5)	3.1 (2.0,4.7)	2.0 (1.1,3.3)	2.9 (1.8,4.3)	14.3 (11.9,17.0)
**L**	**H**	**L**	**H**	**Res. Cor.**	1.0 (0.5,1.9)	48.4 (45.2,51.6)	45.2 (42.0,48.4)	0.0 (0.0,0.4)	0.0 (0.0,0.4)	94.8 (93.1,96.1)
				**Pairwise G^2**	3.3 (2.2,4.6)	12.8 (10.7,15.1)	11.0 (9.1,13.2)	0.0 (0.0,0.4)	0.0 (0.0,0.4)	94.4 (92.8,95.8)
				**Pairwise χ^2**	3.1 (2.1,4.5)	13.0 (10.9,15.3)	11.2 (9.3,13.4)	0.0 (0.0,0.4)	0.0 (0.0,0.4)	94.0 (92.3,95.4)
**Sensitivity: L L H H**										

Specificity				Tool	T_1&T_2	T_1&T_3	T_1&T_4	T_2&T_3	T_2&T_4	T_3&T_4
H	H	L	L	**Res. Cor.**	0.0 (0.0,0.4)	0.0 (0.0,0.4)	0.0 (0.0,0.4)	0.0 (0.0,0.4)	0.0 (0.0,0.4)	99.0 (98.2,99.5)
				**Pairwise G^2**	0.0 (0.0,0.4)	0.0 (0.0,0.4)	0.0 (0.0,0.4)	0.0 (0.0,0.4)	0.0 (0.0,0.4)	86.5 (84.2,88.6)
				**Pairwise χ^2**	0.0 (0.0,0.4)	0.0 (0.0,0.4)	0.0 (0.0,0.4)	0.0 (0.0,0.4)	0.0 (0.0,0.4)	86.5 (84.2,88.6)
H	H	H	L	**Res. Cor.**	3.4 (2.4,4.8)	0.0 (0.0,0.4)	0.8 (0.3,1.6)	0.0 (0.0,0.4)	1.3 (0.7,2.2)	97.6 (96.4,98.4)
				**Pairwise G^2**	7.6 (6.0,9.4)	0.0 (0.0,0.4)	0.1 (0.0,0.6)	0.0 (0.0,0.4)	0.0 (0.0,0.4)	98.7 (97.8,99.3)
				**Pairwise χ^2**	7.2 (5.6,8.9)	0.0 (0.0,0.4)	0.1 (0.0,0.6)	0.0 (0.0,0.4)	0.0 (0.0,0.4)	98.7 (97.8,99.3)
H	H	H	H	**Res. Cor.**	98.4 (97.4,99.1)	0.0 (0.0,0.4)	0.0 (0.0,0.4)	0.0 (0.0,0.4)	0.0 (0.0,0.4)	3.9 (2.8,5.4)
				**Pairwise G^2**	99.5 (98.8,99.8)	0.0 (0.0,0.4)	0.0 (0.0,0.4)	0.0 (0.0,0.4)	0.0 (0.0,0.4)	7.0 (5.5,8.7)
				**Pairwise χ^2**	99.5 (98.8,99.8)	0.0 (0.0,0.4)	0.0 (0.0,0.4)	0.0 (0.0,0.4)	0.0 (0.0,0.4)	6.7 (5.2,8.4)
L	L	L	L	**Res. Cor.**	5.4 (3.9,7.2)	25.9 (22.9,29.1)	22.0 (19.2,25.0)	22.0 (19.2,25.0)	26.8 (23.8,29.9)	93.4 (91.5,95.0)
				**Pairwise G^2**	7.3 (5.6,9.3)	7.3 (5.6,9.3)	5.1 (3.7,6.9)	5.1 (3.7,6.9)	6.8 (5.2,8.8)	96.5 (94.9,97.6)
				**Pairwise χ^2**	7.2 (5.5,9.2)	7.6 (5.9,9.6)	5.3 (3.8,7.0)	5.4 (3.9,7.2)	7.6 (5.9,9.6)	96.5 (94.9,97.6)
L	L	H	L	**Res. Cor.**	79.3 (75.9,82.4)	8.2 (6.2,10.6)	18.8 (15.8,22.1)	7.4 (5.5,9.7)	20.7 (17.6,24.1)	25.9 (22.5,29.5)
				**Pairwise G^2**	78.9 (75.5,82.1)	2.6 (1.5,4.1)	5.6 (4.0,7.7)	3.5 (2.2,5.3)	7.4 (5.5,9.7)	34.6 (30.8,38.5)
				**Pairwise χ^2**	78.9 (75.5,82.1)	2.6 (1.5,4.1)	5.9 (4.2,8.1)	3.7 (2.4,5.5)	7.6 (5.6,9.9)	34.6 (30.8,38.5)
L	L	H	H	**Res. Cor.**	100.0 (99.6,100.0)	0.0 (0.0,0.4)	0.2 (0.0,0.8)	0.0 (0.0,0.4)	0.0 (0.0,0.4)	0.0 (0.0,0.4)
				**Pairwise G^2**	99.9 (99.4,100.0)	0.0 (0.0,0.4)	0.0 (0.0,0.4)	0.0 (0.0,0.4)	0.0 (0.0,0.4)	0.0 (0.0,0.4)
				**Pairwise χ^2**	99.9 (99.4,100.0)	0.0 (0.0,0.4)	0.0 (0.0,0.4)	0.0 (0.0,0.4)	0.0 (0.0,0.4)	0.0 (0.0,0.4)
L	H	L	H	**Res. Cor.**	26.0 (23.0,29.1)	46.3 (42.9,49.7)	42.0 (38.6,45.4)	0.2 (0.0,0.9)	0.0 (0.0,0.4)	70.7 (67.5,73.8)
				**Pairwise G^2**	37.7 (34.4,41.1)	14.0 (11.7,16.5)	20.4 (17.7,23.3)	0.0 (0.0,0.4)	0.0 (0.0,0.4)	80.1 (77.2,82.7)
				**Pairwise χ^2**	37.1 (33.9,40.5)	14.2 (11.9,16.8)	21.0 (18.3,23.9)	0.0 (0.0,0.4)	0.0 (0.0,0.4)	79.8 (77.0,82.5)

Table vi.4 Mean absolute biases (x10^{-2}) of posterior medians of each parameter obtained from the conditional independence model

Sensitivity: H H L L												
**Specificity**				π	Se_1	Se_2	Se_3	Se_4	Sp_1	Sp_2	Sp_3	Sp_4
H	H	L	L	-1.8 (-2.0,-1.6)	4.2 (4.1,4.4)	4.1 (4.0,4.3)	-0.5 (-0.7,-0.3)	-0.4 (-0.6,-0.2)	1.0 (0.8,1.1)	1.1 (0.9,1.3)	-1.1 (-1.3,-0.9)	-1.1 (-1.3,-0.9)
H	H	H	L	-2.9 (-3.1,-2.8)	5.1 (5.0,5.2)	5.2 (5.0,5.3)	-0.4 (0.6,-0.2)	-0.1 (-0.3,-0.1)	0.0 (-0.1,0.2)	0.1 (-0.1,0.2)	-3.3 (-3.4,-3.2)	-1.3 (-1.5,-1.1)
H	H	H	H	-2.9 (-3.0,-2.7)	5.1 (4.9,5.2)	5.1 (45.0,5.2)	-0.2 (-0.4,0.0)	-0.1 (-0.3,0.1)	-0.2 (-0.3,0.0)	-0.1 (-0.2,0.0)	-3.1 (-3.2,-2.9)	-3.3 (-3.4,-3.1)
L	L	L	L	2.8 (2.5,3.2)	2.3 (2.1,2.6)	2.6 (2.3,2.8)	-1.3 (-1.5,-1.0)	-1.2 (-1.4,-0.9)	5.6 (5.2,6.0)	5.7 (5.3,6.1)	-0.4 (-0.7,-0.2)	-0.5 (-0.7,-0.2)
L	L	H	L	-3.6 (-3.8,-3.4)	5.8 (5.7,5.9)	5.8 (5.7,5.9)	-1.2 (-1.5,-0.9)	-0.3 (-0.5,-0.1)	1.6 (1.3,1.8)	1.7 (1.4,2.0)	-4.6 (-4.8,-4.5)	-1.6 (-1.8,-1.4)
L	L	H	H	-4.0 (-4.3,-3.8)	5.7 (5.5,5.8)	5.7 (5.5,5.8)	-0.3 (-0.6,-0.1)	-0.1 (-0.3,0.2)	0.9 (0.7,1.2)	0.9 (0.7,1.2)	-4.1 (-4.3,-4.0)	-3.9 (-4.1,-3.7)
L	H	L	H	-3.3 (-3.5,-3.1)	5.1 (5.0,5.2)	5.9 (5.7,6.0)	0.1 (-0.1,0.4)	-0.5 (-0.8,-0.3)	1.3 (1.1,1.5)	0.1 (-0.1,0.3)	-1.5 (-1.7,-1.3)	-3.7 (-3.8,3.5)
**Sensitivity: H H H L**												
**Specificity**				π	Se_1	Se_2	Se_3	Se_4	Sp_1	Sp_2	Sp_3	Sp_4
H	H	L	L	-3.1 (-3.2,-2.9)	5.2 (5.0,5.3)	5.2 (5.1,5.4)	-0.3 (-0.5,-0.2)	0.0 (-0.2,0.3)	-0.1 (-0.3,0.0)	0.0 (-0.1,0.2)	-3.1 (-3.3,-2.9)	-1.2 (-1.4,-1.0)
H	H	H	L	-3.4 (-3.6,-3.3)	5.3 (5.2,5.4)	5.4 (5.3,5.5)	-0.3 (-0.5,-0.2)	0.2 (0.0,0.4)	-0.3 (-0.4,-0.1)	-0.3 (-0.4,-0.1)	-5.2 (-5.4,-5.1)	-1.2 (-1.4,-1.0)
H	H	H	H	-3.0 (-3.2,-2.9)	5.0 (4.9,5.1)	5.0 (4.8,5.1)	-0.2 (-0.3,0.0)	0.5 (0.3,0.7)	-0.1 (-0.2,0.0)	-0.1 (-0.2,0.1)	-4.8 (-5.0,-4.7)	-2.7 (-2.8,-2.6)

**L**	**L**	**L**	**L**	-2.3 (-2.6,-2.1)	5.2 (5.1,5.4)	5.1 (4.9,5.2)	-1.4 (-1.6,-1.2)	-0.5 (-0.7,-0.3)	2.3 (2.0,2.6)	2.3 (2.1,2.6)	-3.7 (-4.0,-3.5)	-1.5 (-1.7,-1.3)
**L**	**L**	**H**	**L**	-5.0 (-5.2,-4.8)	6.8 (6.7,6.9)	6.7 (6.6,6.8)	-1.2 (-1.4,-1.0)	0.2 (0.0,0.5)	1.0 (0.8,1.2)	1.1 (0.9,1.4)	-8.1 (-8.3,-8.0)	-1.6 (-1.8,-1.5)
**L**	**L**	**H**	**H**	-4.1 (-4.3,-3.9)	5.3 (5.2,5.4)	5.3 (5.2,5.5)	0.0 (-0.2,0.1)	0.6 (0.4,0.8)	0.6 (0.4,0.9)	0.5 (0.3,0.7)	-6.3 (-6.5,-6.1)	-3.4 (-3.5,-3.2)
**L**	**H**	**L**	**H**	-3.3 (-3.5,-3.1)	5.0 (4.9,5.1)	5.8 (5.6,5.9)	-0.2 (-0.4,-0.1)	-0.3 (-0.5,0.0)	1.4 (1.2,1.6)	0.0 (-0.2,0.2)	-3.4 (-3.6,-3.1)	-3.4 (-3.5,-3.2)
**Sensitivity: H H H H**												
**Specificity**				π	Se_1	Se_2	Se_3	Se_4	Sp_1	Sp_2	Sp_3	Sp_4
**H**	**H**	**L**	**L**	-3.0 (-3.2,-2.9)	5.2 (5.1,5.3)	5.1 (4.9,5.2)	-0.2 (-0.4,-0.1)	-0.3 (-0.4,-0.1)	-0.1 (-0.2,0.1)	-0.1 (-0.3,0.0)	-3.4 (-3.6,-3.2)	-3.2 (-3.4,-3.0)
**H**	**H**	**H**	**L**	-3.0 (-3.2,-2.9)	5.0 (4.9,5.1)	5.0 (4.9,5.1)	-0.3 (-0.4,-0.2)	-0.1 (-0.3,0.0)	0.0 (-0.2,0.1)	-0.1 (-0.2,0.0)	-4.7 (-4.9,-4.6)	-2.9 (-3.1,-2.7)
**H**	**H**	**H**	**H**	-2.5 (-2.6,-2.4)	4.3 (4.2,4.4)	4.3 (4.2,4.4)	-0.1 (-0.2,0.1)	-0.1 (-0.2,0.0)	0.1 (0.0,0.3)	0.2 (0.0,0.3)	-4.0 (-4.1,-3.9)	-4.1 (-4.2,-3.9)
**L**	**L**	**L**	**L**	-3.8 (-4.0,-3.5)	5.6 (5.5,5.7)	5.6 (5.5,5.7)	-0.4 (-0.6,-0.2)	-0.5 (-0.6,-0.3)	1.2 (1.0,1.5)	1.4 (1.2,1.6)	-3.9 (-4.1,-3.7)	-3.9 (-4.1,-3.7)
**L**	**L**	**H**	**L**	-4.5 (-4.7,-4.3)	5.8 (5.7,6.0)	5.8 (5.7,5.9)	-0.2 (-0.4,0.0)	0.5 (0.3,0.6)	0.7 (0.4,0.9)	0.7 (0.5,0.9)	-6.7 (-6.9,-6.5)	-3.6 (-3.8,-3.4)
**L**	**L**	**H**	**H**	-3.0 (-3.1,-2.8)	3.6 (3.4,3.7)	3.6 (3.4,3.7)	0.3 (0.2,0.5)	0.5 (0.3,0.6)	0.3 (0.1,0.5)	0.4 (0.2,0.6)	-3.9 (-4.1,-3.7)	-3.9 (-4.0,-3.7)
**L**	**H**	**L**	**H**	-3.6 (-3.7,-3.4)	4.9 (4.7,5.0)	5.8 (5.7,6.0)	0.0 (-0.1,0.2)	-0.3 (-0.5,-0.2)	0.9 (0.7,1.1)	-0.1 (-0.3,0.0)	-3.1 (-3.3,-2.9)	-5.5 (-5.6,-5.3)
**Sensitivity: L L L L**												
**Specificity**				π	Se_1	Se_2	Se_3	Se_4	Sp_1	Sp_2	Sp_3	Sp_4
**H**	**H**	**L**	**L**	-16.1 (-16.3,-15.8)	24.0 (23.6,24.4)	24.4 (24.0,24.8)	-0.2 (-0.5,0.1)	0.1 (-0.2,0.4)	-0.3 (-0.5,0.0)	-0.2 (-0.4,0.1)	-5.1 (-5.3,-4.9)	-5.1 (-5.3,-4.9)
**H**	**H**	**H**	**L**	-16.0 (-16.2,-15.7)	23.6 (23.3,23.9)	23.6 (23.3,24.0)	-0.4 (-0.7,0.1)	0.2 (-0.1,0.4)	-0.1 (-0.3,0.0)	0.0 (-0.2,0.2)	-12.5 (-12.7,-12.4)	-4.7 (-4.9,-4.5)
**H**	**H**	**H**	**H**	-14.6 (-14.8,-14.3)	21.3 (21.0,21.7)	21.4 (21.1,21.7)	0.5 (0.2,0.7)	0.6 (0.4,0.9)	0.3 (0.1,0.4)	0.2 (0.1,0.4)	-11.1 (-11.2,-10.9)	-11.3 (-11.5,-11.1)
**L**	**L**	**L**	**L**	-12.9 (-13.4,-12.3)	24.6 (24.1,25.2)	24.1 (23.6,24.6)	-2.7 (-3.1,-2.2)	-2.5 (-2.9,-2.1)	9.9 (9.5,10.3)	9.5 (9.1,9.9)	-5.7 (-5.9,-5.4)	-6.0 (-6.2,-5.7)
**L**	**L**	**H**	**L**	-20.9 (-21.2,-20.6)	30.8 (30.5,31.2)	30.6 (30.3,30.9)	-5.5 (-6.0,-5.1)	-0.4 (-0.8,-0.1)	6.6 (6.3,6.9)	6.4 (6.1,6.7)	-17.4 (-17.6,-17.2)	-6.3 (-6.5,-6.1)
**L**	**L**	**H**	**H**	-20.7 (-21.1,-20.3)	26.0 (25.4,26.6)	25.7 (25.1,26.4)	0.4 (-0.1,0.9)	0.3 (-0.2,0.8)	4.3 (4.0,4.5)	4.1 (3.8,4.4)	-14.3 (-14.6,-14.0)	-14.3 (-14.6,-14.0)
**L**	**H**	**L**	**H**	-19.3 (-19.6,-19.1)	23.6 (23.3,23.9)	32.2 (31.9,32.4)	0.4 (0.2,0.7)	-0.3 (-0.6,0.1)	4.7 (4.5,4.8)	0.1 (-0.2,0.3)	-5.5 (-5.7,-5.3)	-14.5 (-14.6,-14.3)
**Sensitivity: L L H L**												
**Specificity**				π	Se_1	Se_2	Se_3	Se_4	Sp_1	Sp_2	Sp_3	Sp_4
**H**	**H**	**L**	**L**	-16.1 (-16.4,-15.9)	23.7 (23.4,24.0)	23.7 (23.3,24.0)	-0.4 (-0.6,-0.3)	0.3 (0.0,0.5)	-0.2 (-0.3,0.0)	-0.1 (-0.3,0.1)	-12.4 (-12.6,-12.2)	-4.8 (-5.0,-4.7)
**H**	**H**	**H**	**L**	-15.9 (-16.1,-15.7)	23.4 (23.1,23.6)	23.3 (23.0,23.6)	-0.4 (-0.6,-0.2)	1.1 (0.8,1.3)	-0.1 (-0.2,0.1)	-0.3 (-0.4,-0.1)	-19.7 (-19.9,-19.5)	-4.4 (-4.6,-4.2)
**H**	**H**	**H**	**H**	-11.6 (-11.9,-11.3)	16.5 (16.1,17.0)	16.6 (16.2,17.0)	0.1 (-0.1,0.3)	2.9 (2.6,3.1)	0.2 (0.1,0.4)	0.4 (0.2,0.5)	-14.8 (-15.1,-14.5)	-7.6 (-7.8,-7.3)
**L**	**L**	**L**	**L**	-20.5 (-20.9,-20.2)	30.3 (30.0,30.6)	30.2 (29.9,30.6)	-5.7 (-6.1,-5.4)	-0.3 (-0.6,0.1)	6.7 (6.4,7.0)	6.4 (6.2,6.7)	-17.3 (-17.5,-17.0)	-6.1 (-6.3,-5.9)
**L**	**L**	**H**	**L**	-24.0 (-24.2,-23.7)	33.1 (32.9,33.4)	33.3 (33.1,33.5)	-7.1 (-7.5,-6.7)	1.4 (1.0,1.7)	5.0 (4.8,5.2)	5.1 (4.9,5.3)	-28.5 (-28.7,-28.4)	-6.1 (-6.3,-6.0)

**L**	**L**	**H**	**H**	-8.0 (-8.5,-7.4)	7.5 (6.8,8.2)	7.3 (6.6,8.0)	0.1 (-0.2,0.4)	5.9 (5.5,6.3)	1.2 (0.9,1.5)	1.1 (0.8,1.3)	-10.2 (-10.8,-9.6)	-2.3 (-2.7,-1.9)
**L**	**H**	**L**	**H**	-17.7 (-18.0,-17.4)	21.5 (21.1,21.8)	28.4 (28.0,28.8)	-0.1 (-0.3,0.2)	0.7 (0.4,1.0)	4.9 (4.7,5.1)	0.1 (-0.1,0.3)	-13.1 (-13.3,-12.9)	-12.9 (-13.1,-12.7)
**Sensitivity: L L H H**												
**Specificity**				π	Se_1	Se_2	Se_3	Se_4	Sp_1	Sp_2	Sp_3	Sp_4
**H**	**H**	**L**	**L**	-15.0 (-15.2,-14.8)	22.1 (21.8,22.4)	22.3 (22.0,22.6)	-0.2 (-0.4,0.0)	0.0 (-0.2,0.2)	0.3 (0.1,0.4)	0.3 (0.1,0.4)	-11.8 (-12.0,-11.6)	-11.9 (-12.1,-11.7)
**H**	**H**	**H**	**L**	-13.1 (-13.3,-12.9)	19.1 (18.7,19.4)	19.0 (18.7,19.3)	-0.2 (-0.4,0.0)	0.9 (0.8,1.1)	0.4 (0.3,0.5)	0.5 (0.4,0.7)	-16.8 (-17.1,-16.6)	-9.7 (-10.0,-9.5)
**H**	**H**	**H**	**H**	-2.6 (-2.8,-2.4)	3.4 (3.1,3.7)	3.4 (3.1,3.8)	0.7 (0.5,0.8)	0.8 (0.6,0.9)	0.4 (0.3,0.5)	0.3 (0.2,0.4)	-3.1 (-3.3,-2.9)	-3.2 (-3.4,-2.9)
**L**	**L**	**L**	**L**	-21.3 (-21.7,-21.0)	29.1 (28.6,29.6)	29.1 (28.6,29.6)	-1.1 (-1.4,-0.8)	-1.1 (-1.4,-0.8)	5.0 (4.7,5.2)	5.2 (5.0,5.4)	-15.3 (-15.6,-15.0)	-15.3 (-15.6,-15.0)
**L**	**L**	**H**	**L**	-7.5 (-8.4,-6.6)	10.4 (9.4,11.5)	10.6 (9.5,11.6)	-1.6 (-2.0,-1.2)	2.4 (2.2,2.6)	2.1 (1.8,2.4)	2.4 (2.1,2.7)	-10.5 (-11.4,-9.7)	-3.2 (-3.9,-2.5)
**L**	**L**	**H**	**H**	-0.9 (-1.1,-0.7)	0.8 (0.6,1.0)	0.7 (0.4,0.9)	0.5 (0.3,0.7)	0.5 (0.3,0.7)	0.2 (0.0,0.4)	0.0 (-0.2,0.2)	-1.0 (-1.2,-0.8)	-0.9 (-1.2,-0.7)
**L**	**H**	**L**	**H**	-13.2 (-13.7,-12.7)	16.0 (15.4,16.6)	20.5 (19.7,21.3)	1.2 (1.0,1.3)	0.0 (-0.2,0.3)	4.0 (3.8,4.2)	0.1 (-0.1,0.2)	-8.9 (-9.4,-8.5)	-16.0 (-16.6,-15.5)

Table vi.5 Coverages of the 95% CrIs for each model parameter

**Sensitivity: H H L L**												
**Specificity**				π	Se_1	Se_2	Se_3	Se_4	Sp_1	Sp_2	Sp_3	Sp_4
**H**	**H**	**L**	**L**	96.3 (95.1,97.5)	89.5 (87.6,91.5)	89.4 (87.4,91.4)	95.6 (94.3,96.9)	95.9 (94.6,97.2)	98.3 (97.5,99.1)	97.7 (96.7,98.6)	93.1 (91.5,94.7)	92.7 (91.0,94.3)
**H**	**H**	**H**	**L**	83.2 (80.9,85.5)	53.5 (50.4,56.6)	52.2 (49.1,55.3)	94.7 (93.3,96.1)	95.3 (94.0,96.6)	95.7 (94.4,97.0)	96.5 (95.4,97.6)	66.9 (64.0,69.8)	93.6 (92.1,95.1)
**H**	**H**	**H**	**H**	79.5 (77.0,82.0)	42.0 (38.9,45.1)	43.1 (40.0,46.2)	95.4 (94.1,96.7)	95.8 (94.6,97.0)	95.6 (94.3,96.9)	94.7 (93.3,96.1)	69.6 (66.7,72.5)	66.2 (63.3,69.1)
**L**	**L**	**L**	**L**	98.7 (97.8,99.5)	98.2 (97.3,99.2)	98.1 (97.1,99.1)	93.9 (92.2,95.6)	95.1 (93.6,96.7)	89.3 (87.1,91.6)	90.4 (88.3,92.5)	96.6 (95.3,97.9)	96.2 (94.8,97.6)
**L**	**L**	**H**	**L**	91.8 (90.1,93.5)	58.7 (55.7,61.8)	56.7 (53.6,59.8)	93.4 (91.8,94.9)	93.4 (91.8,94.9)	94.8 (93.4,96.2)	94.6 (93.2,96.0)	60.0 (56.9,63.0)	91.5 (89.7,93.2)
**L**	**L**	**H**	**H**	82.9 (80.5,85.2)	45.4 (42.3,48.5)	45.7 (42.6,48.8)	95.1 (93.7,96.4)	95.7 (94.4,96.9)	95.9 (94.6,97.1)	94.5 (93.0,95.9)	62.8 (59.8,65.8)	66.7 (63.8,69.7)
**L**	**H**	**L**	**H**	86.5 (94.3,88.6)	48.5 (45.4,51.6)	59.2 (56.1,62.2)	94.9 (93.5,96.3)	94.1 (92.6,95.5)	94.6 (93.2,96.0)	97.2 (96.2,98.2)	93.5 (91.9,95.0)	67.3 (64.4,70.2)
**Sensitivity: H H H L**												
**Specificity**				π	Se_1	Se_2	Se_3	Se_4	Sp_1	Sp_2	Sp_3	Sp_4
**H**	**H**	**L**	**L**	81.0 (78.5,83.4)	49.6 (46.5,52.7)	50.8 (47.7,53.9)	96.2 (95.0,97.4)	94.7 (93.3,96.1)	97.3 (96.4,98.4)	97.5 (96.5,98.5)	84.5 (82.2,86.7)	92.6 (91.0,94.2)
**H**	**H**	**H**	**L**	73.7 (71.0,76.4)	30.1 (27.3,32.9)	26.9 (24.2,29.6)	94.1 (92.6,95.6)	95.1 (93.8,96.4)	95.3 (94.0,96.6)	94.3 (92.9,95.7)	37.0 (34.0,40.0)	92.9 (91.3,94.5)
**H**	**H**	**H**	**H**	76.0 (73.4,78.6)	33.5 (30.6,36.4)	34.3 (31.4,37.2)	94.7 (93.3,96.1)	95.1 (93.8,96.4)	94.4 (93.0,95.8)	95.0 (93.6,96.4)	42.6 (39.5,45.7)	72.0 (69.2,74.8)
**L**	**L**	**L**	**L**	97.1 (96.1,98.2)	69.8 (66.9,72.7)	72.3 (69.4,75.1)	94.0 (92.6,95.5)	96.1 (94.9,97.3)	93.8 (92.3,95.3)	93.9 (92.4,95.4)	86.7 (84.6,88.9)	93.2 (91.6,94.8)
**L**	**L**	**H**	**L**	73.1 (70.3,75.8)	18.5 (16.1,20.9)	20.6 (18.1,23.1)	95.6 (94.3,96.9)	95.5 (94.2,96.8)	95.3 (94.0,96.6)	95.0 (93.6,96.3)	19.7 (17.2,22.1)	93.3 (91.7,94.8)
**L**	**L**	**H**	**H**	73.7 (70.9,76.4)	38.7 (35.7,41.8)	40.9 (37.9,44.0)	95.9 (94.7,97.1)	95.4 (94.1,96.7)	95.7 (94.4,97.0)	95.9 (94.7,97.1)	41.3 (38.3,44.4)	69.8 (66.9,72.6)
**L**	**H**	**L**	**H**	81.6 (79.2,84.0)	40.2 (37.2,43.2)	51.2 (48.1,54.3)	96.4 (95.2,97.6)	95.1 (93.8,96.4)	92.8 (91.2,94.4)	96.6 (95.5,97.7)	82.9 (80.6,85.2)	68.5 (65.6,71.4)
**Sensitivity: H H H H**												

Specificity				π	Se_1	Se_2	Se_3	Se_4	Sp_1	Sp_2	Sp_3	Sp_4
**H**	**H**	**L**	**L**	80.5 (78.0,83.0)	39.7 (36.7,42.7)	41.7 (38.6,44.8)	95.0 (93.6,96.4)	94.2 (92.8,95.6)	94.7 (93.3,96.1)	95.3 (94.0,96.6)	80.8 (78.4,83.2)	83.1 (80.8,85.4)
**H**	**H**	**H**	**L**	76.0 (73.4,78.6)	30.3 (27.5,33.1)	32.8 (29.9,35.7)	94.5 (93.1,95.9)	96.2 (95.0,97.4)	95.2 (93.9,96.5)	95.6 (94.3,96.9)	41.8 (38.7,44.9)	83.0 (80.7,85.3)
**H**	**H**	**H**	**H**	82.0 (79.6,94.4)	43.7 (40.6,46.8)	42.8 (39.7,45.9)	94.3 (92.9,95.7)	96.2 (95.0,97.4)	95.4 (94.1,96.7)	95.0 (93.6,96.4)	53.9 (50.8,57.0)	52.8 (49.7,55.9)
**L**	**L**	**L**	**L**	85.0 (82.8,87.2)	49.7 (46.6,52.9)	48.0 (44.9,51.1)	95.8 (94.5,97.0)	95.8 (94.5,97.0)	95.2 (93.8,96.5)	95.6 (94.3,96.9)	83.6 (81.3,85.9)	81.4 (79.0,83.8)
**L**	**L**	**H**	**L**	73.2 (70.4,75.9)	29.1 (26.3,31.9)	28.0 (25.2,30.8)	96.5 (95.4,97.6)	95.2 (93.9,96.5)	95.1 (93.8,96.4)	95.0 (93.6,96.3)	33.6 (30.7,36.6)	83.2 (80.9,85.5)
**L**	**L**	**H**	**H**	80.1 (77.6,82.6)	65.9 (63.0,68.8)	64.8 (61.8,67.8)	95.1 (93.8,96.4)	95.3 (94.0,96.6)	95.7 (94.4,97.0)	95.6 (94.3,96.9)	68.4 (65.5,71.3)	69.8 (67.0,72.6)
**L**	**H**	**L**	**H**	72.2 (69.4,75.0)	34.2 (31.3,37.1)	31.0 (28.1,33.9)	95.5 (94.2,96.8)	95.9 (94.7,97.1)	94.7 (63.3,96.1)	95.7 (94.4,97.0)	83.5 (81.2,85.8)	36.5 (33.5,39.5)
**Sensitivity: L L L L**												
**Specificity**				π	Se_1	Se_2	Se_3	Se_4	Sp_1	Sp_2	Sp_3	Sp_4
**H**	**H**	**L**	**L**	9.5 (7.5,11.6)	0.7 (0.2,1.3)	0.9 (0.2,1.5)	95.7 (94.3,97.1)	95.7 (94.3,97.1)	99.3 (98.7,99.8)	99.3 (98.7,99.8)	60.6 (57.3,64.0)	58.8 (55.4,62.2)
**H**	**H**	**H**	**L**	3.4 (2.2,4.5)	0.5 (0.1,1.0)	0.4 (0.0,0.8)	95.2 (93.8,96.5)	94.6 (93.2,96.1)	96.4 (95.2,97.6)	95.9 (94.6,97.2)	0.1 (0.0,0.3)	62.9 (59.9,66.0)
**H**	**H**	**H**	**H**	5.8 (4.3,7.2)	2.1 (1.2,3.0)	1.9 (1.1,2.8)	95.4 (94.1,96.7)	95.2 (93.9,96.6)	95.2 (93.9,96.6)	96.2 (95.1,97.4)	2.7 (1.7,3.8)	3.2 (2.1,4.4)
**L**	**L**	**L**	**L**	95.3 (93.4,97.2)	9.7 (7.0,12.3)	11.7 (8.8,14.6)	92.6 (90.3,94.9)	94.0 (91.9,96.1)	51.5 (47.1,56.0)	52.6 (48.1,57.0)	64.1 (59.8,68.3)	61.2 (56.9,65.5)
**L**	**L**	**H**	**L**	33.2 (29.8,36.6)	0.9 (0.2,1.6)	1.1 (0.3,1.8)	86.9 (84.5,89.3)	96.7 (95.4,97.9)	63.6 (60.1,67.0)	64.7 (61.2,68.1)	0.3 (0.0,0.6)	44.8 (41.3,48.4)
**L**	**L**	**H**	**H**	26.6 (23.8,29.3)	19.6 (17.1,22.1)	20.7 (18.2,23.3)	91.7 (90.0,93.5)	93.0 (91.3,94.6)	77.1 (74.5,79.8)	77.6 (75.0,80.2)	22.5 (19.9,25.1)	21.5 (18.9,24.0)
**L**	**H**	**L**	**H**	2.5 (1.5,3.5)	0.0 (0.0,0.0)	0.3 (0.0,0.7)	96.1 (94.9,97.4)	95.9 (94.6,97.2)	66.9 (63.9,69.9)	97.0 (95.9,98.1)	52.6 (49.4,55.9)	0.2 (0.0,0.5)
**Sensitivity: L L H L**												
**Specificity**				π	Se_1	Se_2	Se_3	Se_4	Sp_1	Sp_2	Sp_3	Sp_4
**H**	**H**	**L**	**L**	2.4 (1.4,3.3)	0.3 (0.0,0.7)	0.4 (0.0,0.8)	94.3 (92.9,95.8)	95.5 (94.1,96.8)	96.4 (95.2,97.6)	97.1 (96.1,98.2)	3.6 (2.4,4.8)	59.5 (56.5,62.6)
**H**	**H**	**H**	**L**	0.3 (0.0,0.6)	0.2 (0.0,0.5)	0.0 (0.0,0.0)	94.6 (93.2,96.0)	93.7 (92.2,95.2)	94.5 (93.1,95.9)	94.7 (93.3,96.1)	0.0 (0.0,0.0)	66.1 (63.1,69.0)
**H**	**H**	**H**	**H**	17.3 (14.9,19.7)	16.0 (13.7,18.3)	15.3 (13.0,17.5)	95.7 (94.4,97.0)	89.3 (87.3,91.2)	95.8 (94.5,97.1)	95.5 (94.2,96.8)	7.4 (5.7,9.0)	33.3 (30.3,36.2)
**L**	**L**	**L**	**L**	31.3 (28.0,34.6)	1.2 (0.4,1.9)	1.0 (0.3,1.8)	82.9 (80.2,85.5)	95.1 (93.5,96.6)	60.6 (57.2,64.1)	63.8 (60.4,67.2)	0.9 (0.2,1.6)	50.5 (47.0,54.1)
**L**	**L**	**H**	**L**	2.3 (1.4,3.3)	0.4 (0.0,0.8)	0.3 (0.0,0.7)	84.4 (82.1,86.7)	96.1 (94.9,97.3)	70.1 (67.1,73.0)	69.8 (66.9,72.8)	0.0 (0.0,0.0)	42.0 (38.9,45.1)
**L**	**L**	**H**	**H**	75.9 (72.8,79.0)	72.9 (69.7,76.1)	74.1 (70.9,77.3)	98.2 (97.3,99.2)	84.2 (81.6,86.8)	92.5 (90.6,94.4)	92.9 (91.1,94.8)	66.6 (63.2,70.0)	89.1 (86.8,91.4)
**L**	**H**	**L**	**H**	8.8 (7.0,10.6)	4.4 (3.1,5.7)	6.5 (4.9,8.1)	94.8 (93.3,96.2)	94.8 (93.3,96.2)	65.6 (62.6,68.6)	97.5 (96.5,98.5)	10.8 (8.8,12.8)	8.9 (7.1,10.7)
**Sensitivity: L L H H**												
**Specificity**				π	Se_1	Se_2	Se_3	Se_4	Sp_1	Sp_2	Sp_3	Sp_4
**H**	**H**	**L**	**L**	1.6 (0.8,2.4)	0.3 (0.0,0.6)	0.4 (0.0,0.8)	94.5 (93.0,95.9)	94.3 (92.8,95.7)	94.5 (93.0,95.9)	95.8 (94.5,97.0)	4.2 (3.0,5.5)	4.4 (3.1,5.7)
**H**	**H**	**H**	**L**	7.2 (5.6,8.8)	6.4 (4.6,7.5)	6.6 (5.0,8.1)	94.7 (93.3,96.1)	94.7 (93.3,96.1)	94.3 (92.8,95.7)	95.3 (93.9,96.6)	2.6 (1.6,3.6)	20.7 (18.1,23.2)
**H**	**H**	**H**	**H**	84.1 (91.8,96.4)	84.5 (82.3,86.8)	85.1 (82.9,87.4)	94.6 (93.2,96.0)	94.3 (92.9,95.8)	95.1 (93.8,96.5)	94.5 (93.1,96.0)	79.6 (77.1,82.1)	79.6 (77.1,82.1)
**L**	**L**	**L**	**L**	14.4 (12.0,16.8)	7.7 (5.9,9.5)	8.2 (6.3,10.1)	96.2 (94.9,97.5)	95.7 (94.3,97.1)	72.1 (69.1,75.2)	70.4 (67.3,73.5)	10.5 (8.4,12.6)	11.0 (8.9,13.1)
**L**	**L**	**H**	**L**	77.6 (74.9,80.2)	70.6 (67.7,73.5)	71.8 (68.9,74.6)	96.9 (95.8,98.0)	93.9 (92.4,95.4)	89.3 (87.3,91.2)	89.7 (87.7,91.6)	69.8 (66.8,72.7)	78.3 (75.7,80.9)
**L**	**L**	**H**	**H**	97.4 (96.3,98.4)	93.2 (91.6,94.8)	94.2 (92.7,95.7)	97.6 (96.6,98.6)	98.2 (97.4,99.1)	95.1 (93.8,96.5)	95.9 (94.6,97.1)	97.8 (96.8,98.7)	98.0 (97.1,98.9)
**L**	**H**	**L**	**H**	33.3 (30.2,36.5)	27.9 (24.8,30.9)	31.2 (28.1,34.3)	95.1 (93.7,96.6)	96.3 (95.1,97.6)	71.2 (68.1,74.2)	94.8 (93.3,96.3)	41.6 (38.3,45.0)	25.4 (22.4,28.3)

vii. Setting 7: $\pi = 0.2, \omega = 0.9, n_{obs} = 5000$

Table vii.1 Number of converged data sets for different sensitivity-specificity combinations

				**Number of converged data sets**
**Specificity**				**Sensitivity: H H L L**
**H**	**H**	**L**	**L**	945
**H**	**H**	**H**	**L**	999
**H**	**H**	**H**	**H**	1000
**L**	**L**	**L**	**L**	671
**L**	**L**	**H**	**L**	985
**L**	**L**	**H**	**H**	1000
**L**	**H**	**L**	**H**	999
**Specificity**				**Sensitivity: H H H L**
**H**	**H**	**L**	**L**	1000
**H**	**H**	**H**	**L**	1000
**H**	**H**	**H**	**H**	1000
**L**	**L**	**L**	**L**	980
**L**	**L**	**H**	**L**	988
**L**	**L**	**H**	**H**	1000
**L**	**H**	**L**	**H**	1000
**Specificity**				**Sensitivity: H H H H**
**H**	**H**	**L**	**L**	1000
**H**	**H**	**H**	**L**	1000
**H**	**H**	**H**	**H**	1000
**L**	**L**	**L**	**L**	993
**L**	**L**	**H**	**L**	999
**L**	**L**	**H**	**H**	1000
**L**	**H**	**L**	**H**	1000
**Specificity**				**Sensitivity: L L L L**
**H**	**H**	**L**	**L**	906
**H**	**H**	**H**	**L**	1000
**H**	**H**	**H**	**H**	999
**L**	**L**	**L**	**L**	614
**L**	**L**	**H**	**L**	993
**L**	**L**	**H**	**H**	996
**L**	**H**	**L**	**H**	999
**Specificity**				**Sensitivity: L L H L**
**H**	**H**	**L**	**L**	1000
**H**	**H**	**H**	**L**	1000

**H**	**H**	**H**	**H**	1000
**L**	**L**	**L**	**L**	990
**L**	**L**	**H**	**L**	992
**L**	**L**	**H**	**H**	914
**L**	**H**	**L**	**H**	1000
**Specificity**				**Sensitivity: L L H H**
**H**	**H**	**L**	**L**	1000
**H**	**H**	**H**	**L**	1000
**H**	**H**	**H**	**H**	998
**L**	**L**	**L**	**L**	995
**L**	**L**	**H**	**L**	998
**L**	**L**	**H**	**H**	684
**L**	**H**	**L**	**H**	1000

Table vii.2 Percentages of the time 95% credible intervals (CrIs) for residual correlations did not included '0' for any pairs, and percentages of the time that the overall χ^2 or G^2 statistic indicated a lack of overall fit

**Sensitivity: H H L L**						
**Specificity**				**Res. Cor.**	**Overall G^2**	**Overall χ^2**
**H**	**H**	**L**	**L**	63.7 (60.5,66.8)	13.9 (11.7,16.2)	13.9 (11.7,16.2)
**H**	**H**	**H**	**L**	36.3 (33.3,39.4)	18.3 (16.0,20.9)	16.9 (14.6,19.4)
**H**	**H**	**H**	**H**	72.1 (69.2,74.9)	84.0 (81.6,86.2)	85.8 (83.5,87.9)
**L**	**L**	**L**	**L**	65.9 (62.1,69.5)	24.0 (20.8,27.4)	24.6 (21.4,28.0)
**L**	**L**	**H**	**L**	58.7 (55.5,61.8)	73.4 (70.5,76.1)	74.3 (71.5,77.0)
**L**	**L**	**H**	**H**	88.4 (86.3,90.3)	99.7 (99.1,99.9)	99.9 (99.4,100.0)
**L**	**H**	**L**	**H**	67.6 (64.6,70.5)	44.1 (41.0,47.3)	44.3 (41.2,47.5)
**Sensitivity: H H H L**						
**Specificity**				**Res. Cor.**	**Overall G^2**	**Overall χ^2**
**H**	**H**	**L**	**L**	40.9 (37.8,44.0)	14.9 (12.7,17.3)	15.1 (12.9,17.5)
**H**	**H**	**H**	**L**	29.6 (26.8,32.5)	35.2 (32.2,38.3)	33.1 (30.2,36.1)
**H**	**H**	**H**	**H**	96.1 (94.7,97.2)	99.3 (98.6,99.7)	99.6 (99.0,99.9)
**L**	**L**	**L**	**L**	70.2 (67.2,73.1)	51.7 (48.6,54.9)	52.7 (49.5,55.8)
**L**	**L**	**H**	**L**	79.3 (76.6,81.7)	96.7 (95.3,97.7)	96.9 (95.6,97.9)
**L**	**L**	**H**	**H**	99.5 (98.8,99.8)	100.0 (99.6,100.0)	100.0 (99.6,100.0)
**L**	**H**	**L**	**H**	90.0 (88.0,91.8)	83.5 (81.1,85.7)	84.1 (81.7,86.3)
**Sensitivity: H H H H**						
**Specificity**				**Res. Cor.**	**Overall G^2**	**Overall χ^2**
**H**	**H**	**L**	**L**	66.2 (63.2,69.1)	29.9 (27.1,32.8)	30.2 (27.4,33.2)
**H**	**H**	**H**	**L**	97.1 (95.9,98.0)	90.4 (88.4,92.2)	90.3 (88.3,92.1)
**H**	**H**	**H**	**H**	100 (99.6,100.0)	100.0 (99.6,100.0)	100.0 (99.6,100.0)

**L**	**L**	**L**	**L**	87.7 (85.5,89.7)	81.1 (78.5,83.5)	82.0 (79.4,84.3)
**L**	**L**	**H**	**L**	99.6 (99.0,99.9)	100.0 (99.6,100.0)	100.0 (99.6,100.0)
**L**	**L**	**H**	**H**	100.0 (99.6,100.0)	100.0 (99.6,100.0)	100.0 (99.6,100.0)
**L**	**H**	**L**	**H**	99.4 (98.7,99.8)	99.4 (98.7,99.8)	99.6 (99.0,99.9)
**Sensitivity: L L L L**						
**Specificity**				**Res. Cor.**	**Overall G^2**	**Overall χ^2**
**H**	**H**	**L**	**L**	80.8 (78.1,83.3)	24.6 (21.8,27.6)	23.7 (21.0,26.6)
**H**	**H**	**H**	**L**	98.3 (97.3,99.0)	88.4 (86.3,90.3)	88.2 (86.0,90.1)
**H**	**H**	**H**	**H**	100.0 (99.6,100.0)	100.0 (99.6,100.0)	100.0 (99.6,100.0)
**L**	**L**	**L**	**L**	97.6 (96.0,98.6)	91.5 (89.0,93.6)	91.5 (89.0,93.6)
**L**	**L**	**H**	**L**	100.0 (99.6,100.0)	100.0 (99.6,100.0)	100.0 (99.6,100.0)
**L**	**L**	**H**	**H**	100.0 (99.6,100.0)	100.0 (99.6,100.0)	100.0 (99.6,100.0)
**L**	**H**	**L**	**H**	100.0 (99.6,100.0)	100.0 (99.6,100.0)	100.0 (99.6,100.0)
**Sensitivity: L L H L**						
**Specificity**				**Res. Cor.**	**Overall G^2**	**Overall χ^2**
**H**	**H**	**L**	**L**	95.2 (93.7,96.4)	66.3 (63.3,69.2)	66.3 (63.3,69.2)
**H**	**H**	**H**	**L**	100.0 (99.6,100.0)	99.6 (99.0,99.9)	99.5 (98.8,99.8)
**H**	**H**	**H**	**H**	100.0 (99.6,100.0)	100.0 (99.6,100.0)	100.0 (99.6,100.0)
**L**	**L**	**L**	**L**	100.0 (99.6,100.0)	100.0 (99.6,100.0)	100.0 (99.6,100.0)
**L**	**L**	**H**	**L**	100.0 (99.6,100.0)	100.0 (99.6,100.0)	100.0 (99.6,100.0)
**L**	**L**	**H**	**H**	100.0 (99.6,100.0)	100.0 (99.6,100.0)	100.0 (99.6,100.0)
**L**	**H**	**L**	**H**	100.0 (99.6,100.0)	100.0 (99.6,100.0)	100.0 (99.6,100.0)
**Sensitivity: L L H H**						
**Specificity**				**Res. Cor.**	**Overall G^2**	**Overall χ^2**
**H**	**H**	**L**	**L**	100.0 (99.6,100.0)	99.8 (99.3,100.0)	99.8 (99.3,100.0)
**H**	**H**	**H**	**L**	100.0 (99.6,100.0)	100.0 (99.6,100.0)	100.0 (99.6,100.0)
**H**	**H**	**H**	**H**	100.0 (99.6,100.0)	100.0 (99.6,100.0)	100.0 (99.6,100.0)
**L**	**L**	**L**	**L**	100.0 (99.6,100.0)	100.0 (99.6,100.0)	100.0 (99.6,100.0)
**L**	**L**	**H**	**L**	100.0 (99.6,100.0)	100.0 (99.6,100.0)	100.0 (99.6,100.0)
**L**	**L**	**H**	**H**	100.0 (99.5,100.0)	100.0 (99.5,100.0)	100.0 (99.5,100.0)
**L**	**H**	**L**	**H**	100.0 (99.6,100.0)	100.0 (99.6,100.0)	100.0 (99.6,100.0)

Table vii.3 Percentages of the time 95% CrIs for residual correlations did not included '0' for each pair, and percentages of the time the pairwise χ^2 or G^2 statistic for each pair indicated a lack of pairwise fit

**Sensitivity: H H L L**										
**Specificity**				**Tool**	**T_1&T_2**	**T_1&T_3**	**T_1&T_4**	**T_2&T_3**	**T_2&T_4**	**T_3&T_4**
**H**	**H**	**L**	**L**	**Res. Cor.**	0.0 (0.0,0.4)	0.2 (0.0,0.8)	0.2 (0.0,0.8)	0.2 (0.0,0.8)	0.0 (0.0,0.4)	63.5 (60.3,66.6)
				**Pairwise G^2**	0.0 (0.0,0.4)	0.0 (0.0,0.4)	0.0 (0.0,0.4)	0.0 (0.0,0.4)	0.0 (0.0,0.4)	5.0 (3.7,6.6)

				Pairwise χ^2	0.0 (0.0,0.4)	0.0 (0.0,0.4)	0.0 (0.0,0.4)	0.0 (0.0,0.4)	0.0 (0.0,0.4)	5.0 (3.7,6.6)
H	H	H	L	Res. Cor.	0.0 (0.0,0.4)	0.0 (0.0,0.4)	0.3 (0.1,0.9)	0.0 (0.0,0.4)	0.4 (0.1,1.0)	35.8 (32.9,38.9)
				Pairwise G^2	0.0 (0.0,0.4)	0.0 (0.0,0.4)	0.0 (0.0,0.4)	0.0 (0.0,0.4)	0.1 (0.0,0.6)	6.5 (5.1,8.2)
				Pairwise χ^2	0.0 (0.0,0.4)	0.0 (0.0,0.4)	0.0 (0.0,0.4)	0.0 (0.0,0.4)	0.1 (0.0,0.6)	6.5 (5.1,8.2)
H	H	H	H	Res. Cor.	0.0 (0.0,0.4)	0.0 (0.0,0.4)	0.0 (0.0,0.4)	0.0 (0.0,0.4)	0.0 (0.0,0.4)	72.1 (69.2,74.9)
				Pairwise G^2	0.0 (0.0,0.4)	0.0 (0.0,0.4)	0.0 (0.0,0.4)	0.0 (0.0,0.4)	0.0 (0.0,0.4)	42.9 (39.8,46.0)
				Pairwise χ^2	0.0 (0.0,0.4)	0.0 (0.0,0.4)	0.0 (0.0,0.4)	0.0 (0.0,0.4)	0.0 (0.0,0.4)	43.0 (39.9,46.1)
L	L	L	L	Res. Cor.	0.0 (0.0,0.5)	7.0 (5.2,9.2)	6.6 (4.8,8.7)	8.6 (6.6,11.0)	6.4 (4.7,8.5)	54.4 (50.5,58.2)
				Pairwise G^2	0.0 (0.0,0.5)	0.9 (0.3,1.9)	0.4 (0.1,1.3)	1.3 (0.6,2.5)	0.9 (0.3,1.9)	5.8 (4.2,7.9)
				Pairwise χ^2	0.0 (0.0,0.5)	0.9 (0.3,1.9)	0.4 (0.1,1.3)	1.3 (0.6,2.5)	0.9 (0.3,1.9)	5.8 (4.2,7.9)
L	L	H	L	Res. Cor.	0.0 (0.0,0.4)	9.6 (7.9,11.7)	16.3 (14.1,18.8)	11.9 (9.9,14.1)	15.2 (13.0,17.6)	31.1 (28.2,34.1)
				Pairwise G^2	0.0 (0.0,0.4)	3.1 (2.1,4.4)	1.7 (1.0,2.7)	4.0 (2.8,5.4)	2.9 (2.0,4.2)	8.3 (6.7,10.2)
				Pairwise χ^2	0.0 (0.0,0.4)	3.2 (2.2,4.6)	1.7 (1.0,2.7)	4.1 (2.9,5.5)	2.9 (2.0,4.2)	8.3 (6.7,10.2)
L	L	H	H	Res. Cor.	10.6 (8.8,12.7)	18.0 (15.7,20.5)	19.0 (16.6,21.6)	20.2 (17.8,22.8)	17.9 (15.6,20.4)	79.9 (77.3,82.3)
				Pairwise G^2	3.2 (2.2,4.5)	5.0 (3.7,6.5)	5.7 (4.3,7.3)	6.4 (5.0,8.1)	5.5 (4.2,7.1)	67.9 (64.9,70.8)
				Pairwise χ^2	3.2 (2.2,4.5)	5.0 (3.7,6.5)	6.3 (4.9,8.0)	7.0 (5.5,8.8)	5.9 (4.5,7.5)	68.1 (65.1,71.0)
L	H	L	H	Res. Cor.	0.0 (0.0,0.4)	27.1 (24.4,30.0)	35.5 (32.6,38.6)	0.0 (0.0,0.4)	0.0 (0.0,0.4)	30.4 (27.6,33.4)
				Pairwise G^2	0.0 (0.0,0.4)	3.3 (2.3,4.6)	11.8 (9.9,14.0)	0.0 (0.0,0.4)	0.0 (0.0,0.4)	5.7 (4.3,7.3)
				Pairwise χ^2	0.0 (0.0,0.4)	3.4 (2.4,4.7)	12.3 (10.3,14.5)	0.0 (0.0,0.4)	0.0 (0.0,0.4)	5.7 (4.3,7.3)
**Sensitivity: H H H L**										
**Specificity**				**Tool**	**T_1&T_2**	**T_1&T_3**	**T_1&T_4**	**T_2&T_3**	**T_2&T_4**	**T_3&T_4**
H	H	L	L	Res. Cor.	0.0 (0.0,0.4)	0.0 (0.0,0.4)	0.0 (0.0,0.4)	0.6 (0.2,1.3)	0.3 (0.1,0.9)	40.1 (37.0,43.2)
				Pairwise G^2	0.0 (0.0,0.4)	0.0 (0.0,0.4)	0.0 (0.0,0.4)	0.0 (0.0,0.4)	0.0 (0.0,0.4)	4.3 (3.1,5.7)
				Pairwise χ^2	0.0 (0.0,0.4)	0.0 (0.0,0.4)	0.0 (0.0,0.4)	0.0 (0.0,0.4)	0.0 (0.0,0.4)	4.3 (3.1,5.7)
H	H	H	L	Res. Cor.	0.0 (0.0,0.4)	0.0 (0.0,0.4)	0.8 (0.3,1.6)	0.0 (0.0,0.4)	1.1 (0.6,2.0)	28.4 (25.6,31.3)
				Pairwise G^2	0.0 (0.0,0.4)	0.0 (0.0,0.4)	0.1 (0.0,0.6)	0.0 (0.0,0.4)	0.1 (0.0,0.6)	10.0 (8.2,12.0)
				Pairwise χ^2	0.0 (0.0,0.4)	0.0 (0.0,0.4)	0.1 (0.0,0.6)	0.0 (0.0,0.4)	0.1 (0.0,0.6)	9.9 (8.1,11.9)
H	H	H	H	Res. Cor.	0.0 (0.0,0.4)	0.0 (0.0,0.4)	0.0 (0.0,0.4)	0.0 (0.0,0.4)	0.0 (0.0,0.4)	96.1 (94.7,97.2)
				Pairwise G^2	0.0 (0.0,0.4)	0.0 (0.0,0.4)	0.0 (0.0,0.4)	0.0 (0.0,0.4)	0.0 (0.0,0.4)	91.5 (89.6,93.2)
				Pairwise χ^2	0.0 (0.0,0.4)	0.0 (0.0,0.4)	0.0 (0.0,0.4)	0.0 (0.0,0.4)	0.0 (0.0,0.4)	91.5 (89.6,93.2)
L	L	L	L	Res. Cor.	0.0 (0.0,0.4)	20.3 (17.8,23.0)	17.7 (15.4,20.2)	20.4 (17.9,23.1)	17.8 (15.4,20.3)	32.1 (29.2,35.2)
				Pairwise G^2	0.0 (0.0,0.4)	5.1 (3.8,6.7)	2.2 (1.4,3.4)	5.3 (4.0,6.9)	1.8 (1.1,2.9)	5.7 (4.3,7.4)
				Pairwise χ^2	0.0 (0.0,0.4)	5.1 (3.8,6.7)	2.2 (1.4,3.4)	5.4 (4.1,7.0)	1.8 (1.1,2.9)	5.7 (4.3,7.4)
L	L	H	L	Res. Cor.	0.0 (0.0,0.4)	28.7 (25.9,31.7)	27.6 (24.9,30.5)	27.0 (24.3,29.9)	25.9 (23.2,28.8)	30.6 (27.7,33.5)
				Pairwise G^2	0.0 (0.0,0.4)	14.8 (12.6,17.1)	4.0 (2.9,5.5)	14.0 (11.9,16.3)	3.8 (2.7,5.2)	11.9 (10.0,14.1)
				Pairwise χ^2	0.0 (0.0,0.4)	15.2 (13.0,17.6)	4.1 (3.0,5.6)	14.7 (12.5,17.0)	3.8 (2.7,5.2)	11.9 (10.0,14.1)
L	L	H	H	Res. Cor.	41.3 (38.2,44.4)	36.5 (33.5,39.6)	21.8 (19.3,24.5)	38.0 (35.0,41.1)	24.6 (22.0,27.4)	98.5 (97.5,99.2)
				Pairwise G^2	13.2 (11.2,15.5)	19.8 (17.4,22.4)	8.3 (6.7,10.2)	18.0 (15.7,20.5)	8.6 (6.9,10.5)	98.1 (97.0,98.9)
				Pairwise χ^2	13.2 (11.2,15.5)	20.7 (18.2,23.3)	8.9 (7.2,10.8)	18.9 (16.5,21.5)	9.1 (7.4,11.1)	98.1 (97.0,98.9)
L	H	L	H	Res. Cor.	0.0 (0.0,0.4)	49.8 (46.7,52.9)	40.0 (36.9,43.1)	0.0 (0.0,0.4)	0.0 (0.0,0.4)	73.0 (70.1,75.7)
				Pairwise G^2	0.0 (0.0,0.4)	12.6 (10.6,14.8)	13.0 (11.0,15.2)	0.0 (0.0,0.4)	0.0 (0.0,0.4)	41.7 (38.6,44.8)

				**Pairwise χ^2**	0.0 (0.0,0.4)	12.8 (10.8,15.0)	13.8 (11.7,16.1)	0.0 (0.0,0.4)	0.0 (0.0,0.4)	41.0 (37.9,44.1)
**Sensitivity: H H H H**										
**Specificity**				**Tool**	**T_1&T_2**	**T_1&T_3**	**T_1&T_4**	**T_2&T_3**	**T_2&T_4**	**T_3&T_4**
**H**	**H**	**L**	**L**	**Res. Cor.**	0.0 (0.0,0.4)	0.8 (0.3,1.6)	1.0 (0.5,1.8)	1.1 (0.6,2.0)	1.2 (0.6,2.1)	65.4 (62.4,68.3)
				**Pairwise G^2**	0.0 (0.0,0.4)	0.1 (0.0,0.6)	0.2 (0.0,0.7)	0.1 (0.0,0.6)	0.5 (0.2,1.2)	20.9 (18.4,23.6)
				**Pairwise χ^2**	0.0 (0.0,0.4)	0.1 (0.0,0.6)	0.2 (0.0,0.7)	0.1 (0.0,0.6)	0.7 (0.3,1.4)	20.9 (18.4,23.6)
**H**	**H**	**H**	**L**	**Res. Cor.**	0.0 (0.0,0.4)	0.0 (0.0,0.4)	1.3 (0.7,2.2)	0.0 (0.0,0.4)	1.9 (1.1,3.0)	96.9 (95.6,97.9)
				**Pairwise G^2**	0.0 (0.0,0.4)	0.0 (0.0,0.4)	0.1 (0.0,0.6)	0.0 (0.0,0.4)	0.3 (0.1,0.9)	86.2 (83.9,88.3)
				**Pairwise χ^2**	0.0 (0.0,0.4)	0.0 (0.0,0.4)	0.1 (0.0,0.6)	0.0 (0.0,0.4)	0.3 (0.1,0.9)	85.9 (83.6,88.0)
**H**	**H**	**H**	**H**	**Res. Cor.**	0.0 (0.0,0.4)	0.0 (0.0,0.4)	0.0 (0.0,0.4)	0.0 (0.0,0.4)	0.0 (0.0,0.4)	100.0 (99.6,100.0)
				**Pairwise G^2**	0.0 (0.0,0.4)	0.0 (0.0,0.4)	0.0 (0.0,0.4)	0.0 (0.0,0.4)	0.0 (0.0,0.4)	100.0 (99.6,100.0)
				**Pairwise χ^2**	0.0 (0.0,0.4)	0.0 (0.0,0.4)	0.0 (0.0,0.4)	0.0 (0.0,0.4)	0.0 (0.0,0.4)	100.0 (99.6,100.0)
**L**	**L**	**L**	**L**	**Res. Cor.**	6.2 (4.8,7.9)	30.0 (27.2,33.0)	29.7 (26.9,32.7)	30.8 (28.0,33.8)	28.7 (25.9,31.6)	61.0 (57.9,64.1)
				**Pairwise G^2**	1.8 (1.1,2.8)	9.1 (7.4,11.0)	8.7 (7.0,10.6)	7.7 (6.1,9.5)	7.9 (6.3,9.7)	24.1 (21.4,26.9)
				**Pairwise χ^2**	1.8 (1.1,2.8)	9.4 (7.6,11.4)	8.7 (7.0,10.6)	7.9 (6.3,9.7)	7.9 (6.3,9.7)	24.1 (21.4,26.9)
**L**	**L**	**H**	**L**	**Res. Cor.**	29.9 (27.1,32.9)	37.8 (34.8,40.9)	36.0 (33.1,39.1)	35.1 (32.2,38.2)	40.3 (37.3,43.5)	98.2 (97.2,98.9)
				**Pairwise G^2**	7.5 (6.0,9.3)	18.3 (16.0,20.9)	10.1 (8.3,12.1)	18.0 (15.7,20.5)	9.3 (7.6,11.3)	91.6 (89.7,93.2)
				**Pairwise χ^2**	7.5 (6.0,9.3)	19.1 (16.7,21.7)	10.2 (8.4,12.3)	18.7 (16.3,21.3)	9.8 (8.0,11.8)	91.4 (89.5,93.1)
**L**	**L**	**H**	**H**	**Res. Cor.**	61.7 (58.6,64.7)	41.8 (38.7,44.9)	39.7 (36.7,42.8)	43.3 (40.2,46.4)	42.0 (38.9,45.1)	100.0 (99.6,100.0)
				**Pairwise G^2**	22.3 (19.8,25.0)	20.0 (17.6,22.6)	20.3 (17.8,22.9)	21.9 (19.4,24.6)	19.9 (17.5,22.5)	100.0 (99.6,100.0)
				**Pairwise χ^2**	22.3 (19.8,25.0)	20.8 (18.3,23.4)	21.1 (18.6,23.8)	22.7 (20.1,25.4)	20.8 (18.3,23.4)	100.0 (99.6,100.0)
**L**	**H**	**L**	**H**	**Res. Cor.**	0.8 (0.3,1.6)	48.1 (45.0,51.2)	67.8 (64.8,70.7)	0.3 (0.1,0.9)	0.0 (0.0,0.4)	96.6 (95.3,97.6)
				**Pairwise G^2**	0.1 (0.0,0.6)	11.8 (9.9,14.0)	40.7 (37.6,43.8)	0.0 (0.0,0.4)	0.0 (0.0,0.4)	86.9 (84.6,88.9)
				**Pairwise χ^2**	0.1 (0.0,0.6)	11.8 (9.9,14.0)	41.5 (38.4,44.6)	0.0 (0.0,0.4)	0.0 (0.0,0.4)	86.7 (84.4,88.7)
**Sensitivity: L L L L**										
**Specificity**				**Tool**	**T_1&T_2**	**T_1&T_3**	**T_1&T_4**	**T_2&T_3**	**T_2&T_4**	**T_3&T_4**
**H**	**H**	**L**	**L**	**Res. Cor.**	0.0 (0.0,0.4)	2.1 (1.3,3.3)	2.3 (1.4,3.5)	2.4 (1.5,3.7)	2.3 (1.4,3.5)	79.6 (76.8,82.2)
				**Pairwise G^2**	0.0 (0.0,0.4)	0.3 (0.1,1.0)	0.4 (0.1,1.1)	0.3 (0.1,1.0)	0.4 (0.1,1.0)	14.5 (12.2,16.9)
				**Pairwise χ^2**	0.0 (0.0,0.4)	0.3 (0.1,1.0)	0.4 (0.1,1.1)	0.3 (0.1,1.0)	0.4 (0.1,1.0)	14.5 (12.2,16.9)
**H**	**H**	**H**	**L**	**Res. Cor.**	0.0 (0.0,0.4)	0.4 (0.1,1.0)	2.1 (1.3,3.2)	0.7 (0.3,1.4)	2.3 (1.5,3.4)	98.3 (97.3,99.0)
				**Pairwise G^2**	0.0 (0.0,0.4)	0.1 (0.0,0.6)	0.5 (0.2,1.2)	0.3 (0.1,0.9)	0.5 (0.2,1.2)	78.4 (75.7,80.9)
				**Pairwise χ^2**	0.0 (0.0,0.4)	0.1 (0.0,0.6)	0.5 (0.2,1.2)	0.3 (0.1,0.9)	0.5 (0.2,1.2)	78.4 (75.7,80.9)
**H**	**H**	**H**	**H**	**Res. Cor.**	0.0 (0.0,0.4)	1.4 (0.8,2.3)	1.4 (0.8,2.3)	1.1 (0.6,2.0)	0.6 (0.2,1.3)	100.0 (99.6,100.0)
				**Pairwise G^2**	0.0 (0.0,0.4)	0.3 (0.1,0.9)	0.5 (0.2,1.2)	0.2 (0.0,0.7)	0.1 (0.0,0.6)	100.0 (99.6,100.0)
				**Pairwise χ^2**	0.0 (0.0,0.4)	0.3 (0.1,0.9)	0.5 (0.2,1.2)	0.2 (0.0,0.7)	0.1 (0.0,0.6)	100.0 (99.6,100.0)
**L**	**L**	**L**	**L**	**Res. Cor.**	0.0 (0.0,0.6)	38.6 (34.7,42.6)	35.2 (31.4,39.1)	41.7 (37.8,45.7)	38.1 (34.3,42.1)	85.2 (82.1,87.9)
				**Pairwise G^2**	0.0 (0.0,0.6)	11.9 (9.4,14.7)	12.2 (9.7,15.1)	13.2 (10.6,16.1)	10.6 (8.3,13.3)	23.8 (20.5,27.3)
				**Pairwise χ^2**	0.0 (0.0,0.6)	11.9 (9.4,14.7)	12.2 (9.7,15.1)	13.2 (10.6,16.1)	10.6 (8.3,13.3)	23.9 (20.6,27.5)
**L**	**L**	**H**	**L**	**Res. Cor.**	0.0 (0.0,0.4)	99.7 (99.1,99.9)	61.5 (58.4,64.6)	99.7 (99.1,99.9)	62.2 (59.1,65.3)	99.9 (99.4,100.0)
				**Pairwise G^2**	0.0 (0.0,0.4)	96.6 (95.2,97.6)	26.6 (23.9,29.5)	97.6 (96.4,98.4)	23.9 (21.2,26.6)	97.0 (95.7,98.0)
				**Pairwise χ^2**	0.0 (0.0,0.4)	96.8 (95.5,97.8)	26.6 (23.9,29.5)	97.7 (96.5,98.5)	24.1 (21.4,26.9)	97.0 (95.7,98.0)

**L**	**L**	**H**	**H**	**Res. Cor.**	89.7 (87.6,91.5)	100.0 (99.6,100.0)	100.0 (99.6,100.0)	100.0 (99.6,100.0)	100.0 (99.6,100.0)	100.0 (99.6,100.0)
				**Pairwise G^2**	84.8 (82.5,87.0)	99.4 (98.7,99.8)	99.8 (99.3,100.0)	99.9 (99.4,100.0)	99.9 (99.4,100.0)	100.0 (99.6,100.0)
				**Pairwise χ^2**	84.8 (82.5,87.0)	99.5 (98.8,99.8)	99.8 (99.3,100.0)	100.0 (99.6,100.0)	99.9 (99.4,100.0)	100.0 (99.6,100.0)
**L**	**H**	**L**	**H**	**Res. Cor.**	0.0 (0.0,0.4)	73.3 (70.4,76.0)	100.0 (99.6,100.0)	0.5 (0.2,1.2)	0.0 (0.0,0.4)	99.1 (98.3,99.6)
				**Pairwise G^2**	0.0 (0.0,0.4)	29.2 (326.4,32.2)	100.0 (99.6,100.0)	0.1 (0.0,0.6)	0.0 (0.0,0.4)	85.3 (82.9,87.4)
				**Pairwise χ^2**	0.0 (0.0,0.4)	29.4 (26.6,32.4)	100.0 (99.6,100.0)	0.1 (0.0,0.6)	0.0 (0.0,0.4)	85.4 (83.0,87.5)
**Sensitivity: L L H L**										
**Specificity**				**Tool**	**T_1&T_2**	**T_1&T_3**	**T_1&T_4**	**T_2&T_3**	**T_2&T_4**	**T_3&T_4**
**H**	**H**	**L**	**L**	**Res. Cor.**	0.0 (0.0,0.4)	12.2 (10.2,14.4)	3.0 (2.0,4.3)	10.0 (8.2,12.0)	3.9 (2.8,5.3)	94.1 (92.5,95.5)
				**Pairwise G^2**	0.0 (0.0,0.4)	2.8 (1.9,4.0)	0.6 (0.2,1.3)	2.0 (1.2,3.1)	0.6 (0.2,1.3)	57.4 (54.3,60.5)
				**Pairwise χ^2**	0.0 (0.0,0.4)	3.0 (2.0,4.3)	0.6 (0.2,1.3)	2.3 (1.5,3.4)	0.6 (0.2,1.3)	57.4 (54.3,60.5)
**H**	**H**	**H**	**L**	**Res. Cor.**	0.0 (0.0,0.4)	4.6 (3.4,6.1)	3.8 (2.7,5.2)	3.7 (2.6,5.1)	3.1 (2.1,4.4)	100.0 (99.6,100.0)
				**Pairwise G^2**	0.0 (0.0,0.4)	1.5 (0.8,2.5)	0.5 (0.2,1.2)	1.2 (0.6,2.1)	0.5 (0.2,1.2)	98.5 (97.5,99.2)
				**Pairwise χ^2**	0.0 (0.0,0.4)	1.5 (0.8,2.5)	0.5 (0.2,1.2)	1.3 (0.7,2.2)	0.5 (0.2,1.2)	98.5 (97.5,99.2)
**H**	**H**	**H**	**H**	**Res. Cor.**	0.0 (0.0,0.4)	9.2 (7.5,11.2)	3.3 (2.3,4.6)	8.6 (6.9,10.5)	2.7 (1.8,3.5)	100.0 (99.6,100.0)
				**Pairwise G^2**	0.0 (0.0,0.4)	3.4 (2.4,4.7)	0.8 (0.3,1.6)	3.2 (2.2,4.5)	0.6 (0.2,1.3)	100.0 (99.6,100.0)
				**Pairwise χ^2**	0.0 (0.0,0.4)	3.6 (2.5,4.9)	0.8 (0.3,1.6)	3.2 (2.2,4.5)	0.6 (0.2,1.3)	100.0 (99.6,100.0)
**L**	**L**	**L**	**L**	**Res. Cor.**	0.0 (0.0,0.4)	97.2 (95.9,98.1)	55.3 (52.1,58.4)	96.3 (94.9,97.4)	59.4 (56.3,62.5)	99.1 (98.3,99.6)
				**Pairwise G^2**	0.0 (0.0,0.4)	85.6 (83.2,87.7)	21.4 (18.9,24.1)	82.6 (80.1,84.9)	25.3 (22.6,28.1)	84.3 (81.9,86.6)
				**Pairwise χ^2**	0.0 (0.0,0.4)	86.1 (83.7,88.2)	21.4 (18.9,24.1)	82.7 (80.2,85.0)	25.3 (22.6,28.1)	84.3 (81.9,86.6)
**L**	**L**	**H**	**L**	**Res. Cor.**	0.4 (0.1,1.0)	100.0 (99.6,100.0)	78.9 (76.3,81.4)	100.0 (99.6,100.0)	80.8 (78.3,83.3)	100.0 (99.6,100.0)
				**Pairwise G^2**	0.6 (0.2,1.3)	100.0 (99.6,100.0)	43.2 (40.1,46.4)	100.0 (99.6,100.0)	44.6 (41.4,47.7)	100.0 (99.6,100.0)
				**Pairwise χ^2**	0.6 (0.2,1.3)	100.0 (99.6,100.0)	43.2 (40.1,46.4)	100.0 (99.6,100.0)	44.6 (41.4,47.7)	100.0 (99.6,100.0)
**L**	**L**	**H**	**H**	**Res. Cor.**	100.0 (99.6,100.0)	98.1 (97.0,98.9)	99.8 (99.2,100.0)	98.1 (97.0,98.9)	99.6 (98.9,99.9)	97.7 (96.5,98.6)
				**Pairwise G^2**	0.6 (0.2,1.3)	100.0 (99.6,100.0)	43.2 (40.1,46.4)	100.0 (99.6,100.0)	44.6 (41.4,47.7)	100.0 (99.6,100.0)
				**Pairwise χ^2**	0.6 (0.2,1.3)	100.0 (99.6,100.0)	43.2 (40.1,46.4)	100.0 (99.6,100.0)	44.6 (41.4,47.7)	100.0 (99.6,100.0)
**L**	**H**	**L**	**H**	**Res. Cor.**	0.2 (0.0,0.7)	99.8 (99.3,100.0)	100.0 (99.6,100.0)	3.8 (2.7,5.2)	0.1 (0.0,0.6)	100.0 (99.6,100.0)
				**Pairwise G^2**	0.2 (0.0,0.7)	97.2 (96.0,98.1)	100.0 (99.6,100.0)	0.8 (0.3,1.6)	0.1 (0.0,0.6)	100.0 (99.6,100.0)
				**Pairwise χ^2**	0.2 (0.0,0.7)	97.3 (96.1,98.2)	100.0 (99.6,100.0)	0.9 (0.4,1.7)	0.1 (0.0,0.6)	100.0 (99.6,100.0)
**Sensitivity: L L H H**										
**Specificity**				**Tool**	**T_1&T_2**	**T_1&T_3**	**T_1&T_4**	**T_2&T_3**	**T_2&T_4**	**T_3&T_4**
**H**	**H**	**L**	**L**	**Res. Cor.**	0.0 (0.0,0.4)	12.7 (10.7,14.9)	13.5 (11.4,15.8)	12.4 (10.4,14.6)	14.2 (12.1,16.5)	100.0 (99.6,100.0)
				**Pairwise G^2**	0.0 (0.0,0.4)	2.9 (2.0,4.1)	2.7 (1.8,3.9)	2.8 (1.9,4.0)	2.9 (2.0,4.1)	100.0 (99.6,100.0)
				**Pairwise χ^2**	0.0 (0.0,0.4)	2.9 (2.0,4.1)	2.8 (1.9,4.0)	2.8 (1.9,4.0)	3.4 (2.4,4.7)	100.0 (99.6,100.0)
**H**	**H**	**H**	**L**	**Res. Cor.**	0.0 (0.0,0.4)	6.7 (5.2,8.4)	16.5 (14.3,18.9)	6.0 (4.6,7.7)	14.3 (12.2,16.6)	100.0 (99.6,100.0)
				**Pairwise G^2**	0.0 (0.0,0.4)	2.6 (1.7,3.8)	4.7 (3.5,6.2)	1.6 (0.9,2.6)	4.2 (3.0,5.6)	100.0 (99.6,100.0)
				**Pairwise χ^2**	0.0 (0.0,0.4)	2.6 (1.7,3.8)	5.2 (3.9,6.8)	1.6 (0.9,2.6)	4.3 (3.1,5.7)	100.0 (99.6,100.0)
**H**	**H**	**H**	**H**	**Res. Cor.**	0.2 (0.0,0.7)	23.4 (20.8,26.2)	21.7 (19.2,24.4)	25.7 (23.0,28.5)	22.9 (20.4,25.7)	100.0 (99.6,100.0)
				**Pairwise G^2**	0.0 (0.0,0.4)	11.0 (9.1,13.1)	10.9 (9.1,13.0)	12.6 (10.6,14.8)	10.6 (8.8,12.7)	100.0 (99.6,100.0)
				**Pairwise χ^2**	0.0 (0.0,0.4)	11.3 (9.4,13.5)	11.1 (9.2,13.2)	13.0 (11.0,15.3)	11.0 (9.1,13.1)	100.0 (99.6,100.0)
**L**	**L**	**L**	**L**	**Res. Cor.**	69.1 (66.2,72.0)	99.8 (99.3,100.0)	99.7 (99.1,99.9)	99.9 (99.4,100.0)	99.9 (99.4,100.0)	100.0 (99.6,100.0)

				**Pairwise G²**	56.5 (53.3,59.6)	96.7 (95.4,97.7)	96.1 (94.7,97.2)	96.2 (94.8,97.3)	96.8 (95.5,97.8)	100.0 (99.6,100.0)
				**Pairwise χ²**	56.5 (53.3,59.6)	96.8 (95.5,97.8)	96.1 (94.7,97.2)	96.2 (94.8,97.3)	96.9 (95.6,97.9)	100.0 (99.6,100.0)
**L**	**L**	**H**	**L**	**Res. Cor.**	100.0 (99.6,100.0)	100.0 (99.6,100.0)	99.9 (99.4,100.0)	100.0 (99.6,100.0)	100.0 (99.6,100.0)	100.0 (99.6,100.0)
				**Pairwise G²**	98.4 (97.4,99.1)	100.0 (99.6,100.0)	99.0 (98.2,99.5)	100.0 (99.6,100.0)	99.3 (98.6,99.7)	100.0 (99.6,100.0)
				**Pairwise χ²**	98.4 (97.4,99.1)	100.0 (99.6,100.0)	99.0 (98.2,99.5)	100.0 (99.6,100.0)	99.3 (98.6,99.7)	100.0 (99.6,100.0)
**L**	**L**	**H**	**H**	**Res. Cor.**	100.0 (99.5,100.0)	2.0 (1.1,3.4)	1.9 (1.0,3.2)	2.3 (1.3,3.8)	2.0 (1.1,3.4)	0.1 (0.0,0.8)
				**Pairwise G²**	100.0 (99.5,100.0)	0.9 (0.3,1.9)	0.7 (0.2,1.7)	0.1 (0.0,0.8)	0.4 (0.1,1.3)	0.1 (0.0,0.8)
				**Pairwise χ²**	100.0 (99.5,100.0)	0.9 (0.3,1.9)	0.7 (0.2,1.7)	0.1 (0.0,0.8)	0.4 (0.1,1.3)	0.1 (0.0,0.8)
**L**	**H**	**L**	**H**	**Res. Cor.**	11.1 (9.2,13.2)	99.9 (99.4,100.0)	100.0 (99.6,100.0)	9.8 (8.0,11.8)	0.1 (0.0,0.6)	100.0 (99.6,100.0)
				**Pairwise G²**	5.7 (4.3,7.3)	97.9 (96.8,98.7)	100.0 (99.6,100.0)	3.1 (2.1,4.4)	0.0 (0.0,0.4)	100.0 (99.6,100.0)
				**Pairwise χ²**	5.5 (4.2,7.1)	97.9 (96.8,98.7)	100.0 (99.6,100.0)	3.4 (2.4,4.7)	0.0 (0.0,0.4)	100.0 (99.6,100.0)

Table vii.4 Mean absolute biases (x10⁻²) of posterior medians of each parameter obtained from the conditional independence model

**Sensitivity: H H L L**												
**Specificity**				π	Se_1	Se_2	Se_3	Se_4	Sp_1	Sp_2	Sp_3	Sp_4
**H**	**H**	**L**	**L**	-0.9 (-0.9,-0.8)	6.9 (6.8,7.0)	7.0 (6.9,7.1)	-0.4 (-0.5,-0.3)	-0.2 (-0.3,-0.1)	0.7 (0.7,0.8)	0.7 (0.7,0.8)	-0.3 (-0.3,-0.2)	-0.3 (-0.3,-0.2)
**H**	**H**	**H**	**L**	-1.7 (-1.7,-1.7)	8.8 (8.8,8.8)	8.8 (8.8,8.9)	-0.3 (-0.4,-0.2)	-0.1 (-0.2,0.0)	0.3 (0.2,0.3)	0.3 (0.2,0.3)	-1.1 (-1.1,-1.1)	-0.5 (-0.5,-0.4)
**H**	**H**	**H**	**H**	-1.8 (-1.8,-1.8)	9.0 (9.0,9.1)	9.0 (9.0,9.1)	-0.2 (-0.3,0.0)	-0.1 (-0.2,0.0)	0.2 (0.2,0.2)	0.2 (0.2,0.2)	-1.1 (-1.2,-1.1)	-1.1 (-1.2,-1.1)
**L**	**L**	**L**	**L**	1.4 (1.3,1.5)	5.6 (5.4,5.7)	5.5 (5.3,5.6)	-1.7 (-1.9,-1.5)	-1.6 (-1.8,-1.5)	2.2 (2.1,2.3)	2.4 (2.2,2.4)	-0.1 (-0.2,0.0)	-0.1 (-0.2,0.0)
**L**	**L**	**H**	**L**	-1.1 (-1.1,-1.0)	8.9 (8.8,8.9)	8.8 (8.8,8.9)	-2.4 (-2.6,-2.2)	-0.6 (-0.7,-0.5)	1.4 (1.3,1.4)	1.4 (1.3,1.5)	-1.3 (-1.3,-1.3)	-0.5 (-0.5,-0.4)
**L**	**L**	**H**	**H**	-1.9 (-2.0,-1.9)	9.2 (9.2,9.2)	9.2 (9.2,9.2)	-0.5 (-0.6,-0.3)	-0.5 (-0.6,-0.4)	0.8 (0.9,0.9)	0.8 (0.9,0.9)	-1.3 (-1.4,-1.3)	-1.3 (-1.4,-1.3)
**L**	**H**	**L**	**H**	-1.5 (-1.6,-1.5)	8.9 (8.8,8.9)	8.7 (8.6,8.7)	-0.3 (-0.4,-0.2)	-0.6 (-0.7,-0.5)	1.0 (1.0,1.1)	0.3 (0.3,0.4)	-0.5 (-0.5,-0.5)	-1.1 (-1.2,-1.1)
**Sensitivity: H H H L**												
**Specificity**				π	Se_1	Se_2	Se_3	Se_4	Sp_1	Sp_2	Sp_3	Sp_4
**H**	**H**	**L**	**L**	-1.5 (-1.5,-1.4)	8.4 (8.3,8.4)	8.3 (8.3,8.4)	-0.3 (-0.4,-0.3)	-0.1 (-0.2,0.0)	0.4 (0.4,0.4)	0.4 (0.3,0.4)	-1.0 (-1.0,-0.9)	-0.5 (-0.5,-0.4)
**H**	**H**	**H**	**L**	-1.9 (-1.9,-1.8)	9.2 (9.2,9.3)	9.2 (9.2,9.2)	-0.1 (-0.2,0.0)	-0.1 (-0.2,0.0)	0.2 (0.1,0.2)	0.2 (0.1,0.2)	-1.9 (-1.9,-1.9)	-0.5 (-0.6,-0.5)
**H**	**H**	**H**	**H**	-1.9 (-1.9,-1.9)	9.1 (9.1,9.2)	9.2 (9.1,9.2)	-0.2 (-0.2,-0.1)	0.0 (-0.1,0.1))	0.2 (0.1,0.2)	0.2 (0.2,0.2)	-1.9 (-1.9,-1.9)	-1.2 (-1.2,-1.2)
**L**	**L**	**L**	**L**	-0.5 (-0.6,-0.5)	8.0 (8.0,8.1)	8.0 (8.0,8.1)	-2.7 (-2.8,-2.5)	-0.6 (-0.8,-0.5)	1.5 (1.5,1.6)	1.6 (1.5,1.6)	-1.0 (-1.1,-1.0)	-0.3 (-0.3,-0.2)
**L**	**L**	**H**	**L**	-1.6 (-1.6,-1.5)	9.4 (9.4,9.4)	9.4 (9.4,9.4)	-3.1 (-3.3,-2.9)	0.1 (0.0,0.2)	1.1 (1.0,1.2)	1.1 (1.1,1.2)	-2.3 (-2.3,-2.3)	-0.4 (-0.5,-0.4)
**L**	**L**	**H**	**H**	-2.3 (-2.4,-2.3)	9.4 (9.4,9.4)	9.4 (9.4,9.4)	0.3 (0.2,0.4)	0.6 (0.5,0.7)	0.6 (0.5,0.6)	0.5 (0.5,0.6)	-2.2 (-2.3,-2.2)	-1.3 (-1.4,-1.3)
**L**	**H**	**L**	**H**	-1.7 (-1.8,-1.8)	9.0 (9.0,9.0)	9.0 (8.9,9.0)	-0.3 (-0.4,-0.2)	-0.4 (-0.5,-0.2)	0.9 (0.9,1.0)	0.2 (0.2,0.3)	-1.1 (-1.2,-1.1)	-1.2 (-1.2,-1.2)
**Sensitivity: H H H H**												
**Specificity**				π	Se_1	Se_2	Se_3	Se_4	Sp_1	Sp_2	Sp_3	Sp_4
**H**	**H**	**L**	**L**	-1.7 (-1.7,-1.6)	8.8 (8.7,8.8)	8.8 (8.7,8.8)	-0.1 (-0.2,-0.1)	-0.2 (-0.3,-0.1)	0.3 (0.2,0.3)	0.3 (0.2,0.3)	-1.1 (-1.1,-1.0)	-1.1 (-1.1,-1.0)
**H**	**H**	**H**	**L**	-1.9 (-1.9,-1.8)	9.2 (9.2,9.2)	9.2 (9.2,9.2)	-0.2 (-0.2,-0.1)	0.0 (-0.1,0.0)	0.1 (0.1,0.2)	0.1 (0.1,0.2)	-1.9 (-1.9,-1.9)	-1.2 (-1.2,-1.1)
**H**	**H**	**H**	**H**	-1.9 (-1.9,-1.8)	9.1 (9.1,9.1)	9.1 (9.1,9.1)	-0.1 (-0.2,0.0)	0.0 (-0.1,0.0)	0.2 (0.1,0.2)	0.2 (0.1,0.2)	-1.9 (-1.9,-1.9)	-1.9 (-1.9,-1.9)
**L**	**L**	**L**	**L**	-1.5 (-1.5,-1.4)	8.7 (8.6,8.7)	8.7 (8.6,8.7)	-0.7 (-0.9,-0.6)	-0.7 (-0.8,-0.6)	1.1 (1.0,1.1)	1.0 (1.0,1.1)	-1.0 (-1.1,-1.0)	-1.1 (-1.2,-1.1)
**L**	**L**	**H**	**L**	-2.2 (-2.2,-2.1)	9.4 (9.4,9.4)	9.4 (9.4,9.4)	-0.4 (-0.5,-0.2)	0.6 (0.5,0.7)	0.7 (0.7,0.8)	0.7 (0.7,0.8)	-2.2 (-2.3,-2.2)	-1.2 (-1.3,-1.2)

**L**	**L**	**H**	**H**	-2.5 (-2.6,-2.5)	9.4 (9.3,9.4)	9.4 (9.3,9.4)	1.2 (1.1,1.3)	1.2 (1.2,1.3)	0.4 (0.4,0.5)	0.5 (0.4,0.5)	-2.2 (-2.3,-2.2)	-2.2 (-2.3,-2.2)
**L**	**H**	**L**	**H**	-1.9 (-2.0,-1.9)	9.2 (9.1,9.2)	9.4 (9.3,9.4)	0.0 (-0.1,0.1)	-0.2 (-0.3,-0.1)	0.8 (0.8,0.9)	0.1 (0.1,0.2)	-1.2 (-1.2,-1.1)	-2.0 (-2.0,-1.9)
**Sensitivity: L L L L**												
**Specificity**				π	Se_1	Se_2	Se_3	Se_4	Sp_1	Sp_2	Sp_3	Sp_4
**H**	**H**	**L**	**L**	-7.2 (-7.3,-7.2)	35.8 (35.7,35.9)	35.8 (35.7,35.9)	-0.4 (-0.6,-0.3)	-0.5 (-0.6,-0.4)	1.0 (1.0,1.0)	1.0 (1.0,1.0)	-1.8 (-1.8,-1.7)	-1.7 (-1.8,-1.7)
**H**	**H**	**H**	**L**	-8.1 (-8.1,-8.1)	38.5 (38.5,38.6)	38.5 (38.5,38.6)	-0.6 (-0.7,-0.4)	-0.2 (-0.3,-0.1)	0.6 (0.5,0.6)	0.6 (0.6,0.6)	-4.7 (-4.7,-4.7)	-1.9 (-1.9,-1.8)
**H**	**H**	**H**	**H**	-8.1 (-8.1,-8.0)	38.4 (38.3,38.4)	38.4 (38.4,38.5)	-0.4 (-0.5,-0.3)	-0.4 (-0.5,-0.3)	0.6 (0.5,0.6)	0.6 (0.6,0.6)	-4.7 (-4.7,-4.7)	-4.7 (-4.7,-4.6)
**L**	**L**	**L**	**L**	-4.2 (-4.3,-4.1)	35.7 (35.6,35.8)	35.7 (35.5,35.8)	-5.1 (-5.3,-4.8)	-5.1 (-5.3,-4.9)	5.5 (5.5,5.6)	5.6 (5.5,5.7)	-2.0 (-2.0,-1.9)	-2.0 (-2.0,-1.9)
**L**	**L**	**H**	**L**	-6.3 (-6.4,-6.3)	39.1 (39.1,39.1)	39.1 (39.1,39.1)	-11.2 (-11.4,-11.0)	-2.9 (-3.0,-2.7)	4.7 (4.6,4.7)	4.7 (4.6,4.8)	-5.4 (-5.5,-5.4)	-2.0 (-2.0,-1.9)
**L**	**L**	**H**	**H**	-8.3 (-8.4,-8.3)	39.4 (39.4,39.4)	39.4 (39.4,39.4)	-3.6 (-3.8,-3.3)	-3.5 (-3.8,-3.3)	3.3 (3.3,3.4)	3.3 (3.3,3.4)	-5.2 (-5.3,-5.2)	-5.2 (-5.2,-5.2)
**L**	**H**	**L**	**H**	-7.9 (-7.9,-7.9)	38.2 (38.1,38.3)	39.2 (39.2,39.2)	-0.8 (-0.9,-0.6)	-2.2 (-2.4,-2.1)	3.5 (3.4,3.5)	0.9 (0.8,0.9)	-1.9 (-2.0,-1.9,)	-4.8 (-4.9,-4.8)
**Sensitivity: L L H L**												
**Specificity**				π	Se_1	Se_2	Se_3	Se_4	Sp_1	Sp_2	Sp_3	Sp_4
**H**	**H**	**L**	**L**	-7.9 (-8.0,-7.9)	37.9 (37.9,38.0)	38.0 (37.9,38.1)	-0.5 (-0.6,-0.5)	-0.1 (-0.2,0.0)	0.7 (0.6,0.7)	0.7 (0.6,0.7)	-4.6 (-4.6,-4.5)	-1.9 (-1.9,-1.8)
**H**	**H**	**H**	**L**	-8.3 (-8.3,-8.2)	39.1 (39.1,39.2)	39.2 (39.1,39.2)	-0.6 (-0.7,-0.5)	-0.1 (-0.2,0.1)	0.5 (0.4,0.5)	0.5 (0.4,0.5)	-7.6 (-7.6,-7.5)	-1.9 (-2.0,-1.9)
**H**	**H**	**H**	**H**	-8.1 (-8.2,-8.1)	38.3 (38.2,38.3)	38.3 (38.3,38.4)	-0.3 (-0.4,-0.2)	0.2 (0.1,0.3)	0.5 (0.5,0.6)	0.6 (0.5,0.6)	-7.4 (-7.4,-7.4)	-4.6 (-4.6,-4.6)
**L**	**L**	**L**	**L**	-5.9 (-6.0,-5.9)	38.6 (38.5,38.6)	38.6 (38.5,38.6)	-11.2 (-11.4,-11.0)	-3.1 (-3.3,-3.0)	4.9 (4.8,4.9)	4.8 (4.8,4.9)	-5.3 (-5.3,-5.2)	-1.9 (-2.0,-1.9)
**L**	**L**	**H**	**L**	-6.9 (-6.9,-6.8)	39.6 (39.6,39.6)	39.6 (39.6,39.6)	-16.2 (-16.5,-16.0)	-0.7 (-0.9,-0.5)	4.4 (4.3,4.4)	4.4 (4.3,4.4)	-8.8 (-8.8,-8.8)	-1.7 (-1.8,-1.7)
**L**	**L**	**H**	**H**	-9.6 (-9.6,-9.5)	38.7 (38.3,39.1)	38.7 (38.4,39.1)	1.9 (1.7,2.2)	3.8 (3.6,4.0)	2.3 (2.2,2.3)	2.3 (2.2,2.4)	-8.3 (-8.4,-8.3)	-4.9 (-5.0,-4.8)
**L**	**H**	**L**	**H**	-8.2 (-8.2,-8.1)	38.2 (38.1,38.2)	39.3 (39.3,39.3)	-0.7 (-0.8,-0.6)	-1.0 (-1.2,-0.9)	3.3 (3.2,3.3)	0.6 (0.6,0.6)	-4.7 (-4.7,-4.7)	-4.8 (-4.9,-4.8)
**Sensitivity: L L H H**												
**Specificity**				π	Se_1	Se_2	Se_3	Se_4	Sp_1	Sp_2	Sp_3	Sp_4
**H**	**H**	**L**	**L**	-8.1 (-8.1,-8.0)	38.3 (38.2,38.4)	38.3 (38.2,38.4)	-0.3 (-0.4,-0.2)	-0.3 (-0.4,-0.2)	0.6 (0.6,0.6)	0.6 (0.6,0.6)	-4.6 (-4.7,-4.6)	-4.6 (-4.7,-4.6)
**H**	**H**	**H**	**L**	-8.2 (-8.2,-8.2)	38.7 (38.6,38.7)	38.7 (38.6,38.7)	-0.3 (-0.3,-0.2)	0.1 (0.0,0.2)	0.5 (0.5,0.5)	0.5 (0.5,0.5)	-7.5 (-7.5,-7.4)	-4.7 (-4.7,-4.6)
**H**	**H**	**H**	**H**	-8.0 (-8.0,-7.9)	37.5 (37.4,37.5)	37.5 (37.4,37.5)	0.1 (0.0,0.2)	0.1 (0.0,0.2)	0.6 (0.6,0.6)	0.6 (0.5,0.6)	-7.2 (-7.3,-7.2)	-7.2 (-7.3,-7.2)
**L**	**L**	**L**	**L**	-7.7 (-7.8,-7.7)	39.2 (39.1,39.2)	39.2 (39.2,39.2)	-3.8 (-4.0,-3.6)	-3.8 (-4.0,-3.6)	3.7 (3.6,3.7)	3.7 (3.7,3.8)	-5.0 (-5.0,-4.9)	-5.0 (-5.0,-5.0)
**L**	**L**	**H**	**L**	-9.2 (-9.3,-9.2)	39.6 (39.6,39.6)	39.6 (39.6,39.6)	-1.9 (-2.2,-1.7)	3.7 (3.6,3.8)	2.7 (2.6,2.7)	2.7 (2.6,2.7)	-8.6 (-8.6,-8.5)	-4.8 (-4.8,-4.7)
**L**	**L**	**H**	**H**	-0.3 (-0.4,-0.2)	2.0 (1.8,2.2)	2.0 (1.9,2.2)	-0.1 (-0.3,0.2)	0.2 (0.0,0.5)	0.4 (0.4,0.5)	0.4 (0.4,0.5)	-0.3 (-0.4,-0.2)	-0.2 (-0.3,-0.2)
**L**	**H**	**L**	**H**	-8.4 (-8.4,-8.4)	38.2 (38.2,38.3)	39.6 (39.6,39.6)	0.6 (0.5,0.7)	-0.6 (-0.7,-0.5)	3.1 (3.1,3.2)	0.4 (0.3,0.4)	-4.7 (-4.7,-4.6)	-7.7 (-7.8,-7.7)

Table vii.5 Coverages of the 95% CrIs for each model parameter

**Sensitivity: H H L L**												
**Specificity**				π	Se_1	Se_2	Se_3	Se_4	Sp_1	Sp_2	Sp_3	Sp_4
**H**	**H**	**L**	**L**	91.0 (89.2,92.8)	27.3 (24.5,30.1)	24.7 (21.9,27.4)	94.6 (93.2,96.0)	95.1 (93.8,96.5)	84.4 (82.1,86.8)	85.6 (83.4,87.8)	94.4 (92.9,95.9)	93.2 (91.6,94.8)
**H**	**H**	**H**	**L**	23.6 (21.0,26.3)	0.0 (0.0,0.0)	0.0 (0.0,0.0)	94.8 (93.4,96.2)	94.8 (93.4,96.2)	92.9 (91.3,94.5)	92.1 (90.4,93.8)	38.8 (35.8,41.9)	91.1 (89.3,92.9)
**H**	**H**	**H**	**H**	15.7 (13.4,18.0)	0.0 (0.0,0.0)	0.0 (0.0,0.0)	94.3 (92.9,95.7)	95.7 (94.4,97.0)	93.9 (92.4,95.4)	93.1 (91.5,94.7)	35.7 (32.7,38.7)	34.0 (31.1,36.9)

**L**	**L**	**L**	**L**	96.4 (95.0,97.8)	79.7 (76.7,82.8)	81.8 (78.9,84.7)	88.2 (85.8,90.7)	89.6 (87.3,91.9)	59.9 (56.2,63.6)	56.2 (52.4,59.9)	96.6 (95.2,97.9)	95.5 (94.0,97.1)
**L**	**L**	**H**	**L**	87.2 (85.1,89.3)	0.0 (0.0,0.0)	0.0 (0.0,0.0)	84.7 (82.4,86.9)	95.4 (94.1,96.7)	72.5 (69.7,75.3)	71.0 (68.1,73.8)	36.5 (33.5,39.6)	91.8 (90.1,93.5)
**L**	**L**	**H**	**H**	41.3 (38.2,44.4)	0.0 (0.0,0.0)	0.0 (0.0,0.0)	93.8 (82.3,95.3)	94.4 (93.0,95.8)	85.8 (83.6,88.0)	84.7 (82.5,86.9)	29.0 (26.2,31.8)	30.5 (27.6,33.4)
**L**	**H**	**L**	**H**	47.3 (44.3,50.4)	0.0 (0.0,0.0)	0.3 (0.0,0.6)	93.7 (92.2,95.2)	93.9 (92.4,95.4)	77.5 (74.9,80.1)	92.5 (90.9,94.1)	91.8 (90.1,93.5)	42.9 (39.9,46.0)
**Sensitivity: H H H L**												
**Specificity**				π	Se_1	Se_2	Se_3	Se_4	Sp_1	Sp_2	Sp_3	Sp_4
**H**	**H**	**L**	**L**	41.8 (38.7,44.9)	0.1 (0.0,0.3)	0.0 (0.0,0.0)	95.6 (94.3,96.6)	96.1 (94.9,97.3)	90.1 (88.2,92.0)	91.9 (90.2,93.6)	75.7 (73.0,78.4)	90.9 (89.1,92.7)
**H**	**H**	**H**	**L**	12.1 (10.1,14.1)	0.0 (0.0,0.0)	0.0 (0.0,0.0)	94.1 (92.6,95.6)	94.8 (93.4,96.2)	94.2 (92.8,95.6)	94.7 (93.3,96.1)	2.8 (1.8,3.8)	89.0 (87.1,90.9)
**H**	**H**	**H**	**H**	8.8 (7.0,10.6)	0.0 (0.0,0.0)	0.0 (0.0,0.0)	94.4 (93.0,95.8)	94.4 (93.0,95.8)	93.4 (91.9,94.9)	93.0 (91.4,94.6)	4.4 (3.1,5.7)	31.9 (29.0,34.8)
**L**	**L**	**L**	**L**	96.8 (95.7,97.9)	4.4 (3.1,5.7)	3.7 (2.5,4.9)	82.9 (80.5,85.2)	95.9 (94.7,97.2)	70.2 (67.3,73.1)	69.0 (66.1,71.9)	82.0 (79.6,84.4)	93.6 (92.0,95.1)
**L**	**L**	**H**	**L**	65.1 (62.1,68.1)	0.0 (0.0,0.0)	0.0 (0.0,0.0)	84.0 (81.7,86.3)	94.9 (93.6,96.7)	78.1 (75.6,80.7)	78.5 (76.0,81.1)	1.9 (1.1,2.8)	91.6 (88.9,93.3)
**L**	**L**	**H**	**H**	10.8 (8.9,12.7)	0.0 (0.0,0.0)	0.0 (0.0,0.0)	94.7 (93.3,96.1)	92.5 (90.9,94.1)	88.8 (86.8,90.8)	89.9 (88.0,91.8)	2.0 (1.1,2.9)	29.1 (26.3,31.9)
**L**	**H**	**L**	**H**	27.1 (24.3,29.9)	0.0 (0.0,0.0)	0.0 (0.0,0.0)	94.2 (92.8,95.6)	94.5 (93.1,95.9)	78.7 (76.2,81.2)	92.7 (91.1,94.3)	68.6 (65.7,71.5)	33.9 (31.0,36.8)
**Sensitivity: H H H H**												
**Specificity**				π	Se_1	Se_2	Se_3	Se_4	Sp_1	Sp_2	Sp_3	Sp_4
**H**	**H**	**L**	**L**	21.7 (19.1,24.3)	0.0 (0.0,0.0)	0.0 (0.0,0.0)	94.8 (93.4,96.2)	94.7 (93.3,96.1)	92.6 (91.0,94.2)	93.0 (91.4,94.6)	72.1 (69.3,74.9)	72.8 (70.0,75.6)
**H**	**H**	**H**	**L**	10.7 (8.8,12.6)	0.0 (0.0,0.0)	0.0 (0.0,0.0)	94.9 (93.5,96.3)	96.3 (95.1,97.5)	94.8 (93.4,96.2)	93.2 (91.6,94.8)	3.3 (2.2,4.4)	67.8 (64.9,70.7)
**H**	**H**	**H**	**H**	9.0 (7.2,10.8)	0.0 (0.0,0.0)	0.0 (0.0,0.0)	95.7 (94.4,97.0)	94.5 (93.1,95.9)	94.5 (93.1,95.9)	93.2 (91.6,94.8)	4.1 (2.9,5.3)	2.2 (1.3,3.1)
**L**	**L**	**L**	**L**	71.9 (69.1,74.7)	0.1 (0.0,0.3)	0.1 (0.0,0.3)	92.8 (91.2,94.5)	93.1 (91.5,94.6)	80.3 (77.8,82.7)	82.1 (79.7,84.5)	78.9 (76.3,81.4)	74.6 (71.9,77.3)
**L**	**L**	**H**	**L**	21.8 (19.3,24.4)	0.0 (0.0,0.0)	0.0 (0.0,0.0)	95.2 (93.9,96.5)	91.8 (90.1,93.5)	87.3 (85.2,89.4)	86.3 (84.2,88.4)	2.1 (1.2,3.0)	69.4 (66.5,72.2)
**L**	**L**	**H**	**H**	2.2 (1.3,3.1)	0.0 (0.0,0.0)	0.0 (0.0,0.0)	88.5 (86.5,90.5)	87.3 (85.2,89.4)	91.9 (90.2,93.6)	91.1 (89.3,92.9)	1.3 (0.6,2.0)	1.7 (0.9,2.5)
**L**	**H**	**L**	**H**	12.8 (10.7,14.9)	0.0 (0.0,0.0)	0.0 (0.0,0.0)	94.6 (93.2,96.0)	94.9 (93.5,96.3)	82.9 (80.6,85.2)	95.2 (93.9,96.5)	66.6 (63.7,69.5)	3.5 (2.4,4.6)
**Sensitivity: L L L L**												
**Specificity**				π	Se_1	Se_2	Se_3	Se_4	Sp_1	Sp_2	Sp_3	Sp_4
**H**	**H**	**L**	**L**	0.0 (0.0,0.0)	0.0 (0.0,0.0)	0.0 (0.0,0.0)	93.6 (92.0,95.2)	93.6 (92.0,95.2)	66.0 (62.9,69.1)	68.5 (65.5,71.6)	36.3 (33.2,39.4)	36.2 (33.1,39.3)
**H**	**H**	**H**	**L**	0.0 (0.0,0.0)	0.0 (0.0,0.0)	0.0 (0.0,0.0)	95.3 (94.0,96.6)	94.6 (93.2,96.0)	81.5 (79.1,83.9)	78.5 (76.0,81.0)	0.0 (0.0,0.0)	29.8 (27.0,32.6)
**H**	**H**	**H**	**H**	0.0 (0.0,0.0)	0.0 (0.0,0.0)	0.0 (0.0,0.0)	94.5 (93.1,95.9)	95.0 (93.6,96.3)	79.4 (76.9,81.9)	79.3 (76.8,81.8)	0.0 (0.0,0.0)	0.0 (0.0,0.0)
**L**	**L**	**L**	**L**	71.8 (68.3,75.4)	0.0 (0.0,0.0)	0.0 (0.0,0.0)	55.0 (51.1,59.0)	55.0 (51.1,59.0)	0.0 (0.0,0.0)	0.2 (0.0,0.5)	35.5 (31.7,39.3)	35.2 (31.4,39.0)
**L**	**L**	**H**	**L**	0.0 (0.0,0.0)	0.0 (0.0,0.0)	0.0 (0.0,0.0)	9.6 (7.7,11.4)	79.3 (76.7,81.8)	0.2 (0.0,0.5)	0.1 (0.0,0.3)	0.0 (0.0,0.0)	28.1 (25.3,30.9)
**L**	**L**	**H**	**H**	0.0 (0.0,0.0)	0.0 (0.0,0.0)	0.0 (0.0,0.0)	80.0 (77.5,82.5)	80.0 (77.5,82.5)	4.7 (3.4,6.0)	4.1 (2.9,5.4)	0.0 (0.0,0.0)	0.0 (0.0,0.0)
**L**	**H**	**L**	**H**	0.0 (0.0,0.0)	0.0 (0.0,0.0)	0.0 (0.0,0.0)	93.0 (91.4,94.6)	85.5 (83.3,87.7)	0.5 (0.1,0.9)	72.4 (69.6,75.1)	27.4 (24.7,30.2)	0.0 (0.0,0.0)
**Sensitivity: L L H L**												
**Specificity**				π	Se_1	Se_2	Se_3	Se_4	Sp_1	Sp_2	Sp_3	Sp_4
**H**	**H**	**L**	**L**	0.0 (0.0,0.0)	0.0 (0.0,0.0)	0.0 (0.0,0.0)	92.8 (91.2,94.4)	95.0 (93.6,96.4)	75.2 (72.5,77.9)	76.2 (73.6,78.8)	0.0 (0.0,0.0)	29.9 (27.1,32.7)
**H**	**H**	**H**	**L**	0.0 (0.0,0.0)	0.0 (0.0,0.0)	0.0 (0.0,0.0)	92.9 (91.3,94.5)	95.9 (94.7,97.1)	85.3 (83.1,87.5)	84.5 (82.3,86.7)	0.0 (0.0,0.0)	28.8 (26.0,31.6)
**H**	**H**	**H**	**H**	0.0 (0.0,0.0)	0.0 (0.0,0.0)	0.0 (0.0,0.0)	92.8 (91.2,94.4)	95.2 (93.9,96.5)	82.7 (80.4,85.0)	78.9 (76.4,81.4)	0.0 (0.0,0.0)	0.0 (0.0,0.0)
**L**	**L**	**L**	**L**	0.1 (0.0,0.3)	0.0 (0.0,0.0)	0.0 (0.0,0.0)	8.9 (7.1,10.7)	77.4 (74.8,80.0)	0.2 (0.0,0.5)	0.3 (0.0,0.6)	0.0 (0.0,0.0)	32.7 (29.8,35.7)
**L**	**L**	**H**	**L**	0.0 (0.0,0.0)	0.0 (0.0,0.0)	0.0 (0.0,0.0)	6.1 (4.7,7.6)	94.1 (92.6,95.5)	0.2 (0.0,0.5)	0.7 (0.2,1.2)	0.0 (0.0,0.0)	39.9 (36.9,43.0)
**L**	**L**	**H**	**H**	2.1 (1.2,3.0)	2.0 (1.1,2.9)	1.6 (0.8,2.5)	90.7 (88.8,92.6)	70.0 (67.1,73.0)	19.9 (17.3,22.5)	17.9 (15.5,20.4)	2.1 (1.2,3.0)	2.2 (1.2,3.1)
**L**	**H**	**L**	**H**	0.0 (0.0,0.0)	0.0 (0.0,0.0)	0.0 (0.0,0.0)	94.2 (92.8,95.6)	92.0 (90.3,93.7)	0.8 (0.2,1.4)	80.8 (78.4,83.2)	0.0 (0.0,0.0)	0.0 (0.0,0.0)
**Sensitivity: L L H H**												

Specificity				π	Se_1	Se_2	Se_3	Se_4	Sp_1	Sp_2	Sp_3	Sp_4
**H**	**H**	**L**	**L**	0.0 (0.0,0.0)	0.0 (0.0,0.0)	0.0 (0.0,0.0)	94.3 (92.9,95.7)	94.3 (92.9,95.7)	78.5 (76.0,81.0)	76.1 (73.5,78.7)	0.0 (0.0,0.0)	0.0 (0.0,0.0)
**H**	**H**	**H**	**L**	0.0 (0.0,0.0)	0.0 (0.0,0.0)	0.0 (0.0,0.0)	94.6 (93.2,96.0)	95.3 (94.0,96.6)	82.9 (80.6,85.2)	82.9 (80.6,85.2)	0.0 (0.0,0.0)	0.0 (0.0,0.0)
**H**	**H**	**H**	**H**	0.0 (0.0,0.0)	0.0 (0.0,0.0)	0.0 (0.0,0.0)	95.2 (93.9,96.5)	96.4 (95.2,97.6)	74.8 (72.2,77.5)	76.0 (73.3,78.6)	0.0 (0.0,0.0)	0.0 (0.0,0.0)
**L**	**L**	**L**	**L**	0.0 (0.0,0.0)	0.0 (0.0,0.0)	0.0 (0.0,0.0)	73.3 (70.5,76.0)	75.4 (72.7,78.1)	0.8 (0.2,1.4)	1.4 (0.7,2.1)	0.0 (0.0,0.0)	0.0 (0.0,0.0)
**L**	**L**	**H**	**L**	0.0 (0.0,0.0)	0.0 (0.0,0.0)	0.0 (0.0,0.0)	92.8 (91.2,94.4)	59.9 (56.9,63.0)	9.5 (7.7,11.3)	9.5 (7.7,11.3)	0.0 (0.0,0.0)	0.0 (0.0,0.0)
**L**	**L**	**H**	**H**	95.2 (93.6,96.8)	79.8 (76.8,82.8)	78.2 (75.1,81.3)	96.8 (95.5,98.1)	95.0 (93.4,96.7)	90.1 (87.8,92.3)	93.4 (91.6,95.3)	93.7 (91.9,95.5)	93.1 (91.2,95.0)
**L**	**H**	**L**	**H**	0.0 (0.0,0.0)	0.0 (0.0,0.0)	0.0 (0.0,0.0)	93.1 (91.5,94.7)	92.5 (90.9,94.1)	2.2 (1.3,3.1)	89.9 (88.0,91.8)	0.0 (0.0,0.0)	0.0 (0.0,0.0)

viii. Setting 8: $\pi = 0.2, \omega = 0.5, n_{obs} = 5000$

Table viii.1 Number of converged data sets for different sensitivity-specificity combinations

				**Number of converged data sets**
**Specificity**				**Sensitivity: H H L L**
**H**	**H**	**L**	**L**	866
**H**	**H**	**H**	**L**	1000
**H**	**H**	**H**	**H**	1000
**L**	**L**	**L**	**L**	526
**L**	**L**	**H**	**L**	957
**L**	**L**	**H**	**H**	995
**L**	**H**	**L**	**H**	995
**Specificity**				**Sensitivity: H H H L**
**H**	**H**	**L**	**L**	997
**H**	**H**	**H**	**L**	1000
**H**	**H**	**H**	**H**	1000
**L**	**L**	**L**	**L**	883
**L**	**L**	**H**	**L**	967
**L**	**L**	**H**	**H**	1000
**L**	**H**	**L**	**H**	1000
**Specificity**				**Sensitivity: H H H H**
**H**	**H**	**L**	**L**	1000
**H**	**H**	**H**	**L**	1000
**H**	**H**	**H**	**H**	1000
**L**	**L**	**L**	**L**	972
**L**	**L**	**H**	**L**	999
**L**	**L**	**H**	**H**	1000
**L**	**H**	**L**	**H**	1000

Specificity				Sensitivity: L L L L
**H**	**H**	**L**	**L**	633
**H**	**H**	**H**	**L**	989
**H**	**H**	**H**	**H**	994
**L**	**L**	**L**	**L**	280
**L**	**L**	**H**	**L**	901
**L**	**L**	**H**	**H**	670
**L**	**H**	**L**	**H**	939
**Specificity**				**Sensitivity: L L H L**
**H**	**H**	**L**	**L**	971
**H**	**H**	**H**	**L**	1000
**H**	**H**	**H**	**H**	998
**L**	**L**	**L**	**L**	846
**L**	**L**	**H**	**L**	962
**L**	**L**	**H**	**H**	794
**L**	**H**	**L**	**H**	949
**Specificity**				**Sensitivity: L L H H**
**H**	**H**	**L**	**L**	1000
**H**	**H**	**H**	**L**	1000
**H**	**H**	**H**	**H**	999
**L**	**L**	**L**	**L**	890
**L**	**L**	**H**	**L**	699
**L**	**L**	**H**	**H**	840
**L**	**H**	**L**	**H**	938

Table viii.2 Percentages of the time 95% credible intervals (CrIs) for residual correlations did not included '0' for any pairs, and percentages of the time that the overall χ^2 or G^2 statistic indicated a lack of overall fit

Sensitivity: H H L L						
**Specificity**				**Res. Cor.**	**Overall G^2**	**Overall χ^2**
**H**	**H**	**L**	**L**	62.6 (59.3,65.8)	6.5 (4.9,8.3)	6.8 (5.2,8.7)
**H**	**H**	**H**	**L**	27.1 (24.4,30.0)	6.9 (5.4,8.7)	6.2 (4.8,7.9)
**H**	**H**	**H**	**H**	37.8 (34.8,40.9)	38.0 (35.0,41.1)	42.0 (38.9,45.1)
**L**	**L**	**L**	**L**	53.6 (49.2,57.9)	11.6 (9.0,14.6)	11.8 (9.2,14.9)
**L**	**L**	**H**	**L**	33.6 (30.7,36.7)	15.7 (13.4,18.1)	16.0 (13.7,18.5)
**L**	**L**	**H**	**H**	32.1 (29.2,35.1)	78.3 (75.6,80.8)	82.2 (79.7,84.5)
**L**	**H**	**L**	**H**	39.1 (36.0,42.2)	10.4 (8.5,12.4)	10.6 (8.7,12.6)
**Sensitivity: H H H L**						
**Specificity**				**Res. Cor.**	**Overall G^2**	**Overall χ^2**
**H**	**H**	**L**	**L**	35.5 (32.5,38.6)	5.1 (3.8,6.7)	4.9 (3.7,6.4)

H	H	H	L	10.9 (9.0,13.0)	7.9 (6.3,9.7)	7.7 (6.1,9.5)
H	H	H	H	46.0 (42.9,49.1)	73.2 (70.3,75.9)	77.3 (74.6,79.9)
L	L	L	L	39.4 (36.2,42.7)	11.9 (9.8,14.2)	11.8 (9.7,14.1)
L	L	H	L	43.1 (40.0,46.3)	23.0 (20.3,25.7)	23.6 (20.9,26.4)
L	L	H	H	59.5 (56.4,62.6)	97.3 (96.1,98.2)	98.5 (97.5,99.2)
L	H	L	H	51.1 (48.0,54.2)	29.5 (26.7,32.4)	29.8 (27.0,32.7)
**Sensitivity: H H H H**						
**Specificity**				**Res. Cor.**	**Overall G^2**	**Overall χ^2**
H	H	L	L	44.9 (41.8,48.0)	10.2 (8.4,12.2)	10.3 (8.5,12.4)
H	H	H	L	61.3 (58.2,64.3)	41.1 (38.0,44.2)	40.3 (37.2,43.4)
H	H	H	H	93.5 (91.8,94.9)	99.0 (98.2,99.5)	99.5 (98.8,99.8)
L	L	L	L	48.5 (45.3,51.7)	25.7 (23.0,28.6)	26.5 (23.8,29.4)
L	L	H	L	82.9 (80.4,85.2)	86.4 (84.1,88.5)	87.8 (85.6,89.8)
L	L	H	H	94.5 (92.9,95.8)	100.0 (99.6,100.0)	100.0 (99.6,100.0)
L	H	L	H	74.6 (71.8,77.3)	63.2 (60.1,66.2)	64.1 (61.0,67.1)
**Sensitivity: L L L L**						
**Specificity**				**Res. Cor.**	**Overall G^2**	**Overall χ^2**
H	H	L	L	70.8 (67.1,74.3)	8.5 (6.5,11.0)	8.5 (6.5,11.0)
H	H	H	L	87.0 (84.7,89.0)	40.7 (37.7,43.9)	40.5 (37.5,43.7)
H	H	H	H	100.0 (99.6,100.0)	100.0 (99.6,100.0)	100.0 (99.6,100.0)
L	L	L	L	83.9 (79.1,88.0)	45.0 (39.1,51.0)	45.7 (39.8,51.7)
L	L	H	L	99.9 (99.4,100.0)	97.8 (96.6,98.6)	98.0 (96.9,98.8)
L	L	H	H	100.0 (99.5,100.0)	100.0 (99.5,100.0)	100.0 (99.5,100.0)
L	H	L	H	98.8 (97.9,99.4)	84.3 (81.9,86.6)	84.7 (82.2,86.9)
**Sensitivity: L L H L**						
**Specificity**				**Res. Cor.**	**Overall G^2**	**Overall χ^2**
H	H	L	L	82.9 (80.4,85.2)	22.3 (19.8,25.1)	21.7 (19.2,24.5)
H	H	H	L	96.7 (95.4,97.7)	81.4 (78.8,83.8)	81.6 (79.1,84.0)
H	H	H	H	100.0 (99.6,100.0)	100.0 (99.6,100.0)	100.0 (99.6,100.0)
L	L	L	L	99.3 (98.5,99.7)	87.2 (84.8,89.4)	87.5 (85.0,89.6)
L	L	H	L	100.0 (99.6,100.0)	100.0 (99.6,100.0)	100.0 (99.6,100.0)
L	L	H	H	100.0 (99.5,100.0)	100.0 (99.5,100.0)	100.0 (99.5,100.0)
L	H	L	H	100.0 (99.6,100.0)	100.0 (99.6,100.0)	100.0 (99.6,100.0)
**Sensitivity: L L H H**						
**Specificity**				**Res. Cor.**	**Overall G^2**	**Overall χ^2**
H	H	L	L	99.9 (99.4,100.0)	92.0 (90.1,93.6)	92.0 (90.1,93.6)
H	H	H	L	100.0 (99.6,100.0)	100.0 (99.6,100.0)	100.0 (99.6,100.0)
H	H	H	H	100.0 (99.6,100.0)	100.0 (99.6,100.0)	100.0 (99.6,100.0)
L	L	L	L	100.0 (99.6,100.0)	100.0 (99.6,100.0)	100.0 (99.6,100.0)
L	L	H	L	100.0 (99.5,100.0)	100.0 (99.5,100.0)	100.0 (99.5,100.0)
L	L	H	H	100.0 (99.6,100.0)	100.0 (99.6,100.0)	100.0 (99.6,100.0)

**L**	**H**	**L**	**H**	100.0 (99.6,100.0)	100.0 (99.6,100.0)	100.0 (99.6,100.0)

Table viii.3 Percentages of the time 95% CrIs for residual correlations did not included '0' for each pair, and percentages of the time the pairwise χ^2 or G^2 statistic for each pair indicated a lack of pairwise fit

**Sensitivity: H H L L**										
**Specificity**				**Tool**	**T_1&T_2**	**T_1&T_3**	**T_1&T_4**	**T_2&T_3**	**T_2&T_4**	**T_3&T_4**
**H**	**H**	**L**	**L**	**Res. Cor.**	0.0 (0.0,0.4)	0.1 (0.0,0.6)	0.0 (0.0,0.4)	0.0 (0.0,0.4)	0.3 (0.1,1.0)	62.4 (59.0,65.6)
				**Pairwise G^2**	0.0 (0.0,0.4)	0.1 (0.0,0.6)	0.0 (0.0,0.4)	0.0 (0.0,0.4)	0.0 (0.0,0.4)	5.0 (3.6,6.6)
				**Pairwise χ^2**	0.0 (0.0,0.4)	0.1 (0.0,0.6)	0.0 (0.0,0.4)	0.0 (0.0,0.4)	0.0 (0.0,0.4)	5.0 (3.6,6.6)
**H**	**H**	**H**	**L**	**Res. Cor.**	0.0 (0.0,0.4)	0.0 (0.0,0.4)	0.2 (0.0,0.7)	0.0 (0.0,0.4)	0.1 (0.0,0.6)	26.9 (24.2,29.8)
				**Pairwise G^2**	0.0 (0.0,0.4)	0.0 (0.0,0.4)	0.0 (0.0,0.4)	0.0 (0.0,0.4)	0.0 (0.0,0.4)	4.5 (3.3,6.0)
				**Pairwise χ^2**	0.0 (0.0,0.4)	0.0 (0.0,0.4)	0.0 (0.0,0.4)	0.0 (0.0,0.4)	0.0 (0.0,0.4)	4.5 (3.3,6.0)
**H**	**H**	**H**	**H**	**Res. Cor.**	0.0 (0.0,0.4)	0.0 (0.0,0.4)	0.0 (0.0,0.4)	0.0 (0.0,0.4)	0.0 (0.0,0.4)	37.8 (34.8,40.9)
				**Pairwise G^2**	0.0 (0.0,0.4)	0.0 (0.0,0.4)	0.0 (0.0,0.4)	0.0 (0.0,0.4)	0.0 (0.0,0.4)	15.7 (13.5,18.1)
				**Pairwise χ^2**	0.0 (0.0,0.4)	0.0 (0.0,0.4)	0.0 (0.0,0.4)	0.0 (0.0,0.4)	0.0 (0.0,0.4)	15.9 (13.7,18.3)
**L**	**L**	**L**	**L**	**Res. Cor.**	0.0 (0.0,0.7)	3.8 (2.3,5.8)	4.6 (2.9,6.7)	4.2 (2.6,6.3)	3.0 (1.7,4.9)	44.5 (40.2,48.8)
				**Pairwise G^2**	0.0 (0.0,0.7)	0.0 (0.0,0.7)	0.6 (0.1,1.7)	0.6 (0.1,1.7)	0.6 (0.1,1.7)	3.4 (2.0,5.4)
				**Pairwise χ^2**	0.0 (0.0,0.7)	0.0 (0.0,0.7)	0.6 (0.1,1.7)	0.6 (0.1,1.7)	0.6 (0.1,1.7)	3.4 (2.0,5.4)
**L**	**L**	**H**	**L**	**Res. Cor.**	0.0 (0.0,0.4)	0.0 (0.0,0.4)	11.2 (9.3,13.4)	0.0 (0.0,0.4)	12.4 (10.4,14.7)	17.1 (14.8,19.7)
				**Pairwise G^2**	0.0 (0.0,0.4)	0.0 (0.0,0.4)	0.7 (0.3,1.5)	0.0 (0.0,0.4)	1.7 (1.0,2.7)	3.7 (2.6,5.0)
				**Pairwise χ^2**	0.0 (0.0,0.4)	0.0 (0.0,0.4)	0.7 (0.3,1.5)	0.0 (0.0,0.4)	1.7 (1.0,2.7)	3.7 (2.6,5.0)
**L**	**L**	**H**	**H**	**Res. Cor.**	6.3 (4.9,8.0)	2.0 (1.2,3.1)	1.4 (0.8,2.3)	2.3 (1.5,3.4)	1.8 (1.1,2.8)	28.6 (25.9,31.6)
				**Pairwise G^2**	1.7 (1.0,2.7)	0.5 (0.2,1.2)	0.4 (0.1,1.0)	0.5 (0.2,1.2)	0.1 (0.0,0.6)	22.7 (20.1,25.4)
				**Pairwise χ^2**	1.7 (1.0,2.7)	0.5 (0.2,1.2)	0.4 (0.1,1.0)	0.5 (0.2,1.2)	0.1 (0.0,0.6)	22.8 (20.2,25.6)
**L**	**H**	**L**	**H**	**Res. Cor.**	0.0 (0.0,0.4)	21.4 (18.9,24.1)	2.8 (1.9,4.0)	0.0 (0.0,0.4)	0.0 (0.0,0.4)	21.2 (18.7,23.9)
				**Pairwise G^2**	0.0 (0.0,0.4)	2.1 (1.3,3.2)	0.3 (0.1,0.9)	0.0 (0.0,0.4)	0.0 (0.0,0.4)	3.6 (2.5,5.0)
				**Pairwise χ^2**	0.0 (0.0,0.4)	2.1 (1.3,3.2)	0.3 (0.1,0.9)	0.0 (0.0,0.4)	0.0 (0.0,0.4)	3.6 (2.5,5.0)
**Sensitivity: H H H L**										
**Specificity**				**Tool**	**T_1&T_2**	**T_1&T_3**	**T_1&T_4**	**T_2&T_3**	**T_2&T_4**	**T_3&T_4**
**H**	**H**	**L**	**L**	**Res. Cor.**	0.0 (0.0,0.4)	0.0 (0.0,0.4)	0.2 (0.0,0.7)	0.0 (0.0,0.4)	0.2 (0.0,0.7)	35.2 (32.2,38.3)
				**Pairwise G^2**	0.0 (0.0,0.4)	0.0 (0.0,0.4)	0.0 (0.0,0.4)	0.0 (0.0,0.4)	0.0 (0.0,0.4)	3.1 (2.1,4.4)
				**Pairwise χ^2**	0.0 (0.0,0.4)	0.0 (0.0,0.4)	0.0 (0.0,0.4)	0.0 (0.0,0.4)	0.0 (0.0,0.4)	3.1 (2.1,4.4)
**H**	**H**	**H**	**L**	**Res. Cor.**	0.0 (0.0,0.4)	0.0 (0.0,0.4)	0.7 (0.3,1.4)	0.0 (0.0,0.4)	0.6 (0.2,1.3)	9.7 (7.9,11.7)
				**Pairwise G^2**	0.0 (0.0,0.4)	0.0 (0.0,0.4)	0.0 (0.0,0.4)	0.0 (0.0,0.4)	0.0 (0.0,0.4)	2.3 (1.5,3.4)
				**Pairwise χ^2**	0.0 (0.0,0.4)	0.0 (0.0,0.4)	0.0 (0.0,0.4)	0.0 (0.0,0.4)	0.0 (0.0,0.4)	2.3 (1.5,3.4)
**H**	**H**	**H**	**H**	**Res. Cor.**	0.0 (0.0,0.4)	0.0 (0.0,0.4)	0.0 (0.0,0.4)	0.0 (0.0,0.4)	0.0 (0.0,0.4)	46.0 (42.9,49.1)
				**Pairwise G^2**	0.0 (0.0,0.4)	0.0 (0.0,0.4)	0.0 (0.0,0.4)	0.0 (0.0,0.4)	0.0 (0.0,0.4)	31.6 (28.7,34.6)
				**Pairwise χ^2**	0.0 (0.0,0.4)	0.0 (0.0,0.4)	0.0 (0.0,0.4)	0.0 (0.0,0.4)	0.0 (0.0,0.4)	31.2 (28.3,34.2)

**L**	**L**	**L**	**L**	**Res. Cor.**	0.0 (0.0,0.4)	2.3 (1.4,3.5)	10.6 (8.7,12.9)	2.3 (1.4,3.5)	10.8 (8.8,13.0)	24.6 (21.8,27.6)
				**Pairwise G^2**	0.0 (0.0,0.4)	0.8 (0.3,1.6)	1.0 (0.5,1.9)	0.3 (0.1,1.0)	0.9 (0.4,1.8)	4.4 (3.2,6.0)
				**Pairwise χ^2**	0.0 (0.0,0.4)	0.8 (0.3,1.6)	1.0 (0.5,1.9)	0.3 (0.1,1.0)	0.9 (0.4,1.8)	4.4 (3.2,6.0)
**L**	**L**	**H**	**L**	**Res. Cor.**	0.0 (0.0,0.4)	0.0 (0.0,0.4)	20.5 (18.0,23.2)	0.0 (0.0,0.4)	19.4 (17.0,22.1)	12.6 (10.6,14.9)
				**Pairwise G^2**	0.0 (0.0,0.4)	0.0 (0.0,0.4)	2.2 (1.3,3.3)	0.0 (0.0,0.4)	1.2 (0.6,2.2)	4.3 (3.1,5.8)
				**Pairwise χ^2**	0.0 (0.0,0.4)	0.0 (0.0,0.4)	2.2 (1.3,3.3)	0.0 (0.0,0.4)	1.2 (0.6,2.2)	4.3 (3.1,5.8)
**L**	**L**	**H**	**H**	**Res. Cor.**	26.4 (23.7,29.2)	1.3 (0.7,2.2)	5.6 (4.3,7.2)	1.7 (1.0,2.7)	5.0 (5.7,6.5)	47.6 (44.5,50.7)
				**Pairwise G^2**	6.6 (5.1,8.3)	0.2 (0.0,0.7)	1.1 (0.6,2.0)	0.6 (0.2,1.3)	0.7 (0.3,1.4)	43.2 (40.1,46.3)
				**Pairwise χ^2**	6.5 (5.1,8.2)	0.2 (0.0,0.7)	1.2 (0.6,2.1)	0.7 (0.3,1.4)	0.8 (0.3,1.6)	42.6 (39.5,45.7)
**L**	**H**	**L**	**H**	**Res. Cor.**	0.0 (0.0,0.4)	18.0 (15.7,20.5)	4.4 (3.2,5.9)	0.0 (0.0,0.4)	0.0 (0.0,0.4)	40.9 (37.8,44.0)
				**Pairwise G^2**	0.0 (0.0,0.4)	2.7 (1.8,3.9)	0.6 (0.2,1.3)	0.0 (0.0,0.4)	0.0 (0.0,0.4)	18.3 (15.9,20.8)
				**Pairwise χ^2**	0.0 (0.0,0.4)	2.7 (1.8,3.9)	0.6 (0.2,1.3)	0.0 (0.0,0.4)	0.0 (0.0,0.4)	18.0 (15.7,20.5)
**Sensitivity: H H H H**										
**Specificity**				**Tool**	**T_1&T_2**	**T_1&T_3**	**T_1&T_4**	**T_2&T_3**	**T_2&T_4**	**T_3&T_4**
**H**	**H**	**L**	**L**	**Res. Cor.**	0.0 (0.0,0.4)	0.1 (0.0,0.6)	0.2 (0.0,0.7)	0.0 (0.0,0.4)	0.0 (0.0,0.4)	44.8 (41.7,47.9)
				**Pairwise G^2**	0.0 (0.0,0.4)	0.0 (0.0,0.4)	0.0 (0.0,0.4)	0.0 (0.0,0.4)	0.0 (0.0,0.4)	11.0 (9.1.13.1)
				**Pairwise χ^2**	0.0 (0.0,0.4)	0.0 (0.0,0.4)	0.0 (0.0,0.4)	0.0 (0.0,0.4)	0.0 (0.0,0.4)	11.0 (9.1.13.1)
**H**	**H**	**H**	**L**	**Res. Cor.**	0.0 (0.0,0.4)	0.0 (0.0,0.4)	1.1 (0.6,2.0)	0.0 (0.0,0.4)	0.7 (0.3,1.4)	60.7 (57.6,63.7)
				**Pairwise G^2**	0.0 (0.0,0.4)	0.0 (0.0,0.4)	0.4 (0.1,1.0)	0.0 (0.0,0.4)	0.2 (0.0,0.7)	34.9 (31.9,37.9)
				**Pairwise χ^2**	0.0 (0.0,0.4)	0.0 (0.0,0.4)	0.4 (0.1,1.0)	0.0 (0.0,0.4)	0.2 (0.0,0.7)	34.4 (31.5,37.4)
**H**	**H**	**H**	**H**	**Res. Cor.**	0.0 (0.0,0.4)	0.0 (0.0,0.4)	0.0 (0.0,0.4)	0.0 (0.0,0.4)	0.0 (0.0,0.4)	93.5 (91.8,94.9)
				**Pairwise G^2**	0.0 (0.0,0.4)	0.0 (0.0,0.4)	0.0 (0.0,0.4)	0.0 (0.0,0.4)	0.0 (0.0,0.4)	87.2 (85.0,89.2)
				**Pairwise χ^2**	0.0 (0.0,0.4)	0.0 (0.0,0.4)	0.0 (0.0,0.4)	0.0 (0.0,0.4)	0.0 (0.0,0.4)	86.9 (84.6,88.9)
**L**	**L**	**L**	**L**	**Res. Cor.**	2.7 (1.8,3.9)	5.1 (3.8,6.7)	5.7 (4.3,7.3)	6.7 (5.2,8.4)	7.1 (5.6,8.9)	37.7 (34.6,40.8)
				**Pairwise G^2**	0.6 (0.2,1.3)	0.4 (0.1,1.1)	0.7 (0.3,1.5)	0.6 (0.2,1.3)	0.8 (0.4,1.6)	13.7 (11.6,16.0)
				**Pairwise χ^2**	0.6 (0.2,1.3)	0.4 (0.1,1.1)	0.7 (0.3,1.5)	0.6 (0.2,1.3)	0.8 (0.4,1.6)	13.6 (11.5,15.9)
**L**	**L**	**H**	**L**	**Res. Cor.**	14.5 (12.4,16.9)	0.3 (0.1,0.9)	14.0 (11.9,16.3)	0.4 (0.1,1.0)	14.7 (12.6,17.1)	77.7 (75.0,80.2)
				**Pairwise G^2**	3.5 (2.5,4.8)	0.0 (0.0,0.4)	1.4 (0.8,2.3)	0.0 (0.0,0.4)	2.6 (1.7,3.8)	62.5 (59.4,65.5)
				**Pairwise χ^2**	3.5 (2.5,4.8)	0.0 (0.0,0.4)	1.5 (0.8,2.5)	0.0 (0.0,0.4)	2.7 (1.8,3.9)	61.5 (58.4,64.5)
**L**	**L**	**H**	**H**	**Res. Cor.**	46.0 (42.9,49.1)	2.6 (1.7,3.8)	3.5 (2.4,4.8)	2.3 (1.5,3.4)	3.7 (2.6,5.1)	90.9 (88.9,92.6)
				**Pairwise G^2**	14.1 (12.0,16.4)	0.8 (0.3,1.6)	1.5 (0.8,2.5)	0.8 (0.3,1.6)	0.8 (0.3,1.6)	89.3 (87.2,91.1)
				**Pairwise χ^2**	13.9 (11.8,16.2)	1.1 (0.6,2.0)	1.7 (1.0,2.7)	0.9 (0.4,1.7)	1.0 (0.5,1.8)	89.1 (87.0,91.0)
**L**	**H**	**L**	**H**	**Res. Cor.**	0.1 (0.0,0.6)	23.7 (21.1,26.5)	4.4 (3.2,5.9)	0.4 (0.1,1.0)	0.0 (0.0,0.4)	67.2 (64.2,70.1)
				**Pairwise G^2**	0.0 (0.0,0.4)	2.7 (1.8,3.9)	0.9 (0.4,1.7)	0.0 (0.0,0.4)	0.0 (0.0,0.4)	47.1 (44.0,50.2)
				**Pairwise χ^2**	0.0 (0.0,0.4)	2.7 (1.8,3.9)	1.0 (0.5,1.8)	0.0 (0.0,0.4)	0.0 (0.0,0.4)	46.3 (43.2,49.4)
**Sensitivity: L L L L**										
**Specificity**				**Tool**	**T_1&T_2**	**T_1&T_3**	**T_1&T_4**	**T_2&T_3**	**T_2&T_4**	**T_3&T_4**
**H**	**H**	**L**	**L**	**Res. Cor.**	0.0 (0.0,0.6)	0.3 (0.0,1.1)	0.2 (0.0,0.9)	0.2 (0.0,0.9)	0.2 (0.0,0.9)	70.8 (67.1,74.3)
				**Pairwise G^2**	0.0 (0.0,0.6)	0.0 (0.0,0.6)	0.0 (0.0,0.6)	0.0 (0.0,0.6)	0.0 (0.0,0.6)	13.3 (10.7,16.2)
				**Pairwise χ^2**	0.0 (0.0,0.6)	0.0 (0.0,0.6)	0.0 (0.0,0.6)	0.0 (0.0,0.6)	0.0 (0.0,0.6)	13.3 (10.7,16.2)
**H**	**H**	**H**	**L**	**Res. Cor.**	0.0 (0.0,0.4)	0.0 (0.0,0.4)	1.9 (1.2,3.0)	0.0 (0.0,0.4)	1.5 (0.9,2.5)	86.9 (84.6,88.9)

				Pairwise G^2	0.0 (0.0,0.4)	0.0 (0.0,0.4)	0.2 (0.0,0.7)	0.0 (0.0,0.4)	0.0 (0.0,0.4)	51.1 (47.9,54.2)
				Pairwise χ^2	0.0 (0.0,0.4)	0.0 (0.0,0.4)	0.2 (0.0,0.7)	0.0 (0.0,0.4)	0.0 (0.0,0.4)	51.3 (48.1,54.4)
H	H	H	H	Res. Cor.	0.1 (0.0,0.6)	0.4 (0.1,1.0)	0.2 (0.0,0.7)	0.1 (0.1,0.6)	0.5 (0.2,1.2)	100.0 (99.6,100.0)
				Pairwise G^2	0.4 (0.1,1.0)	0.0 (0.0,0.4)	0.1 (0.0,0.6)	0.0 (0.0,0.4)	0.0 (0.0,0.4)	100.0 (99.6,100.0)
				Pairwise χ^2	0.4 (0.1,1.0)	0.0 (0.0,0.4)	0.1 (0.0,0.6)	0.0 (0.0,0.4)	0.0 (0.0,0.4)	100.0 (99.6,100.0)
L	L	L	L	Res. Cor.	0.0 (0.0,1.3)	11.4 (7.9,15.7)	17.5 (13.2,22.5)	13.2 (9.5,17.8)	16.1 (12.0,20.9)	72.9 (67.2,78.0)
				Pairwise G^2	0.0 (0.0,1.3)	2.1 (0.8,4.6)	3.2 (1.5,6.0)	1.4 (0.4,3.6)	1.4 (0.4,3.6)	22.5 (17.7,27.8)
				Pairwise χ^2	0.0 (0.0,1.3)	2.1 (0.8,4.6)	3.2 (1.5,6.0)	1.4 (0.4,3.6)	1.4 (0.4,3.6)	22.9 (18.1,28.2)
L	L	H	L	Res. Cor.	0.2 (0.0,0.8)	32.1 (29.0,35.2)	36.2 (33.0,39.4)	31.7 (28.7,34.9)	36.8 (33.7,40.1)	99.7 (99.0,99.9)
				Pairwise G^2	0.2 (0.0,0.8)	9.2 (7.4,11.3)	8.7 (6.9,10.7)	11.8 (9.7,14.0)	8.2 (6.5,10.2)	95.4 (93.9,96.7)
				Pairwise χ^2	0.2 (0.0,0.8)	9.4 (7.6,11.5)	8.7 (6.9,10.7)	11.8 (9.7,14.0)	8.2 (6.5,10.2)	95.7 (94.1,96.9)
L	L	H	H	Res. Cor.	99.7 (98.9,100.0)	41.2 (37.4,45.0)	43.6 (39.8,47.4)	42.2 (38.5,46.1)	41.3 (37.6,45.2)	24.9 (21.7,28.4)
				Pairwise G^2	99.4 (98.5,99.8)	20.9 (17.9,24.2)	21.5 (18.4,24.8)	21.9 (18.9,25.3)	22.2 (19.1,25.6)	41.5 (37.7,45.3)
				Pairwise χ^2	99.4 (98.5,99.8)	21.3 (18.3,24.6)	21.9 (18.9,25.3)	22.4 (19.3,25.7)	22.5 (19.4,25.9)	41.8 (38.0,45.6)
L	H	L	H	Res. Cor.	0.0 (0.0,0.4)	49.1 (45.9,52.3)	66.3 (63.2,69.4)	0.1 (0.0,0.6)	0.0 (0.0,0.4)	95.5 (94.0,96.8)
				Pairwise G^2	0.0 (0.0,0.4)	6.7 (5.2,8.5)	29.1 (26.2,32.1)	0.0 (0.0,0.4)	0.0 (0.0,0.4)	75.6 (72.7,78.3)
				Pairwise χ^2	0.0 (0.0,0.4)	6.8 (5.3,8.6)	29.3 (26.4,32.3)	0.0 (0.0,0.4)	0.0 (0.0,0.4)	75.6 (72.7,78.3)
**Sensitivity: L L H L**										
**Specificity**				**Tool**	**T_1&T_2**	**T_1&T_3**	**T_1&T_4**	**T_2&T_3**	**T_2&T_4**	**T_3&T_4**
H	H	L	L	Res. Cor.	0.0 (0.0,0.4)	0.0 (0.0,0.4)	1.5 (0.9,2.5)	0.0 (0.0,0.4)	1.3 (0.7,2.3)	82.5 (80.0,84.8)
				Pairwise G^2	0.0 (0.0,0.4)	0.0 (0.0,0.4)	0.0 (0.0,0.4)	0.0 (0.0,0.4)	0.0 (0.0,0.4)	36.5 (33.4,39.6)
				Pairwise χ^2	0.0 (0.0,0.4)	0.0 (0.0,0.4)	0.0 (0.0,0.4)	0.0 (0.0,0.4)	0.0 (0.0,0.4)	36.6 (33.5,39.7)
H	H	H	L	Res. Cor.	0.0 (0.0,0.4)	0.0 (0.0,0.4)	6.3 (4.9,8.0)	0.0 (0.0,0.4)	6.9 (5.4,8.7)	96.6 (95.3,97.6)
				Pairwise G^2	0.0 (0.0,0.4)	0.0 (0.0,0.4)	1.3 (0.7,2.2)	0.0 (0.0,0.4)	1.3 (0.7,2.2)	84.6 (82.2,86.8)
				Pairwise χ^2	0.0 (0.0,0.4)	0.0 (0.0,0.4)	1.3 (0.7,2.2)	0.0 (0.0,0.4)	1.4 (0.8,2.3)	84.8 (82.4,87.0)
H	H	H	H	Res. Cor.	75.9 (73.1,78.5)	0.0 (0.0,0.4)	29.6 (26.7,32.5)	0.0 (0.0,0.4)	30.1 (27.2,33.0)	100.0 (99.6,100.0)
				Pairwise G^2	82.2 (79.6,84.5)	0.0 (0.0,0.4)	14.3 (12.2,16.7)	0.0 (0.0,0.4)	14.0 (11.9,16.3)	100.0 (99.6,100.0)
				Pairwise χ^2	82.0 (79.4,84.3)	0.0 (0.0,0.4)	14.4 (12.3,16.8)	0.0 (0.0,0.4)	14.0 (11.9,16.3)	100.0 (99.6,100.0)
L	L	L	L	Res. Cor.	0.0 (0.0,0.4)	32.6 (29.5,35.9)	32.5 (29.4,35.8)	32.3 (29.1,35.5)	35.1 (31.9,38.4)	97.8 (96.5,98.6)
				Pairwise G^2	0.0 (0.0,0.4)	8.0 (6.3,10.1)	5.8 (4.3,7.6)	10.4 (8.4,12.7)	8.3 (6.5,10.3)	81.7 (78.9,84.2)
				Pairwise χ^2	0.0 (0.0,0.4)	8.2 (6.4,10.2)	5.8 (4.3,7.6)	10.5 (8.5,12.8)	8.3 (6.5,10.3)	81.7 (78.9,84.2)
L	L	H	L	Res. Cor.	1.8 (1.0,2.8)	52.7 (49.5,55.9)	54.4 (51.2,57.5)	56.1 (52.9,59.3)	54.5 (51.3,57.7)	100.0 (99.6,100.0)
				Pairwise G^2	0.8 (0.4,1.6)	26.0 (23.2,28.9)	15.4 (13.2,17.8)	26.7 (23.9,29.6)	12.6 (10.5,14.8)	99.9 (99.4,100.0)
				Pairwise χ^2	0.8 (0.4,1.6)	26.1 (23.3,29.0)	15.5 (13.3,17.9)	27.2 (24.4,30.2)	12.8 (10.7,15.1)	99.9 (99.4,100.0)
L	L	H	H	Res. Cor.	100.0 (99.5,100.0)	2.4 (1.4,3.7)	28.7 (25.6,32.0)	2.3 (1.3,3.6)	29.3 (26.2,32.6)	0.0 (0.0,0.5)
				Pairwise G^2	99.9 (99.3,100.0)	0.6 (0.2,1.5)	9.7 (7.7,12.0)	0.3 (0.0,0.9)	7.8 (6.0,9.9)	0.1 (0.0,0.7)
				Pairwise χ^2	99.9 (99.3,100.0)	0.6 (0.2,1.5)	9.7 (7.7,12.0)	0.3 (0.0,0.9)	8.1 (6.3,10.2)	0.1 (0.0,0.7)
L	H	L	H	Res. Cor.	6.0 (4.6,7.7)	85.9 (83.5,88.0)	93.6 (91.8,95.0)	0.3 (0.1,0.9)	0.0 (0.0,0.4)	100.0 (99.6,100.0)
				Pairwise G^2	5.0 (3.7,6.5)	49.5 (46.3,52.8)	75.1 (72.3,77.9)	0.1 (0.0,0.6)	0.0 (0.0,0.4)	100.0 (99.6,100.0)
				Pairwise χ^2	4.8 (3.6,6.4)	49.5 (46.3,52.8)	75.6 (72.7,78.3)	0.1 (0.0,0.6)	0.0 (0.0,0.4)	100.0 (99.6,100.0)
**Sensitivity: L L H H**										

Specificity				Tool	T_1&T_2	T_1&T_3	T_1&T_4	T_2&T_3	T_2&T_4	T_3&T_4
H	H	L	L	Res. Cor.	0.0 (0.0,0.4)	0.9 (0.4,1.7)	0.4 (0.1,1.0)	0.7 (0.3,1.4)	0.9 (0.4,1.7)	99.9 (99.4,100.0)
				Pairwise G^2	0.0 (0.0,0.4)	0.0 (0.0,0.4)	0.0 (0.0,0.4)	0.2 (0.0,0.7)	0.0 (0.0,0.4)	98.3 (97.3,99.0)
				Pairwise χ^2	0.0 (0.0,0.4)	0.0 (0.0,0.4)	0.0 (0.0,0.4)	0.2 (0.0,0.7)	0.0 (0.0,0.4)	98.3 (97.3,99.0)
H	H	H	L	Res. Cor.	0.9 (0.4,1.7)	0.0 (0.0,0.4)	12.4 (10.4,14.6)	0.0 (0.0,0.4)	12.7 (10.7,14.9)	100.0 (99.6,100.0)
				Pairwise G^2	1.4 (0.8,2.3)	0.0 (0.0,0.4)	3.7 (2.6,5.1)	0.0 (0.0,0.4)	3.0 (2.0,4.3)	100.0 (99.6,100.0)
				Pairwise χ^2	1.4 (0.8,2.3)	0.0 (0.0,0.4)	3.8 (2.7,5.2)	0.0 (0.0,0.4)	3.0 (2.0,4.3)	100.0 (99.6,100.0)
H	H	H	H	Res. Cor.	100.0 (99.6,100.0)	0.5 (0.2,1.2)	0.3 (0.1,0.9)	0.4 (0.1,1.0)	0.4 (0.1,1.0)	90.2 (88.2,92.0)
				Pairwise G^2	100.0 (99.6,100.0)	0.1 (0.0,0.6)	0.1 (0.0,0.6)	0.2 (0.0,0.7)	0.0 (0.0,0.4)	95.6 (94.1,96.8)
				Pairwise χ^2	100.0 (99.6,100.0)	0.1 (0.0,0.6)	0.1 (0.0,0.6)	0.2 (0.0,0.7)	0.0 (0.0,0.4)	95.1 (93.6,96.3)
L	L	L	L	Res. Cor.	62.9 (59.7,66.1)	73.5 (70.5,76.4)	70.7 (67.6,73.6)	72.4 (69.3,75.3)	73.1 (70.1,76.0)	100.0 (99.6,100.0)
				Pairwise G^2	41.5 (38.2,44.8)	39.8 (36.5,43.1)	34.9 (31.8,38.2)	33.0 (29.9,36.2)	38.1 (34.9,41.4)	100.0 (99.6,100.0)
				Pairwise χ^2	41.5 (38.2,44.8)	40.0 (36.8,43.3)	35.4 (32.2,38.6)	33.1 (30.1,36.3)	38.2 (35.0,41.5)	100.0 (99.6,100.0)
L	L	H	L	Res. Cor.	100.0 (99.5,100.0)	18.3 (15.5,21.4)	49.8 (46.0,53.6)	19.2 (16.3,22.3)	50.1 (46.3,53.8)	45.2 (41.5,49.0)
				Pairwise G^2	99.9 (99.2,100.0)	12.9 (10.5,15.6)	19.3 (16.4,22.4)	12.2 (9.8,14.8)	19.0 (16.2,22.1)	62.7 (59.0,66.3)
				Pairwise χ^2	99.9 (99.2,100.0)	12.9 (10.5,15.6)	19.3 (16.4,22.4)	12.2 (9.8,14.8)	19.0 (16.2,22.1)	61.9 (58.2,65.6)
L	L	H	H	Res. Cor.	100.0 (99.6,100.0)	0.0 (0.0,0.4)	0.0 (0.0,0.4)	0.2 (0.0,0.9)	0.4 (0.1,1.0)	0.0 (0.0,0.4)
				Pairwise G^2	100.0 (99.6,100.0)	0.0 (0.0,0.4)	0.0 (0.0,0.4)	0.0 (0.0,0.4)	0.0 (0.0,0.4)	0.0 (0.0,0.4)
				Pairwise χ^2	100.0 (99.6,100.0)	0.0 (0.0,0.4)	0.0 (0.0,0.4)	0.0 (0.0,0.4)	0.0 (0.0,0.4)	0.0 (0.0,0.4)
L	H	L	H	Res. Cor.	66.6 (63.5,69.6)	90.4 (88.3,92.2)	99.9 (99.4,100.0)	4.5 (3.2,6.0)	0.0 (0.0,0.4)	100.0 (99.6,100.0)
				Pairwise G^2	65.0 (61.9,68.1)	54.6 (51.3,57.8)	99.0 (98.2,99.6)	0.6 (0.2,1.4)	0.0 (0.0,0.4)	100.0 (99.6,100.0)
				Pairwise χ^2	64.4 (61.2,67.5)	54.7 (51.4,57.9)	99.0 (98.2,99.6)	0.6 (0.2,1.4)	0.0 (0.0,0.4)	100.0 (99.6,100.0)

Table viii.4 Mean absolute biases ($x10^{-2}$) of posterior medians of each parameter obtained from the conditional independence model

Sensitivity: H H L L												
Specificity				π	Se_1	Se_2	Se_3	Se_4	Sp_1	Sp_2	Sp_3	Sp_4
H	H	L	L	-0.7 (-0.8,-0.7)	4.6 (4.4,4.7)	4.6 (4.4,4.7)	-0.1 (-0.2,0.0)	-0.1 (-0.2,0.0)	0.3 (0.2,0.3)	0.3 (0.2,0.3)	-0.2 (-0.3,-0.2)	-0.2 (-0.3,-0.2)
H	H	H	L	-1.2 (-1.3,-1.2)	5.4 (5.3,5.5)	5.4 (5.3,5.5)	-0.1 (-0.3,0.0)	0.1 (0.0,0.2)	0.0 (0.0,0.1)	0.0 (0.0,0.0)	-0.8 (-0.8,-0.8)	-0.3 (-0.4,-0.3)
H	H	H	H	-1.2 (-1.2,-1.1)	5.3 (5.2,5.3)	5.3 (5.2,5.4)	0.1 (0.0,0.2)	0.0 (-0.1,0.1)	0.0 (0.0,0.1)	0.1 (0.0,0.1)	-0.8 (-0.7,-0.7)	-0.8 (-0.8,-0.7)
L	L	L	L	0.7 (0.6,0.9)	3.5 (3.2,3.8)	3.4 (3.1,3.6)	-1.2 (-1.4,-1.0)	-1.0 (-1.2,-0.8)	1.3 (1.2,1.4)	1.3 (1.1,1.4)	-0.1 (-0.2,0.0)	0.0 (-0.1,0.1)
L	L	H	L	-1.7 (-1.8,-1.6)	6.6 (6.5,6.7)	6.5 (6.4,6.6)	-0.7 (-0.9,-0.5)	0.0 (-0.1,0.2)	0.4 (0.4,0.5)	0.4 (0.3,0.5)	-1.2 (-1.3,-1.2)	-0.4 (-0.5,-0.4)
L	L	H	H	-1.7 (-1.7,-1.6)	5.8 (5.7,5.9)	5.9 (5.8,6.0)	0.1 (-0.1,0.2)	0.1 (0.0,0.3)	0.3 (0.2,0.3)	0.3 (0.2,0.4)	-1.0 (-1.0,-1.0)	-1.0 (-1.1,-1.0)
L	H	L	H	-1.4 (-1.5,-1.3)	5.3 (5.2,5.4)	6.5 (6.4,6.6)	-0.1 (-0.2,0.0)	-0.2 (0.3,0.0)	0.3 (0.3,0.4)	0.0 (0.0,0.1)	-0.3 (-0.4,-0.3)	-0.9 (-1.0,-0.9)
Sensitivity: H H H L												
Specificity				π	Se_1	Se_2	Se_3	Se_4	Sp_1	Sp_2	Sp_3	Sp_4
H	H	L	L	-1.2 (-1.2,-1.1)	5.4 (5.3,5.5)	5.3 (5.2,5.4)	-0.1 (-0.2,0.0)	0.0 (-0.1,0.1)	0.0 (0.0,0.1)	0.0 (0.0,0.1)	-0.7 (-0.8,-0.7)	-0.3 (-0.4,-0.3)
H	H	H	L	-1.3 (-1.3,-1.3)	5.4 (5.4,5.5)	5.5 (5.4,5.5)	-0.1 (-0.1,0.0)	0.1 (0.0,0.2)	0.0 (0.0,0.0)	0.0 (0.0,0.0)	-1.3 (-1.3,-1.2)	-0.3 (-0.3,-0.2)
H	H	H	H	-1.1 (-1.2,-1.1)	5.1 (5.0,5.2)	5.2 (5.1,5.2)	0.0 (-0.1,0.0)	0.2 (0.0,0.3)	0.0 (0.0,0.1)	0.0 (0.0,0.1)	-1.2 (-1.2,-1.1)	-0.7 (-0.7,-0.7)

**L**	**L**	**L**	**L**	-1.2 (-1.3,-1.1)	5.7 (5.6,5.8)	5.7 (5.5,5.8)	-1.0 (-1.2,-0.8)	-0.2 (-0.3,-0.1)	0.6 (0.5,0.6)	0.5 (0.5,0.6)	-1.0 (-1.0,-0.9)	-0.3 (-0.4,-0.3)
**L**	**L**	**H**	**L**	-2.2 (-2.2,-2.1)	7.3 (7.2,7.4)	7.3 (7.2,7.4)	-0.4 (-0.7,-0.2)	0.4 (0.3,0.6)	0.2 (0.1,0.3)	0.2 (0.2,0.3)	-2.3 (-2.3,-2.2)	-0.5 (-0.5,-0.4)
**L**	**L**	**H**	**H**	-1.7 (-1.7,-1.6)	5.5 (5.4,5.5)	5.5 (5.4,5.5)	0.5 (0.4,0.7)	0.8 (0.6,0.9)	0.2 (0.1,0.2)	0.2 (0.2,0.3)	-1.5 (-1.6,-1.5)	-0.9 (-0.9,-0.8)
**L**	**H**	**L**	**H**	-1.4 (-1.4,-1.3)	5.2 (5.1,5.2)	6.2 (6.1,6.3)	0.0 (-0.1,0.0)	-0.1 (-0.2,0.1)	0.3 (0.,0.4)	0.0 (0.0,0.1)	-0.9 (-0.9,-0.8)	-0.9 (-0.9,-0.8)
**Sensitivity: H H H H**												
**Specificity**				π	Se_1	Se_2	Se_3	Se_4	Sp_1	Sp_2	Sp_3	Sp_4
**H**	**H**	**L**	**L**	-1.2 (-1.2,-1.1)	5.2 (5.1,5.3)	5.4 (5.3,5.5)	-0.1 (-0.2,0.0)	-0.1 (-0.2,0.0)	0.0 (0.0,0.1)	0.1 (0.0,0.1)	-0.7 (-0.8,-0.7)	-0.8 (-0.8,-0.7)
**H**	**H**	**H**	**L**	-1.2 (-1.2,-1.2)	5.2 (5.2,5.3)	5.3 (5.2,5.3)	0.0 (-0.1,0.0)	0.0 (0.0,0.1)	0.1 (0.0,0.1)	0.0 (0.0,0.1)	-1.2 (-1.2,-1.1)	-0.7 (-0.8,-0.7)
**H**	**H**	**H**	**H**	-1.1 (-1.1,-1.1)	4.9 (4.9,5.0)	4.9 (4.8,4.9)	0.0 (-0.1,0.0)	-0.1 (-0.1,0.0)	0.1 (0.1,0.1)	0.1 (0.0,0.1)	-1.1 (-1.1,-1.0)	-1.1 (-1.1,-1.0)
**L**	**L**	**L**	**L**	-1.6 (-1.7,-1.6)	6.1 (6.0,6.2)	6.0 (5.9,6.1)	0.0 (-0.2,0.1)	0.1 (-0.1,0.2)	0.3 (0.3,0.4)	0.3 (0.3,0.4)	-1.0 (-1.1,-0.9)	-1.0 (-1.1,-1.0)
**L**	**L**	**H**	**L**	-1.9 (-1.9,-1.8)	6.0 (5.9,6.1)	6.0 (5.9,6.1)	0.5 (0.3,0.7)	0.8 (0.7,0.8)	0.2 (0.1,0.2)	0.2 (0.1,0.3)	-1.8 (-1.8,-1.7)	-1.0 (-1.0,-0.9)
**L**	**L**	**H**	**H**	-1.4 (-1.5,-1.4)	4.6 (4.6,4.7)	4.7 (4.6,4.8)	0.8 (0.7,0.9)	0.8 (0.7,0.9)	0.1 (0.1,0.2)	0.2 (0.1,0.2)	-1.2 (-1.3,-1.2)	-1.3 (-1.3,-1.2)
**L**	**H**	**L**	**H**	-1.4 (-1.4,-1.3)	5.1 (5.1,5.2)	6.3 (6.2,6.4)	0.1 (.0,0.2)	0.0 (-0.1,0.1)	0.3 (0.3,0.4)	0.0 (0.0,0.1)	-0.9 (-0.9,-0.8)	-1.4 (-1.4,-1.4)
**Sensitivity: L L L L**												
**Specificity**				π	Se_1	Se_2	Se_3	Se_4	Sp_1	Sp_2	Sp_3	Sp_4
**H**	**H**	**L**	**L**	-6.4 (-6.5,-6.3)	24.5 (24.1,25.0)	24.2 (23.8,24.7)	-0.1 (-0.3,0.1)	0.0 (-0.1,0.2)	0.0 (-0.1,0.1)	0.0 (0.0,0.1)	-1.5 (-1.5,-1.4)	-1.5 (-1.6,-1.5)
**H**	**H**	**H**	**L**	-6.3 (-6.4,-6.2)	23.5 (23.3,23.7)	23.3 (23.1,23.5)	-0.1 (-0.2,0.1)	0.5 (0.3,0.6)	0.0 (0.0,0.1)	0.1 (0.0,0.1)	-3.7 (-3.7,-3.6)	-1.4 (-1.4,-1.3)
**H**	**H**	**H**	**H**	-5.4 (-5.4,-5.3)	20.1 (19.9,20.2)	20.1 (19.9,20.3)	0.7 (0.6,0.9)	0.7 (0.5,0.8)	0.3 (0.2,0.3)	0.3 (0.2,0.3)	-3.1 (-3.1,-3.0)	-3.0 (-3.1,-3.0)
**L**	**L**	**L**	**L**	-7.7 (-8.0,-7.5)	30.6 (30.2,31.1)	30.3 (29.8,30.8)	-2.0 (-2.5,-1.6)	-2.2 (-2.6,-1.7)	2.4 (2.3,2.5)	2.3 (2.1,2.4)	-2.1 (-2.2,-2.0)	-2.1 (-2.2,-2.0)
**L**	**L**	**H**	**L**	-10.2 (-10.3,-10.2)	35.3 (35.2,35.5)	35.4 (35.3,35.5)	-4.6 (-4.9,-4.2)	1.0 (0.8,1.3)	1.5 (1.4,1.6)	1.5 (1.5,1.6)	-6.2 (-6.3,-6.2)	-2.2 (-2.2,-2.1)
**L**	**L**	**H**	**H**	-5.0 (-5.3,-4.8)	11.1 (10.6,11.7)	11.2 (10.7,11.7)	4.7 (4.4,5.0)	4.7 (4.3,5.0)	0.6 (0.5,0.7)	0.6 (0.5,0.6)	-2.1 (-2.2,-2.0)	-2.1 (-2.3,-2.1)
**L**	**H**	**L**	**H**	-8.0 (-8.0,-7.9)	22.9 (22.7,23.1)	35.3 (35.1,35.4)	0.2 (0.1,0.4)	-0.5 (-0.8,-0.3)	1.3 (1.2,1.3)	0.2 (0.1,0.2)	-1.8 (-1.8,-1.7)	-4.7 (-4.7,-4.6)
**Sensitivity: L L H L**												
**Specificity**				π	Se_1	Se_2	Se_3	Se_4	Sp_1	Sp_2	Sp_3	Sp_4
**H**	**H**	**L**	**L**	-6.4 (-6.4,-6.3)	23.7 (23.5,23.9)	23.8 (23.6,24.0)	-0.1 (-0.2,0.0)	0.3 (0.1,0.4)	0.0 (-0.1,0.0)	0.0 (0.0,0.1)	-3.8 (-3.8,-3.7)	-1.5 (-1.5,-1.4)
**H**	**H**	**H**	**L**	-6.3 (-6.3,-6.2)	23.3 (23.2,23.5)	23.2 (23.1,23.4)	-0.1 (-0.2,0.0)	0.9 (0.8,1.1)	0.0 (0.0,0.1)	0.0 (0.0,0.1)	-5.9 (-5.9,-5.8)	-1.3 (-1.4,-1.3)
**H**	**H**	**H**	**H**	-4.3 (-4.4,-4.3)	15.6 (15.5,15.8)	15.6 (15.4,15.8)	0.1 (0.0,0.2)	2.4 (2.2,2.5)	0.3 (0.3,0.3)	0.3 (0.3,0.3)	-4.1 (-4.2,-4.1)	-2.2 (-2.2,-2.1)
**L**	**L**	**L**	**L**	-9.6 (-9.7,-9.5)	34.0 (33.8,34.2)	33.9 (33.7,34.0)	-5.3 (-5.6,-5.0)	0.2 (-0.1,0.4)	1.8 (1.7,1.9)	1.8 (1.7,1.8)	-6.0 (-6.1,-6.0)	-2.2 (-2.2,-2.1)
**L**	**L**	**H**	**L**	-10.8 (-10.9,-10.8)	36.7 (36.6,36.8)	36.5 (36.4,36.6)	-5.6 (-5.9,-5.2)	3.4 (3.1,3.6)	1.3 (1.2,1.4)	1.3 (1.2,1.3)	-10.2 (-10.2,-10.1)	-2.1 (-2.1,-2.0)
**L**	**L**	**H**	**H**	-1.9 (-2.0,-1.8)	4.3 (4.1,4.5)	4.2 (4.0,4.4)	0.4 (0.1,0.6)	4.0 (3.7,4.2)	0.4 (0.4,0.5)	0.4 (0.4,0.5)	-1.9 (-2.0,-1.8)	-0.3 (-0.4,-0.3)
**L**	**H**	**L**	**H**	-6.8 (-6.9,-6.7)	21.0 (20.9,21.2)	26.7 (26.4,27.0)	0.8 (0.7,1.0)	0.8 (0.7,1.0)	1.6 (1.5,1.6)	0.1 (0.0,0.1)	-3.8 (-3.8,-3.7)	-3.8 (-3.9,-3.8)
**Sensitivity: L L H H**												
**Specificity**				π	Se_1	Se_2	Se_3	Se_4	Sp_1	Sp_2	Sp_3	Sp_4
**H**	**H**	**L**	**L**	-5.8 (-5.8,-5.7)	21.9 (21.7,22.0)	22.0 (21.8,22.1)	0.1 (0.0,0.2)	0.1 (0.0,0.2)	0.2 (0.2,0.3)	0.2 (0.2,0.2)	-3.4 (-3.5,-3.4)	-3.4 (-3.5,-3.3)
**H**	**H**	**H**	**L**	-5.3 (-5.3,-5.3)	19.8 (19.6,19.9)	19.7 (19.6,19.9)	0.0 (-0.1,0.1)	1.0 (0.9,1.1)	0.2 (0.2,0.3)	0.2 (0.2,0.3)	-5.0 (-5.0,-5.0)	-3.0 (-3.0,-2.9)
**H**	**H**	**H**	**H**	-1.2 (-1.2,-1.1)	4.6 (4.4,4.7)	4.6 (4.5,4.8)	0.2 (0.1,0.3)	0.2 (0.1,0.3)	0.3 (0.3,0.4)	0.3 (0.3,0.4)	-1.1 (-1.2,-1.1)	-1.2 (-1.2,-1.1)
**L**	**L**	**L**	**L**	-9.7 (-9.8,-9.6)	30.1 (29.8,30.5)	30.2 (29.9,30.5)	2.2 (1.9,2.4)	2.1 (1.9,2.3)	1.2 (1.2,1.3)	1.3 (1.2,1.3)	-5.2 (-5.2,-5.1)	-5.2 (-5.2,-5.1)
**L**	**L**	**H**	**L**	-3.4 (-3.6,-3.2)	7.8 (7.4,8.2)	7.7 (7.3,8.1)	0.6 (0.2,0.9)	4.7 (4.5,4.9)	0.6 (0.6,0.7)	0.6 (0.6,0.7)	-3.2 (-3.4,-3.1)	-1.2 (-1.3,-1.1)
**L**	**L**	**H**	**H**	-0.1 (-0.1,0.0)	1.0 (0.9,1.1)	1.0 (0.9,1.1)	0.1 (-0.2,0.3)	0.0 (-0.2,0.3)	0.3 (0.2,0.3)	0.2 (0.2,0.3)	-0.1 (-0.2,-0.1)	-0.1 (-0.2,0.0)

**L**	**H**	**L**	**H**	-5.6 (-5.7,-5.5)	17.3 (17.1,17.6)	20.4 (20.1,20.7)	1.8 (1.7,1.8)	0.4 (0.3,0.6)	1.5 (1.5,1.6)	0.1 (0.0,0.1)	-3.0 (-3.0,-2.9)	-5.2 (-5.3,-5.1)

Table viii.5 Coverages of the 95% CrIs for each model parameter

**Sensitivity: H H L L**												
**Specificity**				π	Se_1	Se_2	Se_3	Se_4	Sp_1	Sp_2	Sp_3	Sp_4
**H**	**H**	**L**	**L**	93.4 (91.8,95.1)	72.9 (69.9,75.8)	74.0 (71.1,76.9)	96.5 (95.3,97.8)	94.3 (92.8,95.9)	96.9 (95.7,98.0)	96.9 (95.7,98.0)	93.6 (92.0,95.3)	95.3 (93.9,96.7)
**H**	**H**	**H**	**L**	60.8 (57.8,63.8)	9.1 (7.3,10.9)	10.4 (8.5,12.3)	96.5 (95.4,97.6)	95.7 (94.4,97.0)	94.2 (92.8,95.6)	97.0 (95.9,98.1)	66.9 (64.0,69.8)	93.0 (91.4,94.6)
**H**	**H**	**H**	**H**	57.5 (54.4,60.6)	2.0 (1.1,2.9)	1.9 (1.1,2.7)	95.4 (94.1,96.7)	95.4 (94.1,96.7)	95.3 (94.0,96.6)	95.9 (94.7,97.1)	67.2 (64.3,70.1)	67.0 (64.1,69.9)
**L**	**L**	**L**	**L**	98.5 (97.4,99.5)	97.0 (95.5,98.4)	97.0 (95.5,98.4)	93.2 (91.0,95.3)	93.0 (90.8,95.2)	90.3 (87.8,92.8)	88.2 (85.5,91.0)	96.6 (95.0,98.1)	97.0 (95.5,98.4)
**L**	**L**	**H**	**L**	83.4 (81.0,85.7)	16.4 (14.1,18.8)	18.8 (16.3,21.3)	95.1 (93.7,96.5)	95.4 (94.1,96.7)	94.9 (93.5,96.3)	92.9 (91.3,94.5)	57.8 (54.7,60.9)	93.1 (91.5,94.7)
**L**	**L**	**H**	**H**	66.0 (63.1,69.0)	6.8 (5.3,8.4)	5.6 (4.2,7.1)	95.6 (94.3,96.9)	95.6 (94.3,96.9)	93.8 (92.3,95.3)	94.4 (92.9,95.8)	60.0 (57.0,63.0)	60.2 (57.2,63.2)
**L**	**H**	**L**	**H**	70.5 (67.6,73.3)	7.5 (5.9,9.2)	29.0 (26.2,31.9)	95.0 (93.6,96.3)	93.7 (92.2,95.2)	94.2 (92.7,95.6)	93.9 (92.4,95.4)	93.8 (92.3,95.3)	66.0 (63.1,69.0)
**Sensitivity: H H H L**												
**Specificity**				π	Se_1	Se_2	Se_3	Se_4	Sp_1	Sp_2	Sp_3	Sp_4
**H**	**H**	**L**	**L**	71.5 (68.7,74.3)	22.5 (19.9,25.1)	24.5 (21.8,27.1)	96.3 (95.1,97.5)	94.5 (93.1,95.9)	95.4 (94.1,96.7)	96.4 (95.2,97.5)	85.0 (82.7,87.2)	93.7 (92.2,95.2)
**H**	**H**	**H**	**L**	46.6 (43.5,49.7)	0.7 (0.2,1.2)	0.6 (0.1,1.1)	94.2 (92.8,95.6)	94.7 (93.3,96.1)	95.6 (94.3,96.9)	94.0 (92.5,95.5)	36.0 (33.0,39.0)	91.2 (89.4,93.0)
**H**	**H**	**H**	**H**	53.5 (50.4,56.6)	0.1 (0.0,0.3)	0.4 (0.0,0.8)	94.7 (93.3,96.1)	94.3 (92.9,95.7)	95.1 (93.8,96.4)	96.2 (95.0,97.4)	37.6 (34.6,40.6)	69.7 (66.9,72.5)
**L**	**L**	**L**	**L**	94.2 (92.7,95.8)	49.4 (46.1,52.7)	51.0 (47.7,54.3)	93.9 (92.3,95.5)	96.1 (94.9,97.4)	94.2 (92.7,95.8)	93.4 (91.8,95.1)	86.7 (84.5,89.0)	94.1 (92.6,95.7)
**L**	**L**	**H**	**L**	55.6 (52.5,58.8)	0.3 (0.0,0.7)	0.6 (0.1,1.1)	95.1 (93.8,96.5)	93.2 (91.6,94.8)	94.8 (93.4,96.2)	95.6 (94.3,96.9)	9.8 (7.9,11.7)	92.1 (90.4,93.8)
**L**	**L**	**H**	**H**	48.8 (45.7,51.9)	2.1 (1.2,3.0)	1.4 (0.7,2.1)	94.8 (93.4,96.2)	93.4 (91.9,94.9)	94.0 (92.5,95.5)	94.3 (92.9,95.7)	32.6 (29.7,35.5)	64.5 (61.5,67.5)
**L**	**H**	**L**	**H**	62.6 (59.6,65.6)	2.6 (1.6,3.6)	14.3 (12.1,16.5)	95.3 (94.0,96.6)	94.8 (93.4,96.2)	92.7 (91.1,94.3)	95.6 (94.3,96.9)	82.3 (79.9,84.7)	64.7 (61.7,67.7)
**Sensitivity: H H H H**												
**Specificity**				π	Se_1	Se_2	Se_3	Se_4	Sp_1	Sp_2	Sp_3	Sp_4
**H**	**H**	**L**	**L**	61.5 (58.5,64.5)	5.9 (4.4,7.4)	5.7 (4.3,7.1)	94.4 (93.0,95.8)	94.1 (92.6,95.6)	95.4 (94.1,96.7)	93.7 (92.2,95.2)	82.8 (80.5,85.1)	84.7 (82.5,86.9)
**H**	**H**	**H**	**L**	49.5 (46.4,52.6)	0.1 (0.0,0.3)	0.3 (0.0,0.6)	95.5 (94.2,96.8)	94.3 (92.9,95.7)	93.7 (92.2,95.2)	94.7 (93.3,96.1)	36.4 (33.4,39.4)	85.5 (83.3,87.7)
**H**	**H**	**H**	**H**	55.9 (52.8,59.0)	0.1 (0.0,0.3)	0.1 (0.0,0.3)	95.0 (93.6,96.4)	93.9 (92.4,95.4)	94.0 (92.5,95.5)	95.6 (94.3,96.9)	42.0 (38.9,45.1)	42.4 (39.9,45.5)
**L**	**L**	**L**	**L**	77.2 (74.5,79.8)	19.5 (17.1,22.0)	20.7 (18.1,23.2)	95.4 (94.0,96.7)	95.3 (93.9,96.6)	95.1 (93.7,96.4)	93.6 (92.1,95.2)	81.4 (78.9,83.8)	81.2 (78.7,83.6)
**L**	**L**	**H**	**L**	47.1 (44.1,50.2)	0.2 (0.0,0.5)	1.0 (0.4,1.6)	94.5 (93.1,95.9)	91.6 (89.9,93.3)	95.8 (94.6,97.0)	95.7 (94.4,97.0)	24.3 (21.7,27.0)	79.8 (77.3,82.3)
**L**	**L**	**H**	**H**	50.2 (47.1,53.3)	3.4 (2.3,4.5)	2.1 (1.2,3.0)	91.6 (89.9,99.3)	92.5 (90.9,94.1)	94.2 (92.8,95.6)	93.5 (92.0,95.0)	44.1 (41.0,47.2)	42.3 (39.2,45.4)
**L**	**H**	**L**	**H**	47.9 (44.8,51.0)	0.0 (0.0,0.0)	1.0 (0.4,1.6)	94.8 (93.4,96.2)	93.9 (92.4,95.4)	93.7 (92.2,95.2)	94.8 (93.4,96.2)	80.8 (78.4,83.2)	30.1 (27.3,32.9)
**Sensitivity: L L L L**												
**Specificity**				π	Se_1	Se_2	Se_3	Se_4	Sp_1	Sp_2	Sp_3	Sp_4
**H**	**H**	**L**	**L**	13.3 (10.6,15.9)	0.5 (0.0,1.0)	1.4 (0.5,2.3)	95.1 (93.4,96.8)	96.2 (94.7,97.7)	94.9 (93.2,96.7)	97.9 (96.8,99.1)	55.1 (51.3,59.0)	52.0 (48.1,55.9)
**H**	**H**	**H**	**L**	0.0 (0.0,0.0)	0.0 (0.0,0.0)	0.0 (0.0,0.0)	95.3 (94.0,96.7)	95.0 (93.7,96.4)	93.9 (92.4,95.4)	95.2 (93.9,96.6)	0.0 (0.0,0.0)	57.2 (54.1,60.3)
**H**	**H**	**H**	**H**	0.2 (0.0,0.5)	0.0 (0.0,0.0)	0.0 (0.0,0.0)	94.3 (92.8,95.7)	95.5 (94.2,96.8)	92.9 (91.3,94.5)	91.8 (90.0,93.5)	0.0 (0.0,0.0)	0.4 (0.0,0.8)
**L**	**L**	**L**	**L**	57.9 (52.1,63.6)	1.8 (0.2,3.3)	0.7 (0.0,1.7)	92.1 (89.0,95.3)	91.4 (88.1,94.7)	51.8 (45.9,57.6)	55.0 (49.2,60.8)	39.3 (33.6,45.0)	41.4 (35.7,47.2)
**L**	**L**	**H**	**L**	0.0 (0.0,0.0)	0.0 (0.0,0.0)	0.0 (0.0,0.0)	87.3 (85.2,89.5)	93.8 (92.2,95.4)	69.6 (66.6,72.6)	66.8 (63.7,69.9)	0.0 (0.0,0.0)	23.6 (20.9,26.4)
**L**	**L**	**H**	**H**	49.3 (45.5,53.0)	23.0 (19.8,26.2)	24.9 (21.6,28.2)	83.9 (81.1,86.7)	83.4 (80.6,86.2)	91.3 (89.2,93.5)	91.3 (89.2,93.5)	53.6 (49.8,57.4)	52.1 (48.3,55.9)
**L**	**H**	**L**	**H**	0.1 (0.0,0.3)	0.0 (0.0,0.0)	0.0 (0.0,0.0)	95.4 (94.1,96.8)	95.3 (94.0,96.7)	66.6 (63.5,69.6)	94.2 (92.8,95.7)	38.9 (35.8,42.0)	0.0 (0.0,0.0)

Sensitivity: L L H L												
Specificity				π	Se_1	Se_2	Se_3	Se_4	Sp_1	Sp_2	Sp_3	Sp_4
**H**	**H**	**L**	**L**	0.1 (0.0,0.3)	0.0 (0.0,0.0)	0.0 (0.0,0.0)	95.3 (93.9,96.6)	94.2 (92.8,95.7)	95.1 (93.7,96.4)	94.7 (93.3,96.2)	1.0 (0.4,1.7)	53.2 (50.1,56.4)
**H**	**H**	**H**	**L**	0.0 (0.0,0.0)	0.0 (0.0,0.0)	0.0 (0.0,0.0)	95.5 (94.2,96.8)	92.7 (91.1,94.3)	95.6 (94.3,96.9)	95.3 (94.0,96.6)	0.0 (0.0,0.0)	58.5 (55.4,61.6)
**H**	**H**	**H**	**H**	0.4 (0.0,0.8)	0.0 (0.0,0.0)	0.0 (0.0,0.0)	96.1 (94.9,97.3)	77.7 (75.1,80.2)	92.0 (90.3,93.7)	91.0 (89.2,92.8)	0.0 (0.0,0.0)	7.6 (6.0,9.3)
**L**	**L**	**L**	**L**	2.0 (1.1,3.0)	0.0 (0.0,0.0)	0.0 (0.0,0.0)	85.5 (83.1,87.8)	94.6 (93.0,96.1)	59.9 (56.6,63.2)	62.6 (59.4,65.9)	0.0 (0.0,0.0)	26.8 (23.8,29.8)
**L**	**L**	**H**	**L**	0.0 (0.0,0.0)	0.0 (0.0,0.0)	0.0 (0.0,0.0)	88.5 (86.4,90.5)	82.6 (80.2,85.0)	71.3 (68.5,74.2)	71.9 (69.1,74.8)	0.0 (0.0,0.0)	24.4 (21.7,27.1)
**L**	**L**	**H**	**H**	79.3 (76.5,82.2)	54.0 (50.6,57.5)	54.5 (51.1,58.0)	97.5 (96.4,98.6)	79.0 (76.1,81.8)	90.8 (88.8,92.8)	92.7 (90.9,94.5)	65.4 (62.1,68.7)	92.9 (91.2,94.7)
**L**	**H**	**L**	**H**	0.7 (0.2,1.3)	0.0 (0.0,0.0)	0.0 (0.0,0.0)	92.6 (91.0,94.3)	95.0 (93.7,96.4)	50.8 (47.6,54.0)	95.4 (94.0,96.7)	3.2 (2.0,4.3)	0.3 (0.0,0.7)
**Sensitivity: L L H H**												
**Specificity**				π	Se_1	Se_2	Se_3	Se_4	Sp_1	Sp_2	Sp_3	Sp_4
**H**	**H**	**L**	**L**	0.0 (0.0,0.0)	0.0 (0.0,0.0)	0.0 (0.0,0.0)	95.6 (94.3,96.9)	95.0 (93.6,96.4)	92.5 (90.9,94.1)	92.2 (90.5,93.9)	1.2 (0.5,.1.9)	1.8 (1.0,2.6)
**H**	**H**	**H**	**L**	0.0 (0.0,0.0)	0.0 (0.0,0.0)	0.0 (0.0,0.0)	93.7 (92.2,95.2)	89.1 (87.2,91.0)	93.5 (92.0,95.0)	93.2 (91.6,94.8)	0.0 (0.0,0.0)	5.9 (4.4,7.4)
**H**	**H**	**H**	**H**	63.4 (60.4,66.4)	37.1 (34.1,40.1)	36.5 (33.6,39.5)	94.8 (93.4,96.2)	95.0 (93.6,96.3)	90.0 (88.1,91.9)	90.8 (89.0,92.6)	53.4 (50.3,56.4)	51.9 (48.8,55.0)
**L**	**L**	**L**	**L**	1.6 (0.8,2.4)	0.1 (0.0,0.3)	0.1 (0.0,0.3)	95.2 (93.8,96.6)	93.9 (92.4,95.5)	75.2 (72.3,78.0)	72.1 (69.2,75.1)	1.8 (0.9,2.7)	1.3 (0.6,2.1)
**L**	**L**	**H**	**L**	61.8 (58.2,65.4)	34.8 (31.2,38.3)	34.9 (31.4,38.4)	98.4 (97.5,99.3)	58.4 (54.7,62.0)	88.1 (85.7,90.5)	88.1 (85.7,90.5)	35.9 (32.4,39.5)	81.1 (78.2,84.0)
**L**	**L**	**H**	**H**	95.1 (93.7,96.6)	90.6 (88.6,92.6)	90.6 (88.6,92.6)	95.1 (93.7,96.6)	95.7 (94.3,97.1)	92.9 (91.1,94.6)	95.2 (93.8,96.7)	95.1 (93.7,96.6)	94.4 (92.9,96.0)
**L**	**H**	**L**	**H**	1.7 (0.9,2.5)	0.0 (0.0,0.0)	0.4 (0.0,0.8)	81.4 (79.0,83.9)	94.6 (93.1,96.0)	50.2 (47.0,53.4)	93.4 (91.8,95.0)	13.4 (11.3,15.6)	0.3 (0.0,0.7)

ix. Setting 9: $\pi = 0.2, \omega = 0.9, n_{obs} = 2000$

Table ix.1 Number of converged data sets for different sensitivity-specificity combinations

				Number of converged data sets
**Specificity**				**Sensitivity: H H L L**
**H**	**H**	**L**	**L**	939
**H**	**H**	**H**	**L**	1000
**H**	**H**	**H**	**H**	1000
**L**	**L**	**L**	**L**	595
**L**	**L**	**H**	**L**	983
**L**	**L**	**H**	**H**	1000
**L**	**H**	**L**	**H**	999
**Specificity**				**Sensitivity: H H H L**
**H**	**H**	**L**	**L**	1000
**H**	**H**	**H**	**L**	1000
**H**	**H**	**H**	**H**	1000
**L**	**L**	**L**	**L**	963
**L**	**L**	**H**	**L**	994

**L**	**L**	**H**	**H**	1000
**L**	**H**	**L**	**H**	1000
**Specificity**				**Sensitivity: H H H H**
**H**	**H**	**L**	**L**	1000
**H**	**H**	**H**	**L**	1000
**H**	**H**	**H**	**H**	1000
**L**	**L**	**L**	**L**	997
**L**	**L**	**H**	**L**	1000
**L**	**L**	**H**	**H**	1000
**L**	**H**	**L**	**H**	1000
**Specificity**				**Sensitivity: L L L L**
**H**	**H**	**L**	**L**	852
**H**	**H**	**H**	**L**	1000
**H**	**H**	**H**	**H**	1000
**L**	**L**	**L**	**L**	346
**L**	**L**	**H**	**L**	989
**L**	**L**	**H**	**H**	988
**L**	**H**	**L**	**H**	999
**Specificity**				**Sensitivity: L L H L**
**H**	**H**	**L**	**L**	999
**H**	**H**	**H**	**L**	999
**H**	**H**	**H**	**H**	1000
**L**	**L**	**L**	**L**	960
**L**	**L**	**H**	**L**	989
**L**	**L**	**H**	**H**	920
**L**	**H**	**L**	**H**	1000
**Specificity**				**Sensitivity: L L H H**
**H**	**H**	**L**	**L**	1000
**H**	**H**	**H**	**L**	1000
**H**	**H**	**H**	**H**	998
**L**	**L**	**L**	**L**	988
**L**	**L**	**H**	**L**	998
**L**	**L**	**H**	**H**	721
**L**	**H**	**L**	**H**	1000

Table ix.2 Percentages of the time 95% credible intervals (CrIs) for residual correlations did not included '0' for any pairs, and percentages of the time that the overall χ^2 or G^2 statistic indicated a lack of overall fit

**Sensitivity: H H L L**			
**Specificity**	**Res. Cor.**	**Overall G^2**	**Overall χ^2**

**H**	**H**	**L**	**L**	65.4 (62.2,68.4)	13.3 (11.2,15.7)	12.5 (10.4,14.7)
**H**	**H**	**H**	**L**	27.9 (25.1,30.8)	16.4 (14.2,18.8)	13.2 (11.2,15.5)
**H**	**H**	**H**	**H**	36.6 (33.6,39.7)	40.9 (37.8,44.0)	49.4 (46.3,52.5)
**L**	**L**	**L**	**L**	56.3 (52.2,60.3)	19.5 (16.4,22.9)	19.0 (15.9,22.4)
**L**	**L**	**H**	**L**	35.4 (32.4,38.5)	43.2 (40.1,46.4)	43.7 (40.6,46.9)
**L**	**L**	**H**	**H**	40.3 (37.2,43.4)	86.3 (84.0,88.4)	89.9 (87.9,91.7)
**L**	**H**	**L**	**H**	51.0 (47.8,54.1)	28.5 (25.7,31.4)	26.7 (24.0,29.6)
**Sensitivity: H H H L**						
**Specificity**				**Res. Cor.**	**Overall G^2**	**Overall χ^2**
**H**	**H**	**L**	**L**	38.9 (35.9,42.0)	12.5 (10.5,14.7)	11.5 (9.6,13.6)
**H**	**H**	**H**	**L**	12.8 (10.8,15.0)	21.3 (18.8,24.0)	17.7 (15.4,20.2)
**H**	**H**	**H**	**H**	60.0 (56.9,63.1)	74.7 (71.9,77.4)	84.2 (81.8,86.4)
**L**	**L**	**L**	**L**	49.7 (46.5,52.9)	29.3 (26.4,32.3)	30.2 (27.3,33.2)
**L**	**L**	**H**	**L**	46.1 (42.9,49.2)	70.0 (67.1,72.9)	70.8 (67.9,73.6)
**L**	**L**	**H**	**H**	71.2 (68.3,74.0)	98.0 (96.9,98.8)	99.8 (99.3,100.0)
**L**	**H**	**L**	**H**	58.1 (55.0,61.2)	48.1 (45.0,51.2)	49.5 (46.4,52.6)
**Sensitivity: H H H H**						
**Specificity**				**Res. Cor.**	**Overall G^2**	**Overall χ^2**
**H**	**H**	**L**	**L**	46.8 (43.7,49.9)	18.3 (15.9,20.8)	17.5 (15.2,20.0)
**H**	**H**	**H**	**L**	62.9 (59.8,65.9)	54.4 (51.3,57.5)	52.7 (49.6,55.8)
**H**	**H**	**H**	**H**	97.0 (95.7,98.0)	96.0 (94.6,97.1)	99.4 (98.7,99.8)
**L**	**L**	**L**	**L**	65.2 (62.1,68.2)	50.8 (47.6,53.9)	51.5 (48.3,54.6)
**L**	**L**	**H**	**L**	78.9 (76.2,81.4)	92.5 (90.7,94.1)	93.1 (91.3,94.6)
**L**	**L**	**H**	**H**	98.1 (97.0,98.9)	99.6 (99.0,99.9)	100.0 (99.6,100.0)
**L**	**H**	**L**	**H**	79.6 (77.0,82.1)	80.8 (78.2,83.2)	81.9 (79.4,84.2)
**Sensitivity: L L L L**						
**Specificity**				**Res. Cor.**	**Overall G^2**	**Overall χ^2**
**H**	**H**	**L**	**L**	73.8 (70.7,76.7)	18.4 (15.9,21.2)	17.7 (15.2,20.5)
**H**	**H**	**H**	**L**	82.1 (79.6,84.4)	55.2 (52.1,58.3)	52.3 (49.2,55.4)
**H**	**H**	**H**	**H**	100.0 (99.6,100.0)	98.8 (97.9,99.4)	100.0 (99.6,100.0)
**L**	**L**	**L**	**L**	84.1 (79.8,87.8)	55.8 (50.4,61.1)	56.4 (51.0,61.7)
**L**	**L**	**H**	**L**	99.8 (99.3,100.0)	99.9 (99.4,100.0)	99.9 (99.4,100.0)
**L**	**L**	**H**	**H**	100.0 (99.6,100.0)	100.0 (99.6,100.0)	100.0 (99.6,100.0)
**L**	**H**	**L**	**H**	100.0 (99.6,100.0)	98.1 (97.0,98.9)	98.0 (96.9,98.8)
**Sensitivity: L L H L**						
**Specificity**				**Res. Cor.**	**Overall G^2**	**Overall χ^2**
**H**	**H**	**L**	**L**	81.9 (79.4,84.2)	39.3 (36.3,42.4)	38.4 (35.4,41.5)
**H**	**H**	**H**	**L**	94.1 (92.5,95.5)	83.1 (80.6,85.4)	81.8 (79.2,84.1)
**H**	**H**	**H**	**H**	100.0 (99.6,100.0)	99.8 (99.3,100.0)	100.0 (99.6,100.0)
**L**	**L**	**L**	**L**	99.2 (98.4,96.6)	98.3 (97.3,99.0)	98.5 (97.6,99.2)
**L**	**L**	**H**	**L**	100.0 (99.6,100.0)	100.0 (99.6,100.0)	100.0 (99.6,100.0)

**L**	**L**	**H**	**H**	100.0 (99.6,100.0)	100.0 (99.6,100.0)	100.0 (99.6,100.0)
**L**	**H**	**L**	**H**	100.0 (99.6,100.0)	99.7 (99.1,99.9)	100.0 (99.6,100.0)
**Sensitivity: L L H H**						
**Specificity**				**Res. Cor.**	**Overall G^2**	**Overall χ^2**
**H**	**H**	**L**	**L**	99.7 (99.1,99.9)	90.7 (88.7,92.4)	90.8 (88.8,92.5)
**H**	**H**	**H**	**L**	100.0 (99.6,100.0)	100.0 (99.6,100.0)	100.0 (99.6,100.0)
**H**	**H**	**H**	**H**	100.0 (99.6,100.0)	100.0 (99.6,100.0)	100.0 (99.6,100.0)
**L**	**L**	**L**	**L**	100.0 (99.6,100.0)	100.0 (99.6,100.0)	100.0 (99.6,100.0)
**L**	**L**	**H**	**L**	100.0 (99.6,100.0)	100.0 (99.6,100.0)	100.0 (99.6,100.0)
**L**	**L**	**H**	**H**	100.0 (99.5,100.0)	100.0 (99.5,100.0)	100.0 (99.5,100.0)
**L**	**H**	**L**	**H**	100.0 (99.6,100.0)	100.0 (99.6,100.0)	100.0 (99.6,100.0)

Table ix.3 Percentages of the time 95% CrIs for residual correlations did not included '0' for each pair, and percentages of the time the pairwise χ^2 or G^2 statistic for each pair indicated a lack of pairwise fit

**Sensitivity: H H L L**										
**Specificity**				**Tool**	**T_1&T_2**	**T_1&T_3**	**T_1&T_4**	**T_2&T_3**	**T_2&T_4**	**T_3&T_4**
**H**	**H**	**L**	**L**	**Res. Cor.**	0.0 (0.0,0.4)	0.2 (0.0,0.8)	0.0 (0.0,0.4)	0.4 (0.1,1.1)	0.1 (0.0,0.6)	64.9 (61.7,67.9)
				**Pairwise G^2**	0.0 (0.0,0.4)	0.0 (0.0,0.4)	0.0 (0.0,0.4)	0.1 (0.0,0.4)	0.1 (0.0,0.4)	5.1 (3.8,6.7)
				**Pairwise χ^2**	0.0 (0.0,0.4)	0.0 (0.0,0.4)	0.0 (0.0,0.4)	0.1 (0.0,0.4)	0.1 (0.0,0.4)	5.1 (3.8,6.7)
**H**	**H**	**H**	**L**	**Res. Cor.**	0.0 (0.0,0.4)	0.0 (0.0,0.4)	0.5 (0.2,1.2)	0.0 (0.0,0.4)	0.6 (0.2,1.3)	27.3 (24.6,30.2)
				**Pairwise G^2**	0.0 (0.0,0.4)	0.0 (0.0,0.4)	0.0 (0.0,0.4)	0.0 (0.0,0.4)	0.0 (0.0,0.4)	3.6 (2.5,4.9)
				**Pairwise χ^2**	0.0 (0.0,0.4)	0.0 (0.0,0.4)	0.0 (0.0,0.4)	0.0 (0.0,0.4)	0.0 (0.0,0.4)	3.6 (2.5,4.9)
**H**	**H**	**H**	**H**	**Res. Cor.**	0.0 (0.0,0.4)	0.0 (0.0,0.4)	0.0 (0.0,0.4)	0.0 (0.0,0.4)	0.0 (0.0,0.4)	36.6 (33.6,39.7)
				**Pairwise G^2**	0.0 (0.0,0.4)	0.0 (0.0,0.4)	0.0 (0.0,0.4)	0.0 (0.0,0.4)	0.0 (0.0,0.4)	15.7 (13.5,18.1)
				**Pairwise χ^2**	0.0 (0.0,0.4)	0.0 (0.0,0.4)	0.0 (0.0,0.4)	0.0 (0.0,0.4)	0.0 (0.0,0.4)	15.9 (13.7,18.3)
**L**	**L**	**L**	**L**	**Res. Cor.**	0.0 (0.0,0.6)	4.4 (2.9,6.3)	5.9 (4.1,8.1)	4.5 (3.0,6.5)	3.2 (1.9,4.9)	46.4 (42.3,50.5)
				**Pairwise G^2**	0.0 (0.0,0.6)	0.3 (0.0,1.2)	0.5 (0.1,1.5)	0.0 (0.0,0.6)	0.8 (0.3,2.0)	2.4 (1.3,3.9)
				**Pairwise χ^2**	0.0 (0.0,0.6)	0.3 (0.0,1.2)	0.7 (0.2,1.7)	0.2 (0.0,0.9)	0.8 (0.3,2.0)	2.4 (1.3,3.9)
**L**	**L**	**H**	**L**	**Res. Cor.**	0.0 (0.0,0.4)	2.3 (1.5,3.5)	13.4 (11.4,15.7)	1.6 (0.9,2.6)	11.9 (9.9,14.1)	14.6 (12.5,17.0)
				**Pairwise G^2**	0.0 (0.0,0.4)	0.5 (0.2,1.2)	2.3 (1.5,3.5)	0.1 (0.0,0.6)	1.8 (1.1,2.9)	2.7 (1.8,4.0)
				**Pairwise χ^2**	0.0 (0.0,0.4)	0.6 (0.2,1.3)	2.4 (1.6,3.6)	0.1 (0.0,0.6)	1.8 (1.1,2.9)	2.7 (1.8,4.0)
**L**	**L**	**H**	**H**	**Res. Cor.**	4.5 (3.3,6.0)	6.0 (4.6,7.7)	6.1 (4.7,7.8)	5.7 (4.3,7.3)	6.0 (4.6,7.7)	26.9 (24.2,29.8)
				**Pairwise G^2**	0.9 (0.4,1.7)	0.6 (0.2,1.3)	1.4 (0.8,2.3)	1.4 (0.8,2.3)	0.6 (0.2,1.3)	16.2 (14.0,18.6)
				**Pairwise χ^2**	0.8 (0.3,1.6)	0.9 (0.4,1.7)	1.4 (0.8,2.3)	1.7 (1.0,2.7)	0.7 (0.3,1.4)	16.6 (14.3,19.1)
**L**	**H**	**L**	**H**	**Res. Cor.**	0.0 (0.0,0.4)	24.2 (21.6,27.0)	16.3 (14.1,18.8)	0.0 (0.0,0.4)	0.0 (0.0,0.4)	21.8 (19.3,24.5)
				**Pairwise G^2**	0.0 (0.0,0.4)	2.8 (1.9,4.0)	3.7 (2.6,5.1)	0.0 (0.0,0.4)	0.0 (0.0,0.4)	4.5 (3.3,6.0)
				**Pairwise χ^2**	0.0 (0.0,0.4)	2.9 (2.0,4.1)	3.7 (2.6,5.1)	0.0 (0.0,0.4)	0.0 (0.0,0.4)	4.4 (3.2,5.9)
**Sensitivity: H H H L**										

Specificity				Tool	T_1&T_2	T_1&T_3	T_1&T_4	T_2&T_3	T_2&T_4	T_3&T_4
**H**	**H**	**L**	**L**	**Res. Cor.**	0.0 (0.0,0.4)	0.4 (0.1,1.0)	0.2 (0.0,0.7)	0.3 (0.1,0.9)	0.4 (0.1,1.0)	38.3 (35.3,41.4)
				**Pairwise G^2**	0.0 (0.0,0.4)	0.0 (0.0,0.4)	0.0 (0.0,0.4)	0.1 (0.0,0.6)	0.1 (0.0,0.6)	4.9 (3.6,6.4)
				**Pairwise χ^2**	0.0 (0.0,0.4)	0.0 (0.0,0.4)	0.0 (0.0,0.4)	0.1 (0.0,0.6)	0.1 (0.0,0.6)	4.9 (3.6,6.4)
**H**	**H**	**H**	**L**	**Res. Cor.**	0.0 (0.0,0.4)	0.0 (0.0,0.4)	0.7 (0.3,1.4)	0.0 (0.0,0.4)	0.5 (0.2,1.2)	11.6 (9.7,13.7)
				**Pairwise G^2**	0.0 (0.0,0.4)	0.0 (0.0,0.4)	0.1 (0.0,0.6)	0.0 (0.0,0.4)	0.0 (0.0,0.4)	2.8 (1.9,4.0)
				**Pairwise χ^2**	0.0 (0.0,0.4)	0.0 (0.0,0.4)	0.1 (0.0,0.6)	0.0 (0.0,0.4)	0.0 (0.0,0.4)	2.8 (1.9,4.0)
**H**	**H**	**H**	**H**	**Res. Cor.**	0.0 (0.0,0.4)	0.0 (0.0,0.4)	0.0 (0.0,0.4)	0.0 (0.0,0.4)	0.0 (0.0,0.4)	60.0 (56.9,63.1)
				**Pairwise G^2**	0.0 (0.0,0.4)	0.0 (0.0,0.4)	0.0 (0.0,0.4)	0.0 (0.0,0.4)	0.0 (0.0,0.4)	41.6 (38.5,44.7)
				**Pairwise χ^2**	0.0 (0.0,0.4)	0.0 (0.0,0.4)	0.0 (0.0,0.4)	0.0 (0.0,0.4)	0.0 (0.0,0.4)	41.6 (38.5,44.7)
**L**	**L**	**L**	**L**	**Res. Cor.**	0.0 (0.0,0.4)	7.5 (5.9,9.3)	11.5 (9.6,13.7)	8.5 (6.8,10.5)	12.7 (10.6,14.9)	24.1 (21.4,26.9)
				**Pairwise G^2**	0.0 (0.0,0.4)	1.1 (0.6,2.0)	1.6 (0.9,2.6)	1.6 (0.9,2.6)	1.2 (0.6,2.2)	3.4 (2.4,4.8)
				**Pairwise χ^2**	0.0 (0.0,0.4)	1.1 (0.6,2.0)	1.6 (0.9,2.6)	1.6 (0.9,2.6)	1.2 (0.6,2.2)	3.4 (2.4,4.8)
**L**	**L**	**H**	**L**	**Res. Cor.**	0.2 (0.0,0.7)	4.9 (3.7,6.5)	20.2 (17.8,22.9)	3.3 (2.3,4.6)	21.7 (19.2,24.4)	9.5 (7.7,11.4)
				**Pairwise G^2**	0.0 (0.0,0.4)	1.4 (0.8,2.4)	2.9 (2.0,4.2)	1.4 (0.8,2.4)	3.3 (2.3,4.6)	2.8 (1.9,4.0)
				**Pairwise χ^2**	0.0 (0.0,0.4)	1.6 (0.9,2.6)	3.0 (2.0,4.3)	1.4 (0.8,2.4)	3.3 (2.3,4.6)	2.8 (1.9,4.0)
**L**	**L**	**H**	**H**	**Res. Cor.**	26.0 (23.3,26.8)	8.4 (6.8,10.3)	8.1 (6.5,10.0)	9.4 (7.7,11.4)	8.5 (6.8,10.4)	55.6 (52.5,58.7)
				**Pairwise G^2**	6.5 (5.1,8.2)	3.1 (2.1,4.4)	2.2 (1.4,3.3)	2.6 (1.7,3.8)	1.8 (1.1,2.8)	42.6 (39.5,45.7)
				**Pairwise χ^2**	6.4 (5.0,8.1)	3.4 (2.4,4.7)	2.6 (1.7,3.8)	3.1 (2.1,4.4)	2.1 (1.3,3.2)	42.3 (38.2,45.4)
**L**	**H**	**L**	**H**	**Res. Cor.**	0.0 (0.0,0.4)	30.6 (57.8,33.6)	16.6 (14.3,19.1)	0.0 (0.0,0.4)	0.0 (0.0,0.4)	34.0 (31.1,37.0)
				**Pairwise G^2**	0.0 (0.0,0.4)	4.9 (3.6,6.4)	3.6 (2.5,4.9)	0.0 (0.0,0.4)	0.0 (0.0,0.4)	11.8 (9.9,14.0)
				**Pairwise χ^2**	0.0 (0.0,0.4)	5.1 (3.8,6.7)	3.7 (2.6,5.1)	0.0 (0.0,0.4)	0.0 (0.0,0.4)	11.4 (9.5,13.5)
**Sensitivity: H H H H**										
**Specificity**				**Tool**	**T_1&T_2**	**T_1&T_3**	**T_1&T_4**	**T_2&T_3**	**T_2&T_4**	**T_3&T_4**
**H**	**H**	**L**	**L**	**Res. Cor.**	0.0 (0.0,0.4)	0.7 (0.3,1.4)	0.6 (0.2,1.3)	1.0 (0.5,1.8)	0.8 (0.3,1.6)	45.5 (42.4,48.6)
				**Pairwise G^2**	0.0 (0.0,0.4)	0.4 (0.1,1.0)	0.3 (0.1,0.9)	0.1 (0.0,0.6)	0.2 (0.0,0.7)	7.8 (6.2,9.6)
				**Pairwise χ^2**	0.0 (0.0,0.4)	0.4 (0.1,1.0)	0.3 (0.1,0.9)	0.2 (0.0,0.7)	0.2 (0.0,0.7)	7.8 (6.2,9.6)
**H**	**H**	**H**	**L**	**Res. Cor.**	0.0 (0.0,0.4)	0.0 (0.0,0.4)	1.7 (1.0,2.7)	0.0 (0.0,0.4)	1.6 (0.9,2.6)	62.1 (59.0,65.1)
				**Pairwise G^2**	0.0 (0.0,0.4)	0.0 (0.0,0.4)	0.6 (0.2,1.3)	0.0 (0.0,0.4)	0.3 (0.1,0.9)	33.3 (30.4,36.3)
				**Pairwise χ^2**	0.0 (0.0,0.4)	0.0 (0.0,0.4)	0.6 (0.2,1.3)	0.0 (0.0,0.4)	0.3 (0.1,0.9)	32.3 (29.4,35.3)
**H**	**H**	**H**	**H**	**Res. Cor.**	0.0 (0.0,0.4)	0.0 (0.0,0.4)	0.0 (0.0,0.4)	0.0 (0.0,0.4)	0.0 (0.0,0.4)	97.0 (95.7,98.0)
				**Pairwise G^2**	0.0 (0.0,0.4)	0.0 (0.0,0.4)	0.6 (0.2,1.3)	0.0 (0.0,0.4)	0.3 (0.1,0.9)	33.3 (30.4,36.3)
				**Pairwise χ^2**	0.0 (0.0,0.4)	0.0 (0.0,0.4)	0.6 (0.2,1.3)	0.0 (0.0,0.4)	0.3 (0.1,0.9)	32.3 (29.4,35.3)
**L**	**L**	**L**	**L**	**Res. Cor.**	4.1 (3.0,5.5)	16.5 (14.3,19.0)	16.0 (13.8,18.5)	16.1 (13.9,18.6)	16.9 (14.6,19.3)	30.8 (27.9,33.8)
				**Pairwise G^2**	1.1 (0.6,2.0)	1.9 (1.2,3.0)	3.1 (2.1,4.4)	3.1 (2.1,4.4)	2.5 (1.6,4.7)	6.9 (5.4,8.7)
				**Pairwise χ^2**	1.1 (0.6,2.0)	2.0 (1.2,3.1)	3.3 (2.3,4.6)	3.2 (2.2,4.5)	2.5 (1.6,4.7)	6.9 (5.4,8.7)
**L**	**L**	**H**	**L**	**Res. Cor.**	16.6 (14.3,19.1)	7.7 (6.1,9.5)	22.6 (20.0,25.3)	7.1 (5.6,8.9)	26.3 (23.6,29.1)	61.2 (58.1,64.2)
				**Pairwise G^2**	3.6 (2.5,4.9)	2.8 (1.9,4.0)	3.9 (2.8,5.3)	1.9 (1.1,3.0)	4.6 (3.4,6.1)	35.5 (32.5,38.6)
				**Pairwise χ^2**	3.6 (2.5,4.9)	3.1 (2.1,4.4)	3.9 (2.8,5.3)	2.2 (1.4,3.3)	4.7 (3.5,6.2)	34.0 (31.1,37.0)
**L**	**L**	**H**	**H**	**Res. Cor.**	44.0 (40.9,47.1)	10.2 (8.4,12.2)	9.4 (7.7,11.4)	11.3 (9.4,13.4)	8.4 (6.8,10.3)	97.4 (96.2,98.3)
				**Pairwise G^2**	11.4 (9.5,13.5)	2.9 (2.0,4.1)	1.8 (1.1,2.8)	3.4 (2.4,4.7)	2.7 (1.8,3.9)	93.4 (91.7,94.9)

				Tool	T_1&T_2	T_1&T_3	T_1&T_4	T_2&T_3	T_2&T_4	T_3&T_4
				**Pairwise χ^2**	11.3 (9.4,13.4)	3.1 (2.1,4.4)	2.3 (1.5,3.4)	3.9 (2.8,5.3)	2.9 (2.0,4.1)	93.1 (91.3,94.6)
**L**	**H**	**L**	**H**	**Res. Cor.**	0.5 (0.2,1.2)	35.3 (32.3,38.4)	30.1 (27.3,33.0)	0.3 (0.1,0.9)	0.0 (0.0,0.4)	61.7 (58.6,64.7)
				**Pairwise G^2**	0.1 (0.0,0.6)	5.6 (4.3,7.2)	9.4 (7.7,11.4)	0.1 (0.0,0.6)	0.0 (0.0,0.4)	32.8 (29.9,35.8)
				**Pairwise χ^2**	0.0 (0.0,0.4)	5.7 (4.3,7.3)	10.4 (8.6,12.5)	0.1 (0.0,0.6)	0.0 (0.0,0.4)	31.6 (28.7,34.6)
**Sensitivity: L L L L**										
**Specificity**				**Tool**	**T_1&T_2**	**T_1&T_3**	**T_1&T_4**	**T_2&T_3**	**T_2&T_4**	**T_3&T_4**
**H**	**H**	**L**	**L**	**Res. Cor.**	0.0 (0.0,0.4)	0.5 (0.1,1.2)	0.8 (0.3,1.7)	1.1 (0.5,2.0)	0.8 (0.3,1.7)	73.1 (70.0,76.1)
				**Pairwise G^2**	0.0 (0.0,0.4)	0.0 (0.0,0.4)	0.2 (0.0,0.8)	0.0 (0.0,0.4)	0.2 (0.0,0.8)	7.5 (5.8,9.5)
				**Pairwise χ^2**	0.0 (0.0,0.4)	0.0 (0.0,0.4)	0.2 (0.0,0.8)	0.0 (0.0,0.4)	0.2 (0.0,0.8)	7.5 (5.8,9.5)
**H**	**H**	**H**	**L**	**Res. Cor.**	0.0 (0.0,0.4)	0.0 (0.0,0.4)	2.1 (1.3,3.2)	0.1 (0.0,0.6)	1.9 (1.1,3.0)	81.6 (79.1,84.0)
				**Pairwise G^2**	0.0 (0.0,0.4)	0.0 (0.0,0.4)	0.2 (0.0,0.7)	0.0 (0.0,0.4)	0.4 (0.1,1.0)	35.7 (32.7,38.8)
				**Pairwise χ^2**	0.0 (0.0,0.4)	0.0 (0.0,0.4)	0.2 (0.0,0.7)	0.0 (0.0,0.4)	0.4 (0.1,1.0)	35.7 (32.7,38.8)
**H**	**H**	**H**	**H**	**Res. Cor.**	0.0 (0.0,0.4)	0.4 (0.1,1.0)	0.6 (0.2,1.3)	0.1 (0.0,0.6)	0.1 (0.0,0.6)	100.0 (99.6,100.0)
				**Pairwise G^2**	0.0 (0.0,0.4)	0.0 (0.0,0.4)	0.0 (0.0,0.4)	0.0 (0.0,0.4)	0.0 (0.0,0.4)	99.7 (99.1,99.9)
				**Pairwise χ^2**	0.0 (0.0,0.4)	0.0 (0.0,0.4)	0.0 (0.0,0.4)	0.0 (0.0,0.4)	0.0 (0.0,0.4)	99.7 (99.1,99.9)
**L**	**L**	**L**	**L**	**Res. Cor.**	0.0 (0.0,1.1)	17.3 (13.5,21.8)	17.6 (13.8,22.1)	21.7 (17.4,26.4)	17.9 (14.0,22.4)	69.4 (64.2,74.2)
				**Pairwise G^2**	0.0 (0.0,1.1)	3.2 (1.6,55.6)	1.7 (0.6,3.7)	2.9 (1.4,5.3)	2.6 (1.2,4.9)	6.9 (4.5,10.1)
				**Pairwise χ^2**	0.0 (0.0,1.1)	3.2 (1.6,55.6)	1.7 (0.6,3.7)	2.9 (1.4,5.3)	2.6 (1.2,4.9)	6.9 (4.5,10.1)
**L**	**L**	**H**	**L**	**Res. Cor.**	0.0 (0.0,0.4)	79.5 (76.8,82.0)	37.0 (34.0,40.1)	78.7 (76.0,81.2)	38.3 (35.3,41.4)	93.3 (91.6,94.8)
				**Pairwise G^2**	0.0 (0.0,0.4)	50.2 (47.0,53.3)	8.6 (6.9,10.5)	50.6 (47.4,53.7)	8.5 (6.8,10.4)	61.3 (58.2,64.3)
				**Pairwise χ^2**	0.0 (0.0,0.4)	50.6 (47.4,53.7)	8.6 (6.9,10.5)	51.1 (47.9,54.2)	8.7 (7.0,10.6)	61.4 (58.3,64.4)
**L**	**L**	**H**	**H**	**Res. Cor.**	36.0 (33.0,39.1)	92.8 (91.0,94.3)	92.9 (91.1,94.4)	94.8 (93.3,96.1)	94.5 (92.9,95.9)	100.0 (99.6,100.0)
				**Pairwise G^2**	31.5 (28.6,34.5)	74.1 (71.2,76.8)	74.3 (71.4,77.0)	74.2 (71.3,76.9)	74.6 (71.8,77.3)	100.0 (99.6,100.0)
				**Pairwise χ^2**	31.4 (28.5,34.4)	74.4 (71.6,77.1)	74.7 (71.9,77.4)	74.5 (71.7,77.2)	74.9 (72.1,77.6)	100.0 (99.6,100.0)
**L**	**H**	**L**	**H**	**Res. Cor.**	0.0 (0.0,0.4)	54.3 (51.1,57.4)	98.4 (97.4,99.1)	0.4 (0.1,1.0)	0.0 (0.0,0.4)	84.6 (82.2,86.8)
				**Pairwise G^2**	0.0 (0.0,0.4)	12.5 (10.5,14.7)	84.8 (82.4,87.0)	0.2 (0.0,0.7)	0.0 (0.0,0.4)	43.8 (40.7,47.0)
				**Pairwise χ^2**	0.0 (0.0,0.4)	12.7 (10.7,14.9)	84.8 (82.4,87.0)	0.2 (0.0,0.7)	0.0 (0.0,0.4)	43.8 (40.7,47.0)
**Sensitivity: L L H L**										
**Specificity**				**Tool**	**T_1&T_2**	**T_1&T_3**	**T_1&T_4**	**T_2&T_3**	**T_2&T_4**	**T_3&T_4**
**H**	**H**	**L**	**L**	**Res. Cor.**	0.0 (0.0,0.4)	5.9 (4.5,7.6)	2.0 (1.2,3.1)	5.3 (4.0,6.9)	2.6 (1.7,3.8)	78.8 (76.1,81.3)
				**Pairwise G^2**	0.0 (0.0,0.4)	1.3 (0.7,2.2)	0.2 (0.0,0.7)	0.7 (0.3,1.4)	0.3 (0.1,0.9)	26.3 (23.6,29.2)
				**Pairwise χ^2**	0.0 (0.0,0.4)	1.5 (0.8,2.5)	0.2 (0.0,0.7)	0.7 (0.3,1.4)	0.3 (0.1,0.9)	26.3 (23.6,29.2)
**H**	**H**	**H**	**L**	**Res. Cor.**	0.0 (0.0,0.4)	0.4 (0.1,1.0)	3.5 (2.5,4.8)	0.5 (0.2,1.2)	2.2 (1.4,3.3)	93.8 (92.1,95.2)
				**Pairwise G^2**	0.0 (0.0,0.4)	0.0 (0.0,0.4)	0.9 (0.4,1.7)	0.0 (0.0,0.4)	0.4 (0.1,1.0)	70.5 (67.5,73.3)
				**Pairwise χ^2**	0.0 (0.0,0.4)	0.0 (0.0,0.4)	0.9 (0.4,1.7)	0.0 (0.0,0.4)	0.4 (0.1,1.0)	70.6 (67.9,73.4)
**H**	**H**	**H**	**H**	**Res. Cor.**	0.0 (0.0,0.4)	1.0 (0.5,1.8)	0.0 (0.0,0.4)	0.6 (0.2,1.3)	0.5 (0.2,1.2)	100.0 (99.6,100.0)
				**Pairwise G^2**	0.0 (0.0,0.4)	0.1 (0.0,0.6)	0.0 (0.0,0.4)	0.0 (0.0,0.4)	0.2 (0.0,0.7)	100.0 (99.6,100.0)
				**Pairwise χ^2**	0.0 (0.0,0.4)	0.1 (0.0,0.6)	0.0 (0.0,0.4)	0.0 (0.0,0.4)	0.2 (0.0,0.7)	100.0 (99.6,100.0)
**L**	**L**	**L**	**L**	**Res. Cor.**	0.0 (0.0,0.4)	66.0 (62.9,69.0)	37.7 (34.6,40.9)	65.4 (62.3,68.4)	33.9 (30.9,36.9)	85.4 (83.0,87.6)
				**Pairwise G^2**	0.0 (0.0,0.4)	34.3 (31.3,37.4)	9.5 (7.7,11.5)	33.2 (30.3,36.3)	9.0 (7.2,10.9)	43.6 (40.5,46.9)
				**Pairwise χ^2**	0.0 (0.0,0.4)	34.4 (31.4,37.5)	9.5 (7.7,11.5)	33.4 (30.5,36.5)	9.0 (7.2,10.9)	43.6 (40.5,46.9)

L	L	H	L	Res. Cor.	0.5 (0.2,1.2)	98.7 (97.8,99.3)	49.9 (46.8,53.1)	99.1 (98.3,99.6)	53.7 (50.5,56.8)	98.5 (97.5,99.1)
				Pairwise G^2	0.4 (0.1,1.0)	94.6 (93.0,96.0)	14.4 (12.2,16.7)	94.3 (92.7,95.7)	16.3 (14.0,18.7)	89.1 (87.0,91.0)
				Pairwise χ^2	0.4 (0.1,1.0)	94.8 (93.3,96.1)	14.6 (12.4,16.9)	94.5 (92.9,95.9)	16.3 (14.0,18.7)	89.1 (87.0,91.0)
L	L	H	H	Res. Cor.	95.3 (93.8,96.6)	97.2 (95.9,98.1)	95.8 (94.3,97.0)	96.8 (95.5,97.9)	95.9 (94.4,97.1)	97.2 (95.9,98.1)
				Pairwise G^2	80.8 (78.1,83.3)	95.7 (94.1,96.9)	81.7 (79.1,84.2)	95.7 (94.1,96.9)	82.3 (79.7,84.7)	97.2 (95.9,98.1)
				Pairwise χ^2	80.7 (77.9,83.2)	95.7 (94.1,96.9)	81.7 (79.1,84.2)	95.8 (94.3,97.0)	82.7 (80.1,85.1)	97.2 (95.9,98.1)
L	H	L	H	Res. Cor.	0.2 (0.0,0.7)	92.9 (91.1,94.4)	97.6 (96.4,98.5)	1.3 (0.7,2.2)	0.0 (0.0,0.4)	100.0 (99.6,100.0)
				Pairwise G^2	0.1 (0.0,0.6)	62.6 (59.5,65.6)	84.7 (82.3,86.9)	0.2 (0.0,0.7)	0.0 (0.0,0.4)	99.8 (99.3,100.0)
				Pairwise χ^2	0.1 (0.0,0.6)	63.1 (60.0,66.1)	85.1 (82.7,87.3)	0.2 (0.0,0.7)	0.0 (0.0,0.4)	99.8 (99.3,100.0)
**Sensitivity: L L H H**										
**Specificity**				**Tool**	**T_1&T_2**	**T_1&T_3**	**T_1&T_4**	**T_2&T_3**	**T_2&T_4**	**T_3&T_4**
H	H	L	L	Res. Cor.	0.0 (0.0,0.4)	5.6 (4.3,7.2)	5.1 (3.8,6.7)	7.6 (6.0,9.4)	6.7 (5.2,8.4)	99.6 (99.0,99.9)
				Pairwise G^2	0.0 (0.0,0.4)	1.3 (0.7,2.2)	1.1 (0.6,2.0)	1.0 (0.5,1.8)	1.6 (0.9,2.6)	92.3 (90.5,93.9)
				Pairwise χ^2	0.0 (0.0,0.4)	1.4 (0.8,2.3)	1.2 (0.6,2.1)	1.0 (0.5,1.8)	1.8 (1.1,2.8)	92.2 (90.4,93.8)
H	H	H	L	Res. Cor.	0.0 (0.0,0.4)	0.5 (0.2,1.2)	8.4 (6.8,10.3)	0.6 (0.2,1.3)	6.4 (5.0,8.1)	100.0 (99.6,100.0)
				Pairwise G^2	0.0 (0.0,0.4)	0.1 (0.0,0.6)	1.5 (0.8,2.5)	0.2 (0.0,0.7)	1.4 (0.8,2.3)	100.0 (99.6,100.0)
				Pairwise χ^2	0.0 (0.0,0.4)	0.1 (0.0,0.6)	1.7 (1.0,2.7)	0.2 (0.0,0.7)	1.6 (0.9,2.6)	100.0 (99.6,100.0)
H	H	H	H	Res. Cor.	0.1 (0.0,0.6)	1.7 (1.0,2.7)	1.2 (0.6,2.1)	1.4 (0.8,2.3)	1.4 (0.8,2.3)	100.0 (99.6,100.0)
				Pairwise G^2	0.0 (0.0,0.4)	0.1 (0.0,0.6)	0.4 (0.1,1.0)	0.4 (0.1,1.0)	0.3 (0.1,0.9)	100.0 (99.6,100.0)
				Pairwise χ^2	0.0 (0.0,0.4)	0.1 (0.0,0.6)	0.4 (0.1,1.0)	0.4 (0.1,1.0)	0.4 (0.1,1.0)	100.0 (99.6,100.0)
L	L	L	L	Res. Cor.	21.1 (18.5,23.7)	87.0 (84.8,89.1)	87.8 (85.5,89.7)	88.0 (85.8,89.9)	88.0 (85.8,89.9)	100.0 (99.6,100.0)
				Pairwise G^2	12.7 (10.6,14.9)	55.6 (52.4,58.7)	60.4 (57.3,63.5)	58.9 (55.8,62.0)	59.1 (56.0,62.2)	98.5 (97.5,99.1)
				Pairwise χ^2	12.7 (10.6,14.9)	55.8 (52.6,58.9)	60.6 (57.5,63.7)	59.2 (56.1,62.3)	59.2 (56.1,62.3)	98.4 (97.4,99.1)
L	L	H	L	Res. Cor.	85.1 (82.7,87.2)	100.0 (99.6,100.0)	92.9 (91.1,94.4)	99.7 (99.1,99.9)	94.9 (93.3,96.2)	100.0 (99.6,100.0)
				Pairwise G^2	62.0 (58.9,65.0)	98.4 (97.4,99.1)	69.3 (66.4,72.2)	97.6 (96.4,98.5)	70.6 (67.7,73.5)	100.0 (99.6,100.0)
				Pairwise χ^2	61.9 (58.8,64.9)	98.4 (97.4,99.1)	69.6 (66.7,72.5)	97.9 (96.8,98.7)	70.6 (67.7,73.5)	100.0 (99.6,100.0)
L	L	H	H	Res. Cor.	100.0 (99.5,100.0)	2.6 (1.6,4.1)	2.6 (1.6,4.1)	3.1 (1.9,4.6)	2.6 (1.6,4.1)	1.9 (1.1,3.2)
				Pairwise G^2	99.7 (99.0,100.0)	2.1 (1.2,3.4)	2.4 (1.4,3.7)	2.1 (1.2,3.4)	1.9 (1.1,3.2)	1.9 (1.1,3.2)
				Pairwise χ^2	99.7 (99.0,100.0)	2.1 (1.2,3.4)	2.4 (1.4,3.7)	2.1 (1.2,3.4)	1.9 (1.1,3.2)	1.9 (1.1,3.2)
L	H	L	H	Res. Cor.	3.4 (2.4,4.7)	94.4 (92.8,95.7)	100.0 (99.6,100.0)	5.1 (3.8,6.7)	0.0 (0.0,0.4)	100.0 (99.6,100.0)
				Pairwise G^2	1.0 (0.5,1.8)	65.4 (62.4,68.3)	99.8 (99.3,100.0)	0.9 (0.4,1.7)	0.0 (0.0,0.4)	100.0 (99.6,100.0)
				Pairwise χ^2	0.9 (0.4,1.7)	65.4 (62.4,68.3)	99.8 (99.3,100.0)	1.0 (0.5,1.8)	0.0 (0.0,0.4)	100.0 (99.6,100.0)

Table ix.4 Mean absolute biases (x10^{-2}) of posterior medians of each parameter obtained from the conditional independence model

**Sensitivity: H H L L**												
**Specificity**				π	Se_1	Se_2	Se_3	Se_4	Sp_1	Sp_2	Sp_3	Sp_4
H	H	L	L	0.0 (-0.1,0.0)	5.2 (5.1,5.4)	5.3 (5.1,5.5)	-0.5 (-0.7,-0.3)	-0.4 (-0.6,-0.3)	1.1 (1.1,1.2)	1.1 (1.1,1.2)	-0.1 (-0.2,-0.1)	-0.1 (-0.2,-0.1)
H	H	H	L	-1.4 (-1.5,-1.4)	8.1 (8.0,8.2)	8.2 (8.1,8.2)	-0.3 (-0.5,-0.1)	-0.2 (-0.3,0.0)	0.4 (0.4,0.5)	0.4 (0.4,0.5)	-1.0 (-1.0,-0.9)	-0.4 (-0.5,-0.3)

**H**	**H**	**H**	**H**	-1.6 (-1.7,-1.5)	8.6 (8.5,8.6)	8.6 (8.5,8.6)	-0.3 (-0.5,-0.1)	-0.3 (-0.5,-0.1)	0.2 (0.2,0.3)	0.3 (0.3,0.4)	-1.1 (-1.2,-1.1)	-1.1 (-1.2,-1.1)
**L**	**L**	**L**	**L**	3.6 (3.4,3.9)	2.5 (2.2,2.8)	2.9 (2.6,3.1)	-2.4 (-2.7,-2.1)	-2.2 (-2.5,-1.9)	3.0 (2.8,3.2)	3.2 (3.0,3.3)	0.4 (0.2,0.5)	0.3 (0.2,0.4)
**L**	**L**	**H**	**L**	-0.4 (-0.5,-0.3)	7.6 (7.6,7.7)	7.7 (7.6,7.7)	-3.1 (-3.3,-2.8)	-0.7 (-0.9,-0.5)	1.6 (1.5,1.7)	1.7 (1.6,1.8)	-1.0 (-1.1,-1.0)	-0.3 (-0.4,-0.3)
**L**	**L**	**H**	**H**	-1.5 (-1.6,-1.4)	8.5 (8.4,8.5)	8.5 (8.4,8.6)	-1.0 (-1.2,-0.7)	-0.9 (-1.1,-0.6)	1.0 (0.9,1.1)	0.9 (0.8,1.0)	-1.2 (-1.3,-1.2)	-1.2 (-1.2,-1.1)
**L**	**H**	**L**	**H**	-1.2 (-1.2,-1.1)	8.2 (8.1,8.2)	7.5 (7.4,7.5)	-0.3 (-0.5,-0.1)	-0.8 (-1.0,-0.6)	1.2 (1.1,1.3)	0.5 (0.4,0.6)	-0.4 (-0.4,-0.3)	-1.0 (-1.0,-0.9)
**Sensitivity: H H H L**												
**Specificity**				π	Se_1	Se_2	Se_3	Se_4	Sp_1	Sp_2	Sp_3	Sp_4
**H**	**H**	**L**	**L**	-1.1 (-1.2,-1.1)	7.5 (7.4,7.5)	7.3 (7.2,7.4)	-0.4 (-0.5,-0.3)	-0.3 (-0.5,-0.2)	0.6 (0.5,0.6)	0.5 (0.5,0.6)	-0.8 (-0.9,-0.7)	-0.3 (-0.4,-0.2)
**H**	**H**	**H**	**L**	-1.7 (-1.8,-1.7)	8.8 (8.7,8.8)	8.7 (8.7,8.8)	-0.4 (-0.5,-0.3)	0.0 (-0.2,0.2)	0.2 (0.2,0.3)	0.2 (0.2,0.3)	-1.8 (-1.8,-1.7)	-0.5 (-0.6,-0.4)
**H**	**H**	**H**	**H**	-1.7 (-1.8,-1.7)	8.9 (8.8,8.9)	8.9 (8.8,8.9)	-0.3 (-0.4,-0.2)	0.0 (-0.2,0.1)	0.2 (0.1,0.2)	0.2 (0.2,0.3)	-1.8 (-1.9,-1.8)	-1.2 (-1.2,-1.1)
**L**	**L**	**L**	**L**	0.5 (0.4,0.6)	6.2 (6.1,6.4)	6.3 (6.2,6.5)	-3.5 (-3.7,-3.3)	-1.0 (-1.2,-0.8)	1.9 (1.8,2.0)	1.8 (1.7,1.9)	-0.6 (-0.7,-0.5)	-0.2 (-0.2,-0.1)
**L**	**L**	**H**	**L**	-1.1 (-1.2,-1.0)	8.7 (8.6,8.7)	8.8 (8.7,8.8)	-3.6 (-3.9,-3.3)	-0.3 (-0.5,-0.1)	1.3 (1.2,1.3)	1.3 (1.2,1.4)	-2.0 (-2.1,-2.0)	-0.4 (-0.5,-0.3)
**L**	**L**	**H**	**H**	-2.1 (-2.2,-2.0)	8.9 (8.9,9.0)	8.9 (8.9,8.9)	0.0 (-0.2,0.2)	0.4 (0.2,0.6)	0.7 (0.6,0.7)	0.6 (0.5,0.7)	-2.1 (-2.2,-2.1)	-1.3 (-1.4,-1.3)
**L**	**H**	**L**	**H**	-1.5 (-1.5,-1.4)	8.5 (8.5,8.6)	8.0 (7.9,8.0)	-0.4 (-0.5,-03)	-0.8 (-1.0,-0.6)	1.0 (0.9,1.1)	0.3 (0.3,0.4)	-0.9 (-1.0,-0.9)	-1.1 (-1.1,-1.0)
**Sensitivity: H H H H**												
**Specificity**				π	Se_1	Se_2	Se_3	Se_4	Sp_1	Sp_2	Sp_3	Sp_4
**H**	**H**	**L**	**L**	-1.4 (-1.5,-1.3)	8.1 (8.0,8.1)	8.1 (8.0,8.1)	-0.3 (-0.4,-0.2)	-0.4 (-0.5,-0.3)	0.4 (0.3,0.4)	0.4 (0.3,0.4)	-1.0 (-1.0,-0.9)	-1.0 (-1.0,-0.9)
**H**	**H**	**H**	**L**	-1.8 (-1.8,-1.7)	8.8 (8.8,8.9)	8.8 (8.8,8.9)	-0.3 (-0.4,-0.2)	-0.3 (-0.4,-0.2)	0.2 (0.1,0.2)	0.2 (0.1,0.2)	-1.9 (-1.9,-1.8)	-1.2 (-1.2,-1.1)
**H**	**H**	**H**	**H**	-1.8 (-1.9,-1.8)	8.9 (8.9,8.9)	8.9 (8.9,9.0)	-0.1 (-0.2,0.0)	-0.2 (-0.3,-0.1)	0.2 (0.1,0.2)	0.1 (0.1,0.2)	-1.9 (-1.9,-1.8)	-1.9 (-1.9,-1.8)
**L**	**L**	**L**	**L**	-0.9 (-1.0,-0.8)	7.5 (7.5,7.6)	7.6 (7.5,7.7)	-1.1 (-1.3,-0.9)	-1.1 (-1.3,-0.9)	1.2 (1.1,1.3)	1.1 (1.0,1.2)	-0.9 (-1.0,-0.8)	-0.8 (-0.9,-0.7)
**L**	**L**	**H**	**L**	-1.9 (-2.0,-1.8)	8.8 (8.8,8.9)	8.8 (8.8,8.9)	-0.8 (-1.0,-0.6)	0.4 (0.3,0.6)	0.8 (0.7,0.9)	0.7 (0.7,0.8)	-2.1 (-2.2,-2.1)	-1.1 (-1.1,-1.0)
**L**	**L**	**H**	**H**	-2.3 (-2.4,-2.3)	9.0 (8.9,9.0)	9.0 (9.0,9.0)	0.9 (0.7,1.0)	0.9 (0.8,1.0)	0.5 (0.4,0.6)	0.4 (0.4,0.5)	-2.1 (-2.2,-2.1)	-2.1 (-2.1,-2.0)
**L**	**H**	**L**	**H**	-1.7 (-1.8,-1.7)	8.8 (8.8,8.9)	8.8 (8.7,8.8)	-0.2 (-0.3,-0.1)	-0.3 (-0.5,-0.2)	0.8 (0.7,0.9)	0.1 (0.0,0.2)	-1.2 (-1.3,-1.1)	-1.8 (-1.9,-1.8)
**Sensitivity: L L L L**												
**Specificity**				π	Se_1	Se_2	Se_3	Se_4	Sp_1	Sp_2	Sp_3	Sp_4
**H**	**H**	**L**	**L**	-6.3 (-6.4,-6.2)	33.1 (32.8,33.3)	33.2 (32.9,33.4)	-0.9 (-1.1,-0.6)	-0.8 (-1.0,-0.6)	1.4 (1.3,1.4)	1.4 (1.3,1.4)	-1.5 (-1.6,-1.5)	-1.6 (-1.7,-1.5)
**H**	**H**	**H**	**L**	-7.7 (-7.8,-7.7)	37.3 (37.2,37.4)	37.4 (37.3,37.5)	-0.9 (-1.2,-0.7)	-0.4 (-0.6,-0.2)	0.7 (0.6,0.7)	0.7 (0.7,0.8)	-4.6 (-4.7,-4.6)	-1.8 (-1.9,-1.8)
**H**	**H**	**H**	**H**	-7.9 (-8.0,-7.9)	37.5 (37.5,37.6)	37.6 (37.5,37.7)	-0.4 (-0.6,-0.2)	-0.5 (-0.7,-0.3)	0.6 (0.6,0.7)	0.6 (0.6,0.7)	-4.6 (-4.7,-4.6)	4.6 (-4.7,-4.6)
**L**	**L**	**L**	**L**	-1.9 (-2.2,-1.7)	31.8 (31.5,32.2)	31.6 (31.2,32.0)	-5.2 (-5.7,-4.8)	-4.9 (-5.4,-4.5)	6.3 (6.1,6.5)	6.2 (6.1,6.4)	-1.5 (-1.6,-1.3)	-1.5 (-1.6,-1.4)
**L**	**L**	**H**	**L**	-5.8 (-5.9,-5.7)	37.9 (37.8,37.9)	37.9 (37.8,37.9)	-11.4 (-11.8,-11.1)	-2.7 (-3.0,-2.4)	4.8 (4.7,4.9)	4.8 (4.7,4.9)	-5.4 (-5.5,-5.4)	-1.9 (-2.0,-1.8)
**L**	**L**	**H**	**H**	-8.1 (-8.2,-8.0)	38.6 (38.6,38.6)	38.6 (38.6,38.6)	-3.7 (-4.1,-3.3)	-3.6 (-4.0,-3.2)	3.3 (3.2,3.4)	3.3 (3.2,3.4)	-5.2 (-5.3,-5.1)	-5.2 (-5.3,-5.1)
**L**	**H**	**L**	**H**	-7.5 (-7.6,-7.5)	37.1 (37.0,37.2)	38.0 (37.9,38.0)	-0.7 (-0.9,-0.5)	-2.8 (-3.0,-2.5)	3.6 (3.5,3.6)	1.0 (0.9,1.1)	-1.8 (-1.9,-1.8)	-4.7 (-4.8,-4.6)
**Sensitivity: L L H L**												
**Specificity**				π	Se_1	Se_2	Se_3	Se_4	Sp_1	Sp_2	Sp_3	Sp_4
**H**	**H**	**L**	**L**	-7.5 (-7.6,-7.5)	36.5 (36.3,36.6)	36.3 (36.2,36.4)	-0.9 (-1.0,-0.7)	-0.4 (-0.6,-0.2)	0.8 (0.8,0.9)	0.8 (0.8,0.9)	-4.4 (-4.5,-4.3)	-1.7 (-1.8,-1.7)
**H**	**H**	**H**	**L**	-8.1 (-8.1,-8.0)	38.3 (38.3,38.4)	38.3 (38.3,38.4)	-0.8 (-1.0,-0.7)	-0.1 (-0.3,0.1)	0.6 (0.5,0.6)	0.5 (0.5,0.6)	-7.5 (-7.5,-7.4)	-1.9 (-1.9,-1.8)
**H**	**H**	**H**	**H**	-8.0 (-8.1,-8.0)	37.8 (37.7,37.9)	37.7 (37.7,37.8)	-0.4 (-0.5,-0.2)	0.1 (-0.1,0.3)	0.6 (0.5,0.6)	0.6 (0.5,0.6)	-7.4 (-7.5,-7.3)	-4.6 (-4.7,-4.5)
**L**	**L**	**L**	**L**	-5.2 (-5.3,-5.1)	36.6 (36.5,36.7)	36.6 (36.5,36.7)	-12.0 (-12.3,-11.7)	-3.2 (-3.5,-2.9)	5.0 (4.9,5.1)	5.0 (4.9,5.1)	-5.2 (-5.3,-5.1)	-1.9 (-1.9,-1.8)

**L**	**L**	**H**	**L**	-6.8 (-6.9,-6.7)	38.9 (38.9,39.0)	38.9 (38.9,39.0)	-16.0 (-16.4,-15.6)	-0.7 (-0.9,-0.4)	4.3 (4.2,4.4)	4.3 (4.3,4.4)	-8.8 (-8.8,-8.7)	-1.7 (-1.8,-1.6)
**L**	**L**	**H**	**H**	-9.2 (-9.3,-9.1)	37.8 (37.4,38.2)	37.8 (37.4,38.2)	0.9 (0.6,1.1)	3.4 (3.1,3.7)	2.3 (2.2,2.4)	2.3 (2.2,2.4)	-8.2 (-8.3,-8.1)	-4.8 (-4.9,-4.7)
**L**	**H**	**L**	**H**	-7.9 (-7.9,-7.8)	37.3 (37.2,37.4)	38.3 (38.3,38.3)	-1.0 (-1.2,-0.8)	-1.4 (-1.6,-1.1)	3.3 (3.2,3.4)	0.7 (0.7,0.8)	-4.6 (-4.7,-4.5)	-4.7 (-4.8,-4.7)
**Sensitivity: L L H H**												
**Specificity**				π	Se_1	Se_2	Se_3	Se_4	Sp_1	Sp_2	Sp_3	Sp_4
**H**	**H**	**L**	**L**	-7.8 (-7.8,-7.7)	37.2 (37.1,37.3)	37.1 (37.0,37.2)	-0.6 (-0.8,-0.5)	-0.7 (-0.8,-0.5)	0.7 (0.7,0.8)	0.7 (0.7,0.8)	-4.5 (-4.6,-4.4)	-4.5 (-4.6,-4.4)
**H**	**H**	**H**	**L**	-8.1 (-8.1,-8.0)	37.9 (37.8,38.0)	38.0 (37.9,38.0)	-0.6 (-0.7,-0.4)	0.0 (-0.2,0.1)	0.5 (0.5,0.6)	0.5 (0.5,0.6)	-7.4 (-7.5,-7.3)	-4.5 (-4.6,-4.5)
**H**	**H**	**H**	**H**	-7.9 (-8.0,-7.9)	37.1 (37.0,37.2)	37.1 (37.0,37.2)	0.0 (-0.2,0.1)	0.0 (-0.1,0.2)	0.5 (0.5,0.6)	0.5 (0.5,0.6)	-7.3 (-7.3,-7.2)	-7.2 (-7.3,-7.1)
**L**	**L**	**L**	**L**	-7.3 (-7.4,-7.2)	37.9 (37.9,38.0)	38.0 (37.9,38.0)	-4.4 (-4.7,-4.1)	-4.3 (-4.5,-4.0)	3.8 (3.7,3.9)	3.8 (3.7,3.9)	-4.9 (-5.0,-4.8)	-4.9 (-5.0,-4.8)
**L**	**L**	**H**	**L**	-8.9 (-9.0,-8.9)	38.9 (38.8,39.0)	38.9 (38.9,39.0)	-2.8 (-3.1,-2.5)	3.1 (2.9,3.3)	2.9 (2.8,2.9)	2.8 (2.8,2.9)	-8.5 (-8.5,-8.4)	-4.7 (-4.8,-4.6)
**L**	**L**	**H**	**H**	-0.1 (-0.3,0.1)	2.3 (1.9,2.8)	2.4 (1.9,2.8)	-0.2 (-0.5,0.1)	-0.2 (-0.5,0.1)	0.4 (0.4,0.5)	0.4 (0.4,0.5)	-0.2 (-0.4,-0.1)	-0.2 (-0.3,-0.1)
**L**	**H**	**L**	**H**	-8.3 (-8.4,-8.2)	37.7 (37.6,37.8)	39.0 (39.0,39.0)	0.3 (0.2,0.5)	-0.9 (-1.1,-0.7)	3.0 (3.0,3.1)	0.4 (0.4,0.5)	-4.7 (-4.8,-4.5)	-7.6 (-7.7,-7.6)

Table ix.5 Coverages of the 95% CrIs for each model parameter

**Sensitivity: H H L L**												
**Specificity**				π	Se_1	Se_2	Se_3	Se_4	Sp_1	Sp_2	Sp_3	Sp_4
**H**	**H**	**L**	**L**	98.9 (98.3,99.6)	83.3 (80.9,85.7)	81.0 (78.5,83.6)	94.4 (92.9,95.8)	95.8 (94.6,97.1)	85.3 (83.6,88.1)	85.1 (82.8,87.4)	95.2 (93.8,96.6)	96.3 (95.1,97.5)
**H**	**H**	**H**	**L**	73.1 (70.4,75.8)	2.5 (1.5,3.5)	2.3 (1.4,3.2)	94.4 (93.0,95.8)	94.5 (93.1,95.9)	90.4 (8.6,92.2)	93.8 (92.3,95.3)	76.3 (73.7,78.9)	93.4 (91.9,94.9)
**H**	**H**	**H**	**H**	61.4 (58.4,64.4)	0.1 (0.0,0.3)	0.0 (0.0,0.0)	94.5 (93.1,95.9)	94.7 (93.3,96.1)	94.8 (93.4,96.2)	94.2 (92.8,95.6)	69.6 (66.7,72.5)	69.3 (66.4,72.2)
**L**	**L**	**L**	**L**	89.9 (87.5,92.3)	99.3 (98.7,100.0)	99.5 (98.9,100.0)	88.9 (86.4,91.4)	91.6 (89.4,93.8)	72.8 (69.2,76.3)	70.6 (66.9,74.2)	94.6 (92.8,96.4)	94.8 (93.0,96.6)
**L**	**L**	**H**	**L**	98.1 (97.2,98.9)	14.3 (12.2,16.5)	14.6 (12.4,16.9)	89.8 (87.9,91.7)	93.5 (91.9,95.0)	83.5 (81.2,85.8)	85.2 (83.0,87.5)	82.2 (79.8,84.6)	94.5 (93.1,95.9)
**L**	**L**	**H**	**H**	83.0 (80.7,85.3)	0.5 (0.1,0.9)	1.1 (0.5,1.7)	94.4 (93.0,95.8)	93.9 (82.4,95.4)	91.1 (89.3,92.9)	89.6 (87.7,91.5)	71.6 (68.8,74.4)	72.7 (69.9,75.5)
**L**	**H**	**L**	**H**	88.4 (86.4,90.4)	1.0 (0.4,1.6)	26.3 (23.6,29.1)	95.8 (94.6,97.0)	94.2 (92.7,95.6)	85.4 (83.2,87.6)	93.0 (91.4,94.6)	93.2 (91.6,94.8)	79.4 (76.9,81.9)
**Sensitivity: H H H L**												
**Specificity**				π	Se_1	Se_2	Se_3	Se_4	Sp_1	Sp_2	Sp_3	Sp_4
**H**	**H**	**L**	**L**	85.1 (82.9,87.3)	10.0 (8.1,11.9)	13.8 (11.7,15.9)	94.1 (92.6,95.6)	94.8 (93.4,96.2)	92.4 (90.8,94.0)	93.8 (92.3,95.3)	89.7 (87.8,91.6)	94.5 (93.1,95.9)
**H**	**H**	**H**	**L**	57.9 (54.8,61.0)	0.0 (0.0,0.0)	0.0 (0.0,0.0)	93.7 (92.2,95.2)	94.4 (93.0,95.8)	94.6 (93.2,96.0)	94.0 (92.5,95.5)	43.0 (39.9,46.1)	94.3 (92.9,95.7)
**H**	**H**	**H**	**H**	53.5 (50.4,56.6)	0.0 (0.0,0.0)	0.0 (0.0,0.0)	92.9 (91.3,94.5)	94.9 (93.5,96.3)	94.2 (92.8,95.6)	92.7 (91.1,94.3)	37.2 (34.2,40.2)	67.5 (64.6,70.4)
**L**	**L**	**L**	**L**	98.0 (97.1,98.9)	63.4 (60.4,66.5)	59.1 (56.0,62.2)	88.2 (86.1,90.2)	94.8 (93.4,96.2)	84.0 (81.7,86.3)	83.1 (80.7,85.4)	95.6 (94.3,96.9)	95.5 (94.2,96.8)
**L**	**L**	**H**	**L**	91.3 (89.6,93.1)	0.3 (0.0,0.6)	0.2 (0.0,0.5)	90.7 (88.9,92.5)	93.2 (91.6,94.7)	86.4 (84.3,88.5)	87.7 (85.7,89.8)	40.1 (37.1,43.2)	93.7 (92.1,95.2)
**L**	**L**	**H**	**H**	56.7 (53.6,59.8)	0.0 (0.0,0.0)	0.0 (0.0,0.0)	95.8 (94.6,97.0)	95.1 (93.8,96.4)	92.7 (91.1,94.3)	93.7 (92.2,95.2)	32.6 (29.7,35.5)	64.3 (61.3,67.3)
**L**	**H**	**L**	**H**	76.8 (74.2,79.4)	0.0 (0.0,0.0)	7.0 (5.4,8.6)	94.0 (92.5,95.5)	94.9 (93.5,96.3)	87.5 (85.5,89.5)	94.7 (93.3,96.1)	88.8 (86.8,90.8)	75.5 (72.8,78.2)
**Sensitivity: H H H H**												
**Specificity**				π	Se_1	Se_2	Se_3	Se_4	Sp_1	Sp_2	Sp_3	Sp_4
**H**	**H**	**L**	**L**	72.7 (69.9,75.5)	0.8 (0.2,1.4)	0.7 (0.2,1.2)	94.3 (92.9,95.7)	94.8 (93.4,96.2)	93.1 (91.5,94.7)	94.2 (92.8,95.6)	88.1 (86.1,90.1)	87.3 (85.2,89.4)
**H**	**H**	**H**	**L**	52.8 (49.7,55.9)	0.0 (0.0,0.0)	0.0 (0.0,0.0)	94.9 (93.5,96.3)	95.4 (94.1,96.7)	94.6 (93.2,96.0)	93.8 (92.3,95.3)	34.5 (31.6,37.4)	84.5 (82.3,86.7)
**H**	**H**	**H**	**H**	48.8 (45.7,51.9)	0.0 (0.0,0.0)	0.0 (0.0,0.0)	95.0 (93.6,96.4)	94.7 (93.3,96.1)	93.9 (92.4,95.4)	95.0 (93.6,96.4)	33.1 (30.2,36.0)	31.7 (28.8,34.6)

**L**	**L**	**L**	**L**	95.3 (94.0,96.6)	13.6 (11.5,15.8)	12.9 (10.9,15.0)	93.8 (92.3,95.3)	93.2 (91.6,94.7)	87.7 (85.6,89.7)	88.4 (86.4,90.4)	91.4 (89.6,93.1)	91.7 (90.0,93.4)
**L**	**L**	**H**	**L**	68.6 (65.7,71.5)	0.0 (0.0,0.0)	0.0 (0.0,0.0)	95.0 (93.6,96.4)	94.3 (92.9,95.7)	89.3 (87.4,91.2)	91.9 (90.2,93.6)	32.5 (29.6,35.4)	87.8 (85.8,89.8)
**L**	**L**	**H**	**H**	37.9 (34.9,40.9)	0.0 (0.0,0.0)	0.0 (0.0,0.0)	93.9 (92.4,95.4)	94.7 (93.3,96.1)	92.5 (90.9,94.1)	94.7 (93.3,96.1)	28.8 (26.0,31.6)	32.0 (29.1,34.9)
**L**	**H**	**L**	**H**	59.8 (56.8,62.8)	0.0 (0.0,0.0)	0.0 (0.0,0.0)	95.5 (94.2,96.8)	94.6 (93.2,96.0)	91.1 (89.3,92.9)	94.7 (93.3,96.1)	83.0 (80.7,85.3)	39.8 (36.8,42.8)
**Sensitivity: L L L L**												
**Specificity**				π	Se_1	Se_2	Se_3	Se_4	Sp_1	Sp_2	Sp_3	Sp_4
**H**	**H**	**L**	**L**	8.5 (6.6,10.3)	0.0 (0.0,0.0)	0.1 (0.0,0.3)	94.4 (92.8,95.9)	95.4 (94.0,96.8)	74.3 (71.4,77.2)	76.4 (73.6,79.3)	77.5 (74.7,80.3)	73.6 (70.6,76.6)
**H**	**H**	**H**	**L**	0.0 (0.0,0.0)	0.0 (0.0,0.0)	0.0 (0.0,0.0)	93.7 (92.2,95.2)	95.3 (94.0,96.6)	85.9 (83.7,88.1)	86.6 (84.5,88.7)	0.0 (0.0,0.0)	64.6 (61.6,67.6)
**H**	**H**	**H**	**H**	0.0 (0.0,0.0)	0.0 (0.0,0.0)	0.0 (0.0,0.0)	94.8 (93.4,96.2)	95.2 (93.9,96.5)	87.7 (85.7,89.7)	89.0 (87.1,90.9)	0.0 (0.0,0.0)	0.0 (0.0,0.0)
**L**	**L**	**L**	**L**	100.0 (100.0,100.0)	2.3 (0.7,3.9)	2.3 (0.7,3.9)	76.6 (72.1,81.1)	78.9 (74.6,83.2)	4.0 (2.0,6.1)	2.9 (1.1,4.7)	85.3 (81.5,89.0)	82.7 (78.7,86.6)
**L**	**L**	**H**	**L**	9.5 (7.7,11.3)	0.0 (0.0,0.0)	0.0 (0.0,0.0)	42.3 (39.2,45.3)	88.5 (86.5,90.5)	12.1 (10.1,14.2)	11.4 (9.4,13.4)	0.0 (0.0,0.0)	67.3 (64.4,70.3)
**L**	**L**	**H**	**H**	0.1 (0.0,0.3)	0.0 (0.0,0.0)	0.0 (0.0,0.0)	87.8 (85.7,89.8)	86.4 (84.3,88.6)	35.2 (32.2,38.2)	35.2 (32.2,38.2)	0.0 (0.0,0.0)	0.0 (0.0,0.0)
**L**	**H**	**L**	**H**	0.0 (0.0,0.0)	0.0 (0.0,0.0)	0.0 (0.0,0.0)	94.2 (92.7,95.6)	90.5 (88.7,92.3)	18.4 (16.0,20.8)	83.8 (81.5,86.1)	67.0 (64.1,69.9)	0.0 (0.0,0.0)
**Sensitivity: L L H L**												
**Specificity**				π	Se_1	Se_2	Se_3	Se_4	Sp_1	Sp_2	Sp_3	Sp_4
**H**	**H**	**L**	**L**	0.0 (0.0,0.0)	0.0 (0.0,0.0)	0.0 (0.0,0.0)	94.5 (93.1,95.9)	94.2 (92.7,95.6)	85.3 (83.1,87.5)	85.2 (83.0,87.4)	4.4 (3.1,5.7)	67.4 (64.5,70.3)
**H**	**H**	**H**	**L**	0.0 (0.0,0.0)	0.0 (0.0,0.0)	0.0 (0.0,0.0)	95.2 (93.9,96.5)	93.8 (92.3,95.3)	88.7 (86.7,90.7)	89.9 (88.0,91.8)	0.0 (0.0,0.0)	64.7 (61.7,67.6)
**H**	**H**	**H**	**H**	0.0 (0.0,0.0)	0.0 (0.0,0.0)	0.0 (0.0,0.0)	95.4 (94.1,96.7)	95.2 (93.9,96.5)	88.5 (86.5,90.5)	88.9 (87.0,90.8)	0.0 (0.0,0.0)	0.0 (0.0,0.0)
**L**	**L**	**L**	**L**	37.2 (34.1,40.2)	0.0 (0.0,0.0)	0.0 (0.0,0.0)	40.1 (37.0,43.2)	86.8 (84.6,88.9)	9.2 (7.3,11.0)	8.6 (6.9,10.4)	2.6 (1.6,3.6)	69.3 (66.4,72.2)
**L**	**L**	**H**	**L**	0.2 (0.0,0.5)	0.0 (0.0,0.0)	0.0 (0.0,0.0)	39.7 (36.7,42.8)	93.8 (92.3,95.3)	16.4 (14.1,18.7)	16.6 (14.3,18.9)	0.0 (0.0,0.0)	71.7 (68.9,74.5)
**L**	**L**	**H**	**H**	2.7 (1.7,3.8)	2.8 (1.8,3.9)	2.8 (1.8,3.9)	97.3 (96.2,98.3)	87.6 (85.5,89.7)	56.0 (52.8,59.2)	53.8 (50.6,57.0)	2.7 (1.7,3.8)	2.6 (1.6,3.6)
**L**	**H**	**L**	**H**	0.0 (0.0,0.0)	0.0 (0.0,0.0)	0.0 (0.0,0.0)	94.2 (92.8,95.6)	92.0 (90.3,93.7)	24.5 (21.6,27.2)	88.6 (86.6,90.6)	3.3 (2.2,4.4)	0.0 (0.0,0.0)
**Sensitivity: L L H H**												
**Specificity**				π	Se_1	Se_2	Se_3	Se_4	Sp_1	Sp_2	Sp_3	Sp_4
**H**	**H**	**L**	**L**	0.0 (0.0,0.0)	0.0 (0.0,0.0)	0.0 (0.0,0.0)	93.2 (91.6,94.8)	95.4 (94.1,96.7)	86.2 (84.1,8.3)	86.3 (84.2,88.4)	4.4 (3.1,5.7)	4.4 (3.1,5.7)
**H**	**H**	**H**	**L**	0.0 (0.0,0.0)	0.0 (0.0,0.0)	0.0 (0.0,0.0)	94.7 (93.3,96.1)	94.9 (93.5,96.3)	90.0 (88.1,91.9)	90.3 (88.5,92.1)	0.0 (0.0,0.0)	3.5 (2.4,4.6)
**H**	**H**	**H**	**H**	0.0 (0.0,0.0)	0.0 (0.0,0.0)	0.0 (0.0,0.0)	95.5 (94.2,96.8)	95.6 (94.3,96.9)	90.1 (88.2,91.9)	89.1 (87.1,91.0)	0.0 (0.0,0.0)	0.0 (0.0,0.0)
**L**	**L**	**L**	**L**	0.7 (0.2,1.2)	0.0 (0.0,0.0)	0.0 (0.0,0.0)	84.1 (81.8,86.4)	85.4 (83.2,87.6)	24.0 (21.3,26.7)	23.0 (20.4,25.6)	4.3 (3.0,5.5)	2.4 (1.5,3.4)
**L**	**L**	**H**	**L**	0.0 (0.0,0.0)	0.0 (0.0,0.0)	0.0 (0.0,0.0)	92.6 (91.0,94.2)	84.8 (82.5,87.0)	40.9 (37.8,43.9)	40.6 (37.5,43.6)	0.0 (0.0,0.0)	3.5 (2.4,4.6)
**L**	**L**	**H**	**H**	94.0 (92.3,95.8)	86.5 (84.1,89.0)	89.2 (86.9,91.4)	97.6 (96.5,98.7)	95.4 (93.9,96.9)	95.0 (93.4,96.6)	94.6 (92.9,96.2)	94.0 (92.3,95.8)	95.4 (93.9,96.9)
**L**	**H**	**L**	**H**	0.0 (0.0,0.0)	0.0 (0.0,0.0)	0.0 (0.0,0.0)	94.9 (93.5,96.3)	93.8 (92.3,95.3)	25.9 (23.2,28.6)	91.3 (89.6,93.0)	2.3 (1.4,3.2)	0.0 (0.0,0.0)

x. Setting 10: $\pi = 0.2, \omega = 0.9, n_{obs} = 500$

Table x.1 Number of converged data sets for different sensitivity-specificity combinations

	**Number of converged data sets**

Specificity				Sensitivity: H H L L
H	H	L	L	961
H	H	H	L	998
H	H	H	H	998
L	L	L	L	518
L	L	H	L	943
L	L	H	H	993
L	H	L	H	999
**Specificity**				**Sensitivity: H H H L**
H	H	L	L	1000
H	H	H	L	1000
H	H	H	H	1000
L	L	L	L	911
L	L	H	L	991
L	L	H	H	999
L	H	L	H	999
**Specificity**				**Sensitivity: H H H H**
H	H	L	L	1000
H	H	H	L	999
H	H	H	H	998
L	L	L	L	989
L	L	H	L	999
L	L	H	H	998
L	H	L	H	1000
**Specificity**				**Sensitivity: L L L L**
H	H	L	L	907
H	H	H	L	996
H	H	H	H	1000
L	L	L	L	406
L	L	H	L	809
L	L	H	H	803
L	H	L	H	991
**Specificity**				**Sensitivity: L L H L**
H	H	L	L	996
H	H	H	L	1000
H	H	H	H	1000
L	L	L	L	695
L	L	H	L	973
L	L	H	H	668
L	H	L	H	995
**Specificity**				**Sensitivity: L L H H**

**H**	**H**	**L**	**L**	1000
**H**	**H**	**H**	**L**	999
**H**	**H**	**H**	**H**	929
**L**	**L**	**L**	**L**	893
**L**	**L**	**H**	**L**	818
**L**	**L**	**H**	**H**	711
**L**	**H**	**L**	**H**	995

Table x.2 Percentages of the time 95% credible intervals (CrIs) for residual correlations did not included '0' for any pairs, and percentages of the time that the overall χ^2 or G^2 statistic indicated a lack of overall fit

**Sensitivity: H H L L**						
**Specificity**				**Res. Cor.**	**Overall G^2**	**Overall χ^2**
**H**	**H**	**L**	**L**	61.1 (57.9,64.2)	12.0 (10.0,14.2)	10.0 (8.2,12.1)
**H**	**H**	**H**	**L**	25.7 (23.0,28.5)	3.4 (2.4,4.7)	7.4 (5.9,9.2)
**H**	**H**	**H**	**H**	16.3 (14.1,18.8)	2.4 (1.5,3.6)	18.1 (15.8,20.7)
**L**	**L**	**L**	**L**	56.4 (52.0,60.7)	17.4 (14.2,20.9)	16.0 (13.0,19.5)
**L**	**L**	**H**	**L**	29.3 (26.4,32.3)	20.6 (18.0,23.3)	19.2 (16.7,21.9)
**L**	**L**	**H**	**H**	8.4 (6.7,10.3)	16.9 (14.6,19.4)	44.6 (41.5,47.8)
**L**	**H**	**L**	**H**	36.7 (33.7,39.8)	6.6 (5.1,8.3)	12.6 (10.6,14.8)
**Sensitivity: H H H L**						
**Specificity**				**Res. Cor.**	**Overall G^2**	**Overall χ^2**
**H**	**H**	**L**	**L**	33.9 (31.0,36.9)	13.1 (11.1,15.4)	10.4 (8.6,12.5)
**H**	**H**	**H**	**L**	7.4 (5.9,9.2)	5.5 (4.2,7.1)	10.0 (8.2,12.0)
**H**	**H**	**H**	**H**	14.4 (12.3,16.7)	6.7 (5.2,8.4)	32.8 (29.9,35.8)
**L**	**L**	**L**	**L**	38.9 (35.7,42.1)	19.0 (16.5,21.7)	18.0 (15.6,20.7)
**L**	**L**	**H**	**L**	37.0 (34.0,40.1)	33.1 (30.2,36.1)	31.5 (28.6,34.5)
**L**	**L**	**H**	**H**	24.5 (21.9,27.3)	32.4 (29.5,35.4)	69.1 (66.1,71.9)
**L**	**H**	**L**	**H**	32.2 (29.3,35.2)	10.8 (9.0,12.9)	19.1 (16.7,21.7)
**Sensitivity: H H H H**						
**Specificity**				**Res. Cor.**	**Overall G^2**	**Overall χ^2**
**H**	**H**	**L**	**L**	33.1 (30.2,36.1)	12.1 (10.1,14.3)	13.0 (11.0,15.2)
**H**	**H**	**H**	**L**	24.8 (22.2,27.6)	9.5 (7.8,11.5)	18.3 (16.0,20.9)
**H**	**H**	**H**	**H**	42.3 (39.2,45.4)	16.7 (14.5,19.2)	62.6 (59.5,65.6)
**L**	**L**	**L**	**L**	33.5 (30.5,36.5)	24.5 (21.8,27.3)	24.8 (22.1,27.6)
**L**	**L**	**H**	**L**	42.6 (39.6,45.8)	48.5 (45.4,51.7)	51.8 (48.6,54.9)
**L**	**L**	**H**	**H**	48.2 (45.1,51.3)	50.7 (47.6,53.8)	87.6 (85.4,89.6)
**L**	**H**	**L**	**H**	42.1 (39.0,45.2)	18.7 (16.3,21.3)	34.1 (31.2,37.1)
**Sensitivity: L L L L**						

Specificity				Res. Cor.	Overall G^2	Overall χ^2
**H**	**H**	**L**	**L**	71.6 (68.5,74.5)	15.4 (13.1,18.0)	11.9 (9.9,14.2)
**H**	**H**	**H**	**L**	58.0 (54.9,61.1)	10.9 (9.1,13.0)	20.1 (17.6,22.7)
**H**	**H**	**H**	**H**	84.9 (82.5,87.1)	32.2 (29.3,35.2)	79.0 (76.3,81.5)
**L**	**L**	**L**	**L**	84.7 (80.9,88.1)	26.4 (22.1,30.9)	24.9 (20.7,29.4)
**L**	**L**	**H**	**L**	78.1 (75.1,80.9)	77.8 (74.7,80.6)	76.9 (73.8,79.7)
**L**	**L**	**H**	**H**	91.2 (89.0,93.0)	65.8 (62.4,69.0)	99.1 (98.2,99.6)
**L**	**H**	**L**	**H**	81.4 (78.9,83.8)	34.7 (31.7,37.8)	48.6 (45.5,51.8)
**Sensitivity: L L H L**						
**Specificity**				**Res. Cor.**	**Overall G^2**	**Overall χ^2**
**H**	**H**	**L**	**L**	60.2 (57.1,63.3)	19.6 (17.2,22.2)	18.5 (16.1,21.0)
**H**	**H**	**H**	**L**	59.5 (56.4,62.6)	24.3 (21.7,27.1)	32.9 (30.0,35.9)
**H**	**H**	**H**	**H**	98.7 (97.8,99.3)	58.0 (54.9,61.1)	98.0 (96.9,98.8)
**L**	**L**	**L**	**L**	78.6 (75.3,71.6)	59.6 (55.8,63.2)	59.4 (55.7,63.1)
**L**	**L**	**H**	**L**	91.4 (89.4,93.1)	95.1 (93.5,96.3)	96.6 (95.3,97.7)
**L**	**L**	**H**	**H**	97.0 (95.4,98.2)	78.6 (75.3,81.6)	100.0 (99.4,100.0)
**L**	**H**	**L**	**H**	95.3 (93.8,96.5)	60.4 (57.3,63.5)	88.0 (85.9,90.0)
**Sensitivity: L L H H**						
**Specificity**				**Res. Cor.**	**Overall G^2**	**Overall χ^2**
**H**	**H**	**L**	**L**	83.3 (80.8,85.6)	35.1 (32.1,38.1)	38.3 (35.3,41.4)
**H**	**H**	**H**	**L**	99.2 (98.4,99.7)	66.0 (62.9,68.9)	91.5 (89.6,93.1)
**H**	**H**	**H**	**H**	98.5 (97.5,99.2)	68.8 (65.7,71.8)	100.0 (99.6,100.0)
**L**	**L**	**L**	**L**	95.0 (93.3,96.3)	89.8 (87.6,91.7)	89.9 (87.8,91.8)
**L**	**L**	**H**	**L**	99.8 (99.1,100.0)	99.4 (98.6,99.8)	100.0 (99.6,100.0)
**L**	**L**	**H**	**H**	98.6 (97.4,99.3)	91.3 (89.0,93.2)	100.0 (99.5,100.0)
**L**	**H**	**L**	**H**	99.7 (99.1,99.9)	77.1 (74.3,79.7)	99.3 (98.6,99.7)

Table x.3 Percentages of the time 95% CrIs for residual correlations did not included '0' for each pair, and percentages of the time the pairwise χ^2 or G^2 statistic for each pair indicated a lack of pairwise fit

**Sensitivity: H H L L**										
**Specificity**				**Tool**	**T_1&T_2**	**T_1&T_3**	**T_1&T_4**	**T_2&T_3**	**T_2&T_4**	**T_3&T_4**
**H**	**H**	**L**	**L**	**Res. Cor.**	0.0 (0.0,0.4)	0.6 (0.2,1.4)	0.4 (0.1,1.1)	0.7 (0.3,1.5)	0.3 (0.1,0.9)	60.8 (57.6,63.9)
				**Pairwise G^2**	0.0 (0.0,0.4)	0.1 (0.0,0.6)	0.0 (0.0,0.4)	0.0 (0.0,0.4)	0.0 (0.0,0.4)	4.0 (2.8,5.4)
				**Pairwise χ^2**	0.0 (0.0,0.4)	0.1 (0.0,0.6)	0.0 (0.0,0.4)	0.0 (0.0,0.4)	0.0 (0.0,0.4)	4.0 (2.8,5.4)
**H**	**H**	**H**	**L**	**Res. Cor.**	0.0 (0.0,0.4)	0.0 (0.0,0.4)	0.7 (0.3,1.4)	0.0 (0.0,0.4)	1.1 (0.6,2.0)	24.2 (21.6,27.0)
				**Pairwise G^2**	0.0 (0.0,0.4)	0.0 (0.0,0.4)	0.0 (0.0,0.4)	0.0 (0.0,0.4)	0.1 (0.0,0.6)	2.4 (1.5,3.6)
				**Pairwise χ^2**	0.0 (0.0,0.4)	0.0 (0.0,0.4)	0.0 (0.0,0.4)	0.0 (0.0,0.4)	0.1 (0.0,0.6)	2.3 (1.5,3.4)
**H**	**H**	**H**	**H**	**Res. Cor.**	0.0 (0.0,0.4)	0.0 (0.0,0.4)	0.0 (0.0,0.4)	0.0 (0.0,0.4)	0.0 (0.0,0.4)	16.3 (14.1,18.8)

				**Pairwise G^2**	0.0 (0.0,0.4)	0.0 (0.0,0.4)	0.0 (0.0,0.4)	0.0 (0.0,0.4)	0.0 (0.0,0.4)	4.3 (3.1,5.8)
				**Pairwise χ^2**	0.0 (0.0,0.4)	0.0 (0.0,0.4)	0.0 (0.0,0.4)	0.0 (0.0,0.4)	0.0 (0.0,0.4)	4.3 (3.1,5.8)
**L**	**L**	**L**	**L**	**Res. Cor.**	0.0 (0.0,0.7)	4.8 (3.1.7.0)	10.4 (7.9,13.4)	8.5 (6.2,11.2)	7.9 (5.7,10.6)	42.3 (38.0,46.7)
				**Pairwise G^2**	0.0 (0.0,0.7)	0.2 (0.0,1.1)	0.6 (0.1,1.7)	0.2 (0.0,1.1)	0.6 (0.1,1.7)	3.3 (1.9,5.2)
				**Pairwise χ^2**	0.0 (0.0,0.7)	0.2 (0.0,1.1)	0.6 (0.1,1.7)	0.2 (0.0,1.1)	0.6 (0.1,1.7)	3.3 (1.9,5.2)
**L**	**L**	**H**	**L**	**Res. Cor.**	0.0 (0.0,0.4)	0.1 (0.0,0.6)	14.0 (11.8,16.4)	0.0 (0.0,0.4)	12.0 (10.0,14.2)	8.8 (7.1,10.8)
				**Pairwise G^2**	0.0 (0.0,0.4)	0.0 (0.0,0.4)	1.4 (0.7,2.3)	0.0 (0.0,0.4)	0.8 (0.4,1.7)	1.1 (0.5,1.9)
				**Pairwise χ^2**	0.0 (0.0,0.4)	0.0 (0.0,0.4)	1.4 (0.7,2.3)	0.0 (0.0,0.4)	0.8 (0.4,1.7)	1.1 (0.5,1.9)
**L**	**L**	**H**	**H**	**Res. Cor.**	2.7 (1.8,3.9)	0.4 (0.1,1.0)	1.2 (0.6,2.1)	0.8 (0.3,1.6)	0.6 (0.2,1.3)	3.3 (2.3,4.6)
				**Pairwise G^2**	0.5 (0.2,1.2)	0.0 (0.0,0.4)	0.2 (0.0,0.7)	0.0 (0.0,0.4)	0.1 (0.0,0.6)	1.7 (1.0,2.7)
				**Pairwise χ^2**	0.5 (0.2,1.2)	0.0 (0.0,0.4)	0.2 (0.0,0.7)	0.0 (0.0,0.4)	0.1 (0.0,0.6)	1.7 (1.0,2.7)
**L**	**H**	**L**	**H**	**Res. Cor.**	0.0 (0.0,0.4)	21.9 (19.4,24.6)	3.6 (2.5,5.0)	0.4 (0.1,1.0)	0.0 (0.0,0.4)	16.9 (14.6,19.4)
				**Pairwise G^2**	0.0 (0.0,0.4)	1.5 (0.8,2.5)	0.6 (0.2,1.3)	0.0 (0.0,0.4)	0.0 (0.0,0.4)	1.9 (1.1,3.0)
				**Pairwise χ^2**	0.0 (0.0,0.4)	1.5 (0.8,2.5)	0.7 (0.3,1.4)	0.0 (0.0,0.4)	0.0 (0.0,0.4)	1.9 (1.1,3.0)
**Sensitivity: H H H L**										
**Specificity**				**Tool**	**T_1&T_2**	**T_1&T_3**	**T_1&T_4**	**T_2&T_3**	**T_2&T_4**	**T_3&T_4**
**H**	**H**	**L**	**L**	**Res. Cor.**	0.0 (0.0,0.4)	0.0 (0.0,0.4)	0.6 (0.2,1.3)	0.3 (0.1,0.9)	1.0 (0.5,1.8)	32.8 (29.9,35.8)
				**Pairwise G^2**	0.0 (0.0,0.4)	0.0 (0.0,0.4)	0.0 (0.0,0.4)	0.0 (0.0,0.4)	0.0 (0.0,0.4)	3.2 (2.2,4.5)
				**Pairwise χ^2**	0.0 (0.0,0.4)	0.0 (0.0,0.4)	0.0 (0.0,0.4)	0.0 (0.0,0.4)	0.0 (0.0,0.4)	3.2 (2.2,4.5)
**H**	**H**	**H**	**L**	**Res. Cor.**	0.0 (0.0,0.4)	0.0 (0.0,0.4)	1.1 (0.6,2.0)	0.0 (0.0,0.4)	1.3 (0.7,2.2)	5.6 (4.3,7.2)
				**Pairwise G^2**	0.0 (0.0,0.4)	0.0 (0.0,0.4)	0.3 (0.1,0.9)	0.0 (0.0,0.4)	0.0 (0.0,0.4)	0.6 (0.2,1.3)
				**Pairwise χ^2**	0.0 (0.0,0.4)	0.0 (0.0,0.4)	0.3 (0.1,0.9)	0.0 (0.0,0.4)	0.0 (0.0,0.4)	0.5 (0.2,1.2)
**H**	**H**	**H**	**H**	**Res. Cor.**	0.0 (0.0,0.4)	0.0 (0.0,0.4)	0.0 (0.0,0.4)	0.0 (0.0,0.4)	0.0 (0.0,0.4)	14.4 (12.3,16.7)
				**Pairwise G^2**	0.0 (0.0,0.4)	0.0 (0.0,0.4)	0.0 (0.0,0.4)	0.0 (0.0,0.4)	0.0 (0.0,0.4)	5.9 (4.5,7.5)
				**Pairwise χ^2**	0.0 (0.0,0.4)	0.0 (0.0,0.4)	0.0 (0.0,0.4)	0.0 (0.0,0.4)	0.0 (0.0,0.4)	5.6 (4.3,7.2)
**L**	**L**	**L**	**L**	**Res. Cor.**	0.1 (0.0,0.6)	1.6 (0.9,2.7)	11.9 (9.8,14.1)	1.9 (1.1,3.0)	14.6 (12.4,17.1)	18.8 (16.3,21.5)
				**Pairwise G^2**	0.0 (0.0,0.4)	0.0 (0.0,0.4)	0.9 (0.4,1.7)	0.5 (0.2,1.3)	0.5 (0.2,1.3)	2.3 (1.4,3.5)
				**Pairwise χ^2**	0.0 (0.0,0.4)	0.0 (0.0,0.4)	0.9 (0.4,1.7)	0.5 (0.2,1.3)	0.5 (0.2,1.3)	2.3 (1.4,3.5)
**L**	**L**	**H**	**L**	**Res. Cor.**	1.6 (0.9,2.6)	0.1 (0.0,0.6)	19.0 (16.6,21.6)	0.2 (0.0,0.7)	19.8 (17.3,22.4)	3.3 (2.3,4.6)
				**Pairwise G^2**	0.2 (0.0,0.7)	0.1 (0.0,0.6)	2.3 (1.5,3.5)	0.0 (0.0,0.4)	1.5 (0.8,2.5)	0.7 (0.3,1.4)
				**Pairwise χ^2**	0.2 (0.0,0.7)	0.1 (0.0,0.6)	2.3 (1.5,3.5)	0.0 (0.0,0.4)	1.5 (0.8,2.5)	0.7 (0.3,1.4)
**L**	**L**	**H**	**H**	**Res. Cor.**	14.3 (12.2,16.6)	0.3 (0.1,0.9)	3.7 (2.6,5.1)	0.7 (0.3,1.4)	3.0 (2.0,4.3)	7.4 (5.9,9.2)
				**Pairwise G^2**	2.1 (1.3,3.2)	0.1 (0.0,0.6)	0.3 (0.1,0.9)	0.2 (0.0,0.7)	0.4 (0.1,1.0)	4.3 (3.1,5.8)
				**Pairwise χ^2**	2.1 (1.3,3.2)	0.1 (0.0,0.6)	0.5 (0.2,1.2)	0.2 (0.0,0.7)	0.5 (0.2,1.2)	4.1 (3.0,5.5)
**L**	**H**	**L**	**H**	**Res. Cor.**	0.0 (0.0,0.4)	20.5 (18.1,23.2)	5.7 (4.3,7.3)	0.0 (0.0,0.4)	0.0 (0.0,0.4)	10.9 (9.0,13.0)
				**Pairwise G^2**	0.0 (0.0,0.4)	2.3 (1.5,3.4)	0.2 (0.0,0.7)	0.0 (0.0,0.4)	0.0 (0.0,0.4)	2.8 (1.9,4.0)
				**Pairwise χ^2**	0.0 (0.0,0.4)	2.3 (1.5,3.4)	0.2 (0.0,0.7)	0.0 (0.0,0.4)	0.0 (0.0,0.4)	2.4 (1.5,3.6)
**Sensitivity: H H H H**										
**Specificity**				**Tool**	**T_1&T_2**	**T_1&T_3**	**T_1&T_4**	**T_2&T_3**	**T_2&T_4**	**T_3&T_4**
**H**	**H**	**L**	**L**	**Res. Cor.**	0.0 (0.0,0.4)	0.4 (0.1,1.0)	0.3 (0.1,0.9)	0.0 (0.0,0.4)	0.5 (0.2,1.2)	32.4 (29.5,35.4)
				**Pairwise G^2**	0.0 (0.0,0.4)	0.0 (0.0,0.4)	0.1 (0.0,0.6)	0.0 (0.0,0.4)	0.2 (0.0,0.7)	3.1 (2.1,4.4)

				**Pairwise χ^2**	0.0 (0.0,0.4)	0.0 (0.0,0.4)	0.1 (0.0,0.6)	0.0 (0.0,0.4)	0.2 (0.0,0.7)	3.1 (2.1,4.4)
**H**	**H**	**H**	**L**	**Res. Cor.**	0.0 (0.0,0.4)	0.0 (0.0,0.4)	1.1 (0.6,2.0)	0.0 (0.0,0.4)	1.1 (0.6,2.0)	23.0 (20.4,25.8)
				**Pairwise G^2**	0.0 (0.0,0.4)	0.0 (0.0,0.4)	0.1 (0.0,0.6)	0.0 (0.0,0.4)	0.3 (0.1,0.9)	7.6 (6.0,9.4)
				**Pairwise χ^2**	0.0 (0.0,0.4)	0.0 (0.0,0.4)	0.1 (0.0,0.6)	0.0 (0.0,0.4)	0.3 (0.1,0.9)	6.7 (5.2,8.4)
**H**	**H**	**H**	**H**	**Res. Cor.**	0.1 (0.0,0.6)	0.0 (0.0,0.4)	0.0 (0.0,0.4)	0.0 (0.0,0.4)	0.0 (0.0,0.4)	42.3 (39.2,45.4)
				**Pairwise G^2**	0.0 (0.0,0.4)	0.0 (0.0,0.4)	0.0 (0.0,0.4)	0.0 (0.0,0.4)	0.0 (0.0,0.4)	24.8 (22.2,27.7)
				**Pairwise χ^2**	0.0 (0.0,0.4)	0.0 (0.0,0.4)	0.0 (0.0,0.4)	0.0 (0.0,0.4)	0.0 (0.0,0.4)	23.3 (20.8,26.1)
**L**	**L**	**L**	**L**	**Res. Cor.**	1.5 (0.9,2.5)	8.5 (6.8,10.4)	7.4 (5.8,6.2)	7.0 (5.5,8.7)	7.5 (5.9,9.3)	12.7 (10.7,15.0)
				**Pairwise G^2**	0.4 (0.1,1.0)	1.1 (0.6,2.0)	0.7 (0.3,1.5)	0.7 (0.3,1.5)	0.9 (0.4,1.7)	1.9 (1.2,3.0)
				**Pairwise χ^2**	0.4 (0.1,1.0)	1.4 (0.8,2.4)	0.7 (0.3,1.5)	0.7 (0.3,1.5)	0.9 (0.4,1.7)	1.9 (1.2,3.0)
**L**	**L**	**H**	**L**	**Res. Cor.**	10.1 (8.3,12.1)	0.9 (0.4,1.7)	15.9 (13.7,18.3)	0.7 (0.3,1.4)	17.5 (15.2,20.0)	13.1 (11.1,15.4)
				**Pairwise G^2**	1.9 (1.1,3.0)	0.1 (0.0,0.6)	1.8 (1.1,2.8)	0.2 (0.0,0.7)	2.4 (1.5,3.6)	4.2 (3.0,5.6)
				**Pairwise χ^2**	1.9 (1.1,3.0)	0.1 (0.0,0.6)	1.8 (1.1,2.8)	0.2 (0.0,0.7)	2.5 (2.4,4.7)	3.4 (2.4,4.7)
**L**	**L**	**H**	**H**	**Res. Cor.**	28.0 (25.2,30.9)	1.8 (1.1,2.8)	0.8 (0.3,1.6)	1.5 (0.8,2.5)	2.4 (1.5,3.6)	29.7 (26.8,32.6)
				**Pairwise G^2**	1.9 (1.1,3.0)	0.1 (0.0,0.6)	1.8 (1.1,2.8)	0.2 (0.0,0.7)	2.4 (1.5,3.6)	4.2 (3.0,5.6)
				**Pairwise χ^2**	1.9 (1.1,3.0)	0.1 (0.0,0.6)	1.8 (1.1,2.8)	0.2 (0.0,0.7)	2.5 (2.4,4.7)	3.4 (2.4,4.7)
**L**	**H**	**L**	**H**	**Res. Cor.**	0.5 (0.2,1.2)	23.9 (21.3,26.7)	4.8 (3.6,6.3)	0.3 (0.1,0.9)	0.0 (0.0,0.4)	19.7 (17.3,22.3)
				**Pairwise G^2**	0.3 (0.1,0.9)	1.8 (1.1,2.8)	0.7 (0.3,1.4)	0.0 (0.0,0.4)	0.0 (0.0,0.4)	5.2 (3.9,6.8)
				**Pairwise χ^2**	0.3 (0.1,0.9)	1.9 (1.1,3.0)	0.8 (0.3,1.6)	0.0 (0.0,0.4)	0.0 (0.0,0.4)	4.4 (3.2,5.9)
**Sensitivity: L L L L**										
**Specificity**				**Tool**	**T_1&T_2**	**T_1&T_3**	**T_1&T_4**	**T_2&T_3**	**T_2&T_4**	**T_3&T_4**
**H**	**H**	**L**	**L**	**Res. Cor.**	0.0 (0.0,0.4)	4.9 (3.5,6.5)	2.8 (1.8,4.0)	4.2 (3.0,5.7)	3.4 (2.3,4.8)	69.2 (66.2,72.2)
				**Pairwise G^2**	0.0 (0.0,0.4)	0.1 (0.0,0.6)	0.0 (0.0,0.4)	0.1 (0.0,0.6)	0.0 (0.0,0.4)	6.6 (5.1,8.4)
				**Pairwise χ^2**	0.0 (0.0,0.4)	0.1 (0.0,0.6)	0.0 (0.0,0.4)	0.1 (0.0,0.6)	0.0 (0.0,0.4)	6.6 (5.1,8.4)
**H**	**H**	**H**	**L**	**Res. Cor.**	0.0 (0.0,0.4)	0.0 (0.0,0.4)	4.9 (3.7,6.5)	0.0 (0.0,0.4)	4.3 (3.1,5.8)	54.4 (51.3,57.5)
				**Pairwise G^2**	0.0 (0.0,0.4)	0.0 (0.0,0.4)	0.2 (0.0,0.7)	0.0 (0.0,0.4)	0.0 (0.0,0.4)	10.3 (8.5,12.4)
				**Pairwise χ^2**	0.0 (0.0,0.4)	0.0 (0.0,0.4)	0.2 (0.0,0.7)	0.0 (0.0,0.4)	0.0 (0.0,0.4)	10.3 (8.5,12.4)
**H**	**H**	**H**	**H**	**Res. Cor.**	0.0 (0.0,0.4)	0.0 (0.0,0.4)	0.0 (0.0,0.4)	0.0 (0.0,0.4)	0.0 (0.0,0.4)	84.9 (82.5,87.1)
				**Pairwise G^2**	0.1 (0.0,0.6)	0.0 (0.0,0.4)	0.0 (0.0,0.4)	0.0 (0.0,0.4)	0.0 (0.0,0.4)	63.3 (60.2,66.3)
				**Pairwise χ^2**	0.1 (0.0,0.6)	0.0 (0.0,0.4)	0.0 (0.0,0.4)	0.0 (0.0,0.4)	0.0 (0.0,0.4)	63.7 (60.6,66.7)
**L**	**L**	**L**	**L**	**Res. Cor.**	0.0 (0.0,0.4)	23.2 (19.1,27.6)	29.6 (25.2,34.3)	31.0 (26.6,35.8)	30.0 (25.6,34.8)	61.3 (56.4,66.1)
				**Pairwise G^2**	0.0 (0.0,0.9)	1.0 (0.3,2.5)	1.7 (0.7,3.5)	2.7 (1.4,4.8)	2.5 (1.2,4.5)	4.4 (2.6,6.9)
				**Pairwise χ^2**	0.0 (0.0,0.9)	1.0 (0.3,2.5)	1.7 (0.7,3.5)	2.7 (1.4,4.8)	2.5 (1.2,4.5)	4.4 (2.6,6.9)
**L**	**L**	**H**	**L**	**Res. Cor.**	0.0 (0.0,0.5)	13.5 (11.2,16.0)	28.1 (25.0,31.3)	12.7 (10.5,15.2)	33.5 (30.2,36.9)	50.6 (47.1,54.1)
				**Pairwise G^2**	0.1 (0.0,0.7)	1.1 (0.5,2.1)	3.0 (1.9,4.4)	2.6 (1.6,3.9)	2.8 (1.8,4.2)	14.2 (11.9,16.8)
				**Pairwise χ^2**	0.1 (0.0,0.7)	1.2 (0.6,2.3)	3.0 (1.9,4.4)	2.5 (1.5,3.8)	2.8 (1.8,4.2)	14.3 (12.0,16.9)
**L**	**L**	**H**	**H**	**Res. Cor.**	14.2 (11.9,16.8)	27.5 (24.5,30.8)	23.7 (20.8,26.8)	25.4 (22.4,28.6)	26.3 (23.3,29.5)	74.3 (71.2,77.3)
				**Pairwise G^2**	12.3 (10.1,14.8)	6.1 (4.5,8.0)	5.2 (3.8,7.0)	5.4 (3.9,7.1)	5.1 (3.7,6.9)	70.7 (67.5,73.9)
				**Pairwise χ^2**	12.2 (10.0,14.7)	6.1 (4.5,8.0)	5.4 (3.9,7.1)	5.5 (4.0,7.3)	5.2 (3.8,7.0)	71.5 (68.2,74.6)
**L**	**H**	**L**	**H**	**Res. Cor.**	0.0 (0.0,0.4)	40.8 (37.7,43.9)	49.8 (46.7,53.0)	4.5 (3.3,6.0)	0.0 (0.0,0.4)	47.8 (44.7,51.0)
				**Pairwise G^2**	0.0 (0.0,0.4)	3.9 (2.8,5.3)	14.4 (12.3,16.8)	0.0 (0.0,0.4)	0.0 (0.0,0.4)	9.4 (7.6,11.4)

				**Pairwise χ^2**	0.0 (0.0,0.4)	3.9 (2.8,5.3)	14.6 (12.5,17.0)	0.0 (0.0,0.4)	0.0 (0.0,0.4)	9.4 (7.6,11.4)
**Sensitivity: L L H L**										
**Specificity**				**Tool**	**T_1&T_2**	**T_1&T_3**	**T_1&T_4**	**T_2&T_3**	**T_2&T_4**	**T_3&T_4**
**H**	**H**	**L**	**L**	**Res. Cor.**	0.0 (0.0,0.4)	1.9 (1.2,3.0)	4.7 (3.5,6.2)	1.6 (0.9,2.6)	5.3 (4.0,6.9)	55.7 (52.6,58.8)
				**Pairwise G^2**	0.0 (0.0,0.4)	0.1 (0.0,0.6)	0.1 (0.0,0.6)	0.1 (0.0,0.6)	0.2 (0.0,0.7)	8.1 (6.5,10.0)
				**Pairwise χ^2**	0.0 (0.0,0.4)	0.1 (0.0,0.6)	0.1 (0.0,0.6)	0.2 (0.0,0.7)	0.2 (0.0,0.7)	8.1 (6.5,10.0)
**H**	**H**	**H**	**L**	**Res. Cor.**	0.0 (0.0,0.4)	0.0 (0.0,0.4)	5.9 (4.5,7.5)	0.0 (0.0,0.4)	6.0 (4.6,7.7)	55.1 (52.0,58.2)
				**Pairwise G^2**	0.0 (0.0,0.4)	0.0 (0.0,0.4)	0.3 (0.1,0.9)	0.0 (0.0,0.4)	0.2 (0.0,0.7)	16.9 (14.6,19.4)
				**Pairwise χ^2**	0.0 (0.0,0.4)	0.0 (0.0,0.4)	0.3 (0.1,0.9)	0.0 (0.0,0.4)	0.2 (0.0,0.7)	16.8 (14.5,19.3)
**H**	**H**	**H**	**H**	**Res. Cor.**	0.0 (0.0,0.4)	0.0 (0.0,0.4)	0.2 (0.0,0.7)	0.0 (0.0,0.4)	0.0 (0.0,0.4)	98.7 (97.8,99.3)
				**Pairwise G^2**	0.0 (0.0,0.4)	0.0 (0.0,0.4)	0.0 (0.0,0.4)	0.0 (0.0,0.4)	0.0 (0.0,0.4)	96.1 (94.7,97.2)
				**Pairwise χ^2**	0.0 (0.0,0.4)	0.0 (0.0,0.4)	0.0 (0.0,0.4)	0.0 (0.0,0.4)	0.0 (0.0,0.4)	96.1 (94.7,97.2)
**L**	**L**	**L**	**L**	**Res. Cor.**	0.0 (0.0,0.5)	17.3 (14.5,20.3)	28.9 (25.6,32.4)	15.4 (12.8,18.3)	30.5 (27.1,34.1)	51.4 (47.6,55.1)
				**Pairwise G^2**	0.0 (0.0,0.5)	3.9 (2.6,5.6)	4.0 (2.7,5.8)	3.7 (2.5,5.4)	2.7 (1.7,4.2)	9.5 (7.4,11.9)
				**Pairwise χ^2**	0.0 (0.0,0.5)	3.9 (2.6,5.6)	4.0 (2.7,5.8)	3.7 (2.5,5.4)	2.7 (1.7,4.2)	9.4 (7.3,11.8)
**L**	**L**	**H**	**L**	**Res. Cor.**	0.3 (0.1,0.9)	34.1 (31.1,37.2)	39.0 (35.9,42.1)	33.2 (30.2,36.3)	39.6 (36.5,42.7)	63.6 (60.5,66.6)
				**Pairwise G^2**	0.1 (0.0,0.6)	13.3 (11.2,15.6)	5.1 (3.8,6.7)	14.1 (12.0,16.4)	5.2 (3.9,6.8)	26.1 (23.4,29.0)
				**Pairwise χ^2**	0.1 (0.0,0.6)	13.6 (11.5,15.9)	5.1 (3.8,6.7)	14.4 (12.2,16.8)	5.2 (3.9,6.8)	25.9 (23.2,28.8)
**L**	**L**	**H**	**H**	**Res. Cor.**	51.2 (47.3,55.1)	29.5 (26.1,33.1)	36.4 (32.7,40.2)	30.4 (26.9,34.0)	37.1 (33.5,40.9)	70.7 (67.0,74.1)
				**Pairwise G^2**	30.5 (27.1,34.2)	13.9 (11.4,16.8)	12.0 (9.6,14.7)	14.2 (11.7,17.1)	11.4 (9.1,14.0)	79.2 (75.9,82.2)
				**Pairwise χ^2**	30.1 (26.6,33.7)	14.5 (11.9,17.4)	12.1 (9.7,14.8)	14.7 (12.1,17.6)	11.5 (9.2,14.2)	79.0 (75.8,82.1)
**L**	**H**	**L**	**H**	**Res. Cor.**	0.0 (0.0,0.4)	56.8 (53.6,59.9)	57.4 (54.2,60.5)	0.4 (0.1,1.0)	0.0 (0.0,0.4)	88.3 (86.2,90.3)
				**Pairwise G^2**	0.0 (0.0,0.4)	14.9 (12.7,17.2)	17.9 (15.6,20.4)	0.1 (0.0,0.6)	0.0 (0.0,0.4)	62.8 (59.7,65.8)
				**Pairwise χ^2**	0.0 (0.0,0.4)	15.0 (12.8,17.3)	18.2 (15.8,20.7)	0.1 (0.0,0.6)	0.0 (0.0,0.4)	61.3 (58.2,64.3)
**Sensitivity: L L H H**										
**Specificity**				**Tool**	**T_1&T_2**	**T_1&T_3**	**T_1&T_4**	**T_2&T_3**	**T_2&T_4**	**T_3&T_4**
**H**	**H**	**L**	**L**	**Res. Cor.**	0.0 (0.0,0.4)	1.8 (1.1,2.8)	2.2 (1.4,3.3)	2.1 (1.3,3.2)	2.5 (1.6,3.7)	82.1 (79.6,84.4)
				**Pairwise G^2**	0.0 (0.0,0.4)	0.3 (0.1,0.9)	0.2 (0.0,0.7)	0.1 (0.0,0.6)	0.2 (0.0,0.7)	34.4 (31.5,37.4)
				**Pairwise χ^2**	0.0 (0.0,0.4)	0.4 (0.1,1.0)	0.2 (0.0,0.7)	0.2 (0.0,0.7)	0.3 (0.1,0.9)	34.1 (31.2,37.1)
**H**	**H**	**H**	**L**	**Res. Cor.**	0.0 (0.0,0.4)	0.0 (0.0,0.4)	4.1 (3.0,5.5)	0.1 (0.0,0.6)	3.1 (2.1,4.4)	99.2 (98.4,99.7)
				**Pairwise G^2**	0.0 (0.0,0.4)	0.0 (0.0,0.4)	0.3 (0.1,0.9)	0.0 (0.0,0.4)	0.6 (0.2,1.3)	92.6 (90.8,94.1)
				**Pairwise χ^2**	0.0 (0.0,0.4)	0.0 (0.0,0.4)	0.4 (0.1,1.0)	0.0 (0.0,0.4)	0.7 (0.3,1.4)	91.7 (89.8,93.3)
**H**	**H**	**H**	**H**	**Res. Cor.**	1.9 (1.2,3.0)	0.0 (0.0,0.4)	0.0 (0.0,0.4)	0.0 (0.0,0.4)	0.2 (0.0,0.8)	96.6 (95.2,97.6)
				**Pairwise G^2**	3.1 (2.1,4.5)	0.0 (0.0,0.4)	0.0 (0.0,0.4)	0.0 (0.0,0.4)	0.0 (0.0,0.4)	98.2 (97.1,98.9)
				**Pairwise χ^2**	3.0 (2.0,4.3)	0.0 (0.0,0.4)	0.0 (0.0,0.4)	0.0 (0.0,0.4)	0.0 (0.0,0.4)	98.1 (97.0,98.8)
**L**	**L**	**L**	**L**	**Res. Cor.**	1.1 (0.5,2.0)	35.6 (32.5,38.9)	35.7 (32.6,39.0)	34.2 (31.0,37.4)	31.5 (28.4,34.6)	84.3 (81.8,86.6)
				**Pairwise G^2**	0.7 (0.2,1.5)	8.6 (6.9,10.7)	11.3 (9.3,13.6)	9.2 (7.4,11.3)	7.7 (6.1,9.7)	48.2 (44.8,51.5)
				**Pairwise χ^2**	0.6 (0.2,1.3)	8.7 (7.0,10.8)	11.3 (9.3,13.6)	9.6 (7.8,11.8)	8.0 (6.3,9.9)	48.0 (44.7,51.4)
**L**	**L**	**H**	**L**	**Res. Cor.**	24.8 (21.9,27.9)	52.2 (48.7,55.7)	53.1 (49.6,56.5)	53.2 (49.7,56.6)	54.6 (51.2,58.1)	98.0 (96.8,98.9)
				**Pairwise G^2**	8.4 (6.6,10.6)	24.4 (21.5,27.5)	16.4 (13.9,19.1)	23.6 (20.7,26.7)	18.1 (15.5,20.9)	95.5 (93.8,96.8)
				**Pairwise χ^2**	8.3 (6.5,10.4)	25.6 (22.6,28.7)	16.5 (14.0,19.2)	24.2 (21.3,27.3)	18.3 (15.7,21.2)	95.4 (93.7,96.7)

**L**	**L**	**H**	**H**	**Res. Cor.**	96.3 (94.7,97.6)	2.5 (1.5,4.0)	2.5 (1.5,4.0)	3.1 (1.9,4.6)	3.8 (2.5,5.5)	5.5 (3.9,7.4)
				**Pairwise G^2**	90.0 (87.6,92.1)	1.3 (0.6,2.4)	1.5 (0.8,2.8)	2.5 (1.5,4.0)	1.1 (0.5,2.2)	9.7 (7.6,12.1)
				**Pairwise χ^2**	90.0 (87.6,92.1)	1.4 (0.7,2.6)	1.5 (0.8,2.8)	2.5 (1.5,4.0)	1.4 (0.7,2.6)	9.7 (7.6,12.1)
**L**	**H**	**L**	**H**	**Res. Cor.**	0.8 (0.3,1.6)	60.2 (57.1,63.3)	82.4 (79.9,84.7)	2.2 (1.4,3.3)	0.0 (0.0,0.4)	99.2 (98.4,99.7)
				**Pairwise G^2**	0.4 (0.1,1.0)	16.3 (14.0,18.7)	50.7 (47.5,53.8)	0.2 (0.0,0.7)	0.0 (0.0,0.4)	93.5 (91.7,94.9)
				**Pairwise χ^2**	0.3 (0.1,0.9)	16.5 (14.2,18.9)	51.8 (48.6,54.9)	0.2 (0.0,0.7)	0.0 (0.0,0.4)	92.9 (91.1,94.4)

Table x.4 Mean absolute biases (x10^{-2}) of posterior medians of each parameter obtained from the conditional independence model

**Sensitivity: H H L L**												
**Specificity**				π	Se_1	Se_2	Se_3	Se_4	Sp_1	Sp_2	Sp_3	Sp_4
**H**	**H**	**L**	**L**	2.3 (2.1,2.4)	0.9 (0.6,1.2)	0.6 (0.3,0.9)	-1.4 (-1.7,-1.1)	-1.3 (-1.6,-1.0)	2.0 (1.9,2.1)	2.1 (2.0,2.2)	-0.1 (-0.2,0.1)	0.1 (-0.1,0.3)
**H**	**H**	**H**	**L**	-0.5 (-0.7,-0.4)	5.9 (5.8,6.0)	5.7 (5.6,5.9)	-1.1 (-1.5,-0.8)	-0.3 (-0.6,0.0)	0.7 (0.6,0.8)	0.6 (0.5,0.7)	-0.8 (-0.9,-0.7)	-0.2 (-0.4,-0.1)
**H**	**H**	**H**	**H**	-1.1 (-1.2,-1.0)	6.8 (6.7,6.9)	6.8 (6.7,6.9)	-1.0 (-1.3,-.7)	-1.1 (-1.4,-0.7)	0.4 (0.3,0.5)	0.5 (0.4,0.6)	-1.0 (-1.0,-0.9)	-1.1 (-1.2,-1.0)
**L**	**L**	**L**	**L**	9.7 (9.2,10.3)	-4.1 (-4.6,-3.6)	-3.8 (-4.3,-3.3)	-2.7 (-3.3,-2.1)	-2.6 (-3.2,-2.1)	4.7 (4.4,5.1)	5.0 (4.6,5.4)	1.4 (1.2,1.6)	1.2 (1.0,1.5)
**L**	**L**	**H**	**L**	1.4 (1.2,1.6)	3.9 (3.7,4.1)	3.8 (3.6,4.1)	-3.6 (-4.2,-3.0)	-0.9 (-1.3,-0.5)	2.0 (1.8,2.2)	2.0 (1.8,2.2)	-0.5 (-0.6,-0.4)	-0.1 (-0.2,-0.1)
**L**	**L**	**H**	**H**	-0.5 (-0.7,-0.3)	6.0 (5.8,6.1)	5.8 (5.7,6.0)	-1.6 (-2.1,-1.2)	-1.6 (-2.1,-1.1)	1.1 (0.9,1.3)	1.0 (0.8,1.2)	-0.9 (-1.0,-0.8)	-0.9 (-1.0,-0.7)
**L**	**H**	**L**	**H**	0.2 (0.1,0.4)	6.2 (6.1,6.4)	3.4 (3.2,3.6)	-0.9 (-1.2,-0.5)	-2.0 (-2.4,-1.6)	1.7 (1.5,1.8)	0.8 (0.7,0.9)	-0.3 (-0.4,-0.1)	-0.6 (-0.7,-0.5)
**Sensitivity: H H H L**												
**Specificity**				π	Se_1	Se_2	Se_3	Se_4	Sp_1	Sp_2	Sp_3	Sp_4
**H**	**H**	**L**	**L**	-0.1 (-0.2,0.2)	4.8 (4.7,5.0)	4.7 (4.6,4.9)	-1.3 (-1.5,-1.0)	-0.8 (-1.2,-0.5)	0.9 (0.8,1.0)	1.0 (0.9,1.1)	-0.4 (-0.6,-0.3)	-0.3 (-0.4,-0.1)
**H**	**H**	**H**	**L**	-1.3 (-1.4,-1.1)	7.3 (7.2,7.4)	7.3 (7.2,7.4)	-0.9 (-1.1,-0.6)	-0.4 (-0.7,-0.1)	0.2 (0.1,0.3)	0.2 (0.1,0.3)	-1.7 (-1.8,-1.6)	-0.5 (-0.7,-0.4)
**H**	**H**	**H**	**H**	-1.4 (-1.6,-1.3)	7.6 (7.5,7.7)	7.5 (7.4,7.6)	-1.0 (-1.2,-0.7)	-0.6 (-0.9,-0.3)	0.3 (0.2,0.4)	0.3 (0.2,0.4)	-1.7 (-1.8,-1.6)	-1.3 (-1.4,-1.2)
**L**	**L**	**L**	**L**	4.3 (4.1,4.6)	1.7 (1.5,2.0)	1.4 (1.1,1.6)	-6.5 (-6.9,-6.1)	-1.9 (-2.3,-1.5)	3.2 (3.0,3.4)	3.1 (2.9,3.3)	0.4 (0.2,0.6)	0.3 (0.2,0.5)
**L**	**L**	**H**	**L**	0.5 (0.4,0.7)	6.4 (6.3,6.5)	6.4 (6.3,6.5)	-6.7 (-7.2,-6.3)	-0.5 (-0.9,-0.2)	1.9 (1.7,2.1)	2.0 (1.8,2.1)	-1.5 (-1.6,-1.4)	-0.2 (-0.4,-0.1)
**L**	**L**	**H**	**H**	-1.2 (-1.3,-1.1)	7.1 (7.0,7.2)	7.2 (7.1,7.3)	-1.5 (-1.8,-1.1)	-0.2 (-0.6,0.1)	0.9 (0.7,1.1)	0.8 (0.7,1.0)	-1.9 (-2.0,-1.8)	-1.0 (-1.2,-0.9)
**L**	**H**	**L**	**H**	-0.4 (-0.5,-0.3)	6.7 (6.6,6.8)	5.1 (4.9,5.2)	-1.4 (-1.7,-1.2)	-1.5 (-1.8,-1.1)	1.4 (1.3,1.6)	0.7 (0.5,0.8)	-0.6 (-0.7,-0.4)	-0.9 (-1.0,-0.8)
**Sensitivity: H H H H**												
**Specificity**				π	Se_1	Se_2	Se_3	Se_4	Sp_1	Sp_2	Sp_3	Sp_4
**H**	**H**	**L**	**L**	-0.7 (-0.9,-0.6)	6.0 (5.9,6.1)	6.0 (5.9,6.2)	-1.3 (-1.5,-1.0)	-1.0 (-1.2,-0.7)	0.6 (0.5,0.7)	0.7 (0.6,0.8)	-0.6 (-0.8,-0.5)	-0.8 (-1.0,-0.7)
**H**	**H**	**H**	**L**	-1.5 (-1.6,-1.3)	7.5 (7.5,7.6)	7.5 (7.4,7.6)	-0.9 (-1.1,-0.6)	-0.8 (-1.0,-0.6)	0.4 (0.3,0.5)	0.2 (0.1,0.3)	-1.7 (-1.8,-1.6)	-1.1 (-1.3,-1.0)
**H**	**H**	**H**	**H**	-1.6 (-1.7,-1.5)	7.8 (7.8,7.9)	7.9 (7.8,7.9)	-0.9 (-1.1,-0.7)	-0.8 (-1.0,-0.6)	0.1 (0.1,0.2)	0.1 (0.0,0.2)	-1.8 (-1.9,-1.7)	-1.9 (-2.0,-1.8)
**L**	**L**	**L**	**L**	1.5 (1.3,1.6)	4.0 (3.8,4.2)	4.1 (4.0,4.3)	-3.3 (-3.6,-2.9)	-3.1 (-3.5,-2.8)	2.0 (1.8,2.2)	2.0 (1.9,2.2)	-0.1 (-0.3,0.1)	-0.1 (-0.2,0.1)
**L**	**L**	**H**	**L**	-0.7 (-0.8,-0.6)	6.9 (6.8,7.0)	6.9 (6.8,7.0)	-3.1 (-3.5,-2.8)	-0.6 (-0.8,-0.3)	1.2 (1.0,1.3)	1.0 (0.9,1.2)	-1.8 (-1.9,-1.7)	-0.7 (-0.9,-0.5)
**L**	**L**	**H**	**H**	-1.9 (-2.0,-1.7)	7.5 (7.4,7.6)	7.5 (7.4,7.6)	0.1 (-0.1,0.4)	-0.2 (-0.4,0.1)	0.4 (0.2,0.5)	0.6 (0.5,0.8)	-1.8 (-1.9,-1.7)	-1.9 (-2.0,-1.7)
**L**	**H**	**L**	**H**	-1.2 (-1.3,-1.1)	7.6 (7.5,7.7)	6.8 (6.7,6.9)	-1.0 (-1.2,-0.7)	-1.4 (-1.7,-1.1)	1.0 (0.9,1.2)	0.2 (0.1,0.3)	-0.9 (-1.0,-0.7)	-1.5 (-1.6,-1.4)
**Sensitivity: L L L L**												
**Specificity**				π	Se_1	Se_2	Se_3	Se_4	Sp_1	Sp_2	Sp_3	Sp_4
**H**	**H**	**L**	**L**	-3.8 (-3.9,-3.7)	26.4 (26.0,26.8)	26.5 (26.1,26.8)	-1.5 (-1.9,-1.1)	-1.8 (-2.2,-1.4)	2.4 (2.3,2.5)	2.4 (2.3,2.5)	-1.4 (-1.6,-1.)	-1.3 (-1.5,-1.1)
**H**	**H**	**H**	**L**	-6.7 (-6.8,-6.5)	33.5 (33.2,33.7)	33.4 (33.2,33.7)	-2.2 (-2.6,-1.7)	-0.3 (-0.7,0.1)	1.1 (1.0,1.2)	1.0 (0.9,1.1)	-4.4 (-4.5,-4.3)	-1.7 (-1.8,-1.5)

**H**	**H**	**H**	**H**	-7.2 (-7.3,-7.1)	34.6 (34.4,34.8)	34.7 (34.5,34.9)	-1.5 (-1.9,-1.1)	-1.6 (-2.0,-1.2)	0.9 (0.8,1.0)	0.8 (0.7,0.9)	-4.6 (-4.7,-4.5)	-4.5 (-4.6,-4.4)
**L**	**L**	**L**	**L**	4.9 (4.2,5.5)	22.1 (21.5,22.8)	21.5 (20.9,22.2)	-5.3 (-6.0,-4.7)	-5.3 (-6.0,-4.7)	8.4 (8.0,8.8)	8.2 (7.8,8.6)	-0.8 (-1.1,-0.6)	-0.8 (-1.1,-0.6)
**L**	**L**	**H**	**L**	-3.9 (-4.1,-3.6)	32.4 (32.2,32.7)	32.5 (32.2,32.7)	-11.6 (-12.3,-10.8)	-1.3 (-1.8,-0.8)	5.2 (5.0,5.4)	4.9 (4.7,5.2)	-5.1 (-5.2,-4.9)	-1.4 (-1.5,-1.2)
**L**	**L**	**H**	**H**	-6.9 (-7.1,-6.7)	33.1 (32.6,33.6)	33.2 (32.7,33.7)	-2.1 (-2.9,-1.3)	-2.5 (-3.3,-1.7)	3.3 (3.1,3.5)	3.4 (3.1,3.6)	-4.7 (-4.9,-4.6)	-4.8 (-4.9,-4.7)
**L**	**H**	**L**	**H**	-6.2 (-6.3,-6.1)	33.4 (33.2,33.6)	32.6 (32.4,32.8)	-0.9 (-1.3,-0.6)	-4.2 (-4.8,-3.7)	3.8 (3.7,4.0)	1.4 (1.2,1.5)	-1.5 (-1.7,-1.4)	-4.5 (-4.6,-4.4)
**Sensitivity: L L H L**												
**Specificity**				π	Se_1	Se_2	Se_3	Se_4	Sp_1	Sp_2	Sp_3	Sp_4
**H**	**H**	**L**	**L**	-6.2 (-6.3,-6.1)	31.8 (31.6,32.1)	32.1 (31.9,32.4)	-2.4 (-2.7,-2.2)	-0.5 (-0.9,-0.2)	1.2 (1.1,1.3)	1.4 (1.3,1.5)	-4.0 (-4.1,-3.8)	-1.6 (-1.7,-1.4)
**H**	**H**	**H**	**L**	-7.6 (-7.7,-7.5)	35.8 (35.7,36.0)	35.7 (35.6,35.9)	-1.9 (-2.2,-1.6)	0.1 (-0.3,0.5)	0.7 (0.6,0.7)	0.7 (0.6,0.8)	-7.3 (-7.4,-7.2)	-1.7 (-1.8,-1.6)
**H**	**H**	**H**	**H**	-7.5 (-7.6,-7.4)	35.4 (35.3,35.6)	35.4 (35.3,35.6)	-1.6 (-1.9,-1.3)	-0.3 (-0.7,0.1)	0.6 (0.5,0.7)	0.7 (0.6,0.8)	-7.2 (-7.4,-7.1)	-4.5 (-4.7,-4.4)
**L**	**L**	**L**	**L**	-1.8 (-2.1,-1.5)	29.3 (29.0,29.7)	29.4 (29.1,29.8)	-14.0 (-14.6,-13.4)	-2.4 (-3.0,-1.9)	5.9 (5.7,6.1)	6.0 (5.7,6.2)	-4.3 (-4.5,-4.1)	-1.0 (-1.2,-0.9)
**L**	**L**	**H**	**L**	-5.6 (-5.8,-5.4)	35.7 (35.6,35.8)	35.7 (35.6,35.9)	-18.2 (-18.9,-17.6)	-0.5 (-0.9,0.0)	4.7 (4.5,4.9)	4.6 (4.5,4.8)	-8.7 (-8.8,-8.5)	-1.7 (-1.8,-1.5)
**L**	**L**	**H**	**H**	-6.0 (-6.4,-5.7)	28.8 (27.8,29.9)	28.9 (27.9,30.0)	-4.5 (-5.0,-3.9)	2.7 (2.0,3.4)	2.4 (2.2,2.6)	2.4 (2.2,2.6)	-6.5 (-6.8,-6.2)	-3.2 (-3.2,-3.0)
**L**	**H**	**L**	**H**	-6.8 (-6.9,-6.6)	34.2 (34.0,34.4)	34.1 (33.9,34.2)	-2.6 (-2.9,-2.2)	-2.9 (-3.4,-2.5)	3.5 (3.4,3.7)	1.1 (1.0,1.2)	-4.4 (-4.6,-4.2)	-4.5 (-4.6,-4.4)
**Sensitivity: L L H H**												
**Specificity**				π	Se_1	Se_2	Se_3	Se_4	Sp_1	Sp_2	Sp_3	Sp_4
**H**	**H**	**L**	**L**	-7.0 (-7.1,-6.9)	33.7 (33.5,33.9)	33.7 (33.4,33.9)	-1.7 (-2.0,-1.5)	-1.7 (-2.0,-1.4)	1.0 (0.9,1.1)	1.1 (1.0,1.2)	-4.2 (-4.3,-4.0)	-4.3 (-4.5,-4.2)
**H**	**H**	**H**	**L**	-7.5 (-7.6,-7.4)	35.7 (35.6,35.8)	35.6 (35.4,35.7)	-1.5 (-1.8,-1.2)	-1.1 (-1.3,-0.8)	0.6 (0.5,0.7)	0.6 (0.5,0.7)	-7.1 (-7.2,-7.0)	-4.5 (-4.7,-4.4)
**H**	**H**	**H**	**H**	-7.2 (-7.4,-7.1)	33.9 (33.5,34.4)	34.1 (33.7,34.5)	-0.9 (-1.2,-0.6)	-1.0 (-1.3,-0.8)	0.6 (0.5,0.7)	0.6 (0.5,0.7)	-6.9 (-7.1,-6.8)	-6.9 (-7.1,-6.8)
**L**	**L**	**L**	**L**	-4.6 (-4.8,-4.5)	32.4 (32.1,32.7)	32.6 (32.4,32.9)	-7.7 (-8.2,-7.7)	-7.5 (-8.0,-6.9)	4.8 (4.6,5.0)	4.9 (4.7,5.0)	-4.3 (-4.5,-4.2)	-4.4 (-4.5,-4.2)
**L**	**L**	**H**	**L**	-7.1 (-7.3,-7.0)	35.6 (35.4,35.9)	35.7 (35.4,36.0)	-7.6 (-8.2,-7.1)	0.0 (-0.3,0.4)	3.5 (3.3,3.6)	3.4 (3.2,3.6)	-7.9 (-8.1,-7.8)	-4.3 (-4.5,-4.2)
**L**	**L**	**H**	**H**	1.6 (1.3,1.9)	3.0 (2.1,3.8)	2.9 (2.0,3.7)	-3.6 (-4.0,-3.1)	-3.9 (-4.3,-3.4)	0.7 (0.5,0.9)	0.6 (0.4,0.8)	0.4 (0.1,0.6)	0.3 (0.1,0.6)
**L**	**H**	**L**	**H**	-7.6 (-7.7,-7.5)	35.2 (35.1,35.4)	36.1 (36.0,36.3)	-0.6 (-0.9,-0.3)	-2.7 (-3.1,-2.4)	3.1 (3.0,3.3)	0.6 (0.5,0.7)	-4.3 (-4.5,-4.2)	-7.5 (-7.6,-7.4)

Table x.5 Coverages of the 95% CrIs for each model parameter

**Sensitivity: H H L L**												
**Specificity**				π	Se_1	Se_2	Se_3	Se_4	Sp_1	Sp_2	Sp_3	Sp_4
**H**	**H**	**L**	**L**	95.1 (93.7,96.5)	100.0 (100.0,100.0)	100.0 (100.0,100.0)	94.5 (93.0,95.9)	95.4 (94.1,96.7)	91.3 (89.5,93.0)	88.4 (86.4,90.5)	94.7 (93.3,96.1)	94.3 (92.8,95.7)
**H**	**H**	**H**	**L**	97.2 (96.2,98.2)	80.6 (78.1,83.0)	81.5 (79.1,83.9)	94.4 (93.0,95.8)	95.5 (94.2,96.8)	92.8 (91.2,94.4)	94.3 (92.8,95.7)	92.4 (90.7,94.0)	94.6 (93.2,96.0)
**H**	**H**	**H**	**H**	92.5 (90.8,94.1)	51.6 (48.5,54.7)	53.3 (50.2,56.4)	94.9 (93.5,96.3)	94.4 (93.0,95.8)	94.8 (93.4,96.2)	95.0 (93.6,96.3)	91.2 (89.4,92.9)	89.1 (87.1,91.0)
**L**	**L**	**L**	**L**	83.4 (80.2,86.6)	99.6 (99.1,100.0)	99.4 (98.8,100.0)	94.0 (92.0,96.1)	92.1 (89.8,94.4)	83.8 (80.6,87.0)	86.9 (84.0,89.8)	95.4 (93.6,97.2)	96.3 (94.7,98.0)
**L**	**L**	**H**	**L**	97.0 (95.9,98.1)	97.9 (97.0,98.8)	98.5 (97.7,99.3)	92.5 (90.8,94.2)	95.9 (94.6,97.1)	92.8 (91.1,94.4)	92.7 (91.0,94.3)	96.6 (95.5,97.8)	95.9 (94.6,97.1)
**L**	**L**	**H**	**H**	95.4 (94.1,96.7)	82.9 (80.5,85.2)	82.9 (80.5,85.2)	95.0 (93.6,96.3)	93.2 (91.6,94.7)	94.4 (92.9,95.8)	94.1 (92.6,95.5)	93.3 (91.7,94.8)	93.6 (92.0,95.1)
**L**	**H**	**L**	**H**	97.0 (95.9,98.1)	72.5 (69.7,75.2)	99.0 (98.4,99.6)	95.4 (94.1,96.7)	93.6 (92.1,95.1)	91.4 (89.7,93.1)	94.5 (93.1,95.9)	95.4 (94.1,96.7)	94.9 (93.5,96.3)

**Sensitivity: H H H L**												
**Specificity**				π	Se_1	Se_2	Se_3	Se_4	Sp_1	Sp_2	Sp_3	Sp_4
**H**	**H**	**L**	**L**	98.3 (97.5,99.1)	92.5 (90.9,94.1)	92.0 (90.3,93.7)	93.9 (92.4,95.4)	93.9 (92.4,95.4)	93.0 (91.4,94.6)	93.1 (91.5,94.7)	95.7 (94.4,97.0)	95.1 (93.8,96.4)
**H**	**H**	**H**	**L**	90.7 (88.9,92.5)	32.4 (29.5,35.3)	29.9 (27.1,32.7)	95.0 (93.6,96.4)	96.3 (95.1,97.5)	95.6 (94.3,96.9)	94.6 (93.2,96.0)	81.3 (78.9,83.7)	95.6 (94.3,96.9)
**H**	**H**	**H**	**H**	89.6 (87.7,91.5)	20.9 (18.4,23.4)	23.8 (21.2,26.4)	94.1 (92.6,95.6)	94.5 (93.1,95.9)	95.5 (94.2,96.8)	94.3 (92.9,95.7)	82.4 (80.0,84.8)	86.1 (84.0,88.2)
**L**	**L**	**L**	**L**	91.4 (89.6,93.3)	99.8 (99.5,100.0)	99.7 (99.3,100.0)	90.3 (88.4,92.3)	94.2 (92.7,95.7)	86.1 (83.8,88.3)	87.4 (85.2,89.5)	96.8 (95.7,98.0)	95.9 (94.7,97.2)
**L**	**L**	**H**	**L**	98.1 (97.2,98.9)	67.6 (64.7,70.5)	67.8 (64.9,70.7)	93.0 (91.5,94.6)	95.3 (93.9,96.6)	92.0 (90.3,93.7)	92.8 (91.2,94.4)	90.1 (88.3,92.0)	94.3 (92.9,95.8)
**L**	**L**	**H**	**H**	94.1 (92.6,95.6)	47.3 (44.3,50.4)	43.8 (40.8,46.9)	96.5 (95.4,97.6)	95.2 (93.9,96.5)	94.1 (92.6,95.6)	94.8 (93.4,96.2)	82.5 (80.1,84.8)	90.3 (88.5,92.1)
**L**	**H**	**L**	**H**	96.0 (94.8,97.2)	51.9 (48.8,55.0)	94.5 (93.1,95.9)	94.6 (93.2,96.0)	94.0 (92.5,95.5)	91.8 (90.1,93.5)	95.2 (93.9,96.5)	94.7 (93.3,96.1)	91.5 (89.8,93.2)
**Sensitivity: H H H H**												
**Specificity**				π	Se_1	Se_2	Se_3	Se_4	Sp_1	Sp_2	Sp_3	Sp_4
**H**	**H**	**L**	**L**	94.4 (93.0,95.8)	69.5 (66.6,72.4)	70.5 (67.7,73.3)	93.7 (92.2,95.2)	94.0 (92.5,95.5)	93.6 (92.1,95.1)	93.8 (92.3,95.3)	94.2 (92.8,95.6)	93.4 (91.9,94.9)
**H**	**H**	**H**	**L**	88.1 (86.1,90.1)	20.8 (18.3,23.3)	22.8 (20.2,25.4)	94.5 (93.1,95.9)	94.8 (93.4,96.2)	94.2 (93.3,96.1)	95.0 (93.6,96.3)	81.3 (78.9,83.7)	91.2 (89.4,92.9)
**H**	**H**	**H**	**H**	85.8 (83.6,87.9)	12.5 (10.5,14.6)	11.9 (9.9,13.9)	94.9 (93.5,96.3)	93.8 (92.3,95.3)	94.7 (93.3,96.1)	94.3 (92.8,95.7)	77.7 (75.1,80.2)	75.5 (72.8,78.1)
**L**	**L**	**L**	**L**	96.9 (95.8,98.0)	96.8 (95.7,97.9)	96.2 (95.0,97.4)	94.2 (92.8,95.7)	94.4 (93.0,95.9)	91.7 (90.0,93.4)	91.9 (90.2,93.6)	96.7 (95.5,97.8)	95.6 (94.3,96.8)
**L**	**L**	**H**	**L**	97.1 (96.1,98.1)	50.2 (47.0,53.3)	49.0 (45.9,52.1)	96.0 (94.8,97.2)	96.9 (95.8,98.0)	93.4 (91.9,94.9)	92.0 (90.3,93.7)	82.6 (80.2,84.9)	96.4 (95.2,97.6)
**L**	**L**	**H**	**H**	88.3 (86.3,90.3)	29.0 (26.1,31.8)	29.2 (26.2,32.0)	97.0 (95.9,98.1)	97.1 (96.1,98.1)	95.3 (94.0,96.6)	95.1 (93.7,96.4)	82.0 (79.6,84.3)	80.2 (77.7,82.6)
**L**	**H**	**L**	**H**	92.8 (91.2,94.4)	19.3 (16.9,21.7)	56.8 (53.7,59.9)	95.2 (93.9,96.5)	96.0 (94.8,97.2)	93.5 (92.0,95.0)	93.8 (92.3,95.3)	94.4 (93.0,95.8)	86.5 (84.4,88.6)
**Sensitivity: L L L L**												
**Specificity**				π	Se_1	Se_2	Se_3	Se_4	Sp_1	Sp_2	Sp_3	Sp_4
**H**	**H**	**L**	**L**	92.3 (90.5,94.0)	22.7 (20.0,25.4)	21.3 (18.6,23.9)	93.9 (92.4,95.5)	94.8 (93.4,96.3)	85.1 (82.8,87.4)	85.7 (83.4,87.9)	93.2 (91.5,94.8)	91.5 (89.7,93.3)
**H**	**H**	**H**	**L**	16.9 (14.5,19.2)	1.0 (0.4,1.6)	0.7 (0.2,1.2)	94.0 (92.5,95.5)	96.3 (95.1,97.5)	90.3 (88.4,92.1)	92.2 (90.5,93.8)	23.2 (20.6,25.8)	89.1 (87.1,91.0)
**H**	**H**	**H**	**H**	3.2 (2.1,4.3)	0.2 (0.0,0.5)	0.1 (0.0,0.3)	94.7 (93.3,96.1)	94.0 (92.5,95.5)	92.3 (90.6,94.0)	92.4 (90.8,94.0)	17.1 (14.8,19.4)	21.6 (19.0,24.2)
**L**	**L**	**L**	**L**	99.5 (98.8,100.0)	65.3 (60.6,69.9)	69.2 (64.7,73.7)	88.7 (85.6,91.8)	87.9 (84.8,91.1)	49.3 (44.4,54.1)	51.0 (46.1,55.8)	97.0 (95.4,98.7)	95.3 (93.3,97.4)
**L**	**L**	**H**	**L**	93.6 (91.9,95.3)	6.1 (4.4,7.7)	5.9 (4.3,7.6)	82.8 (80.2,85.4)	95.8 (94.4,97.2)	65.1 (61.9,68.4)	66.1 (62.9,69.4)	25.6 (22.6,28.6)	91.7 (89.8,93.6)
**L**	**L**	**H**	**H**	59.3 (55.9,62.7)	10.5 (8.3,12.6)	10.5 (8.3,12.6)	93.4 (91.7,95.1)	92.9 (91.1,94.7)	79.6 (76.8,82.4)	77.3 (74.4,80.2)	32.4 (29.1,35.6)	30.1 (27.0,33.3)
**L**	**H**	**L**	**H**	50.3 (47.1,53.4)	0.0 (0.0,0.0)	4.6 (3.3,6.0)	96.4 (95.2,97.5)	90.4 (88.6,92.2)	68.0 (65.1,70.9)	92.1 (90.5,93.8)	91.5 (89.8,93.3)	24.5 (21.8,27.2)
**Sensitivity: L L H L**												
**Specificity**				π	Se_1	Se_2	Se_3	Se_4	Sp_1	Sp_2	Sp_3	Sp_4
**H**	**H**	**L**	**L**	25.3 (22.6,28.0)	1.3 (0.6,2.0)	0.6 (0.1,1.1)	93.5 (91.9,95.0)	94.7 (93.3,96.1)	91.6 (89.8,93.3)	89.9 (88.0,91.7)	63.1 (60.1,66.0)	89.3 (87.3,91.2)
**H**	**H**	**H**	**L**	0.3 (0.0,0.6)	0.0 (0.0,0.0)	0.0 (0.0,0.0)	94.9 (93.5,96.3)	95.2 (93.9,96.5)	93.2 (91.6,94.8)	93.9 (92.4,95.4)	0.6 (0.1,1.1)	88.1 (86.1,90.1)
**H**	**H**	**H**	**H**	0.8 (0.2,1.4)	0.0 (0.0,0.0)	0.0 (0.0,0.0)	94.7 (93.3,96.1)	96.7 (95.6,97.8)	92.2 (90.5,93.9)	93.4 (91.9,94.9)	1.1 (0.5,1.7)	18.8 (16.4,21.2)
**L**	**L**	**L**	**L**	99.0 (98.3,99.7)	18.0 (15.1,20.8)	19.6 (16.6,22.5)	76.5 (73.4,79.7)	95.3 (93.7,96.8)	57.8 (54.2,61.5)	58.3 (54.6,61.9)	73.8 (70.5,77.1)	96.7 (95.4,98.0)
**L**	**L**	**H**	**L**	66.8 (63.8,69.8)	0.4 (0.0,0.8)	0.4 (0.0,0.8)	74.6 (71.9,77.3)	95.8 (94.5,97.0)	63.8 (60.8,66.8)	63.0 (60.0,66.0)	0.8 (0.3,1.4)	91.0 (89.2,92.8)
**L**	**L**	**H**	**H**	43.3 (39.5,47.0)	25.6 (22.3,28.9)	25.4 (22.1,28.8)	97.2 (95.9,98.4)	94.6 (92.9,96.3)	83.7 (80.9,86.5)	83.8 (81.0,86.6)	29.0 (25.6,32.5)	49.0 (45.2,52.7)
**L**	**H**	**L**	**H**	21.8 (19.2,24.4)	0.3 (0.0,0.6)	1.3 (0.6,2.0)	93.8 (92.3,95.3)	94.2 (92.7,95.6)	70.5 (67.6,73.3)	92.2 (90.5,93.8)	56.2 (53.1,59.3)	22.6 (20.0,25.2)
**Sensitivity: L L H H**												
**Specificity**				π	Se_1	Se_2	Se_3	Se_4	Sp_1	Sp_2	Sp_3	Sp_4
**H**	**H**	**L**	**L**	5.5 (4.1,6.9)	0.2 (0.0,0.5)	0.2 (0.0,0.5)	93.9 (92.4,95.4)	92.9 (91.3,94.5)	90.3 (88.5,92.1)	91.6 (89.9,93.3)	58.6 (55.5,61.7)	54.9 (51.8,58.0)

**H**	**H**	**H**	**L**	1.2 (0.5,1.9)	0.0 (0.0,0.0)	0.0 (0.0,0.0)	94.5 (93.1,95.9)	95.8 (94.6,97.0)	92.7 (91.1,94.3)	92.6 (91.0,94.2)	1.2 (0.5,1.9)	52.2 (49.1,55.2)
**H**	**H**	**H**	**H**	5.5 (4.0,7.0)	4.6 (3.3,6.0)	4.6 (3.3,6.0)	94.4 (92.9,95.9)	94.5 (93.0,96.0)	94.7 (93.3,96.2)	93.5 (92.0,95.1)	6.9 (5.3,8.5)	6.9 (5.3,8.5)
**L**	**L**	**L**	**L**	87.9 (85.8,90.0)	5.2 (3.7,6.6)	3.9 (2.6,5.2)	90.6 (88.7,92.5)	88.9 (86.9,91.0)	62.6 (59.4,65.8)	61.8 (58.6,65.0)	64.9 (61.8,68.1)	67.7 (64.7,70.8)
**L**	**L**	**H**	**L**	23.5 (20.6,26.4)	2.8 (1.7,3.9)	2.4 (1.4,3.5)	92.9 (91.2,94.7)	97.7 (96.6,98.7)	74.4 (71.5,77.4)	74.8 (71.8,77.8)	3.9 (2.6,5.2)	57.9 (54.6,61.3)
**L**	**L**	**H**	**H**	91.8 (89.8,93.9)	87.6 (85.2,90.0)	88.0 (85.7,90.4)	98.9 (98.1,99.7)	99.2 (98.5,99.8)	94.7 (93.0,96.3)	94.4 (92.7,96.1)	91.6 (89.5,93.6)	93.1 (91.2,95.0)
**L**	**H**	**L**	**H**	3.7 (2.5,4.9)	0.3 (0.0,0.6)	0.3 (0.0,0.6)	97.1 (96.0,98.1)	95.9 (94.6,97.1)	75.3 (72.6,78.0)	94.8 (93.4,96.2)	57.4 (54.3,60.5)	1.3 (0.6,2.0)

xi. Setting 11: $\pi = 0.2, \omega = 0.5, n_{obs} = 2000$

Table xi.1 Number of converged data sets for different sensitivity-specificity combinations

				**Number of converged data sets**
**Specificity**				**Sensitivity: H H L L**
**H**	**H**	**L**	**L**	877
**H**	**H**	**H**	**L**	1000
**H**	**H**	**H**	**H**	999
**L**	**L**	**L**	**L**	485
**L**	**L**	**H**	**L**	950
**L**	**L**	**H**	**H**	997
**L**	**H**	**L**	**H**	993
**Specificity**				**Sensitivity: H H H L**
**H**	**H**	**L**	**L**	998
**H**	**H**	**H**	**L**	1000
**H**	**H**	**H**	**H**	1000
**L**	**L**	**L**	**L**	904
**L**	**L**	**H**	**L**	971
**L**	**L**	**H**	**H**	999
**L**	**H**	**L**	**H**	1000
**Specificity**				**Sensitivity: H H H H**
**H**	**H**	**L**	**L**	1000
**H**	**H**	**H**	**L**	1000
**H**	**H**	**H**	**H**	1000
**L**	**L**	**L**	**L**	977
**L**	**L**	**H**	**L**	997
**L**	**L**	**H**	**H**	1000
**L**	**H**	**L**	**H**	1000
**Specificity**				**Sensitivity: L L L L**
**H**	**H**	**L**	**L**	646

**H**	**H**	**H**	**L**	984
**H**	**H**	**H**	**H**	993
**L**	**L**	**L**	**L**	161
**L**	**L**	**H**	**L**	845
**L**	**L**	**H**	**H**	654
**L**	**H**	**L**	**H**	917
**Specificity**				**Sensitivity: L L H L**
**H**	**H**	**L**	**L**	979
**H**	**H**	**H**	**L**	1000
**H**	**H**	**H**	**H**	1000
**L**	**L**	**L**	**L**	707
**L**	**L**	**H**	**L**	945
**L**	**L**	**H**	**H**	767
**L**	**H**	**L**	**H**	941
**Specificity**				**Sensitivity: L L H H**
**H**	**H**	**L**	**L**	999
**H**	**H**	**H**	**L**	998
**H**	**H**	**H**	**H**	1000
**L**	**L**	**L**	**L**	827
**L**	**L**	**H**	**L**	687
**L**	**L**	**H**	**H**	849
**L**	**H**	**L**	**H**	948

Table xi.2 Percentages of the time 95% credible intervals (CrIs) for residual correlations did not included '0' for any pairs, and percentages of the time that the overall χ^2 or G^2 statistic indicated a lack of overall fit

**Sensitivity: H H L L**						
**Specificity**				**Res. Cor.**	**Overall G^2**	**Overall χ^2**
**H**	**H**	**L**	**L**	61.5 (58.1,64.7)	6.4 (4.9,8.2)	6.6 (5.1,8.5)
**H**	**H**	**H**	**L**	22.4 (19.9,25.1)	6.6 (5.1,8.3)	6.2 (4.8,7.9)
**H**	**H**	**H**	**H**	18.9 (16.5,21.5)	17.3 (15.0,19.8)	18.6 (16.3,21.2)
**L**	**L**	**L**	**L**	46.8 (42.3,51.4)	11.3 (8.7,14.5)	11.1 (8.5,14.3)
**L**	**L**	**H**	**L**	24.7 (22.0,27.6)	12.0 (10.0,14.2)	11.8 (9.8,14.0)
**L**	**L**	**H**	**H**	11.4 (9.5,13.6)	37.9 (34.9,41.0)	43.9 (40.8,47.1)
**L**	**H**	**L**	**H**	35.1 (32.2,38.2)	9.9 (8.1,11.9)	9.5 (7.7,11.5)
**Sensitivity: H H H L**						
**Specificity**				**Res. Cor.**	**Overall G^2**	**Overall χ^2**
**H**	**H**	**L**	**L**	31.8 (28.9,34.8)	5.9 (4.5,7.6)	5.9 (4.5,7.6)
**H**	**H**	**H**	**L**	7.5 (5.9,9.3)	7.3 (5.8,9.1)	6.2 (4.8,7.9)
**H**	**H**	**H**	**H**	15.2 (13.0,17.6)	35.7 (32.7,38.8)	38.7 (35.7,41.8)

**L**	**L**	**L**	**L**	34.5 (31.4,37.7)	10.4 (8.5,12.6)	10.5 (8.6,12.7)
**L**	**L**	**H**	**L**	37.3 (34.2,40.4)	15.2 (13.0,17.7)	15.0 (12.8,17.4)
**L**	**L**	**H**	**H**	27.3 (24.6,30.2)	68.3 (65.3,71.1)	76.7 (73.9,79.3)
**L**	**H**	**L**	**H**	33.6 (30.7,36.6)	12.9 (10.9,15.1)	14.0 (11.9,16.3)
**Sensitivity: H H H H**						
**Specificity**				**Res. Cor.**	**Overall G^2**	**Overall χ^2**
**H**	**H**	**L**	**L**	33.1 (30.2,36.1)	6.2 (4.8,7.9)	5.8 (4.4,7.4)
**H**	**H**	**H**	**L**	28.4 (25.6,31.3)	16.5 (14.3,18.9)	15.3 (13.1,17.7)
**H**	**H**	**H**	**H**	46.8 (43.7,49.9)	72.9 (70.0,75.6)	77.3 (74.6,79.9)
**L**	**L**	**L**	**L**	30.8 (27.9,33.8)	13.8 (11.7,16.1)	14.2 (12.1,16.6)
**L**	**L**	**H**	**L**	44.7 (41.6,47.9)	42.2 (39.1,45.4)	44.7 (41.6,47.9)
**L**	**L**	**H**	**H**	48.8 (45.7,51.9)	93.5 (91.8,94.9)	96.2 (94.8,97.3)
**L**	**H**	**L**	**H**	42.8 (39.7,45.9)	24.8 (22.2,27.6)	26.7 (24.0,29.6)
**Sensitivity: L L L L**						
**Specificity**				**Res. Cor.**	**Overall G^2**	**Overall χ^2**
**H**	**H**	**L**	**L**	65.0 (61.2,68.7)	6.2 (4.5,8.3)	5.7 (4.1,7.8)
**H**	**H**	**H**	**L**	59.5 (56.3,62.5)	16.9 (14.6,19.4)	16.4 (14.1,18.8)
**H**	**H**	**H**	**H**	96.1 (94.7,97.2)	97.1 (95.8,98.0)	97.9 (96.8,98.7)
**L**	**L**	**L**	**L**	74.5 (67.1,81.1)	23.0 (16.7,30.3)	24.8 (18.4,32.3)
**L**	**L**	**H**	**L**	90.5 (88.4,92.4)	73.8 (70.7,76.8)	74.7 (71.6,77.6)
**L**	**L**	**H**	**H**	97.1 (95.5,98.2)	100.0 (99.4,100.0)	100.0 (99.4,100.0)
**L**	**H**	**L**	**H**	87.7 (85.4,89.7)	45.3 (42.0,48.5)	45.9 (42.6,49.2)
**Sensitivity: L L H L**						
**Specificity**				**Res. Cor.**	**Overall G^2**	**Overall χ^2**
**H**	**H**	**L**	**L**	61.7 (58.6,64.8)	10.0 (8.2,12.1)	9.7 (7.9,11.7)
**H**	**H**	**H**	**L**	72.1 (69.2,74.9)	37.5 (34.5,40.6)	37.4 (34.4,40.5)
**H**	**H**	**H**	**H**	97.6 (96.4,98.5)	100.0 (99.6,100.0)	100.0 (99.6,100.0)
**L**	**L**	**L**	**L**	85.9 (83.1,88.3)	54.3 (50.6,58.0)	55.0 (51.3,58.7)
**L**	**L**	**H**	**L**	98.5 (97.5,99.2)	96.5 (95.1,97.6)	96.7 (95.4,97.8)
**L**	**L**	**H**	**H**	100.0 (99.5,100.0)	100.0 (99.5,100.0)	100.0 (99.5,100.0)
**L**	**H**	**L**	**H**	99.5 (98.8,99.8)	98.0 (96.9,98.8)	98.3 (97.3,99.0)
**Sensitivity: L L H H**						
**Specificity**				**Res. Cor.**	**Overall G^2**	**Overall χ^2**
**H**	**H**	**L**	**L**	96.5 (95.2,97.5)	51.2 (48.0,54.3)	51.1 (47.9,54.2)
**H**	**H**	**H**	**L**	100.0 (99.6,100.0)	99.8 (99.3,100.0)	99.8 (99.3,100.0)
**H**	**H**	**H**	**H**	100.0 (99.6,100.0)	100.0 (99.6,100.0)	100.0 (99.6,100.0)
**L**	**L**	**L**	**L**	99.5 (98.8,99.9)	98.4 (97.3,99.2)	98.8 (97.8,99.4)
**L**	**L**	**H**	**L**	99.6 (98.7,99.9)	100.0 (99.5,100.0)	100.0 (99.5,100.0)
**L**	**L**	**H**	**H**	100.0 (99.6,100.0)	100.0 (99.6,100.0)	100.0 (99.6,100.0)
**L**	**H**	**L**	**H**	99.7 (99.1,99.9)	100.0 (99.6,100.0)	100.0 (99.6,100.0)

Table xi.3 Percentages of the time 95% CrIs for residual correlations did not included ‘0’ for each pair, and percentages of the time the pairwise χ^2 or G^2 statistic for each pair indicated a lack of pairwise fit

Sensitivity: H H L L										
**Specificity**				**Tool**	**T_1&T_2**	**T_1&T_3**	**T_1&T_4**	**T_2&T_3**	**T_2&T_4**	**T_3&T_4**
**H**	**H**	**L**	**L**	**Res. Cor.**	0.0 (0.0,0.4)	0.1 (0.0,0.6)	0.2 (0.0,0.8)	0.1 (0.0,0.6)	0.1 (0.0,0.6)	61.3 (58.0,64.6)
				**Pairwise G^2**	0.0 (0.0,0.4)	0.0 (0.0,0.4)	0.1 (0.0,0.6)	0.0 (0.0,0.4)	0.0 (0.0,0.4)	4.0 (2.8,5.5)
				**Pairwise χ^2**	0.0 (0.0,0.4)	0.0 (0.0,0.4)	0.1 (0.0,0.6)	0.0 (0.0,0.4)	0.0 (0.0,0.4)	4.0 (2.8,5.5)
**H**	**H**	**H**	**L**	**Res. Cor.**	0.0 (0.0,0.4)	0.0 (0.0,0.4)	0.2 (0.0,0.7)	0.0 (0.0,0.4)	0.1 (0.0,0.6)	22.2 (19.7,24.9)
				**Pairwise G^2**	0.0 (0.0,0.4)	0.0 (0.0,0.4)	0.0 (0.0,0.4)	0.0 (0.0,0.4)	0.0 (0.0,0.4)	2.2 (1.4,3.3)
				**Pairwise χ^2**	0.0 (0.0,0.4)	0.0 (0.0,0.4)	0.0 (0.0,0.4)	0.0 (0.0,0.4)	0.0 (0.0,0.4)	2.2 (1.4,3.3)
**H**	**H**	**H**	**H**	**Res. Cor.**	0.0 (0.0,0.4)	0.0 (0.0,0.4)	0.0 (0.0,0.4)	0.0 (0.0,0.4)	0.0 (0.0,0.4)	18.9 (16.5,21.5)
				**Pairwise G^2**	0.0 (0.0,0.4)	0.0 (0.0,0.4)	0.0 (0.0,0.4)	0.0 (0.0,0.4)	0.0 (0.0,0.4)	4.7 (3.5,6.2)
				**Pairwise χ^2**	0.0 (0.0,0.4)	0.0 (0.0,0.4)	0.0 (0.0,0.4)	0.0 (0.0,0.4)	0.0 (0.0,0.4)	4.7 (3.5,6.2)
**L**	**L**	**L**	**L**	**Res. Cor.**	0.0 (0.0,0.8)	2.5 (1.3,4.3)	3.3 (1.9,5.3)	2.9 (1.6,4.8)	3.3 (1.9,5.3)	39.6 (35.2,44.1)
				**Pairwise G^2**	0.0 (0.0,0.8)	0.0 (0.0,0.8)	0.0 (0.0,0.8)	0.2 (0.0,1.1)	0.2 (0.0,1.1)	2.9 (1.6,4.8)
				**Pairwise χ^2**	0.0 (0.0,0.8)	0.0 (0.0,0.8)	0.2 (0.0,1.1)	0.2 (0.0,1.1)	0.2 (0.0,1.1)	2.9 (1.6,4.8)
**L**	**L**	**H**	**L**	**Res. Cor.**	0.0 (0.0,0.4)	0.1 (0.0,0.6)	10.3 (8.5,2.4)	0.2 (0.0,0.8)	9.9 (8.1,12.0)	8.1 (6.4,10.0)
				**Pairwise G^2**	0.0 (0.0,0.4)	0.1 (0.0,0.6)	1.4 (0.7,2.3)	0.0 (0.0,0.4)	1.1 (0.5,1.9)	2.2 (1.4,3.4)
				**Pairwise χ^2**	0.0 (0.0,0.4)	0.1 (0.0,0.6)	1.4 (0.7,2.3)	0.0 (0.0,0.4)	1.1 (0.5,1.9)	2.2 (1.4,3.4)
**L**	**L**	**H**	**H**	**Res. Cor.**	4.0 (2.9,5.4)	0.6 (0.2,1.3)	0.7 (0.3,1.4)	0.8 (0.3,1.6)	0.8 (0.3,1.6)	5.8 (4.4,7.5)
				**Pairwise G^2**	0.7 (0.3,1.4)	0.0 (0.0,0.4)	0.0 (0.0,0.4)	0.1 (0.0,0.6)	0.1 (.0,0.6)	3.1 (2.1,4.4)
				**Pairwise χ^2**	0.7 (0.3,1.4)	0.0 (0.0,0.4)	0.0 (0.0,0.4)	0.1 (0.0,0.6)	0.2 (0.0,0.7)	3.1 (2.1,4.4)
**L**	**H**	**L**	**H**	**Res. Cor.**	0.0 (0.0,0.4)	20.6 (18.2,23.3)	2.3 (1.5,3.5)	0.0 (0.0,0.4)	0.0 (0.0,0.4)	17.2 (14.9,19.7)
				**Pairwise G^2**	0.0 (0.0,0.4)	1.8 (1.1,2.8)	0.1 (0.0,0.6)	0.0 (0.0,0.4)	0.0 (0.0,0.4)	2.3 (1.5,3.5)
				**Pairwise χ^2**	0.0 (0.0,0.4)	1.8 (1.1,2.8)	0.2 (0.0,0.7)	0.0 (0.0,0.4)	0.0 (0.0,0.4)	2.3 (1.5,3.5)
**Sensitivity: H H H L**										
**Specificity**				**Tool**	**T_1&T_2**	**T_1&T_3**	**T_1&T_4**	**T_2&T_3**	**T_2&T_4**	**T_3&T_4**
**H**	**H**	**L**	**L**	**Res. Cor.**	0.0 (0.0,0.4)	0.1 (0.0,0.6)	0.2 (0.0,0.7)	0.0 (0.0,0.4)	0.1 (0.0,0.6)	31.5 (28.6,34.4)
				**Pairwise G^2**	0.0 (0.0,0.4)	0.0 (0.0,0.4)	0.0 (0.0,0.4)	0.0 (0.0,0.4)	0.0 (0.0,0.4)	3.2 (2.2,4.5)
				**Pairwise χ^2**	0.0 (0.0,0.4)	0.0 (0.0,0.4)	0.0 (0.0,0.4)	0.0 (0.0,0.4)	0.0 (0.0,0.4)	3.2 (2.2,4.5)
**H**	**H**	**H**	**L**	**Res. Cor.**	0.0 (0.0,0.4)	0.0 (0.0,0.4)	0.6 (0.2,1.3)	0.0 (0.0,0.4)	0.8 (0.3,1.6)	6.2 (4.8,7.9)
				**Pairwise G^2**	0.0 (0.0,0.4)	0.0 (0.0,0.4)	0.1 (0.0,0.6)	0.0 (0.0,0.4)	0.0 (0.0,0.4)	1.3 (0.7,2.2)
				**Pairwise χ^2**	0.0 (0.0,0.4)	0.0 (0.0,0.4)	0.1 (0.0,0.6)	0.0 (0.0,0.4)	0.0 (0.0,0.4)	1.3 (0.7,2.2)
**H**	**H**	**H**	**H**	**Res. Cor.**	0.0 (0.0,0.4)	0.0 (0.0,0.4)	0.0 (0.0,0.4)	0.0 (0.0,0.4)	0.0 (0.0,0.4)	15.2 (13.0,17.6)
				**Pairwise G^2**	0.0 (0.0,0.4)	0.0 (0.0,0.4)	0.0 (0.0,0.4)	0.0 (0.0,0.4)	0.0 (0.0,0.4)	7.2 (5.7,9.0)
				**Pairwise χ^2**	0.0 (0.0,0.4)	0.0 (0.0,0.4)	0.0 (0.0,0.4)	0.0 (0.0,0.4)	0.0 (0.0,0.4)	7.0 (5.5,8.8)
**L**	**L**	**L**	**L**	**Res. Cor.**	0.0 (0.0,0.4)	1.4 (0.8,2.4)	9.6 (7.8,11.7)	1.4 (0.8,2.4)	10.8 (8.9,13.1)	17.6 (15.2,20.2)
				**Pairwise G^2**	0.0 (0.0,0.4)	0.0 (0.0,0.4)	1.7 (0.9,2.7)	0.1 (0.0,0.6)	1.0 (0.5,1.9)	1.8 (1.0,2.9)
				**Pairwise χ^2**	0.0 (0.0,0.4)	0.0 (0.0,0.4)	1.7 (0.9,2.7)	0.1 (0.0,0.6)	1.0 (0.5,1.9)	1.8 (1.0,2.9)
**L**	**L**	**H**	**L**	**Res. Cor.**	0.3 (0.1,0.9)	0.0 (0.0,0.4)	20.8 (18.3,23.5)	0.0 (0.0,0.4)	18.6 (16.2,21.2)	3.8 (2.7,5.2)

				**Pairwise G^2**	0.0 (0.0,0.4)	0.0 (0.0,0.4)	2.0 (1.2,3.0)	0.0 (0.0,0.4)	2.0 (1.2,3.0)	0.8 (0.4,1.6)
				**Pairwise χ^2**	0.0 (0.0,0.4)	0.0 (0.0,0.4)	2.0 (1.2,3.0)	0.0 (0.0,0.4)	1.9 (1.1,2.9)	0.8 (0.4,1.6)
**L**	**L**	**H**	**H**	**Res. Cor.**	19.4 (17.0,22.00)	0.4 (0.1,1.0)	3.2 (2.2,4.5)	0.5 (0.2,1.2)	3.0 (2.0,4.3)	7.2 (5.7,9.0)
				**Pairwise G^2**	4.1 (3.0,5.5)	0.1 (0.0,0.6)	0.2 (0.0,0.7)	0.0 (0.0,0.4)	0.2 (0.0,0.7)	5.8 (4.4,7.4)
				**Pairwise χ^2**	3.9 (2.8,5.3)	0.1 (0.0,0.6)	0.3 (0.1,0.9)	0.0 (0.0,0.4)	0.5 (0.2,1.2)	5.6 (4.3,7.2)
**L**	**H**	**L**	**H**	**Res. Cor.**	0.1 (0.0,0.6)	20.1 (17.7,22.7)	3.3 (2.3,4.6)	0.0 (0.0,0.4)	0.0 (0.0,0.4)	15.2 (13.0,17.6)
				**Pairwise G^2**	0.1 (0.0,0.6)	1.6 (0.9,2.6)	0.3 (0.1,0.9)	0.0 (0.0,0.4)	0.0 (0.0,0.4)	5.3 (4.0,6.9)
				**Pairwise χ^2**	0.1 (0.0,0.6)	1.7 (1.0,2.7)	0.3 (0.1,0.9)	0.0 (0.0,0.4)	0.0 (0.0,0.4)	5.1 (3.8,6.7)
**Sensitivity: H H H H**										
**Specificity**				**Tool**	**T_1&T_2**	**T_1&T_3**	**T_1&T_4**	**T_2&T_3**	**T_2&T_4**	**T_3&T_4**
**H**	**H**	**L**	**L**	**Res. Cor.**	0.0 (0.0,0.4)	0.2 (0.0,0.7)	0.3 (0.1,0.9)	0.1 (0.0,0.6)	0.0 (0.0,0.4)	32.8 (29.9,35.8)
				**Pairwise G^2**	0.0 (0.0,0.4)	0.0 (0.0,0.4)	0.0 (0.0,0.4)	0.0 (0.0,0.4)	0.0 (0.0,0.4)	5.3 (4.0,6.9)
				**Pairwise χ^2**	0.0 (0.0,0.4)	0.0 (0.0,0.4)	0.0 (0.0,0.4)	0.0 (0.0,0.4)	0.0 (0.0,0.4)	5.3 (4.0,6.9)
**H**	**H**	**H**	**L**	**Res. Cor.**	0.0 (0.0,0.4)	0.0 (0.0,0.4)	1.1 (0.6,2.0)	0.0 (0.0,0.4)	1.0 (0.5,1.8)	26.8 (24.1,29.7)
				**Pairwise G^2**	0.0 (0.0,0.4)	0.0 (0.0,0.4)	0.3 (0.1,0.9)	0.0 (0.0,0.4)	0.3 (0.1,0.9)	10.4 (8.6,12.5)
				**Pairwise χ^2**	0.0 (0.0,0.4)	0.0 (0.0,0.4)	0.3 (0.1,0.9)	0.0 (0.0,0.4)	0.3 (0.1,0.9)	9.9 (8.1,11.9)
**H**	**H**	**H**	**H**	**Res. Cor.**	0.0 (0.0,0.4)	0.0 (0.0,0.4)	0.1 (0.0,0.6)	0.0 (0.0,0.4)	0.0 (0.0,0.4)	46.8 (43.7,49.9)
				**Pairwise G^2**	0.0 (0.0,0.4)	0.0 (0.0,0.4)	0.0 (0.0,0.4)	0.0 (0.0,0.4)	0.0 (0.0,0.4)	33.1 (30.2,36.1)
				**Pairwise χ^2**	0.0 (0.0,0.4)	0.0 (0.0,0.4)	0.0 (0.0,0.4)	0.0 (0.0,0.4)	0.0 (0.0,0.4)	31.4 (28.5,34.4)
**L**	**L**	**L**	**L**	**Res. Cor.**	2.1 (1.3,3.3)	4.8 (3.6,6.3)	5.8 (4.4,7.5)	5.9 (4.5,7.6)	4.3 (3.1,5.8)	16.6 (14.3,19.1)
				**Pairwise G^2**	0.7 (0.3,1.5)	0.5 (0.2,1.2)	1.0 (0.5,1.9)	0.1 (0.0,0.6)	0.3 (0.1,0.9)	3.6 (2.5,4.9)
				**Pairwise χ^2**	0.7 (0.3,1.5)	0.5 (0.2,1.2)	1.0 (0.5,1.9)	0.2 (0.0,0.7)	0.3 (0.1,0.9)	3.6 (2.5,4.9)
**L**	**L**	**H**	**L**	**Res. Cor.**	11.1 (9.2,13.3)	0.1 (0.0,0.6)	12.9 (10.9,15.2)	0.4 (0.1,1.0)	11.9 (10.0,14.1)	24.3 (21.6,27.1)
				**Pairwise G^2**	1.9 (1.2,3.0)	0.0 (0.0,0.4)	2.2 (1.4,3.3)	0.0 (0.0,0.4)	0.8 (0.3,1.6)	13.6 (11.6,15.9)
				**Pairwise χ^2**	1.9 (1.2,3.0)	0.0 (0.0,0.4)	2.3 (1.5,3.4)	0.0 (0.0,0.4)	0.8 (0.3,1.6)	13.0 (11.0,15.3)
**L**	**L**	**H**	**H**	**Res. Cor.**	31.1 (28.2,34.1)	1.3 (0.7,2.2)	0.7 (0.3,1.4)	0.9 (0.4,1.7)	0.8 (0.3,1.6)	26.6 (23.9,29.5)
				**Pairwise G^2**	5.7 (4.3,7.3)	0.3 (0.1,0.9)	0.1 (0.0,0.6)	0.0 (0.0,0.4)	0.0 (0.0,0.4)	24.4 (21.8,27.2)
				**Pairwise χ^2**	5.7 (4.3,7.3)	0.4 (0.1,1.0)	0.1 (0.0,0.6)	0.0 (0.0,0.4)	0.0 (0.0,0.4)	23.6 (21.0,26.4)
**L**	**H**	**L**	**H**	**Res. Cor.**	0.0 (0.0,0.4)	22.3 (19.8,25.0)	1.5 (0.8,2.5)	0.2 (0.0,0.7)	0.0 (0.0,0.4)	26.4 (23.7,29.2)
				**Pairwise G^2**	0.0 (0.0,0.4)	2.0 (1.2,3.1)	0.4 (0.1,1.0)	0.0 (0.0,0.4)	0.0 (0.0,0.4)	10.1 (8.3,12.1)
				**Pairwise χ^2**	0.0 (0.0,0.4)	2.0 (1.2,3.1)	0.4 (0.1,1.0)	0.0 (0.0,0.4)	0.0 (0.0,0.4)	9.6 (7.8,11.6)
**Sensitivity: L L L L**										
**Specificity**				**Tool**	**T_1&T_2**	**T_1&T_3**	**T_1&T_4**	**T_2&T_3**	**T_2&T_4**	**T_3&T_4**
**H**	**H**	**L**	**L**	**Res. Cor.**	0.0 (0.0,0.6)	0.2 (0.0,0.9)	0.3 (0.0,1.1)	0.5 (0.1,1.4)	0.2 (0.0,0.9)	64.9 (61.0,68.5)
				**Pairwise G^2**	0.0 (0.0,0.6)	0.0 (0.0,0.6)	0.0 (0.0,0.6)	0.0 (0.0,0.6)	0.0 (0.0,0.6)	5.1 (3.5,7.1)
				**Pairwise χ^2**	0.0 (0.0,0.6)	0.0 (0.0,0.6)	0.0 (0.0,0.6)	0.0 (0.0,0.6)	0.0 (0.0,0.6)	5.1 (3.5,7.1)
**H**	**H**	**H**	**L**	**Res. Cor.**	0.0 (0.0,0.4)	0.0 (0.0,0.4)	2.5 (1.7,3.7)	0.0 (0.0,0.4)	1.4 (0.8,2.4)	57.8 (54.7,60.9)
				**Pairwise G^2**	0.0 (0.0,0.4)	0.0 (0.0,0.4)	0.2 (0.0,0.7)	0.0 (0.0,0.4)	0.0 (0.0,0.4)	20.9 (18.4,23.6)
				**Pairwise χ^2**	0.0 (0.0,0.4)	0.0 (0.0,0.4)	0.2 (0.0,0.7)	0.0 (0.0,0.4)	0.0 (0.0,0.4)	20.9 (18.4,23.6)
**H**	**H**	**H**	**H**	**Res. Cor.**	0.0 (0.0,0.4)	0.2 (0.0,0.7)	0.1 (0.0,0.6)	0.1 (0.0,0.6)	0.0 (0.0,0.4)	96.1 (94.7,97.2)
				**Pairwise G^2**	0.2 (0.0,0.7)	0.1 (0.0,0.6)	0.0 (0.0,0.4)	0.0 (0.0,0.4)	0.0 (0.0,0.4)	88.6 (86.5,90.5)

				**Pairwise χ^2**	0.2 (0.0,0.7)	0.1 (0.0,0.6)	0.0 (0.0,0.4)	0.0 (0.0,0.4)	0.0 (0.0,0.4)	88.8 (86.7,90.7)
**L**	**L**	**L**	**L**	**Res. Cor.**	0.0 (0.0,2.3)	14.3 (9.3,20.7)	13.7 (8.8,20.0)	18.0 (12.4,24.8)	8.7 (4.8,14.2)	55.3 (47.3,63.1)
				**Pairwise G^2**	0.0 (0.0,2.3)	1.2 (0.2,4.4)	1.2 (0.2,4.4)	0.6 (0.0,3.4)	0.0 (0.0,2.3)	7.5 (3.9,12.7)
				**Pairwise χ^2**	0.0 (0.0,2.3)	1.2 (0.2,4.4)	1.2 (0.2,4.4)	0.6 (0.0,3.4)	0.0 (0.0,2.3)	7.5 (3.9,12.7)
**L**	**L**	**H**	**L**	**Res. Cor.**	0.9 (0.4,1.9)	11.2 (9.2,13.6)	29.2 (26.2,32.4)	11.2 (9.2,13.6)	29.6 (26.5,32.8)	82.7 (80.0,85.2)
				**Pairwise G^2**	0.8 (0.3,1.7)	3.1 (2.0,4.5)	4.7 (3.4,6.4)	2.0 (1.2,3.2)	3.6 (2.4,5.0)	53.6 (50.2,57.0)
				**Pairwise χ^2**	0.8 (0.3,1.7)	3.1 (2.0,4.5)	4.7 (3.4,6.4)	2.1 (1.3,3.3)	3.6 (2.4,5.0)	53.6 (50.2,57.0)
**L**	**L**	**H**	**H**	**Res. Cor.**	87.9 (85.2,90.3)	11.5 (9.1,14.2)	15.1 (12.5,18.1)	14.1 (11.5,17.0)	15.1 (12.5,18.1)	21.3 (18.2,24.6)
				**Pairwise G^2**	83.0 (79.9,85.8)	3.5 (2.2,5.2)	5.2 (3.6,7.2)	4.1 (2.7,6.0)	4.0 (2.6,5.8)	35.5 (31.8,39.3)
				**Pairwise χ^2**	83.0 (79.9,85.8)	3.5 (2.2,5.2)	5.2 (3.6,7.2)	4.1 (2.7,6.0)	4.1 (2.7,6.0)	35.5 (31.8,39.3)
**L**	**H**	**L**	**H**	**Res. Cor.**	0.0 (0.0,0.4)	42.1 (38.9,45.4)	40.5 (37.3,43.7)	0.2 (0.0,0.8)	0.0 (0.0,0.4)	71.5 (68.5,74.4)
				**Pairwise G^2**	0.0 (0.0,0.4)	5.2 (3.9,6.9)	9.3 (7.5,11.3)	0.0 (0.0,0.4)	0.0 (0.0,0.4)	33.5 (30.4,36.6)
				**Pairwise χ^2**	0.0 (0.0,0.4)	5.2 (3.9,6.9)	9.3 (7.5,11.3)	0.0 (0.0,0.4)	0.0 (0.0,0.4)	33.6 (30.5,36.7)
**Sensitivity: L L H L**										
**Specificity**				**Tool**	**T_1&T_2**	**T_1&T_3**	**T_1&T_4**	**T_2&T_3**	**T_2&T_4**	**T_3&T_4**
**H**	**H**	**L**	**L**	**Res. Cor.**	0.0 (0.0,0.4)	0.2 (0.0,0.7)	1.7 (1.0,2.8)	0.0 (0.0,0.4)	1.3 (0.7,2.3)	60.6 (57.4,63.6)
				**Pairwise G^2**	0.0 (0.0,0.4)	0.0 (0.0,0.4)	0.0 (0.0,0.4)	0.0 (0.0,0.4)	0.2 (0.0,0.7)	17.8 (15.4,20.3)
				**Pairwise χ^2**	0.0 (0.0,0.4)	0.0 (0.0,0.4)	0.0 (0.0,0.4)	0.0 (0.0,0.4)	0.2 (0.0,0.7)	17.8 (15.4,20.3)
**H**	**H**	**H**	**L**	**Res. Cor.**	0.0 (0.0,0.4)	0.0 (0.0,0.4)	4.4 (3.2,5.9)	0.0 (0.0,0.4)	7.0 (5.5,8.8)	69.1 (66.1,72.0)
				**Pairwise G^2**	0.0 (0.0,0.4)	0.0 (0.0,0.4)	0.7 (0.3,1.4)	0.0 (0.0,0.4)	0.4 (0.1,1.0)	37.0 (34.0,40.1)
				**Pairwise χ^2**	0.0 (0.0,0.4)	0.0 (0.0,0.4)	0.7 (0.3,1.4)	0.0 (0.0,0.4)	0.4 (0.1,1.0)	37.1 (34.1,40.2)
**H**	**H**	**H**	**H**	**Res. Cor.**	25.8 (23.1,28.6)	0.0 (0.0,0.4)	5.1 (3.8,6.7)	0.0 (0.0,0.4)	6.2 (4.8,7.9)	93.4 (91.7,94.9)
				**Pairwise G^2**	33.8 (30.9,36.8)	0.0 (0.0,0.4)	0.9 (0.4,1.7)	0.0 (0.0,0.4)	1.3 (0.7,2.2)	95.9 (94.5,97.0)
				**Pairwise χ^2**	33.8 (30.9,36.8)	0.0 (0.0,0.4)	1.0 (0.5,1.8)	0.0 (0.0,0.4)	1.3 (0.7,2.2)	95.9 (94.5,97.0)
**L**	**L**	**L**	**L**	**Res. Cor.**	0.0 (0.0,0.5)	14.1 (11.7,16.9)	26.0 (22.8,29.4)	15.1 (12.6,18.0)	29.4 (26.1,32.9)	74.7 (71.3,77.8)
				**Pairwise G^2**	0.1 (0.0,0.8)	1.7 (0.9,2.9)	3.7 (2.4,5.3)	3.5 (2.3,5.2)	3.8 (2.5,5.5)	38.3 (34.7,42.0)
				**Pairwise χ^2**	0.1 (0.0,0.8)	1.7 (0.9,2.9)	3.7 (2.4,5.3)	3.5 (2.3,5.2)	3.8 (2.5,5.5)	38.3 (34.7,42.0)
**L**	**L**	**H**	**L**	**Res. Cor.**	0.5 (0.2,1.2)	19.7 (17.2,22.4)	43.2 (40.0,46.4)	18.2 (15.8,20.8)	41.0 (37.8,44.2)	97.1 (95.9,98.1)
				**Pairwise G^2**	0.4 (0.1,1.1)	6.8 (5.3,8.6)	8.4 (6.7,10.3)	3.8 (2.7,5.2)	6.7 (5.2,8.4)	85.9 (83.5,88.1)
				**Pairwise χ^2**	0.4 (0.1,1.1)	6.9 (5.3,8.7)	8.4 (6.7,10.3)	3.8 (2.7,5.2)	6.7 (5.2,8.4)	85.9 (83.5,88.1)
**L**	**L**	**H**	**H**	**Res. Cor.**	99.9 (99.3,100.0)	0.8 (0.3,1.7)	16.0 (13.5,18.8)	0.4 (0.1,1.1)	16.9 (14.4,19.8)	0.5 (0.1,1.3)
				**Pairwise G^2**	97.3 (95.8,98.3)	0.3 (0.0,0.9)	2.1 (1.2,3.4)	0.1 (0.0,0.7)	3.0 (1.9,4.5)	1.4 (0.7,2.6)
				**Pairwise χ^2**	97.4 (96.0,98.4)	0.3 (0.0,0.9)	2.2 (1.3,5.5)	0.1 (0.0,0.7)	3.1 (2.0,4.6)	1.4 (0.7,2.6)
**L**	**H**	**L**	**H**	**Res. Cor.**	2.1 (1.3,3.3)	59.4 (56.2,62.6)	59.8 (56.6,63.0)	0.0 (0.0,0.4)	0.0 (0.0,0.4)	98.3 (97.3,99.0)
				**Pairwise G^2**	3.0 (2.0,4.3)	19.7 (17.2,22.3)	25.3 (22.5,28.2)	0.0 (0.0,0.4)	0.0 (0.0,0.4)	96.9 (95.6,97.9)
				**Pairwise χ^2**	2.7 (1.7,3.9)	19.9 (17.4,22.6)	25.6 (22.8,28.5)	0.0 (0.0,0.4)	0.0 (0.0,0.4)	96.9 (95.6,97.9)
**Sensitivity: L L H H**										
**Specificity**				**Tool**	**T_1&T_2**	**T_1&T_3**	**T_1&T_4**	**T_2&T_3**	**T_2&T_4**	**T_3&T_4**
**H**	**H**	**L**	**L**	**Res. Cor.**	0.0 (0.0,0.4)	0.1 (0.0,0.6)	0.3 (0.1,0.9)	0.2 (0.0,0.7)	0.1 (0.0,0.6)	96.5 (95.2,97.5)
				**Pairwise G^2**	0.0 (0.0,0.4)	0.1 (0.0,0.6)	0.0 (0.0,0.4)	0.0 (0.0,0.4)	0.0 (0.0,0.4)	72.5 (69.6,75.2)
				**Pairwise χ^2**	0.0 (0.0,0.4)	0.1 (0.0,0.6)	0.0 (0.0,0.4)	0.0 (0.0,0.4)	0.0 (0.0,0.4)	72.4 (69.5,75.1)

**H**	**H**	**H**	**L**	**Res. Cor.**	0.5 (0.2,1.2)	0.0 (0.0,0.4)	5.4 (4.1,7.0)	0.0 (0.0,0.4)	6.5 (5.1,8.2)	100.0 (99.6,100.0)
				**Pairwise G^2**	0.6 (0.2,1.3)	0.0 (0.0,0.4)	1.0 (0.5,1.8)	0.0 (0.0,0.4)	1.2 (0.6,2.1)	99.8 (99.3,100.0)
				**Pairwise χ^2**	0.6 (0.2,1.3)	0.0 (0.0,0.4)	1.0 (0.5,1.8)	0.0 (0.0,0.4)	1.5 (0.8,2.5)	99.8 (99.3,100.0)
**H**	**H**	**H**	**H**	**Res. Cor.**	99.9 (99.4,100.0)	0.0 (0.0,0.4)	0.1 (0.0,0.6)	0.0 (0.0,0.4)	0.0 (0.0,0.4)	16.5 (14.3,18.9)
				**Pairwise G^2**	100.0 (99.6,100.0)	0.0 (0.0,0.4)	0.0 (0.0,0.4)	0.0 (0.0,0.4)	0.0 (0.0,0.4)	31.0 (28.1,34.0)
				**Pairwise χ^2**	100.0 (99.6,100.0)	0.0 (0.0,0.4)	0.0 (0.0,0.4)	0.0 (0.0,0.4)	0.0 (0.0,0.4)	30.2 (27.4,33.2)
**L**	**L**	**L**	**L**	**Res. Cor.**	22.7 (19.9,25.7)	42.8 (39.4,46.3)	41.8 (38.4,45.3)	40.3 (36.9,43.7)	43.4 (40.0,46.9)	96.3 (94.7,97.4)
				**Pairwise G^2**	10.5 (8.5,12.8)	9.8 (7.9,12.0)	10.6 (8.6,12.9)	10.9 (8.8,13.2)	12.1 (9.9,14.5)	96.0 (94.4,97.2)
				**Pairwise χ^2**	10.5 (8.5,12.8)	9.9 (8.0,12.2)	10.6 (8.6,12.9)	11.0 (9.0,13.3)	12.1 (9.9,14.5)	96.0 (94.4,97.2)
**L**	**L**	**H**	**L**	**Res. Cor.**	94.5 (92.5,96.1)	9.2 (7.1,11.6)	35.4 (31.8,39.1)	9.2 (7.1,11.6)	32.9 (29.4,36.6)	22.4 (19.3,25.7)
				**Pairwise G^2**	86.2 (83.4,88.7)	4.5 (3.1,6.3)	9.5 (7.4,11.9)	6.6 (4.8,8.7)	9.0 (7.0,11.4)	35.1 (31.5,38.8)
				**Pairwise χ^2**	86.3 (83.5,88.8)	4.5 (3.1,6.3)	9.5 (7.4,11.9)	6.6 (4.8,8.7)	9.0 (7.0,11.4)	34.8 (31.2,38.5)
**L**	**L**	**H**	**H**	**Res. Cor.**	100.0 (99.6,100.0)	0.4 (0.1,1.0)	0.4 (0.1,1.0)	0.1 (0.0,0.7)	0.1 (0.0,0.7)	0.0 (0.0,0.4)
				**Pairwise G^2**	99.1 (98.2,99.6)	0.0 (0.0,0.4)	0.0 (0.0,0.4)	0.0 (0.0,0.4)	0.0 (0.0,0.4)	0.0 (0.0,0.4)
				**Pairwise χ^2**	99.1 (98.2,99.6)	0.0 (0.0,0.4)	0.0 (0.0,0.4)	0.0 (0.0,0.4)	0.0 (0.0,0.4)	0.0 (0.0,0.4)
**L**	**H**	**L**	**H**	**Res. Cor.**	30.7 (27.8,33.7)	61.3 (58.1,64.4)	77.8 (75.1,80.5)	1.2 (0.6,2.1)	0.0 (0.0,0.4)	96.1 (94.7,97.2)
				**Pairwise G^2**	30.2 (27.3,33.2)	18.1 (15.7,20.7)	53.8 (50.6,57.0)	0.0 (0.0,0.4)	0.0 (0.0,0.4)	98.7 (97.8,99.3)
				**Pairwise χ^2**	29.9 (27.0,32.9)	18.2 (15.8,20.9)	54.4 (51.2,57.6)	0.0 (0.0,0.4)	0.0 (0.0,0.4)	98.6 (97.7,99.3)

Table xi.4 Mean absolute biases (x10^{-2}) of posterior medians of each parameter obtained from the conditional independence model

**Sensitivity: H H L L**												
**Specificity**				π	Se_1	Se_2	Se_3	Se_4	Sp_1	Sp_2	Sp_3	Sp_4
**H**	**H**	**L**	**L**	0.1 (0.0,0.2)	3.0 (2.7,3.2)	2.9 (2.7,3.2)	-0.4 (-0.6,-0.2)	-0.3 (-0.5,-0.1)	0.7 (0.6,0.7)	0.6 (0.5,0.7)	-0.1 (-0.2,0.0)	-0.1 (-0.2,0.0)
**H**	**H**	**H**	**L**	-1.1 (-1.1,-1.0)	5.0 (4.9,5.1)	5.1 (4.9,5.2)	-0.1 (-0.3,0.1)	-0.1 (-0.3,0.1)	0.0 (0.0,0.1)	0.1 (0.0,0.1)	-0.8 (-0.9,-0.8)	-0.4 (-0.4,-0.3)
**H**	**H**	**H**	**H**	-1.1 (-1.1,-1.0)	5.0 (4.9,5.1)	5.0 (4.8,5.1)	0.0 (-0.2,0.1)	-0.1 (-0.2,0.1)	0.1 (0.0,0.1)	0.0 (0.0,0.1)	-0.8 (-0.8,-0.7)	-0.7 (-0.8,-0.7)
**L**	**L**	**L**	**L**	3.0 (2.6,3.3)	0.3 (-0.1,0.7)	0.7 (0.2,1.1)	-1.1 (-1.4,-0.7)	-1.2 (-1.6,-0.9)	2.0 (1.8,2.2)	1.9 (1.7,2.1)	0.3 (0.1,0.4)	0.4 (0.2,0.5)
**L**	**L**	**H**	**L**	-0.9 (-1.1,-0.8)	5.2 (5.0,5.3)	5.3 (5.1,5.4)	-1.0 (-1.4,-0.7)	-0.1 (-0.3,0.1)	0.7 (0.6,0.8)	0.5 (0.4,0.6)	-1.0 (-1.0,-0.9)	-0.3 (-0.4,-0.2)
**L**	**L**	**H**	**H**	-1.4 (-1.5,-1.3)	5.4 (5.3,5.6)	5.3 (5.2,5.4)	-0.2 (-0.5,0.0)	0.1 (-0.2,0.3)	0.4 (0.3,0.5)	0.3 (0.2,0.4)	-0.9 (-1.0,-0.9)	-0.9 (-1.0,-0.9)
**L**	**H**	**L**	**H**	-1.1 (-1.1,-1.0)	5.0 (4.9,5.1)	5.1 (5.0,5.3)	-0.1 (-0.3,0.0)	-0.2 (-0.4,0.0)	0.5 (0.4,0.5)	0.1 (0.0,0.2)	-0.3 (-0.4,-0.2)	-0.8 (-0.8,-0.7)
**Sensitivity: H H H L**												
**Specificity**				π	Se_1	Se_2	Se_3	Se_4	Sp_1	Sp_2	Sp_3	Sp_4
**H**	**H**	**L**	**L**	-0.9 (-1.0,-0.8)	4.6 (4.5,4.8)	4.8 (4.7,5.0)	-0.4 (-0.5,-0.3)	-0.2 (-0.4,-0.1)	0.1 (0.1,0.2)	0.1 (0.1,0.2)	-0.7 (-0.7,-0.6)	-0.3 (-0.4,-0.2)
**H**	**H**	**H**	**L**	-1.2 (-1.3,-1.2)	5.3 (5.2,5.4)	5.4 (5.3,5.5)	-0.1 (-0.2,0.0)	-0.1 (-0.3,0.1)	0.0 (-0.1,0.1)	0.0 (-0.1,0.1)	-1.3 (-1.3,-1.2)	-0.3 (-0.4,-0.2)
**H**	**H**	**H**	**H**	-1.1 (-1.1,-1.0)	5.0 (4.9,5.1)	5.0 (4.9,5.0)	-0.1 (-0.2,0.0)	-0.1 (-0.2,0.1)	0.1 (0.0,0.1)	0.0 (-0.1,0.0)	-1.1 (-1.2,-1.1)	-0.7 (-0.8,-0.7)
**L**	**L**	**L**	**L**	0.1 (0.0,0.3)	3.9 (3.7,4.1)	3.9 (3.7,4.0)	-2.2 (-2.5,-1.9)	-0.4 (-0.6,-0.2)	0.9 (0.8,1.0)	1.0 (0.9,1.1)	-0.5 (-0.6,-0.4)	-0.1 (-0.2,0.0)
**L**	**L**	**H**	**L**	-1.5 (-1.6,-1.4)	6.4 (6.2,6.5)	6.4 (6.3,6.5)	-1.7 (-2.0,-1.4)	0.2 (0.0,0.4)	0.5 (0.4,0.5)	0.5 (0.4,0.6)	-2.0 (-2.1,-1.9)	-0.4 (-0.5,-0.3)
**L**	**L**	**H**	**H**	-1.5 (-1.6,-1.4)	5.3 (5.1,5.4)	5.2 (5.1,5.4)	0.2 (0.0,0.4)	0.6 (0.4,0.8)	0.2 (0.1,0.3)	0.2 (0.1,0.3)	-1.5 (-1.6,-1.4)	-0.9 (-0.9,-0.8)
**L**	**H**	**L**	**H**	-1.1 (-1.2,-1.1)	4.9 (4.8,5.0)	5.3 (5.2,5.5)	-0.3 (-0.5,-0.2)	-0.1 (-0.3,0.1)	0.5 (0.4,0.6)	0.0 (0.0,0.1)	-0.8 (-0.9,-0.7)	-0.8 (-0.9,-0.7)

Sensitivity: H H H H												
**Specificity**				π	Se_1	Se_2	Se_3	Se_4	Sp_1	Sp_2	Sp_3	Sp_4
**H**	**H**	**L**	**L**	-1.1 (-1.2,-1.0)	5.1 (4.9,5.2)	5.1 (4.9,5.2)	-0.2 (-0.3,-0.1)	-0.2 (-0.3,-0.1)	0.1 (0.0,0.1)	0.1 (0.0,0.1)	-0.7 (-0.8,-0.6)	-0.7 (-0.8,-0.7)
**H**	**H**	**H**	**L**	-1.2 (-1.2,-1.1)	5.1 (5.0,5.2)	5.1 (5.0,5.2)	-0.2 (-0.4,-0.1)	-0.2 (-0.3,-0.1)	0.0 (0.0,0.1)	0.0 (0.0,0.1)	-1.2 (-1.3,-1.2)	-0.8 (-0.8,-0.7)
**H**	**H**	**H**	**H**	-1.0 (-1.1,-1.0)	4.7 (4.6,4.8)	4.8 (4.7,4.9)	-0.1 (-0.2,0.1)	-0.1 (-0.2,0.0)	0.0 (0.0,0.1)	0.0 (0.0,0.1)	-1.1 (-1.1,-1.1)	-1.1 (-1.1,-1.0)
**L**	**L**	**L**	**L**	-1.0 (-1.1,-0.9)	5.0 (4.9,5.2)	4.9 (4.7,5.0)	-0.5 (-0.7,-0.3)	-0.5 (-0.7,-0.3)	0.5 (0.4,0.6)	0.5 (0.4,0.6)	-0.8 (-0.9,-0.7)	-0.8 (-0.9,-0.7)
**L**	**L**	**H**	**L**	-1.7 (-1.7,-1.6)	5.6 (5.5,5.7)	5.6 (5.5,5.8)	0.1 (-0.1,0.3)	0.6 (0.5,0.8)	0.3 (0.2,0.4)	0.2 (0.1,0.3)	-1.7 (-1.7,-1.6)	-1.0 (-1.0,-0.9)
**L**	**L**	**H**	**H**	-1.4 (-1.4,-1.3)	4.5 (4.4,4.6)	4.5 (4.4,4.6)	0.7 (0.5,0.8)	0.7 (0.6,0.9)	0.1 (0.0,0.2)	0.2 (0.1,0.3)	-1.3 (-1.3,-1.2)	-1.2 (-1.3,-1.2)
**L**	**H**	**L**	**H**	-1.3 (-1.4,-1.2)	4.9 (4.9,5.0)	5.9 (5.8,6.0)	-0.1 (-0.2,0.1)	-0.1 (-0.2,0.1)	0.3 (0.2,0.4)	0.0 (-0.1,0.0)	-0.8 (-0.9,-0.8)	-1.4 (-1.4,-1.3)
**Sensitivity: L L L L**												
**Specificity**				π	Se_1	Se_2	Se_3	Se_4	Sp_1	Sp_2	Sp_3	Sp_4
**H**	**H**	**L**	**L**	-5.9 (-6.0,-5.7)	23.0 (22.4,23.5)	23.2 (22.7,23.7)	-0.1 (-0.4,0.2)	-0.2 (-0.4,0.1)	0.2 (0.1,0.3)	0.3 (0.2,0.4)	-1.4 (-1.5,-1.4)	-1.4 (-1.5,-1.3)
**H**	**H**	**H**	**L**	-6.2 (-6.3,-6.1)	23.0 (22.7,23.3)	23.4 (23.1,23.7)	-0.2 (-0.4,0.1)	0.3 (0.1,0.5)	0.1 (0.0,0.1)	0.1 (0.0,0.1)	-3.8 (-3.8,-3.7)	-1.3 (-1.4,-1.3)
**H**	**H**	**H**	**H**	-5.4 (-5.5,-5.3)	20.0 (19.7,20.3)	20.1 (19.8,20.4)	0.7 (0.4,0.9)	0.7 (0.5,0.9)	0.2 (0.2,0.3)	0.2 (0.2,0.3)	-3.1 (-3.1,-3.0)	-3.1 (-3.2,-3.1)
**L**	**L**	**L**	**L**	-4.9 (-5.7,-4.2)	24.8 (23.9,25.8)	24.8 (23.8,25.9)	-2.1 (-3.2,-1.0)	-1.6 (-2.6,-0.5)	3.1 (2.7,3.5)	2.9 (2.5,3.2)	-1.6 (-1.8,-1.3)	-1.6 (-1.8,-1.4)
**L**	**L**	**H**	**L**	-9.4 (-9.5,-9.2)	31.8 (31.6,32.1)	32.0 (31.7,32.3)	-4.1 (-4.8,-3.5)	1.4 (1.0,1.8)	1.7 (1.6,1.8)	1.7 (1.6,1.8)	-5.9 (-6.0,-5.8)	-2.0 (-2.0,-1.9)
**L**	**L**	**H**	**H**	-5.9 (-6.2,-5.6)	13.9 (13.2,14.7)	13.8 (13.0,14.5)	5.8 (5.2,6.4)	5.4 (4.9,5.9)	0.5 (0.4,0.6)	0.5 (0.4,0.6)	-2.6 (-2.8,-2.5)	-2.6 (-2.8,-2.5)
**L**	**H**	**L**	**H**	-7.6 (-7.7,-7.5)	22.7 (22.4,23.0)	32.4 (32.1,32.6)	0.5 (0.2,0.7)	-0.6 (-0.9,-0.2)	1.5 (1.4,1.5)	0.1 (0.0,0.2)	-1.7 (-1.8,-1.6)	-4.5 (-4.5,-4.4)
**Sensitivity: L L H L**												
**Specificity**				π	Se_1	Se_2	Se_3	Se_4	Sp_1	Sp_2	Sp_3	Sp_4
**H**	**H**	**L**	**L**	-6.4 (-6.5,-6.3)	23.7 (23.4,24.0)	23.5 (23.2,23.8)	-0.4 (-0.6,-0.3)	0.2 (0.0,0.4)	0.0 (-0.1,0.1)	0.0 (-0.1,0.0)	-3.7 (-3.8,-3.6)	-1.5 (-1.6,-1.5)
**H**	**H**	**H**	**L**	-6.3 (-6.4,-6.2)	23.4 (23.2,23.7)	22.9 (22.7,23.1)	-0.2 (-0.4,0.0)	1.0 (0.8,1.2)	0.0 (0.0,0.1)	0.0 (-0.1,0.1)	-5.9 (-6.0,-5.9)	-1.4 (-1.4,-1.3)
**H**	**H**	**H**	**H**	-4.2 (-4.3,-4.2)	15.3 (15.0,15.6)	15.3 (15.0,15.6)	-0.1 (-0.3,0.0)	2.3 (2.1,2.5)	0.2 (0.2,0.3)	0.2 (0.2,0.3)	-4.0 (-4.1,-4.0)	-2.1 (-2.2,-2.0)
**L**	**L**	**L**	**L**	-8.1 (-8.2,-7.9)	29.9 (29.6,30.3)	29.8 (29.4,30.1)	-7.3 (-7.8,-6.9)	0.0 (-0.4,0.4)	2.2 (2.1,2.3)	2.2 (2.1,2.4)	-5.7 (-5.8,-5.6)	-1.9 (-2.0,-1.8)
**L**	**L**	**H**	**L**	-9.9 (-10.0,-9.8)	33.8 (33.6,34.1)	34.0 (33.8,34.2)	-8.1 (-8.6,-7.6)	3.1 (2.8,3.4)	1.6 (1.5,1.7)	1.5 (1.5,1.6)	-9.9 (-10.0,-9.8)	-1.8 (-1.9,-1.7)
**L**	**L**	**H**	**H**	-1.6 (-1.8,-1.5)	4.7 (4.4,5.0)	4.8 (4.5,5.1)	-0.9 (-1.3,-0.5)	4.1 (3.7,4.5)	0.5 (0.4,0.6)	0.5 (0.4,0.6)	-2.0 (-2.1,-1.8)	-0.2 (-0.3,-0.1)
**L**	**H**	**L**	**H**	-6.4 (-6.6,-6.3)	20.4 (20.1,20.7)	25.6 (25.1,26.0)	0.7 (0.5,0.9)	0.3 (0.0,0.6)	1.6 (1.5,1.7)	0.0 (0.0,0.1)	-3.5 (-3.6,-3.4)	-3.7 (-3.7,-3.6)
**Sensitivity: L L H H**												
**Specificity**				π	Se_1	Se_2	Se_3	Se_4	Sp_1	Sp_2	Sp_3	Sp_4
**H**	**H**	**L**	**L**	-5.8 (-5.9,-5.7)	22.0 (21.7,22.3)	21.9 (21.7,22.2)	-0.1 (-0.2,0.1)	-0.1 (-0.3,0.0)	0.2 (0.1,0.2)	0.2 (0.1,0.3)	-3.4 (-3.5,-3.3)	-3.5 (-3.6,-3.4)
**H**	**H**	**H**	**L**	-5.2 (-5.3,-5.2)	19.4 (19.1,19.6)	19.4 (19.1,19.6)	-0.2 (-0.3,0.0)	0.8 (0.6,0.9)	0.2 (0.1,0.2)	0.3 (0.2,0.3)	-4.9 (-5.0,-4.9)	-2.9 (-3.0,-2.8)
**H**	**H**	**H**	**H**	-1.2 (-1.3,-1.1)	4.7 (4.5,4.9)	4.9 (4.6,5.1)	0.1 (-0.1,0.2)	0.0 (-0.1,0.1)	0.3 (0.2,0.3)	0.3 (0.2,0.3)	-1.2 (-1.3,-1.1)	-1.2 (-1.3,-1.1)
**L**	**L**	**L**	**L**	-8.3 (-8.4,-8.1)	27.1 (26.6,27.6)	27.3 (26.9,27.8)	0.1 (-0.2,0.4)	0.0 (-0.3,0.3)	1.6 (1.5,1.7)	1.6 (1.5,1.7)	-4.8 (-4.9,-4.7)	-4.7 (-4.8,-4.6)
**L**	**L**	**H**	**L**	-2.8 (-3.1,-2.6)	9.1 (8.5,9.8)	9.2 (8.5,9.8)	-1.8 (-2.2,-1.4)	3.4 (3.2,3.7)	0.8 (0.7,0.9)	0.8 (0.7,0.9)	-3.3 (-3.5,-3.1)	-1.0 (-1.2,-0.8)
**L**	**L**	**H**	**H**	0.4 (0.2,0.5)	0.9 (0.7,1.1)	0.9 (0.7,1.1)	-0.8 (-1.1,-0.5)	-0.6 (-0.9,-0.3)	0.2 (0.1,0.3)	0.3 (0.2,0.4)	0.1 (0.1,0.2)	0.0 (-0.1,0.1)
**L**	**H**	**L**	**H**	-5.4 (-5.5,-5.3)	17.0 (16.7,17.3)	19.9 (19.5,20.4)	1.6 (1.5,1.8)	0.1 (-0.1,0.3)	1.5 (1.4,1.6)	0.1 (0.0,0.1)	-2.9 (-3.0,-2.8)	-5.1 (-5.2,-5.0)

Table xi.5 Coverages of the 95% CrIs for each model parameter

**Sensitivity: H H L L**												
**Specificity**				π	Se_1	Se_2	Se_3	Se_4	Sp_1	Sp_2	Sp_3	Sp_4
**H**	**H**	**L**	**L**	98.5 (97.7,99.3)	96.4 (95.1,97.6)	96.2 (95.0,97.5)	94.3 (92.8,95.8)	95.0 (93.5,96.4)	94.2 (92.6,95.7)	96.4 (95.0,97.6)	94.1 (92.5,95.6)	95.2 (93.8,96.6)
**H**	**H**	**H**	**L**	84.3 (82.0,86.6)	52.2 (49.1,55.3)	50.6 (47.5,53.7)	94.6 (93.2,96.0)	95.5 (94.2,96.8)	95.0 (93.6,96.4)	94.8 (93.4,96.2)	82.5 (80.1,84.9)	93.8 (92.3,95.3)
**H**	**H**	**H**	**H**	82.8 (80.4,85.1)	35.8 (32.9,38.8)	36.7 (33.7,39.7)	95.3 (94.0,96.6)	95.3 (94.0,96.6)	94.7 (93.3,96.1)	95.3 (94.0,96.6)	84.1 (81.8,86.4)	85.3 (83.1,87.5)
**L**	**L**	**L**	**L**	96.1 (94.7,97.8)	99.4 (98.7,100.0)	99.6 (99.0,100.0)	95.1 (93.1,97.0)	96.9 (95.4,98.4)	91.8 (89.3,94.2)	92.2 (89.8,94.6)	95.9 (94.1,97.6)	95.7 (93.9,97.5)
**L**	**L**	**H**	**L**	95.8 (94.5,97.1)	68.9 (66.0,71.9)	69.6 (66.7,72.5)	94.7 (93.3,96.2)	95.2 (93.8,96.5)	94.1 (92.6,95.6)	94.5 (93.1,96.0)	88.4 (86.4,90.5)	93.9 (92.4,95.4)
**L**	**L**	**H**	**H**	87.6 (85.5,89.6)	46.3 (43.2,49.4)	44.5 (41.4,47.6)	93.4 (91.8,94.9)	94.4 (93.0,95.8)	95.0 (93.6,96.3)	93.8 (92.3,95.3)	83.7 (81.4,85.9)	82.7 (80.4,85.1)
**L**	**H**	**L**	**H**	92.2 (90.6,93.9)	45.7 (42.6,48.8)	78.5 (76.0,81.1)	94.9 (93.5,96.2)	94.2 (92.7,95.6)	94.5 (93.0,95.9)	95.4 (94.1,96.7)	93.8 (92.3,95.3)	88.2 (86.2,90.2)
**Sensitivity: H H H L**												
**Specificity**				π	Se_1	Se_2	Se_3	Se_4	Sp_1	Sp_2	Sp_3	Sp_4
**H**	**H**	**L**	**L**	92.0 (90.3,93.7)	65.7 (62.8,68.7)	64.6 (61.7,67.6)	93.1 (91.5,94.7)	95.5 (94.2,96.8)	96.0 (94.8,97.2)	96.0 (94.8,97.2)	92.7 (91.1,94.3)	95.1 (93.7,96.4)
**H**	**H**	**H**	**L**	77.2 (74.6,79.8)	20.5 (18.0,23.0)	20.5 (18.0,23.0)	94.4 (93.0,95.8)	95.5 (94.2,96.8)	94.9 (93.5,96.3)	95.9 (94.7,97.1)	67.7 (64.8,70.6)	94.4 (93.0,95.8)
**H**	**H**	**H**	**H**	80.1 (77.6,82.6)	18.0 (15.6,20.4)	18.8 (16.4,21.2)	95.2 (93.9,96.5)	94.9 (93.5,96.3)	94.5 (93.1,95.9)	96.0 (94.8,97.2)	72.7 (69.9,75.5)	83.4 (81.1,85.7)
**L**	**L**	**L**	**L**	98.2 (97.4,99.1)	90.6 (88.7,92.5)	92.6 (90.9,94.3)	92.9 (91.2,94.6)	95.8 (94.5,97.1)	93.6 (92.0,95.2)	92.8 (91.1,94.5)	95.8 (94.5,97.1)	96.8 (95.6,97.9)
**L**	**L**	**H**	**L**	89.4 (87.5,91.3)	27.0 (24.2,29.8)	25.1 (22.4,27.9)	96.4 (95.2,97.6)	93.7 (92.2,95.2)	95.9 (94.6,97.1)	95.1 (93.7,96.4)	56.7 (53.6,59.9)	93.9 (92.4,95.4)
**L**	**L**	**H**	**H**	80.3 (77.8,82.7)	28.8 (26.0,31.6)	29.7 (26.9,32.6)	96.4 (95.2,97.6)	95.3 (94.0,96.6)	94.6 (93.2,96.0)	94.6 (93.2,96.0)	67.0 (64.1,69.9)	83.5 (81.2,85.8)
**L**	**H**	**L**	**H**	85.8 (83.6,88.0)	34.7 (31.7,37.7)	59.8 (56.8,62.8)	95.9 (94.7,97.1)	94.6 (93.2,96.0)	94.5 (93.1,95.9)	95.7 (94.4,97.0)	91.6 (89.9,93.3)	83.5 (81.2,85.8)
**Sensitivity: H H H H**												
**Specificity**				π	Se_1	Se_2	Se_3	Se_4	Sp_1	Sp_2	Sp_3	Sp_4
**H**	**H**	**L**	**L**	85.1 (82.9,87.3)	40.5 (37.5,43.5)	42.5 (39.4,45.6)	94.6 (93.2,96.0)	93.6 (92.1,95.1)	94.0 (92.5,95.5)	94.6 (93.2,96.0)	91.9 (90.2,93.6)	91.5 (89.8,93.2)
**H**	**H**	**H**	**L**	76.4 (73.8,79.0)	16.4 (14.1,18.7)	18.6 (16.2,21.0)	95.7 (94.4,97.0)	94.9 (93.5,96.3)	95.9 (94.7,97.1)	95.2 (93.9,96.5)	66.2 (63.3,69.1)	90.7 (88.9,92.5)
**H**	**H**	**H**	**H**	80.2 (77.7,82.7)	15.9 (13.6,18.2)	16.9 (14.6,19.2)	94.9 (93.5,96.3)	95.4 (94.1,96.7)	94.2 (92.8,95.6)	95.3 (94.0,96.6)	72.5 (69.7,75.3)	71.7 (68.9,74.5)
**L**	**L**	**L**	**L**	94.6 (93.2,96.0)	66.6 (63.7,69.6)	68.2 (65.2,71.1)	96.1 (94.9,97.3)	94.5 (93.0,95.9)	94.4 (92.9,95.8)	94.5 (93.0,95.9)	92.8 (91.2,94.5)	91.5 (89.8,93.3)
**L**	**L**	**H**	**L**	79.8 (77.3,82.3)	25.0 (22.3,27.7)	23.8 (21.1,26.4)	96.7 (95.6,97.8)	93.6 (92.1,95.1)	94.9 (93.5,96.3)	95.2 (93.9,96.5)	61.4 (58.4,64.4)	90.7 (88.9,92.5)
**L**	**L**	**H**	**H**	77.3 (74.7,79.9)	33.1 (30.2,36.0)	32.0 (29.1,34.9)	95.5 (94.2,96.8)	93.8 (92.3,95.3)	94.8 (93.4,96.2)	95.2 (93.9,96.5)	73.2 (70.5,75.9)	73.8 (71.1,76.5)
**L**	**H**	**L**	**H**	79.7 (77.2,82.2)	14.2 (12.0,16.4)	28.3 (25.5,31.1)	94.1 (92.6,95.6)	95.0 (93.6,96.4)	93.8 (92.3,95.3)	95.3 (94.0,96.6)	89.6 (87.7,91.5)	66.4 (63.5,69.3)
**Sensitivity: L L L L**												
**Specificity**				π	Se_1	Se_2	Se_3	Se_4	Sp_1	Sp_2	Sp_3	Sp_4
**H**	**H**	**L**	**L**	50.8 (46.9,54.6)	21.1 (17.9,24.2)	16.9 (14.0,19.8)	94.6 (92.8,96.3)	94.9 (93.2,96.6)	97.7 (96.5,98.8)	96.9 (95.6,98.2)	79.3 (76.1,82.4)	81.3 (78.3,84.3)
**H**	**H**	**H**	**L**	6.0 (4.5,7.5)	0.3 (0.0,0.6)	0.1 (0.0,0.3)	94.3 (92.9,95.8)	95.4 (94.1,96.7)	95.7 (94.5,97.0)	94.6 (93.2,96.0)	2.7 (1.7,3.8)	77.2 (74.6,79.9)
**H**	**H**	**H**	**H**	6.9 (5.4,8.5)	0.3 (0.0,0.6)	0.7 (0.2,1.2)	95.9 (94.6,97.1)	94.4 (92.9,95.8)	94.4 (92.9,95.8)	94.4 (92.9,95.8)	10.2 (8.3,12.1)	10.8 (8.8,12.7)
**L**	**L**	**L**	**L**	93.8 (90.1,97.5)	35.4 (28.0,42.8)	33.5 (26.2,40.8)	91.3 (87.0,95.7)	95.7 (92.5,98.8)	73.9 (67.1,80.7)	75.2 (68.5,81.8)	82.6 (76.8,88.5)	85.1 (79.6,90.6)
**L**	**L**	**H**	**L**	23.0 (20.1,25.8)	0.7 (0.1,1.3)	0.5 (0.0,0.9)	89.8 (87.8,91.9)	93.5 (91.8,95.2)	82.2 (79.7,84.8)	82.1 (79.5,84.7)	0.6 (0.1,1.1)	68.9 (65.8,72.0)
**L**	**L**	**H**	**H**	59.3 (55.6,63.1)	45.0 (41.1,48.8)	48.2 (44.3,52.0)	90.2 (87.9,92.5)	91.4 (89.3,93.6)	94.5 (92.7,96.2)	95.1 (93.5,96.8)	60.1 (56.3,63.8)	61.0 (57.3,64.7)
**L**	**H**	**L**	**H**	9.8 (7.9,11.7)	0.2 (0.0,0.5)	0.3 (0.0,0.7)	95.2 (93.8,96.6)	95.4 (94.1,96.8)	81.2 (78.7,83.8)	96.0 (94.7,97.2)	73.7 (70.9,76.6)	1.0 (0.3,1.6)
**Sensitivity: L L H L**												
**Specificity**				π	Se_1	Se_2	Se_3	Se_4	Sp_1	Sp_2	Sp_3	Sp_4

**H**	**H**	**L**	**L**	6.0 (4.5,7.5)	0.4 (0.0,0.8)	0.3 (0.0,0.7)	95.6 (94.3,96.9)	94.6 (93.2,96.0)	96.2 (95.0,97.4)	94.7 (93.3,96.1)	23.4 (20.7,26.0)	76.5 (73.9,79.2)
**H**	**H**	**H**	**L**	0.1 (0.0,0.3)	0.0 (0.0,0.0)	0.0 (0.0,0.0)	95.0 (93.6,96.4)	93.7 (82.2,95.2)	94.4 (93.0,95.8)	95.1 (93.8,96.4)	0.0 (0.0,0.0)	79.0 (76.5,81.5)
**H**	**H**	**H**	**H**	15.7 (13.4,18.0)	5.5 (4.1,6.9)	7.0 (5.4,8.6)	95.6 (94.3,96.9)	89.6 (87.7,91.5)	93.7 (92.2,95.2)	94.3 (92.9,95.7)	7.1 (5.5,8.7)	37.0 (34.0,40.0)
**L**	**L**	**L**	**L**	53.3 (49.6,57.0)	2.4 (1.3,3.5)	2.4 (1.3,3.5)	90.4 (88.2,92.6)	96.3 (94.9,97.7)	76.7 (73.5,79.8)	73.8 (70.6,77.1)	7.5 (5.6,9.4)	72.6 (69.3,75.8)
**L**	**L**	**H**	**L**	1.6 (0.8,2.4)	0.3 (0.0,0.7)	0.1 (0.0,0.3)	90.9 (89.1,92.7)	91.0 (89.2,92.8)	81.3 (78.8,83.8)	82.4 (80.0,84.9)	0.1 (0.0,0.3)	71.3 (68.4,74.2)
**L**	**L**	**H**	**H**	91.7 (89.7,93.6)	76.3 (73.3,79.3)	75.5 (72.4,78.5)	97.8 (96.798.8)	88.4 (86.1,90.7)	94.3 (92.6,95.9)	92.0 (90.1,94.0)	80.2 (77.4,83.0)	95.0 (93.5,96.6)
**L**	**H**	**L**	**H**	17.4 (15.0,19.9)	1.0 (0.3,1.6)	5.4 (4.0,6.9)	94.3 (92.8,95.7)	93.5 (91.9,95.1)	77.0 (74.4,79.7)	94.7 (93.3,96.1)	31.6 (28.6,34.5)	10.8 (8.9,12.8)
**Sensitivity: L L H H**												
**Specificity**				π	Se_1	Se_2	Se_3	Se_4	Sp_1	Sp_2	Sp_3	Sp_4
**H**	**H**	**L**	**L**	2.5 (1.5,3.5)	0.0 (0.0,0.0)	0.0 (0.0,0.0)	96.1 (94.9,97.3)	94.6 (93.2,96.0)	94.4 (93.0,95.8)	93.6 (92.1,95.1)	25.7 (23.0,28.4)	23.5 (209,26.2)
**H**	**H**	**H**	**L**	1.5 (0.7,2.3)	0.4 (0.0,0.8)	0.1 (0.0,0.3)	95.6 (94.3,96.9)	93.7 (92.2,95.2)	95.6 (94.3,96.9)	94.3 (92.8,95.7)	0.4 (0.0,0.8)	37.8 (34.8,40.8)
**H**	**H**	**H**	**H**	82.9 (80.6,85.2)	68.0 (65.1,70.9)	65.3 (62.3,68.3)	96.7 (95.6,97.8)	94.4 (93.0,95.8)	94.1 (92.6,95.6)	94.8 (93.4,96.2)	75.9 (73.2,78.6)	74.1 (71.4,76.8)
**L**	**L**	**L**	**L**	25.3 (22.3,28.2)	5.6 (4.0,7.1)	6.2 (4.5,7.8)	98.7 (97.9,99.5)	98.8 (98.0,99.5)	83.4 (80.9,86.0)	81.5 (78.9,84.1)	24.2 (21.3,27.1)	23.8 (209,26.7)
**L**	**L**	**H**	**L**	80.6 (77.7,83.6)	58.5 (54.8,62.2)	56.8 (53.1,60.5)	99.1 (98.4,99.8)	88.2 (85.8,90.6)	92.0 (90.0,94.0)	92.0 (90.0,94.0)	59.8 (56.2,63.5)	86.9 (84.4,89.4)
**L**	**L**	**H**	**H**	95.1 (93.6,96.5)	92.9 (91.2,94.7)	93.4 (91.7,95.1)	97.1 (95.9,98.2)	97.5 (96.5,98.6)	94.2 (92.7,95.8)	94.3 (92.8,95.9)	96.1 (94.8,97.4)	96.5 (95.2,97.7)
**L**	**H**	**L**	**H**	20.8 (18.2,23.4)	7.0 (5.3,8.6)	12.7 (10.5,14.8)	89.7 (87.7,91.6)	97.0 (96.0,98.1)	76.8 (74.1,79.5)	95.9 (94.6,97.2)	47.6 (44.4,50.8)	11.4 (9.4,13.4)

xii. Setting 12: $\pi = 0.2, \omega = 0.5, n_{obs} = 500$

Table xii.1 Number of converged data sets for different sensitivity-specificity combinations

				**Number of converged data sets**
**Specificity**				**Sensitivity: H H L L**
**H**	**H**	**L**	**L**	931
**H**	**H**	**H**	**L**	998
**H**	**H**	**H**	**H**	1000
**L**	**L**	**L**	**L**	475
**L**	**L**	**H**	**L**	923
**L**	**L**	**H**	**H**	988
**L**	**H**	**L**	**H**	986
**Specificity**				**Sensitivity: H H H L**
**H**	**H**	**L**	**L**	999
**H**	**H**	**H**	**L**	1000
**H**	**H**	**H**	**H**	1000
**L**	**L**	**L**	**L**	857
**L**	**L**	**H**	**L**	989
**L**	**L**	**H**	**H**	999
**L**	**H**	**L**	**H**	998

Specificity				Sensitivity: H H H H
H	H	L	L	1000
H	H	H	L	999
H	H	H	H	1000
L	L	L	L	976
L	L	H	L	999
L	L	H	H	1000
L	H	L	H	1000
**Specificity**				**Sensitivity: L L L L**
H	H	L	L	784
H	H	H	L	968
H	H	H	H	990
L	L	L	L	248
L	L	H	L	532
L	L	H	H	730
L	H	L	H	853
**Specificity**				**Sensitivity: L L H L**
H	H	L	L	970
H	H	H	L	1000
H	H	H	H	995
L	L	L	L	379
L	L	H	L	738
L	L	H	H	782
L	H	L	H	928
**Specificity**				**Sensitivity: L L H H**
H	H	L	L	996
H	H	H	L	998
H	H	H	H	997
L	L	L	L	581
L	L	H	L	727
L	L	H	H	909
L	H	L	H	944

Table xii.2 Percentages of the time 95% credible intervals (CrIs) for residual correlations did not included '0' for any pairs, and percentages of the time that the overall χ^2 or G^2 statistic indicated a lack of overall fit

Sensitivity: H H L L						
**Specificity**				**Res. Cor.**	**Overall G^2**	**Overall χ^2**
H	H	L	L	60.8 (57.6,63.9)	10.3 (8.4,12.4)	8.2 (6.5,10.1)
H	H	H	L	23.8 (21.2,26.6)	4.5 (3.3,6.0)	5.2 (3.9,6.8)

**H**	**H**	**H**	**H**	9.8 (8.0,11.8)	4.9 (3.6,6.4)	7.5 (5.9,9.3)
**L**	**L**	**L**	**L**	54.5 (49.9,59.1)	12.4 (9.6,15.7)	12.0 (9.2,15.3)
**L**	**L**	**H**	**L**	32.3 (29.3,35.4)	11.6 (9.6,13.8)	10.0 (8.1,12.1)
**L**	**L**	**H**	**H**	5.1 (3.8,6.6)	11.0 (9.1,13.2)	19.3 (16.9,21.9)
**L**	**H**	**L**	**H**	36.1 (33.1,39.2)	6.8 (5.3,8.5)	8.6 (6.9,10.5)
**Sensitivity: H H H L**						
**Specificity**				**Res. Cor.**	**Overall G^2**	**Overall χ^2**
**H**	**H**	**L**	**L**	33.6 (30.7,36.7)	8.3 (6.7,10.2)	7.7 (6.1,9.5)
**H**	**H**	**H**	**L**	8.0 (6.4,9.9)	4.3 (3.1,5.7)	4.6 (3.4,6.1)
**H**	**H**	**H**	**H**	3.6 (2.5,4.9)	8.1 (6.5,10.0)	11.5 (9.6,13.6)
**L**	**L**	**L**	**L**	37.5 (34.2,40.8)	13.9 (11.6,16.4)	13.9 (11.6,16.4)
**L**	**L**	**H**	**L**	35.7 (32.7,38.8)	13.1 (11.1,15.4)	11.4 (9.5,13.6)
**L**	**L**	**H**	**H**	14.2 (12.1,16.5)	19.5 (17.1,22.1)	28.9 (26.1,31.8)
**L**	**H**	**L**	**H**	23.8 (21.2,26.6)	7.8 (6.2,9.7)	10.2 (8.4,12.3)
**Sensitivity: H H H H**						
**Specificity**				**Res. Cor.**	**Overall G^2**	**Overall χ^2**
**H**	**H**	**L**	**L**	28.9 (26.1,31.8)	6.5 (5.1,8.2)	7.1 (5.6,8.9)
**H**	**H**	**H**	**L**	13.7 (11.6,16.0)	5.0 (3.7,6.5)	7.2 (5.7,9.0)
**H**	**H**	**H**	**H**	8.4 (6.8,10.3)	16.8 (14.5,19.3)	25.3 (22.6,28.1)
**L**	**L**	**L**	**L**	23.7 (21.0,26.5)	12.3 (10.3,14.5)	13.0 (11.0,15.3)
**L**	**L**	**H**	**L**	31.9 (29.0,34.9)	19.6 (17.2,22.2)	20.6 (18.2,23.3)
**L**	**L**	**H**	**H**	27.1 (24.4,30.0)	37.2 (34.2,40.3)	47.6 (44.5,50.7)
**L**	**H**	**L**	**H**	28.6 (25.8,31.5)	9.0 (7.3,10.9)	11.6 (9.7,13.7)
**Sensitivity: L L L L**						
**Specificity**				**Res. Cor.**	**Overall G^2**	**Overall χ^2**
**H**	**H**	**L**	**L**	68.0 (64.6,71.2)	6.8 (5.1,8.7)	5.5 (4.0,7.3)
**H**	**H**	**H**	**L**	39.0 (36.0,42.2)	8.8 (7.1,10.7)	8.0 (6.3,9.8)
**H**	**H**	**H**	**H**	33.6 (30.7,36.7)	38.0 (34.9,41.1)	42.1 (39.0,45.3)
**L**	**L**	**L**	**L**	79.8 (74.3,84.6)	17.3 (12.8,22.6)	16.1 (11.8,21.3)
**L**	**L**	**H**	**L**	61.1 (56.8,65.3)	32.9 (28.9,37.1)	32.0 (28.0,36.1)
**L**	**L**	**H**	**H**	62.1 (58.4,65.6)	65.9 (62.3,69.3)	70.4 (67.0,73.7)
**L**	**H**	**L**	**H**	63.4 (60.1,66.7)	16.8 (14.3,19.4)	16.4 (14.0,19.1)
**Sensitivity: L L H L**						
**Specificity**				**Res. Cor.**	**Overall G^2**	**Overall χ^2**
**H**	**H**	**L**	**L**	48.8 (45.6,52.0)	6.1 (4.7,7.8)	6.5 (5.0,8.2)
**H**	**H**	**H**	**L**	36.7 (33.7,39.8)	13.5 (11.4,15.8)	12.4 (10.4,14.6)
**H**	**H**	**H**	**H**	30.1 (27.2,33.0)	66.0 (63.0,69.0)	75.3 (72.5,77.9)
**L**	**L**	**L**	**L**	58.8 (53.7,63.8)	25.6 (21.3,30.3)	25.1 (20.8,29.7)
**L**	**L**	**H**	**L**	63.6 (60.0,67.0)	43.8 (40.2,47.4)	42.5 (38.9,46.2)
**L**	**L**	**H**	**H**	81.6 (78.7,84.2)	87.0 (84.4,89.2)	86.7 (84.1,89.0)
**L**	**H**	**L**	**H**	60.9 (57.7,64.0)	38.8 (35.6,42.0)	47.4 (44.2,50.7)

Sensitivity: L L H H						
Specificity				Res. Cor.	Overall G^2	Overall χ^2
**H**	**H**	**L**	**L**	63.7 (60.6,66.6)	13.1 (11.0,15.3)	13.9 (11.8,16.2)
**H**	**H**	**H**	**L**	73.2 (70.4,76.0)	43.4 (40.3,46.5)	53.6 (50.5,56.7)
**H**	**H**	**H**	**H**	68.1 (65.1,71.0)	86.7 (84.4,88.7)	94.5 (92.9,95.8)
**L**	**L**	**L**	**L**	56.6 (52.5,60.7)	48.5 (44.4,52.7)	50.1 (45.9,54.2)
**L**	**L**	**H**	**L**	74.3 (70.9,77.4)	90.6 (88.3,92.7)	91.1 (88.7,93.0)
**L**	**L**	**H**	**H**	92.0 (90.0,93.7)	94.3 (92.6,95.7)	93.9 (92.2,95.4)
**L**	**H**	**L**	**H**	64.2 (61.0,67.3)	70.2 (67.2,73.1)	78.9 (76.2,81.5)

Table xii.3 Percentages of the time 95% CrIs for residual correlations did not included '0' for each pair, and percentages of the time the pairwise χ^2 or G^2 statistic for each pair indicated a lack of pairwise fit

Sensitivity: H H L L										
Specificity				Tool	T_1&T_2	T_1&T_3	T_1&T_4	T_2&T_3	T_2&T_4	T_3&T_4
				**Res. Cor.**	0.0 (0.0,0.4)	0.6 (0.2,1.4)	1.0 (0.4,1.8)	0.4 (0.1,1.1)	0.4 (0.1,1.1)	60.5 (57.2,63.6)
**H**	**H**	**L**	**L**	**Pairwise G^2**	0.0 (0.0,0.4)	0.0 (0.0,0.4)	0.0 (0.0,0.4)	0.0 (0.0,0.4)	0.1 (0.0,0.6)	4.2 (3.0,5.7)
				**Pairwise χ^2**	0.0 (0.0,0.4)	0.0 (0.0,0.4)	0.0 (0.0,0.4)	0.0 (0.0,0.4)	0.1 (0.0,0.6)	4.1 (2.9,5.6)
				**Res. Cor.**	0.0 (0.0,0.4)	0.0 (0.0,0.4)	0.7 (0.3,1.4)	0.0 (0.0,0.4)	0.9 (0.4,1.7)	22.4 (19.9,25.1)
**H**	**H**	**H**	**L**	**Pairwise G^2**	0.0 (0.0,0.4)	0.0 (0.0,0.4)	0.0 (0.0,0.4)	0.0 (0.0,0.4)	0.0 (0.0,0.4)	2.4 (1.5,3.6)
				**Pairwise χ^2**	0.0 (0.0,0.4)	0.0 (0.0,0.4)	0.0 (0.0,0.4)	0.0 (0.0,0.4)	0.0 (0.0,0.4)	2.3 (1.5,3.4)
				**Res. Cor.**	0.0 (0.0,0.4)	0.0 (0.0,0.4)	0.0 (0.0,0.4)	0.0 (0.0,0.4)	0.0 (0.0,0.4)	9.8 (8.0,11.8)
**H**	**H**	**H**	**H**	**Pairwise G^2**	0.0 (0.0,0.4)	0.0 (0.0,0.4)	0.0 (0.0,0.4)	0.0 (0.0,0.4)	0.0 (0.0,0.4)	1.7 (1.0,2.7)
				**Pairwise χ^2**	0.0 (0.0,0.4)	0.0 (0.0,0.4)	0.0 (0.0,0.4)	0.0 (0.0,0.4)	0.0 (0.0,0.4)	1.7 (1.0,2.7)
				**Res. Cor.**	0.0 (0.0,0.8)	7.6 (5.4,10.3)	10.5 (7.9,13.6)	8.0 (5.7,10.8)	5.3 (3.7,7.7)	38.7 (34.3,43.3)
**L**	**L**	**L**	**L**	**Pairwise G^2**	0.0 (0.0,0.8)	1.1 (0.3,2.4)	0.4 (0.1,1.5)	0.4 (0.1,1.5)	0.4 (0.1,1.5)	2.1 (1.0,3.8)
				**Pairwise χ^2**	0.0 (0.0,0.8)	1.1 (0.3,2.4)	0.4 (0.1,1.5)	0.4 (0.1,1.5)	0.4 (0.1,1.5)	2.1 (1.0,3.8)
				**Res. Cor.**	0.1 (0.0,0.6)	0.1 (0.0,0.6)	14.6 (12.4,17.1)	0.1 (0.0,0.6)	15.2 (12.9,17.6)	8.3 (6.6,10.3)
**L**	**L**	**H**	**L**	**Pairwise G^2**	0.0 (0.0,0.4)	0.0 (0.0,0.4)	1.1 (0.5,2.0)	0.0 (0.0,0.4)	1.3 (0.7,2.3)	0.3 (0.1,0.9)
				**Pairwise χ^2**	0.0 (0.0,0.4)	0.0 (0.0,0.4)	1.1 (0.5,2.0)	0.0 (0.0,0.4)	1.3 (0.7,2.3)	0.3 (0.1,0.9)
				**Res. Cor.**	3.0 (2.1,4.3)	0.5 (0.2,1.2)	0.4 (0.1,1.0)	0.5 (0.2,1.2)	0.4 (0.1,1.0)	0.6 (0.2,1.3)
**L**	**L**	**H**	**H**	**Pairwise G^2**	0.9 (0.4,1.7)	0.0 (0.0,0.4)	0.0 (0.0,0.4)	0.0 (0.0,0.4)	0.0 (0.0,0.4)	0.4 (0.1,1.0)
				**Pairwise χ^2**	0.9 (0.4,1.7)	0.0 (0.0,0.4)	0.0 (0.0,0.4)	0.0 (0.0,0.4)	0.0 (0.0,0.4)	0.4 (0.1,1.0)
				**Res. Cor.**	0.0 (0.0,0.4)	23.7 (21.1,26.5)	1.5 (0.9,2.5)	0.4 (0.1,1.0)	0.0 (0.0,0.4)	15.4 (13.2,17.8)
**L**	**H**	**L**	**H**	**Pairwise G^2**	0.0 (0.0,0.4)	2.1 (1.3,3.2)	0.1 (0.0,0.6)	0.0 (0.0,0.4)	0.0 (0.0,0.4)	1.4 (0.8,2.4)
				**Pairwise χ^2**	0.0 (0.0,0.4)	2.1 (1.3,3.2)	0.1 (0.0,0.6)	0.0 (0.0,0.4)	0.0 (0.0,0.4)	1.3 (0.7,2.2)
**Sensitivity: H H H L**										

Specificity				Tool	T_1&T_2	T_1&T_3	T_1&T_4	T_2&T_3	T_2&T_4	T_3&T_4
H	H	L	L	**Res. Cor.**	0.0 (0.0,0.4)	0.0 (0.0,0.4)	0.9 (0.4,1.7)	0.0 (0.0,0.4)	1.0 (0.5,1.8)	32.9 (30.0,35.9)
				**Pairwise G^2**	0.0 (0.0,0.4)	0.0 (0.0,0.4)	0.0 (0.0,0.4)	0.0 (0.0,0.4)	0.0 (0.0,0.4)	3.7 (2.6,5.1)
				**Pairwise χ^2**	0.0 (0.0,0.4)	0.0 (0.0,0.4)	0.0 (0.0,0.4)	0.0 (0.0,0.4)	0.0 (0.0,0.4)	3.6 (2.5,5.0)
H	H	H	L	**Res. Cor.**	0.0 (0.0,0.4)	0.0 (0.0,0.4)	1.9 (1.1,3.0)	0.0 (0.0,0.4)	1.4 (0.8,2.3)	5.3 (4.0,6.9)
				**Pairwise G^2**	0.0 (0.0,0.4)	0.0 (0.0,0.4)	0.2 (0.0,0.7)	0.0 (0.0,0.4)	0.0 (0.0,0.4)	0.7 (0.3,1.4)
				**Pairwise χ^2**	0.0 (0.0,0.4)	0.0 (0.0,0.4)	0.2 (0.0,0.7)	0.0 (0.0,0.4)	0.0 (0.0,0.4)	0.7 (0.3,1.4)
H	H	H	H	**Res. Cor.**	0.0 (0.0,0.4)	0.0 (0.0,0.4)	0.0 (0.0,0.4)	0.0 (0.0,0.4)	0.0 (0.0,0.4)	3.6 (2.5,4.9)
				**Pairwise G^2**	0.0 (0.0,0.4)	0.0 (0.0,0.4)	0.0 (0.0,0.4)	0.0 (0.0,0.4)	0.0 (0.0,0.4)	1.8 (1.1,2.8)
				**Pairwise χ^2**	0.0 (0.0,0.4)	0.0 (0.0,0.4)	0.0 (0.0,0.4)	0.0 (0.0,0.4)	0.0 (0.0,0.4)	1.7 (1.0,2.7)
L	L	L	L	**Res. Cor.**	0.1 (0.0,0.6)	0.7 (0.3,1.5)	13.1 (10.9,15.5)	1.5 (0.8,2.6)	13.0 (10.8,15.4)	16.9 (14.5,19.6)
				**Pairwise G^2**	0.0 (0.0,0.4)	0.1 (0.0,0.6)	1.4 (0.7,2.4)	0.0 (0.0,0.4)	0.7 (0.3,1.5)	1.5 (0.8,2.6)
				**Pairwise χ^2**	0.0 (0.0,0.4)	0.1 (0.0,0.6)	1.3 (0.6,2.3)	0.0 (0.0,0.4)	0.7 (0.3,1.5)	1.5 (0.8,2.6)
L	L	H	L	**Res. Cor.**	1.5 (0.9,2.5)	0.0 (0.0,0.4)	19.6 (17.2,22.2)	0.0 (0.0,0.4)	19.9 (17.5,22.5)	1.7 (1.0,2.7)
				**Pairwise G^2**	0.2 (0.0,0.7)	0.0 (0.0,0.4)	1.6 (0.9,2.6)	0.0 (0.0,0.4)	0.9 (0.4,1.7)	0.2 (0.0,0.7)
				**Pairwise χ^2**	0.3 (0.1,0.9)	0.0 (0.0,0.4)	1.6 (0.9,2.6)	0.0 (0.0,0.4)	0.9 (0.4,1.7)	0.2 (0.0,0.7)
L	L	H	H	**Res. Cor.**	12.0 (10.1,14.2)	0.1 (0.0,0.6)	1.4 (0.8,2.3)	0.0 (0.0,0.4)	1.5 (0.8,2.5)	0.2 (0.0,0.7)
				**Pairwise G^2**	1.8 (1.1,2.8)	0.0 (0.0,0.4)	0.0 (0.0,0.4)	0.0 (0.0,0.4)	0.3 (0.1,0.9)	0.1 (0.0,0.6)
				**Pairwise χ^2**	1.8 (1.1,2.8)	0.0 (0.0,0.4)	0.0 (0.0,0.4)	0.0 (0.0,0.4)	0.3 (0.1,0.9)	0.1 (0.0,0.6)
L	H	L	H	**Res. Cor.**	0.0 (0.0,0.4)	16.0 (13.8,18.5)	2.8 (1.9,4.0)	0.0 (0.0,0.4)	0.0 (0.0,0.4)	7.4 (5.9,9.2)
				**Pairwise G^2**	0.0 (0.0,0.4)	1.8 (1.1,2.8)	0.1 (0.0,0.6)	0.0 (0.0,0.4)	0.0 (0.0,0.4)	1.7 (1.0,2.7)
				**Pairwise χ^2**	0.0 (0.0,0.4)	1.9 (1.2,3.0)	0.2 (0.0,0.7)	0.0 (0.0,0.4)	0.0 (0.0,0.4)	1.5 (0.8,2.5)
**Sensitivity: H H H H**										
**Specificity**				**Tool**	**T_1&T_2**	**T_1&T_3**	**T_1&T_4**	**T_2&T_3**	**T_2&T_4**	**T_3&T_4**
H	H	L	L	**Res. Cor.**	0.0 (0.0,0.4)	0.5 (0.2,1.2)	0.3 (0.1,0.9)	0.4 (0.1,1.0)	0.5 (0.2,1.2)	27.8 (25.0,30.7)
				**Pairwise G^2**	0.0 (0.0,0.4)	0.1 (0.0,0.6)	0.0 (0.0,0.4)	0.0 (0.0,0.4)	0.0 (0.0,0.4)	2.7 (1.8,3.9)
				**Pairwise χ^2**	0.0 (0.0,0.4)	0.1 (0.0,0.6)	0.0 (0.0,0.4)	0.0 (0.0,0.4)	0.0 (0.0,0.4)	2.7 (1.8,3.9)
H	H	H	L	**Res. Cor.**	0.0 (0.0,0.4)	0.0 (0.0,0.4)	1.2 (0.6,2.1)	0.0 (0.0,0.4)	0.7 (0.3,1.4)	11.9 (10.0,14.1)
				**Pairwise G^2**	0.0 (0.0,0.4)	0.0 (0.0,0.4)	0.0 (0.0,0.4)	0.0 (0.0,0.4)	0.3 (0.1,0.9)	3.5 (2.5,4.8)
				**Pairwise χ^2**	0.0 (0.0,0.4)	0.0 (0.0,0.4)	0.0 (0.0,0.4)	0.0 (0.0,0.4)	0.3 (0.1,0.9)	3.0 (2.0,4.3)
H	H	H	H	**Res. Cor.**	0.1 (0.0,0.6)	0.0 (0.0,0.4)	0.0 (0.0,0.4)	0.0 (0.0,0.4)	0.0 (0.0,0.4)	8.4 (6.8,10.3)
				**Pairwise G^2**	0.0 (0.0,0.4)	0.0 (0.0,0.4)	0.0 (0.0,0.4)	0.0 (0.0,0.4)	0.0 (0.0,0.4)	4.5 (3.3,6.0)
				**Pairwise χ^2**	0.0 (0.0,0.4)	0.0 (0.0,0.4)	0.0 (0.0,0.4)	0.0 (0.0,0.4)	0.0 (0.0,0.4)	3.7 (2.6,5.1)
L	L	L	L	**Res. Cor.**	1.8 (1.1,2.9)	5.5 (4.2,7.2)	4.3 (3.1,5.8)	3.8 (2.7,5.2)	6.4 (4.9,8.1)	5.7 (4.4,7.4)
				**Pairwise G^2**	0.0 (0.0,0.4)	0.4 (0.1,1.0)	0.3 (0.1,0.9)	0.3 (0.1,0.9)	0.4 (0.1,1.0)	0.5 (0.2,1.2)
				**Pairwise χ^2**	0.0 (0.0,0.4)	0.5 (0.2,1.2)	0.3 (0.1,0.9)	0.3 (0.1,0.9)	0.4 (0.1,1.0)	0.5 (0.2,1.2)
L	L	H	L	**Res. Cor.**	8.5 (6.9,10.4)	0.2 (0.0,0.7)	13.4 (11.4,15.7)	0.0 (0.0,0.4)	13.8 (11.7,16.1)	3.7 (2.6,5.1)
				**Pairwise G^2**	0.9 (0.4,1.7)	0.0 (0.0,0.4)	1.0 (0.5,1.8)	0.0 (0.0,0.4)	1.0 (0.5,1.8)	1.2 (0.6,2.1)
				**Pairwise χ^2**	0.8 (0.3,1.6)	0.0 (0.0,0.4)	1.1 (0.6,2.0)	0.0 (0.0,0.4)	1.1 (0.6,2.0)	0.9 (0.4,1.7)
L	L	H	H	**Res. Cor.**	25.8 (23.1,28.6)	0.0 (0.0,0.4)	0.2 (0.0,0.7)	0.5 (0.2,1.2)	0.6 (0.2,1.3)	1.4 (0.8,2.3)
				**Pairwise G^2**	3.0 (2.0,4.3)	0.0 (0.0,0.4)	0.0 (0.0,0.4)	0.0 (0.0,0.4)	0.1 (0.0,0.6)	0.9 (0.4,1.7)

				**Pairwise χ²**	2.8 (1.9,4.0)	0.0 (0.0,0.4)	0.0 (0.0,0.4)	0.0 (0.0,0.4)	0.1 (0.0,0.6)	0.9 (0.4,1.7)
**L**	**H**	**L**	**H**	**Res. Cor.**	0.4 (0.1,1.0)	23.6 (21.0,26.4)	0.7 (0.3,1.4)	0.3 (0.1,0.9)	0.0 (0.0,0.4)	6.7 (5.2,8.4)
				**Pairwise G²**	0.1 (0.0,0.6)	1.4 (0.8,2.3)	0.2 (0.0,0.7)	0.1 (0.0,0.6)	0.0 (0.0,0.4)	1.9 (1.1,3.0)
				**Pairwise χ²**	0.1 (0.0,0.6)	1.4 (0.8,2.3)	0.2 (0.0,0.7)	0.1 (0.0,0.6)	0.0 (0.0,0.4)	1.8 (1.1,2.8)
**Sensitivity: L L L L**										
**Specificity**				**Tool**	**T_1&T_2**	**T_1&T_3**	**T_1&T_4**	**T_2&T_3**	**T_2&T_4**	**T_3&T_4**
**H**	**H**	**L**	**L**	**Res. Cor.**	0.0 (0.0,0.5)	4.7 (3.3,6.4)	3.2 (2.1,4.7)	4.7 (3.3,6.4)	3.6 (2.4,5.1)	64.3 (60.8,67.6)
				**Pairwise G²**	0.0 (0.0,0.5)	0.1 (0.0,0.7)	0.0 (0.0,0.5)	0.4 (0.1,1.1)	0.1 (0.0,0.7)	4.7 (3.3,6.4)
				**Pairwise χ²**	0.0 (0.0,0.5)	0.1 (0.0,0.7)	0.0 (0.0,0.5)	0.3 (0.0,0.9)	0.1 (0.0,0.7)	4.7 (3.3,6.4)
**H**	**H**	**H**	**L**	**Res. Cor.**	0.0 (0.0,0.4)	0.0 (0.0,0.4)	5.4 (4.0,7.0)	0.0 (0.0,0.4)	5.5 (4.1,7.1)	33.5 (30.5,36.5)
				**Pairwise G²**	0.0 (0.0,0.4)	0.0 (0.0,0.4)	0.1 (0.0,0.6)	0.0 (0.0,0.4)	0.4 (0.1,1.1)	6.5 (5.0,8.3)
				**Pairwise χ²**	0.0 (0.0,0.4)	0.0 (0.0,0.4)	0.1 (0.0,0.6)	0.0 (0.0,0.4)	0.4 (0.1,1.1)	6.5 (5.0,8.3)
**H**	**H**	**H**	**H**	**Res. Cor.**	0.1 (0.0,0.6)	0.0 (0.0,0.4)	0.0 (0.0,0.4)	0.0 (0.0,0.4)	0.2 (0.0,0.7)	33.4 (30.5,36.5)
				**Pairwise G²**	0.1 (0.0,0.6)	0.0 (0.0,0.4)	0.0 (0.0,0.4)	0.0 (0.0,0.4)	0.0 (0.0,0.4)	22.6 (20.1,25.4)
				**Pairwise χ²**	0.1 (0.0,0.6)	0.0 (0.0,0.4)	0.0 (0.0,0.4)	0.0 (0.0,0.4)	0.0 (0.0,0.4)	23.3 (20.7,26.1)
**L**	**L**	**L**	**L**	**Res. Cor.**	0.0 (0.0,1.5)	24.6 (19.4,30.4)	27.8 (22.3,33.8)	29.4 (23.8,35.5)	26.6 (21.2,32.6)	35.5 (29.5,41.8)
				**Pairwise G²**	0.4 (0.0,2.2)	1.6 (0.4,4.1)	1.2 (0.3,3.5)	0.4 (0.0,2.2)	0.8 (0.1,2.9)	1.2 (0.3,3.5)
				**Pairwise χ²**	0.4 (0.0,2.2)	1.6 (0.4,4.1)	1.2 (0.3,3.5)	0.4 (0.0,2.2)	0.8 (0.1,2.9)	1.2 (0.3,3.5)
**L**	**L**	**H**	**L**	**Res. Cor.**	1.1 (0.4,2.4)	3.0 (1.7,4.8)	30.1 (26.2,34.2)	2.8 (1.6,4.6)	32.9 (28.9,37.1)	18.8 (15.6,22.4)
				**Pairwise G²**	1.7 (0.8,3.2)	0.4 (0.0,1.4)	2.6 (1.4,4.4)	0.2 (0.0,1.0)	2.4 (1.3,4.1)	4.7 (3.1,6.9)
				**Pairwise χ²**	1.7 (0.8,3.2)	0.4 (0.0,1.4)	2.6 (1.4,4.4)	0.2 (0.0,1.0)	2.4 (1.3,4.1)	4.7 (3.1,6.9)
**L**	**L**	**H**	**H**	**Res. Cor.**	50.8 (47.154.5)	6.3 (4.6,8.3)	5.5 (3.9,7.4)	6.7 (5.0,8.8)	8.4 (6.5,10.6)	2.5 (1.5,3.9)
				**Pairwise G²**	31.8 (28.4,35.3)	0.5 (0.1,1.4)	0.5 (0.1,1.4)	0.7 (0.2,1.6)	0.7 (0.2,1.6)	5.8 (4.2,7.7)
				**Pairwise χ²**	31.8 (28.4,35.3)	0.5 (0.1,1.4)	0.5 (0.1,1.4)	0.7 (0.2,1.6)	0.7 (0.2,1.6)	6.3 (4.6,8.3)
**L**	**H**	**L**	**H**	**Res. Cor.**	0.0 (0.0,0.4)	42.3 (39.0,45.7)	20.5 (17.9,23.4)	5.4 (4.0,7.1)	0.0 (0.0,0.4)	25.2 (22.3,28.3)
				**Pairwise G²**	0.0 (0.0,0.4)	3.6 (2.5,5.1)	1.9 (1.1,3.0)	0.2 (0.0,0.8)	0.0 (0.0,0.4)	4.6 (3.3,6.2)
				**Pairwise χ²**	0.0 (0.0,0.4)	3.6 (2.5,5.1)	1.9 (1.1,3.0)	0.2 (0.0,0.8)	0.0 (0.0,0.4)	4.6 (3.3,6.2)
**Sensitivity: L L H L**										
**Specificity**				**Tool**	**T_1&T_2**	**T_1&T_3**	**T_1&T_4**	**T_2&T_3**	**T_2&T_4**	**T_3&T_4**
**H**	**H**	**L**	**L**	**Res. Cor.**	0.0 (0.0,0.4)	0.1 (0.0,0.6)	4.5 (3.3,6.0)	0.0 (0.0,0.4)	4.6 (3.4,6.2)	44.4 (41.3,47.6)
				**Pairwise G²**	0.0 (0.0,0.4)	0.0 (0.0,0.4)	0.2 (0.0,0.7)	0.0 (0.0,0.4)	0.1 (0.0,0.6)	6.2 (4.8,7.9)
				**Pairwise χ²**	0.0 (0.0,0.4)	0.0 (0.0,0.4)	0.2 (0.0,0.7)	0.0 (0.0,0.4)	0.1 (0.0,0.6)	6.1 (4.7,7.8)
**H**	**H**	**H**	**L**	**Res. Cor.**	0.0 (0.0,0.4)	0.0 (0.0,0.4)	7.8 (6.2,9.6)	0.0 (0.0,0.4)	6.8 (5.3,8.5)	27.5 (24.8,30.4)
				**Pairwise G²**	0.0 (0.0,0.4)	0.0 (0.0,0.4)	0.7 (0.3,1.4)	0.0 (0.0,0.4)	0.3 (0.1,0.9)	8.1 (6.5,10.0)
				**Pairwise χ²**	0.0 (0.0,0.4)	0.0 (0.0,0.4)	0.7 (0.3,1.4)	0.0 (0.0,0.4)	0.3 (0.1,0.9)	8.1 (6.5,10.0)
**H**	**H**	**H**	**H**	**Res. Cor.**	3.9 (2.8,5.3)	0.0 (0.0,0.4)	0.8 (0.3,1.6)	0.0 (0.0,0.4)	0.7 (0.3,1.4)	25.0 (22.4,27.8)
				**Pairwise G²**	6.1 (4.7,7.8)	0.0 (0.0,0.4)	0.1 (0.0,0.6)	0.0 (0.0,0.4)	0.0 (0.0,0.4)	29.7 (26.9,32.7)
				**Pairwise χ²**	5.6 (4.3,7.2)	0.0 (0.0,0.4)	0.1 (0.0,0.6)	0.0 (0.0,0.4)	0.0 (0.0,0.4)	29.2 (26.4,32.2)
**L**	**L**	**L**	**L**	**Res. Cor.**	0.0 (0.0,1.0)	7.9 (5.4,11.1)	25.1 (20.8,29.7)	6.6 (4.3,9.6)	25.3 (21.0,30.0)	27.4 (23.0,32.2)
				**Pairwise G²**	0.0 (0.0,1.0)	0.0 (0.0,1.0)	2.1 (0.9,4.1)	0.0 (0.0,1.0)	0.3 (0.0,1.5)	5.0 (3.0,7.7)
				**Pairwise χ²**	0.0 (0.0,1.0)	0.0 (0.0,1.0)	2.1 (0.9,4.1)	0.0 (0.0,1.0)	0.3 (0.0,1.5)	4.7 (2.8,7.4)

**L**	**L**	**H**	**L**	**Res. Cor.**	1.5 (0.7,2.7)	2.4 (1.5,3.8)	34.0 (30.6,37.6)	1.9 (1.0,3.2)	33.7 (30.3,37.3)	22.0 (19.0,25.1)
				**Pairwise G^2**	0.4 (0.1,1.2)	0.3 (0.0,1.0)	2.8 (1.8,4.3)	0.0 (0.0,0.5)	3.4 (2.2,5.0)	9.6 (7.6,12.0)
				**Pairwise χ^2**	0.4 (0.1,1.2)	0.3 (0.0,1.0)	3.0 (1.9,4.5)	0.0 (0.0,0.5)	3.4 (2.2,5.0)	9.5 (7.5,11.8)
**L**	**L**	**H**	**H**	**Res. Cor.**	77.9 (74.8,80.7)	0.8 (0.3,1.7)	8.3 (6.5,10.5)	0.9 (0.4,1.8)	10.1 (8.1,12.4)	0.3 (0.0,0.9)
				**Pairwise G^2**	43.4 (39.8,46.9)	0.0 (0.0,0.5)	0.5 (0.1,1.3)	0.0 (0.0,0.5)	1.3 (0.6,2.3)	1.4 (0.7,2.5)
				**Pairwise χ^2**	43.4 (39.8,46.9)	0.0 (0.0,0.5)	0.5 (0.1,1.3)	0.0 (0.0,0.5)	1.3 (0.6,2.3)	1.4 (0.7,2.5)
**L**	**H**	**L**	**H**	**Res. Cor.**	1.7 (1.0,2.8)	32.4 (29.4,35.6)	22.8 (20.2,25.7)	0.0 (0.0,0.4)	0.1 (0.0,0.6)	33.8 (30.8,37.0)
				**Pairwise G^2**	2.0 (1.2,3.2)	5.7 (4.3,7.4)	4.1 (2.9,5.6)	0.0 (0.0,0.4)	0.0 (0.0,0.4)	26.9 (24.1,29.9)
				**Pairwise χ^2**	1.8 (1.1,2.9)	5.7 (4.3,7.4)	4.3 (3.1,5.8)	0.0 (0.0,0.4)	0.0 (0.0,0.4)	25.2 (22.5,28.1)
**Sensitivity: L L H H**										
**Specificity**				**Tool**	**T_1&T_2**	**T_1&T_3**	**T_1&T_4**	**T_2&T_3**	**T_2&T_4**	**T_3&T_4**
**H**	**H**	**L**	**L**	**Res. Cor.**	0.0 (0.0,0.4)	0.7 (0.3,1.4)	0.4 (0.1.1.0)	0.3 (0.1,0.9)	0.6 (0.2,1.3)	63.0 (59.9,66.0)
				**Pairwise G^2**	0.0 (0.0,0.4)	0.1 (0.0,0.6)	0.0 (0.0,0.4)	0.0 (0.0,0.4)	0.2 (0.0,0.7)	20.5 (18.0,23.1)
				**Pairwise χ^2**	0.0 (0.0,0.4)	0.1 (0.0,0.6)	0.0 (0.0,0.4)	0.0 (0.0,0.4)	0.1 (0.0,0.6)	20.5 (18.0,23.1)
**H**	**H**	**H**	**L**	**Res. Cor.**	0.4 (0.1,1.0)	0.0 (0.0,0.4)	2.7 (1.8,3.9)	0.1 (0.0,0.6)	3.7 (2.6,5.1)	71.0 (68.1,73.8)
				**Pairwise G^2**	0.5 (0.2,1.2)	0.0 (0.0,0.4)	0.3 (0.1,0.9)	0.1 (0.0,0.6)	0.3 (0.1,0.9)	55.5 (52.4,58.6)
				**Pairwise χ^2**	0.5 (0.2,1.2)	0.0 (0.0,0.4)	0.2 (0.0,0.6)	0.1 (0.0,0.6)	0.3 (0.1,0.9)	53.1 (50.0,56.2)
**H**	**H**	**H**	**H**	**Res. Cor.**	62.4 (59.3,65.4)	0.0 (0.0,0.4)	0.0 (0.0,0.4)	0.0 (0.0,0.4)	0.0 (0.0,0.4)	6.2 (4.8,7.9)
				**Pairwise G^2**	62.7 (59.6,65.7)	0.0 (0.0,0.4)	0.0 (0.0,0.4)	0.0 (0.0,0.4)	0.0 (0.0,0.4)	11.8 (9.9,14.0)
				**Pairwise χ^2**	61.9 (58.8,64.9)	0.0 (0.0,0.4)	0.0 (0.0,0.4)	0.0 (0.0,0.4)	0.0 (0.0,0.4)	11.6 (9.7,13.8)
**L**	**L**	**L**	**L**	**Res. Cor.**	5.3 (3.7,7.5)	14.6 (11.9,17.8)	14.3 (11.5,17.4)	15.0 (12.2,18.1)	9.8 (7.5,12.5)	28.9 (25.3,32.8)
				**Pairwise G^2**	3.1 (1.8,4.9)	2.4 (1.3,4.0)	2.1 (1.1,3.6)	1.9 (0.9,3.4)	1.9 (0.9,3.4)	16.2 (13.3,19.4)
				**Pairwise χ^2**	3.1 (1.8,4.9)	2.4 (1.3,4.0)	2.1 (1.1,3.6)	1.9 (0.9,3.4)	1.9 (0.9,3.4)	16.2 (13.3,19.4)
**L**	**L**	**H**	**L**	**Res. Cor.**	50.8 (47.1,54.5)	2.2 (1.3,3.5)	22.1 (19.2,25.3)	1.7 (0.9,2.9)	21.6 (18.7,24.8)	9.5 (7.5,11.9)
				**Pairwise G^2**	30.0 (26.7,33.5)	0.4 (0.1,1.2)	3.6 (2.3,5.2)	0.6 (0.2,1.4)	3.0 (1.9,4.5)	17.6 (14.9,20.6)
				**Pairwise χ^2**	30.1 (26.8,33.6)	0.4 (0.1,1.2)	3.6 (2.3,5.2)	0.6 (0.2,1.4)	3.0 (1.9,4.5)	16.4 (13.8,19.3)
**L**	**L**	**H**	**H**	**Res. Cor.**	92.0 (90.0,93.7)	0.7 (0.2,1.4)	1.0 (0.5,1.9)	0.6 (0.2,1.3)	0.7 (0.2,1.4)	0.0 (0.0,0.4)
				**Pairwise G^2**	53.7 (50.4,57.0)	0.1 (0.0,0.6)	0.1 (0.0,0.6)	0.0 (0.0,0.4)	0.0 (0.0,0.4)	0.0 (0.0,0.4)
				**Pairwise χ^2**	53.7 (50.4,57.0)	0.1 (0.0,0.6)	0.1 (0.0,0.6)	0.0 (0.0,0.4)	0.0 (0.0,0.4)	0.0 (0.0,0.4)
**L**	**H**	**L**	**H**	**Res. Cor.**	10.7 (8.8,12.8)	36.1 (33.1,39.3)	13.7 (11.5,16.0)	0.5 (0.2,1.2)	0.0 (0.0,0.4)	33.9 (30.9,37.0)
				**Pairwise G^2**	9.4 (7.6,11.5)	4.3 (3.1,5.8)	3.3 (2.2,4.6)	0.0 (0.0,0.4)	0.0 (0.0,0.4)	39.8 (36.7,43.0)
				**Pairwise χ^2**	9.1 (3.2,6.0)	4.4 (3.2,6.0)	3.6 (2.5,5.0)	0.0 (0.0,0.4)	0.0 (0.0,0.4)	38.1 (35.0,41.3)

Table xii.4 Mean absolute biases (x10^{-2}) of posterior medians of each parameter obtained from the conditional independence model

**Sensitivity: H H L L**												
**Specificity**				**π**	**Se_1**	**Se_2**	**Se_3**	**Se_4**	**Sp_1**	**Sp_2**	**Sp_3**	**Sp_4**
**H**	**H**	**L**	**L**	2.2 (2.1,2.4)	-1.7 (-2.0,-1.3)	-1.4 (-1.7,-1.1)	-1.2 (-1.6,-0.9)	-1.1 (-1.4,-0.8)	1.5 (1.4,1.6)	1.7 (1.6,1.9)	0.1 (0.0,0.3)	0.2 (0.1,0.4)
**H**	**H**	**H**	**L**	-0.2 (-0.4,-0.1)	3.1 (2.8,3.3)	3.0 (2.8,3.2)	-0.9 (-1.3,-0.6)	-0.4 (-0.8,-0.1)	0.4 (0.3,0.5)	0.3 (0.2,0.4)	-0.6 (-0.7,-0.5)	-0.3 (-0.4,-0.1)
**H**	**H**	**H**	**H**	-0.6 (+0.7,-0.5)	3.6 (3.4,3.8)	3.6 (3.4,3.8)	-0.5 (-0.8,-0.1)	-0.8 (-1.2,-0.5)	0.1 (0.0,0.2)	0.2 (0.0,0.3)	-0.7 (-0.8,-0.6)	-0.7 (-0.8,-0.6)

**L**	**L**	**L**	**L**	10.5 (9.8,11.2)	-7.0 (-7.6,-6.4)	-7.1 (-7.7,-6.4)	-2.8 (-3.4,-2.2)	-3.0 (-3.6,-2.4)	4.0 (3.6,4.5)	4.2 (3.7,4.7)	1.4 (1.1,1.7)	1.6 (1.3,1.9)
**L**	**L**	**H**	**L**	1.4 (1.1,1.7)	1.6 (1.3,1.8)	1.3 (1.0,1.6)	-2.2 (-2.8,-1.6)	-1.0 (-1.4,-0.6)	1.2 (1.0,1.5)	1.1 (0.9,1.3)	-0.2 (-0.4,-0.1)	0.0 (-0.2,0.1)
**L**	**L**	**H**	**H**	-0.3 (-0.5,-0.1)	2.8 (2.6,3.1)	2.7 (2.4,2.9)	-1.2 (-1.6,-0.7)	-1.0 (-1.4,-0.5)	0.6 (0.4,0.8)	0.7 (0.5,0.9)	-0.6 (-0.7,-0.5)	-0.6 (-0.7,-0.5)
**L**	**H**	**L**	**H**	0.2 (0.1,0.4)	3.2 (3.0,3.4)	0.9 (0.6,1.2)	-0.6 (-1.0,-0.3)	-1.0 (-1.4,-0.6)	1.1 (0.9,1.2)	0.3 (0.2,0.5)	-0.1 (-0.2,0.1)	-0.3 (-0.4,-0.2)
**Sensitivity: H H H L**												
**Specificity**				π	Se_1	Se_2	Se_3	Se_4	Sp_1	Sp_2	Sp_3	Sp_4
**H**	**H**	**L**	**L**	0.1 (0.0,0.2)	2.2 (1.9,2.4)	2.2 (2.0,2.5)	-1.1 (-1.4,-0.9)	-0.7 (-1.0,-0.3)	0.5 (0.4,0.6)	0.5 (0.4,0.6)	-0.3 (-0.4,-0.1)	-0.3 (-0.5,-0.2)
**H**	**H**	**H**	**L**	-0.8 (-0.9,-0.7)	4.2 (4.0,4.4)	4.1 (3.9,4.3)	-0.7 (-1.0,-0.5)	-0.4 (-0.7,0.0)	-0.1 (-0.2,0.0)	0.0 (-0.1,0.1)	-1.1 (-1.2,-1.0)	-0.4 (-0.6,-0.3)
**H**	**H**	**H**	**H**	-0.8 (-0.9,-0.7)	4.0 (3.9,4.2)	4.0 (3.8,4.2)	-0.8 (-1.1,-0.6)	-0.4 (-0.7,0.0)	0.0 (-0.1,0.1)	0.0 (-0.1,0.1)	-1.2 (-1.3,-1.1)	-0.8 (-0.9,-0.7)
**L**	**L**	**L**	**L**	4.0 (3.7,4.3)	-0.6 (-0.9,-0.3)	-1.3 (-1.6,-0.9)	-5.3 (-5.7,-4.8)	-1.3 (-1.7,-0.9)	2.3 (2.1,2.5)	2.2 (1.9,2.4)	0.7 (0.5,0.9)	0.7 (0.5,0.9)
**L**	**L**	**H**	**L**	0.7 (0.5,0.9)	3.5 (3.3,3.7)	3.4 (3.2,3.6)	-5.9 (-6.3,-5.4)	-0.5 (-0.8,-0.1)	1.3 (1.1,1.4)	1.4 (1.2,1.5)	-1.2 (-1.3,-1.0)	-0.1 (-0.3,0.1)
**L**	**L**	**H**	**H**	-0.5 (-0.6,-0.3)	3.5 (3.3,3.7)	3.5 (3.3,3.7)	-1.7 (-2.0,-1.3)	-0.6 (-1.0,-0.2)	0.3 (0.2,0.5)	0.5 (0.3,0.7)	-1.2 (-1.3,-1.0)	-0.7 (-0.8,-0.6)
**L**	**H**	**L**	**H**	-0.1 (-0.3,0.0)	3.5 (3.3,3.7)	2.5 (2.3,2.7)	-1.2 (-1.5,-1.0)	-1.4 (-1.7,-1.0)	0.7 (0.5,0.8)	0.3 (0.2,0.4)	-0.5 (-0.7,-0.5)	-0.6 (-0.7,-0.5)
**Sensitivity: H H H H**												
**Specificity**				π	Se_1	Se_2	Se_3	Se_4	Sp_1	Sp_2	Sp_3	Sp_4
**H**	**H**	**L**	**L**	-0.5 (-0.6,-0.4)	3.3 (3.1,3.5)	3.4 (3.2,3.6)	-1.2 (-1.5,-1.0)	-1.0 (-1.2,-0.8)	0.2 (0.1,0.3)	0.4 (0.3,0.5)	-0.8 (-0.9,-0.6)	-0.5 (-0.7,-0.4)
**H**	**H**	**H**	**L**	-1.0 (-1.1,-0.8)	4.3 (4.1,4.5)	4.1 (3.9,4.3)	-0.8 (-1.1,-0.6)	-0.8 (-1.1,-0.6)	0.2 (0.1,0.3)	0.0 (-0.1,0.1)	-1.2 (-1.3,-1.1)	-0.7 (-0.8,-0.5)
**H**	**H**	**H**	**H**	-0.9 (-1.0,-0.7)	3.9 (3.7,4.1)	4.0 (3.8,4.2)	-0.9 (-1.1,-0.6)	-0.6 (-0.8,-0.4)	0.0 (-0.1,0.1)	0.1 (0.0,0.2)	-1.0 (-1.1,-0.9)	-1.1 (-1.2,-1.0)
**L**	**L**	**L**	**L**	1.8 (1.6,2.0)	1.4 (1.1,1.6)	1.4 (1.1,1.6)	-3.0 (-3.3,-2.6)	-3.2 (-3.5,-2.9)	1.2 (1.0,1.3)	1.2 (1.0,1.3)	0.1 (0.0,0.3)	-0.1 (-0.2,0.1)
**L**	**L**	**H**	**L**	-0.4 (-0.5,-0.3)	3.8 (3.6,4.0)	3.9 (3.7,4.1)	-2.7 (-3.1,-2.4)	-0.7 (-1.0,-0.5))	0.7 (0.5,0.8)	0.5 (0.4,0.7)	-1.3 (-1.4,-1.2)	-0.5 (-0.7,-0.4)
**L**	**L**	**H**	**H**	-0.8 (-1.0,-0.7)	3.2 (3.0,3.4)	3.2 (3.0,3.4)	-0.3 (-0.6,-0.1)	-0.5 (-0.8,-0.3)	0.1 (-0.1,0.2)	0.3 (0.2,0.5)	-1.0 (-1.1,-0.9)	-1.0 (-1.1,-0.9)
**L**	**H**	**L**	**H**	-0.9 (-1.0,-0.8)	4.0 (3.8,4.2)	3.9 (3.7,4.1)	-0.6 (-0.8,-0.4)	-0.7 (-1.0,-0.5)	0.4 (0.2,0.5)	-0.1 (-0.2,0.0)	-0.6 (-0.8,-0.5)	-1.1 (-1.2,-1.0)
**Sensitivity: L L L L**												
**Specificity**				π	Se_1	Se_2	Se_3	Se_4	Sp_1	Sp_2	Sp_3	Sp_4
**H**	**H**	**L**	**L**	-3.7 (-3.9,-3.5)	17.9 (17.4,18.5)	17.8 (17.2,18.4)	-0.8 (-1.3,-0.3)	-0.6 (-1.1,-0.1)	1.0 (0.8,1.2)	0.9 (0.8,1.1)	-1.2 (-1.3-1.0)	-1.1 (-1.3,-0.9)
**H**	**H**	**H**	**L**	-5.5 (-5.6,-5.3)	21.3 (20.7,21.8)	21.4 (20.9,21.9)	-1.1 (-1.6,-0.6)	0.4 (0.0,0.8)	0.2 (0.1,0.3)	0.1 (0.0,0.3)	-3.6 (-3.7,-3.5)	-1.3 (-1.5,-1.2)
**H**	**H**	**H**	**H**	-5.0 (-5.1,-4.8)	19.1 (18.6,19.6)	18.6 (18.1,19.1)	0.2 (-0.3,0.6)	0.2 (-0.2,0.7)	0.2 (0.1,0.3)	0.2 (0.1,0.3)	-2.9 (-3.0,-2.8)	-2.9 (-3.0,-2.8)
**L**	**L**	**L**	**L**	2.7 (1.7,3.8)	13.3 (12.2,14.4)	12.4 (11.3,13.5)	-2.7 (-3.7,-1.6)	-3.1 (-4.1,-2.1)	4.5 (3.9,5.0)	4.3 (3.8,4.8)	-0.3 (-0.6,0.1)	-0.4 (-0.8,0.0)
**L**	**L**	**H**	**L**	-5.4 (-5.8,-4.9)	22.4 (21.7,23.1)	21.7 (21.0,22.4)	-6.2 (-7.3,-5.1)	1.7 (0.9,2.4)	2.7 (2.4,3.0)	2.2 (1.9,2.5)	-4.9 (-5.1,-4.7)	-1.1 (-1.3,-0.8)
**L**	**L**	**H**	**H**	-5.0 (-5.3,-4.6)	12.8 (11.9,13.6)	12.5 (11.6,13.3)	3.3 (2.5,4.1)	3.7 (2.9,4.5)	0.7 (0.5,0.9)	0.7 (0.5,0.9)	-2.6 (-2.8,-2.4)	-2.5 (-2.7,-2.3)
**L**	**H**	**L**	**H**	-6.1 (-6.3,-5.9)	20.5 (19.9,21.0)	23.8 (23.3,24.3)	0.2 (-0.3,0.7)	-0.8 (-1.5,-0.1)	1.7 (1.5,1.8)	0.2 (0.0,0.3)	-1.5 (-1.7,-1.3)	-3.8 (-4.0,-3.7)
**Sensitivity: L L H L**												
**Specificity**				π	Se_1	Se_2	Se_3	Se_4	Sp_1	Sp_2	Sp_3	Sp_4
**H**	**H**	**L**	**L**	-5.2 (-5.4,-5.1)	20.8 (20.3,21.3)	21.2 (20.7,21.7)	-1.9 (-2.2,-1.5)	-0.1 (-0.5,0.3)	0.2 (0.1,0.3)	0.4 (0.2,0.5)	-3.5 (-3.7,-3.4)	-1.2 (-1.4,-1.1)
**H**	**H**	**H**	**L**	-5.8 (-5.9,-5.7)	21.9 (21.5,22.3)	22.2 (21.8,22.6)	-1.9 (-2.2,-1.5)	0.8 (0.4,1.2)	0.1 (0.0,0.2)	0.1 (0.0,0.2)	-6.0 (-6.1,-5.8)	-1.3 (-1.5,-1.2)
**H**	**H**	**H**	**H**	-4.0 (-4.2,-3.9)	15.3 (14.7,15.8)	15.3 (14.8,15.8)	-1.2 (-1.5,-0.9)	1.4 (1.0,1.7)	0.2 (0.1,0.3)	0.2 (0.1,0.3)	-4.1 (-4.3,-4.0)	-2.2 (-2.3,-2.0)
**L**	**L**	**L**	**L**	-1.2 (-1.8,-0.6)	18.9 (18.0,19.8)	18.9 (18.0,19.8)	-13.7 (-14.6,-12.8)	0.0 (-0.8,0.9)	3.9 (3.5,4.2)	3.9 (3.5,4.3)	-4.1 (-4.4,-3.7)	-0.5 (-0.8,-0.2)
**L**	**L**	**H**	**L**	-6.5 (-6.7,-6.3)	26.0 (25.5,26.6)	25.7 (25.2,26.2)	-15.2 (-15.9,-14.5)	3.5 (2.9,4.1)	2.4 (2.2,2.6)	2.3 (2.2,2.5)	-8.6 (-8.8,-8.4)	-1.1 (-1.3,-0.9)
**L**	**L**	**H**	**H**	-0.8 (-1.1,-0.5)	5.3 (4.6,5.9)	5.0 (4.3,5.6)	-5.4 (-5.9,-4.9)	4.6 (3.9,5.2)	0.6 (0.4,0.8)	0.8 (0.6,1.0)	-2.3 (-2.6,-2.1)	0.3 (0.2,0.5)

**L**	**H**	**L**	**H**	-4.8 (-5.0,-4.6)	17.9 (17.4,18.4)	19.2 (18.6,19.9)	-1.4 (-1.8,-1.1)	-0.7 (-1.3,-0.2)	1.8 (1.6,2.0)	0.2 (0.0,0.3)	-2.9 (-3.1,-2.7)	-3.2 (-3.3,-3.0)
**Sensitivity: L L H H**												
**Specificity**				π	Se_1	Se_2	Se_3	Se_4	Sp_1	Sp_2	Sp_3	Sp_4
**H**	**H**	**L**	**L**	-5.2 (-5.3,-5.0)	20.1 (19.7,20.6)	20.1 (19.6,20.5)	-1.4 (-1.7,-1.1)	-1.5 (-1.8,-1.2)	0.3 (0.1,0.4)	0.3 (0.2,0.4)	-3.2 (-3.3,-3.0)	-3.4 (-3.5,-3.2)
**H**	**H**	**H**	**L**	-4.7 (-4.8,-4.5)	18.2 (17.8,18.7)	17.7 (17.2,18.1)	-1.6 (-1.9,-1.3)	-0.4 (-0.6,-0.1)	0.2 (0.1,0.3)	0.2 (0.1,0.3)	-4.8 (-4.9,-4.6)	-2.8 (-3.0,-2.7)
**H**	**H**	**H**	**H**	-1.3 (-1.5,-1.2)	5.4 (4.9,5.9)	5.3 (4.8,5.8)	-0.5 (-0.7,-0.2)	-0.7 (-1.0,-0.5)	0.2 (0.1,0.3)	0.3 (0.2,0.4)	-1.5 (-1.7,-1.4)	-1.6 (-1.7,-1.4)
**L**	**L**	**L**	**L**	-2.2 (-2.7,-1.8)	18.7 (17.8,19.5)	19.4 (18.5,20.2)	-6.9 (-7.5,-6.3)	-6.3 (-6.9,-5.7)	2.8 (2.6,3.0)	3.1 (2.9,3.4)	-2.8 (-3.1,-2.6)	-2.8 (-3.1,-2.5)
**L**	**L**	**H**	**L**	-0.3 (-0.7,0.1)	9.4 (8.6,10.2)	9.4 (8.6,10.2)	-8.6 (-9.1,-8.0)	-0.1 (-0.4,0.3)	1.5 (1.3,1.7)	1.5 (1.3,1.6)	-2.7 (-2.9,-2.4)	0.1 (-0.2,0.4)
**L**	**L**	**H**	**H**	2.1 (1.9,2.3)	0.4 (0.0,0.7)	0.2 (-0.1,0.6)	-4.3 (-4.7,-3.9)	-4.1 (-4.5,-3.7)	0.3 (0.2,0.5)	0.6 (0.4,0.7)	0.8 (0.7,1.0)	0.9 (0.7,1.0)
**L**	**H**	**L**	**H**	-3.9 (-4.2,-3.7)	14.5 (13.9,15.0)	16.5 (15.8,17.2)	0.3 (0.0,0.6)	-2.5 (-2.69,-2.1)	0.3 (0.2,0.4)	0.3 (0.2,0.4)	-2.3 (-2.5,-2.1)	-4.3 (-4.5,-4.1)

Table xii.5 Coverages of the 95% CrIs for each model parameter

**Sensitivity: H H L L**												
**Specificity**				π	Se_1	Se_2	Se_3	Se_4	Sp_1	Sp_2	Sp_3	Sp_4
**H**	**H**	**L**	**L**	95.7 (94.4,97.0)	99.9 (99.7,100.0)	100.0 (100.0,100.0)	93.8 (92.2,95.3)	94.8 (93.4,96.3)	95.1 (93.7,96.5)	93.8 (92.2,95.3)	96.0 (94.8,97.3)	94.4 (92.9,95.9)
**H**	**H**	**H**	**L**	96.9 (95.8,98.0)	96.1 (94.9,97.3)	96.2 (95.0,97.4)	94.7 (93.3,96.1)	94.8 (93.4,96.2)	95.7 (94.4,97.0)	95.4 (94.1,96.7)	94.9 (93.5,96.3)	94.1 (92.6,95.6)
**H**	**H**	**H**	**H**	95.1 (93.8,96.4)	89.1 (87.2,91.0)	89.6 (87.7,91.5)	95.4 (94.1,96.7)	95.0 (93.6,96.4)	95.4 (94.1,96.7)	95.5 (94.2,96.8)	94.1 (92.6,95.6)	93.9 (92.4,95.4)
**L**	**L**	**L**	**L**	88.0 (85.1,90.9)	99.6 (99.0,100.0)	99.2 (98.3,100.0)	94.5 (92.5,96.6)	94.9 (93.0,96.9)	91.6 (89.1,94.1)	90.1 (87.4,92.8)	95.6 (93.7,97.4)	94.1 (92.0,96.2)
**L**	**L**	**H**	**L**	97.1 (96.0,98.2)	99.7 (99.3,100.0)	99.0 (98.4,99.7)	94.6 (93.1,96.0)	94.9 (93.5,96.3)	95.1 (93.7,96.5)	95.3 (94.0,96.7)	97.4 (96.4,98.4)	96.4 (95.2,97.6)
**L**	**L**	**H**	**H**	96.3 (95.1,97.4)	96.8 (95.7,97.9)	97.2 (96.1,98.2)	96.0 (94.7,97.2)	95.5 (94.3,96.8)	95.5 (94.3,96.8)	94.3 (92.9,95.8)	95.4 (94.1,96.7)	95.6 (94.4,96.9)
**L**	**H**	**L**	**H**	96.8 (95.6,97.9)	94.7 (93.3,96.1)	99.8 (99.5,100.0)	96.5 (95.3,97.6)	95.0 (93.7,96.4)	94.4 (93.0,95.9)	96.1 (94.9,97.3)	95.9 (94.7,97.2)	96.0 (94.8,97.3)
**Sensitivity: H H H L**												
**Specificity**				π	Se_1	Se_2	Se_3	Se_4	Sp_1	Sp_2	Sp_3	Sp_4
**H**	**H**	**L**	**L**	97.1 (96.1,98.1)	99.1 (98.5,99.7)	98.4 (97.6,99.2)	93.5 (92.0,95.0)	93.8 (92.3,95.3)	96.2 (95.0,97.4)	95.6 (94.3,96.9)	94.8 (93.4,96.2)	94.5 (93.1,95.9)
**H**	**H**	**H**	**L**	93.8 (92.3,95.3)	83.5 (81.2,85.8)	83.7 (81.4,86.0)	94.7 (93.3,96.1)	94.8 (93.4,96.2)	95.9 (94.7,97.1)	95.2 (93.9,96.5)	90.9 (89.1,92.7)	94.3 (92.9,95.7)
**H**	**H**	**H**	**H**	92.6 (91.0,94.2)	80.7 (78.3,83.1)	81.7 (79.3,84.1)	95.2 (93.9,96.5)	94.2 (92.8,95.6)	95.7 (94.4,97.0)	95.8 (94.6,97.0)	89.3 (87.4,91.2)	92.4 (90.8,94.0)
**L**	**L**	**L**	**L**	94.3 (92.7,95.8)	99.3 (98.7,99.9)	99.1 (98.4,99.7)	93.8 (92.2,95.4)	95.6 (94.2,96.9)	93.7 (92.1,95.3)	92.9 (91.2,94.6)	97.8 (96.8,98.8)	96.3 (95.0,97.5)
**L**	**L**	**H**	**L**	98.1 (97.2,98.9)	95.4 (94.2,96.7)	94.9 (93.6,96.3)	95.7 (94.4,96.9)	96.1 (94.8,97.3)	95.2 (93.9,96.6)	93.9 (92.4,95.4)	93.3 (91.8,94.9)	96.7 (95.5,97.8)
**L**	**L**	**H**	**H**	97.0 (95.9,98.1)	89.3 (87.4,91.2)	89.9 (88.0,91.8)	96.9 (95.8,98.0)	93.8 (92.3,95.3)	95.4 (94.1,96.7)	95.4 (94.1,96.7)	91.6 (89.9,93.3)	94.4 (93.0,95.8)
**L**	**H**	**L**	**H**	96.7 (95.6,97.8)	89.0 (87.0,90.9)	99.4 (98.9,99.9)	94.3 (92.8,95.7)	93.8 (92.3,95.3)	95.5 (94.2,96.8)	96.6 (95.5,97.7)	94.6 (93.2,96.0)	94.3 (92.8,95.7)
**Sensitivity: H H H H**												
**Specificity**				π	Se_1	Se_2	Se_3	Se_4	Sp_1	Sp_2	Sp_3	Sp_4
**H**	**H**	**L**	**L**	95.0 (93.6,96.4)	92.2 (90.5,93.9)	92.4 (90.8,94.0)	94.2 (92.8,95.6)	95.5 (94.2,96.8)	96.4 (95.2,97.6)	95.3 (94.0,96.8)	94.5 (93.1,95.9)	93.9 (92.4,95.4)
**H**	**H**	**H**	**L**	93.0 (91.4,94.6)	79.1 (76.6,81.6)	80.9 (78.4,83.3)	95.5 (94.2,96.8)	94.1 (92.6,95.6)	95.3 (94.0,96.6)	95.5 (94.2,96.8)	89.5 (87.6,91.4)	92.9 (91.3,94.5)
**H**	**H**	**H**	**H**	92.0 (90.3,93.7)	79.5 (77.0,82.0)	76.0 (73.7,78.6)	94.3 (92.9,95.7)	93.6 (92.1,95.1)	94.3 (92.9,95.7)	95.0 (93.6,96.4)	89.9 (88.0,91.8)	88.3 (86.3,90.3)

**L**	**L**	**L**	**L**	97.5 (96.6,98.5)	99.0 (98.3,99.6)	99.7 (99.3,100.0)	96.0 (94.8,97.2)	95.2 (93.8,96.5)	94.2 (92.7,95.6)	95.1 (93.7,96.4)	96.6 (95.5,97.8)	95.9 (94.7,97.1)
**L**	**L**	**H**	**L**	98.6 (97.9,99.3)	90.0 (88.1,91.9)	89.2 (87.3,91.1)	97.1 (96.1,98.1)	96.4 (95.2,97.6)	95.0 (93.6,96.3)	94.8 (93.4,96.2)	90.2 (88.3,92.0)	93.9 (92.4,95.4)
**L**	**L**	**H**	**H**	95.3 (94.0,96.6)	86.7 (84.6,88.8)	86.7 (84.6,88.8)	97.2 (96.2,98.2)	96.8 (95.7,97.9)	95.4 (94.1,96.7)	95.1 (93.8,96.4)	91.3 (89.6,93.0)	92.0 (90.3,93.7)
**L**	**H**	**L**	**H**	94.9 (93.5,96.3)	79.5 (77.0,82.0)	92.4 (90.8,94.0)	95.9 (94.7,97.1)	97.2 (96.2,98.2)	94.8 (93.4,96.2)	95.7 (94.4,97.0)	95.3 (94.0,96.6)	91.0 (89.2,92.8
**Sensitivity: L L L L**												
**Specificity**				$\boldsymbol{\pi}$	$\boldsymbol{Se_1}$	$\boldsymbol{Se_2}$	$\boldsymbol{Se_3}$	$\boldsymbol{Se_4}$	$\boldsymbol{Sp_1}$	$\boldsymbol{Sp_2}$	$\boldsymbol{Sp_3}$	$\boldsymbol{Sp_4}$
**H**	**H**	**L**	**L**	96.2 (94.8,97.5)	79.1 (76.2,81.9)	79.1 (76.2,81.9)	95.0 (93.5,96.5)	96.0 (94.7,97.4)	97.4 (96.3,98.6)	97.2 (96.0,98.3)	93.9 (92.2,95.6)	93.0 (91.2,94.8)
**H**	**H**	**H**	**L**	66.9 (64.0,69.9)	38.8 (35.8,41.9)	40.9 (37.8,44.0)	95.5 (94.1,96.8)	96.0 (94.7,97.2)	96.1 (94.9,97.3)	95.7 (94.4,96.9)	50.8 (47.7,54.0)	91.8 (90.1,93.6)
**H**	**H**	**H**	**H**	60.5 (57.5,63.6)	37.5 (34.5,40.5)	39.3 (36.3,42.3)	95.7 (94.4,96.9)	96.2 (95.0,97.4)	95.4 (94.0,96.7)	94.8 (93.5,96.2)	65.9 (62.9,68.8)	63.4 (60.4,66.4)
**L**	**L**	**L**	**L**	100.0 (100.0,100.0)	96.0 (93.5,98.4)	95.6 (93.0,98.1)	98.0 (96.2,99.7)	98.8 (97.4,100.0)	88.3 (84.3,92.3)	89.9 (86.2,93.7)	99.2 (98.1,100.0)	98.0 (96.2,99.7)
**L**	**L**	**H**	**L**	97.0 (95.5,98.4)	63.3 (59.3,67.4)	63.2 (59.1,67.3)	93.6 (91.5,95.7)	97.7 (96.5,99.0)	91.0 (88.5,93.4)	90.4 (87.9,92.9)	62.8 (58.7,66.9)	96.2 (94.6,97.9)
**L**	**L**	**H**	**H**	91.2 (89.2,93.3)	74.5 (71.4,77.7)	77.7 (74.7,80.7)	97.3 (96.1,98.4)	97.9 (96.9,99.0)	95.5 (94.0,97.0)	96.3 (94.9,97.7)	85.6 (83.1,88.2)	86.6 (84.1,89.0)
**L**	**H**	**L**	**H**	81.9 (79.4,84.5)	34.5 (31.3,37.7)	59.1 (55.8,62.4)	97.3 (96.2,98.4)	96.4 (95.1,97.6)	91.9 (90.1,93.7)	96.8 (95.7,98.0)	91.8 (90.0,93.6)	59.7 (56.4,63.0)
**Sensitivity: L L H L**												
**Specificity**				$\boldsymbol{\pi}$	$\boldsymbol{Se_1}$	$\boldsymbol{Se_2}$	$\boldsymbol{Se_3}$	$\boldsymbol{Se_4}$	$\boldsymbol{Sp_1}$	$\boldsymbol{Sp_2}$	$\boldsymbol{Sp_3}$	$\boldsymbol{Sp_4}$
**H**	**H**	**L**	**L**	68.0 (65.1,71.0)	40.4 (37.3,43.5)	38.1 (35.1,41.2)	95.1 (93.7,96.4)	96.4 (95.2,97.6)	97.7 (96.8,98.7)	96.1 (94.9,97.3)	75.7 (73.0,78.4)	92.2 (90.5,93.9)
**H**	**H**	**H**	**L**	37.9 (34.9,40.9)	18.2 (15.8,20.6)	16.9 (14.9,19.2)	96.1 (94.9,97.3)	94.8 (93.4,96.2)	95.5 (94.2,96.8)	96.6 (95.5,97.7)	15.4 (13.2,17.6)	91.2 (89.4,93.0)
**H**	**H**	**H**	**H**	63.7 (60.7,66.7)	49.1 (46.0,52.3)	46.2 (43.1,49.3)	96.3 (95.1,97.5)	95.1 (93.7,96.4)	95.4 (94.1,96.7)	94.7 (93.3,96.1)	49.9 (46.8,53.1)	76.4 (73.7,79.0)
**L**	**L**	**L**	**L**	99.5 (98.7,100.0)	75.5 (71.1,79.8)	75.5 (71.1,79.8)	91.3 (88.5,94.1)	97.6 (96.1,99.2)	88.1 (84.9,91.4)	86.5 (83.1,90.0)	89.4 (86.4,92.5)	96.6 (94.7,98.4)
**L**	**L**	**H**	**L**	87.5 (85.2,89.9)	31.6 (28.2,34.9)	33.6 (30.2,37.0)	93.8 (820,95.5)	96.5 (95.1,97.8)	89.8 (87.7,92.0)	90.9 (88.8,93.0)	22.6 (19.6,25.6)	94.3 (92.6,96.0)
**L**	**L**	**H**	**H**	95.5 (94.1,97.0)	85.9 (83.5,88.4)	88.0 (85.7,90.3)	98.3 (97.4,99.2)	96.9 (95.7,98.1)	96.5 (95.3,97.8)	94.2 (92.6,95.9)	89.5 (87.4,91.7)	95.7 (94.2,97.1)
**L**	**H**	**L**	**H**	80.7 (78.2,83.2)	40.7 (37.6,43.9)	62.9 (59.8,66.0)	97.8 (96.9,98.8)	96.4 (95.3,97.6)	89.1 (87.1,91.1)	96.1 (94.9,97.4)	83.7 (81.4,86.1)	68.8 (65.8,71.7)
**Sensitivity: L L H H**												
**Specificity**				$\boldsymbol{\pi}$	$\boldsymbol{Se_1}$	$\boldsymbol{Se_2}$	$\boldsymbol{Se_3}$	$\boldsymbol{Se_4}$	$\boldsymbol{Sp_1}$	$\boldsymbol{Sp_2}$	$\boldsymbol{Sp_3}$	$\boldsymbol{Sp_4}$
**H**	**H**	**L**	**L**	54.9 (51.8,58.0)	32.7 (29.8,35.6)	31.4 (28.5,34.3)	94.7 (93.3,96.1)	94.3 (92.8,95.7)	95.6 (94.3,96.9)	95.4 (94.1,96.7)	77.3 (74.7,79.9)	74.4 (71.7,77.1)
**H**	**H**	**H**	**L**	54.9 (51.8,58.0)	33.6 (30.6,36.5)	35.9 (32.9,38.8)	95.8 (94.5,97.0)	97.1 (96.1,98.1)	96.2 (95.0,97.4)	95.2 (93.9,96.5)	35.3 (32.3,38.2)	79.2 (76.6,81.7)
**H**	**H**	**H**	**H**	90.2 (88.3,92.0)	82.1 (79.8,84.5)	83.0 (80.7,85.4)	96.1 (94.9,97.3)	95.1 (93.7,96.4)	95.1 (93.7,96.4)	94.6 (93.2,96.0)	85.3 (83.1,87.5)	83.6 (81.2,85.9)
**L**	**L**	**L**	**L**	97.6 (96.3,98.8)	59.7 (55.7,63.7)	58.3 (54.3,62.4)	95.9 (94.3,97.5)	95.9 (94.3,97.5)	89.2 (86.6,91.7)	83.5 (80.5,86.5)	88.1 (85.5,90.8)	86.9 (84.2,89.7)
**L**	**L**	**H**	**L**	95.5 (93.9,97.0)	78.5 (75.6,81.5)	78.3 (75.3,81.3)	98.1 (97.1,99.1)	99.7 (99.3,100.0)	92.2 (90.2,94.1)	92.8 (91.0,94.7)	83.8 (81.1,86.4)	95.2 (93.6,96.7)
**L**	**L**	**H**	**H**	97.1 (96.1,98.2)	93.6 (92.0,95.2)	94.9 (93.5,96.4)	98.3 (97.5,99.2)	98.3 (97.5,99.2)	95.5 (94.1,96.8)	94.6 (93.1,96.1)	98.0 (97.1,98.9)	97.8 (96.8,98.8)
**L**	**H**	**L**	**H**	75.6 (72.9,78.4)	50.3 (47.1,53.5)	58.9 (55.8,62.0)	97.6 (96.6,98.5)	97.2 (96.2,98.3)	92.9 (91.3,94.5)	96.3 (95.1,97.5)	85.2 (82.9,87.4)	60.4 (57.3,63.5)
