# Supplementary material for: Detecting departures from the conditional independence assumption in diagnostic latent class models: a simulation study
Source: BMC Med Res Methodol. 2024 Dec 5;24:299. doi: 10.1186/s12874-024-02432-x (PMC11619692; doi:10.1186/s12874-024-02432-x)
Supplement: Supplementary file 4 — Additional file 4: Median absolute biases and 95% CrI coverages summarised across all converged data sets (of the 504,000), across all converged data sets with each value of prevalence ($\pi$π) or correlation ($\omega$ω), and across all converged data sets with each sample size. [file 12874_2024_2432_MOESM4_ESM.pdf]

Median absolute biases and 95% CrI coverages summarised across all converged data sets (of the 504,000), across all converged data sets with each value of prevalence (π) or correlation (ω), and across all converged data sets with each sample size

Median of absolute biases (x10^{-2}) of posterior medians of each parameter in different scenarios

	Parameter								
**Scenarios**	π	Se_1	Se_2	Se_3	Se_4	Sp_1	Sp_2	Sp_3	Sp_4
**All**	-4.8 (-23.5,2.8)	9.4 (-0.3,39.7)	9.4 (-0.3,39.7)	-0.5 (-12.8,5.5)	-0.2 (-8.6,7.1)	1.1 (-2.5,13.3)	0.9 (-2.5,13.2)	-4.3 (-25.0,1.6)	-2.9 (-17.8,2.1)
$\pi = 0.5$	-6.3 (-26.3,0.7)	9.6 (1.5,39.8)	9.7 (1.5,39.8)	-0.5 (-10.3,4.1)	-0.2 (-6.9,5.5)	1.8 (-2.7,14.5)	1.4 (-2.8,14.4)	-7.2 (-26.9,0.4)	-5.2 (-21.7,1.2)
$\pi = 0.2$	-2.4 (-10.0,4.1)	9.0 (-1.8,39.3)	9.0 (-2.1,39.4)	-0.7 (-15.4,6.5)	-0.2 (-10.1,8.6)	0.7 (-2.3,6.0)	0.6 (-2.3,5.8)	-2.0 (-9.0,2.1)	-1.5 (-7.1,2.5)
$\omega = 0.9$	-5.6 (-21.6,2.4)	9.8 (2.4,39.8)	9.8 (2.1,39.8)	-0.9 (-14.2,4.8)	-0.6 (-9.2,6.1)	1.8 (-1.5,14.4)	1.4 (-1.6,14.3)	-5.2 (-25.9,1.2)	-3.9 (-20.4,1.7)
$\omega = 0.5$	-3.9 (-26.3,3.1)	6.7 (-1.5,37.0)	7.0 (-1.7,37.5)	-0.1 (-10.8,6.1)	0.3 (-7.8,8.1)	0.6 (-3.0,6.6)	0.4 (-3.2,6.2)	-3.5 (-19.9,1.9)	-2.3 (-16.6,2.4)
$n_{obs} = 5000$	-5.1 (-25.6,0.0)	9.7 (2.2,39.8)	9.7 (2.2,39.9)	-0.3 (-10.1,3.7)	0.0 (-5.3,4.8)	0.9 (-1.0,13.1)	0.8 (-1.1,13.1)	-4.4 (-25.0,0.2)	-2.9 (-17.4,0.5)
$n_{obs} = 2000$	-4.9 (-24.0,1.2)	9.5 (1.1,39.6)	9.5 (1.1,39.7)	-0.5 (-11.5,4.9)	-0.1 (-6.9,6.1)	1.1 (-1.7,13.1)	0.9 (-1.8,13.1)	-4.3 (-24.9,0.9)	-3.0 (-17.5,1.3)
$n_{obs} = 500$	-4.2 (-22.6,5.4)	8.7 (-3.2,38.8)	8.7 (-3.6,38.9)	-1.2 (-15.9,7.1)	-0.6 (-11.5,9.9)	1.5 (-3.8,14.1)	1.3 (-3.9,13.8)	-4.2 (-25.1,3.0)	-3.0 (-18.4,3.6)

Posterior medians were obtained from the CInd model. Median biases are shown with 2.5th and 97.5th percentiles.

Coverages of 95% CrIs for each model parameters in different scenarios

	Parameter								
**Scenarios**	π	Se_1	Se_2	Se_3	Se_4	Sp_1	Sp_2	Sp_3	Sp_4
**All**	37.3 (37.2,37.5)	17.6 (17.5,17.8)	18.5 (18.4,18.6)	88.3 (88.3,88.4)	91.1 (91.0,91.2)	72.8 (72.7,72.9)	77.3 (77.2,77.4)	35.5 (35.3,35.6)	46.5 (46.4,46.6)
$\pi = 0.5$	22.5 (22.3,22.7)	8.8 (8.7,8.9)	9.0 (8.9,9.1)	84.7 (84.6,84.9)	88.9 (88.7,89.0)	63.1 (63.0,63.3)	68.9 (68.7,69.1)	19.6 (19.4,19.7)	30.4 (30.2,30.5)
$\pi = 0.2$	52.5 (52.3,52.7)	26.6 (26.5,26.8)	28.1 (27.9,28.3)	92.0 (91.9,92.2)	93.4 (93.3,93.5)	82.7 (82.5,82.8)	85.8 (85.7,85.9)	51.7 (51.5,51.9)	63.0 (62.8,63.2)
$\omega = 0.9$	29.3 (29.1,29.4)	8.9 (8.8,9.0)	9.5 (9.4,9.6)	83.7 (83.6,83.9)	89.5 (89.3,89.6)	60.0 (59.8,60.2)	65.7 (65.5,65.9)	28.4 (28.2,28.6)	38.8 (38.6,39.0)
$\omega = 0.5$	45.9 (45.7,46.1)	26.9 (26.7,27.1)	28.0 (27.8,28.2)	93.2 (93.1,93.3)	92.9 (92.8,93.0)	86.3 (86.2,86.5)	89.5 (89.4,89.7)	43.0 (42.8,43.2)	54.6 (54.4,54.8)
$n_{obs} = 5000$	18.4 (18.2,18.6)	5.0 (4.9,5.1)	5.3 (5.2,5.4)	84.5 (84.3,84.7)	87.8 (87.7,88.0)	62.9 (62.6,63.1)	68.7 (68.4,68.9)	19.9 (19.7,20.1)	30.5 (30.2,30.7)
$n_{obs} = 2000$	33.2 (32.9,33.4)	11.9 (11.8,12.1)	12.7 (12.5,12.8)	88.2 (88.1,88.4)	91.3 (91.2,91.5)	72.4 (72.2,72.7)	77.2 (77.0,77.4)	31.9 (31.6,32.1)	43.8 (43.6,44.1)
$n_{obs} = 500$	60.8 (60.6,61.1)	36.2 (36.0,36.5)	37.7 (37.5,38.0)	92.4 (92.2,92.5)	94.2 (94.1,94.3)	83.3 (83.1,83.5)	86.1 (85.9,86.3)	55.0 (54.7,55.2)	65.5 (65.3,65.7)

Coverages are shown with 95% MC CIs
